# Supplementary material for: Has working-age morbidity been declining? Changes over time in survey measures of general health, chronic diseases, symptoms and biomarkers in England 1994–2014
Source: BMJ Open. 2020 Mar 15;10(3):e032378. doi: 10.1136/bmjopen-2019-032378 (PMC7073795; doi:10.1136/bmjopen-2019-032378)
Supplement: Supplementary data [file bmjopen-2019-032378supp001.pdf]

WEB APPENDICES

Table of contents *(pages refer to within web appendices)*

Appendix 1: Working-age mortality trends 1

Appendix 2: Overall missingness in health measures 2

Appendix 3: Changing non-response & weights 4

Appendix 4: General self-reported health/disability 8

Appendix 5: Health measures 10

Appendix 6: Measures not included in the main paper 23

Appendix 7: Year-by-year trends 30

Appendix 8: Others' analyses over change over time using HSE data 34

Appendix 9: Summarising multiple measures 36

Bibliography for Web Appendices 41

Appendix 1: Working-age mortality trends

Mortality in general

Given debates about whether historic improvements in life expectancy are being sustained, particularly in the US and UK, [1 2] it is important to note that in the period under study in this paper, working-age life expectancy was increasing. This can be seen in data from the Human Mortality Database (May 2016 update) 1993-2013, using one-year age and one-year period. This data shows that increases in mortality are not found for working-age people as a whole in any major country – for example, standardised working-age death rates have declined by 23% in the US and 35% in the UK over 1993-2013.

Cause-specific mortality for the 0-64 population

The main text refers to cause-specific morality in several places, referring to the death rate among 064 year olds from cardiovascular disease (CVD), respiratory conditions, diabetes, and liver cirrhosis. These death rates refer to UK deaths within relevant ICD-10 codes (I00-I99 for CVD, J00-J99 for respiratory conditions, E10-E14 for diabetes), standardised to the European standard population, and taken from the World Health Organization European Office's Health for All Database (May 2016 version), http://www.euro.who.int/en/data-and-evidence/databases/european-health-for-all-databasehfa-db.

Appendix 2: Overall missingness in health measures

This appendix refers to overall item-level missi

ngness; changing item- and unit-level missingness is covered in Appendix 3.

Interview measures

For those who took part in the initial face-to-face interview, the level of item missingness is shown below (including only those years in which each question was asked). This shows the itemmissingness is generally very low – only 1 of the 30 measures variables have item-missingness greater than 1%.

Table 1: Missingness at the initial face-to-face interview

	n non-missing	*n* missing	% missingness
BMI	124,682	15,415	11.0%
Any recent CVD	43,274	354	0.8%
Recent high blood pressure	43,366	262	0.6%
Breathlessness-Grade 2+	25,620	68	0.3%
Breathlessness-Grade 3	25,620	68	0.3%
Recent heart attack/stroke	43,519	109	0.3%
COPD symptoms	25,631	57	0.2%
Recent angina	43,551	77	0.2%
Heart attack symptoms	43,595	33	0.1%
Angina symptoms	43,592	36	0.1%
Recent diabetes	66,637	54	0.1%
Mini stroke (TIA) symptoms	23,487	16	0.1%
Diagnosed asthma	41,225	28	0.1%
Wheezing stopping sleep	41,224	29	0.1%
Recent wheezing/asthma	41,224	29	0.1%
Locomotor limitation	25,347	10	0.0%
Self-care limitation	25,347	10	0.0%
Limitations in past 2wks	140,041	56	0.0%
Longstanding illness (LSI)	124,906	43	0.0%
Limiting LSI (LLSI)	104,798	36	0.0%
Any CVD LSI	124,912	37	0.0%
IHD/stroke LSI	124,912	37	0.0%
Mental health LSI	124,912	37	0.0%
Arthritis LSI	124,912	37	0.0%
Asthma LSI	124,912	37	0.0%
Diabetes LSI	124,912	37	0.0%
High blood pressure LSI	124,912	37	0.0%
Other musculoskeletal LSI	124,912	37	0.0%
Good general health	140,048	49	0.0%
Bad general health	140,048	49	0.0%

The only variable with noticeable missingness is BMI, which is understandable as this involves the interviewer taking height and weight measurements rather than simply asking for a verbal response. There are various reasons why people do not have a BMI measurement:

- *High weight*: people with a very high weight are not weighed in HSE 'because the scales are inaccurate above this level', but the definition of this changed (from 130kg before 2011 to 200kg afterwards). This only applied to <0.1% of respondents 2012-14.
- *Difficult to take measurement*: other respondents (between 3.8% and 6.1% depending on the year) have no valid BMI measurement because height or weight measures were not attempted, attempted but not obtained or useable, because the respondent was pregnant, or the respondent was too sick or unsteady.
- *Refusal*: the most common reason for no BMI measurement is an outright refusal (including those refusing out of anxiety, though this tends to be a minor reason). Refusal rates are 8.3% in 2014.

Self-completion measures

For those who completed the self-completion booklet, the level of item missingness is shown in the table below.

Table 2: Missingness within the self-completion booklet

	n *non-missing*	*n* *missing*	*%* *missingness*
Psychological distress symptoms	108,324	2,462	2.2%
Problems washing/dressing today	62,703	1,310	2.1%
Anxiety/depression	62,725	1,288	2.0%
Problems w/activities	62,742	1,271	2.0%
Problems walking about today	62,772	1,241	1.9%
Pain	62,783	1,230	1.9%

Item missingness is relatively low compared to missingness from not completing the self-completion survey (51.5% of respondents in 2014).

Nurse visit measures

For those who took part in the nurse visit, the level of item missingness is shown in the table below.

Table 3: Missingness within the nurse visit

	n *non-missing*	*n* *missing*	*%* *missingness*
Biomarker high blood pressure	87,726	15,517	15.0%
High waist-hip ratio	78,637	2,664	3.3%

This shows that far more people have missing observations for measured high blood pressure than for their waist-hip ratio. This is despite the fact that we explicitly INCLUDE those who are on blood pressure-lowering drugs (about 5% of the sample at the start of the period and 10% at the end), on the grounds that their lowered blood pressure still conveys useful information about their health state. The main reason for the remaining high level of missingness is because people have recently exercised, smoked, drank or ate (12.2%).

Blood sample measures

For those from whom a blood sample was taken, the level of item missingness is shown in the table below.

Geiger BB. *BMJ Open* 2021; 10:e032378. doi: 10.1136/bmjopen-2019-032378

Table 4: Missingness within the blood sample

	n *non-missing*	*n* *missing*	*%* *missingness*
Raised fibrinogen	16,166	3,341	17.1%
Raised C-reactive protein	17,814	1,693	8.7%
Glycated haemoglobin	28,810	1,436	4.8%
Anaemia	20,302	939	4.4%
Iron deficiency	20,375	866	4.1%
Low HDL cholesterol	36,076	1,406	3.8%
High total cholesterol	43,409	1,472	3.3%

All of these measures are affected by problems in transferring and storing the blood sample and with the measurement process, which results in problems with 3-10% of the blood samples depending on the measure and year. As for blood pressure, we explicitly INCLUDE those who are on lipidlowering drugs (0.4% 1994 to 7.9% 2014), on the grounds that their changed cholesterol level still conveys useful information about their health state. Item missingness is highest for fibrinogen, which not only has high rates of such failures (7.0-9.5%), but also has ineligibility due to likely infection (from raised CRP, 3.6-5.6% of those with blood samples) and taking drugs that affect the reading (3.7% to 7.7% dependent on the year). Item missingness is also high for C-reactive protein (CRP), which also excludes those with likely infections.

Dealing with item-level missingness

Because of the high level of item non-response for certain measures (BMI, high blood pressure, fibrinogen, and CRP), and moderate level for others (other blood sample biomarkers and waist-hip ratio) – and because of evidence of changing non-response at various stages of the survey process – non-response weights were created to try to correct for any biases that these introduce. This is described in further detail in Appendix 3.

Appendix 3: Changing non-response & weights

This appendix focuses on *changes* in unit-level non-response at different stages of HSE.

Changing non-response

Sample frame coverage

As noted in the main paper, HSE is a household sample that excludes those in communal establishments. If we combine data from the 1991, 2001 and 2011 Censuses,[1] the communal population is as follows:

Table 1: Population in communal establishments over time (all working-age) and by age (in 2011)

		Education	Medical/ care	Defence	Prison	Other / not stated
**All working age**	*1991*	21,149	86,683	44,562	13,279	63,340
	2001	204,606	73,705	46,428	44,185	86,288

	2011	328,772	76,026	41,659	47,849	61,124
**16-24**	*2011*	305,154	9,346	22,677	12,607	25,673
**25-34**	*2011*	20,443	12,000	15,025	15,407	14,417
**35-49**	*2011*	2,663	26,796	3,725	14,725	14,708
**50-SPA[1] (est)**	*2011*	512	27,884	232	5,110	6,326

1 SPA = State Pension Age, which is 60 for women and 65 for men. This is estimated because the Census totals are given for 50-64 year olds, so we have excluded 1/3 of women aged 50-64 from these totals.

This shows two things. Firstly, that there was a sharp rise in the working-age population in communal establishments 1991-2001 (from 230k to 560k), which was concentrated (>90% of the rise) among education-related communal establishments – although this is perhaps a slight overestimate given a definition change in the Census data.[2] Secondly, looking at education-related communal establishments in 2011, these are overwhelmingly (>90%) among 16-24 year olds. It therefore seems likely that the exclusion of communal establishments in HSE will lead to biases in young adults, and we therefore exclude 16-24 year olds from the trend analyses.

Changing unit non-response within the sample frame

As noted in the main paper, HSE supplies non-response weights from 2003, including adjustments for non-response to the nurse visit and blood sample using health and socioeconomic status from the initial interview. However, there had been a substantial decline in response rates prior to 2003, as shown in the table below:

Table 2: Response rates to HSE

	Household	Individual	Self-comp.	BMI	Nurse	Blood
1991	85.3%	81.1%				
1992	81.8%	77.4%				
1993	80.8%	75.7%				
1994	77.4%	71.6%	71.2%	67.1%	63.3%	53.3%
1995	78.3%	72.9%	72.0%	66.8%	63.7%	
1996	79.4%	74.7%	73.7%	69.6%	66.1%	
1997	76.0%	71.1%	69.8%	66.9%	64.0%	
1998	74.0%	68.9%	66.7%	63.3%	59.6%	49.0%
1999	76.2%	70.3%	68.5%	63.6%		
2000	75.5%	68.4%	65.8%	60.5%	58.2%	

1 Data are obtained from nomis on 6/8/2015, from Census tables DC1104EW and DC4210EWla (2011), S126 (2011) and L03/L04/L05 (2001).

2 The guide to Census SARs notes, *"In the 1991 Census, students and schoolchildren were treated as usually resident at their 'home' or vacation address. In the 2001 census students and schoolchildren in full-time education studying away from the family home were enumerated as resident at their term-time address."* See https://census.ukdataservice.ac.uk/use-data/guides/microdata/comparability-91-01 [accessed 1/11/2016].

2001	74.2%	67.1%	64.5%	60.1%	54.2%	
2002	74 %	67 %	64.4%	59.6%	54.3%	
2003	72.7%	66.4%	64.1%	59.7%	52.2%	39.9%
2004	72.4%	65.6%	62.4%	56.1%		
2005	71.4%	64.1%	60.6%	54.8%	46.7%	
2006	68.1%	60.5%	57.7%	52.8%	45.4%	34.7%
2007	65.7%	58.3%	56.1%	51.3%	42.6%	
2008	64.5%	57.9%	55.9%	50.0%	41.5%	30.4%
2009	67.6%	61.0%	58.7%	52.5%	43.1%	33.7%
2010	66.1%	58.7%	54.9%	49.3%	39.1%	29.9%
2011	65.7%	58.9%	54.3%	49.0%	39.4%	29.8%
2012	64.1%	56.3%	52.5%	47.4%	36.3%	27.9%
2013	63.8%	57.6%	54.2%	49.3%	40.1%	31.2%
2014	61.6%	55.5%	51.5%	48.4%	37.3%	28.7%

In general these trends are due to increases in refusal rates. However, the blood sample response rate is affected by two noticeable changes in eligibility over this period (people who are pregnant or who had blood/clotting disorders were ineligible throughout):

1. In 1998, people who had ever had an epileptic fit were excluded from the blood sample. This raised the ineligibility rate to 3.5% of the sample in 1998, from 0.6% in 1994.
2. In 2010, this was then relaxed so that those who had had an epileptic fit more than 5 years ago were again included in the blood sample. This lowered the ineligibility rate from 3.1% in 2009 to 2.4% in 2010.

Changing item non-response within responding people

There are also changes over time in item non-response (further detail on overall item non-response is given in Appendix 2). This includes:

- *BMI:* there has been little systematic trend in one reason for the absence of a BMI measure (difficulty in taking BMI measurements). However, there are trends in other reasons:
    - *High weight*: the definition of high weight changed from 130kg before 2011 to 200kg afterwards. 1.0% of respondents were not weighted for this reason in 2010, which fell to <0.1% 2012-14.
    - *Refusal*: in line with the general participation rates at each stage of the interview above, BMI refusal rates rose sharply from 1.9% in 1994 to a peak of 11.5% in 2011, and remain at 8.3% in the 2014 data.

- *Psychological distress:* similarly to wider participation rates at each stage of the survey, item missingness within the self-completion survey does increase over time (e.g. for psychological distress symptoms, from 1.8% 1994 to 5.9% 2014).
- *Measured high blood pressure:* there was a noticeable rise over time in exclusion of high blood pressure measures on the grounds that people recently exercised, smoked, drank or ate (from 6.1% to 13.6%).
- *Fibrinogen:* taking drugs that affect the fibrinogen reading rose from 3.7% 1994 to 7.7% 2009.

Creating non-response weights

To increase comparability over time, we create new weights 1994-2014 in several phases.

First-stage non-response weights

Firstly, we created a selection weight because some households were slightly more likely to be interviewed than others. (Until 2009, only three households at each address were interviewed. Those living at addresses with many households are therefore less likely to be interviewed). NatCen supplied selection weights for 2004-2013 to enable this (funded by this project), which are not available on the public HSE datasets.

Secondly, after adjusting for the selection weight, we created new individual-level (inverse probability) weights to match population age-sex-region totals in each year. Population data are annual mid-year population estimates from nomis. NatCen added the region variable for the 19941997 datasets to the public HSE datasets to enable this.

Second-stage non-response weights

After the first-stage adjustment for individual non-response, for the later stages of the interview (self-completion, BMI measurement, nurse visit, blood sample), we created a further weight that adjusts for non-response among those responding to the individual interview. This is based on a logit regression model to predict that stage of response based on:

- Age and gender (4 age group categories interacted with gender);
- Qualifications (degree or FT student / A-level or above / other qualifications / no qualifications);
- Household type (presence of other adults in the household);
- Employment status (yes/no);
- Smoking (never regular smoker / ex-regular smoker / current regular smoker); and
- Self-reported general health (bad or very bad health vs. other categories).

On the basis of these criteria, we create inverse probability weights – that is, we create a predicted probability of response for each respondent based on the logit regression model, and then create a weight that is the inverse of this predicted probability. The revised weights are included in the Stata code to enable replication of the full paper.

Final sample size

The final sample size is as follows:

Table 3: HSE sample size in each year

	Interview	Self-completion	Nurse visit	Blood sample
1994	9,948	9,884	8,786	7,399
1995	10,167	10,049	8,881	
1996	10,401	10,269	9,206	
1997	5,563	5,458	5,005	
1998	10,177	9,843	8,805	7,236
1999	5,008	4,884		
2000	5,188	4,993	4,417	
2001	10,002	9,613	8,079	
2002	4,662	4,482	3,775	
2003	9,420	9,089	7,395	5,665
2004	4,165	3,961		
2005	4,810	4,548	3,505	
2006	8,825	8,420	6,622	5,064
2007	4,198	4,039	3,064	
2008	9,242	8,922	6,625	4,845
2009	2,795	2,689	1,973	1,542
2010	5,120	4,794	3,411	2,610
2011	5,258	4,853	3,518	2,667
2012	4,936	4,605	3,188	2,447
2013	5,303	4,992	3,691	2,875
2014	4,909	4,552	[redacted]	2,531
**Total**	**140,097**	**134,939**	[redacted]	**44,881**

Appendix 4: General self-reported health/disability

Trends in seven general health/disability measure are available in HSE:

Table 1: HSE general health measures

Measure	Operationalisation (years available)
Good general health	Health in general is 'good' or 'very good' (1994-2014)
Bad general health	Health in general is 'bad' or 'very bad' (1994-2014)
Longstanding illness (LSI)	Any long-standing illness, disability or infirmity (1994-2011)
Limiting LSI (LLSI)	LSI limits activities in any way (1996-2011)
Problems with activities-some	Some problems with performing usual activities (1996-2014)
Problems with activities-unable	Unable to perform usual activities (1996-2014)
Limitations in past 2wks	Cut down on activities in past 2wks due to LSI or other illness/injury (1994-2014)

See Web Appendix 5 for full details on all measures .

Geiger BB. *BMJ Open* 2021; 10:e032378. doi: 10.1136/bmjopen-2019-032378

Trends for these measures are shown in Table 9 below. Looking first at good general health, the table shows the trend from 1994-6, when 80.9% reported good general health. By 2011-14, there had been a decline of 0.8 percentage points. When we adjust for the changing age and sex distribution of the working-age population (labelled 'Adj.' in Table 1), the decline is only 0.1%, with a wide confidence interval (-0.9 to +0.7%), and there is therefore little evidence for any systematic trend.

Table 2: Changes over time in general health

	Starting period		Change from start to end period			
	Period	Prevalence	End period	Raw change	Adj.[a] change	Adj. change 95% CI
Good general health	1994-96	80.9%	2011-14	-0.8%	-0.1%	[-0.9, 0.7%]
Bad general health	1994-96	4.4%	2011-14	1.3%	1.0%	[0.6, 1.5%]
Longstanding illness (LSI)	1994-96	36.2%	2011-14	-1.0%	-2.0%	[-3.7, -0.3%]
Limiting LSI (LLSI)	1994-96	21.4%	2011-14	-2.9%	-3.6%	[-5.2, -2.1%]
Problems w/activities-some	1994-96	14.8%	2011-14	-1.2%	-1.8%	[-2.8, -0.8%]
Problems w/activities-unable	1994-96	1.9%	2011-14	-0.6%	-0.8%	[-1.1, -0.4%]
Limitations in past 2wks	1994-96	14.7%	2011-14	-0.1%	-0.3%	[-1.0, 0.4%]

[a] 'Adj.' = adjusted for changing age and sex distribution of the working-age population.

For several of the general health measures, there is evidence of change over this period – but interpreting this is difficult, because the trends are in opposite directions. There is strong evidence for a *rise* in bad general health (a rise of 0.6-1.5% from a base of 4.4%), yet equally strong evidence for a *decline* in having problems with everyday activities (at both levels of severity), and being limited in activities by a longstanding illness. This shows the challenges in tracking population morbidity change through general, non-specific measures, which are likely to be as influenced by changes in reporting styles as much as changes in morbidity *per se*.

As an aside, UK Government publications have made claims based on healthy/disability-free life expectancy – sometimes using these to argue that morbidity has been improving [3], but more recently to argue that morbidity has been deteriorating.[4-6] However, these trends are potentially misleading: they include older people as well as the working-age population; they confuse a combined mortality-morbidity measure with morbidity; and they are based on self-reports of global health that are unreliable, as we show here and discuss in the main text.

Appendix 5: Health measures

We systematically searched HSE questions, and have included every morbidity measure that is comparable over a significant duration. We have excluded questions only available for short time frames (ADLs 2012-14, EQ-5D visual analogue scale 2008-14, SF-12 1996-2000, eczema/hayfever 1995-2001, breathlessness 1991-98 and 1995-2001, lung function 1995-2001, bladder limitations 1995-2001, LDL cholesterol, triglycerides and glucose 1999-2003, IgE 1996-2002 and an alternate measure of high blood pressure 2009-14), with the exception of five key measures of activity limitations 1995-2001. We have also excluded questions that are not direct measures of health (medication or health service use, demispan, health risk factors such as fractures, accidents, alcohol/tobacco use (including biomarkers), physical activity, and wellbeing).

Short summaries of the resulting 39 measures are given in this paper, and full details are given in the table below. Measures are taken from the initial face-to-face survey unless otherwise specified. The Stata code to create these variables in consistent form from the publicly available HSE files are available from OSF[7] and www.benbgeiger.co.uk.

Measure	Details
Activity limitations and MSDs	
Problems walking today	In the self-completion survey in 1996, 2003-6, 2008, 2010-12 and 2014, respondents were asked *'Now we would like to know how your health is today. Please answer ALL the questions. By ticking one box for each question below, please indicate which statements best describe your own health state today':*<br>- *"I have no problems in walking about"*<br>- *"I have some problems in walking about"* - *"I am confined to bed"*<br>[This is part of the widely-used EQ-5D health status indicator [8]. However, for the purposes of this paper we have separated the individual measures that make up the EQ5D in order to compare these to similar indicators of morbidity within each domain].<br>People are classified as having a problem with self-care today if they had some problems walking about or were confined to bed.
Locomotor limitation	This is based on the personal care disability scale used in the 2001 HSE report [9]. Respondents in 1995, 2000 and 2001 were asked if any of the following applied to them (interviewers were instructed to ignore temporary disabilities that are expected to last less than one year):<br>- *"Cannot walk 200 yards or more on own without stopping or discomfort"*. People who reported a limitation were asked if they used a walking aid, and if they did, were then asked if they could walk 200 yards without the walking aid.<br>- *"Cannot walk up and down a flight of 12 stairs without resting"*<br>- *"Cannot bend down and pick up a shoe from the floor when standing"*<br>People are classified as having a locomotor limitation if they reported ANY of these limitations.
Problems with washing/dressing today	In the self-completion survey in 1996, 2003-6, 2008, 2010-12 and 2014, respondents were asked *'Now we would like to know how your health is today. Please answer ALL the questions. By ticking one box for each question below, please indicate which statements best describe your own health state today':*<br>- *"I have no problems with self-care"*<br>- *"I have some problems washing or dressing myself"*<br>- *"I am unable to wash or dress myself"*

	[This is part of the widely-used EQ-5D health status indicator [8]. However, for the purposes of this paper we have separated the individual measures that make up the EQ-5D in order to compare these to similar indicators of morbidity within each domain].<br>People are classified as having a problem with self-care today if they had some problems washing/dressing or were unable to wash/dress themselves.
Self-care limitation	This is based on the personal care disability scale used in the 2001 HSE report [9]. Respondents in 1995, 2000 and 2001 were asked if any of the following applied to them (interviewers were instructed to ignore temporary disabilities that are expected to last less than one year):<br>- *"Cannot get in and out of bed on own without difficulty"*<br>- *"Cannot get in and out of a chair without difficulty"*<br>- *"Cannot dress and undress without difficulty"*<br>- *"Cannot wash hands and face without difficulty"*<br>- *"Cannot feed, including cutting up food without difficulty"*<br>- *"Cannot get to and use toilet on own without difficulty"*<br>People are classified as having a self-care limitation if they reported ANY of these limitations.
Pain *(any / extreme)*	In the self-completion survey in 1996, 2003-6, 2008, 2010-12 and 2014, respondents were asked *'Now we would like to know how your health is today. Please answer ALL the questions. By ticking one box for each question below, please indicate which statements best describe your own health state today'*:<br>- *"I have no pain or discomfort"*<br>- *"I have moderate pain or discomfort"*<br>- *"I have extreme pain or discomfort"*<br>[This is part of the widely-used EQ-5D health status indicator [8]. However, for the purposes of this paper we have separated the individual measures that make up the EQ5D in order to compare these to similar indicators of morbidity within each domain]. Two outcome measures are based on this: whether people have any pain (the 2nd and 3rd categories combined), and whether they have extreme pain (3rd category only).
Arthritis LSI	Every year 1994-2011, people who report a longstanding illness (LSI) are then asked, *'what is the matter with you?'*; up to 6 responses are then coded by the interviewer into a consistent coding frame based on the International Classification of Diseases.<br>The arthritis LSI measure is based on the group labelled *'Arthritis/rheumatism/fibrositis'*, which as of 2011 includes: Arthritis as result of broken limb; Arthritis/rheumatism in any part of the body; Gout; Osteoarthritis, rheumatoid arthritis, polymyalgia rheumatic; Polyarteritis Nodosa; Psoriasis arthritis; Rheumatic symptoms; and Still's disease.<br>While the LSI coding frame generally stays consistent over this period, interpretation of 'LSI arthritis' is complicated by two changes: Gout and Polyarteritis Nodosa are moved into this code (the documentation is not clear on whether this occurred in 2000 or 2001).
Other musculoskeletal LSI	People who report a longstanding illness (LSI) are then asked, *'what is the matter with you?'*; up to 6 responses are then coded by the interviewer into a consistent coding frame based on the International Classification of Diseases.<br>The other musculoskeletal LSI measure is based on the groups labelled *'Back problems/slipped disc/spine/neck'* and *'Other problems of bones/joints/muscles'*, which as of 2011 includes: Brittle bones, osteoporosis; Bursitis, housemaid's knee, tennis elbow; Cartilage problems; Chondrodystrophia; Chondromalacia; Cramp in hand; Deformity of limbs eg. club foot, claw-hand, malformed jaw; Delayed healing of bones or badly set fractures; Deviated septum; Disc trouble; Dislocations eg. dislocation of hip, clicky hip, dislocated knee/finger; Disseminated lupus; Dupuytren's contraction; Fibromyalgia; Flat feet, bunions; Fracture, damage or injury to extremities, ribs, collarbone, pelvis, skull, eg. knee injury, broken leg, gun shot wounds in leg/shoulder, can't hold arm out flat - broke

	it as a child, broken nose; Frozen shoulder; Hip infection, TB hip; Hip replacement (nes); Legs won't go, difficulty in walking; Lumbago, inflammation of spinal joint; Marfan Syndrome; Osteomyelitis; Paget's disease; Perthe's disease; Physically handicapped (nes); Pierre Robin syndrome; Prolapsed invertebral discs; Schlatter's disease; Schuermann's disease; Sever's disease; Spondylitis, spondylosis; Stiff joints, joint pains, contraction of sinews, muscle wastage; Strained leg muscles, pain in thigh muscles; Systemic sclerosis, myotonia (nes); Tenosynovitis; Torn muscle in leg, torn ligaments, tendonitis; Walk with limp as a result of polio, polio (nes), after affects of polio (nes); Weak legs, leg trouble, pain in legs; and Worn discs in spine - affects legs. The code explicitly <u>excludes</u>: Damage/injury to spine results in paralysis; Sciatica or trapped nerve in spine; and Muscular dystrophy.
Circulatory	
High blood pressure LSI	Every year 1994-2011, people who report a longstanding illness (LSI) are then asked, *'what is the matter with you?'*; up to 6 responses are then coded by the interviewer into a consistent coding frame based on the International Classification of Diseases.<br>The high blood pressure LSI measure is based on the group labelled *'Hypertension/high blood pressure/blood pressure (nes)'*, which as of 2011 includes only the conditions listed in the group label.
Recent high blood pressure	Respondents in 1994, 1998, 2003, 2006 and 2009-2014 were asked a series of questions on whether they have high blood pressure:<br>- *"Do you now have, or have you ever had... high blood pressure (sometimes called hypertension)?"*<br>- Those responding 'yes' were then asked *"Were you told by a doctor or nurse that you had high blood pressure?"*<br>- Women responding 'yes' were then asked, *"Can I just check, were you pregnant when you were told that you had high blood pressure?"*, and those responding 'yes' were then asked *"Have you ever had high blood pressure apart from when you were pregnant?"*<br>- Finally, those with doctor-diagnosed high blood pressure (excluding only when pregnant were asked: *"Are you currently taking any medicines, tablets or pills for high blood pressure?*", and those saying 'no' (or not giving an answer) were then asked, *"Do you still have high blood pressure?"*<br>People were considered to have recent high blood pressure if they said they had ever been diagnosed as having high blood pressure by a doctor (excluding when pregnant), and that they still have high blood pressure or are currently taking medicines for it.<br>While the question wording has stayed consistent, a discontinuity seems to be introduced by a change in question context. In some years (1994, 1998, 2003, 2006 and 2011), this question was preceded by a question that asked, *"May I just check, have you ever had your blood pressure measured by a doctor or nurse?"* (and then for those saying yes, they were asked how recently this was, and whether they were told that it was *'normal (alright/fine), higher than normal, lower than normal, or were you not told anything?'*). However, in other years (2009-10, 2012-14), this question was not asked. Given the way in which context can affect question interpretation, we treat these as two separate measures of recent high blood pressure.
Biomarker high blood pressure	During the nurse visit (which took place for all consenting respondents in all years except 1999, 2002 and 2004, when the nurse visit focussed on particular subsamples), respondents' blood pressure was measured.<br>High blood pressure is defined as a systolic blood pressure >= 140mmHg and diastolic blood pressure >= 90mmHg following HSE established practice, in turn following [10].<br>The measurement of blood pressure changed in 2003, from a Dinamap monitor to an Omron monitor. A conversion is available between the two monitors based on a calibration study, and this has been regularly used by the HSE team to produce

Geiger BB. *BMJ Open* 2021; 10:e032378. doi: 10.1136/bmjopen-2019-032378

continuous trends in blood pressure – see www.hscic.gov.uk/catalogue/PUB00480. For adults, the conversion is as follows:

- For systolic blood pressure: *Predicted Omron=8.90 (SE=2.94) + 0.91 (SE=0.02) * Dinamap.*
- For diastolic blood pressure: *Predicted Omron=19.78 (SE=1.86) + 0.73 (SE=0.03) * Dinamap.*

There are several reasons why respondents who had a nurse visit do not have a valid blood pressure measurement – these are discussed in the Web Appendices 2 and 3.

High cholesterol	In the years 1994, 1998, 2006, and 2008-14, blood samples were obtained during the nurse visit, which were then analysed for total cholesterol. A high level of total cholesterol ('hypercholesterolaemia') is an established risk factor for CVD, and high cholesterol is defined following conventional practice at the NICE guidance 'audit level' of 5mmol/L or above [11 12].<br><br>The measurement of cholesterol changed slightly in 2010 when a new laboratory was used. This resulted in values that are an average of 0.1mmol/L higher, and later values are therefore adjusted by this amount to maintain comparability over time as in [11].
Low HDL cholesterol	In the years 1994, 1998, 2006, and 2008-14, blood samples were obtained during the nurse visit, which were then analysed for high density lipoprotein (HDL) cholesterol. HDL cholesterol *reduces* the risk of CVD (it carries cholesterol away from the arteries towards the liver), and it is therefore low HDL cholesterol that indicates poorer health; low HDL cholesterol is here defined as 1 mmol/L or less [11 12].<br><br>The measurement of HDL cholesterol changed slightly in 2010 when a new laboratory was used. This resulted in values that are an average of 0.1mmol/L lower, and later values are therefore adjusted by this amount to maintain comparability over time as in [11].
Recent heart attack/stroke	Respondents in 1994, 1998, 2003, 2006 and 2011 were asked a series of questions on whether they have had a heart attack (within a battery of questions about different types of heart disease):<br>- *"Have you ever had a heart attack (including myocardial infarction or coronary thrombosis)?"*<br>- Those responding 'yes' were then asked *"Were you told by a doctor that you had a Heart Attack (including myocardial infarction or coronary thrombosis)?"*<br>- Those with doctor-diagnosed angina were asked, *"Have you had a heart attack (including myocardial infarction and coronary thrombosis) during the past 12 months?"*<br>Respondents in these years were similarly asked about stroke:<br>- *"Have you ever had a stroke?"*<br>- Those responding 'yes' were then asked, *"Were you told by a doctor that you had a stroke?"*<br>- Those with doctor-diagnosed stroke were asked, *"Have you had a stroke during the past 12 months?"*<br>People were considered to have recent IHD or stroke if they said they had ever been diagnosed as having stroke or a heart attack by a doctor, and that they have had a heart attack or stroke during the past 12 months.
Recent angina	Respondents in 1994, 1998, 2003, 2006 and 2011 were asked a series of questions on whether they have angina (within a battery of questions about different types of heart disease):<br>- *"Have you ever had angina?"*<br>- Those responding 'yes' were then asked *"You said that you had Angina. Were you told by a doctor that you had Angina?"*<br>- Those with doctor-diagnosed angina were asked, *"Have you had angina during the past 12 months?"*<br>People were considered to have recent angina if they said they had ever been diagnosed as having angina by a doctor, and that they have had it during the past 12 months.

IHD LSI	Every year 1994-2011, people who report a longstanding illness (LSI) are then asked, *'what is the matter with you?'*; up to 6 responses are then coded by the interviewer into a consistent coding frame based on the International Classification of Diseases.<br>The IHD LSI measure is based on the groups labelled *'Stroke/cerebral haemorrhage/cerebral thrombosis'* and *'Heart attack/angina'*. As of 2011 this includes: Cerebro-vascular accident; Coronary thrombosis, myocardial infarction; Heart attack/angina; Hemiplegia, apoplexy, cerebral embolism; Stroke/cerebral haemorrhage/cerebral thrombosis; and Stroke victim - partially paralysed and speech difficulty.
Recent	Respondents in 1994, 1998, 2003, 2006 and 2011 were asked a series of questions on

cardiovascular disease (CVD)	different types of heart disease – including angina; heart attack (including myocardial infarction or coronary thrombosis); a heart murmur; abnormal heart rhythm; or other heart trouble. For EACH of these, they were asked:
	- *"Have you ever had <type of heart disease>?"*<br>- Those responding 'yes' were then asked *"You said that you had <type of heart disease>. Were you told by a doctor that you had <type of heart disease>?"*<br>- For heart murmurs only, women saying they had doctor-diagnosed heart murmurs were asked if they were pregnant when told this, and if so, whether they were ever told they had a heart murmur when they were not pregnant. - Those with doctor-diagnosed heart disease (excluding heart murmurs when pregnant) were asked, *"Have you had <type of heart disease> during the past 12 months?"*<br>People were considered to have recent CVD if they said they had a doctor-diagnosed heart condition and that they had had this during the past 12 months.
Cardiovascular (CVD) LSI	Every year 1994-2011, people who report a longstanding illness (LSI) are then asked, *'what is the matter with you?'*; up to 6 responses are then coded by the interviewer into a consistent coding frame based on the International Classification of Diseases. The CVD LSI measure is based on the groups labelled *'Stroke/cerebral haemorrhage/cerebral thrombosis', 'Heart attack/angina', Hypertension/high blood pressure/blood pressure (nes)', 'Other heart problems', 'Piles/haemorrhoids incl. Varicose Veins in anus', 'Varicose veins/phlebitis in lower extremities', and 'Other blood vessels/embolic'*. As of 2011 this includes: Aorta replacement; Aortic valve stenosis; Aortic/mitral valve regurgitation; Arterial thrombosis; Arteriosclerosis, hardening of arteries (nes); Artificial arteries (nes); Atrial Septal Defect (ASD); Blocked arteries in leg; Blood clots (nes); Cardiac asthma; Cardiac diffusion; Cardiac problems, heart trouble (nes); Cerebrovascular accident; Coronary thrombosis, myocardial infarction; Dizziness, giddiness, balance problems (nes); Hand Arm Vibration Syndrome (White Finger); Hardening of arteries in heart; Heart attack/angina; Heart disease, heart complaint; Heart failure; Heart murmur, palpitations; Hemiplegia, apoplexy, cerebral embolism; Hole in the heart; Hypersensitive to the cold; Hypertension/high blood pressure/blood pressure (nes);<br>Intermittent claudication; Ischaemic heart disease; Low blood pressure/hypertension; Mitral valve stenosis; Pacemaker; Pains in chest (nes); Pericarditis; Piles/haemorrhoids incl. Varicose Veins in anus; Poor circulation; Pulmonary embolism; Raynaud's disease; St Vitus dance; Stroke victim - partially paralysed and speech difficulty; Stroke/cerebral haemorrhage/cerebral thrombosis; Swollen legs and feet; Tachycardia, sick sinus syndrome; Telangiectasia (nes); Thrombosis (nes); Tired heart; Valvular heart disease; Valvular heart disease; Varicose veins in Oesophagus; Varicose veins/phlebitis in lower extremities; Various ulcers, varicose eczema; Weak heart because of rheumatic fever; Wolff - Parkinson - White syndrome; and Wright's syndrome. It explicitly excludes balance problems due to ear complaint & haemorrhage behind eye.<br>While the LSI coding frame generally stays consistent over this period, interpretation of 'IHD LSI' is complicated by two changes: 'Too much cholesterol in blood' is included in this category in 1994 only, and Polyarteritis Nodosa is later moved into this code (the documentation is not clear on whether this occurred in 2000 or 2001).
Angina symptoms	This is taken from the Rose Angina questionnaire [13 14]. Respondents in 1994, 1998, 2003, 2006 and 2011 were asked a series of questions about *symptoms* of heart trouble (rather than whether they had been diagnosed):<br>- *"I am now going to ask you some questions mainly about symptoms of the chest. Have you ever had any pain or discomfort in your chest?"* - Those that said 'yes' were asked:<br>o *Do you get it when you walk uphill or hurry? Yes | No | Sometimes/ Occasionally | Never walks uphill or hurries | (Cannot walk)"*. If sometimes/occasionally, they were asked: *"Does this happen on most occasions?"*<br>o If not 'no' to having pain/discomfort in their chest, they were asked: *"Do you get it when you walk at an ordinary pace on the level? Yes | No |*

	Sometimes/Occasionally | Never walks at an ordinary pace on the level". If sometimes/occasionally, they were asked: *"Does this happen on most occasions?"*<br>- Those who every had pain/discomfort when walking uphill/hurrying or walking at ordinary pace on the level were asked:<br>o *"What do you do if you get it while you are walking? Do you stop, slow down or carry on?"* (If respondents were unsure, they were asked, *"What do you do on most occasions?")*<br>o Those who said they stop or slow down were asked, *"If you stand still does the pain go away or not?"* (If respondents were unsure, they were asked, *"What happens to the pain on most occasions?").* If the pain goes away, they were asked, *"How soon does the pain go away? Does it go in 10 minutes or less, or more than 10 minutes?"*<br>o Those who said the pain goes away in 10 minutes or less were asked, *"Will you show me where you get this pain or discomfort? Where else"* The interviewer then coded the site as Sternum (upper or middle) | Sternum lower | Left anterior chest | Left arm | Right anterior chest | Right arm | (Somewhere else).<br>Following the HSE reports, possible angina is defined as chest pain or discomfort that (i) includes either the sternum or the left arm and left anterior chest; (ii) is prompted by hurrying or walking uphill (or by walking on the level, for those who never attempt more); (iii) makes the respondent either stop or slacken pace; and (iv) usually disappears in 10 minutes or less when they stand still.
Heart attack symptoms	This is taken from the Rose Angina questionnaire. Respondents in 1994, 1998, 2003, 2006 and 2011 were asked, *"Have you ever had a severe pain across the front of your chest lasting for half an hour or more?"* As in the 2006 HSE report, those responding 'yes' are treated as having a possible heart attack (myocardial infarction).
Mini stroke (TIA) symptoms	Respondents in 2003, 2006 and 2011 were asked:<br>o *"In the last twelve months, have you had a sudden attack of weakness or numbness on one side of the body?"*<br>o *"Have you had a sudden attack of slurred speech or difficulty in finding words in the last twelve months?"*<br>o *"Have you had a sudden attack of vision loss or blurred vision in one or both eyes in the last twelve months?"*<br>People reporting ANY of these symptoms were considered as possibly having had a transient ischaemic attack (TIA), often called a 'mini stroke'.
Respiratory	
COPD symptoms	Respondents in 1995, 1996 and 2010 were asked:<br>o *"Do you usually cough first thing in the morning in the winter?"* (In 2010 only, respondents had previously been asked *"Do you usually cough first thing in the morning?"* – but this is not used to filter people into the questions on coughing in winter).<br>o *"Do you usually bring up any phlegm from your chest, first thing in the morning in the winter?"* (Again, this was asked to everyone in all years, but was preceded by an additional, non-winter-specific question in 2010).<br>o Those saying 'yes' to each question were then asked, "*Do you [cough/bring up phlegm] like this on most days for as much as three months each year?"* In 2010 only, this was followed by the additional clarification '*That is, for three consecutive months'.*<br>People who reported three months/year of BOTH coughing first thing and of phlegm are considered to have possible symptoms of Chronic Obstructive Pulmonary Disease (COPD).

Diagnosed asthma	In 1995-7, 2001 and 2010, respondents were asked *"Did a doctor <1997 and 2010 only: or nurse> ever tell you that you had asthma?"* Whereas for other doctor-diagnosed conditions (heart problems/diabetes) we focus on those reporting problems in the past 12 months, it is not possible to construct a consistent measure of recent asthma, hence this variable refers to *lifetime* doctor-diagnosed asthma.
Asthma LSI	Every year 1994-2011, people who report a longstanding illness (LSI) are then asked, *'what is the matter with you?'*; up to 6 responses are then coded by the interviewer into a consistent coding frame based on the International Classification of Diseases.<br>The asthma LSI measure is based on the group labelled *'Asthma'*, which as of 2011 includes: Asthma; Bronchial asthma, allergic asthma; and Asthma - allergy to house dust/grass/cat fur. It explicitly <u>excludes</u> cardiac asthma.
Shortness of breath *(Grade 2+ / Grade 3)*	Respondents in 1995, 1996 and 2010 were asked the following questions about shortness of breath ('dyspnoea'):<br>○ *"Are you troubled by shortness of breath when hurrying on level ground or walking up a slight hill? Yes | No | Never walks up hill or hurries | Cannot walk"*<br>○ Those responding 'yes' or 'never walks up hill or hurries' are then asked, *"Do you get short of breath walking with other people of (your/his/her) own age on level ground? Yes | No | Never walks with people of own age on level ground".*<br>○ Those responding 'yes' or 'never walks with people of own age' are then asked, *"Do you have to stop for breath after walking at (your/his/her) own pace on level ground?"*<br>This has been combined into the longstanding MRC dyspnoea scale [15] as follows:<br>- Grade 2 dyspnoea: people who report shortness of breath when hurrying on level ground or walking up a slight hill (or who report shortness of breath when walking on level ground, but who say they never walk up hill or hurry).<br>- Grade 3 dyspnoea: people who report shortness of breath when walking with people of own age on level ground, or who have to stop for breath when walking at own pace on level ground.<br>(The same questions also exist in 1994 and 1998, but (i) the wider bank of questions differs substantially in the two versions and question context effects are likely; and (ii) the filtering into the final question differs between versions. However, the 1991-98 trends are included below).
Recent wheezing/ asthma symptoms	Respondents in 1995-97, 2001 and 2010 were asked the following two questions as part of the battery of questions on breathing problems:<br>- *"I am now going to ask you some questions about your breathing... Have you ever had wheezing or whistling in the chest at any time, either now, or in the past?"*<br>- Those that said yes were then asked, *"Have you had wheezing or whistling in the chest in the last 12 months?"*<br>- (For those who said they had ever been told by a doctor they had asthma; see above), "*When was your most recent attack of asthma? PROMPT IF NECESSARY: Less than 4 weeks ago | More than 4 weeks but within the last 12 months | One to five years ago | More than 5 years ago"*<br>People who said they had EITHER wheezing/whistling in the past 12 months or an asthma attack in the past 12 months were counted as having recent wheezing/asthma symptoms.<br>[It should be noted that the filtering to the second question is very slightly different in 2010 compared to previous years (it was only asked to people who said they had not had wheezing/whistling in the chest in the past 12 months). However, given the way that the derived variable is calculated here, the change in filtering does not introduce any discontinuities over time].

Wheezing stopping sleep	Respondents in 1995-97, 2001 and 2010 were asked the following two questions as part of the battery of questions on breathing problems:<br>- *"I am now going to ask you some questions about your breathing... Have you ever had wheezing or whistling in the chest at any time, either now, or in the past?"*<br>- Those that said yes were then asked, *"Have you had wheezing or whistling in the chest in the last 12 months?"*<br>- Those that said yes were then asked, *"In the last 12 months, how often on average has your sleep been disturbed due to wheezing or whistling in the chest?: Have you: Never woken with wheezing | Woken less than one night per week, or | Woken one or more nights per week?"*<br>People were considered to have wheezing during sleep if they reported this at least once per week.
Anthropometric & diabetes	
BMI *(Underweight / Obese)*	During the initial face-to-face interview in all years (except 2013), respondents were asked if they would consent to having their height and weight measured by the interviewer. The reasons for missingness (and their trends over time) are given in Web Appendices 2 & 3; note that there are three changes that give rise to small discontinuities in 2009 and 2011.<br>Obesity is a risk factor for diabetes (hence its inclusion in this section) but also heart disease and some cancers. Obesity is defined as a Body Mass Index (BMI) of >= 30kg/m^2 as per the World Health Organization's BMI classification [16]. Using the same definition, underweight is defined as <=18.5 kg/m^2.
High waist-hip ratio	During the nurse visit in most years (excluding 1995-96, 2002, 2004 and 2013), respondents had their waist and hip circumferences measured. While BMI is a standard measurement of obesity, some evidence suggests that fat around the waist – 'central adiposity' – is a greater risk to health than fat elsewhere [17]. We use NICE's suggested 2006 thresholds for a high waist-hip ratio of >1 for men and >0.85 for women [18], as used in Hotchkiss et al [19].
Recent diabetes	Respondents in 1994, 1998, 2003, 2006 and 2009-2014 were asked a series of questions on whether they have diabetes:<br>- *"Do you now have, or have you ever had diabetes?"*<br>- Those responding 'yes' were then asked *"Were you told by a doctor that you had diabetes?"*<br>- Women responding 'yes' were then asked, *"Can I just check, were you pregnant when you were told that you had diabetes?"*, and those responding 'yes' were then asked *"Have you ever had diabetes apart from when you were pregnant?"*<br>- Finally, those with doctor-diagnosed diabetes (excluding only when pregnant were asked: *"Do you currently inject insulin for diabetes?"* and *"Are you currently taking any medicines, tablets or pills (other than insulin injections) for diabetes?"*<br>People were considered to have recent diabetes if they said they had ever been diagnosed as having diabetes by a doctor (excluding when pregnant), and that they are injecting insulin or taking any other medicines for diabetes.
Diabetes LSI	Every year 1994-2011, people who report a longstanding illness (LSI) are then asked, *'what is the matter with you?'*; up to 6 responses are then coded by the interviewer into a consistent coding frame based on the International Classification of Diseases.<br>The diabetes LSI measure is based on the group labelled *'Diabetes'*, which as of 2011 includes Diabetes and Hyperglycaemia.

High glycated haemoglobin	In the years 2003, 2006, and 2008-14, blood samples were obtained during the nurse visit, which were then analysed for glycated haemoglobin (HbA_{1C}). HbA_{1C} is a measure of the share of haemoglobin (within red blood cells) that glucose is attached to, with higher levels indicated less well-controlled diabetes in the previous three months [20]. Following the recommendations of a 2009 expert committee, we mirror recent HSE reports in using a threshold of 48mmol/mol (i.e. 48 millimoles of glycated haemoglobin per mole of haemoglobin) as the threshold for raised HbA_{1C}, a different threshold to that used in earlier HSE reports.<br><br>While the measurement of HbA_{1C} has been consistent in HSE from 1994, the units reported have changed from the % of haemoglobin that is glycated to mmol/mol. Earlier measures have been transformed into mmol/mol through the formula, mmol/mol = (% - 2.15) x 10.929. HbA_{1C} was also measured in 1994 but using a different technique, which cannot be made comparable [21:67].
Other biomarkers	
Raised C-reactive protein	In the years 1998, 2003, 2006, and 2009, blood samples were obtained during the nurse visit, which were then analysed for C-reactive protein (CRP). CRP is an inflammatory marker, which can indicate heart-related inflammation (it is used to test for heart failure) but can also indicate other sorts of health damage including diabetes. However, there are still debates about exactly what CRP shows, both in terms of its causal role in heart disease, and whether it also indicates depression.[22]<br><br>Raised CRP is defined as >3mg/L, the standard cut-off for a clinically significant rise in CVD [23 24]. Participants with CRP >10mg/L are excluded, as this is taken to be evidence of current infection rather than inflammation from chronic disease.
Raised Fibrinogen	In the years 1998, 2003, 2006, and 2009, blood samples were obtained during the nurse visit, which were then analysed for fibrinogen. Like CRP, fibrinogen is an inflammatory marker, which is both commonly thought to be a causal risk factor for CVD (it is a component of coagulation), and which seems to be a risk factor for other diseases (including cancer and diabetes)[25].<br><br>While fibrinogen is often analysed as a continuous variable with no cutpoints [24], we here define raised fibrinogen as>4mg/L as in [12]. As for CRP, participants with CRP >10mg/L are excluded, as this is taken to be evidence of current infection rather than inflammation from chronic disease. A change of analysis method and laboratory between 1994 and 1998 means that the 1994 results are not comparable to the later results [26:8.10.4].
Anaemia	In the years 1994, 1998, 2006, and 2009, blood samples were obtained during the nurse visit, which were then analysed for haemoglobin. Haemoglobin dist ributes oxygen around the body, and low haemoglobin levels usually indicate anaemia. Various different thresholds for low haemoglobin have been used in the literature, particularly for older populations [27], but we here used the longstanding WHO definition of <13g/dL for men and <12g/dL for women [24].
Iron deficiency	In the years 1994, 1998, 2006, and 2009, blood samples were obtained during the nurse visit, which were then analysed for serum ferritin (which correlates directly with the amount of iron stored in the body). Iron deficiency is one of several possible causes of anaemia (alongside other nutritional deficiencies, genetic conditions such as sickle cell anaemia, infections, and blood loss). Iron deficiency is defined as a serum ferritin less than 45ng/ml [27].
Mental health	
Mental health LSI	Every year 1994-2011, people who report a longstanding illness (LSI) are then asked, *'what is the matter with you?'*; up to 6 responses are then coded by the interviewer into a consistent coding frame based on the International Classification of Diseases. The mental health LSI measure is based on the group labelled *'Mental illness/anxiety/depression/nerves (nes)'*, which as of 2011 includes: Alcoholism, recovered not cured alcoholic; Angelman Syndrome; Anorexia nervosa; Anxiety, panic attacks; Asperger

	Syndrome; Autism/Autistic (BBG: changed from 'autistic child'); Bipolar Affective Disorder; Catalepsy; Concussion syndrome; Depression; Drug addict; Dyslexia; Hyperactive child.; Nerves (nes); Nervous breakdown, neurasthenia, nervous trouble; Phobias; Schizophrenia, manic depressive; Senile dementia, forgetfulness, gets confused; Speech impediment, stammer; and Stress. It explicitly <u>excludes</u> Alzheimer's disease, degenerative brain disease.<br>While the LSI coding frame generally stays consistent over this period, it is worth being aware of a minor wording change within 'mental health LSI': the condition labelled 'Autistic child' 1994-1997 was relabelled 'Autism/Autistic' in 1998.
Psychological distress (GHQ)	In the self-completion survey in most years (except 1996, 2007, 2011 and 2013), respondents were asked the following series of questions:<br>- *"Please read this carefully: We should like to know how your health has been in general over the past few weeks. Please answer ALL the questions by ticking the box below the answer which you think most applies to you. Have you recently...*<br>- *"…been able to concentrate on whatever you're doing?" RESPONSES: "Better than usual" | "Same as usual" | "Less than usual" | "Much less than usual"*<br>- *"…lost much sleep over worry?" RESPONSES: "Not at all" | "No more than usual" | "Rather more than usual" | "Much more than usual""*

- *"…felt you were playing a useful part in things?" RESPONSES: "More so than usual" | "Same as usual" | "Less useful than usual" | "Much less useful""*
- *"…felt capable of making decisions about things?" RESPONSES: "More so than usual" | "Same as usual" | "Less so than usual" | "Much less capable""*
- *"…felt constantly under strain? RESPONSES: "Not at all" | "No more than usual" | "Rather more than usual" | "Much more than usual""*
- *"..felt you couldn't overcome your difficulties?" RESPONSES: "Not at all" | "No more than usual" | "Rather more than usual" | "Much more than usual""*
- *"…been able to enjoy your normal day-to-day activities?" RESPONSES: "More so than usual" | "Same as usual" | "Less so than usual" | "Much less than usual"*
- *"…been able to face up to your problems?" RESPONSES: "More so than usual" | "Same as usual" | "Less able than usual" | "Much less able"*
- *"…been feeling unhappy and depressed? RESPONSES: "Not at all" | "No more than usual" | "Rather more than usual" | "Much more than usual"*
- *"…been losing confidence in yourself? RESPONSES: "Not at all" | "No more than usual" | "Rather more than usual" | "Much more than usual"*
- *"…been thinking of yourself as a worthless person?" RESPONSES: "Not at all" | "No more than usual" | "Rather more than usual" | "Much more than usual""*
- *"…been feeling reasonably happy, all things considered?" RESPONSES: "More so than usual" | "Same as usual" | "Less so than usual" | "Much less happy"*

These make up the 12-item General Health Questionnaire GHQ-12; [28], a well-validated, widely-used measure of probable mental ill-health. This is often termed general nonpsychotic psychiatric morbidity, but I here use the more easily understood term 'psychological distress' following Stochl et al 2016.[29]

A total score has been created by first ensuring that all questions were coded from 1 (positive symptom) to 4 (negative symptom), and then creating a sum score for all the number of questions in which people answered with categories 3 or 4 (indicating a negative symptom). A binary measure (often called GHQ caseness) was created for people who had negative symptoms for 4 or more of the 12 questions.

Anxiety/depression *(moderately / Extremely)*	In the self-completion survey in 1996, 2003-6, 2008, 2010-12 and 2014, respondents were asked *'Now we would like to know how your health is today. Please answer ALL the questions. By ticking one box for each question below, please indicate which statements best describe your own health state today':*<br>- *"I am not anxious or depressed"*<br>- *"I am moderately anxious or depressed"*<br>- *"I am extremely anxious or depressed"*

Web Appendix page 20

	[This is part of the widely-used EQ-5D health status indicator [8]. However, for the purposes of this paper we have separated the individual measures that make up the EQ5D in order to compare these to similar indicators of morbidity within each domain]. Two outcome measures are based on this: whether people have any anxiety/depression (the 2nd and 3rd categories combined), and whether they have extreme anxiety/depression (3rd category only).
Communication	
Hearing, seeing & communication limitations	These measures were not included in the main paper due to the short time frame that we can examine trends over, but are included in the Web Appendix as they relate to important domains of morbidity.<br>They were included in the disability scale used in the 2001 HSE report [9]. Respondents in 1995, 2000 and 2001 were asked if of the following applied to them (interviewers were instructed to ignore temporary disabilities that are expected to last less than one year):<br>• *"Cannot follow a TV programme at a volume others find acceptable (with hearing aid if normally worn)"* ('hearing limitation')<br>• *"Cannot see well enough to recognise a friend across a road (four yards away) (with glasses or contact lenses if normally worn)"* ('seeing limitation')<br>• *"Have problem communicating with other people - that is, have problem understanding them or being understood by them"* ('communication limitation')
Eye/Ear LSI	Every year 1994-2011, people who report a longstanding illness (LSI) are then asked, *'what is the matter with you?'*; up to 6 responses are then coded by the interviewer into a consistent coding frame based on the International Classification of Diseases. The Eye/Ear LSI includes the following groups:<br>• *Poor hearing/deafness*, including Conductive/nerve/noise induced deafness, Deaf mute/deaf and dumb, Heard of hearing, slightly deaf, Otosclerosis, Poor hearing after mastoid operation.<br>• *Tinnitus/noises in the ear*, Incl. pulsing in the ear<br>• *Other ear complaints*, Incl. otitis media - glue ear, Disorders of Eustachian tube, Perforated ear drum (nes), Middle/inner ear problems, Mastoiditis, Ear trouble (nes),, Ear problem (wax), Ear aches and discharges, Ear infection<br>• *Cataract/poor eye sight/blindness*, Incl. operation for cataracts, now need glasses, Bad eyesight, restricted vision, partially sighted, Bad eyesight/nearly blind because of cataracts, Blind in one eye, loss of one eye, Blindness caused by diabetes, Blurred vision, Detached/scarred retina, Hardening of lens, Lens implants in both eyes, Short sighted, long sighted, myopia, Trouble with eyes (nes), eyes not good (nes), Tunnel vision<br>• *Other eye complaints*, including Astigmatism, Buphthalmos, Colour blind, Double vision, Dry eye syndrome, trouble with tear ducts, watery eyes, Eye infection, conjunctivitis, Eyes are light sensitive, Floater in eye, Glaucoma, Haemorrhage behind eye, Injury to eye, Iritis, Keratoconus, Night blindness, Retinitis pigmentosa, Scarred cornea, corneal ulcers, Squint, lazy eye, Sty on eye.

Changes over time in several other measures are only presented in Web Appendices 4 & 6, rather than the main paper. Details of these variables are included below:

**Measure**	**Details**
General health	
General health *(bad / good)*	Every year, respondents were asked, *"How is your health in general? Would you say it was ... very good, good, fair, bad, or very bad?"*

Web Appendix page 21

	Two outcome measures are based on this, following standard practice in the HSE reports: bad general health (which includes 'bad' or 'very bad' health) and good general health (which includes 'good' or 'very good' health).
Longstanding illness (LSI)	Every year 1994-2011, respondents were asked *"Do you have any long-standing illness, disability or infirmity? By long-standing I mean anything that has troubled you over a period of time, or that is likely to affect you over a period of time?"* (The response options were 'Yes' and 'No').<br>In 2012 the question was changed to be consistent with the Government's new harmonised disability questions for use in social surveys [30], and is not comparable to the previous version.
Limiting LSI	Every year 1996-2011, respondents who said they had an LSI were than asked, *"Does this illness or disability (do any of these illnesses or disabilities) limit your activities in any way?"* (again allowing only Yes/No answers).<br>In 2012 the question was changed to be consistent with the Government's new harmonised disability questions for use in social surveys (see HSE 2012 report), and is not comparable to the previous version.
Problems with usual activities<br>*(some problems / unable)*	In the self-completion survey in 1996, 2003-6, 2008, 2010-12 and 2014, respondents were asked *'Now we would like to know how your health is today. Please answer ALL the questions. By ticking one box for each question below, please indicate which statements best describe your own health state today'*:<br>- *"I have no problems with performing my usual activities (e.g. work, study, housework, family or leisure activities)"*<br>- *"I have some problems with performing my usual activities"*<br>- *"I am unable to perform my usual activities"*<br>[This is part of the widely-used EQ-5D health status indicator [8]. However, for the purposes of this paper we have separated the individual measures that make up the EQ5D in order to compare these to similar indicators of morbidity within each domain].<br>Two outcome measures are based on this: whether people have any problems (the 2nd and 3rd categories combined), and whether they are unable to perform their usual activities (3rd category only).
Limitations in past 2wks	Every year, respondents were asked, *"Now I'd like you to think about the two weeks ending yesterday. During those 2 weeks did you have to cut down on any of the things you usually do (about the house or at work or in your free time) because of your answer at <the LSI question> or some other illness or injury?"*<br>There have been two small changes to this question's wording in 1996. Firstly, 'work' was changed to 'work/school'. Secondly, '*your answer at <the LSI question>*' was changed to '*a condition you have just told me about*'. While it is impossible to be sure of the exact effect of these changes, neither seem likely to influence the results (at least for the 25+ age group where fewer individuals are in full-time education).

Appendix 6: Measures not included in the main paper

Trends in several measures are not included in the main paper, either

Table 1: Changes over time in measures not included in the main paper

	Starting period		Change from start to end period			
	Period	Prevalence	End period	Raw change	Adj.[a] change	Adj. change 95% CI
CVD						
Component measures nec[b]						
Recent heart murmur	1994-96	0.8%	2011-14	0.1%	0.0%	[-0.3, 0.4%]
Recent irregular heart rhythm	1994-96	1.6%	2011-14	0.4%	0.4%	[-0.1, 0.8%]
Recent other heart disease	1994-96	0.2%	2011-14	0.7%	0.7%	[0.4, 0.9%]
Ever had (not just recent)						
Ever had high BP DD	1994-96	19.0%	2011-14	4.5%	3.7%	[2.3, 5.1%]
high BP	1994-96	13.2%	2011-14	6.9%	6.0%	[4.7, 7.3%]
Ever IHD or stroke	1994-96	2.9%	2011-14	0.3%	-0.0%	[-0.6, 0.6%]
DD IHD or stroke	1994-96	2.5%	2011-14	0.5%	0.2%	[-0.3, 0.7%]
Ever had angina	1994-96	1.9%	2011-14	-0.2%	-0.4%	[-0.9, 0.0%]
Ever DD angina	1994-96	1.6%	2011-14	-0.1%	-0.3%	[-0.7, 0.1%]
Ever heart murmur	1994-96	3.2%	2011-14	-0.3%	-0.3%	[-0.9, 0.3%]
DD heart murmur	1994-96	2.6%	2011-14	-0.2%	-0.2%	[-0.7, 0.3%]
Ever irregular heart rhythm	1994-96	6.4%	2011-14	-0.7%	-0.9%	[-1.7, -0.1%]
DD irregular heart rhythm	1994-96	3.5%	2011-14	0.5%	0.3%	[-0.3, 1.0%]
Ever other heart disease	1994-96	0.9%	2011-14	1.1%	1.0%	[0.6, 1.5%]
DD other heart disease	1994-96	0.8%	2011-14	1.0%	1.0%	[0.6, 1.4%]
Respiratory						
**Alternate measures**						
Phlegm symptoms	1994-96	9.1%	2008-10	-1.3%	-1.4%	[-2.3, -0.5%]
LSI Respiratory All	1994-96	7.9%	2011-14	-0.7%	-0.7%	[-1.6, 0.1%]
**Ever had (not just recent)**						
Wheezing Ever	1994-96	32.3%	2008-10	0.0%	-0.1%	[-1.8, 1.5%]
Wheezing Past 12mths	1994-96	18.9%	2008-10	-1.0%	-1.1%	[-2.3, 0.2%]
Diabetes						
**Ever had (not just recent)**						
Ever diabetes	1994-96	2.0%	2011-14	2.9%	2.8%	[2.3, 3.2%]
DD diabetes	1994-96	1.7%	2011-14	2.5%	2.3%	[2.0, 2.7%]
Mental health						
**Alternate measures**						
High psychological distress	1994-96	3.2%	2011-14	1.0%	0.9%	[0.4, 1.4%]
Activity limitations & musculoskeletal						
**For comparison**						
Walking limitation	1994-96	4.6%	2001-03	1.4%	1.2%	[0.5, 1.9%]
Washing/dressing limitation	1994-96	1.9%	2001-03	0.5%	0.4%	[0.0, 0.8%]

Other LSIs	Starting period		Change from start to end period			
	Period	Prevalence	End period	Raw change	Adj.[a] change	Adj. change 95% CI
LSI Blood Disorders	1994-96	0.3%	2011-14	0.6%	0.5%	[0.3, 0.8%]
LSI Cancer	1994-96	1.0%	2011-14	0.3%	0.3%	[-0.1, 0.6%]
LSI D,GUM,E&M	1994-96	6.9%	2011-14	1.1%	0.8%	[0.0, 1.6%]
LSI Epilepsy	1994-96	0.7%	2011-14	0.1%	0.1%	[-0.2, 0.3%]
LSI Nervous System	1994-96	3.7%	2011-14	-0.2%	-0.3%	[-0.8, 0.3%]

[a] 'Adj.' = trend adjusted for changing age and sex distribution of the working-age population. [b] 'nec' = not elsewhere included.

The details of these measures are as follows:

Measure	Details
**Circulatory**	
Beyond 'recent': 'Ever had' and 'DD' CVD	In the main paper, we look at whether people report recent doctor-diagnosed CVD (looking separately at heart attack/stroke, angina, and any recent CVD). As shown above, this comes from three questions: whether people report ever having this condition; whether a doctor diagnosed this; and whether they have had an attack in the past 12 months / consider themselves to still have the condition. Web Appendix 6 shows trends in the other versions of these measures, i.e. having ever had this type of CVD, and having ever doctor-diagnosed ('DD') CVD of this type.
Component measure: Heart murmur Irregular heart rhythm Other heart disease	In the main paper, we recent reports of doctor-diagnosed angina; heart attack (including myocardial infarction or coronary thrombosis); a heart murmur; abnormal heart rhythm; or other heart trouble (see above). Angina and heart attack are also analysed in the main paper in their own right; in Web Appendix 6, we further show trends separately in heart murmur, abnormal heart rhythm or other heart trouble.
**Respiratory**	
Component measure: 'phlegm'	In the main paper, we look at whether people report recent COPD (see above). This combines two measures: regular cough + phlegm. Web Appendix 6 shows the trend in the phlegm measure on its own, without being combined with a regular cough.
Alternative version: respiratory' longstanding illness ('LSI') which is included within the broader category of respiratory conditions.	In the main paper, we look at whether an asthma LSI (to examine alongside a direct 'LSI question on diagnosed asthma); see above. Web Appendix 6 also shows people reporting a The respiratory LSI measure is based on the group labelled *'Asthma'*, *'Bronchitis'*, *'Hayfever'*, or *'Respiratory other'*, which as of 2011 includes: Asthma: Asthma; Bronchial asthma, allergic asthma; and Asthma - allergy to house dust/grass/cat fur. It explicitly <u>excludes</u> cardiac asthma. Hayfever: Hayfever, Allergic rhinitis Bronchitis/emphysema: Bronchitis/emphysema, Bronchiectasis, Chronic bronchitis. Other respiratory complaints: Other respiratory complaints, Abscess on larynx, Adenoid problems, nasal polyps, Allergy to dust/cat fur, Bad chest (nes), weak chest – wheezy, Breathlessness, Bronchial trouble, chest trouble (nes), Catarrh, Chest infections, get a lot of colds, Churg-Strauss syndrome, Chronic Obstructive Pulmonary Disease (COPD), Coughing fits, Croup, Damaged lung (nes), lost lower lobe of left lung, Fibrosis of lung, Furred up airways,

collapsed lung, Lung complaint (nes), lung problems (nes), Lung damage by viral pneumonia, Paralysis of vocal cords, Pigeon fancier's lung, Pneumoconiosis, byssinosis, asbestosis and other industrial respiratory disease, Recurrent pleurisy, Rhinitis (nes), Sinus trouble, sinusitis, Sore throat, pharyngitis, Throat

Geiger BB. *BMJ Open* 2021; 10:e032378. doi: 10.1136/bmjopen-2019-032378

Measure	Details
	infection, Throat trouble (nes), throat irritation, Tonsillitis, Ulcer on lung, fluid on lung. Note that:<br>• It explicitly excludes TB (pulmonary tuberculosis), Cystic fibrosis, Skin allergy, Food allergy, Allergy (nes), Pilonidal sinus, Sick sinus syndrome, Whooping cough.
For comparison: Washing & dressing limitation	This is based on the personal care disability scale used in the 2001 HSE report [9]. Respondents in 1995, 2000 and 2001 were asked if any of the following applied to them (interviewers were instructed to ignore temporary disabilities that are expected to last less than one year):<br>- *"Cannot dress and undress without difficulty"*<br>- *"Cannot wash hands and face without difficulty"*<br>For comparison to the 'problems with washing/dressing today' measure in the main paper (which covers a more extended period and is based on a different question; see above), a measure is derived if respondents say they report either of these problems.
Other LSIs	

Geiger BB. *BMJ Open* 2021; 10:e032378. doi: 10.1136/bmjopen-2019-032378

**Measure**	**Details**
Other LSIs	Every year 1994-2011, people who report a longstanding illness (LSI) are then asked, *'what is the matter with you?'*; up to 6 responses are then coded by the interviewer into a consistent coding frame based on the International Classification of Diseases. The various other LSIs are as follows:<br>• The Blood Disorders LSI measure is based on the group *'Disorders of blood and blood forming organs and immunity disorders'*, which as of 2011 includes: Anaemia, pernicious anaemia, Blood condition (nes), blood deficiency, Haemophilia, Idiopathic Thrombochopenic Purpura (ITP), Immunodeficiences, Polycthaemia (blood thickening), blood to thick, Purpura (nes), Removal of spleen, Sarcoidosis (previously code 37), Sickle cell anaemia/disease, Thalassaemia, Thrombocythenia. It explicitly excludes Leukaemia - code 01.<br>• The Cancer LSI measure is based on the group *'Cancer (neoplasm) including lumps, masses, tumours and growths and benign (non-malignant) lumps and cysts'*, which as of
	• If complaint is breathlessness with the cause also stated, this is coded with the cause – hence it also excludes breathlessness as a result of anaemia, breathlessness due to hole in heart, and breathlessness due to angina.
Component measure: Wheezing	In the main paper, we look at whether people report recent wheezing/asthma. As shown above, this comes from three questions: whether people report ever having had wheezing or whistling in the chest; whether they have had this in the past 12 months; and whether they have had an asthma attack in the past 12 months.<br>Web Appendix 6 shows trends in the other versions of these measures, i.e. having ever had wheezing/whistling in the chest, and whether they have had this in the past 1 months.
Beyond 'recent': 'Ever had' and 'DD' having diabetes	In the main paper, we look at whether people report recent doctor-diagnosed diabetes As shown above, this comes from three questions: whether people report ever this condition; whether a doctor diagnosed this; and whether they currently inject insulin / take other medication for diabetes.<br>Web Appendix 6 shows trends in the other versions of these measures, i.e. having ever had diabetes, and having ever doctor-diagnosed ('DD') diabetes.
Activity limitations	
For comparison: Walking limitation	This is based on the personal care disability scale used in the 2001 HSE report [9]. Respondents in 1995, 2000 and 2001 were asked if of the following applied to them (interviewers were instructed to ignore temporary disabilities that are expected to last less than one year): *"Cannot walk 200 yards or more on own without stopping or discomfort"*. People who reported a limitation were asked if they used a walking aid, and if they did, were then asked if they could walk 200 yards without the walking aid.
	2011 includes: Acoustic neuroma, After effect of cancer (nes), All tumours, growths, masses, lumps and cysts, whether malignant or benign eg. tumour on brain,, growth in bowel, growth on spinal cord, lump in, breast, Cancers sited in any part of the body or system eg., Lung, breast, stomach, Colostomy caused by cancer, Cyst on eye, cyst in kidney., General arthroma, Hereditary cancer, Hodgkin's disease, Hysterectomy for cancer of womb, Inch. leukaemia (cancer of the blood), Lymphoma, Mastectomy (nes), Neurofibromatosis, Part of intestines removed (cancer), Pituitary gland removed (cancer), Rodent ulcers, Sarcomas, carcinomas, Skin cancer, bone cancer, Wilms tumour

Measure	Details
	• The D,GUM,E&M (Digestive, Genitourinary Medicine, and Endocrine & Metabolic) LSI is based on the groups, *'Complaints of bowel/colon (large intestine,caecum, bowel, colon, rectum)'* (including Colitis, colon trouble, ulcerative colitis, Coleliac, Colostomy (nes), Crohn's disease, Diverticulitis, Enteritis, Faecal incontinence/encopresis., Frequent diarrhoea, constipation, Grumbling appendix, Hirschsprung's disease, Irritable bowel, inflammation of bowel, Polyp on bowel, Spastic colon, but explicitly excluding piles and Cancer of stomach/bowel), *Other digestive complaints (stomach, liver, pancreas, bile ducts, small intestine - duodenum, jejunum and ileum)* (including Cirrhosis of the liver, liver problems, Food allergies, Ileostomy, Indigestion, heart burn, dyspepsia, Inflamed duodenum, Liver disease, biliary artesia, Nervous stomach, acid stomach, Pancreas problems, Stomach trouble (nes), abdominal trouble (nes), Stone in gallbladder, gallbladder problems, Throat trouble - difficulty in swallowing, Weakness in intestines), *Stomach ulcer/ulcer (nes)/abdominal hernia/rupture* (including Double/inguinal/diaphragm/hiatus/umbilical hernia, Gastric/duodenal/peptic ulcer, Hernia (nes), rupture (nes), Ulcer (nes)), *Complaints of teeth/mouth/tongue* (including Cleft palate, hare lip, Impacted wisdom tooth, gingivitis, No sense of taste, Ulcers on tongue, mouth ulcers), *Other endocrine/metabolic* (including Addison's disease, Beckwith - Wiedemann syndrome, Coeliac disease, Cushing's syndrome, Cystic fibrosis, Gilbert's syndrome, Hormone deficiency, deficiency of growth hormone,, dwarfism, Hypercalcemia, Hypopotassaemia, lack of potassium, Malacia, Myxoedema (nes), Obesity/overweight, Phenylketonuria, Rickets, Too much cholesterol in blood, Underactive/overactive thyroid, goitre, Water/fluid retention, Wilson's disease, but explicitly excluding Thyroid trouble and tiredness and Overactive thyroid and swelling in neck, *Other bladder problems/incontinence* (including Bed wetting, enuresis, Bladder restriction, Water trouble (nes), Weak bladder, bladder complaint (nes), but explicitly excluding Prostate trouble), *Kidney complaints* (including Chronic renal failure, Horseshoe kidney, cystic kidney, Kidney trouble, tube damage, stone in the kidney, Nephritis, pyelonephritis, Nephrotic syndrome, Only one kidney, double kidney on right side, Renal TB, Uraemia), *Reproductive system disorders* (including Abscess on breast, mastitis, cracked nipple, Amenorrhea, Damaged testicles, Endometriosis, Gynaecological problems, Hysterectomy (nes), Impotence, infertility, Menopause, Pelvic inflammatory disease/PID (female), Period problems, flooding, pre-menstrual tension/syndrome, Prolapse (nes) if female, Prolapsed womb, Prostrate gland trouble, Turner's syndrome, Vaginitis, vulvitis, dysmenorrhoea) and *Urinary tract infection* (including Cystitis, urine infection).<br>• The Epilepsy LSI is based on the group, *'Epilepsy/fits/convulsion',* including Grand mal, Petit mal, Jacksonian fit, Lennox-Gastaut syndrome, blackouts, febrile convulsions, fit (nes)<br>• The Nervous System LSI is based on the groups:<br>○ *Migraine/headaches* ○ *Other problems of nervous system*, including Abscess on brain, Alzheimer's

Measure	Details
	disease, Bell's palsy, Brain damage resulting from infection (eg. meningitis,, encephalitis) or injury, Carpal tunnel syndrome, Cerebral palsy (spastic), Degenerative brain disease, Fibromyalgia, Friedreich's Ataxia, GuillainBarre syndrome, Huntington's chorea, Hydrocephalus, microcephaly, fluid on brain, Injury to spine resulting in paralysis, Metachromatic leucodystrophy, Motor neurone disease, Multiple Sclerosis (MS), disseminated sclerosis, Muscular dystrophy, Myalgic encephalomyelitis (ME), Myasthenia gravis, Myotonic dystrophy, Neuralgia, neuritis, Numbness/loss of feeling in fingers, hand, leg etc, Paraplegia (paralysis of lower limbs), Parkinson's disease (paralysis agitans), Partially paralysed (nes), Physically handicapped - spasticity of all limbs, Pins and needles in arm, Post viral syndrome (ME), Removal of nerve in arm, Restless legs, Sciatica, Shingles, Spina bifida, Syringomyelia, Trapped nerve, Trigeminal neuralgia, Teraplegia"<br>o *Meniere's disease/ear complaints causing balance problems* (including Labryrinthitis,, loss of balance - inner ear, Vertigo).

Appendix 7: Year-by-year trends

This appendix presents the year-by-year trends for all of the variables included in the main paper. The table row labelled 'start v end sig' presents the p-value for testing the null hypothesis that there is no difference between the first and last years in the series (whichever these years are). Note that this will differ from the confidence intervals presented in the main paper as these are grouped into multi-year periods with larger sample sizes and therefore greater precision.

Table 1: Year-to-year trends in cardiovascular health

	High blood pressure LSI	Recent high blood pressure	*Biomarker high blood pressure*	Recent heart attack	IHD/stroke LSI	Heart attack symptoms	Mini stroke (TIA) symptoms	Recent angina	*Angina symptoms*
**1994**	2.2%	4.2%	8.4%	1.2%	1.4%	5.5%		1.1%	2.3%
**1995**	2.9%		8.3%		1.5%				
**1996**	3.0%		8.3%		1.5%				
**1997**	3.8%		7.7%		1.4%				
**1998**	3.1%	5.4%	7.0%	1.5%	1.3%	6.5%		1.4%	2.2%
**1999**	3.4%				1.4%				
**2000**	4.0%		6.5%		1.3%				
**2001**	4.5%		7.3%		1.7%				
**2002**	4.3%		6.1%		1.4%				
**2003**	4.5%	7.9%	4.9%	1.3%	1.3%	5.5%	8.1%	1.0%	1.8%
**2004**	4.0%				1.2%				
**2005**	5.0%		4.4%		1.3%				
**2006**	4.4%	8.7%	3.9%	1.1%	1.2%	6.2%	7.8%	0.9%	1.6%
**2007**	4.9%		4.5%		1.0%				
**2008**	5.1%		3.9%		1.1%				
**2009**	4.7%		3.2%		1.3%				
**2010**	4.6%		4.1%		1.1%				
**2011**	4.0%	9.5%	3.2%	1.0%	1.0%	5.2%	6.7%	0.7%	1.2%
**2012**			4.1%						
**2013**			3.7%						
**2014**			3.9%						
**Start v end sig.**	0.00	0.00	0.00	0.14	0.05	0.52	0.01	0.03	0.00
**N**	124,830	43,292	79,601	43,445	124,830	43,521	23,487	43,477	43,518

Table 12: Year-to-year trends in respiratory health

	COPD symptoms	Diagnosed asthma	Asthma LSI	Breathlessness Grade 2+	Breathlessness Grade 3	Recent wheezing/asthma	Wheezing stopping slee
**1994**		10.8%	4.7%				3.6%
**1995**	6.6%		4.8%	19.1%	7.6%	19.8%	
**1996**	6.6%	11.5%	5.3%	20.3%	8.0%	19.3%	3.5%
**1997**		11.9%	6.0%			18.9%	3.7%
**1998**			5.3%				
**1999**			5.7%				
**2000**			5.5%				
**2001**		14.1%	5.9%			19.9%	3.4%
**2002**			6.0%				
**2003**			5.8%				
**2004**			6.3%				
**2005**			6.1%				
**2006**			5.8%				
**2007**			5.7%				
**2008**			6.2%				
**2009**			5.5%				
**2010**	5.1%	16.6%	6.0%	15.4%	6.4%	18.4%	3.2%
**2011**			5.6%				
**2012**							
**2013**							
**2014**							
**Start v end sig.**	0.00	0.00	0.02	0.00	0.01	0.05	0.18
**N**	25,631	41,219	124,830	25,620	25,620	41,218	41,218

Table 2: Year-to-year trends in activity limitations & musculoskeletal health

Table 14: Year-to-year trends in obesity & diabetes

Year	Underweight - BMI	Obese - BMI	High waist ratio	Recent diabetes	Diabetes any	Glycated haemoglobin extreme
1994	1.1%	15.7%	9.5%	1.2%	1.5%	
1995	1.1%	17.0%			1.6%	
1996	0.9%	[illegible]			1.6%	
1997	0.9%	19.3%	12.1%		1.7%	
1998	1.0%	19.5%	11.3%	1.4%	1.5%	
1999	1.1%	20.1%	16.3%		1.9%	
2000	0.9%	21.5%			2.0%	
2001	0.9%	22.8%	[illegible]		2.1%	
2002	1.0%	23.5%	16.5%		2.1%	
2003	0.9%	[illegible]	18.7%	2.1%	2.4%	2.7%
2004	1.0%	[illegible]			2.8%	
2005	0.8%	[illegible]	21.6%		2.9%	
2006	0.8%	[illegible]	20.7%	2.7%	2.9%	3.1%
2007	1.0%	25.3%	22.1%		3.4%	
2008	0.9%	[illegible]	22.5%		2.9%	3.8%
2009	1.4%	24.3%	23.5%	3.4%	3.8%	4.3%
2010	1.1%	27.8%	24.3%	3.4%	3.5%	3.7%
2011	0.8%	25.4%	24.3%	3.6%	3.8%	5.5%
2012	1.1%	25.6%	24.0%	3.6%		4.9%
2013	1.0%	26.8%	24.2%	3.6%		4.8%
2014	0.8%	27.1%	24.7%	3.7%		4.4%
Start v end sig.	0.00	0.01	0.05	0.04	0.01	0.00
N	[illegible]	62,612	[illegible]	[illegible]	[illegible]2,692	62,69[illegible]

Overlapping values from Table 2 (activity limitations & musculoskeletal health):

Year	Problems walking about today	Walking limitation	Any locomotor limitation	Washing/dressing problems today	Washing/dressing limitation	Any self care limitation	Pain	Pain	LSI	Other musculoskeletal LSI
1994			6.8%		1.9%	3.9%			4.9%	8.9%
1995										9.9%
1996				[illegible]			[illegible]2.0%	3.[illegible]		10.3%
1997									6.0%	11.4%
1998										11.7%
1999										11.0%
2000			[illegible]%							10.7%
2001		5.9%	7.8%		[illegible]	4.7%			6.1%	10.9%
2002										12.3%
2003				[illegible]			[illegible]7.1%	3.[illegible]		11.8%
2004	11.6%			3.6%			28.6%	3.5%	6.3%	11.6%
2005				4.0%			[illegible]7.8%	3.[illegible]		11.3%
2006				3.6%			[illegible]6.8%	3.[illegible]		10.1%
2007										9.9%
2008	11.5%			3.6%			28.1%	3.1%	4.7%	9.5%
2009										9.0%
2010				4.1%			[illegible]9.9%	3.[illegible]		10.3%
2011	13.6%			4.0%			34.0%	4.0%	4.9%	9.2%
2012				3.8%			[illegible]7.4%	3.[illegible]		
2013										
2014				4.2%			[illegible]7.7%	3.[illegible]		
Start v end sig.								0.89	0.97	0.57
N									[illegible]30	124,830

Table 3: Year-to-year trends in other biomarkers

	High total cholesterol	Low HDL cholesterol	Raised C-reactive protein	Raised fibrinogen	Anaemia	Iron deficiency	Cancer LSI
**1994**	75.7%				6.7%	39.9%	0.2%
**1995**							0.3%
**1996**							0.3%
**1997**							0.4%
**1998**	64.8%	11.8%	21.4%	2.3%	6.3%	38.2%	0.5%
**1999**							0.4%
**2000**							0.5%
**2001**							0.5%
**2002**							0.5%
**2003**	71.4%	4.0%	24.1%	5.7%			0.6%
**2004**							0.6%
**2005**							0.6%
**2006**	67.2%	5.1%	22.7%	5.7%	4.6%	29.3%	0.7%
**2007**							0.5%
**2008**	66.7%	4.3%					0.6%
**2009**	66.9%	4.5%	23.5%	3.8%	5.3%	27.0%	0.5%
**2010**	64.1%	4.6%					0.8%
**2011**	60.2%	4.5%					0.8%
**2012**	64.0%	4.4%					
**2013**	58.0%	3.4%					
**2014**	55.4%	2.9%					
**Start v end sig.**	0.00	0.00	0.11	0.01	0.04	0.00	0.00
**N**	41,224	33,937	17,749	16,105	20,228	20,304	124,830

	Mental health LSI	Psychological distress symptoms	Anxiety/depression - moderately	Anxiety/depression - extremely
**1994**	1.8%	16.1%		
**1995**	2.3%	18.0%		
**1996**	2.4%		21.9%	1.8%
**1997**	2.9%	16.5%		
**1998**	3.0%	15.6%		
**1999**	3.0%	17.7%		

Table 16: Year-to-year trends in mental health

**2000**	3.5%	14.4%		
**2001**	3.3%	13.7%		
**2002**	3.1%	16.6%		
**2003**	3.7%	13.5%	18.5%	1.9%
**2004**	3.6%	13.4%	18.8%	2.1%
**2005**	4.4%	14.0%	19.6%	2.1%
**2006**	4.1%	13.9%	18.8%	2.1%
**2007**	4.5%			
**2008**	4.2%	13.7%	18.5%	2.0%
**2009**	4.9%	17.1%		
**2010**	5.2%	16.1%	23.5%	2.7%
**2011**	4.6%		26.8%	3.0%
**2012**		16.0%	20.0%	2.7%
**2013**				
**2014**		15.6%	19.6%	2.5%
**Start v end sig.**	0.00	0.47	0.01	0.02
**N**	124,830	107,834	62,635	62,635

Appendix 8: Others' analyses over change over time using HSE data

Changes over time in some of these indicators have not previously been analysed (e.g. waist-hip ratio, fibrinogen). However, others have been studied but never integrated into a single picture of changing morbidity; we review these in this section. (For reasons of space these are included here rather than in the main text).

Cardiovascular morbidity

1998-2011 trends in the two biomarkers for total and HDL cholesterol using HSE data are shown in Oyebode,[11] who find similar results.

Respiratory morbidity

A subset of the HSE respiratory indicators (ever/past year wheezing, doctor-diagnosed asthma) were analysed by Hall and Mindell[31] looking at 2001-2010, and finding similar changes over time to our analysis. They found stability in some measures (ever wheezing) but improvements in others

(pastyear wheezing) – at the same time as the reported prevalence of doctor-diagnosed asthma increased.

Obesity & diabetes

While the English trends in waist-hip ratio have not previously been analysed, earlier Scottish trends are given in Hotchkiss et al 2012.[19] Trends in diabetes have been covered in several HSE reports, e.g. Moody 2012,[20] as has BMI (see particularly the paper by Sperrin et al 2014,[32] who also created a publicly-available time-series HSE dataset for this purpose).

Activity limitations, pain & musculoskeletal morbidity

While musculoskeletal LSIs have not previously been analysed in HSE, a decline can also be seen in the General Household Survey.[33]

Mental health

In the UK and most other high-income countries, benefit claims due to mental ill-health have been rising,[34] which has come alongside considerable increases in mental health diagnosis and treatment.[35] The extent to which this reflects rises in mental ill-health and genuinely declining work capacity, however, has long been the subject of debate.[36 37] Perhaps the most robust long-term general population data series in the UK is the Adult Psychiatric Morbidity Survey.[35 38]

While some studies have used HSE to show rises in mental ill-health, others have used the same data to come to the opposite conclusion.[39 40] These contrasting conclusions are explained by the tables in Web Appendix 7 which show year-by-year changes: moderate mental ill-health fell between the mid1990s and the mid-2000s, before rising in 2009, and with a particularly high prevalence in 2011. The conclusions of studies will therefore depend on the years they use as their start and end periods for the trend analysis.[3] It is also worth noting that our results for considerable increases in mental health LSIs can also be seen in a similar measure in the Labour Force Survey.[41 42]

Other morbidity measures

While CRP and fibrinogen are collected in HSE at considerable efforts, their trends have rarely been studied (e.g. they appear only in supplementary descriptive tables in Hughes et al [23]). A decline in anaemia using HSE data 1998-2005 has been observed by Tull et al 2009,[43] but this has not hitherto been updated to the 2008-10 period.

It has been suggested that multimorbidity has risen among older people in England [44] and for all age groups in Ontario,[45] although others have cautioned against using simple disease counts,[46] and the evidence cited in the introduction of the main paper suggests that rising chronic disease reporting may partly be a result of increasing awareness (rather than underlying prevalence) of disease.

[3] The major explanation why 'moderate anxiety/depression today' does not show a decline 2011-14 compared to 1994-6 is because of a single very high reported prevalence in 2011, which had reduced by 2012 and 2014.
The alternate measure ('psychological distress symptoms') was not asked in 2011.

Appendix 9: Summarising multiple measures

Having reviewed trends in 39 morbidity measures, we have seen that morbidity in the English working-age population has improved in some respects and deteriorated in others. For those who view work-related morbidity as intrinsically multidimensional,[47], this is the endpoint of our analysis. However, for those who conceive of morbidity as unidimensional – or those who are interested in morbidity as it relates to a unidimensional work capacity – this raises the question of how we weight different dimensions of morbidity to decide if the overall change in morbidity has been positive or negative.

Methods for creating unidimensional morbidity scales

Several methods have been proposed for creating unidimensional morbidity scales, but most of these are unavailable using the HSE data:

- Weights can be based on empirically-derived preferences for different health states, of which the most famous example is the WHO Global Burden of Disease (GBD) study [48]. Some GBD estimates for trends in disability in the UK do exist, and suggest that the prevalence of disability in the working-age population is unchanged 1990-2010, though these results are only presented in passing.[4] For our analyses, however, we have no preference-based weights for most of the HSE measures (excluding the subset of measures that make up the EQ-5D scale).
- Those reporting limitations beyond a certain severity in any domain can be categorised as 'disabled', as recommended by the Washington Group on Disability Statistics (see above). However, as previously discussed, we have few functional limitations measures available in HSE.
- Latent morbidity scales can be created based on the inter-correlations between different measures (using item response theory), as used in the World Disability Report [51] and by researchers associated with the US National Bureau of Economic Research e.g. [52]. However, it is unclear why we would wish to weight items in this way: a given morbidity indicator may be severe, yet if it is unrelated to other morbidity measures it will be given a low weight.
- Latent morbidity scales can also be created based on the independent correlation between each indicator and a general measure of morbidity, such as general self-reported health or [53] as in [54]. This maintains some of the advantages of single-item measures (in providing a basis for making morbidity unidimensional), while avoiding the potential threats to validity discussed above. However, the inconsistent inclusion of measures in each HSE wave prevents a unidimensional morbidity scale being constructed here.

[4] Trends in the UK GBD results are reported in Murray et al.[49] However, Murray et al do not focus on trends in years lived with disability (YLD), other than to note that *"YLDs per person by age and sex have not changed substantially in the UK, but age-specific mortality has been improving"* (p1005). The figure in the supplementary appendix shows that YLDs have barely changed for either men or women at any age. However, the confidence intervals for YLDs as a whole in the main paper (Table 1) suggest that the confidence intervals for these trends are very wide. The public GBD data [50] do provide cause-disaggregated YLDs for the UK (and all other countries) for a slightly different period (2000-2015), but are not age-standardised, are within broad age groups only (e.g. 15-29), and again lack estimates of uncertainty.

Geiger BB. *BMJ Open* 2021; 10:e032378. doi: 10.1136/bmjopen-2019-032378

An alternative way of summarising heterogeneous trends

Nevertheless, we can examine if the areas in which morbidity has been improving or declining are those that are particularly important for general health.[53] (This uses the same intuition as the scales in Diederichs et al 2012).[54] To see how important measures are for general health, we regress 'bad' general health (see Appendix 5 for detail on the underlying question) on age, sex (and their interaction), educational level and each individual morbidity measure in turn, using all years for which that morbidity measure is available. That is, for each morbidity indicator morbidity we use the following model:

badhealth =logit β morbidity + +β +
!" *male$%+ &'() *+,-

… where β is our primary outcome coefficient showing the importance of that morbidity indicator for bad health, refers to a vector of age dummy variables, male$ refers to a binary gender dummy variable, '() *+, refers to a vector of education dummy variables (with four levels: degree/full-time student, A-levels/NVQ3/higher education below degree, other qualifications, or no qualifications), and , . , !, and & refer to the coefficients on age, gender, their interaction and education respectively.

We adjust for education as well as age & sex to enable us to examine the importance of the measure for bad health, after taking account of whether general health and the measure are both strongly related to social status. Note however that it is not possible to control for all morbidity measures simultaneously (as we discuss just above) – so this is a rough indicator of the importance of that morbidity measure for general health, rather than a reliable indicator of the causal impact net of comorbidities.

The results of this analysis are shown overleaf, ordered by the effect on bad health. (We also repeat the trend in each measure for convenience; this is discussed following the table).

Measure	Type	Effect on bad health *(95% CI)*		Change over time in measure *(95% CI)*	
Pain-extreme	S	46.4%	*[44.0, 48.9%]*	0.2%	*[-0.3, 0.7%]*
Problems washing/dressing today	S	43.7%	*[41.4, 46.0%]*	0.3%	*[-0.2, 0.9%]*
Anxiety/depression-extremely	S	35.4%	*[32.8, 38.0%]*	0.9%	*[0.5, 1.3%]*
Any locomotor limitation	S	33.6%	*[31.2, 36.0%]*	0.9%	*[0.1, 1.7%]*
Any self-care limitation	S	32.6%	*[29.7, 35.5%]*	0.7%	*[0.1, 1.3%]*
Problems walking about today	S	26.3%	*[25.2, 27.4%]*	0.4%	*[-0.6, 1.3%]*
High psychological distress	S	26.4%	*[24.9, 27.9%]*	0.9%	*[0.4, 1.4%]*
Recent angina	L	23.8%	*[20.1, 27.5%]*	-0.5%	*[-0.8, -0.1%]*
Recent heart attack/stroke	L	23.2%	*[19.7, 26.7%]*	-0.4%	*[-0.7, 0.0%]*
Breathlessness-Grade 3	S	22.9%	*[20.9, 24.9%]*	-1.6%	*[-2.5, -0.8%]*
Mental health LSI	L	20.4%	*[19.1, 21.7%]*	2.4%	*[1.8, 3.0%]*
IHD/stroke LSI	L	19.7%	*[17.9, 21.5%]*	-0.6%	*[-0.9, -0.2%]*
Wheezing stopping sleep	S	19.1%	*[17.1, 21.1%]*	-0.5%	*[-1.0, 0.1%]*
Mini stroke (TIA) symptoms	S	16.8%	*[15.0, 18.6%]*	-1.4%	*[-2.4, -0.4%]*
Angina symptoms	S	16.6%	*[14.1, 19.1%]*	-1.2%	*[-1.6, -0.7%]*
Psychological distress symptoms	S	15.2%	*[14.6, 15.8%]*	-1.3%	*[-2.4, -0.3%]*
Arthritis LSI	L	15.2%	*[14.3, 16.1%]*	-0.7%	*[-1.4, 0.0%]*
Any recent CVD	L	14.4%	*[12.7, 16.1%]*	0.5%	*[-0.1, 1.2%]*
Heart attack symptoms	S	14.1%	*[12.6, 15.6%]*	-0.5%	*[-1.3, 0.3%]*

Anxiety/depression-moderately	S	13.6%	*[13.0, 14.2%]*	0.1%	*[-1.1, 1.3%]*
Pain-any	S	12.9%	*[12.4, 13.4%]*	-3.3%	*[-4.6, -2.0%]*
COPD symptoms	S	12.6%	*[11.0, 14.2%]*	-1.6%	*[-2.3, -0.8%]*
Diabetes LSI	L	12.4%	*[11.1, 13.7%]*	2.1%	*[1.5, 2.6%]*
Recent diabetes	L	11.8%	*[10.2, 13.4%]*	2.2%	*[1.9, 2.6%]*
Breathlessness-Grade 2+	S	11.5%	*[10.5, 12.5%]*	-4.8%	*[-6.1, -3.5%]*
Any CVD LSI	L	11.0%	*[10.3, 11.7%]*	0.6%	*[-0.1, 1.4%]*
Other musculoskeletal LSI	L	9.8%	*[9.2, 10.4%]*	-0.8%	*[-1.7, 0.1%]*
Glycated haemoglobin	B	9.9%	*[7.9, 11.9%]*	2.1%	*[1.4, 2.7%]*
Asthma LSI	L	8.6%	*[7.8, 9.4%]*	0.7%	*[0.0, 1.4%]*
Recent wheezing/asthma	S	8.4%	*[7.7, 9.1%]*	-1.2%	*[-2.5, 0.1%]*
Recent high blood pressure	L	6.8%	*[5.7, 7.9%]*	4.8%	*[3.9, 5.6%]*
BMI-Underweight	B	6.2%	*[4.3, 8.1%]*	-0.1%	*[-0.3, 0.1%]*
Diagnosed asthma	L	5.9%	*[5.1, 6.7%]*	5.7%	*[4.5, 6.8%]*
High waist-hip ratio	B	4.6%	*[4.1, 5.1%]*	14.1%	*[13.0, 15.2%]*
Raised fibrinogen	B	4.3%	*[1.9, 6.7%]*	1.5%	*[0.3, 2.6%]*
Low HDL cholesterol	B	4.3%	*[2.8, 5.8%]*	-8.0%	*[-9.0, -7.1%]*
Raised C-reactive protein	B	3.7%	*[2.7, 4.7%]*	1.9%	*[-0.7, 4.5%]*
BMI-Obese	B	2.8%	*[2.5, 3.1%]*	8.9%	*[8.0, 9.7%]*
Anaemia	B	2.4%	*[0.8, 4.0%]*	-1.4%	*[-2.7, -0.1%]*
Biomarker high blood pressure	B	0.4%	*[-0.3, 1.1%]*	-5.0%	*[-5.6, -4.5%]*
High total cholesterol	B	0.0%	*[-0.6, 0.6%]*	-17.6%	*[-19.1, -16.1%]*
Iron deficiency	B	-0.5%	*[-1.3, 0.3%]*	-12.5%	*[-14.8, -10.2%]*

Having estimated this, we can see if the areas in which morbidity has been improving or declining are those that are particularly important for general health. This is shown visually in Figure 1 below (the measures are not labelled to enable the overall pattern to be seen, but the top-to-bottom order of measures is the same in the figure as in the preceding table; i.e. the measure at the top of the figure is 'Pain-extreme').

Figure 1: Change over time in morbidity measures & their association with bad general health[a]

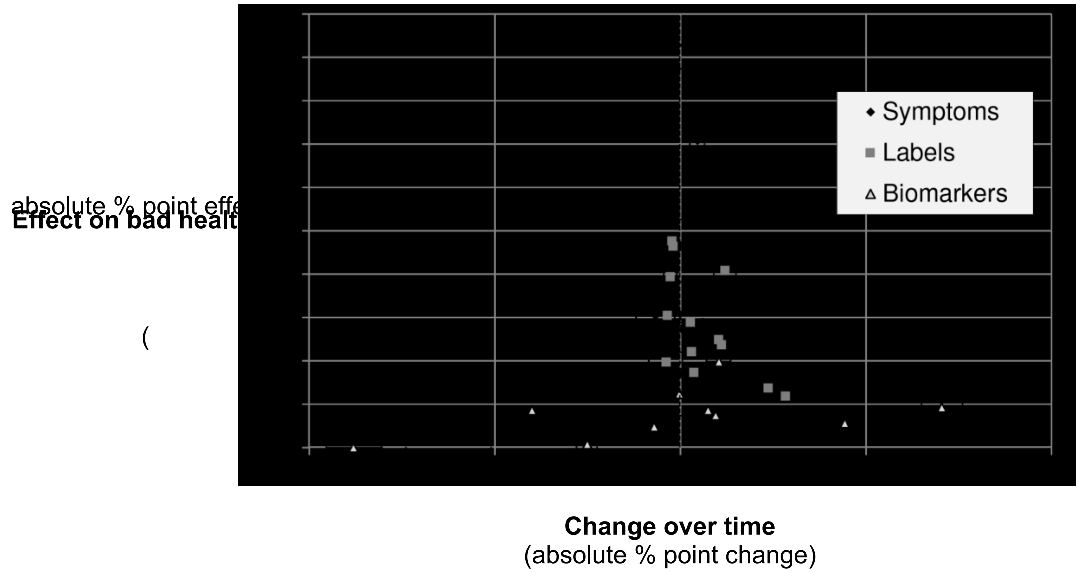

[a] 'Trend' is as reported above in the main paper. 'Effect on bad health' shows the effect of the morbidity measure on (very) bad health after controlling for age, sex (and their interaction) and educational level, using all years for which the individual morbidity measure is available. (This shows average marginal effects following a logistic regression; see text above).

It is easiest to interpret the figure by focussing on each group of measures in turn. Firstly, the biomarkers tend to have the weakest relationship with general health. Those with high levels of the diabetes biomarker (glycated haemoglobin) are 9.7% more likely to say they have bad health, and those who are underweight, with a high waist-hip ratio, raised fibrinogen, or low HDL cholesterol are 4-6% more likely to report bad health, but the other measures only had weaker relationships. Indeed, there was effectively no relationship between bad reported health and any of measured high blood pressure, high total cholesterol or iron deficiency.

Secondly, most of the measures based on medical labels have a moderately strong relationship with bad health (the weakest being lifetime asthma and recent high blood pressure, both of which can be asymptomatic), and these measures have mostly risen over time. There are however notable exceptions to this, including IHD/stroke LSI, recent angina and recent heart attack/stroke (the labelbased measures with some of the strongest relationships with bad reported health), as well as arthritis and other musculoskeletal LSIs.

Finally, symptom-based measures unsurprisingly tend to have stronger relationships with bad reported health, although this ranges from the moderate (those reporting 'recent wheezing/asthma attack' were 8.5% more likely to report bad health) to the very strong (those reporting 'extreme pain today' were 46.4% more likely to report bad health). In general, those symptoms-based measures with the strongest relationship with bad reported health were more likely to have increased over time ('extreme anxiety/depression today', 'locomotor limitations', and 'self-care limitations'). However, the size of the aforementioned declines in symptom-based measures of respiratory and cardiovascular morbidity was often greater.

Bibliography for Web Appendices

1. Case A, Deaton A. Rising morbidity and mortality in midlife among white non-Hispanic Americans in the 21st century. *Proceedings of the National Academy of Sciences* 2015;112(49):15078-83. doi: 10.1073/pnas.1518393112
2. Hiam L, Dorling D, Harrison D, et al. Why has mortality in England and Wales been increasing? An iterative demographic analysis. *Journal of the Royal Society of Medicine* 2017;110(4):153-62. doi: 10.1177/0141076817693599
3. Department of Health. Our Health and Wellbeing Today. London: HM Government, 2010.
4. Jagger C. Trends in life expectancy and healthy life expectancy. Future of an ageing population: evidence review. London: Foresight, Government Office for Science, 2015.
5. Office for Budget Responsibility. Welfare trends report: October 2016. Cm 9341. London: Her Majesty's Stationery Office, 2016.
6. Department for Work and Pensions, Department of Health. Improving Lives: The Work, Health and Disability Green Paper. Cm 9342. London: Her Majesty's Stationery Office, 2016.
7. Geiger BB. Morbidity in England 1994-2014 2019 [Available from:http://osf.io/dy6sv].
8. Szende A, Janssen B, Cabases J, editors. *Self-Reported Population Health: An International Perspective based on EQ-5D*. Netherlands: Springer, 2014.
9. Erens B, Primatesta P, Prior G. Health survey for England 1999: the health of minority ethnic groups. London: The Stationery Office, 2001.
10. National Heart Lung and Blood Institute. Sixth Report of the Joint National Committee on the Prevention, Detection, Evaluation and Treatment of High Blood Pressure. NIH Publication No 98-04080: National Institutes on Health, 1997.
11. Oyebode O. Cardiovascular disease. In: Craig R, Mindell J, eds. Health Survey for England 2011: Volume 1 - Health, social care and lifestyles. Leeds, UK: Health and Social Care Information Centre 2012.
12. Banks J, Marmot M, Oldfield Z, et al. The SES Health Gradient on Both Sides of the Atlantic. NBER Working Paper No 12674, 2006.
13. Lawlor DA, Adamson J, Ebrahim S. Performance of the WHO Rose angina questionnaire in postmenopausal women: Are all of the questions necessary? *Journal of Epidemiology and Community Health* 2003;57(7):538-41. doi: 10.1136/jech.57.7.538
14. Cook D, Shaper A, Macfarlane P. Using the WHO (Rose) Angina Questionnaire in Cardiovascular Epidemiology. *International Journal of Epidemiology* 1989;18(3):607-13. doi: 10.1093/ije/18.3.607
15. Fletcher C, Elmes P, Fairbairn M, et al. The significance of respiratory symptoms and the diagnosis of chronic bronchitis in a working population. *BMJ* 1959;2:257-66.
16. WHO. Physical status: the use and interpretation of anthropometry. Report of a WHO Expert Committee. WHO Technical Report Series 854. Geneva: World Health Organization (WHO), 1995.
17. National Obesity Observatory. Obesity and Overweight Surveillance in England: what is measured and where are the gaps?, 2009.
18. NICE. Obesity: The Prevention, Identification, Assessment and Management of Overweight and Obesity in Adults and Children NICE Clinical Guidelines, No 43. London: Centre for Public Health Excellence and National Collaborating Centre for Primary Care at the National Institute for Health and Clinical Excellence (NICE), 2006.

19. Hotchkiss JW, Davies CA, Gray L, et al. Trends in cardiovascular disease biomarkers and their socioeconomic patterning among adults in the Scottish population 1995 to 2009: crosssectional surveys. *BMJ Open* 2012;2(3) doi: 10.1136/bmjopen-2011-000771
20. Moody A. Diabetes and hyperglycaemia. In: Craig R, Mindell J, eds. Health Survey for England 2011: Volume 1 - Health, social care and lifestyles. Leeds, UK: Health and Social Care Information Centre 2012.
21. Aresu M, Gordon-Dseagu V, Shelton N. Diabetes and glycaemia. In: Craig R, Hirani V, eds. Health Survey for England 2009, Volume 1: Health and lifestyles. Leeds, UK: The NHS Information Centre for health and social care 2010:59-74.
22. Steptoe A. Psychosocial biomarker research: integrating social, emotional and economic factors into population studies of aging and health. *Social Cognitive and Affective Neuroscience* 2011;6(2):226-33. doi: 10.1093/scan/nsq032
23. Hughes A, McMunn A, Bartley M, et al. Elevated inflammatory biomarkers during unemployment: modification by age and country in the UK. *Journal of Epidemiology and Community Health* 2015;69(7):673-79. doi: 10.1136/jech-2014-204404
24. Benzeval M, Davillas A, Kumari M, et al. Understanding Society: The UK Household Longitudinal Study Biomarker User Guide and Glossary (version 1). Colchester, Essex: Institute for Social and Economic Research, 2014.
25. Chaudhury M. Blood analytes. In: Sproston K, Primatesta P, eds. Health Survey for England, 2003, Vol 2: Risk Factors for Cardiovascular Disease: TSO 2004:241-88.
26. Erens B, Primatesta P. Health survey for England 1998: cardiovascular disease. *London: The Stationery Office* 1999
27. Chaudhury M, Tull K. Nutrition and haematological status. In: Craig R, Mindell J, eds. Health Survey for England, 2005: The health of older people, Vol 1: General health and function: TSO 2006:67-96.
28. Goldberg D, PA W. User Guide to the General Health Questionnaire. Windsor, UK: NFERNelson, 1988.
29. Stochl J, Böhnke JR, Pickett KE, et al. An evaluation of computerized adaptive testing for general psychological distress: combining GHQ-12 and Affectometer-2 in an item bank for public mental health research. *BMC Medical Research Methodology* 2016;16(1):58. doi: 10.1186/s12874-016-0158-7
30. ONS. Harmonised Concepts and Questions for Social Data Sources, Primary Principles: Longlasting Health Conditions and Illnesses; Impairments and Disability [version 1.1]. London: Office for National Statistics (ONS), 2015.
31. Hall J, Mindell J. Respiratory symptoms and disease in adults In: Craig R, Mindell J, eds. Health Survey for England, 2010, Volume 1: Respiratory health: TSO 2011.
32. Sperrin M, Marshall A, Higgins V, et al. Slowing down of adult body mass index trend increases in England: a latent class analysis of cross-sectional surveys (1992-2010). *International Journal of Obesity* 2014;38(6)
33. Parsons S, Ingram M, Clarke-Cornwell AM, et al. A Heavy Burden: The occurrence and impact of musculoskeletal conditions in the United Kingdom today. Manchester: Arthritis Research UK & University of Manchester, 2011.
34. OECD. Fit Mind, Fit Job: From evidence to practice in mental health and work. Paris: OECD, 2015.
35. Spiers N, Qassem T, Bebbington P, et al. Prevalence and treatment of common mental disorders in the English national population, 1993–2007. *The British Journal of Psychiatry* 2016;209(2):150-56. doi: 10.1192/bjp.bp.115.174979

36. Moncrieff J, Pomerleau J. Trends in sickness benefits in Great Britain and the contribution of mental disorders. *Journal of Public Health Medicine* 2000;22:59-67.
37. Stansfeld SA, Woodley-Jones D, Rasul F, et al. Work-related distress in the 1990s - a real increase in ill health? . *Journal of Public Mental Health* 2008;7(1):25-31.
38. McManus S, Bebbington P, Jenkins R, et al., editors. *Mental health and wellbeing in England: Adult Psychiatric Morbidity Survey 2014*. Leeds: NHS Digital, 2016.
39. Maheswaran H, Kupek E, Petrou S. Self-reported health and socio-economic inequalities in England, 1996–2009: Repeated national cross-sectional study. *Social Science & Medicine* 2015;136–137:135-46. doi: http://dx.doi.org/10.1016/j.socscimed.2015.05.026
40. Katikireddi SV, Niedzwiedz CL, Popham F. Trends in population mental health before and after the 2008 recession: a repeat cross-sectional analysis of the 1991–2010 Health Surveys of England. *BMJ Open* 2012;2(5) doi: 10.1136/bmjopen-2012-001790
41. Jones M, Wass V. Understanding changing disability-related employment gaps in Britain 1998–2011. *Work, Employment & Society* 2013;27(6):982-1003. doi: 10.1177/0950017013475372
42. Barr B, Kinderman P, Whitehead M. Trends in mental health inequalities in England during a period of recession, austerity and welfare reform 2004 to 2013. *Social Science & Medicine* 2015;147:324-31. doi: http://dx.doi.org/10.1016/j.socscimed.2015.11.009
43. Tull KI, Hirani V, Ali A, et al. Impact of different diagnostic thresholds and the anaemia–ferritin–transferrin receptor model on the prevalence of anaemia and impaired iron status in older people. *Age and Ageing* 2009;38(5):609-13. doi: 10.1093/ageing/afp102
44. Dhalwani NN, O'Donovan G, Zaccardi F, et al. Long terms trends of multimorbidity and association with physical activity in older English population. *International Journal of Behavioral Nutrition and Physical Activity* 2016;13(1):8. doi: 10.1186/s12966-016-0330-9
45. Koné Pefoyo AJ, Bronskill SE, Gruneir A, et al. The increasing burden and complexity of multimorbidity. *BMC Public Health* 2015;15(1):415. doi: 10.1186/s12889-015-1733-2
46. Tetzlaff J, Junius-Walker U, Muschik D, et al. Identifying time trends in multimorbidity—defining multimorbidity in times of changing diagnostic practices. *Journal of Public Health* 2016:1-8. doi: 10.1007/s10389-016-0771-2
47. Marfeo EE, Haley SM, Jette AM, et al. Conceptual Foundation for Measures of Physical Function and Behavioral Health Function for Social Security Work Disability Evaluation. *Archives of Physical Medicine and Rehabilitation* 2013;94(9):1645-52.e2. doi: 10.1016/j.apmr.2013.03.015
48. Salomon JA, Vos T, Hogan DR, et al. Common values in assessing health outcomes from disease and injury: disability weights measurement study for the Global Burden of Disease Study 2010. *The Lancet* 2012;380(9859):2129-43. doi: http://dx.doi.org/10.1016/S01406736(12)61680-8
49. Murray CJL, Richards MA, Newton JN, et al. UK health performance: findings of the Global Burden of Disease Study 2010. *The Lancet* 2013;381(9871):997-1020. doi: https://doi.org/10.1016/S0140-6736(13)60355-4
50. WHO. Global Health Estimates 2015: Disease burden by Cause, Age, Sex, by Country and by Region, 2000-2015. Geneva: World Health Organization (WHO), 2016.
51. WHO. World report on disability. Geneva: World Health Organization (WHO) 2011.
52. Soldo BJ, Mitchell OS, Tfaily R, et al. Cross-cohort differences in health on the verge of retirement. NBER Working Paper No 12762: National Bureau of Economic Research, 2006.
53. Stewart ST, Cutler DM, Rosen AB. Comparison of Trends in U.S. Health-Related Quality of Life over the 2000's Using the SF-6D, HALex, EQ-5D, and EQ-5D Visual Analog Scale versus a Broader Set of Symptoms and Impairments. *Medical care* 2014;52(12):1010-16. doi: 10.1097/MLR.0000000000000181

54. Diederichs CP, Wellmann J, Bartels DB, et al. How to weight chronic diseases in multimorbidity indices? Development of a new method on the basis of individual data from five populationbased studies. *Journal of Clinical Epidemiology* 2012;65(6):679-85. doi: 10.1016/j.jclinepi.2011.11.006
