# Supplementary material for: Has working-age morbidity been declining? Changes over time in survey measures of general health, chronic diseases, symptoms and biomarkers in England 1994–2014
Source: BMJ Open. 2020 Mar 15;10(3):e032378. doi: 10.1136/bmjopen-2019-032378 (PMC7073795; doi:10.1136/bmjopen-2019-032378)
Supplement: Author's manuscript [file bmjopen-2019-032378.draft_revisions.pdf]

BMJ Open is committed to open peer review. As part of this commitment we make the peer review history of every article we publish publicly available.

When an article is published we post the peer reviewers' comments and the authors' responses online. We also post the versions of the paper that were used during peer review. These are the versions that the peer review comments apply to.

The versions of the paper that follow are the versions that were submitted during the peer review process. They are not the versions of record or the final published versions. They should not be cited or distributed as the published version of this manuscript.

BMJ Open is an open access journal and the full, final, typeset and author-corrected version of record of the manuscript is available on our site with no access controls, subscription charges or pay-per-view fees (http://bmjopen.bmj.com).

If you have any questions on BMJ Open's open peer review process please email info.bmjopen@bmj.com

BMJ Open

Has working-age morbidity been declining? Trends in general health, chronic diseases, symptoms and biomarkers in England 1994-2014

Journal:	*BMJ Open*
Manuscript ID	bmjopen-2019-032378
Article Type:	Research
Date Submitted by the Author:	17-Jun-2019
Complete List of Authors:	Geiger, Ben; University of Kent, School of Social Policy, Sociology and Social Research (SSPSSR);
Keywords:	Morbidity, Population Health, Biomarkers, Cross-sectional studies, Trends, England

SCHOLARONE™
Manuscripts

BMJ

I, the Submitting Author has the right to grant and does grant on behalf of all authors of the Work (as defined in the below author licence), an exclusive licence and/or a non-exclusive licence for contributions from authors who are: i) UK Crown employees; ii) where BMJ has agreed a CC-BY licence shall apply, and/or iii) in accordance with the terms applicable for US Federal Government officers or employees acting as part of their official duties; on a worldwide, perpetual, irrevocable, royalty-free basis to BMJ Publishing Group Ltd ("BMJ") its licensees and where the relevant Journal is co-owned by BMJ to the co-owners of the Journal, to publish the Work in this journal and any other BMJ products and to exploit all rights, as set out in our licence.

The Submitting Author accepts and understands that any supply made under these terms is made by BMJ to the Submitting Author unless you are acting as an employee on behalf of your employer or a postgraduate student of an affiliated institution which is paying any applicable article publishing charge ("APC") for Open Access articles. Where the Submitting Author wishes to make the Work available on an Open Access basis (and intends to pay the relevant APC), the terms of reuse of such Open Access shall be governed by a Creative Commons licence – details of these licences and which Creative Commons licence will apply to this Work are set out in our licence referred to above.

Other than as permitted in any relevant BMJ Author's Self Archiving Policies, I confirm this Work has not been accepted for publication elsewhere, is not being considered for publication elsewhere and does not duplicate material already published. I confirm all authors consent to publication of this Work and authorise the granting of this licence.

For peer review only

Has working-age morbidity been declining? Trends in general health, chronic diseases, symptoms and biomarkers in England 1994-2014

Authors:

Dr Ben B. Geiger, University of Kent, UK

Postal address: Ben B Geiger, School of Social Policy, Sociology and Social Research (SSPSSR), University of Kent, Canterbury, Kent, CT2 7NZ, UK

Email: b.b.geiger@kent.ac.uk

Word count *(exc. title page, abstract, tables, acknowledgements, contributions & references)*

3,240 words + 5 tables

MeSH Keywords

Morbidity; Population Health; Biomarkers; Cross-Sectional Studies; Trends; England

Acknowledgements

This work was supported by the Economic and Social Research Council (grant number ES/K009583/1).

For peer review only - http://bmjopen.bmj.com/site/about/guidelines.xhtml

For peer review only

Author statement

BBG was responsible for the design, data preparation, analysis and reporting of the study. A research assistant (Mariska van der Horst) briefly helped with some of the data preparation.

Acknowledgements

Many thanks to Clare Bambra for comments, and to Mariska van der Horst for research assistance; neither should be held responsible for the analysis or interpretation of the paper itself.

Funding

This work was supported by the Economic and Social Research Council (ESRC), grant number ES/K009583/1.

Competing interests

The author has worked on secondment at the UK Department for Work and Pensions (DWP) in 2015-16.

Data sharing

The statistical code enabling replication using publicly available data will be made available from www.benbgeiger.co.uk at the point the article is accepted.

For peer review only

Has working-age morbidity been declining? Trends in general health, chronic diseases, symptoms and biomarkers in England 1994-2014

Abstract:

Objectives: As life expectancy has increased in high-income countries, there has been a global debate about whether additional years of life are free from ill-health/disability. However, little attention has been given to morbidity trends in the *working-age* population, particularly outside the US, despite its importance for health monitoring and social policy. This study therefore asks: what are the trends in working-age morbidity in England over two decades?

Design, setting and participants: We use a high-quality annual cross-sectional survey, the Health Survey for England ('HSE') 1994-2014. HSE uses a random sample of the English household population, with a combined sample size of over 140,000 people. We produce a newly-harmonised version of HSE that maximises comparability over time, including new non-response weights. While HSE is used for monitoring population health, it has hitherto not used for investigating morbidity as a whole.

Outcome measures: We analyse all 39 measures that are fully comparable over time – including chronic disease diagnoses, symptomology and a number of biomarkers – adjusting for gender and age.

Results: We find a mixed picture: we see improving cardiovascular and respiratory health, but deteriorations in obesity, diabetes, some biomarkers, and mental ill-

For peer review only - http://bmjopen.bmj.com/site/about/guidelines.xhtml

health at the highest levels, alongside stability in moderate mental ill-health and musculoskeletal-related health. In several domains we also see stable or rising chronic disease *diagnoses* even where *symptomology* has declined. While data limitations make it challenging to combine these measures into a single morbidity index, there is little systematic trend for declining morbidity to be seen in the measures that predict self-reported health most strongly.

Conclusions: Despite considerable falls in working-age mortality – and the assumptions of many policymakers that morbidity will follow mortality – there is no systematic improvement in overall working-age morbidity in England from 1994 to 2014.

Strengths and limitations of this study

- We provide a robust analysis of morbidity trends in England for 39 measures across two decades using the Health Survey for England ('HSE').
- We include every morbidity measure for which consistent trends can be constructed in the HSE.
- We take care to maximise comparability over time, including constructing new non-response weights.
- However, response rates for each stage of the HSE have declined over time, and it is impossible to rule out changing non-response biases.

For peer review only - http://bmjopen.bmj.com/site/about/guidelines.xhtml

- There are also several dimensions of morbidity for which there is little trend data in HSE.

For peer review only

For peer review only - http://bmjopen.bmj.com/site/about/guidelines.xhtml

INTRODUCTION

As life expectancy has increased in high-income countries, there has been a global debate about whether additional years of life are free from ill-health/disability. It is now largely accepted that old-age disability has declined in the US (albeit varying by age/method),[1 2] although chronic illness increased,[3] and the picture beyond the US is more mixed.[4-6] Yet this research agenda has not been matched by similar attention to morbidity trends in the *working-age* population. In the absence of evidence, policymakers have either made claims based on self-reports of general health[6-8] (which are unreliable, as we explain below), or in the case of social security, have assumed that working-age morbidity *must* have improved in recent decades given improvements in mortality[9 10] (despite the potential for declining mortality to coexist with rising morbidity).[6]

Almost the only evidence on working-age trends in overall morbidity in high-income countries comes from the US. These studies have generally found *deteriorating* morbidity since the mid-1990s, particularly activities of daily living (ADLs) and physical functioning.[11-14] Other studies have focused on the older working-age population with similar results.[2 15] Again, not all measures show deteriorations, and not all studies come to identical conclusions,[16] but there is little sign of any improvement in morbidity among working-age Americans. Outside of the US, there is a paucity of evidence, but from the limited evidence that exists, there is again little sign of improving morbidity.[17-19]

For peer review only - http://bmjopen.bmj.com/site/about/guidelines.xhtml

In this paper we provide new evidence on trends in morbidity in England over two decades, using 39 measures from the Health Survey for England (HSE), a high quality Government survey with a combined sample of 140,000 individuals. We make two contributions. Firstly, we provide one of the few systematic trend analyses of working-age morbidity in any high-income country outside the US. Secondly, we supplement self-report measures with 10 'biomarkers', which provide further insight into whether reported changes are simply reporting changes, but which have rarely been examined alongside self-reported working-age morbidity trends (Martin et al. 2010[20] being an exception).

DATA AND METHODS

Data source

This section follows the STROBE cross sectional reporting guidelines.[31] We use the HSE, an annual government-sponsored cross-sectional survey of 3,000-11,000 adults with no proxy responses.[21] A particular advantage is that the initial interview is followed by a nurse visit, which in selected years has also included a blood sample. Nevertheless, analysing change in HSE is more complex than it might appear:

- Firstly, HSE was run by the Government Office of Population Censuses and Surveys in 1991-3, before changing to NatCen in 1994. We focus on 1994-2014 data given evidence of a discontinuity at this point across multiple variables.

For peer review only - http://bmjopen.bmj.com/site/about/guidelines.xhtml

- Secondly, the coverage of HSE varies year-to-year, and moreover there are multiple changes in question wording/filtering. Based on a systematic search of HSE questions, we have included every morbidity measure that is comparable over a significant duration. Even for measures that have been previously been analysed (e.g. BMI[22]), this new analysis uncovered further discontinuities (Web Appendix 1 & Web Appendix 2).
- Third, HSE excludes those in communal establishments. While a smaller problem for the working-age population than older ages,[2] we minimise the impact of rising university attendance by focussing on those aged 25+ (Web Appendix 3). The upper limit of the working-age population is set to 59 (women) and 64 (men) to match state pension ages at the start of the period.
- Fourth, HSE supplies non-response weights from 2003. However, there had been a substantial decline in response rates prior to the introduction of weights, particularly for blood samples (from 53.3% 1994 to 39.9% 2003; Web Appendix 3). We therefore reduce non-response biases by creating new non-response weights, as described in Web Appendix 3.

The resulting sample sizes for the various stages of data collection are shown in Web Appendix 3. Our dataset substantially extends an existing HSE time-series dataset (UK Data Archive SN7025); the code enabling other researchers to assemble this extended time-series dataset are freely available from [authors' website].

For peer review only - http://bmjopen.bmj.com/site/about/guidelines.xhtml

Patient involvement

As this is a health monitoring (rather than intervention) study using all available secondary data, patients were not directly involved. However, from previous discussions we are aware that the study will be of interest to patient/disability advocacy groups, who will receive jargon-free summaries of the research.

Measures

We do not focus on general health/participation restriction measures, as there are numerous non-health factors that influence how they are reported, including *inter alia* social security incentives,[23] gendered- and age-related expectations, and medicalisation.[24] Trends in such measures can therefore differ wildly between surveys covering nominally the same concept and population, e.g. for disability in England[25] or self-rated health in the US.[26] However, the measures clearly do capture something meaningful;[27] interested readers can find trends in seven such measures in Web Appendix 4.

Our systematic search found 39 morbidity measures that are comparable over time: these are summarised in Table 1, with further details in Web Appendix 1 and Web Appendix 2. The measures form one of five types:

1. *Diagnosed chronic diseases*. It has been argued that chronic disease diagnoses are not always a good basis for morbidity comparisons[28] – they partly reflect healthcare systems, changing medicalisation etc. – but there have real consequences via increasing awareness/labelling of morbidity.

For peer review only - http://bmjopen.bmj.com/site/about/guidelines.xhtml

2. *Longstanding illnesses* (LSIs): those reporting a longstanding illness were asked, *'what is the matter with you?'*; up to 6 responses were then coded by the interviewer based on the International Classification of Diseases. *A priori*, these are likely to show similar trends to self-reports of diagnosed chronic disease, and are similarly valuable when contrasted to symptom- or limitation-based measures or biomarkers.
3. *Symptoms*, either single items (e.g. pain, anxiety/depression) or validated symptom scales (e.g. the Rose angina scale, GHQ psychiatric morbidity). While there is no guarantee that a given question will be interpreted identically over time, we would expect symptom-based measures to be more comparable over time than measures based on medical labels.
4. *Activity limitations*, which are perhaps most likely to be comparable over time/place (as argued by a UN agency, the Washington Group on Disability Statistics[29]), although few measures are available (see Conclusions).
5. *Biomarkers* largely avoid differences in reporting over socioeconomic groups or time,[30] but at the price of a partial, indirect and sometimes still-debated relationship to morbidity (see Web Appendix 5).

A further 29 measures are also included in Web Appendix 6 – these include alternate versions of measures used in the main text (including 8 sub-components of summary measures, and 16 reports of ever having a condition even if this not recent), plus 5 other categories of LSI.

For peer review only - http://bmjopen.bmj.com/site/about/guidelines.xhtml

Table 1: HSE morbidity measures

Category	Measure	Type[a]	Operationalisation (years available)
Cardio-vascular disease (CVD)	High blood pressure LSI[b]	L	Hypertension reported as longstanding illness (LSI) (1994-2011)
	Recent high blood pressure	L	Still has (or on medication for) doctor-diagnosed hypertension (1994-2013)
	Biomarker high blood pressure	B	Systolic BP >=140mmHg & diastolic BP >=90mmHg (1994-2013)
	High total cholesterol	B	Total cholesterol >= 5mmol/L (1994-2012)
	Low HDL cholesterol	B	High density lipoprotein (HDL) cholesterol <=1 mmol/L (1998-2013)
	Recent heart attack /stroke	L	Doctor-diagnosed heart attack or stroke in past 12mths (1994-2011)
	Recent angina	L	Doctor-diagnosed angina in past 12mths (1994-2011)
	Ischaemic heart/stroke LSI[b]	L	Stroke, heart attack or angina reported as longstanding illness (LSI) (1994-2011)
	Heart attack symptoms	S	Ever had severe pain across chest for ½hr (1994-2011)
	Mini stroke (TIA) symptoms	S	Attack of weakness/slurred speech/blurred vision in past 12mths (2003-11)
	Angina symptoms	S	Rose Angina scale definition of angina symptoms (1994-2011)
	Any recent CVD	L	Doctor-diagnosed heart condition (exc. hypertension) in past 12mths (1994-2011)
	Any CVD LSI	L	Any CVD reported as longstanding illness (LSI) (1994-2011)
Respiratory	COPD symptoms	S	Regular cough & phlegm for at least 3mths each year (1995-2010)
	Lifetime diagnosed asthma	L	Ever had doctor-diagnosed asthma (1995-2010)
	Asthma LSI[b]	L	Asthma reported as longstanding illness (LSI) (1994-2011)
	Breathlessness-grade 2	S	Short of breath when hurrying up walking uphill (1995-2010)
	Breathlessness-grade 3	S	Short of breath when walking on level ground (1995-2010)
	Recent wheezing/asthma	S	Wheezing, whistling in chest or asthma attack in past 12mths (1995-2010)
	Wheezing stopping sleep	S	Woken 1+ times/wk by wheezing/whistling in chest in last 12mths (1994-2010)
Obesity & diabetes	BMI-underweight	B	Body Mass Index (BMI) <=18.5kg/m^2 (1994-2013)
	BMI-obese	B	Body Mass Index (BMI) >= 30kg/m^2 (1994-2013)
	High waist-hip ratio	B	Waist-hip ratio of >1 for men and >0.85 for women (1994-2013)
	Recent diabetes	L	Currently taking medication for doctor-diagnosed diabetes (1994-2013)
	Diabetes LSI[b]	L	Diabetes reported as longstanding illness (LSI) (1994-2011)
	High glycated haemoglobin	B	HbA_{1C} >=48mmol/mol (2003-2013)
Mental	Mental health LSI[b]	L	Mental health reported as longstanding illness (LSI) (1994-2011)

For peer review only - http://bmjopen.bmj.com/site/about/guidelines.xhtml

Category	Measure	Type[a]	Operationalisation (years available)
Health	Psychiatric morbidity (GHQ)	S	4+ negative symptoms from 12-item General Health Questionnaire (1994-2014)
	Anxiety/depression-moderately	S	At least moderately anxious/depressed today (1996-2014)
	Anxiety/depression-extremely	S	Extremely anxious/depressed today (1996-2014)
Activity limitations & musculo-skeletal	Problems walking today	S	Has at least some problems walking about today (1996-2014)
	Locomotor limitation	S	Can't walk far / bend down / go up or down stairs without resting (1996-2001)
	Problems washing/dressing today	S	Has at least some problems washing/dressing today (1996-2014)
	Self-care limitation	S	Difficulty with one of six everyday activities (e.g. feeding, dressing) (1995-2001)
	Pain-any	S	Has at least some pain or discomfort today (1996-2014)
	Pain-extreme	S	Has extreme pain or discomfort today (1996-2014)
	Arthritis LSI[b]	L	Arthritis or rheumatism reported as longstanding illness (LSI) (1994-2011)
	Other musculoskeletal LSI[b]	L	Other musculoskeletal condition reported as longstanding illness (LSI) (1994-2011)
Sensory & Communication	LSI Eye or Ear	L	Eye or ear condition reported as longstanding illness (LSI) (1994-2011)
	Hearing limitation	S	Cannot follow TV programme at volume others find acceptable (1995-2001)
	Seeing limitation	S	Cannot see well enough to recognise friend across the road (1995-2001)
	Communicating limitation	S	Have problem communicating with other people (1995-2001)
Other Biomarkers	Raised C-reactive protein	B	CRP >3mg/L (1998-2009)
	Raised fibrinogen	B	Fibrinogen >4mg/L (1998-2009)
	Anaemia	B	Haemoglobin <13g/dL for men and <12g/dL for women (1994-2009)
	Iron deficiency	B	Serum ferritin < 45ng/ml (1994-2009)

See Web Appendix 5 for full details on all measures .[a] Measure type key: L=medical label; S=symptom-based; B=biomarker. [b] Particular causes of longstanding illness (LSI) come from the open question, 'what is the matter with you?' Up to 6 responses are then coded by the interviewer into a coding frame based on ICD.

For peer review only - http://bmjopen.bmj.com/site/about/guidelines.xhtml

ANALYSIS

We look both at unadjusted trends (reflecting levels of morbidity in the population) and trends after adjustment for sex and age, following others.[32 33] Individual survey years are grouped into 3-4 year periods to increase sample size and precision (single-year prevalence is given in Web Appendix 7). Because the start/end of trends varies across measures, the easiest-to-interpret measure is the percentage point change across the entire period available (sex/age-adjusted models show average marginal effects following a logistic regression).

To avoid a binary cut-off of statistical significance,[34] 95% confidence intervals are used to convey the precision of each estimated trend. All analyses use weights, exclude boost samples that use different sampling methods, and adjust for the clustered nature of the main sample (although standard errors will be slightly underestimated as it is not possible to consistently adjust for sample stratification). For reasons of space, we are unable to discuss previous HSE studies of aspects of morbidity in the main text; these are instead described in Web Appendix 8.

RESULTS

Conditions with sharply declining mortality

We start by focussing on cardiovascular disease (CVD) and respiratory illness, which have both seen sharp falls in mortality (by >50% and >25% respectively among 0-64

For peer review only - http://bmjopen.bmj.com/site/about/guidelines.xhtml

year-olds 1994-2013; Web Appendix A9). Trends in *morbidity*, however, are shown in Table 2.

For peer review only

For peer review only - http://bmjopen.bmj.com/site/about/guidelines.xhtml

Table 2: Trends in cardiovascular and respiratory morbidity

	Start of trend		Trend			
	Period	Incidence	End period	Raw	Adj.[a]	Adj. 95% CI
**Blood pressure/cholesterol**						
High blood pressure LSI	1994-96	2.7%	2011-14	1.3%	1.0%	[0.4, 1.6%]
Recent high blood pressure	1994-96	4.2%	2011-14	5.2%	4.8%	[3.9, 5.6%]
Biomarker high BP	1994-96	8.4%	2011-14	-4.7%	-5.0%	[-5.6, -4.5%]
High total cholesterol	1994-96	75.7%	2011-14	-16.4%	-17.6%	[-19.1, -16.1%]
Low HDL cholesterol	1997-2000	11.8%	2011-14	-8.0%	-8.0%	[-9.0, -7.1%]
**Other CVD**						
Recent heart attack/stroke	1994-96	1.2%	2011-14	-0.3%	-0.4%	[-0.7, 0.0%]
Recent angina	1994-96	1.1%	2011-14	-0.4%	-0.5%	[-0.8, -0.1%]
IHD/stroke LSI	1994-96	1.4%	2011-14	-0.4%	-0.6%	[-0.9, -0.2%]
Heart attack symptoms	1994-96	5.5%	2011-14	-0.3%	-0.5%	[-1.3, 0.3%]
Mini stroke (TIA) symptoms	2001-03	8.1%	2011-14	-1.4%	-1.4%	[-2.4, -0.4%]
Angina symptoms	1994-96	2.3%	2011-14	-1.1%	-1.2%	[-1.6, -0.7%]
Any CVD LSI	1994-96	5.8%	2011-14	1.1%	0.6%	[-0.1, 1.4%]
Any recent CVD	1994-96	3.1%	2011-14	0.7%	0.5%	[-0.1, 1.2%]
**Respiratory**						
Lifetime diagnosed asthma	1994-96	11.2%	2008-10	5.5%	5.7%	[4.5, 6.8%]
Asthma LSI	1994-96	5.0%	2011-14	0.7%	0.7%	[0.0, 1.4%]
Breathlessness-Grade 2+	1994-96	19.7%	2008-10	-4.4%	-4.8%	[-6.1, -3.5%]
Breathlessness-Grade 3	1994-96	7.8%	2008-10	-1.4%	-1.6%	[-2.5, -0.8%]
Recent wheezing/asthma	1994-96	19.5%	2008-10	-1.2%	-1.2%	[-2.5, 0.1%]
Wheezing stopping sleep	1994-96	3.6%	2008-10	-0.4%	-0.5%	[-1.0, 0.1%]
COPD symptoms	1994-96	6.6%	2008-10	-1.5%	-1.6%	[-2.3, -0.8%]

[a] 'Adj.' = trend adjusted for changing age and sex distribution of the working-age population.

Looking first at high blood pressure, biomarker-measured high blood pressure has halved over two decades (similar improvements are found for the biomarkers for total and HDL cholesterol). Yet when we look at self-reports (either people reporting this as an LSI, or in response to a direct question about having recent diagnosed high blood pressure), we see sharp *rises* over time. There has been an increasing diagnosis of high blood pressure and increasing prescriptions of blood pressure-lowering drugs; these may have helped reduce the underlying incidence of high blood pressure while simultaneously raising people's awareness of morbidity.

For peer review only - http://bmjopen.bmj.com/site/about/guidelines.xhtml

Table 2 further shows declines in several key types of CVD (heart attack, mini-stroke, angina), whether measured through people's reports of the disease itself or their reports of its symptoms. Nevertheless, the morbidity declines (8-50%) are often not on the scale of the declines in mortality (>50%); this is likely to be because mortality declines are partly driven by improved treatment,[35] which means each incident CVD case is likely to last longer.[36 37] More surprisingly, the measures of 'any reported CVD' show no improvement (with some, uncertain signs of *rises*). Looking at its sub-components (Web Appendix 6), this seems to be due to possible increases in diagnosed irregular heart rhythm and other heart trouble.

Finally, Table 2 shows that symptoms-based measures of respiratory morbidity have improved, particularly COPD symptoms (regular cough & phlegm) and breathlessness (at both levels), and more uncertainly for recent wheezing/asthma and wheezing stopping sleep. Again, though, diagnosis-related measures of asthma – reported diagnoses, or self-reports of having asthma as a longstanding illness – have risen, even while underlying symptomology is improving.

Overall, Table 2 illustrates how trends in morbidity do not necessarily follow trends in mortality. There are definite improvements in CVD risk factors and respiratory symptomology on the scale of improvements in mortality. But the prevalence of self-reported CVD conditions such as heart attacks have only declined by a smaller amount, and recent doctor-diagnosed hypertension, any CVD, and asthma diagnoses have either stayed stable or risen.

For peer review only - http://bmjopen.bmj.com/site/about/guidelines.xhtml

For peer review only

Conditions with claims of increasing prevalence

The previous section focussed on conditions where there may be an *a priori* expectation that morbidity has improved (given declining mortality); in this section, we focus on three areas where there have been widespread claims of increasing prevalence – obesity, diabetes, and mental health.

Looking at Table 3, we do indeed confirm a considerable rise in obesity in HSE (an 8.0-9.7% rise from an obesity prevalence of 16.9% in 1994-96). The rise in high waist-hip ratios – sometimes suggested to be a better measure of potential morbidity [38] – is if anything even sharper. This has come alongside little change in the prevalence of being *underweight* over this period.

Table 3: Trends in obesity, diabetes and mental health

	Start of trend		Trend			
	Period	Incidence	End period	Raw	Adj.[a]	Adj. 95% CI
**Underweight/Obesity**						
BMI-Underweight	1994-96	1.0%	2011-14	-0.1%	-0.1%	[-0.3, 0.1%]
BMI-Obese	1994-96	16.9%	2011-14	9.3%	8.9%	[8.0, 9.7%]
High waist-hip ratio	1994-96	9.5%	2011-14	14.8%	14.1%	[13.0, 15.2%]
**Diabetes**						
Recent diabetes	1994-96	1.2%	2011-14	2.4%	2.2%	[1.9, 2.6%]
Diabetes LSI	1994-96	1.5%	2011-14	2.3%	2.1%	[1.5, 2.6%]
Glycated haemoglobin	2001-03	2.7%	2011-14	2.1%	2.1%	[1.4, 2.7%]
**Mental health**						
Mental health LSI	1994-96	2.1%	2011-14	2.5%	2.4%	[1.8, 3.0%]
Psychiatric morbidity	1994-96	17.1%	2011-14	-1.3%	-1.3%	[-2.4, -0.3%]
Anx./depression-moderate	1994-96	21.9%	2011-14	0.3%	0.1%	[-1.1, 1.3%]
Anx./depression-extremely	1994-96	1.8%	2011-14	1.0%	0.9%	[0.5, 1.3%]

[a] 'Adj.' = trend adjusted for changing age and sex distribution of the working-age population.
'Anx./depression'= Anxiety/depression – see Table 1.

For peer review only - http://bmjopen.bmj.com/site/about/guidelines.xhtml

Table 3 also confirms a sharp rise in diabetes. This can be seen whether diabetes is measured through people reporting diabetes as an LSI, a specific question about people currently taking medication for diabetes, or via a diabetes biomarker (glycated haemoglobin). It is worth noting that this clear rise in diabetes has occurred despite a *decline* in the age 0-64 death rate from diabetes, by more than one-third 1994-2013 (Web Appendix 3) – indeed, rising prevalence is *because of* falling mortality [39] – again demonstrating the difference between mortality and morbidity trends.

Trends in mental health are more contentious in the wider literature (see Web Appendix 8), and the measures in HSE are not as strong as in the more occasional Adult Psychiatric Morbidity Surveys. Nevertheless, HSE offers a unique annual perspective on mental health trends. As we might expect from increasing treatment/diagnosis, we see a doubling in people reporting a mental health LSI. However, the symptoms-based measures show a more mixed picture:

- Neither of the measures that capture more moderate mental ill-health show rising ill-health (these are 'psychiatric morbidity symptoms' and 'moderate anxiety/depression today', both with a relatively common prevalence of 15-25%). If we break this down by year (see Web Appendix 2 and Web Appendix 8), we can see moderate mental ill-health fell between the mid-1990s and the mid-2000s, before rising in 2009.

For peer review only - http://bmjopen.bmj.com/site/about/guidelines.xhtml

- In contrast, the single measure capturing 'extreme anxiety/depression today' does show rising morbidity. To see if there were similar signs of rising mental ill-health at extremes in our other measure – the psychiatric morbidity scores (obtained from the 12-item GHQ scale) – we created a further measure based on a much higher GHQ threshold of 10 negative responses out of the 12 GHQ questions (compared to the conventional GHQ threshold of 4). Unlike the conventional GHQ measure, this also showed an increase over time (95% CI of a 0.4 to 1.4% rise; see Web Appendix 6). We should note however that the GHQ is not designed to capture severe anxiety/depression in this way.

Overall, while labelling of mental health conditions has undoubtedly risen, trends in mental health vary across measures. If we interpret higher GHQ thresholds as indicating more serious depression/anxiety, then we can see a consistent picture: moderate mental ill-health rose from the mid-1990s to the mid-2000s before falling, whereas more extreme mental ill-health has risen.

Activity limitations, musculoskeletal and pain

Pain/musculoskeletal conditions are a major component of working-age morbidity, yet very few previous studies show trends in symptomology, and even those that exist [40] sometimes have debatable comparability.[41] Table 4 shows a fall in some – but not all – HSE measures focussed on pain and musculoskeletal morbidity. Arthritis as a longstanding illness (LSI) has declined (the precision of the estimates is greater when looking at 2008-10 rather than 2011-14, and shows a decline of 0.3-1.2%).

For peer review only - http://bmjopen.bmj.com/site/about/guidelines.xhtml

There are some (similarly uncertain) signs that other musculoskeletal LSIs have also fallen, and noticeably fewer people say that they have any pain/discomfort today, although there has been no change in people saying they have extreme pain/discomfort. The echoes the limited wider evidence of rises in back pain over an earlier period[40 42].

Similarly, there has been a rise in all four activity limitations measures in HSE – although the increases are sometimes uncertain, and are smaller after adjusting for changes in age/sex structure. Moreover, the timing of the rises differ between the measures: the trend in limitations lasting at least a year shows a rise 1994-6 to 2001-3, but the two measures of 'limitations today' do not, instead showing a possible slight rise in the more recent period (see Web Appendix 7; this difference remains if we focus on the sub-components of year-long limitations that more closely match to the 'limitations today' questions, see Web Appendix 6). Still, the measures can collectively be seen as offering some, albeit relatively weak, evidence for an increase in activity limitations.

For peer review only

Table 4: Trends in activity limitations, pain & musculoskeletal morbidity

	**Start of trend**		**Trend**			
	Period	Incidence	End period	Raw	Adj.[a]	Adj. 95% CI
**Activity limitations**						
Problems walking about	1994-96	11.5%	2011-14	1.0%	0.4%	[-0.6, 1.3%]
Any locomotor limitation	1994-96	6.8%	2001-03	1.1%	0.9%	[0.1, 1.7%]
Probs. washing/dressing	1994-96	3.4%	2011-14	0.6%	0.3%	[-0.2, 0.9%]
Any self-care limitation	1994-96	3.9%	2001-03	0.8%	0.7%	[0.1, 1.3%]
**Musculoskeletal/pain**						
Pain-any	1994-96	32.0%	2011-14	-2.2%	-3.3%	[-4.6, -2.0%]
Pain-extreme	1994-96	3.0%	2011-14	0.4%	0.2%	[-0.3, 0.7%]
Arthritis LSI	1994-96	5.3%	2011-14	-0.3%	-0.7%	[-1.4, 0.0%]

For peer review only - http://bmjopen.bmj.com/site/about/guidelines.xhtml

Other musculoskeletal LSI	1994-96	9.7%	2011-14	-0.5%	-0.8%	[-1.7, 0.1%]

[a] 'Adj.' = trend adjusted for changing age and sex distribution of the working-age population.

Other measures

Trends in other measures (for which we have no clear *a priori* expectations of trends) are shown in Table 5 below. This includes four biomarkers that are more difficult to compare directly to self-reports:

- Trends are available for two biomarkers of inflammation (C-reactive protein ('CRP') and fibrinogen), which are commonly used as measures of heart disease risk. However, their interpretation is difficult as they are also associated with other conditions such as diabetes, cancer [43] and – in the case of CRP – even depression.[44] Table 5 shows that both biomarkers have rising morbidity from 1997-2000 to 2008-10 (although for CRP, the confidence interval is wide and there is a non-negligible possibility that the trend is negative).
- The two other biomarkers available in HSE are clearly focussed on anaemia and iron deficiency. Table 5 shows that both of these have declined, with particularly clear evidence for a decline in iron deficiency.

For peer review only - http://bmjopen.bmj.com/site/about/guidelines.xhtml

Table 5: Trends in other morbidity measures

	Start of trend		Trend			
	Period	Incidence	End period	Raw	Adj.[a]	Adj. 95% CI
**Other biomarkers**						
Raised C-reactive protein	1997-2000	21.4%	2008-10	2.1%	1.9%	[-0.7, 4.5%]
Raised fibrinogen	1997-2000	2.3%	2008-10	1.6%	1.5%	[0.3, 2.6%]
Anaemia	1994-96	6.7%	2008-10	-1.4%	-1.4%	[-2.7, -0.1%]
Iron deficiency	1994-96	39.9%	2008-10	-12.9%	-12.5%	[-14.8, -10.2%]
**Sensory & communication**						
LSI Eye or Ear	1994-96	2.8%	2011-14	-0.9%	-1.0%	[-1.5, -0.6%]
Hearing limitation	1994-96	4.3%	2001-03	-1.5%	-1.6%	[-2.1, -1.0%]
Seeing limitation	1994-96	1.4%	2001-03	-0.2%	-0.2%	[-0.6, 0.1%]
Communicating limitation	1994-96	1.0%	2001-03	0.1%	0.1%	[-0.2, 0.4%]

[a] 'Adj.' = trend adjusted for changing age and sex distribution of the working-age population.

Table 5 also shows trends in sensory and communication-related morbidity. This shows a fall in eye/ear conditions (1994-6 to 2011-14) as well as hearing limitations in the earlier period (1994-6 to 2001-03), but no change in people having difficulty communicating with others.

DISCUSSION

Despite considerable evidence on morbidity trends among older people, there are few published studies on morbidity trends among the working-age population, particularly outside the USA. In this paper, we have analysed trends in working-age morbidity in England 1994-2014 using a high-quality repeated cross-sectional study. We see improvements in cardiovascular morbidity, respiratory morbidity and anaemia, but deteriorating obesity, diabetes, some biomarkers (fibrinogen and possibly also CRP) and mental ill-health at the highest levels. We see little systematic trend in more common mental ill-health or musculoskeletal conditions,

For peer review only - http://bmjopen.bmj.com/site/about/guidelines.xhtml

pain/mobility, and self-care limitations. We should also stress that symptomology and chronic disease diagnoses often go in different directions – chronic disease diagnoses have sometimes stayed stable or even risen at the same time that underlying symptomology has declined (such as for mental health conditions, asthma, hypertension, and CVD as a whole), mirroring findings at older ages.[3]

Our analysis has several strengths. We include every morbidity measure for which consistent trends can be constructed, including chronic disease, functioning and symptomology, and biomarkers. We use a single survey series collected by a single survey organisation; exclude under-25s for whom comparability of survey coverage is unlikely; and construct new non-response weights. Nevertheless, we must note three limitations. Firstly, response rates for each stage of the HSE have declined over time (see Web Appendix 3), and while we create new non-response weights covering the entire period, it is impossible to rule out changing non-response biases. Secondly, it is possible that people respond differently over time even to identical questions. Third, there are several dimensions of morbidity for which there is little trend data in HSE. This includes several areas in which morbidity among the working-age population seems to be rising, including *inter alia* cognitive complaints,[45] allergic disorders,[46] and liver cirrhosis (see Web Appendix 3), as well as some areas in which morbidity seems likely to have fallen, such as chronic kidney disease.[47]

For policymakers, this leaves the question of whether working-age morbidity as a whole is getting better or worse in England (at least for those who believe that health

For peer review only - http://bmjopen.bmj.com/site/about/guidelines.xhtml

For peer review only

states can be put on a unidimensional scale). While it is not possible to create a single morbidity index here, Web Appendix 2 shows the association of each measure with bad general self-rated health (net of age, gender and education). This shows little systematic trend for falling morbidity to be seen in the measures that predict health the most (indeed, the evidence weakly points in the other direction, towards rising morbidity). Certainly there is no evidence that working-age morbidity as a whole has declined over the past twenty years in England despite falling mortality. This mirrors both evidence from the Global Burden of Disease study for the UK (see Web Appendix 2), and more detailed analyses available for the US.[11-14]

In conclusion, despite considerable falls in working-age mortality and gains in life expectancy – and the ensuing expectations of social security policymakers for improving morbidity – there is no systematic improvement in overall working-age morbidity in England from 1994 to 2014. However, two pieces of further research could strengthen this evidence base. Firstly, the ideal measures for analysing trends in morbidity are functional limitations measures, which are included in the HSE from 1996. However, these were last asked to the working-age population in 2001, and it is a priority to repeat these measures in future years of HSE. Secondly, there is a surprising paucity of studies looking at the changing morbidity of the working-age population outside the US. Given their importance in public debate – particularly in discussions of retirement ages and disability benefits – we hope that other authors

For peer review only - http://bmjopen.bmj.com/site/about/guidelines.xhtml

will repeat and extend our analyses here, including disaggregating these trends across different regions and sociodemographic groups.

For peer review only

For peer review only - http://bmjopen.bmj.com/site/about/guidelines.xhtml

REFERENCES

1. Freedman VA, Crimmins E, Schoeni RF, et al. Resolving inconsistencies in trends in old-age disability: report from a technical working group. *Demography* 2004;41(3):417-41.
2. Freedman VA, Spillman BC, Andreski PM, et al. Trends in Late-Life Activity Limitations in the United States: An Update From Five National Surveys. *Demography* 2013;50(2):661-71. doi: 10.1007/s13524-012-0167-z
3. Freedman VA, Schoeni RF, Martin LG, et al. Chronic Conditions and the Decline in Late-Life Disability. *Demography* 2007;44(3):459-77.
4. Lafortune G, Balestat G. Trends in severe disability among elderly people. Paris: OECD, 2007.
5. Bowling A. Commentary: Trends in activity limitation. *International Journal of Epidemiology* 2011;40(4):1068-70. doi: 10.1093/ije/dyr069
6. Jagger C. Trends in life expectancy and healthy life expectancy. Future of an ageing population: evidence review. London: Foresight, Government Office for Science, 2015.
7. Office for Budget Responsibility. Welfare trends report: October 2016. Cm 9341. London: Her Majesty's Stationery Office, 2016.
8. Department for Work and Pensions, Department of Health. Improving Lives: The Work, Health and Disability Green Paper. Cm 9342. London: Her Majesty's Stationery Office, 2016.
9. OECD. Sickness, Disability and Work: Breaking the Barriers, Vol 2: Australia, Luxembourg, Spain and the United Kingdom Paris: OECD, 2008.
10. Wise DA, editor. *Social Security Programs and Retirement Around the World: The Capacity to Work at Older Ages*. Chicago: University of Chicago Press for the National Bureau of Economic Research (NBER), 2017.
11. Martin LG, Freedman VA, Schoeni RF, et al. Trends In Disability And Related Chronic Conditions Among People Ages Fifty To Sixty-Four. *Health Affairs* 2010;29(4):725-31. doi: 10.1377/hlthaff.2008.0746
12. Bhattacharya J, Choudhry K, Lakdawalla D. Chronic Disease and Severe Disability among Working-Age Populations. *Medical Care* 2008;46(1):92-100.
13. Martin LG, Schoeni RF. Trends in disability and related chronic conditions among the forty-and-over population: 1997-2010. *Disability and Health Journal* 2014;7(1):S4-S14. doi: 10.1016/j.dhjo.2013.06.007
14. Lakdawalla DN, Bhattacharya J, Goldman DP. Are the young becoming more disabled? *Health affairs* 2004;23(1):168-76.
15. Seeman TE, Merkin SS, Crimmins EM, et al. Disability Trends Among Older Americans: National Health and Nutrition Examination Surveys, 1988–1994 and 1999–2004. *American Journal of Public Health* 2010;100(1):100-07. doi: 10.2105/AJPH.2008.157388
16. Weir D. Are baby boomers living well longer. In: Madrian B, Mitchell OS, Soldo BJ, eds. Redefining retirement: How will boomers fare. Oxford: Oxford UP 2007:95-111.
17. van Oostrom SH, Gijsen R, Stirbu I, et al. Time Trends in Prevalence of Chronic Diseases and Multimorbidity Not Only due to Aging: Data from General Practices and Health Surveys. *PLoS ONE* 2016;11(8):e0160264. doi: 10.1371/journal.pone.0160264
18. Solé-Auró A, Alcañiz M. Are we living longer but less healthy? Trends in mortality and morbidity in Catalonia (Spain), 1994–2011. *European Journal of Ageing* 2015;12(1):61-70. doi: 10.1007/s10433-014-0317-9

For peer review only - http://bmjopen.bmj.com/site/about/guidelines.xhtml

19. Audureau E, Rican S, Coste J. Worsening trends and increasing disparities in health-related quality of life: evidence from two French population-based cross-sectional surveys, 1995–2003. *Quality of Life Research* 2013;22(1):13-26. doi: 10.1007/s11136-012-0117-7
20. Martin LG, Schoeni RF, Andreski PM. Trends in health of older adults in the United States: past, present, future. *Demography* 2010;47(Suppl):S17-S40.
21. Mindell J, Biddulph JP, Hirani V, et al. Cohort Profile: The Health Survey for England. *International Journal of Epidemiology* 2012;41(6):1585-93. doi: 10.1093/ije/dyr199
22. Sperrin M, Marshall A, Higgins V, et al. Slowing down of adult body mass index trend increases in England: a latent class analysis of cross-sectional surveys (1992-2010). *International Journal of Obesity* 2014;38(6)
23. O'Brien R. Disability and the Worlds of Welfare Capitalism. *Sociological Science* 2015;2(1):1-19. doi: 10.15195/v2.a1
24. OECD. Sick on the Job? Myths and Realities about Mental Health and Work. Paris: OECD, 2012.
25. Baumberg B, Jones M, Wass V. Disability prevalence and disability-related employment gaps in the UK 1998–2012: Different trends in different surveys? *Social Science & Medicine* 2015;141:72-81. doi: http://dx.doi.org/10.1016/j.socscimed.2015.07.012
26. Salomon JA, Nordhagen S, Oza S, et al. Aer Americans feeling less healthy? The puzzle of trends in self-rated health. *American Journal of Epidemiology* 2009;170(3):343-51.
27. Quesnel Vallee A. Self-rated health: caught in the crossfire of the quest for 'true' health? *International Journal of Epidemiology* 2007;36:1161-64.
28. Cieza A, Oberhauser C, Bickenbach J, et al. The English are healthier than the Americans: really? *International Journal of Epidemiology* 2015:229-38. doi: 10.1093/ije/dyu182
29. Altman BM, editor. *International Measurement of Disability: Purpose, Method and Application*: Springer, 2016.
30. Benzeval M, Kumari M, Jones AM. How Do Biomarkers and Genetics Contribute to Understanding Society? *Health Economics* 2016;25(10):1219-22. doi: 10.1002/hec.3400
31. von Elm E, Altman D, Egger M, et al. The Strengthening the Reporting of Observational Studies in Epidemiology (STROBE) Statement: guidelines for reporting observational studies. *Annals of Internal Medicine* 2007;147:573-77.
32. Martin LG, Freedman VA, Schoeni RF, et al. Health and Functioning Among Baby Boomers Approaching 60. *The Journals of Gerontology Series B: Psychological Sciences and Social Sciences* 2009;64B(3):369-77. doi: 10.1093/geronb/gbn040
33. Kaye HS. Disability Rates For Working-Age Adults And For The Elderly Have Stabilized, But Trends For Each Mean Different Results For Costs. *Health Affairs* 2013;32(1):127-34. doi: 10.1377/hlthaff.2012.0533
34. Wasserstein RL, Lazar NA. The ASA's Statement on p-Values: Context, Process, and Purpose. *The American Statistician* 2016;70(2):129-33. doi: 10.1080/00031305.2016.1154108
35. Unal B, Critchley JA, Capewell S. Explaining the Decline in Coronary Heart Disease Mortality in England and Wales Between 1981 and 2000. *Circulation* 2004;109(9):1101-07. doi: 10.1161/01.cir.0000118498.35499.b2
36. Davies AR, Smeeth L, Grundy E. Contribution of changes in incidence and mortality to trends in the prevalence of coronary heart disease in the UK: 1996–2005. *European Heart Journal* 2007;28(17):2142-47.

For peer review only - http://bmjopen.bmj.com/site/about/guidelines.xhtml

37. Oyebode O. Cardiovascular disease. In: Craig R, Mindell J, eds. Health Survey for England, 2011, Volume 1: Health, social care and lifestyles: TSO 2012:21-62.
38. Davillas A, Benzeval M. Alternative measures to BMI: Exploring income-related inequalities in adiposity in Great Britain. *Social Science & Medicine* 2016;166:223-32. doi: http://dx.doi.org/10.1016/j.socscimed.2016.08.032
39. Read SH, Kerssens JJ, McAllister DA, et al. Trends in type 2 diabetes incidence and mortality in Scotland between 2004 and 2013. *Diabetologia* 2016;59(10):2106-13. doi: 10.1007/s00125-016-4054-9
40. McBeth J, Jones K. Epidemiology of chronic musculoskeletal pain. *Best Practice & Research Clinical Rheumatology* 2007;21(3):403-25. doi: 10.1016/j.berh.2007.03.003
41. Harkness EF, Macfarlane GJ, Silman AJ, et al. Is musculoskeletal pain more common now than 40 years ago?: two population-based cross-sectional studies. *Rheumatology* 2005;44(7):890-95. doi: 10.1093/rheumatology/keh599
42. Palmer KT, Walsh K, Bendall H, et al. Back pain in Britain: comparison of two prevalence surveys at an interval of 10 years. *BMJ* 2000;320:1577-8.
43. Chaudhury M. Blood analytes. In: Sproston K, Primatesta P, eds. Health Survey for England, 2003, Vol 2: Risk Factors for Cardiovascular Disease: TSO 2004:241-88.
44. Steptoe A. Psychosocial biomarker research: integrating social, emotional and economic factors into population studies of aging and health. *Social Cognitive and Affective Neuroscience* 2011;6(2):226-33. doi: 10.1093/scan/nsq032
45. Begum A, Dewey M, Hassiotis A, et al. Subjective cognitive complaints across the adult life span: a 14-year analysis of trends and associations using the 1993, 2000 and 2007 English Psychiatric Morbidity Surveys. *Psychological Medicine* 2014;44(9):1977-87. doi: 10.1017/S0033291713002444
46. Gupta R, Sheikh A, Strachan DP, et al. Time trends in allergic disorders in the UK. *Thorax* 2007;62(1):91-96. doi: 10.1136/thx.2004.038844
47. Aitken GR, Roderick PJ, Fraser S, et al. Change in prevalence of chronic kidney disease in England over time: comparison of nationally representative cross-sectional surveys from 2003 to 2010. *BMJ Open* 2014;4(9) doi: 10.1136/bmjopen-2014-005480

For peer review only

For peer review only - http://bmjopen.bmj.com/site/about/guidelines.xhtml

WEB APPENDICES

Table of contents *(pages refer to within web appendices)*

Appendix 1: Missingness in health measures 2
Appendix 2: Summarising multiple measures 5
Appendix 3: Sample adjustment & non-response weights 9
Appendix 4: General self-reported health/disability 12
Appendix 5: Health measures 13
Appendix 6: Measures not included in the main paper 25
Appendix 7: Year-by-year trends 30
Appendix 8: Others' health trend analyses using HSE data 36
Appendix 9: Working-age mortality trends 39
Bibliography for Web Appendix 40

For peer review only

For peer review only - http://bmjopen.bmj.com/site/about/guidelines.xhtml

Appendix 1: Missingness in health measures

Interview measures

As shown in Appendix 1 above, the response rate for the initial face-to-face interview fell from 71.6% in 1994 to 55.5% in 2014. For those who took part in the initial face-to-face interview, the level of item missingness is shown below (including only those years in which each question was asked). This shows the item-missingness is generally very low – only 1 of the 30 measures variables have item-missingness greater than 1%.

Table 1: Missingness at the initial face-to-face interview

	n *non-missing*	*n* *missing*	*%* *missingness*
BMI	124,682	15,415	11.0%
Any recent CVD	43,274	354	0.8%
Recent high blood pressure	43,366	262	0.6%
Breathlessness-Grade 2+	25,620	68	0.3%
Breathlessness-Grade 3	25,620	68	0.3%
Recent heart attack/stroke	43,519	109	0.3%
COPD symptoms	25,631	57	0.2%
Recent angina	43,551	77	0.2%
Heart attack symptoms	43,595	33	0.1%
Angina symptoms	43,592	36	0.1%
Recent diabetes	66,637	54	0.1%
Mini stroke (TIA) symptoms	23,487	16	0.1%
Diagnosed asthma	41,225	28	0.1%
Wheezing stopping sleep	41,224	29	0.1%
Recent wheezing/asthma	41,224	29	0.1%
Locomotor limitation	25,347	10	0.0%
Self-care limitation	25,347	10	0.0%
Limitations in past 2wks	140,041	56	0.0%
Longstanding illness (LSI)	124,906	43	0.0%
Limiting LSI (LLSI)	104,798	36	0.0%
Any CVD LSI	124,912	37	0.0%
IHD/stroke LSI	124,912	37	0.0%
Mental health LSI	124,912	37	0.0%
Arthritis LSI	124,912	37	0.0%
Asthma LSI	124,912	37	0.0%
Diabetes LSI	124,912	37	0.0%
High blood pressure LSI	124,912	37	0.0%
Other musculoskeletal LSI	124,912	37	0.0%
Good general health	140,048	49	0.0%
Bad general health	140,048	49	0.0%

The only variable with noticeable missingness is BMI, which is understandable as this involves the interviewer taking height and weight measurements rather than simply asking for a verbal response. There are various reasons why people do not have a BMI measurement:

- *High weight*: people with a very high weight are not weighed in HSE 'because the scales are inaccurate above this level', but the definition of this changed (from 130kg before 2011 to

For peer review only - http://bmjopen.bmj.com/site/about/guidelines.xhtml

200kg afterwards). 1.00% of respondents were not weighted for this reason in 2010, which fell to <0.1% 2012-14.

- *Difficult to take measurement*: other respondents have no valid BMI measurement because height or weight measures were not attempted, attempted but not obtained or useable, because the respondent was pregnant, or the respondent was too sick or unsteady. While this varies a little year to year (between 3.8% and 6.1%), there has been little systematic trend in this reason for non-response.
- *Refusal*: the most common reason for no BMI measurement is an outright refusal (including those refusing out of anxiety, though this tends to be a minor reason). In line with the general participation rates at each stage of the interview above, refusal rates rose sharply from 1.9% in 1994 to a peak of 11.5% in 2011, and remain at 8.3% in the 2014 data.

Because of the high level of item non-response for BMI, a non-response weight was created to try to correct for any biases that this introduces. This followed the identical procedure outlined in Appendix 1 for creating non-response weights for the nurse visit, blood sample etc.

Self-completion measures

As shown in Appendix 1 above, the response rate for the self-completion booklet fell from 71.2% in 1994 (almost everyone who took part in the initial interview) to 51.5% in 2014 (93% of those who took part in the initial interview). For those who completed the self-completion booklet, the level of item missingness is shown in the table below.

Table 2: Missingness within the self-completion booklet

	n *non-missing*	*n* *missing*	*%* *missingness*
Psychiatric morbidity symptoms	108,324	2,462	2.2%
Problems washing/dressing today	62,703	1,310	2.1%
Anxiety/depression	62,725	1,288	2.0%
Problems w/activities	62,742	1,271	2.0%
Problems walking about today	62,772	1,241	1.9%
Pain	62,783	1,230	1.9%

Item missingness is relatively low compared to missingness from not completing the self-completion survey, though similarly to wider participation rates at each stage of the survey, item missingness does increase over time (e.g. for psychiatric morbidity symptoms, from 1.8% 1994 to 5.9% 2014).

Nurse visit measures

As shown in Appendix 1 above, the response rate for the nurse visit fell from 63.3% in 1994 to 37.3% in 2014. For those who took part in the nurse visit, the level of item missingness is shown in the table below.

Table 3: Missingness within the nurse visit

	n *non-missing*	*n* *missing*	*%* *missingness*
Biomarker high blood pressure	87,726	15,517	15.0%
High waist-hip ratio	78,637	2,664	3.3%

For peer review only - http://bmjopen.bmj.com/site/about/guidelines.xhtml

This shows that far more people have missing observations for measured high blood pressure than for their waist-hip ratio. This is despite the fact that we explicitly INCLUDE those who are on blood pressure-lowering drugs (about 5% of the sample at the start of the period and 10% at the end), on the grounds that their lowered blood pressure still conveys useful information about their health state. The main reason for the remaining high level of missingness is because people have recently exercised, smoked, drank or ate (12.2%), which rose noticeably over time (from 6.1% to 13.6%).

Because of the high level of item non-response for high blood pressure (and the moderate level for waist-hip ratio), non-response weights were created to try to correct for any biases that are introduced. This followed the identical procedure outlined in Appendix 1 for creating non-response weights for the nurse visit, blood sample etc.

Blood sample measures

As shown in Appendix 1 above, the response rate for the blood sample fell from 53.3% in 1994 to 28.7% in 2014 (primarily due to higher refusal rates, though also affected by changes in eligibility; see discussion in Appendix 1). For those from whom a blood sample was taken, the level of item missingness is shown in the table below.

Table 4: Missingness within the blood sample

	n non-missing	*n missing*	*% missingness*
Raised fibrinogen	16,166	3,341	17.1%
Raised C-reactive protein	17,814	1,693	8.7%
Glycated haemoglobin	28,810	1,436	4.8%
Anaemia	20,302	939	4.4%
Iron deficiency	20,375	866	4.1%
Low HDL cholesterol	36,076	1,406	3.8%
High total cholesterol	43,409	1,472	3.3%

All of these measures are affected by problems in transferring and storing the blood sample and with the measurement process, which results in problems with 3-10% of the blood samples depending on the measure and year. As for blood pressure, we explicitly INCLUDE those who are on lipid-lowering drugs (0.4% 1994 to 7.9% 2014), on the grounds that their changed cholesterol level still conveys useful information about their health state. Item missingness is highest for fibrinogen, which not only has high rates of such failures (7.0-9.5%), but also has ineligibility due to likely infection (from raised CRP, 3.6-5.6% of those with blood samples) and taking drugs that affect the reading (3.7% 1994 to 7.7% 2009). Item missingness is also high for C-reactive protein (CRP), which also excludes those with likely infections.

Because of the high level of item non-response for fibrinogen and CRP (and the moderate level for other blood sample biomarkers), non-response weights were created to try to correct for any biases that are introduced. This followed the identical procedure outlined in Appendix 1 for creating non-response weights for the nurse visit, blood sample etc.

For peer review only - http://bmjopen.bmj.com/site/about/guidelines.xhtml

Appendix 2: Summarising multiple measures

Having reviewed trends in 39 morbidity measures, we have seen that morbidity in the English working-age population has improved in some respects and deteriorated in others. For those who view work-related morbidity as intrinsically multidimensional,[42], this is the endpoint of our analysis. However, for those who conceive of morbidity as unidimensional – or those who are interested in morbidity as it relates to a unidimensional work capacity – this raises the question of how we weight different dimensions of morbidity to decide if the overall change in morbidity has been positive or negative.

Methods for creating unidimensional morbidity scales

Several methods have been proposed for creating unidimensional morbidity scales, but most of these are unavailable using the HSE data:

- Weights can be based on empirically-derived preferences for different health states, of which the most famous example is the WHO Global Burden of Disease (GBD) study [43]. Some GBD estimates for trends in disability in the UK do exist, and suggest that the prevalence of disability in the working-age population is unchanged 1990-2010, though these results are only presented in passing.[1] For our analyses, however, we have no preference-based weights for most of the HSE measures (excluding the subset of measures that make up the EQ-5D scale).
- Those reporting limitations beyond a certain severity in any domain can be categorised as 'disabled', as recommended by the Washington Group on Disability Statistics (see above). However, as previously discussed, we have few functional limitations measures available in HSE.
- Latent morbidity scales can be created based on the inter-correlations between different measures (using item response theory), as used in the World Disability Report [46] and by researchers associated with the US National Bureau of Economic Research e.g. [47]. However, it is unclear why we would wish to weight items in this way: a given morbidity indicator may be severe, yet if it is unrelated to other morbidity measures it will be given a low weight.
- Latent morbidity scales can also be created based on the independent correlation between each indicator and a general measure of morbidity, such as general self-reported health or [48] as in [49]. This maintains some of the advantages of single-item measures (in providing a basis for making morbidity unidimensional), while avoiding the potential threats to validity discussed above. However, the inconsistent inclusion of measures in each HSE wave prevents a unidimensional morbidity scale being constructed here.

---

[1] Trends in the UK GBD results are reported in Murray et al.[44]. However, Murray et al do not focus on trends in years lived with disability (YLD), other than to note that *"YLDs per person by age and sex have not changed substantially in the UK, but age-specific mortality has been improving"* (p1005). The figure in the supplementary appendix shows that YLDs have barely changed for either men or women at any age. However, the confidence intervals for YLDs as a whole in the main paper (Table 1) suggest that the confidence intervals for these trends are very wide. The public GBD data [45] do provide cause-disaggregated YLDs for the UK (and all other countries) for a slightly different period (2000-2015), but are not age-standardised, are within broad age groups only (e.g. 15-29), and again lack estimates of uncertainty.

For peer review only - http://bmjopen.bmj.com/site/about/guidelines.xhtml

An alternative way of summarising heterogeneous trends

Nevertheless, we can examine if the areas in which morbidity has been improving or declining are those that are particularly important for general health.[48] (This uses the same intuition as the scales in Diederichs et al 2012).[49] The resulting inter-relationship of morbidity trends with their effect on general health is shown in Figure 1 below.

Figure 1: Trends in morbidity measures & their association with bad general health[a]

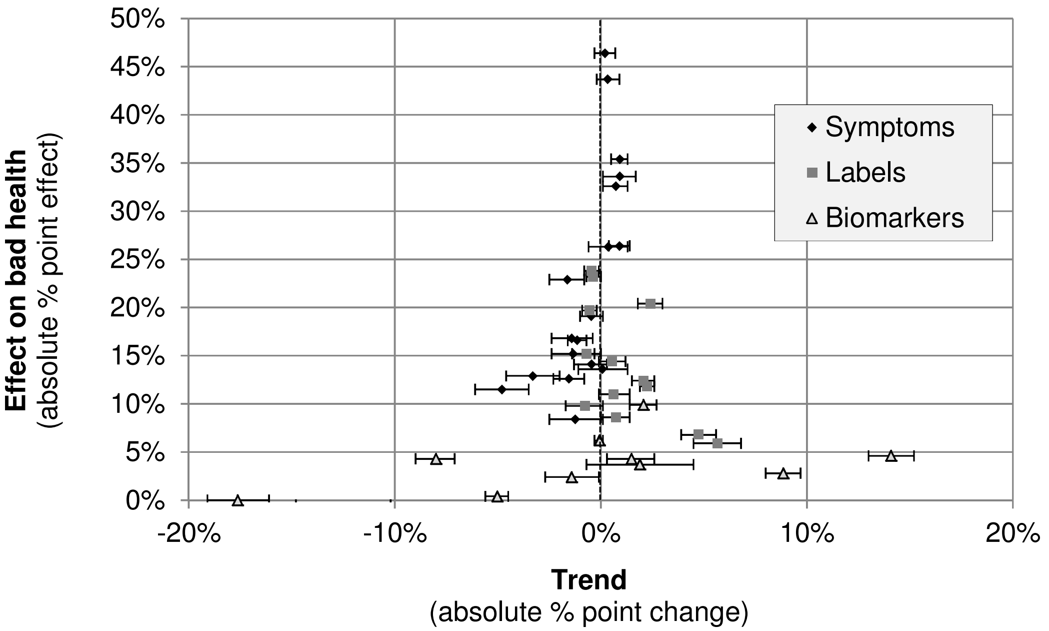

[a] 'Trend' is as reported above. 'Effect on bad health' shows the effect of the morbidity measure on (very) bad health after controlling for age, sex (and their interaction) and educational level, using all years for which the individual morbidity measure is available. (This shows average marginal effects following a logistic regression, with the same survey weights as in the main analyses above). Full details (and 95% CIs for the association with morbidity) are shown below.

It is easiest to interpret the figure by focussing on each group of measures in turn. Firstly, the biomarkers tend to have the weakest relationship with general health. Those with high levels of the diabetes biomarker (glycated haemoglobin) are 9.7% more likely to say they have bad health, and those who are underweight, with a high waist-hip ratio, raised fibrinogen, or low HDL cholesterol are 4-6% more likely to report bad health, but the other measures only had weaker relationships. Indeed, there was effectively no relationship between bad reported health and any of measured high blood pressure, high total cholesterol or iron deficiency.

Secondly, most of the measures based on medical labels have a moderately strong relationship with bad health (the weakest being lifetime asthma and recent high blood pressure, both of which can be asymptomatic), and these measures have mostly risen over time. There are however notable exceptions to this, including IHD/stroke LSI, recent angina and recent heart attack/stroke (the label-based measures with some of the strongest relationships with bad reported health), as well as arthritis and other musculoskeletal LSIs.

Finally, symptom-based measures unsurprisingly tend to have stronger relationships with bad reported health, although this ranges from the moderate (those reporting 'recent wheezing/asthma

For peer review only - http://bmjopen.bmj.com/site/about/guidelines.xhtml

attack' were 8.5% more likely to report bad health) to the very strong (those reporting 'extreme pain today' were 46.4% more likely to report bad health). In general, those symptoms-based measures with the strongest relationship with bad reported health were more likely to have increased over time ('extreme anxiety/depression today', 'locomotor limitations', and 'self-care limitations'). However, the size of the aforementioned declines in symptom-based measures of respiratory and cardiovascular morbidity was often greater.

The corresponding table (also showing the confidence intervals for the association of the measure with bad general health) is shown overleaf, ordered by the effect on bad health (which corresponds to top-to-bottom in Figure 1).

For peer review only

For peer review only - http://bmjopen.bmj.com/site/about/guidelines.xhtml

Measure	Type	Trend in measure	(95% CI)	Effect on bad health	(95% CI)
Pain-extreme	S	0.2%	[-0.3, 0.7%]	46.4%	[44.0, 48.9%]
Problems washing/dressing today	S	0.3%	[-0.2, 0.9%]	43.7%	[41.4, 46.0%]
Anxiety/depression-extremely	S	0.9%	[0.5, 1.3%]	35.4%	[32.8, 38.0%]
Any locomotor limitation	S	0.9%	[0.1, 1.7%]	33.6%	[31.2, 36.0%]
Any self-care limitation	S	0.7%	[0.1, 1.3%]	32.6%	[29.7, 35.5%]
Problems walking about today	S	0.4%	[-0.6, 1.3%]	26.3%	[25.2, 27.4%]
High psychiatric morbidity	S	0.9%	[0.4, 1.4%]	26.4%	[24.9, 27.9%]
Recent angina	L	-0.5%	[-0.8, -0.1%]	23.8%	[20.1, 27.5%]
Recent heart attack/stroke	L	-0.4%	[-0.7, 0.0%]	23.2%	[19.7, 26.7%]
Breathlessness-Grade 3	S	-1.6%	[-2.5, -0.8%]	22.9%	[20.9, 24.9%]
Mental health LSI	L	2.4%	[1.8, 3.0%]	20.4%	[19.1, 21.7%]
IHD/stroke LSI	L	-0.6%	[-0.9, -0.2%]	19.7%	[17.9, 21.5%]
Wheezing stopping sleep	S	-0.5%	[-1.0, 0.1%]	19.1%	[17.1, 21.1%]
Mini stroke (TIA) symptoms	S	-1.4%	[-2.4, -0.4%]	16.8%	[15.0, 18.6%]
Angina symptoms	S	-1.2%	[-1.6, -0.7%]	16.6%	[14.1, 19.1%]
Psychiatric morbidity symptoms	S	-1.3%	[-2.4, -0.3%]	15.2%	[14.6, 15.8%]
Arthritis LSI	L	-0.7%	[-1.4, 0.0%]	15.2%	[14.3, 16.1%]
Any recent CVD	L	0.5%	[-0.1, 1.2%]	14.4%	[12.7, 16.1%]
Heart attack symptoms	S	-0.5%	[-1.3, 0.3%]	14.1%	[12.6, 15.6%]
Anxiety/depression-moderately	S	0.1%	[-1.1, 1.3%]	13.6%	[13.0, 14.2%]
Pain-any	S	-3.3%	[-4.6, -2.0%]	12.9%	[12.4, 13.4%]
COPD symptoms	S	-1.6%	[-2.3, -0.8%]	12.6%	[11.0, 14.2%]
Diabetes LSI	L	2.1%	[1.5, 2.6%]	12.4%	[11.1, 13.7%]
Recent diabetes	L	2.2%	[1.9, 2.6%]	11.8%	[10.2, 13.4%]
Breathlessness-Grade 2+	S	-4.8%	[-6.1, -3.5%]	11.5%	[10.5, 12.5%]
Any CVD LSI	L	0.6%	[-0.1, 1.4%]	11.0%	[10.3, 11.7%]
Other musculoskeletal LSI	L	-0.8%	[-1.7, 0.1%]	9.8%	[9.2, 10.4%]
Glycated haemoglobin	B	2.1%	[1.4, 2.7%]	9.9%	[7.9, 11.9%]
Asthma LSI	L	0.7%	[0.0, 1.4%]	8.6%	[7.8, 9.4%]
Recent wheezing/asthma	S	-1.2%	[-2.5, 0.1%]	8.4%	[7.7, 9.1%]
Recent high blood pressure	L	4.8%	[3.9, 5.6%]	6.8%	[5.7, 7.9%]
BMI-Underweight	B	-0.1%	[-0.3, 0.1%]	6.2%	[4.3, 8.1%]
Diagnosed asthma	L	5.7%	[4.5, 6.8%]	5.9%	[5.1, 6.7%]
High waist-hip ratio	B	14.1%	[13.0, 15.2%]	4.6%	[4.1, 5.1%]
Raised fibrinogen	B	1.5%	[0.3, 2.6%]	4.3%	[1.9, 6.7%]
Low HDL cholesterol	B	-8.0%	[-9.0, -7.1%]	4.3%	[2.8, 5.8%]
Raised C-reactive protein	B	1.9%	[-0.7, 4.5%]	3.7%	[2.7, 4.7%]
BMI-Obese	B	8.9%	[8.0, 9.7%]	2.8%	[2.5, 3.1%]
Anaemia	B	-1.4%	[-2.7, -0.1%]	2.4%	[0.8, 4.0%]
Biomarker high blood pressure	B	-5.0%	[-5.6, -4.5%]	0.4%	[-0.3, 1.1%]
High total cholesterol	B	-17.6%	[-19.1, -16.1%]	0.0%	[-0.6, 0.6%]
Iron deficiency	B	-12.5%	[-14.8, -10.2%]	-0.5%	[-1.3, 0.3%]

For peer review only - http://bmjopen.bmj.com/site/about/guidelines.xhtml

Appendix 3: Sample adjustment & non-response weights

Sample coverage

As noted in the main paper, HSE is a household sample that excludes those in communal establishments. If we combine data from the 1991, 2001 and 2011 Censuses,[2] the communal population is as follows:

Table 5: Population in communal establishments over time (all working-age) and by age (in 2011)

		Education	Medical/ care	Defence	Prison	Other / not stated
**All working age**	*1991*	21,149	86,683	44,562	13,279	63,340
	2001	204,606	73,705	46,428	44,185	86,288
	2011	328,772	76,026	41,659	47,849	61,124
**16-24**	*2011*	305,154	9,346	22,677	12,607	25,673
**25-34**	*2011*	20,443	12,000	15,025	15,407	14,417
**35-49**	*2011*	2,663	26,796	3,725	14,725	14,708
**50-SPA[1] (est)**	*2011*	512	27,884	232	5,110	6,326

[1] SPA = State Pension Age, which is 60 for women and 65 for men. This is estimated because the Census totals are given for 50-64 year olds, so we have excluded 1/3 of women aged 50-64 from these totals.

This shows two things. Firstly, that there was a sharp rise in the working-age population in communal establishments 1991-2001 (from 230k to 560k), which was concentrated (>90% of the rise) among education-related communal establishments – although this is perhaps a slight overestimate given a definition change in the Census data.[3] Secondly, looking at education-related communal establishments in 2011, these are overwhelmingly (>90%) among 16-24 year olds. It therefore seems likely that the exclusion of communal establishments in HSE will lead to biases in young adults, and we therefore exclude 16-24 year olds from the trend analyses.

[2] Data are obtained from nomis on 6/8/2015, from Census tables DC1104EW and DC4210EWla (2011), S126 (2011) and L03/L04/L05 (2001).

[3] The guide to Census SARs notes, *"In the 1991 Census, students and schoolchildren were treated as usually resident at their 'home' or vacation address. In the 2001 census students and schoolchildren in full-time education studying away from the family home were enumerated as resident at their term-time address."* See https://census.ukdataservice.ac.uk/use-data/guides/microdata/comparability-91-01 [accessed 1/11/2016].

For peer review only - http://bmjopen.bmj.com/site/about/guidelines.xhtml

Sample weights

As noted in the main paper, HSE supplies non-response weights from 2003, including adjustments for non-response to the nurse visit and blood sample using health and socioeconomic status from the initial interview. However, there had been a substantial decline in response rates prior to 2003, as shown in the table below:

Table 6: Response rates to HSE

	Household	Individual	Self-comp.	BMI	Nurse	Blood
1991	85.3%	81.1%				
1992	81.8%	77.4%				
1993	80.8%	75.7%				
1994	77.4%	71.6%	71.2%	67.1%	63.3%	53.3%
1995	78.3%	72.9%	72.0%	66.8%	63.7%	
1996	79.4%	74.7%	73.7%	69.6%	66.1%	
1997	76.0%	71.1%	69.8%	66.9%	64.0%	
1998	74.0%	68.9%	66.7%	63.3%	59.6%	49.0%
1999	76.2%	70.3%	68.5%	63.6%		
2000	75.5%	68.4%	65.8%	60.5%	58.2%	
2001	74.2%	67.1%	64.5%	60.1%	54.2%	
2002	74 %	67 %	64.4%	59.6%	54.3%	
2003	72.7%	66.4%	64.1%	59.7%	52.2%	39.9%
2004	72.4%	65.6%	62.4%	56.1%		
2005	71.4%	64.1%	60.6%	54.8%	46.7%	
2006	68.1%	60.5%	57.7%	52.8%	45.4%	34.7%
2007	65.7%	58.3%	56.1%	51.3%	42.6%	
2008	64.5%	57.9%	55.9%	50.0%	41.5%	30.4%
2009	67.6%	61.0%	58.7%	52.5%	43.1%	33.7%
2010	66.1%	58.7%	54.9%	49.3%	39.1%	29.9%
2011	65.7%	58.9%	54.3%	49.0%	39.4%	29.8%
2012	64.1%	56.3%	52.5%	47.4%	36.3%	27.9%
2013	63.8%	57.6%	54.2%	49.3%	40.1%	31.2%
2014	61.6%	55.5%	51.5%	48.4%	37.3%	28.7%

In general these trends are due to increases in refusal rates. However, the blood sample response rate is affected by two noticeable changes in eligibility over this period (people who are pregnant or who had blood/clotting disorders were ineligible throughout):

1. In 1998, people who had ever had an epileptic fit were excluded from the blood sample. This raised the ineligibility rate to 3.5% of the sample in 1998, from 0.6% in 1994.
2. In 2010, this was then relaxed so that those who had had an epileptic fit more than 5 years ago were again included in the blood sample. This lowered the ineligibility rate from 3.1% in 2009 to 2.4% in 2010.

To try to increase the comparability over time, we create new weights 1994-2014 in three phases:

- Firstly, we created a selection weight because some households were slightly more likely to be interviewed than others. (Until 2009, only three households at each address were interviewed. Those living at addresses with many households are therefore less likely to be interviewed). NatCen supplied selection weights for 2004-2013 to enable this (funded by this project), which are not available on the public HSE datasets.

For peer review only

For peer review only - http://bmjopen.bmj.com/site/about/guidelines.xhtml

- Secondly, after adjusting for the first-stage selection weight, we created new individual-level (inverse probability) weights to match population age-sex-region totals in each year.[4] NatCen added the region variable for the 1994-1997 datasets to the public HSE datasets to enable this.
- Third, after the second-stage adjustment for individual non-response, for the later stages of the interview (self-completion, BMI measurement, nurse visit, blood sample), we created a further weight that adjusts for non-response among those responding to the individual interview. This is based on a logit regression model to predict nurse response based on:
  - Age and gender (4 age group categories interacted with gender);
  - Qualifications (degree or FT student / A-level or above / other qualifications / no qualifications);
  - Household type (presence of other adults in the household);
  - Employment status (yes/no);
  - Smoking (never regular smoker / ex-regular smoker / current regular smoker); and
  - Self-reported general health (bad or very bad health vs. other categories).

The revised weights are included in the Stata code to enable replication of the full paper. The final sample size is as follows:

Table 7: HSE sample size in each year

	Interview	Self-completion	Nurse visit	Blood sample
1994	9,948	9,884	8,786	7,399
1995	10,167	10,049	8,881	
1996	10,401	10,269	9,206	
1997	5,563	5,458	5,005	
1998	10,177	9,843	8,805	7,236
1999	5,008	4,884		
2000	5,188	4,993	4,417	
2001	10,002	9,613	8,079	
2002	4,662	4,482	3,775	
2003	9,420	9,089	7,395	5,665
2004	4,165	3,961		
2005	4,810	4,548	3,505	
2006	8,825	8,420	6,622	5,064
2007	4,198	4,039	3,064	
2008	9,242	8,922	6,625	4,845
2009	2,795	2,689	1,973	1,542
2010	5,120	4,794	3,411	2,610
2011	5,258	4,853	3,518	2,667
2012	4,936	4,605	3,188	2,447
2013	5,303	4,992	3,691	2,875
2014	4,909	4,552	3,297	2,531
**Total**	**140,097**	**134,939**	**103,243**	**44,881**

[4] Population data are annual mid-year population estimates from nomis.

For peer review only - http://bmjopen.bmj.com/site/about/guidelines.xhtml

Appendix 4: General self-reported health/disability

Trends in seven general health/disability measure are available in HSE:

Table 8: HSE general health measures

Measure	Operationalisation (years available)
Good general health	Health in general is 'good' or 'very good' (1994-2014)
Bad general health	Health in general is 'bad' or 'very bad' (1994-2014)
Longstanding illness (LSI)	Any long-standing illness, disability or infirmity (1994-2011)
Limiting LSI (LLSI)	LSI limits activities in any way (1996-2011)
Problems with activities-some	Some problems with performing usual activities (1996-2014)
Problems with activities-unable	Unable to perform usual activities (1996-2014)
Limitations in past 2wks	Cut down on activities in past 2wks due to LSI or other illness/injury (1994-2014)

See Web Appendix 2 for full details on all measures .

Trends for these measures are shown in Table 9 below. Looking first at good general health, the table shows the trend from 1994-6, when 80.9% reported good general health. By 2011-14, there had been a decline of 0.8 percentage points. When we adjust for the changing age and sex distribution of the working-age population (labelled 'Adj.' in Table 1), the decline is only 0.1%, with a wide confidence interval (-0.9 to +0.7%), and there is therefore little evidence for any systematic trend.

Table 9: Trends in general health

	Start of trend		Trend			
	Period	Incidence	End period	Raw	Adj.[a]	Adj. 95% CI
Good general health	1994-96	80.9%	2011-14	-0.8%	-0.1%	[-0.9, 0.7%]
Bad general health	1994-96	4.4%	2011-14	1.3%	1.0%	[0.6, 1.5%]
Longstanding illness (LSI)	1994-96	36.2%	2011-14	-1.0%	-2.0%	[-3.7, -0.3%]
Limiting LSI (LLSI)	1994-96	21.4%	2011-14	-2.9%	-3.6%	[-5.2, -2.1%]
Problems w/activities-some	1994-96	14.8%	2011-14	-1.2%	-1.8%	[-2.8, -0.8%]
Problems w/activities-unable	1994-96	1.9%	2011-14	-0.6%	-0.8%	[-1.1, -0.4%]
Limitations in past 2wks	1994-96	14.7%	2011-14	-0.1%	-0.3%	[-1.0, 0.4%]

[a] 'Adj.' = trend adjusted for changing age and sex distribution of the working-age population.

For several of the general health measures, there is more evidence of change over this period – but interpreting these is difficult, because the trends are in opposite directions. There is strong evidence for a *rise* in bad general health (a rise of 0.6-1.5% from a base of 4.4%), yet equally strong evidence for a *decline* in having problems with everyday activities (at both levels of severity), and being limited in activities by a longstanding illness. This shows the challenges in tracking population morbidity change through general, non-specific measures, which are likely to be as influenced by changes in reporting styles as much as changes in morbidity *per se*.

As an aside, UK Government publications have made claims based on healthy/disability-free life expectancy, most recently to argue that morbidity has been deteriorating. However, these trends are potentially misleading: they include older people as well as the working-age population; they confuse a combined mortality-morbidity measure with morbidity; and they are based on self-reports of global health that are unreliable, as we explore further below.

For peer review only - http://bmjopen.bmj.com/site/about/guidelines.xhtml

Appendix 5: Health measures

We systematically searched HSE questions, and have included every morbidity measure that is comparable over a significant duration. We have excluded questions only available for short time frames (ADLs 2012-14, EQ-5D visual analogue scale 2008-14, SF-12 1996-2000, eczema/hayfever 1995-2001, breathlessness 1991-98 and 1995-2001, lung function 1995-2001, bladder limitations 1995-2001, LDL cholesterol, triglycerides and glucose 1999-2003, IgE 1996-2002 and an alternate measure of high blood pressure 2009-14), with the exception of five key measures of activity limitations 1995-2001. We have also excluded questions that are not direct measures of health (medication or health service use, demispan, health risk factors such as fractures, accidents, alcohol/tobacco use (including biomarkers), physical activity, and wellbeing).

Short summaries of the resulting 39 measures are given in this paper, and full details are given in the table below. Measures are taken from the initial face-to-face survey unless otherwise specified. The Stata code to create these variables in consistent form from the publicly available HSE files are available from [authors' website].

Measure	Details
Activity limitations and MSDs	
Problems walking today	In the self-completion survey in 1996, 2003-6, 2008, 2010-12 and 2014, respondents were asked *'Now we would like to know how your health is today. Please answer ALL the questions. By ticking one box for each question below, please indicate which statements best describe your own health state today'*:<br>- *"I have no problems in walking about"*<br>- *"I have some problems in walking about"*<br>- *"I am confined to bed"*<br>[This is part of the widely-used EQ-5D health status indicator [2]. However, for the purposes of this paper we have separated the individual measures that make up the EQ-5D in order to compare these to similar indicators of morbidity within each domain].<br>People are classified as having a problem with self-care today if they had some problems walking about or were confined to bed.
Locomotor limitation	This is based on the personal care disability scale used in the 2001 HSE report [3]. Respondents in 1995, 2000 and 2001 were asked if any of the following applied to them (interviewers were instructed to ignore temporary disabilities that are expected to last less than one year):<br>- *"Cannot walk 200 yards or more on own without stopping or discomfort"*. People who reported a limitation were asked if they used a walking aid, and if they did, were then asked if they could walk 200 yards without the walking aid.<br>- *"Cannot walk up and down a flight of 12 stairs without resting"*<br>- *"Cannot bend down and pick up a shoe from the floor when standing"*<br>People are classified as having a locomotor limitation if they reported ANY of these limitations.
Problems with washing/dressing today	In the self-completion survey in 1996, 2003-6, 2008, 2010-12 and 2014, respondents were asked *'Now we would like to know how your health is today. Please answer ALL the questions. By ticking one box for each question below, please indicate which statements best describe your own health state today'*:<br>- *"I have no problems with self-care"*<br>- *"I have some problems washing or dressing myself"*<br>- *"I am unable to wash or dress myself"*<br>[This is part of the widely-used EQ-5D health status indicator [2]. However, for the purposes of this paper we have separated the individual measures that make up the EQ-

For peer review only - http://bmjopen.bmj.com/site/about/guidelines.xhtml

	5D in order to compare these to similar indicators of morbidity within each domain].<br>People are classified as having a problem with self-care today if they had some problems washing/dressing or were unable to wash/dress themselves.
Self-care limitation	This is based on the personal care disability scale used in the 2001 HSE report [3]. Respondents in 1995, 2000 and 2001 were asked if any of the following applied to them (interviewers were instructed to ignore temporary disabilities that are expected to last less than one year):<br>- *"Cannot get in and out of bed on own without difficulty"*<br>- *"Cannot get in and out of a chair without difficulty"*<br>- *"Cannot dress and undress without difficulty"*<br>- *"Cannot wash hands and face without difficulty"*<br>- *"Cannot feed, including cutting up food without difficulty"*<br>- *"Cannot get to and use toilet on own without difficulty"*<br>People are classified as having a self-care limitation if they reported ANY of these limitations.
Pain<br>*(any / extreme)*	In the self-completion survey in 1996, 2003-6, 2008, 2010-12 and 2014, respondents were asked *'Now we would like to know how your health is today. Please answer ALL the questions. By ticking one box for each question below, please indicate which statements best describe your own health state today'*:<br>- *"I have no pain or discomfort"*<br>- *"I have moderate pain or discomfort"*<br>- *"I have extreme pain or discomfort"*<br>[This is part of the widely-used EQ-5D health status indicator [2]. However, for the purposes of this paper we have separated the individual measures that make up the EQ-5D in order to compare these to similar indicators of morbidity within each domain].<br>Two outcome measures are based on this: whether people have any pain (the 2nd and 3rd categories combined), and whether they have extreme pain (3rd category only).
Arthritis LSI	Every year 1994-2011, people who report a longstanding illness (LSI) are then asked, *'what is the matter with you?'*; up to 6 responses are then coded by the interviewer into a consistent coding frame based on the International Classification of Diseases.<br>The arthritis LSI measure is based on the group labelled *'Arthritis/rheumatism/fibrositis'*, which as of 2011 includes: Arthritis as result of broken limb; Arthritis/rheumatism in any part of the body; Gout; Osteoarthritis, rheumatoid arthritis, polymyalgia rheumatic; Polyarteritis Nodosa; Psoriasis arthritis; Rheumatic symptoms; and Still's disease.<br>While the LSI coding frame generally stays consistent over this period, interpretation of 'LSI arthritis' is complicated by two changes: Gout and Polyarteritis Nodosa are moved into this code (the documentation is not clear on whether this occurred in 2000 or 2001).
Other musculoskeletal LSI	People who report a longstanding illness (LSI) are then asked, *'what is the matter with you?'*; up to 6 responses are then coded by the interviewer into a consistent coding frame based on the International Classification of Diseases.<br>The other musculoskeletal LSI measure is based on the groups labelled *'Back problems/slipped disc/spine/neck'* and *'Other problems of bones/joints/muscles'*, which as of 2011 includes: Brittle bones, osteoporosis; Bursitis, housemaid's knee, tennis elbow; Cartilage problems; Chondrodystrophia; Chondromalacia; Cramp in hand; Deformity of limbs eg. club foot, claw-hand, malformed jaw; Delayed healing of bones or badly set fractures; Deviated septum; Disc trouble; Dislocations eg. dislocation of hip, clicky hip, dislocated knee/finger; Disseminated lupus; Dupuytren's contraction; Fibromyalgia; Flat feet, bunions; Fracture, damage or injury to extremities, ribs, collarbone, pelvis, skull, eg. knee injury, broken leg, gun shot wounds in leg/shoulder, can't hold arm out flat - broke it as a child, broken nose; Frozen shoulder; Hip infection, TB hip; Hip replacement (nes); Legs won't go, difficulty in walking; Lumbago, inflammation of spinal joint; Marfan Syndrome; Osteomyelitis; Paget's disease; Perthe's disease; Physically handicapped (nes); Pierre Robin syndrome; Prolapsed invertebral discs; Schlatter's disease; Schuermann's

For peer review only - http://bmjopen.bmj.com/site/about/guidelines.xhtml

	disease; Sever's disease; Spondylitis, spondylosis; Stiff joints, joint pains, contraction of sinews, muscle wastage; Strained leg muscles, pain in thigh muscles; Systemic sclerosis, myotonia (nes); Tenosynovitis; Torn muscle in leg, torn ligaments, tendonitis; Walk with limp as a result of polio, polio (nes), after affects of polio (nes); Weak legs, leg trouble, pain in legs; and Worn discs in spine - affects legs. The code explicitly excludes: Damage/injury to spine results in paralysis; Sciatica or trapped nerve in spine; and Muscular dystrophy.
Circulatory	
High blood pressure LSI	Every year 1994-2011, people who report a longstanding illness (LSI) are then asked, *'what is the matter with you?'*; up to 6 responses are then coded by the interviewer into a consistent coding frame based on the International Classification of Diseases.<br>The high blood pressure LSI measure is based on the group labelled *'Hypertension/high blood pressure/blood pressure (nes)'*, which as of 2011 includes only the conditions listed in the group label.
Recent high blood pressure	Respondents in 1994, 1998, 2003, 2006 and 2009-2014 were asked a series of questions on whether they have high blood pressure:<br>- *"Do you now have, or have you ever had… high blood pressure (sometimes called hypertension)?"*<br>- Those responding 'yes' were then asked *"Were you told by a doctor or nurse that you had high blood pressure?"*<br>- Women responding 'yes' were then asked, *"Can I just check, were you pregnant when you were told that you had high blood pressure?"*, and those responding 'yes' were then asked *"Have you ever had high blood pressure apart from when you were pregnant?"*<br>- Finally, those with doctor-diagnosed high blood pressure (excluding only when pregnant were asked: *"Are you currently taking any medicines, tablets or pills for high blood pressure?*", and those saying 'no' (or not giving an answer) were then asked, *"Do you still have high blood pressure?"*<br>People were considered to have recent high blood pressure if they said they had ever been diagnosed as having high blood pressure by a doctor (excluding when pregnant), and that they still have high blood pressure or are currently taking medicines for it.<br>While the question wording has stayed consistent, a discontinuity seems to be introduced by a change in question context. In some years (1994, 1998, 2003, 2006 and 2011), this question was preceded by a question that asked, *"May I just check, have you ever had your blood pressure measured by a doctor or nurse?"* (and then for those saying yes, they were asked how recently this was, and whether they were told that it was *'normal (alright/fine), higher than normal, lower than normal, or were you not told anything?'*). However, in other years (2009-10, 2012-14), this question was not asked. Given the way in which context can affect question interpretation, we treat these as two separate measures of recent high blood pressure.
Biomarker high blood pressure	During the nurse visit (which took place for all consenting respondents in all years except 1999, 2002 and 2004, when the nurse visit focussed on particular subsamples), respondents' blood pressure was measured.<br>High blood pressure is defined as a systolic blood pressure >= 140mmHg and diastolic blood pressure >= 90mmHg following HSE established practice, in turn following [4].<br>The measurement of blood pressure changed in 2003, from a Dinamap monitor to an Omron monitor. A conversion is available between the two monitors based on a calibration study, and this has been regularly used by the HSE team to produce continuous trends in blood pressure – see www.hscic.gov.uk/catalogue/PUB00480. For adults, the conversion is as follows:<br>○ For systolic blood pressure: *Predicted Omron=8.90 (SE=2.94) + 0.91 (SE=0.02) * Dinamap.*<br>○ For diastolic blood pressure: *Predicted Omron=19.78 (SE=1.86) + 0.73 (SE=0.03) * Dinamap.*<br>There are several reasons why respondents who had a nurse visit do not have a valid

For peer review only - http://bmjopen.bmj.com/site/about/guidelines.xhtml

	blood pressure measurement – these are discussed in the missing data appendix that follows.
High cholesterol	In the years 1994, 1998, 2006, and 2008-14, blood samples were obtained during the nurse visit, which were then analysed for total cholesterol. A high level of total cholesterol ('hypercholesterolaemia') is an established risk factor for CVD, and high cholesterol is defined following conventional practice at the NICE guidance 'audit level' of 5mmol/L or above [5 6].<br>The measurement of cholesterol changed slightly in 2010 when a new laboratory was used. This resulted in values that are an average of 0.1mmol/L higher, and later values are therefore adjusted by this amount to maintain comparability over time as in [5].
Low HDL cholesterol	In the years 1994, 1998, 2006, and 2008-14, blood samples were obtained during the nurse visit, which were then analysed for high density lipoprotein (HDL) cholesterol. HDL cholesterol *reduces* the risk of CVD (it carries cholesterol away from the arteries towards the liver), and it is therefore low HDL cholesterol that indicates poorer health; low HDL cholesterol is here defined as 1 mmol/L or less [5 6].<br>The measurement of HDL cholesterol changed slightly in 2010 when a new laboratory was used. This resulted in values that are an average of 0.1mmol/L lower, and later values are therefore adjusted by this amount to maintain comparability over time as in [5].
Recent heart attack/stroke	Respondents in 1994, 1998, 2003, 2006 and 2011 were asked a series of questions on whether they have had a heart attack (within a battery of questions about different types of heart disease):<br>- *"Have you ever had a heart attack (including myocardial infarction or coronary thrombosis)?"*<br>- Those responding 'yes' were then asked *"Were you told by a doctor that you had a Heart Attack (including myocardial infarction or coronary thrombosis)?"*<br>- Those with doctor-diagnosed angina were asked, *"Have you had a heart attack (including myocardial infarction and coronary thrombosis) during the past 12 months?"*<br>Respondents in these years were similarly asked about stroke:<br>- *"Have you ever had a stroke?"*<br>- Those responding 'yes' were then asked, *"Were you told by a doctor that you had a stroke?"*<br>- Those with doctor-diagnosed stroke were asked, *"Have you had a stroke during the past 12 months?"*<br>People were considered to have recent IHD or stroke if they said they had ever been diagnosed as having stroke or a heart attack by a doctor, and that they have had a heart attack or stroke during the past 12 months.
Recent angina	Respondents in 1994, 1998, 2003, 2006 and 2011 were asked a series of questions on whether they have angina (within a battery of questions about different types of heart disease):<br>- *"Have you ever had angina?"*<br>- Those responding 'yes' were then asked *"You said that you had Angina. Were you told by a doctor that you had Angina?"*<br>- Those with doctor-diagnosed angina were asked, *"Have you had angina during the past 12 months?"*<br>People were considered to have recent angina if they said they had ever been diagnosed as having angina by a doctor, and that they have had it during the past 12 months.
IHD LSI	Every year 1994-2011, people who report a longstanding illness (LSI) are then asked, *'what is the matter with you?'*; up to 6 responses are then coded by the interviewer into a consistent coding frame based on the International Classification of Diseases.<br>The IHD LSI measure is based on the groups labelled *'Stroke/cerebral haemorrhage/cerebral thrombosis'* and *'Heart attack/angina'*. As of 2011 this includes: Cerebro-vascular accident; Coronary thrombosis, myocardial infarction; Heart attack/angina; Hemiplegia, apoplexy, cerebral embolism; Stroke/cerebral haemorrhage/cerebral thrombosis; and Stroke victim - partially paralysed and speech difficulty.

For peer review only - http://bmjopen.bmj.com/site/about/guidelines.xhtml

Recent cardiovascular disease (CVD)	Respondents in 1994, 1998, 2003, 2006 and 2011 were asked a series of questions on different types of heart disease – including angina; heart attack (including myocardial infarction or coronary thrombosis); a heart murmur; abnormal heart rhythm; or other heart trouble. For EACH of these, they were asked:<br>- *"Have you ever had <type of heart disease>?"*<br>- Those responding 'yes' were then asked *"You said that you had <type of heart disease>. Were you told by a doctor that you had <type of heart disease>?"*<br>- For heart murmurs only, women saying they had doctor-diagnosed heart murmurs were asked if they were pregnant when told this, and if so, whether they were ever told they had a heart murmur when they were not pregnant.<br>- Those with doctor-diagnosed heart disease (excluding heart murmurs when pregnant) were asked, *"Have you had <type of heart disease> during the past 12 months?"*<br>People were considered to have recent CVD if they said they had a doctor-diagnosed heart condition and that they had had this during the past 12 months.
Cardiovascular (CVD) LSI	Every year 1994-2011, people who report a longstanding illness (LSI) are then asked, *'what is the matter with you?'*; up to 6 responses are then coded by the interviewer into a consistent coding frame based on the International Classification of Diseases.<br>The CVD LSI measure is based on the groups labelled *'Stroke/cerebral haemorrhage/cerebral thrombosis', 'Heart attack/angina', Hypertension/high blood pressure/blood pressure (nes)', 'Other heart problems', 'Piles/haemorrhoids incl. Varicose Veins in anus', 'Varicose veins/phlebitis in lower extremities', and 'Other blood vessels/embolic'*. As of 2011 this includes: Aorta replacement; Aortic valve stenosis; Aortic/mitral valve regurgitation; Arterial thrombosis; Arteriosclerosis, hardening of arteries (nes); Artificial arteries (nes); Atrial Septal Defect (ASD); Blocked arteries in leg; Blood clots (nes); Cardiac asthma; Cardiac diffusion; Cardiac problems, heart trouble (nes); Cerebro-vascular accident; Coronary thrombosis, myocardial infarction; Dizziness, giddiness, balance problems (nes); Hand Arm Vibration Syndrome (White Finger); Hardening of arteries in heart; Heart attack/angina; Heart disease, heart complaint; Heart failure; Heart murmur, palpitations; Hemiplegia, apoplexy, cerebral embolism; Hole in the heart; Hypersensitive to the cold; Hypertension/high blood pressure/blood pressure (nes); Intermittent claudication; Ischaemic heart disease; Low blood pressure/hypertension; Mitral valve stenosis; Pacemaker; Pains in chest (nes); Pericarditis; Piles/haemorrhoids incl. Varicose Veins in anus; Poor circulation; Pulmonary embolism; Raynaud's disease; St Vitus dance; Stroke victim - partially paralysed and speech difficulty; Stroke/cerebral haemorrhage/cerebral thrombosis; Swollen legs and feet; Tachycardia, sick sinus syndrome; Telangiectasia (nes); Thrombosis (nes); Tired heart; Valvular heart disease; Valvular heart disease; Varicose veins in Oesophagus; Varicose veins/phlebitis in lower extremities; Various ulcers, varicose eczema; Weak heart because of rheumatic fever; Wolff - Parkinson - White syndrome; and Wright's syndrome. It explicitly excludes balance problems due to ear complaint & haemorrhage behind eye.<br>While the LSI coding frame generally stays consistent over this period, interpretation of 'IHD LSI' is complicated by two changes: 'Too much cholesterol in blood' is included in this category in 1994 only, and Polyarteritis Nodosa is later moved into this code (the documentation is not clear on whether this occurred in 2000 or 2001).
Angina symptoms	This is taken from the Rose Angina questionnaire [7,8]. Respondents in 1994, 1998, 2003, 2006 and 2011 were asked a series of questions about *symptoms* of heart trouble (rather than whether they had been diagnosed):<br>- *"I am now going to ask you some questions mainly about symptoms of the chest. Have you ever had any pain or discomfort in your chest?"*<br>- Those that said 'yes' were asked:<br>o *Do you get it when you walk uphill or hurry? Yes | No | Sometimes/ Occasionally | Never walks uphill or hurries | (Cannot walk)"*. If sometimes/occasionally, they were asked: *"Does this happen on most occasions?"*<br>o If not 'no' to having pain/discomfort in their chest, they were asked: *"Do*

For peer review only - http://bmjopen.bmj.com/site/about/guidelines.xhtml

	you get it when you walk at an ordinary pace on the level? Yes | No | Sometimes/Occasionally | Never walks at an ordinary pace on the level". If sometimes/occasionally, they were asked: *"Does this happen on most occasions?"*<br>- Those who every had pain/discomfort when walking uphill/hurrying or walking at ordinary pace on the level were asked:<br>○ *"What do you do if you get it while you are walking? Do you stop, slow down or carry on?"* (If respondents were unsure, they were asked, *"What do you do on most occasions?")*<br>○ Those who said they stop or slow down were asked, *"If you stand still does the pain go away or not?"* (If respondents were unsure, they were asked, *"What happens to the pain on most occasions?").* If the pain goes away, they were asked, *"How soon does the pain go away? Does it go in 10 minutes or less, or more than 10 minutes?"*<br>○ Those who said the pain goes away in 10 minutes or less were asked, *"Will you show me where you get this pain or discomfort? Where else"* The interviewer then coded the site as Sternum (upper or middle) | Sternum lower | Left anterior chest | Left arm | Right anterior chest | Right arm | (Somewhere else).<br>Following the HSE reports, possible angina is defined as chest pain or discomfort that (i) includes either the sternum or the left arm and left anterior chest; (ii) is prompted by hurrying or walking uphill (or by walking on the level, for those who never attempt more); (iii) makes the respondent either stop or slacken pace; and (iv) usually disappears in 10 minutes or less when they stand still.
Heart attack symptoms	This is taken from the Rose Angina questionnaire. Respondents in 1994, 1998, 2003, 2006 and 2011 were asked, *"Have you ever had a severe pain across the front of your chest lasting for half an hour or more?"* As in the 2006 HSE report, those responding 'yes' are treated as having a possible heart attack (myocardial infarction).
Mini stroke (TIA) symptoms	Respondents in 2003, 2006 and 2011 were asked:<br>○ *"In the last twelve months, have you had a sudden attack of weakness or numbness on one side of the body?"*<br>○ *"Have you had a sudden attack of slurred speech or difficulty in finding words in the last twelve months?"*<br>○ *"Have you had a sudden attack of vision loss or blurred vision in one or both eyes in the last twelve months?"*<br>People reporting ANY of these symptoms were considered as possibly having had a transient ischaemic attack (TIA), often called a 'mini stroke'.
Respiratory	
COPD symptoms	Respondents in 1995, 1996 and 2010 were asked:<br>○ *"Do you usually cough first thing in the morning in the winter?"* (In 2010 only, respondents had previously been asked *"Do you usually cough first thing in the morning?"* – but this is not used to filter people into the questions on coughing in winter).<br>○ *"Do you usually bring up any phlegm from your chest, first thing in the morning in the winter?"* (Again, this was asked to everyone in all years, but was preceded by an additional, non-winter-specific question in 2010).<br>○ Those saying 'yes' to each question were then asked, "*Do you [cough/bring up phlegm] like this on most days for as much as three months each year?"* In 2010 only, this was followed by the additional clarification '*That is, for three consecutive months'.*<br>People who reported three months/year of BOTH coughing first thing and of phlegm are considered to have possible symptoms of Chronic Obstructive Pulmonary Disease (COPD).
Diagnosed asthma	In 1995-7, 2001 and 2010, respondents were asked *"Did a doctor <1997 and 2010 only: or*

For peer review only

For peer review only - http://bmjopen.bmj.com/site/about/guidelines.xhtml

	nurse> ever tell you that you had asthma?" Whereas for other doctor-diagnosed conditions (heart problems/diabetes) we focus on those reporting problems in the past 12 months, it is not possible to construct a consistent measure of recent asthma, hence this variable refers to *lifetime* doctor-diagnosed asthma.
Asthma LSI	Every year 1994-2011, people who report a longstanding illness (LSI) are then asked, *'what is the matter with you?'*; up to 6 responses are then coded by the interviewer into a consistent coding frame based on the International Classification of Diseases.<br>The asthma LSI measure is based on the group labelled *'Asthma'*, which as of 2011 includes: Asthma; Bronchial asthma, allergic asthma; and Asthma - allergy to house dust/grass/cat fur. It explicitly <u>excludes</u> cardiac asthma.
Shortness of breath *(Grade 2+ / Grade 3)*	Respondents in 1995, 1996 and 2010 were asked the following questions about shortness of breath ('dyspnoea'):<br>○ *"Are you troubled by shortness of breath when hurrying on level ground or walking up a slight hill? Yes | No | Never walks up hill or hurries | Cannot walk"*<br>○ Those responding 'yes' or 'never walks up hill or hurries' are then asked, *"Do you get short of breath walking with other people of (your/his/her) own age on level ground? Yes | No | Never walks with people of own age on level ground"*.<br>○ Those responding 'yes' or 'never walks with people of own age' are then asked, *"Do you have to stop for breath after walking at (your/his/her) own pace on level ground?"*<br>This has been combined into the longstanding MRC dyspnoea scale [9] as follows:<br>- Grade 2 dyspnoea: people who report shortness of breath when hurrying on level ground or walking up a slight hill (or who report shortness of breath when walking on level ground, but who say they never walk up hill or hurry).<br>- Grade 3 dyspnoea: people who report shortness of breath when walking with people of own age on level ground, or who have to stop for breath when walking at own pace on level ground.<br>(The same questions also exist in 1994 and 1998, but (i) the wider bank of questions differs substantially in the two versions and question context effects are likely; and (ii) the filtering into the final question differs between versions. However, the 1991-98 trends are included below).
Recent wheezing/ asthma symptoms	Respondents in 1995-97, 2001 and 2010 were asked the following two questions as part of the battery of questions on breathing problems:<br>- *"I am now going to ask you some questions about your breathing... Have you ever had wheezing or whistling in the chest at any time, either now, or in the past?"*<br>- Those that said yes were then asked, *"Have you had wheezing or whistling in the chest in the last 12 months?"*<br>- (For those who said they had ever been told by a doctor they had asthma; see above), "*When was your most recent attack of asthma? PROMPT IF NECESSARY: Less than 4 weeks ago | More than 4 weeks but within the last 12 months | One to five years ago | More than 5 years ago"*<br>People who said they had EITHER wheezing/whistling in the past 12 months or an asthma attack in the past 12 months were counted as having recent wheezing/asthma symptoms.<br>[It should be noted that the filtering to the second question is very slightly different in 2010 compared to previous years (it was only asked to people who said they had not had wheezing/whistling in the chest in the past 12 months). However, given the way that the derived variable is calculated here, the change in filtering does not introduce any discontinuities over time].
Wheezing stopping sleep	Respondents in 1995-97, 2001 and 2010 were asked the following two questions as part of the battery of questions on breathing problems:<br>- *"I am now going to ask you some questions about your breathing... Have you ever had wheezing or whistling in the chest at any time, either now, or in the past?"*<br>- Those that said yes were then asked, *"Have you had wheezing or whistling in the chest in the last 12 months?"*

For peer review only - http://bmjopen.bmj.com/site/about/guidelines.xhtml

	- Those that said yes were then asked, *"In the last 12 months, how often on average has your sleep been disturbed due to wheezing or whistling in the chest?: Have you: Never woken with wheezing | Woken less than one night per week, or | Woken one or more nights per week?"*<br>People were considered to have wheezing during sleep if they reported this at least once per week.
Anthropometric & diabetes	
BMI<br>*(Underweight / Obese)*	During the initial face-to-face interview in all years (except 2013), respondents were asked if they would consent to having their height and weight measured by the interviewer. The reasons for missingness (and their trends over time) are given in the following web appendix; note that there are three changes that give rise to small discontinuities in 2009 and 2011.<br>Obesity is a risk factor for diabetes (hence its inclusion in this section) but also heart disease and some cancers. Obesity is defined as a Body Mass Index (BMI) of >= 30kg/m^2 as per the World Health Organization's BMI classification [10]. Using the same definition, underweight is defined as <=18.5 kg/m^2.
High waist-hip ratio	During the nurse visit in most years (excluding 1995-96, 2002, 2004 and 2013), respondents had their waist and hip circumferences measured. While BMI is a standard measurement of obesity, some evidence suggests that fat around the waist – 'central adiposity' – is a greater risk to health than fat elsewhere [11]. We use NICE's suggested 2006 thresholds for a high waist-hip ratio of >1 for men and >0.85 for women [12], as used in Hotchkiss et al [13].
Recent diabetes	Respondents in 1994, 1998, 2003, 2006 and 2009-2014 were asked a series of questions on whether they have diabetes:<br>- *"Do you now have, or have you ever had diabetes?"*<br>- Those responding 'yes' were then asked *"Were you told by a doctor that you had diabetes?"*<br>- Women responding 'yes' were then asked, *"Can I just check, were you pregnant when you were told that you had diabetes?"*, and those responding 'yes' were then asked *"Have you ever had diabetes apart from when you were pregnant?"*<br>- Finally, those with doctor-diagnosed diabetes (excluding only when pregnant were asked: *"Do you currently inject insulin for diabetes?"* and *"Are you currently taking any medicines, tablets or pills (other than insulin injections) for diabetes?"*<br>People were considered to have recent diabetes if they said they had ever been diagnosed as having diabetes by a doctor (excluding when pregnant), and that they are injecting insulin or taking any other medicines for diabetes.
Diabetes LSI	Every year 1994-2011, people who report a longstanding illness (LSI) are then asked, *'what is the matter with you?'*; up to 6 responses are then coded by the interviewer into a consistent coding frame based on the International Classification of Diseases.<br>The diabetes LSI measure is based on the group labelled *'Diabetes'*, which as of 2011 includes Diabetes and Hyperglycaemia.
High glycated haemoglobin	In the years 2003, 2006, and 2008-14, blood samples were obtained during the nurse visit, which were then analysed for glycated haemoglobin (HbA_{1C}). HbA_{1C} is a measure of the share of haemoglobin (within red blood cells) that glucose is attached to, with higher levels indicated less well-controlled diabetes in the previous three months [14]. Following the recommendations of a 2009 expert committee, we mirror recent HSE reports in using a threshold of 48mmol/mol (i.e. 48 millimoles of glycated haemoglobin per mole of haemoglobin) as the threshold for raised HbA_{1C}, a different threshold to that used in earlier HSE reports.<br>While the measurement of HbA_{1C} has been consistent in HSE from 1994, the units reported have changed from the % of haemoglobin that is glycated to mmol/mol. Earlier measures have been transformed into mmol/mol through the formula, mmol/mol = (% - 2.15) x 10.929. HbA_{1C} was also measured in 1994 but using a different technique, which cannot be made comparable [15:67].

For peer review only - http://bmjopen.bmj.com/site/about/guidelines.xhtml

Other biomarkers	
Raised C-reactive protein	In the years 1998, 2003, 2006, and 2009, blood samples were obtained during the nurse visit, which were then analysed for C-reactive protein (CRP). CRP is an inflammatory marker, which can indicate heart-related inflammation (it is used to test for heart failure) but can also indicate other sorts of health damage including diabetes. However, there are still debates about exactly what CRP shows, both in terms of its causal role in heart disease, and whether it also indicates depression [16].<br>Raised CRP is defined as >3mg/L, the standard cut-off for a clinically significant rise in CVD [17,18]. Participants with CRP >10mg/L are excluded, as this is taken to be evidence of current infection rather than inflammation from chronic disease.
Raised Fibrinogen	In the years 1998, 2003, 2006, and 2009, blood samples were obtained during the nurse visit, which were then analysed for fibrinogen. Like CRP, fibrinogen is an inflammatory marker, which is both commonly thought to be a causal risk factor for CVD (it is a component of coagulation), and which seems to be a risk factor for other diseases (including cancer and diabetes)[19].<br>While fibrinogen is often analysed as a continuous variable with no cutpoints [18], we here define raised fibrinogen as>4mg/L as in [6]. As for CRP, participants with CRP >10mg/L are excluded, as this is taken to be evidence of current infection rather than inflammation from chronic disease. A change of analysis method and laboratory between 1994 and 1998 means that the 1994 results are not comparable to the later results [20:8.10.4].
Anaemia	In the years 1994, 1998, 2006, and 2009, blood samples were obtained during the nurse visit, which were then analysed for haemoglobin. Haemoglobin dist ributes oxygen around the body, and low haemoglobin levels usually indicate anaemia. Various different thresholds for low haemoglobin have been used in the literature, particularly for older populations [21], but we here used the longstanding WHO definition of <13g/dL for men and <12g/dL for women [18].
Iron deficiency	In the years 1994, 1998, 2006, and 2009, blood samples were obtained during the nurse visit, which were then analysed for serum ferritin (which correlates directly with the amount of iron stored in the body). Iron deficiency is one of several possible causes of anaemia (alongside other nutritional deficiencies, genetic conditions such as sickle cell anaemia, infections, and blood loss). Iron deficiency is defined as a serum ferritin less than 45ng/ml [21].
Mental health	
Mental health LSI	Every year 1994-2011, people who report a longstanding illness (LSI) are then asked, *'what is the matter with you?'*; up to 6 responses are then coded by the interviewer into a consistent coding frame based on the International Classification of Diseases.<br>The mental health LSI measure is based on the group labelled *'Mental illness/anxiety/depression/nerves (nes)'*, which as of 2011 includes: Alcoholism, recovered not cured alcoholic; Angelman Syndrome; Anorexia nervosa; Anxiety, panic attacks; Asperger Syndrome; Autism/Autistic {BBG: changed from 'autistic child'}; Bipolar Affective Disorder; Catalepsy; Concussion syndrome; Depression; Drug addict; Dyslexia; Hyperactive child.; Nerves (nes); Nervous breakdown, neurasthenia, nervous trouble; Phobias; Schizophrenia, manic depressive; Senile dementia, forgetfulness, gets confused; Speech impediment, stammer; and Stress. It explicitly excludes Alzheimer's disease, degenerative brain disease.<br>While the LSI coding frame generally stays consistent over this period, it is worth being aware of a minor wording change within 'mental health LSI': the condition labelled 'Autistic child' 1994-1997 was relabelled 'Autism/Autistic' in 1998.
Psychiatric morbidity (GHQ)	In the self-completion survey in most years (except 1996, 2007, 2011 and 2013), respondents were asked the following series of questions:<br>- *"Please read this carefully: We should like to know how your health has been in general over the past few weeks. Please answer ALL the questions by ticking the box below the answer which you think most applies to you. Have you recently...*<br>- *"…been able to concentrate on whatever you're doing?"* RESPONSES: *"Better than usual" | "Same as usual" | "Less than usual" | "Much less than usual"*

For peer review only - http://bmjopen.bmj.com/site/about/guidelines.xhtml

- *“…lost much sleep over worry?”* *RESPONSES: “Not at all” | “No more than usual” | “Rather more than usual” | “Much more than usual”"*
- *“…felt you were playing a useful part in things?”* *RESPONSES: “More so than usual” | “Same as usual” | “Less useful than usual” | “Much less useful”"*
- *“…felt capable of making decisions about things?”* *RESPONSES: “More so than usual” | “Same as usual” | “Less so than usual” | “Much less capable”"*
- *“…felt constantly under strain?* *RESPONSES: “Not at all” | “No more than usual” | “Rather more than usual” | “Much more than usual”"*
- *“..felt you couldn't overcome your difficulties?”* *RESPONSES: “Not at all” | “No more than usual” | “Rather more than usual” | “Much more than usual”"*
- *“…been able to enjoy your normal day-to-day activities?”* *RESPONSES: “More so than usual” | “Same as usual” | “Less so than usual” | “Much less than usual”*
- *“…been able to face up to your problems?”* *RESPONSES: “More so than usual” | “Same as usual” | “Less able than usual” | “Much less able”*
- *“…been feeling unhappy and depressed?* *RESPONSES: “Not at all” | “No more than usual” | “Rather more than usual” | “Much more than usual”*
- *“…been losing confidence in yourself?* *RESPONSES: “Not at all” | “No more than usual” | “Rather more than usual” | “Much more than usual”*
- *“…been thinking of yourself as a worthless person?”* *RESPONSES: “Not at all” | “No more than usual” | “Rather more than usual” | “Much more than usual”"*
- *“…been feeling reasonably happy, all things considered?”* *RESPONSES: “More so than usual” | “Same as usual” | “Less so than usual” | “Much less happy”*

These make up the 12-item General Health Questionnaire GHQ-12; [22], a well-validated, widely-used measure of probable mental ill-health (or more strictly, of general non-psychotic psychiatric morbidity).

A total score has been created by first ensuring that all questions were coded from 1 (positive symptom) to 4 (negative symptom), and then creating a sum score for all the number of questions in which people answered with categories 3 or 4 (indicating a negative symptom). A binary measure (often called GHQ caseness) was created for people who had negative symptoms for 4 or more of the 12 questions.

Anxiety/depression *(moderately / Extremely)*

In the self-completion survey in 1996, 2003-6, 2008, 2010-12 and 2014, respondents were asked *‘Now we would like to know how your health is today. Please answer ALL the questions. By ticking one box for each question below, please indicate which statements best describe your own health state today’:*

- *“I am not anxious or depressed”*
- *“I am moderately anxious or depressed”*
- *“I am extremely anxious or depressed”*

[This is part of the widely-used EQ-5D health status indicator [2]. However, for the purposes of this paper we have separated the individual measures that make up the EQ-5D in order to compare these to similar indicators of morbidity within each domain].

Two outcome measures are based on this: whether people have any anxiety/depression (the 2nd and 3rd categories combined), and whether they have extreme anxiety/depression (3rd category only).

Communication

Hearing, seeing & communication limitations

These measures were not included in the main paper due to the short time frame that we can examine trends over, but are included in the Web Appendix as they relate to important domains of morbidity.

They were included in the disability scale used in the 2001 HSE report [3]. Respondents in 1995, 2000 and 2001 were asked if of the following applied to them (interviewers were instructed to ignore temporary disabilities that are expected to last less than one year):

- *“Cannot follow a TV programme at a volume others find acceptable (with hearing aid if normally worn)”* (‘hearing limitation’)
- *“Cannot see well enough to recognise a friend across a road (four yards away) (with glasses or contact lenses if normally worn)”* (‘seeing limitation’)

For peer review only - http://bmjopen.bmj.com/site/about/guidelines.xhtml

	• *"Have problem communicating with other people - that is, have problem understanding them or being understood by them"* ('communication limitation')
Eye/Ear LSI	Every year 1994-2011, people who report a longstanding illness (LSI) are then asked, *'what is the matter with you?'*; up to 6 responses are then coded by the interviewer into a consistent coding frame based on the International Classification of Diseases. The Eye/Ear LSI includes the following groups:<br>• *Poor hearing/deafness*, including Conductive/nerve/noise induced deafness, Deaf mute/deaf and dumb, Heard of hearing, slightly deaf, Otosclerosis, Poor hearing after mastoid operation.<br>• *Tinnitus/noises in the ear*, Incl. pulsing in the ear<br>• *Other ear complaints*, Incl. otitis media - glue ear, Disorders of Eustachian tube, Perforated ear drum (nes), Middle/inner ear problems, Mastoiditis, Ear trouble (nes),, Ear problem (wax), Ear aches and discharges, Ear infection<br>• *Cataract/poor eye sight/blindness*, Incl. operation for cataracts, now need glasses, Bad eyesight, restricted vision, partially sighted, Bad eyesight/nearly blind because of cataracts, Blind in one eye, loss of one eye, Blindness caused by diabetes, Blurred vision, Detached/scarred retina, Hardening of lens, Lens implants in both eyes, Short sighted, long sighted, myopia, Trouble with eyes (nes), eyes not good (nes), Tunnel vision<br>• *Other eye complaints*, including Astigmatism, Buphthalmos, Colour blind, Double vision, Dry eye syndrome, trouble with tear ducts, watery eyes, Eye infection, conjunctivitis, Eyes are light sensitive, Floater in eye, Glaucoma, Haemorrhage behind eye, Injury to eye, Iritis, Keratoconus, Night blindness, Retinitis pigmentosa, Scarred cornea, corneal ulcers, Squint, lazy eye, Sty on eye.

Several other measures are only used in the Web Appendices of the paper. Details of these variables are included below:

**Measure**	**Details**
General health	
General health *(bad / good)*	Every year, respondents were asked, *"How is your health in general? Would you say it was … very good, good, fair, bad, or very bad?"*<br>Two outcome measures are based on this, following standard practice in the HSE reports: bad general health (which includes 'bad' or 'very bad' health) and good general health (which includes 'good' or 'very good' health).
Longstanding illness (LSI)	Every year 1994-2011, respondents were asked *"Do you have any long-standing illness, disability or infirmity? By long-standing I mean anything that has troubled you over a period of time, or that is likely to affect you over a period of time?"* (The response options were 'Yes' and 'No').<br>In 2012 the question was changed to be consistent with the Government's new harmonised disability questions for use in social surveys [1], and is not comparable to the previous version.
Limiting LSI	Every year 1996-2011, respondents who said they had an LSI were than asked, *"Does this illness or disability (do any of these illnesses or disabilities) limit your activities in any way?"* (again allowing only Yes/No answers).<br>In 2012 the question was changed to be consistent with the Government's new harmonised disability questions for use in social surveys (see HSE 2012 report), and is not comparable to the previous version.
Problems with usual activities *(some problems / unable)*	In the self-completion survey in 1996, 2003-6, 2008, 2010-12 and 2014, respondents were asked *'Now we would like to know how your health is today. Please answer ALL the questions. By ticking one box for each question below, please indicate which statements best describe your own health state today'*:<br>- *"I have no problems with performing my usual activities (e.g. work, study, housework,*

For peer review only - http://bmjopen.bmj.com/site/about/guidelines.xhtml

	family or leisure activities)"<br>- *"I have some problems with performing my usual activities"*<br>- *"I am unable to perform my usual activities"*<br>[This is part of the widely-used EQ-5D health status indicator [2]. However, for the purposes of this paper we have separated the individual measures that make up the EQ-5D in order to compare these to similar indicators of morbidity within each domain].<br>Two outcome measures are based on this: whether people have any problems (the 2nd and 3rd categories combined), and whether they are unable to perform their usual activities (3rd category only).
Limitations in past 2wks	Every year, respondents were asked, *"Now I'd like you to think about the two weeks ending yesterday. During those 2 weeks did you have to cut down on any of the things you usually do (about the house or at work or in your free time) because of your answer at <the LSI question> or some other illness or injury?"*<br>There have been two small changes to this question's wording in 1996. Firstly, 'work' was changed to 'work/school'. Secondly, *'your answer at <the LSI question>'* was changed to *'a condition you have just told me about'*. While it is impossible to be sure of the exact effect of these changes, neither seem likely to influence the results (at least for the 25+ age group where fewer individuals are in full-time education).

For peer review only

For peer review only - http://bmjopen.bmj.com/site/about/guidelines.xhtml

Appendix 6: Measures not included in the main paper

Trends in several measures are not included in the main paper, either

Table 10: Trends for measures not included in the main paper

	Start of trend		Trend			
	Period	Incidence	End period	Raw	Adj.[a]	Adj. 95% CI
CVD						
Component measures nec[b]						
Recent heart murmur	1994-96	0.8%	2011-14	0.1%	0.0%	[-0.3, 0.4%]
Recent irregular heart rhythm	1994-96	1.6%	2011-14	0.4%	0.4%	[-0.1, 0.8%]
Recent other heart disease	1994-96	0.2%	2011-14	0.7%	0.7%	[0.4, 0.9%]
Ever had (not just recent)						
Ever had high BP	1994-96	19.0%	2011-14	4.5%	3.7%	[2.3, 5.1%]
DD high BP	1994-96	13.2%	2011-14	6.9%	6.0%	[4.7, 7.3%]
Ever IHD or stroke	1994-96	2.9%	2011-14	0.3%	-0.0%	[-0.6, 0.6%]
DD IHD or stroke	1994-96	2.5%	2011-14	0.5%	0.2%	[-0.3, 0.7%]
Ever had angina	1994-96	1.9%	2011-14	-0.2%	-0.4%	[-0.9, 0.0%]
Ever DD angina	1994-96	1.6%	2011-14	-0.1%	-0.3%	[-0.7, 0.1%]
Ever heart murmur	1994-96	3.2%	2011-14	-0.3%	-0.3%	[-0.9, 0.3%]
DD heart murmur	1994-96	2.6%	2011-14	-0.2%	-0.2%	[-0.7, 0.3%]
Ever irregular heart rhythm	1994-96	6.4%	2011-14	-0.7%	-0.9%	[-1.7, -0.1%]
DD irregular heart rhythm	1994-96	3.5%	2011-14	0.5%	0.3%	[-0.3, 1.0%]
Ever other heart disease	1994-96	0.9%	2011-14	1.1%	1.0%	[0.6, 1.5%]
DD other heart disease	1994-96	0.8%	2011-14	1.0%	1.0%	[0.6, 1.4%]
Respiratory						
**Alternate measures**						
Phlegm symptoms	1994-96	9.1%	2008-10	-1.3%	-1.4%	[-2.3, -0.5%]
LSI Respiratory All	1994-96	7.9%	2011-14	-0.7%	-0.7%	[-1.6, 0.1%]
**Ever had (not just recent)**						
Wheezing Ever	1994-96	32.3%	2008-10	0.0%	-0.1%	[-1.8, 1.5%]
Wheezing Past 12mths	1994-96	18.9%	2008-10	-1.0%	-1.1%	[-2.3, 0.2%]
Diabetes						
**Ever had (not just recent)**						
Ever diabetes	1994-96	2.0%	2011-14	2.9%	2.8%	[2.3, 3.2%]
DD diabetes	1994-96	1.7%	2011-14	2.5%	2.3%	[2.0, 2.7%]
Mental health						
**Alternate measures**						
High psychiatric morbidity	1994-96	3.2%	2011-14	1.0%	0.9%	[0.4, 1.4%]
Activity limitations & musculoskeletal						
**For comparison**						
Walking limitation	1994-96	4.6%	2001-03	1.4%	1.2%	[0.5, 1.9%]
Washing/dressing limitation	1994-96	1.9%	2001-03	0.5%	0.4%	[0.0, 0.8%]
Other LSIs						
LSI Blood Disorders	1994-96	0.3%	2011-14	0.6%	0.5%	[0.3, 0.8%]
LSI Cancer	1994-96	1.0%	2011-14	0.3%	0.3%	[-0.1, 0.6%]

For peer review only - http://bmjopen.bmj.com/site/about/guidelines.xhtml

	Start of trend		Trend			
	Period	Incidence	End period	Raw	Adj.[a]	Adj. 95% CI
LSI D,GUM,E&M	1994-96	6.9%	2011-14	1.1%	0.8%	[0.0, 1.6%]
LSI Epilepsy	1994-96	0.7%	2011-14	0.1%	0.1%	[-0.2, 0.3%]
LSI Nervous System	1994-96	3.7%	2011-14	-0.2%	-0.3%	[-0.8, 0.3%]

[a] 'Adj.' = trend adjusted for changing age and sex distribution of the working-age population. [b] 'nec' = not elsewhere included.

The details of these measures are as follows:

Measure	Details
Circulatory	
Beyond 'recent': 'Ever had' and 'DD' CVD	In the main paper, we look at whether people report recent doctor-diagnosed CVD (looking separately at heart attack/stroke, angina, and any recent CVD). As shown in Web Appendix 2, this comes from three questions: whether people report ever having this condition; whether a doctor diagnosed this; and whether they have had an attack in the past 12 months / consider themselves to still have the condition.<br>This Web Appendix therefore shows trends in the other versions of these measures, i.e. having ever had this type of CVD, and having ever doctor-diagnosed ('DD') CVD of this type.
Component measure: Heart murmur<br>Irregular heart rhythm<br>Other heart disease	In the main paper, we recent reports of doctor-diagnosed angina; heart attack (including myocardial infarction or coronary thrombosis); a heart murmur; abnormal heart rhythm; or other heart trouble (see Web Appendix 2). Angina and heart attack are also analysed in the main paper in their own right; in this Web Appendix, we further show trends separately in heart murmur, abnormal heart rhythm or other heart trouble.
Respiratory	
Component measure: 'phlegm'	In the main paper, we look at whether people report recent COPD (see Web Appendix 2. This combines two measures: regular cough + phlegm. This Web Appendix shows the trend in the phlegm measure on its own, without being combined with a regular cough.
Alternative version: 'LSI respiratory'	In the main paper, we look at whether an asthma LSI (to examine alongside a direct question on diagnosed asthma); see Web Appendix 2. This Web Appendix also shows people reporting a longstanding illness ('LSI') which is included within the broader category of respiratory conditions.<br>The respiratory LSI measure is based on the group labelled *'Asthma'*, *'Bronchitis'*, *'Hayfever'*, or *'Respiratory other'*, which as of 2011 includes:<br>Asthma: Asthma; Bronchial asthma, allergic asthma; and Asthma - allergy to house dust/grass/cat fur. It explicitly excludes cardiac asthma.<br>Hayfever: Hayfever, Allergic rhinitis<br>Bronchitis/emphysema: Bronchitis/emphysema, Bronchiectasis, Chronic bronchitis.<br>Other respiratory complaints: Other respiratory complaints, Abscess on larynx, Adenoid problems, nasal polyps, Allergy to dust/cat fur, Bad chest (nes), weak chest – wheezy, Breathlessness, Bronchial trouble, chest trouble (nes), Catarrh, Chest infections, get a lot of colds, Churg-Strauss syndrome, Chronic Obstructive Pulmonary Disease (COPD), Coughing fits, Croup, Damaged lung (nes), lost lower lobe of left lung, Fibrosis of lung, Furred up airways, collapsed lung, Lung complaint (nes), lung problems (nes), Lung damage by viral pneumonia, Paralysis of vocal cords, Pigeon fancier's lung, Pneumoconiosis, byssinosis, asbestosis and other industrial respiratory disease, Recurrent pleurisy, Rhinitis (nes), Sinus trouble, sinusitis, Sore throat, pharyngitis, Throat infection, Throat trouble (nes), throat irritation, Tonsillitis, Ulcer on lung, fluid

For peer review only - http://bmjopen.bmj.com/site/about/guidelines.xhtml

Measure	Details
	on lung. Note that:<br>• It explicitly <u>excludes</u> TB (pulmonary tuberculosis), Cystic fibrosis, Skin allergy, Food allergy, Allergy (nes), Pilonidal sinus, Sick sinus syndrome, Whooping cough.<br>• If complaint is breathlessness with the cause also stated, this is coded with the cause – hence it also excludes breathlessness as a result of anaemia, breathlessness due to hole in heart, and breathlessness due to angina.
Component measure: Wheezing	In the main paper, we look at whether people report recent wheezing/asthma. As shown in Web Appendix 2, this comes from three questions: whether people report ever having had wheezing or whistling in the chest; whether they have had this in the past 12 months; and whether they have had an asthma attack in the past 12 months.<br>This Web Appendix shows trends in the other versions of these measures, i.e. having ever had wheezing/whistling in the chest, and whether they have had this in the past 1 months.
Beyond 'recent': 'Ever had' and 'DD' diabetes	In the main paper, we look at whether people report recent doctor-diagnosed diabetes As shown in Web Appendix 2, this comes from three questions: whether people report ever having this condition; whether a doctor diagnosed this; and whether they currently inject insulin / take other medication for diabetes.<br>This Web Appendix shows trends in the other versions of these measures, i.e. having ever had diabetes, and having ever doctor-diagnosed ('DD') diabetes.
Activity limitations	
For comparison: Walking limitation	This is based on the personal care disability scale used in the 2001 HSE report [3]. Respondents in 1995, 2000 and 2001 were asked if of the following applied to them (interviewers were instructed to ignore temporary disabilities that are expected to last less than one year): *"Cannot walk 200 yards or more on own without stopping or discomfort"*. People who reported a limitation were asked if they used a walking aid, and if they did, were then asked if they could walk 200 yards without the walking aid.
For comparison: Washing & dressing limitation	This is based on the personal care disability scale used in the 2001 HSE report [3]. Respondents in 1995, 2000 and 2001 were asked if any of the following applied to them (interviewers were instructed to ignore temporary disabilities that are expected to last less than one year):<br>- *"Cannot dress and undress without difficulty"*<br>- *"Cannot wash hands and face without difficulty"*<br>For comparison to the 'problems with washing/dressing today' measure in the main paper (which covers a more extended period and is based on a different question; see Web Appendix 2), a measure is derived if respondents say they report either of these problems.
Other LSIs	
Other LSIs	Every year 1994-2011, people who report a longstanding illness (LSI) are then asked, *'what is the matter with you?'*; up to 6 responses are then coded by the interviewer into a consistent coding frame based on the International Classification of Diseases. The various other LSIs are as follows:<br>• The Blood Disorders LSI measure is based on the group *'Disorders of blood and blood forming organs and immunity disorders'*, which as of 2011 includes: Anaemia, pernicious anaemia, Blood condition (nes), blood deficiency, Haemophilia, Idiopathic Thrombochopenic Purpura (ITP), Immunodeficiences, Polycthaemia (blood thickening), blood to thick, Purpura (nes), Removal of spleen, Sarcoidosis (previously code 37), Sickle cell anaemia/disease, Thalassaemia, Thrombocythenia. It explicitly excludes Leukaemia - code 01.<br>• The Cancer LSI measure is based on the group *'Cancer (neoplasm) including lumps, masses, tumours and growths and benign (non-malignant) lumps and cysts'*, which as of

For peer review only - http://bmjopen.bmj.com/site/about/guidelines.xhtml

Measure	Details
	2011 includes: Acoustic neuroma, After effect of cancer (nes), All tumours, growths, masses, lumps and cysts, whether malignant or benign eg. tumour on brain,, growth in bowel, growth on spinal cord, lump in, breast, Cancers sited in any part of the body or system eg., Lung, breast, stomach, Colostomy caused by cancer, Cyst on eye, cyst in kidney., General arthroma, Hereditary cancer, Hodgkin's disease, Hysterectomy for cancer of womb, Inch. leukaemia (cancer of the blood), Lymphoma, Mastectomy (nes), Neurofibromatosis, Part of intestines removed (cancer), Pituitary gland removed (cancer), Rodent ulcers, Sarcomas, carcinomas, Skin cancer, bone cancer, Wilms tumour<br>• The D,GUM,E&M (Digestive, Genitourinary Medicine, and Endocrine & Metabolic) LSI is based on the groups, *'Complaints of bowel/colon (large intestine,caecum, bowel, colon, rectum)'* (including Colitis, colon trouble, ulcerative colitis, Coleliac, Colostomy (nes), Crohn's disease, Diverticulitis, Enteritis, Faecal incontinence/encopresis., Frequent diarrhoea, constipation, Grumbling appendix, Hirschsprung's disease, Irritable bowel, inflammation of bowel, Polyp on bowel, Spastic colon, but explicitly excluding piles and Cancer of stomach/bowel), *Other digestive complaints (stomach, liver, pancreas, bile ducts, small intestine - duodenum, jejunum and ileum)* (including Cirrhosis of the liver, liver problems, Food allergies, Ileostomy, Indigestion, heart burn, dyspepsia, Inflamed duodenum, Liver disease, biliary artesia, Nervous stomach, acid stomach, Pancreas problems, Stomach trouble (nes), abdominal trouble (nes), Stone in gallbladder, gallbladder problems, Throat trouble - difficulty in swallowing, Weakness in intestines), *Stomach ulcer/ulcer (nes)/abdominal hernia/rupture* (including Double/inguinal/diaphragm/hiatus/umbilical hernia, Gastric/duodenal/peptic ulcer, Hernia (nes), rupture (nes), Ulcer (nes)), *Complaints of teeth/mouth/tongue* (including Cleft palate, hare lip, Impacted wisdom tooth, gingivitis, No sense of taste, Ulcers on tongue, mouth ulcers), *Other endocrine/metabolic* (including Addison's disease, Beckwith - Wiedemann syndrome, Coeliac disease, Cushing's syndrome, Cystic fibrosis, Gilbert's syndrome, Hormone deficiency, deficiency of growth hormone,, dwarfism, Hypercalcemia, Hypopotassaemia, lack of potassium, Malacia, Myxoedema (nes), Obesity/overweight, Phenylketonuria, Rickets, Too much cholesterol in blood, Underactive/overactive thyroid, goitre, Water/fluid retention, Wilson's disease, but explicitly excluding Thyroid trouble and tiredness and Overactive thyroid and swelling in neck, *Other bladder problems/incontinence* (including Bed wetting, enuresis, Bladder restriction, Water trouble (nes), Weak bladder, bladder complaint (nes), but explicitly excluding Prostate trouble), *Kidney complaints* (including Chronic renal failure, Horseshoe kidney, cystic kidney, Kidney trouble, tube damage, stone in the kidney, Nephritis, pyelonephritis, Nephrotic syndrome, Only one kidney, double kidney on right side, Renal TB, Uraemia), *Reproductive system disorders* (including Abscess on breast, mastitis, cracked nipple, Amenorrhea, Damaged testicles, Endometriosis, Gynaecological problems, Hysterectomy (nes), Impotence, infertility, Menopause, Pelvic inflammatory disease/PID (female), Period problems, flooding, pre-menstrual tension/syndrome, Prolapse (nes) if female, Prolapsed womb, Prostrate gland trouble, Turner's syndrome, Vaginitis, vulvitis, dysmenorrhoea) and *Urinary tract infection* (including Cystitis, urine infection).<br>• The Epilepsy LSI is based on the group, *'Epilepsy/fits/convulsion',* including Grand mal, Petit mal, Jacksonian fit, Lennox-Gastaut syndrome, blackouts, febrile convulsions, fit (nes)<br>• The Nervous System LSI is based on the groups:<br>○ *Migraine/headaches*<br>○ *Other problems of nervous system*, including Abscess on brain, Alzheimer's

For peer review only - http://bmjopen.bmj.com/site/about/guidelines.xhtml

Measure	Details
	disease, Bell's palsy, Brain damage resulting from infection (eg. meningitis,, encephalitis) or injury, Carpal tunnel syndrome, Cerebral palsy (spastic), Degenerative brain disease, Fibromyalgia, Friedreich's Ataxia, Guillain-Barre syndrome, Huntington's chorea, Hydrocephalus, microcephaly, fluid on brain, Injury to spine resulting in paralysis, Metachromatic leucodystrophy, Motor neurone disease, Multiple Sclerosis (MS), disseminated sclerosis, Muscular dystrophy, Myalgic encephalomyelitis (ME), Myasthenia gravis, Myotonic dystrophy, Neuralgia, neuritis, Numbness/loss of feeling in fingers, hand, leg etc, Paraplegia (paralysis of lower limbs), Parkinson's disease (paralysis agitans), Partially paralysed (nes), Physically handicapped - spasticity of all limbs, Pins and needles in arm, Post viral syndrome (ME), Removal of nerve in arm, Restless legs, Sciatica, Shingles, Spina bifida, Syringomyelia, Trapped nerve, Trigeminal neuralgia, Teraplegia"<br>○ *Meniere's disease/ear complaints causing balance problems* (including Labryrinthitis,, loss of balance - inner ear, Vertigo).

peer review only

For peer review only - http://bmjopen.bmj.com/site/about/guidelines.xhtml

Appendix 7: Year-by-year trends

This appendix presents the year-by-year trends for all of the variables included in the main paper. The table row labelled 'start v end sig' presents the p-value for testing the null hypothesis that there is no difference between the first and last years in the series (whichever these years are). Note that this will differ from the confidence intervals presented in the main paper as these are grouped into multi-year periods with larger sample sizes and therefore greater precision.

Table 11: Year-to-year trends in cardiovascular health

	High blood pressure LSI	Recent high blood pressure	*Biomarker high blood pressure*	Recent heart attack/stroke	IHD/stroke LSI	Heart attack symptoms	Mini stroke (TIA) symptoms	Recent angina	*Angina symptoms*
**1994**	2.2%	4.2%	8.4%	1.2%	1.4%	5.5%		1.1%	2.3%
**1995**	2.9%		8.3%		1.5%				
**1996**	3.0%		8.3%		1.5%				
**1997**	3.8%		7.7%		1.4%				
**1998**	3.1%	5.4%	7.0%	1.5%	1.3%	6.5%		1.4%	2.2%
**1999**	3.4%				1.4%				
**2000**	4.0%		6.5%		1.3%				
**2001**	4.5%		7.3%		1.7%				
**2002**	4.3%		6.1%		1.4%				
**2003**	4.5%	7.9%	4.9%	1.3%	1.3%	5.5%	8.1%	1.0%	1.8%
**2004**	4.0%				1.2%				
**2005**	5.0%		4.4%		1.3%				
**2006**	4.4%	8.7%	3.9%	1.1%	1.2%	6.2%	7.8%	0.9%	1.6%
**2007**	4.9%		4.5%		1.0%				
**2008**	5.1%		3.9%		1.1%				
**2009**	4.7%		3.2%		1.3%				
**2010**	4.6%		4.1%		1.1%				
**2011**	4.0%	9.5%	3.2%	1.0%	1.0%	5.2%	6.7%	0.7%	1.2%
**2012**			4.1%						
**2013**			3.7%						
**2014**			3.9%						
**Start v end sig.**	0.00	0.00	0.00	0.14	0.05	0.52	0.01	0.03	0.00
**N**	124,830	43,292	79,601	43,445	124,830	43,521	23,487	43,477	43,518

For peer review only - http://bmjopen.bmj.com/site/about/guidelines.xhtml

Table 12: Year-to-year trends in respiratory health

	COPD symptoms	Diagnosed asthma	Asthma LSI	Breathlessness-Grade 2+	Breathlessness-Grade 3	Recent wheezing/asthma	Wheezing stopping sleep
**1994**			4.7%				
**1995**	6.6%	10.8%	4.8%	19.1%	7.6%	19.8%	3.6%
**1996**	6.6%	11.5%	5.3%	20.3%	8.0%	19.3%	3.5%
**1997**		11.9%	6.0%			18.9%	3.7%
**1998**			5.3%				
**1999**			5.7%				
**2000**			5.5%				
**2001**		14.1%	5.9%			19.9%	3.4%
**2002**			6.0%				
**2003**			5.8%				
**2004**			6.3%				
**2005**			6.1%				
**2006**			5.8%				
**2007**			5.7%				
**2008**			6.2%				
**2009**			5.5%				
**2010**	5.1%	16.6%	6.0%	15.4%	6.4%	18.4%	3.2%
**2011**			5.6%				
**2012**							
**2013**							
**2014**							
**Start v end sig.**	0.00	0.00	0.02	0.00	0.01	0.05	0.18
**N**	25,631	41,219	124,830	25,620	25,620	41,218	41,218

For peer review only - http://bmjopen.bmj.com/site/about/guidelines.xhtml

Table 13: Year-to-year trends in activity limitations & musculoskeletal health

	Problems walking about today	Walking limitation	Any locomotor limitation	Problems washing/dressing today	Washing/dressing limitation	Any self-care limitation	Pain-any	Pain-extreme	Arthritis LSI	Other musculoskeletal LSI
**1994**									4.9%	8.9%
**1995**		4.6%	6.8%		1.9%	3.9%			5.4%	9.9%
**1996**	11.5%			3.4%			32.0%	3.0%	5.4%	10.3%
**1997**									6.0%	11.4%
**1998**									5.6%	11.7%
**1999**									5.5%	11.0%
**2000**		6.3%	8.2%		2.5%	5.2%			5.6%	10.7%
**2001**		5.9%	7.8%		2.4%	4.7%			6.1%	10.9%
**2002**									5.7%	12.3%
**2003**	11.8%			3.2%			27.1%	3.2%	6.2%	11.8%
**2004**	11.6%			3.6%			28.6%	3.5%	6.3%	11.6%
**2005**	12.3%			4.0%			27.8%	3.5%	6.0%	11.3%
**2006**	11.6%			3.6%			26.8%	3.1%	5.4%	10.1%
**2007**									5.4%	9.9%
**2008**	11.5%			3.6%			28.1%	3.1%	4.7%	9.5%
**2009**									5.2%	9.0%
**2010**	13.0%			4.1%			29.9%	3.2%	5.1%	10.3%
**2011**	13.6%			4.0%			34.0%	4.0%	4.9%	9.2%
**2012**	11.8%			3.8%			27.4%	3.1%		
**2013**										
**2014**	12.2%			4.2%			27.7%	3.0%		
**Start v end sig.**	0.30	0.00	0.01	0.05	0.04	0.01	0.00	0.89	0.97	0.57
**N**	62,680	25,341	25,341	62,612	25,341	25,341	62,692	62,692	124,830	124,830

For peer review only - http://bmjopen.bmj.com/site/about/guidelines.xhtml

Table 14: Year-to-year trends in obesity & diabetes

	BMI-Underweight	BMI-Obese	High waist-hip ratio	Recent diabetes	Diabetes LSI	Glycated haemoglobin
**1994**	1.1%	15.7%	9.5%	1.2%	1.5%	
**1995**	1.1%	17.0%			1.6%	
**1996**	0.9%	17.9%			1.6%	
**1997**	0.9%	19.3%	12.1%		1.7%	
**1998**	1.0%	19.5%	11.3%	1.4%	1.5%	
**1999**	1.1%	20.1%	16.3%		1.9%	
**2000**	0.9%	21.5%			2.0%	
**2001**	0.9%	22.8%	15.8%		2.1%	
**2002**	1.0%	23.5%	16.5%		2.1%	
**2003**	0.9%	23.2%	18.7%	2.1%	2.4%	2.7%
**2004**	1.0%	24.3%			2.8%	
**2005**	0.8%	24.5%	21.6%		2.9%	
**2006**	0.8%	25.1%	20.7%	2.7%	2.9%	3.1%
**2007**	1.0%	25.3%	22.1%		3.4%	
**2008**	0.9%	25.3%	22.5%		2.9%	3.8%
**2009**	1.4%	24.3%	23.5%	3.4%	3.8%	4.3%
**2010**	1.1%	27.8%	24.3%	3.4%	3.5%	3.7%
**2011**	0.8%	25.4%	24.3%	3.6%	3.8%	5.5%
**2012**	1.1%	25.6%	24.0%	3.6%		4.9%
**2013**	1.0%	26.8%	24.2%	3.6%		4.8%
**2014**	0.8%	27.1%	24.7%	3.7%		4.4%
**Start v end sig.**	1.1%	15.7%	9.5%	1.2%	1.5%	
**N**	1.1%	17.0%			1.6%	

w only

For peer review only - http://bmjopen.bmj.com/site/about/guidelines.xhtml

Table 15: Year-to-year trends in other biomarkers

	High total cholesterol	Low HDL cholesterol	Raised C-reactive protein	Raised fibrinogen	Anaemia	Iron deficiency	Cancer LSI
**1994**	75.7%				6.7%	39.9%	0.2%
**1995**							0.3%
**1996**							0.3%
**1997**							0.4%
**1998**	64.8%	11.8%	21.4%	2.3%	6.3%	38.2%	0.5%
**1999**							0.4%
**2000**							0.5%
**2001**							0.5%
**2002**							0.5%
**2003**	71.4%	4.0%	24.1%	5.7%			0.6%
**2004**							0.6%
**2005**							0.6%
**2006**	67.2%	5.1%	22.7%	5.7%	4.6%	29.3%	0.7%
**2007**							0.5%
**2008**	66.7%	4.3%					0.6%
**2009**	66.9%	4.5%	23.5%	3.8%	5.3%	27.0%	0.5%
**2010**	64.1%	4.6%					0.8%
**2011**	60.2%	4.5%					0.8%
**2012**	64.0%	4.4%					
**2013**	58.0%	3.4%					
**2014**	55.4%	2.9%					
**Start v end sig.**	0.00	0.00	0.11	0.01	0.04	0.00	0.00
**N**	41,224	33,937	17,749	16,105	20,228	20,304	124,830

w only

For peer review only - http://bmjopen.bmj.com/site/about/guidelines.xhtml

Table 16: Year-to-year trends in mental health

	Mental health LSI	Psychiatric morbidity symptoms	Anxiety/depression -moderately	Anxiety/depression -extremely
**1994**	1.8%	16.1%		
**1995**	2.3%	18.0%		
**1996**	2.4%		21.9%	1.8%
**1997**	2.9%	16.5%		
**1998**	3.0%	15.6%		
**1999**	3.0%	17.7%		
**2000**	3.5%	14.4%		
**2001**	3.3%	13.7%		
**2002**	3.1%	16.6%		
**2003**	3.7%	13.5%	18.5%	1.9%
**2004**	3.6%	13.4%	18.8%	2.1%
**2005**	4.4%	14.0%	19.6%	2.1%
**2006**	4.1%	13.9%	18.8%	2.1%
**2007**	4.5%			
**2008**	4.2%	13.7%	18.5%	2.0%
**2009**	4.9%	17.1%		
**2010**	5.2%	16.1%	23.5%	2.7%
**2011**	4.6%		26.8%	3.0%
**2012**		16.0%	20.0%	2.7%
**2013**				
**2014**		15.6%	19.6%	2.5%
**Start v end sig.**	0.00	0.47	0.01	0.02
**N**	124,830	107,834	62,635	62,635

For peer review only - http://bmjopen.bmj.com/site/about/guidelines.xhtml

Appendix 8: Others' health trend analyses using HSE data

Trends in some of these indicators have not previously been analysed (e.g. waist-hip ratio, fibrinogen). However, others have been studied but never integrated into a single picture of changing morbidity; we review these in this section. (For reasons of space these are included here rather than in the main text).

Cardiovascular morbidity

1998-2011 trends in the two biomarkers for total and HDL cholesterol using HSE data are shown in Oyebode,[5] who find similar results.

Respiratory morbidity

A subset of the HSE respiratory indicators (ever/past year wheezing, doctor-diagnosed asthma) were analysed by Hall and Mindell[23] showing trends 2001-2010, showing similar trends. They found stability in some measures (ever wheezing) but improvements in others (past-year wheezing) – at the same time as the reported prevalence of doctor-diagnosed asthma increased.

Obesity & diabetes

While the English trends in waist-hip ratio have not previously been analysed, earlier Scottish trends are given in Hotchkiss et al 2012.[13] Trends in diabetes have been covered in several HSE reports, e.g. Moody 2012,[14] as has BMI (see particularly the paper by Sperrin et al 2014,[24] who also created a publicly-available time-series HSE dataset for this purpose).

Activity limitations, pain & musculoskeletal morbidity

While musculoskeletal LSIs have not previously been analysed in HSE, a decline can also be seen in the General Household Survey.[28]

Mental health

In the UK and most other high-income countries, benefit claims due to mental ill-health have been rising,[29] which has come alongside considerable increases in mental health diagnosis and treatment.[30] The extent to which this reflects rises in mental ill-health and genuinely declining work capacity, however, has long been the subject of debate.[31 32] Perhaps the most robust long-term general population data series in the UK is the Adult Psychiatric Morbidity Survey.[30 33]

While some studies have used HSE to show rises in mental ill-health, others have used the same data to come to the opposite conclusion.[34 35] These contrasting conclusions are explained by Web Appendix 5 below: moderate mental ill-health fell between the mid-1990s and the mid-2000s, before rising in 2009, and with a particularly high prevalence in 2011. The conclusions of studies will therefore depend on the years they use as their start and end periods for the trend analysis.[5] It is also worth noting that our results for considerable increases in mental health LSIs can also be seen in a similar measure in the Labour Force Survey.[36 37]

---

[5] The major explanation why 'moderate anxiety/depression today' does not show a decline 2011-14 compared to 1994-6 is because of a single very high reported prevalence in 2011, which had reduced by 2012 and 2014. The alternate measure ('psychiatric morbidity symptoms') was not asked in 2011.

For peer review only - http://bmjopen.bmj.com/site/about/guidelines.xhtml

Other morbidity measures

While CRP and fibrinogen are collected in HSE at considerable efforts, their trends have rarely been studied (e.g. they appear only in supplementary descriptive tables in Hughes et al [17]). A decline in anaemia using HSE data 1998-2005 has been observed by Tull et al 2009,[38] but this has not hitherto been updated to the 2008-10 period.

It has been suggested that multimorbidity has risen among older people in England [39] and for all age groups in Ontario,[40] although others have cautioned against using simple disease counts,[41] and the evidence cited in the introduction of the main paper suggests that rising chronic disease reporting may partly be a result of increasing awareness (rather than underlying prevalence) of disease.

For peer review only

For peer review only - http://bmjopen.bmj.com/site/about/guidelines.xhtml

For peer review only

Web Appendix page 38

For peer review only - http://bmjopen.bmj.com/site/about/guidelines.xhtml

Appendix 9: Working-age mortality trends

Mortality in general

Given debates about whether historic improvements in life expectancy are being sustained,[50 51] it is important to note that in the period under study in this paper, working-age life expectancy was increasing. This can be seen in data from the Human Mortality Database (May 2016 update) 1993-2013, using one-year age and one-year period. This data shows that increases in mortality are not found for working-age people as a whole in any major country – for example, standardised working-age death rates have declined by 23% in the US and 35% in the UK over 1993-2013.

Cause-specific mortality for the 0-64 population

The main text refers to cause-specific morality in several places, referring to the death rate among 0-64 year olds from cardiovascular disease (CVD), respiratory conditions, diabetes, and liver cirrhosis. These death rates refer to UK deaths within relevant ICD-10 codes (I00-I99 for CVD, J00-J99 for respiratory conditions, E10-E14 for diabetes), standardised to the European standard population, and taken from the World Health Organization European Office's Health for All Database (May 2016 version), http://www.euro.who.int/en/data-and-evidence/databases/european-health-for-all-database-hfa-db.

For peer review only

For peer review only - http://bmjopen.bmj.com/site/about/guidelines.xhtml

Bibliography for Web Appendix

1. ONS. Harmonised Concepts and Questions for Social Data Sources, Primary Principles: Long-lasting Health Conditions and Illnesses; Impairments and Disability [version 1.1]. London: Office for National Statistics (ONS), 2015.
2. Szende A, Janssen B, Cabases J, editors. *Self-Reported Population Health: An International Perspective based on EQ-5D*. Netherlands: Springer, 2014.
3. Erens B, Primatesta P, Prior G. Health survey for England 1999: the health of minority ethnic groups. London: The Stationery Office, 2001.
4. National Heart L, and Blood Institute. Sixth Report of the Joint National Committee on the Prevention, Detection, Evaluation and Treatment of High Blood Pressure. NIH Publication No 98-04080: National Institutes on Health, 1997.
5. Oyebode O. Cardiovascular disease. In: Craig R, Mindell J, eds. Health Survey for England 2011: Volume 1 - Health, social care and lifestyles. Leeds, UK: Health and Social Care Information Centre 2012.
6. Banks J, Marmot M, Oldfield Z, et al. The SES Health Gradient on Both Sides of the Atlantic. NBER Working Paper No 12674, 2006.
7. Lawlor DA, Adamson J, Ebrahim S. Performance of the WHO Rose angina questionnaire in post-menopausal women: Are all of the questions necessary? *Journal of Epidemiology and Community Health* 2003;57(7):538-41. doi: 10.1136/jech.57.7.538
8. Cook D, Shaper A, Macfarlane P. Using the WHO (Rose) Angina Questionnaire in Cardiovascular Epidemiology. *International Journal of Epidemiology* 1989;18(3):607-13. doi: 10.1093/ije/18.3.607
9. Fletcher C, Elmes P, Fairbairn M, et al. The significance of respiratory symptoms and the diagnosis of chronic bronchitis in a working population. *BMJ* 1959;2:257-66.
10. WHO. Physical status: the use and interpretation of anthropometry. Report of a WHO Expert Committee. WHO Technical Report Series 854. Geneva: World Health Organization (WHO), 1995.
11. National Obesity Observatory. Obesity and Overweight Surveillance in England: what is measured and where are the gaps?, 2009.
12. NICE. Obesity: The Prevention, Identification, Assessment and Management of Overweight and Obesity in Adults and Children NICE Clinical Guidelines, No 43. London: Centre for Public Health Excellence and National Collaborating Centre for Primary Care at the National Institute for Health and Clinical Excellence (NICE), 2006.
13. Hotchkiss JW, Davies CA, Gray L, et al. Trends in cardiovascular disease biomarkers and their socioeconomic patterning among adults in the Scottish population 1995 to 2009: cross-sectional surveys. *BMJ Open* 2012;2(3) doi: 10.1136/bmjopen-2011-000771
14. Moody A. Diabetes and hyperglycaemia. In: Craig R, Mindell J, eds. Health Survey for England 2011: Volume 1 - Health, social care and lifestyles. Leeds, UK: Health and Social Care Information Centre 2012.
15. Aresu M, Gordon-Dseagu V, Shelton N. Diabetes and glycaemia. In: Craig R, Hirani V, eds. Health Survey for England 2009, Volume 1: Health and lifestyles. Leeds, UK: The NHS Information Centre for health and social care 2010:59-74.
16. Steptoe A. Psychosocial biomarker research: integrating social, emotional and economic factors into population studies of aging and health. *Social Cognitive and Affective Neuroscience* 2011;6(2):226-33. doi: 10.1093/scan/nsq032
17. Hughes A, McMunn A, Bartley M, et al. Elevated inflammatory biomarkers during unemployment: modification by age and country in the UK. *Journal of Epidemiology and Community Health* 2015;69(7):673-79. doi: 10.1136/jech-2014-204404
18. Benzeval M, Davillas A, Kumari M, et al. Understanding Society: The UK Household Longitudinal Study Biomarker User Guide and Glossary (version 1). Colchester, Essex: Institute for Social and Economic Research, 2014.
19. Chaudhury M. Blood analytes. In: Sproston K, Primatesta P, eds. Health Survey for England, 2003, Vol 2: Risk Factors for Cardiovascular Disease: TSO 2004:241-88.

For peer review only - http://bmjopen.bmj.com/site/about/guidelines.xhtml

20. Erens B, Primatesta P. Health survey for England 1998: cardiovascular disease. *London: The Stationery Office* 1999
21. Chaudhury M, Tull K. Nutrition and haematological status. In: Craig R, Mindell J, eds. Health Survey for England, 2005: The health of older people, Vol 1: General health and function: TSO 2006:67-96.
22. Goldberg D, PA W. User Guide to the General Health Questionnaire. Windsor, UK: NFER-Nelson, 1988.
23. Hall J, Mindell J. Respiratory symptoms and disease in adults In: Craig R, Mindell J, eds. Health Survey for England, 2010, Volume 1: Respiratory health: TSO 2011.
24. Sperrin M, Marshall A, Higgins V, et al. Slowing down of adult body mass index trend increases in England: a latent class analysis of cross-sectional surveys (1992-2010). *International Journal of Obesity* 2014;38(6)
25. McBeth J, Jones K. Epidemiology of chronic musculoskeletal pain. *Best Practice & Research Clinical Rheumatology* 2007;21(3):403-25. doi: 10.1016/j.berh.2007.03.003
26. Harkness EF, Macfarlane GJ, Silman AJ, et al. Is musculoskeletal pain more common now than 40 years ago?: two population-based cross-sectional studies. *Rheumatology* 2005;44(7):890-95. doi: 10.1093/rheumatology/keh599
27. Palmer KT, Walsh K, Bendall H, et al. Back pain in Britain: comparison of two prevalence surveys at an interval of 10 years. *BMJ* 2000;320:1577-8.
28. Parsons S, Ingram M, Clarke-Cornwell AM, et al. A Heavy Burden: The occurrence and impact of musculoskeletal conditions in the United Kingdom today. Manchester: Arthritis Research UK & University of Manchester, 2011.
29. OECD. Fit Mind, Fit Job: From evidence to practice in mental health and work. Paris: OECD, 2015.
30. Spiers N, Qassem T, Bebbington P, et al. Prevalence and treatment of common mental disorders in the English national population, 1993–2007. *The British Journal of Psychiatry* 2016;209(2):150-56. doi: 10.1192/bjp.bp.115.174979
31. Moncrieff J, Pomerleau J. Trends in sickness benefits in Great Britain and the contribution of mental disorders. *Journal of Public Health Medicine* 2000;22:59-67.
32. Stansfeld SA, Woodley-Jones D, Rasul F, et al. Work-related distress in the 1990s - a real increase in ill health? . *Journal of Public Mental Health* 2008;7(1):25-31.
33. McManus S, Bebbington P, Jenkins R, et al., editors. *Mental health and wellbeing in England: Adult Psychiatric Morbidity Survey 2014*. Leeds: NHS Digital, 2016.
34. Maheswaran H, Kupek E, Petrou S. Self-reported health and socio-economic inequalities in England, 1996–2009: Repeated national cross-sectional study. *Social Science & Medicine* 2015;136–137:135-46. doi: http://dx.doi.org/10.1016/j.socscimed.2015.05.026
35. Katikireddi SV, Niedzwiedz CL, Popham F. Trends in population mental health before and after the 2008 recession: a repeat cross-sectional analysis of the 1991–2010 Health Surveys of England. *BMJ Open* 2012;2(5) doi: 10.1136/bmjopen-2012-001790
36. Jones M, Wass V. Understanding changing disability-related employment gaps in Britain 1998–2011. *Work, Employment & Society* 2013;27(6):982-1003. doi: 10.1177/0950017013475372
37. Barr B, Kinderman P, Whitehead M. Trends in mental health inequalities in England during a period of recession, austerity and welfare reform 2004 to 2013. *Social Science & Medicine* 2015;147:324-31. doi: http://dx.doi.org/10.1016/j.socscimed.2015.11.009
38. Tull KI, Hirani V, Ali A, et al. Impact of different diagnostic thresholds and the anaemia–ferritin–transferrin receptor model on the prevalence of anaemia and impaired iron status in older people. *Age and Ageing* 2009;38(5):609-13. doi: 10.1093/ageing/afp102
39. Dhalwani NN, O'Donovan G, Zaccardi F, et al. Long terms trends of multimorbidity and association with physical activity in older English population. *International Journal of Behavioral Nutrition and Physical Activity* 2016;13(1):8. doi: 10.1186/s12966-016-0330-9
40. Koné Pefoyo AJ, Bronskill SE, Gruneir A, et al. The increasing burden and complexity of multimorbidity. *BMC Public Health* 2015;15(1):415. doi: 10.1186/s12889-015-1733-2

For peer review only - http://bmjopen.bmj.com/site/about/guidelines.xhtml

41. Tetzlaff J, Junius-Walker U, Muschik D, et al. Identifying time trends in multimorbidity—defining multimorbidity in times of changing diagnostic practices. *Journal of Public Health* 2016:1-8. doi: 10.1007/s10389-016-0771-2
42. Marfeo EE, Haley SM, Jette AM, et al. Conceptual Foundation for Measures of Physical Function and Behavioral Health Function for Social Security Work Disability Evaluation. *Archives of Physical Medicine and Rehabilitation* 2013;94(9):1645-52.e2. doi: 10.1016/j.apmr.2013.03.015
43. Salomon JA, Vos T, Hogan DR, et al. Common values in assessing health outcomes from disease and injury: disability weights measurement study for the Global Burden of Disease Study 2010. *The Lancet* 2012;380(9859):2129-43. doi: http://dx.doi.org/10.1016/S0140-6736(12)61680-8
44. Murray CJL, Richards MA, Newton JN, et al. UK health performance: findings of the Global Burden of Disease Study 2010. *The Lancet* 2013;381(9871):997-1020. doi: https://doi.org/10.1016/S0140-6736(13)60355-4
45. WHO. Global Health Estimates 2015: Disease burden by Cause, Age, Sex, by Country and by Region, 2000-2015. Geneva: World Health Organization (WHO), 2016.
46. WHO. World report on disability. Geneva: World Health Organization (WHO) 2011.
47. Soldo BJ, Mitchell OS, Tfaily R, et al. Cross-cohort differences in health on the verge of retirement. NBER Working Paper No 12762: National Bureau of Economic Research, 2006.
48. Stewart ST, Cutler DM, Rosen AB. Comparison of Trends in U.S. Health-Related Quality of Life over the 2000's Using the SF-6D, HALex, EQ-5D, and EQ-5D Visual Analog Scale versus a Broader Set of Symptoms and Impairments. *Medical care* 2014;52(12):1010-16. doi: 10.1097/MLR.0000000000000181
49. Diederichs CP, Wellmann J, Bartels DB, et al. How to weight chronic diseases in multimorbidity indices? Development of a new method on the basis of individual data from five population-based studies. *Journal of Clinical Epidemiology* 2012;65(6):679-85. doi: 10.1016/j.jclinepi.2011.11.006
50. Case A, Deaton A. Rising morbidity and mortality in midlife among white non-Hispanic Americans in the 21st century. *Proceedings of the National Academy of Sciences* 2015;112(49):15078-83. doi: 10.1073/pnas.1518393112
51. Hiam L, Dorling D, Harrison D, et al. Why has mortality in England and Wales been increasing? An iterative demographic analysis. *Journal of the Royal Society of Medicine* 2017;110(4):153-62. doi: 10.1177/0141076817693599

For peer review only

For peer review only - http://bmjopen.bmj.com/site/about/guidelines.xhtml

BMJ Open

Has working-age morbidity been declining? Changes over time in general health, chronic diseases, symptoms and biomarkers in England 1994-2014

Journal:	*BMJ Open*
Manuscript ID	bmjopen-2019-032378.R1
Article Type:	Original research
Date Submitted by the Author:	13-Sep-2019
Complete List of Authors:	Geiger, Ben; University of Kent, School of Social Policy, Sociology and Social Research (SSPSSR);
**Primary Subject Heading**:	Public health
Secondary Subject Heading:	Research methods
Keywords:	Morbidity, Population Health, Biomarkers, Cross-sectional studies, Trends, England

SCHOLARONE™
Manuscripts

BMJ

I, the Submitting Author has the right to grant and does grant on behalf of all authors of the Work (as defined in the below author licence), an exclusive licence and/or a non-exclusive licence for contributions from authors who are: i) UK Crown employees; ii) where BMJ has agreed a CC-BY licence shall apply, and/or iii) in accordance with the terms applicable for US Federal Government officers or employees acting as part of their official duties; on a worldwide, perpetual, irrevocable, royalty-free basis to BMJ Publishing Group Ltd ("BMJ") its licensees and where the relevant Journal is co-owned by BMJ to the co-owners of the Journal, to publish the Work in this journal and any other BMJ products and to exploit all rights, as set out in our licence.

The Submitting Author accepts and understands that any supply made under these terms is made by BMJ to the Submitting Author unless you are acting as an employee on behalf of your employer or a postgraduate student of an affiliated institution which is paying any applicable article publishing charge ("APC") for Open Access articles. Where the Submitting Author wishes to make the Work available on an Open Access basis (and intends to pay the relevant APC), the terms of reuse of such Open Access shall be governed by a Creative Commons licence – details of these licences and which Creative Commons licence will apply to this Work are set out in our licence referred to above.

Other than as permitted in any relevant BMJ Author's Self Archiving Policies, I confirm this Work has not been accepted for publication elsewhere, is not being considered for publication elsewhere and does not duplicate material already published. I confirm all authors consent to publication of this Work and authorise the granting of this licence.

For peer review only

Has working-age morbidity been declining? Changes over time in general health, chronic diseases, symptoms and biomarkers in England 1994-2014

Authors:

Dr Ben B. Geiger, University of Kent, UK

Postal address: Ben B Geiger, School of Social Policy, Sociology and Social Research (SSPSSR), University of Kent, Canterbury, Kent, CT2 7NZ, UK

Email: b.b.geiger@kent.ac.uk

Word count *(exc. title page, abstract, tables, acknowledgements, contributions & references)*

3,913 words + 5 tables

MeSH Keywords

Morbidity; Population Health; Biomarkers; Cross-Sectional Studies; Trends; England

Acknowledgements

This work was supported by the Economic and Social Research Council (grant number ES/K009583/1).

Author statement

BBG was responsible for the design, data preparation, analysis and reporting of the study.

For peer review only

Acknowledgements

Many thanks to Clare Bambra for comments, and to Mariska van der Horst for research assistance; neither should be held responsible for the analysis or interpretation of the paper itself.

Funding

This work was supported by the Economic and Social Research Council (ESRC), grant number ES/K009583/1.

Competing interests

The author has worked on secondment at the UK Department for Work and Pensions (DWP) in 2015-16.

Data sharing

The Health Survey for England 1994-2014 are available for free to registered users at the UK Data Service - see https://beta.ukdataservice.ac.uk/datacatalogue/series/series?id=2000021#!/abstract. There are no conditions for re-use for non-commercial applications of the data.

The statistical code enabling replication using publicly available data is available from OSF (Morbidity in England 1994-2014 2019, available from: http://osf.io/dy6sv) and www.benbgeiger.co.uk.

For peer review only

Has working-age morbidity been declining? Changes over time in general health, chronic diseases, symptoms and biomarkers in England 1994-2014

Abstract:

Objectives: As life expectancy has increased in high-income countries, there has been a global debate about whether additional years of life are free from ill-health/disability. However, little attention has been given to changes over time in morbidity in the *working-age* population, particularly outside the US, despite its importance for health monitoring and social policy. This study therefore asks: what are the changes over time in working-age morbidity in England over two decades?

Design, setting and participants: We use a high-quality annual cross-sectional survey, the Health Survey for England ('HSE') 1994-2014. HSE uses a random sample of the English household population, with a combined sample size of over 140,000 people. We produce a newly-harmonised version of HSE that maximises comparability over time, including new non-response weights. While HSE is used for monitoring population health, it has hitherto not used for investigating morbidity as a whole.

Outcome measures: We analyse all 39 measures that are fully comparable over time – including chronic disease diagnoses, symptomatology and a number of biomarkers – adjusting for gender and age.

Results: We find a mixed picture: we see improving cardiovascular and respiratory health, but deteriorations in obesity, diabetes, some biomarkers, and feelings of extreme anxiety/depression, alongside stability in moderate mental ill-health and

For peer review only - http://bmjopen.bmj.com/site/about/guidelines.xhtml

musculoskeletal-related health. In several domains we also see stable or rising chronic disease *diagnoses* even where *symptomatology* has declined. While data limitations make it challenging to combine these measures into a single morbidity index, there is little systematic trend for declining morbidity to be seen in the measures that predict self-reported health most strongly.

Conclusions: Despite considerable falls in working-age mortality – and the assumptions of many policymakers that morbidity will follow mortality – there is no systematic improvement in overall working-age morbidity in England from 1994 to 2014.

For peer review only

Strengths and limitations of this study

- We provide a robust analysis of changes over time in morbidity in England for 39 measures across two decades using the Health Survey for England ('HSE').
- We include every morbidity measure for which consistent comparisons over time can be constructed in the HSE.
- We take care to maximise comparability over time, including constructing new non-response weights.
- However, response rates for each stage of the HSE have declined over time, and it is impossible to rule out changing non-response biases.
- There are also several dimensions of morbidity for which there is little trend data in HSE.

For peer review only - http://bmjopen.bmj.com/site/about/guidelines.xhtml

INTRODUCTION

As life expectancy has increased in high-income countries, there has been a global debate about whether additional years of life are free from ill-health/disability. It is now largely accepted that old-age disability has declined in the US (albeit varying by age/method),[1 2] although chronic illness increased,[3] and the picture beyond the US is more mixed.[4-6] Yet this research agenda has not been matched by similar attention to changes over time in morbidity in the *working-age* population. In the absence of direct evidence, policymakers have often made claims about population morbidity trends based on self-reports of general health,[6-8] which we know are unreliable.[9 10] The lack of evidence is even more problematic in the case of social security, where many policymakers have assumed that working-age morbidity *must* have improved in recent decades given improvements in mortality (despite the potential for declining mortality to coexist with rising morbidity)[6] – and that therefore high/rising levels of claims are not 'genuine'.[11 12]

Almost the only direct evidence on changes over time in working-age morbidity in high-income countries comes from the US. Contrary to policymaker expectations, these studies have generally found *deteriorating* morbidity since the mid-1990s, particularly activities of daily living (ADLs) and physical functioning.[13-16] Other studies have focused on the older working-age population with similar results.[2 17] Again, not all measures show deteriorations, and not all studies come to identical conclusions,[18] but there is little sign of any improvement in morbidity among working-age Americans – despite a 23% fall in working-age mortality 1993-2013 (Web Appendix 1). Outside of the US, there is a paucity of evidence, but from the limited evidence that exists, there is again little sign of improving morbidity.[19-21]

For peer review only - http://bmjopen.bmj.com/site/about/guidelines.xhtml

This study therefore asks: what are the changes over time in working-age morbidity in England over two decades? We answer this question using the Health Survey for England (HSE), a high quality Government survey with a combined sample of 140,000 individuals. This analysis makes two contributions. Firstly, we provide one of the few systematic analyses of changes over time in working-age morbidity in any high-income country outside the US. Secondly, we supplement self-report measures with 10 'biomarkers', which are particularly valuable for showing genuine changes over time (rather than merely changes in how people describe their health), but which have rarely been examined alongside self-reported working-age morbidity trends (Martin et al. 2010[22] being an exception).

DATA AND METHODS

This section follows the STROBE cross sectional reporting guidelines.[23]

Data source

Robust evidence of change over time requires a consistently-collected, high-quality data source. We use the HSE, an annual government-sponsored cross-sectional survey of 3,000-11,000 adults with no proxy responses.[24] A particular advantage is that the initial interview is followed by a nurse visit, which in selected years has also included a blood sample. Nevertheless, analysing change in HSE is more complex than it might appear:

- Firstly, HSE was run by the Government Office of Population Censuses and Surveys in 1991-3, before changing to NatCen in 1994. We focus on 1994-2014 data given evidence of a discontinuity at this point across multiple variables.

For peer review only - http://bmjopen.bmj.com/site/about/guidelines.xhtml

- Secondly, the coverage of HSE varies year-to-year, and moreover there are multiple changes in question wording/filtering. Based on a systematic search of HSE questions, we have included every morbidity measure that is comparable over a significant duration. Even for measures that have been previously been analysed (e.g. BMI[25]), this new analysis uncovered further discontinuities (Web Appendices 2 & 3).
- Third, HSE excludes those in communal establishments. While a smaller problem for the working-age population than older ages,[2] we minimise the impact of rising university attendance by focussing on those aged 25+ (Web Appendix 3). The upper limit of the working-age population is set to 59 (women) and 64 (men) to match state pension ages at the start of the period.
- Fourth, HSE supplies non-response weights from 2003. However, there had been a substantial decline in response rates prior to the introduction of weights, particularly for blood samples (from 53.3% 1994 to 39.9% 2003; Web Appendix 3). We therefore reduce non-response biases by creating new non-response weights, as described in Web Appendix 3.

The resulting sample sizes for the various stages of data collection are shown in Web Appendix 3. Our dataset substantially extends an existing HSE time-series dataset (UK Data Archive SN7025); the code enabling other researchers to assemble this extended time-series dataset are freely available.[26]

Patient involvement

As this is a health monitoring (rather than intervention) study using all available secondary data, patients were not directly involved. However, from previous

For peer review only - http://bmjopen.bmj.com/site/about/guidelines.xhtml

discussions we are aware that the study will be of interest to patient/disability advocacy groups, who will receive jargon-free summaries of the research.

Measures

We cannot interpret changes over time correctly without understanding different ways of operationalising 'morbidity'.[1] General health/disability measures – e.g. *"How is your health in general?"* – are the best conceptual match to our research question, and clearly do capture something meaningful in practice.[27] However, their generality means they suffer from what is variably conceptualised as 'response shift'[28] or 'differential item functioning'[29]; that is, for any given question, different people (or even the same people at different times) report their general health/disability on different scales. Numerous factors contribute to this, ranging from the experience of ill-health itself[28] to non-health factors such as social security incentives,[30] gendered- and age-related expectations, and medicalisation.[31]

These inconsistent response scales mean that general health/disability measures are inadequate for answering our question: trends in such measures can differ wildly between different surveys covering nominally the same concept and population, e.g. for disability in England[9] or self-rated health in the US.[10] Indeed, the HSE itself shows that England has experienced deteriorating 'bad general health' at the same time as activity limitations have fallen (interested readers can see changes over time in seven general HSE health/disability measures in Web Appendix 4). To robustly answer our research question, we must instead focus on more *specific* morbidity measures that are likely to be interpreted more consistently over time and place. This is similar to the recommendation of the Washington Group on Disability Statistics – a UN agency founded in 2001 – that cross-country disability comparisons

For peer review only - http://bmjopen.bmj.com/site/about/guidelines.xhtml

should be based on multiple measures of specific activity limitations, rather than single questions about general participation restrictions.[32]

Our systematic search found 39 specific morbidity measures that are comparable over time: these are summarised in Table 1, with further details in Web Appendix 5. (A further 29 measures are also included in Web Appendix 6; this includes 8 sub-components of measures in the main text, 16 reports of ever having a condition even if this not recent, and 5 other categories of LSI). These specific morbidity measures can be grouped into three types, which have different strengths and weaknesses with respect to our question:

1. *Medical labels:* some measures are based on medical labels, either diagnosed chronic diseases or self-reported types of longstanding illness. (Those reporting a longstanding illness were asked, *'what is the matter with you?'*; up to 6 responses were then coded by the interviewer based on the International Classification of Diseases). These are imperfect measures of morbidity[33] as they partly reflect healthcare systems and medicalisation more broadly, both of which change over time. Nevertheless, they are an important element of morbidity as they have real consequences via increasing awareness/labelling of people's experiences.
2. *Symptom-based:* some measures are based on self-reports of ill-health symptoms or specific domains of activity limitations. These measures are either single items (e.g. pain, anxiety/depression) or validated symptom scales (e.g. the Rose angina scale, GHQ psychiatric distress). While there is no guarantee that a given question will be interpreted identically over time,[34 35] they seem substantially less likely to be affected by changing medical practice

For peer review only - http://bmjopen.bmj.com/site/about/guidelines.xhtml

than label-based measures, and are therefore likely to be more comparable over time.

3. *Biomarkers* – that is, objective measures of biological or physiological measures – offer a considerable strength in analysing changes over time, as they largely avoiding reporting biases that are likely to vary between socioeconomic groups and over time.[36] They do this at the price of an indirect and sometimes still-debated relationship to morbidity (see Web Appendix 5), and do not cover several important morbidity domains (e.g. we lack good biomarkers for mental distress, pain and fatigue).

These three types of measures are therefore complementary in understanding changing morbidity: biomarkers are most reliable in measuring changes over time, but do not capture morbidity well; symptom-based measures capture morbidity well and are reasonably (if still imperfectly) reliable; and label-based measures are flawed in capturing symptoms/limitations but do enable us to capture whether people consider themselves to have a medical condition.

For peer review only

For peer review only - http://bmjopen.bmj.com/site/about/guidelines.xhtml

Table 1: HSE morbidity measures

Category	Measure	Type[a]	Operationalisation (years available)
Cardio-vascular disease (CVD)	High blood pressure LSI[b]	L	Hypertension reported as longstanding illness (LSI) (1994-2011)
	Recent high blood pressure	L	Still has (or on medication for) doctor-diagnosed hypertension (1994-2013)
	Biomarker high blood pressure	B	Systolic BP >=140mmHg & diastolic BP >=90mmHg (1994-2013)
	High total cholesterol	B	Total cholesterol >= 5mmol/L (1994-2012)
	Low HDL cholesterol	B	High density lipoprotein (HDL) cholesterol <=1 mmol/L (1998-2013)
	Recent heart attack /stroke	L	Doctor-diagnosed heart attack or stroke in past 12mths (1994-2011)
	Recent angina	L	Doctor-diagnosed angina in past 12mths (1994-2011)
	Ischaemic heart/stroke LSI[b]	L	Stroke, heart attack or angina reported as longstanding illness (LSI) (1994-2011)
	Heart attack symptoms	S	Ever had severe pain across chest for ½hr (1994-2011)
	Mini stroke (TIA) symptoms	S	Attack of weakness/slurred speech/blurred vision in past 12mths (2003-11)
	Angina symptoms	S	Rose Angina scale definition of angina symptoms (1994-2011)
	Any recent CVD	L	Doctor-diagnosed heart condition (exc. hypertension) in past 12mths (1994-2011)
	Any CVD LSI	L	Any CVD reported as longstanding illness (LSI) (1994-2011)
Respiratory	COPD symptoms	S	Regular cough & phlegm for at least 3mths each year (1995-2010)
	Lifetime diagnosed asthma	L	Ever had doctor-diagnosed asthma (1995-2010)
	Asthma LSI[b]	L	Asthma reported as longstanding illness (LSI) (1994-2011)
	Breathlessness-grade 2	S	Short of breath when hurrying up walking uphill (1995-2010)
	Breathlessness-grade 3	S	Short of breath when walking on level ground (1995-2010)
	Recent wheezing/asthma	S	Wheezing, whistling in chest or asthma attack in past 12mths (1995-2010)
	Wheezing stopping sleep	S	Woken 1+ times/wk by wheezing/whistling in chest in last 12mths (1994-2010)
Obesity & diabetes	BMI-underweight	B	Body Mass Index (BMI) <=18.5kg/m^2 (1994-2013)
	BMI-obese	B	Body Mass Index (BMI) >= 30kg/m^2 (1994-2013)
	High waist-hip ratio	B	Waist-hip ratio of >1 for men and >0.85 for women (1994-2013)
	Recent diabetes	L	Currently taking medication for doctor-diagnosed diabetes (1994-2013)
	Diabetes LSI[b]	L	Diabetes reported as longstanding illness (LSI) (1994-2011)
	High glycated haemoglobin	B	HbA_{1C} >=48mmol/mol (2003-2013)
Mental Health	Mental health LSI[b]	L	Mental health reported as longstanding illness (LSI) (1994-2011)
	Psychiatric distress (GHQ)	S	4+ negative symptoms from 12-item General Health Questionnaire (1994-2014)
	Anxiety/depression-moderately	S	At least moderately anxious/depressed today (1996-2014)
	Anxiety/depression-extremely	S	Extremely anxious/depressed today (1996-2014)
Activity limitations	Problems walking today	S	Has at least some problems walking about today (1996-2014)
	Locomotor limitation	S	Can't walk far / bend down / go up or down stairs without resting (1996-2001)

For peer review only - http://bmjopen.bmj.com/site/about/guidelines.xhtml

Category	Measure	Type[a]	Operationalisation (years available)
& musculo-skeletal	Problems washing/dressing today	S	Has at least some problems washing/dressing today (1996-2014)
	Self-care limitation	S	Difficulty with one of six everyday activities (e.g. feeding, dressing) (1995-2001)
	Pain-any	S	Has at least some pain or discomfort today (1996-2014)
	Pain-extreme	S	Has extreme pain or discomfort today (1996-2014)
	Arthritis LSI[b]	L	Arthritis or rheumatism reported as longstanding illness (LSI) (1994-2011)
	Other musculoskeletal LSI[b]	L	Other musculoskeletal condition reported as longstanding illness (LSI) (1994-2011)
Sensory & Communication	LSI Eye or Ear	L	Eye or ear condition reported as longstanding illness (LSI) (1994-2011)
	Hearing limitation	S	Cannot follow TV programme at volume others find acceptable (1995-2001)
	Seeing limitation	S	Cannot see well enough to recognise friend across the road (1995-2001)
	Communicating limitation	S	Have problem communicating with other people (1995-2001)
Other Biomarkers	Raised C-reactive protein	B	CRP >3mg/L (1998-2009)
	Raised fibrinogen	B	Fibrinogen >4mg/L (1998-2009)
	Anaemia	B	Haemoglobin <13g/dL for men and <12g/dL for women (1994-2009)
	Iron deficiency	B	Serum ferritin < 45ng/ml (1994-2009)

See Web Appendix 5 for full details on all measures .[a] Measure type key: L=medical label; S=symptom-based; B=biomarker. [b] Particular causes of longstanding illness (LSI) come from the open question, 'what is the matter with you?' Up to 6 responses are then coded by the interviewer into a coding frame based on ICD.

For peer review only

ANALYSIS

In the first instance we look at unadjusted changes over time in each morbidity indicator, showing the actual levels of morbidity found in the population. However, we primarily focus on changes after adjustment for sex and age (following others[37 38]), akin to standardising for the age-sex composition of the population. Given that our aim is to *describe* changes rather than to explain them, we do not further adjust for potential causal influences on morbidity that are likely to vary over the period, such as employment over economic cycles. This is a task for future research, but we should note that such analysis is possible using our publicly-available time-series dataset that includes *inter alia* employment status, education and region.

We chose to examine discrete changes from the start to the end of available data for each measure, rather than using linear or non-linear trend terms. Given our aims of informing policy debates, this has three advantages: a discrete change is simple to interpret; it is compatible with the different start/end years available for different measures; and it does not require any assumptions about the functional form of trends (linear trends are particularly unlikely given the role of non-linear economic cycles). Individual survey years are grouped into 3-4 year periods to increase sample size and precision, but single-year prevalence is given in Web Appendix 7. Given our binary outcome measures, we use logistic regression models with the following form:

$$y_i = \text{logit}\,[\,\boldsymbol{\beta}_1\mathbf{period_i} + \boldsymbol{\beta}_2\mathbf{age_i} + \beta_3 male_i + \boldsymbol{\beta}_4(\mathbf{age_i} * \text{male}_\text{i})\,]$$

For peer review only - http://bmjopen.bmj.com/site/about/guidelines.xhtml

…where $\mathbf{period_i}$ refers to a vector of period dummy variables (covering all periods in which there were any observations: 1994-96, 1997-2000, 2001-03, 2004-07, 2008-10 and 2011-14), $\boldsymbol{\beta_1}$ is a vector of our primary outcome coefficients showing change between each period and the earliest available period, $\mathbf{age_i}$ refers to a vector of age dummy variables, $\mathrm{male_i}$ refers to a binary gender dummy variable, and $\boldsymbol{\beta_2}$, β_3 and $\boldsymbol{\beta_4}$ refer to the coefficients on age, gender and their interaction respectively. We present average marginal effects rather than odds ratios, partly because these are simple to understand – odds ratios have no easy real-world interpretation for policymakers – but primarily because odds ratios are not fully comparable across different models, and cannot therefore underpin our comparison of changes over time between indicators.[39]

To avoid a binary cut-off of statistical significance,[40] 95% confidence intervals are used to convey precision. All analyses use weights, exclude boost samples that use different sampling methods, and adjust for the multistage clustered sample design and the stratification of the sample across survey years using the SVYSET command in Stata (although standard errors will be slightly underestimated as it is not possible to consistently adjust for sample stratification within years). For reasons of space, we are unable to discuss previous HSE studies of aspects of morbidity in the main text; these are instead described in Web Appendix 8.

For peer review only - http://bmjopen.bmj.com/site/about/guidelines.xhtml

RESULTS

Conditions with sharply declining mortality

We start by focussing on cardiovascular disease (CVD) and respiratory illness, which have both seen large falls in mortality (by >50% and >25% respectively among 0-64 year-olds 1994-2013; Web Appendix 1). Changes over time in *morbidity*, however, are shown in Table 2.

For

Table 2: Changes over time in cardiovascular and respiratory morbidity

	Starting period		Change from start to end period			
	Period	Prevalence	End period	Raw change	Adj.[a] change	Adj. change 95% CI
**Blood pressure/cholesterol**						
High blood pressure LSI	1994-96	2.7%	2011-14	1.3%	1.0%	[0.4, 1.6%]
Recent high blood pressure	1994-96	4.2%	2011-14	5.2%	4.8%	[3.9, 5.6%]
Biomarker high BP	1994-96	8.4%	2011-14	-4.7%	-5.0%	[-5.6, -4.5%]
High total cholesterol	1994-96	75.7%	2011-14	-16.4%	-17.6%	[-19.1, -16.1%]
Low HDL cholesterol	1997-2000	11.8%	2011-14	-8.0%	-8.0%	[-9.0, -7.1%]
**Other CVD**						
Recent heart attack/stroke	1994-96	1.2%	2011-14	-0.3%	-0.4%	[-0.7, 0.0%]
Recent angina	1994-96	1.1%	2011-14	-0.4%	-0.5%	[-0.8, -0.1%]
IHD/stroke LSI	1994-96	1.4%	2011-14	-0.4%	-0.6%	[-0.9, -0.2%]
Heart attack symptoms	1994-96	5.5%	2011-14	-0.3%	-0.5%	[-1.3, 0.3%]
Mini stroke (TIA) symptoms	2001-03	8.1%	2011-14	-1.4%	-1.4%	[-2.4, -0.4%]
Angina symptoms	1994-96	2.3%	2011-14	-1.1%	-1.2%	[-1.6, -0.7%]
Any CVD LSI	1994-96	5.8%	2011-14	1.1%	0.6%	[-0.1, 1.4%]
Any recent CVD	1994-96	3.1%	2011-14	0.7%	0.5%	[-0.1, 1.2%]
**Respiratory**						
Lifetime diagnosed asthma	1994-96	11.2%	2008-10	5.5%	5.7%	[4.5, 6.8%]
Asthma LSI	1994-96	5.0%	2011-14	0.7%	0.7%	[0.0, 1.4%]
Breathlessness-Grade 2+	1994-96	19.7%	2008-10	-4.4%	-4.8%	[-6.1, -3.5%]
Breathlessness-Grade 3	1994-96	7.8%	2008-10	-1.4%	-1.6%	[-2.5, -0.8%]
Recent wheezing/asthma	1994-96	19.5%	2008-10	-1.2%	-1.2%	[-2.5, 0.1%]
Wheezing stopping sleep	1994-96	3.6%	2008-10	-0.4%	-0.5%	[-1.0, 0.1%]
COPD symptoms	1994-96	6.6%	2008-10	-1.5%	-1.6%	[-2.3, -0.8%]

[a] 'Adj.' = adjusted for changing age and sex distribution of the working-age population.

Looking first at high blood pressure, biomarker-measured high blood pressure has halved over two decades (similar improvements are found for the biomarkers for total and HDL cholesterol). Yet when we look at self-reports (either people reporting this

For peer review only - http://bmjopen.bmj.com/site/about/guidelines.xhtml

as an LSI, or in response to a direct question about having recent diagnosed high blood pressure), we see large *rises* over time. There has been an increasing diagnosis of high blood pressure and increasing prescriptions of blood pressure-lowering drugs; these may have helped reduce the underlying incidence of high blood pressure while simultaneously raising people's awareness of morbidity.

Table 2 further shows declines in several key types of CVD (heart attack, mini-stroke, angina), whether measured through people's reports of the disease itself or their reports of its symptoms. Nevertheless, the morbidity declines (8-50%) are often not on the scale of the declines in mortality (>50%); this is likely to be because mortality declines are partly driven by improved treatment,[41] which means each incident CVD case is likely to last longer.[42 43] More surprisingly, the measures of 'any reported CVD' show no improvement (with some, uncertain signs of *rises*). Looking at its sub-components (Web Appendix 6), this seems to be due to possible increases in diagnosed irregular heart rhythm and other heart trouble.

Finally, Table 2 shows that symptoms-based measures of respiratory morbidity have improved, particularly COPD symptoms (regular cough & phlegm) and breathlessness (at both levels), and more uncertainly for recent wheezing/asthma and wheezing stopping sleep. Again, though, diagnosis-related measures of asthma – reported diagnoses, or self-reports of having asthma as a longstanding illness – have risen, even while underlying symptomatology is improving.

Overall, Table 2 illustrates how changes over time in morbidity do not necessarily follow changes in mortality. There are definite improvements in CVD risk factors and respiratory symptomatology on the scale of improvements in mortality. But the

For peer review only - http://bmjopen.bmj.com/site/about/guidelines.xhtml

prevalence of self-reported CVD conditions such as heart attacks have only declined by a smaller amount, and recent doctor-diagnosed hypertension, any CVD, and asthma diagnoses have either stayed stable or risen.

Conditions with claims of increasing prevalence

The previous section focussed on conditions where there may be an *a priori* expectation that morbidity has improved (given declining mortality); in this section, we focus on three areas where there have been widespread claims of increasing prevalence – obesity, diabetes, and mental health.

Looking at

For peer review only - http://bmjopen.bmj.com/site/about/guidelines.xhtml

Table 3, we do indeed confirm a large rise in obesity in HSE (an 8.0-9.7% rise from an obesity prevalence of 16.9% in 1994-96). The rise in high waist-hip ratios – sometimes suggested to be a better measure of potential morbidity [44] – is even larger. This has come alongside little change in the prevalence of being *underweight* over this period.

For peer review only

For peer review only - http://bmjopen.bmj.com/site/about/guidelines.xhtml

Table 3: Changes over time in obesity, diabetes and mental health

	Starting period		Change from start to end period			
	Period	Prevalence	End period	Raw change	Adj.[a] change	Adj. change 95% CI
**Underweight/Obesity**						
BMI-Underweight	1994-96	1.0%	2011-14	-0.1%	-0.1%	[-0.3, 0.1%]
BMI-Obese	1994-96	16.9%	2011-14	9.3%	8.9%	[8.0, 9.7%]
High waist-hip ratio	1994-96	9.5%	2011-14	14.8%	14.1%	[13.0, 15.2%]
**Diabetes**						
Recent diabetes	1994-96	1.2%	2011-14	2.4%	2.2%	[1.9, 2.6%]
Diabetes LSI	1994-96	1.5%	2011-14	2.3%	2.1%	[1.5, 2.6%]
Glycated haemoglobin	2001-03	2.7%	2011-14	2.1%	2.1%	[1.4, 2.7%]
**Mental health**						
Mental health LSI	1994-96	2.1%	2011-14	2.5%	2.4%	[1.8, 3.0%]
Psychological distress	1994-96	17.1%	2011-14	-1.3%	-1.3%	[-2.4, -0.3%]
Anx./depression-moderate	1994-96	21.9%	2011-14	0.3%	0.1%	[-1.1, 1.3%]
Anx./depression-extremely	1994-96	1.8%	2011-14	1.0%	0.9%	[0.5, 1.3%]

[a] 'Adj.' = adjusted for changing age and sex distribution of the working-age population. 'Anx./depression'= Feeling of anxiety/depression today – see Table 1.

For peer review only

For peer review only - http://bmjopen.bmj.com/site/about/guidelines.xhtml

Table 3 also confirms a large rise in diabetes. This can be seen whether diabetes is measured through people reporting diabetes as an LSI, a specific question about people currently taking medication for diabetes, or via a diabetes biomarker (glycated haemoglobin). It is worth noting that this clear rise in diabetes has occurred despite a *decline* in the age 0-64 death rate from diabetes, by more than one-third 1994-2013 (Web Appendix 1) – indeed, rising prevalence is *because of* falling mortality [45] – again demonstrating the difference between changes in mortality and morbidity.

Trends in mental health are more contentious in the wider literature (see Web Appendix 8), and the measures in HSE are not as strong as in the more occasional Adult Psychiatric Morbidity Surveys. Nevertheless, HSE offers a unique annual perspective on self-reported mental health. As we might expect from increasing treatment/diagnosis, we see a doubling in people reporting a mental health LSI. However, the symptoms-based measures show a more mixed picture:

- Neither of the measures that capture more moderate mental ill-health show rising ill-health (these are psychological distress symptoms and people reporting a feeling of anxiety/depression today, both with a relatively common prevalence of 15-25%). If we break this down by year (see Web Appendix 7), we can see moderate mental ill-health symptoms fell between the mid-1990s and the mid-2000s, before rising in 2009.
- In contrast, the single measure capturing a feeling of extreme anxiety/depression today does show rising morbidity. To see if there were similar signs of rising mental ill-health at extremes in our other measure (psychological distress), we looked at a much higher GHQ threshold of 10

For peer review only - http://bmjopen.bmj.com/site/about/guidelines.xhtml

negative responses out of the 12 GHQ questions (compared to the conventional GHQ threshold of 4). Unlike the conventional GHQ measure, this also showed an increase over time (95% CI of a 0.4 to 1.4% rise; see Web Appendix 6). We should note however that the GHQ is not designed to capture *severe* psychological distress in this way.

Overall, while labelling of mental health conditions has undoubtedly risen, trends in mental health symptoms vary across measures. If we interpret higher GHQ thresholds as indicating more serious psychological distress, then we can see a consistent picture: moderate mental ill-health symptoms fell from the mid-1990s to the mid-2000s before rising around the time of the 2008 economic crisis (as we would expect[46]), whereas more extreme mental ill-health has more consistently risen.

For peer review only

Activity limitations, musculoskeletal and pain

Pain/musculoskeletal conditions are a major component of working-age morbidity, yet very few previous studies show changes over time in symptomatology, and even those that exist [47] sometimes have debatable comparability.[48]

Table 4 shows a fall in some – but not all – HSE measures focussed on pain and musculoskeletal morbidity. Arthritis as a longstanding illness (LSI) has declined (the precision of the estimates is greater when looking at 2008-10 rather than 2011-14, and shows a decline of 0.3-1.2%). There are some (similarly uncertain) signs that other musculoskeletal LSIs have also fallen, and noticeably fewer people say that they have any pain/discomfort today, although there has been no change in people saying they have extreme pain/discomfort. The echoes a previous study that found different trends in low back pain of different levels of severity.[49]

Similarly, there has been a rise in all four activity limitations measures in HSE – although the increases are sometimes uncertain, and are smaller after adjusting for changes in age/sex structure. Moreover, the timing of the rises differ between the measures: the trend in limitations lasting at least a year shows a rise 1994-6 to 2001-3, but the two measures of 'limitations today' do not, instead showing a possible slight rise in the more recent period (see Web Appendix 7; this difference remains if we focus on the sub-components of year-long limitations that more closely match to the 'limitations today' questions, see Web Appendix 6). Still, the measures can collectively be seen as offering some, albeit relatively weak, evidence for an increase in activity limitations.

For peer review only - http://bmjopen.bmj.com/site/about/guidelines.xhtml

Table 4: Changes over time in activity limitations, pain & musculoskeletal morbidity

	Starting period		Change from start to end period			
	Period	Prevalence	End period	Raw change	Adj.[a] change	Adj. change 95% CI
**Activity limitations**						
Problems walking about	1994-96	11.5%	2011-14	1.0%	0.4%	[-0.6, 1.3%]
Any locomotor limitation	1994-96	6.8%	2001-03	1.1%	0.9%	[0.1, 1.7%]
Probs. washing/dressing	1994-96	3.4%	2011-14	0.6%	0.3%	[-0.2, 0.9%]
Any self-care limitation	1994-96	3.9%	2001-03	0.8%	0.7%	[0.1, 1.3%]
**Musculoskeletal/pain**						
Pain-any	1994-96	32.0%	2011-14	-2.2%	-3.3%	[-4.6, -2.0%]
Pain-extreme	1994-96	3.0%	2011-14	0.4%	0.2%	[-0.3, 0.7%]
Arthritis LSI	1994-96	5.3%	2011-14	-0.3%	-0.7%	[-1.4, 0.0%]
Other musculoskeletal LSI	1994-96	9.7%	2011-14	-0.5%	-0.8%	[-1.7, 0.1%]

[a] 'Adj.' = adjusted for changing age and sex distribution of the working-age population.

Other measures

Changes over time in other measures (for which we have no clear *a priori* expectations) are shown in Table 5 below. This includes four biomarkers that are more difficult to compare directly to self-reports:

- Changes over time are available for two biomarkers of inflammation (C-reactive protein ('CRP') and fibrinogen). These are associated with a number of conditions including heart disease, diabetes, cancer [50] and – in the case of CRP – even depression.[51] Table 5 shows that both biomarkers have rising morbidity from 1997-2000 to 2008-10 (although for CRP, the confidence interval is wide and there is a non-negligible possibility that the change is negative).
- The two other biomarkers available in HSE are clearly focussed on anaemia and iron deficiency. Table 5 shows that both of these have declined, with particularly clear evidence for a decline in iron deficiency.

For peer review only - http://bmjopen.bmj.com/site/about/guidelines.xhtml

Table 5: Changes over time in other morbidity measures

	Starting period		Change from start to end period			
	Period	Prevalence	End period	Raw change	Adj.[a] change	Adj. change 95% CI
**Other biomarkers**						
Raised C-reactive protein	1997-2000	21.4%	2008-10	2.1%	1.9%	[-0.7, 4.5%]
Raised fibrinogen	1997-2000	2.3%	2008-10	1.6%	1.5%	[0.3, 2.6%]
Anaemia	1994-96	6.7%	2008-10	-1.4%	-1.4%	[-2.7, -0.1%]
Iron deficiency	1994-96	39.9%	2008-10	-12.9%	-12.5%	[-14.8, -10.2%]
**Sensory & communication**						
LSI Eye or Ear	1994-96	2.8%	2011-14	-0.9%	-1.0%	[-1.5, -0.6%]
Hearing limitation	1994-96	4.3%	2001-03	-1.5%	-1.6%	[-2.1, -1.0%]
Seeing limitation	1994-96	1.4%	2001-03	-0.2%	-0.2%	[-0.6, 0.1%]
Communicating limitation	1994-96	1.0%	2001-03	0.1%	0.1%	[-0.2, 0.4%]

[a] 'Adj.' = adjusted for changing age and sex distribution of the working-age population.

Table 5 also shows changes over time in sensory and communication-related morbidity. This shows a fall in eye/ear conditions (1994-6 to 2011-14) as well as hearing limitations in the earlier period (1994-6 to 2001-03), but no change in people having difficulty communicating with others.

DISCUSSION

Despite considerable evidence on morbidity trends among older people, there are few published studies on changes in morbidity among the working-age population, particularly outside the USA. In this paper, we have analysed changes over time in working-age morbidity in England 1994-2014 using a high-quality repeated cross-sectional study. We see improvements in cardiovascular morbidity, respiratory morbidity and anaemia, but deteriorating obesity, diabetes, some biomarkers (fibrinogen and possibly also CRP) and feelings of extreme anxiety/depression. We see little systematic change over time in more common mental ill-health or musculoskeletal conditions, pain/mobility, and self-care limitations. We should also stress that symptomatology and chronic disease diagnoses often go in different

For peer review only

directions – chronic disease diagnoses have sometimes stayed stable or even risen at the same time that underlying symptomatology has declined (such as for mental health conditions, asthma, hypertension, and CVD as a whole), mirroring findings at older ages.[3]

Our analysis has several strengths. We include every morbidity measure for which consistent changes over time can be constructed, including chronic disease, functioning and symptomatology, and biomarkers. We use a single survey series collected by a single survey organisation; exclude under-25s for whom comparability of survey coverage is unlikely; and construct new non-response weights. Nevertheless, we must note three limitations. Firstly, response rates for each stage of the HSE have declined over time (see Web Appendix 3), and while we create new non-response weights covering the entire period, it is still possible that socioeconomically disadvantaged people (within any age-sex-region group) have become less likely to respond, and as they tend to be in worse health, this could mask deteriorating morbidity. It is impossible to rule out changing non-response biases, but there is no sign that this has occurred; for example, trends in education are similar in HSE and the gold-standard measure of qualification trends, the Labour Force Survey.[9] Secondly, even if non-response biases have not changed, it is possible that people respond differently over time even to identical questions. Third, there are several dimensions of morbidity for which there is little comparable data in HSE. This includes several areas in which morbidity among the working-age population seems to be rising, including *inter alia* cognitive complaints,[52] allergic disorders,[53] and liver cirrhosis (see Web Appendix 1), as well as some areas in which morbidity seems likely to have fallen, such as chronic kidney disease.[54]

For peer review only - http://bmjopen.bmj.com/site/about/guidelines.xhtml

For policymakers, this leaves the question of whether working-age morbidity as a whole is likely to have been getting better or worse in England (at least for those who believe that health states can be put on a unidimensional scale). While it is not possible to create a single morbidity index here, Web Appendix 9 shows the association of each measure with bad general self-rated health (net of age, gender and education). This shows little systematic trend for falling morbidity to be seen in the measures that predict health the most (indeed, the evidence weakly points in the other direction, towards rising morbidity). Certainly there is no evidence that working-age morbidity as a whole has declined over the past twenty years in England despite falling mortality. This mirrors both evidence from the Global Burden of Disease study for the UK (see Web Appendix 9), and more detailed analyses available for the US.[13-16]

In conclusion, despite considerable falls in working-age mortality and gains in life expectancy – and the ensuing expectations of social security policymakers for improving morbidity – there is no evidence of systematic improvement in overall working-age morbidity in England from 1994 to 2014. However, two pieces of further research could strengthen this evidence base. Firstly, the ideal measures for analysing changes in morbidity are functional limitations measures, which are included in the HSE from 1996. However, these were last asked to the working-age population in 2001, and it is a priority to repeat these measures in future years of HSE. Secondly, there is a surprising paucity of studies looking at the changing morbidity of the working-age population outside the US. Given their importance in public debate – particularly in discussions of retirement ages and disability benefits – we hope that other authors will repeat and extend our analyses here, including

For peer review only - http://bmjopen.bmj.com/site/about/guidelines.xhtml

disaggregating these changes across different regions and sociodemographic groups.

For peer review only

For peer review only - http://bmjopen.bmj.com/site/about/guidelines.xhtml

REFERENCES

1. Freedman VA, Crimmins E, Schoeni RF, et al. Resolving inconsistencies in trends in old-age disability: report from a technical working group. *Demography* 2004;41(3):417-41.
2. Freedman VA, Spillman BC, Andreski PM, et al. Trends in Late-Life Activity Limitations in the United States: An Update From Five National Surveys. *Demography* 2013;50(2):661-71. doi: 10.1007/s13524-012-0167-z
3. Freedman VA, Schoeni RF, Martin LG, et al. Chronic Conditions and the Decline in Late-Life Disability. *Demography* 2007;44(3):459-77.
4. Lafortune G, Balestat G. Trends in severe disability among elderly people. Paris: OECD, 2007.
5. Bowling A. Commentary: Trends in activity limitation. *International Journal of Epidemiology* 2011;40(4):1068-70. doi: 10.1093/ije/dyr069
6. Jagger C. Trends in life expectancy and healthy life expectancy. Future of an ageing population: evidence review. London: Foresight, Government Office for Science, 2015.
7. Office for Budget Responsibility. Welfare trends report: October 2016. Cm 9341. London: Her Majesty's Stationery Office, 2016.
8. Department for Work and Pensions, Department of Health. Improving Lives: The Work, Health and Disability Green Paper. Cm 9342. London: Her Majesty's Stationery Office, 2016.
9. Baumberg B, Jones M, Wass V. Disability prevalence and disability-related employment gaps in the UK 1998–2012: Different trends in different surveys? *Social Science & Medicine* 2015;141:72-81. doi: http://dx.doi.org/10.1016/j.socscimed.2015.07.012
10. Salomon JA, Nordhagen S, Oza S, et al. Aer Americans feeling less healthy? The puzzle of trends in self-rated health. *American Journal of Epidemiology* 2009;170(3):343-51.
11. OECD. Sickness, Disability and Work: Breaking the Barriers, Vol 2: Australia, Luxembourg, Spain and the United Kingdom Paris: OECD, 2008.
12. Wise DA, editor. *Social Security Programs and Retirement Around the World: The Capacity to Work at Older Ages*. Chicago: University of Chicago Press for the National Bureau of Economic Research (NBER), 2017.
13. Martin LG, Freedman VA, Schoeni RF, et al. Trends In Disability And Related Chronic Conditions Among People Ages Fifty To Sixty-Four. *Health Affairs* 2010;29(4):725-31. doi: 10.1377/hlthaff.2008.0746
14. Bhattacharya J, Choudhry K, Lakdawalla D. Chronic Disease and Severe Disability among Working-Age Populations. *Medical Care* 2008;46(1):92-100.
15. Martin LG, Schoeni RF. Trends in disability and related chronic conditions among the forty-and-over population: 1997-2010. *Disability and Health Journal* 2014;7(1):S4-S14. doi: 10.1016/j.dhjo.2013.06.007
16. Lakdawalla DN, Bhattacharya J, Goldman DP. Are the young becoming more disabled? *Health affairs* 2004;23(1):168-76.
17. Seeman TE, Merkin SS, Crimmins EM, et al. Disability Trends Among Older Americans: National Health and Nutrition Examination Surveys, 1988–1994 and 1999–2004. *American Journal of Public Health* 2010;100(1):100-07. doi: 10.2105/AJPH.2008.157388
18. Weir D. Are baby boomers living well longer. In: Madrian B, Mitchell OS, Soldo BJ, eds. Redefining retirement: How will boomers fare. Oxford: Oxford UP 2007:95-111.
19. van Oostrom SH, Gijsen R, Stirbu I, et al. Time Trends in Prevalence of Chronic Diseases and Multimorbidity Not Only due to Aging: Data from General Practices and Health Surveys. *PLoS ONE* 2016;11(8):e0160264. doi: 10.1371/journal.pone.0160264

For peer review only - http://bmjopen.bmj.com/site/about/guidelines.xhtml

20. Solé-Auró A, Alcañiz M. Are we living longer but less healthy? Trends in mortality and morbidity in Catalonia (Spain), 1994–2011. *European Journal of Ageing* 2015;12(1):61-70. doi: 10.1007/s10433-014-0317-9
21. Audureau E, Rican S, Coste J. Worsening trends and increasing disparities in health-related quality of life: evidence from two French population-based cross-sectional surveys, 1995–2003. *Quality of Life Research* 2013;22(1):13-26. doi: 10.1007/s11136-012-0117-7
22. Martin LG, Schoeni RF, Andreski PM. Trends in health of older adults in the United States: past, present, future. *Demography* 2010;47(Suppl):S17-S40.
23. von Elm E, Altman D, Egger M, et al. The Strengthening the Reporting of Observational Studies in Epidemiology (STROBE) Statement: guidelines for reporting observational studies. *Annals of Internal Medicine* 2007;147:573-77.
24. Mindell J, Biddulph JP, Hirani V, et al. Cohort Profile: The Health Survey for England. *International Journal of Epidemiology* 2012;41(6):1585-93. doi: 10.1093/ije/dyr199
25. Sperrin M, Marshall A, Higgins V, et al. Slowing down of adult body mass index trend increases in England: a latent class analysis of cross-sectional surveys (1992-2010). *International Journal of Obesity* 2014;38(6)
26. Geiger BB. Morbidity in England 1994-2014 2019 [Available from:http://osf.io/dy6sv].
27. Quesnel Vallee A. Self-rated health: caught in the crossfire of the quest for 'true' health? *International Journal of Epidemiology* 2007;36:1161-64.
28. Sprangers MA, Schwartz CE. Integrating response shift into health-related quality of life research: a theoretical model *Social Science & Medicine* 1999;48(11):1507-151.
29. Teresi JA, Fleishman JA. Differential item functioning and health assessment. *Quality of Life Research* 2007;16(1):33-42. doi: 10.1007/s11136-007-9184-6
30. O'Brien R. Disability and the Worlds of Welfare Capitalism. *Sociological Science* 2015;2(1):1-19. doi: 10.15195/v2.a1
31. OECD. Sick on the Job? Myths and Realities about Mental Health and Work. Paris: OECD, 2012.
32. Altman BM, editor. *International Measurement of Disability: Purpose, Method and Application*: Springer, 2016.
33. Cieza A, Oberhauser C, Bickenbach J, et al. The English are healthier than the Americans: really? *International Journal of Epidemiology* 2015:229-38. doi: 10.1093/ije/dyu182
34. Chan KS, Kasper JD, Brandt J, et al. Measurement Equivalence in ADL and IADL Difficulty Across International Surveys of Aging: Findings From the HRS, SHARE, and ELSA. *The Journals of Gerontology Series B: Psychological Sciences and Social Sciences* 2012;67B(1):121-32. doi: 10.1093/geronb/gbr133
35. Bago d'Uva T, O'Donnell O, van Doorslaer E. Differential health reporting by education level and its impact on the measurement of health inequalities among older Europeans. *International Journal of Epidemiology* 2008;37:1375-83. doi: 10.1093/ije/dyn146
36. Benzeval M, Kumari M, Jones AM. How Do Biomarkers and Genetics Contribute to Understanding Society? *Health Economics* 2016;25(10):1219-22. doi: 10.1002/hec.3400
37. Martin LG, Freedman VA, Schoeni RF, et al. Health and Functioning Among Baby Boomers Approaching 60. *The Journals of Gerontology Series B: Psychological Sciences and Social Sciences* 2009;64B(3):369-77. doi: 10.1093/geronb/gbn040
38. Kaye HS. Disability Rates For Working-Age Adults And For The Elderly Have Stabilized, But Trends For Each Mean Different Results For Costs. *Health Affairs* 2013;32(1):127-34. doi: 10.1377/hlthaff.2012.0533
39. Mood C. Logistic Regression: Why We Cannot Do What We Think We Can Do, and What We Can Do About It. *European Sociological Review* 2010;26(1):67-82.
40. Wasserstein RL, Lazar NA. The ASA's Statement on p-Values: Context, Process, and Purpose. *The American Statistician* 2016;70(2):129-33. doi: 10.1080/00031305.2016.1154108

For peer review only - http://bmjopen.bmj.com/site/about/guidelines.xhtml

41. Unal B, Critchley JA, Capewell S. Explaining the Decline in Coronary Heart Disease Mortality in England and Wales Between 1981 and 2000. *Circulation* 2004;109(9):1101-07. doi: 10.1161/01.cir.0000118498.35499.b2
42. Davies AR, Smeeth L, Grundy E. Contribution of changes in incidence and mortality to trends in the prevalence of coronary heart disease in the UK: 1996–2005. *European Heart Journal* 2007;28(17):2142-47.
43. Oyebode O. Cardiovascular disease. In: Craig R, Mindell J, eds. Health Survey for England, 2011, Volume 1: Health, social care and lifestyles: TSO 2012:21-62.
44. Davillas A, Benzeval M. Alternative measures to BMI: Exploring income-related inequalities in adiposity in Great Britain. *Social Science & Medicine* 2016;166:223-32. doi: http://dx.doi.org/10.1016/j.socscimed.2016.08.032
45. Read SH, Kerssens JJ, McAllister DA, et al. Trends in type 2 diabetes incidence and mortality in Scotland between 2004 and 2013. *Diabetologia* 2016;59(10):2106-13. doi: 10.1007/s00125-016-4054-9
46. Gili M, Roca M, Basu S, et al. The mental health risks of economic crisis in Spain: evidence from primary care centres, 2006 and 2010. *European Journal of Public Health* 2012;23(1):103-08. doi: 10.1093/eurpub/cks035
47. McBeth J, Jones K. Epidemiology of chronic musculoskeletal pain. *Best Practice & Research Clinical Rheumatology* 2007;21(3):403-25. doi: 10.1016/j.berh.2007.03.003
48. Harkness EF, Macfarlane GJ, Silman AJ, et al. Is musculoskeletal pain more common now than 40 years ago?: two population-based cross-sectional studies. *Rheumatology* 2005;44(7):890-95. doi: 10.1093/rheumatology/keh599
49. Palmer KT, Walsh K, Bendall H, et al. Back pain in Britain: comparison of two prevalence surveys at an interval of 10 years. *BMJ* 2000;320:1577-8.
50. Chaudhury M. Blood analytes. In: Sproston K, Primatesta P, eds. Health Survey for England, 2003, Vol 2: Risk Factors for Cardiovascular Disease: TSO 2004:241-88.
51. Steptoe A. Psychosocial biomarker research: integrating social, emotional and economic factors into population studies of aging and health. *Social Cognitive and Affective Neuroscience* 2011;6(2):226-33. doi: 10.1093/scan/nsq032
52. Begum A, Dewey M, Hassiotis A, et al. Subjective cognitive complaints across the adult life span: a 14-year analysis of trends and associations using the 1993, 2000 and 2007 English Psychiatric Morbidity Surveys. *Psychological Medicine* 2014;44(9):1977-87. doi: 10.1017/S0033291713002444
53. Gupta R, Sheikh A, Strachan DP, et al. Time trends in allergic disorders in the UK. *Thorax* 2007;62(1):91-96. doi: 10.1136/thx.2004.038844
54. Aitken GR, Roderick PJ, Fraser S, et al. Change in prevalence of chronic kidney disease in England over time: comparison of nationally representative cross-sectional surveys from 2003 to 2010. *BMJ Open* 2014;4(9) doi: 10.1136/bmjopen-2014-005480
55. Case A, Deaton A. Rising morbidity and mortality in midlife among white non-Hispanic Americans in the 21st century. *Proceedings of the National Academy of Sciences* 2015;112(49):15078-83. doi: 10.1073/pnas.1518393112
56. Hiam L, Dorling D, Harrison D, et al. Why has mortality in England and Wales been increasing? An iterative demographic analysis. *Journal of the Royal Society of Medicine* 2017;110(4):153-62. doi: 10.1177/0141076817693599
57. Department of Health. Our Health and Wellbeing Today. London: HM Government, 2010.
58. Szende A, Janssen B, Cabases J, editors. *Self-Reported Population Health: An International Perspective based on EQ-5D*. Netherlands: Springer, 2014.
59. Erens B, Primatesta P, Prior G. Health survey for England 1999: the health of minority ethnic groups. London: The Stationery Office, 2001.
60. National Heart Lung and Blood Institute. Sixth Report of the Joint National Committee on the Prevention, Detection, Evaluation and Treatment of High Blood Pressure. NIH Publication No 98-04080: National Institutes on Health, 1997.

For peer review only - http://bmjopen.bmj.com/site/about/guidelines.xhtml

61. Oyebode O. Cardiovascular disease. In: Craig R, Mindell J, eds. Health Survey for England 2011: Volume 1 - Health, social care and lifestyles. Leeds, UK: Health and Social Care Information Centre 2012.
62. Banks J, Marmot M, Oldfield Z, et al. The SES Health Gradient on Both Sides of the Atlantic. NBER Working Paper No 12674, 2006.
63. Lawlor DA, Adamson J, Ebrahim S. Performance of the WHO Rose angina questionnaire in post-menopausal women: Are all of the questions necessary? *Journal of Epidemiology and Community Health* 2003;57(7):538-41. doi: 10.1136/jech.57.7.538
64. Cook D, Shaper A, Macfarlane P. Using the WHO (Rose) Angina Questionnaire in Cardiovascular Epidemiology. *International Journal of Epidemiology* 1989;18(3):607-13. doi: 10.1093/ije/18.3.607
65. Fletcher C, Elmes P, Fairbairn M, et al. The significance of respiratory symptoms and the diagnosis of chronic bronchitis in a working population. *BMJ* 1959;2:257-66.
66. WHO. Physical status: the use and interpretation of anthropometry. Report of a WHO Expert Committee. WHO Technical Report Series 854. Geneva: World Health Organization (WHO), 1995.
67. National Obesity Observatory. Obesity and Overweight Surveillance in England: what is measured and where are the gaps?, 2009.
68. NICE. Obesity: The Prevention, Identification, Assessment and Management of Overweight and Obesity in Adults and Children NICE Clinical Guidelines, No 43. London: Centre for Public Health Excellence and National Collaborating Centre for Primary Care at the National Institute for Health and Clinical Excellence (NICE), 2006.
69. Hotchkiss JW, Davies CA, Gray L, et al. Trends in cardiovascular disease biomarkers and their socioeconomic patterning among adults in the Scottish population 1995 to 2009: cross-sectional surveys. *BMJ Open* 2012;2(3) doi: 10.1136/bmjopen-2011-000771
70. Moody A. Diabetes and hyperglycaemia. In: Craig R, Mindell J, eds. Health Survey for England 2011: Volume 1 - Health, social care and lifestyles. Leeds, UK: Health and Social Care Information Centre 2012.
71. Aresu M, Gordon-Dseagu V, Shelton N. Diabetes and glycaemia. In: Craig R, Hirani V, eds. Health Survey for England 2009, Volume 1: Health and lifestyles. Leeds, UK: The NHS Information Centre for health and social care 2010:59-74.
72. Hughes A, McMunn A, Bartley M, et al. Elevated inflammatory biomarkers during unemployment: modification by age and country in the UK. *Journal of Epidemiology and Community Health* 2015;69(7):673-79. doi: 10.1136/jech-2014-204404
73. Benzeval M, Davillas A, Kumari M, et al. Understanding Society: The UK Household Longitudinal Study Biomarker User Guide and Glossary (version 1). Colchester, Essex: Institute for Social and Economic Research, 2014.
74. Erens B, Primatesta P. Health survey for England 1998: cardiovascular disease. *London: The Stationery Office* 1999
75. Chaudhury M, Tull K. Nutrition and haematological status. In: Craig R, Mindell J, eds. Health Survey for England, 2005: The health of older people, Vol 1: General health and function: TSO 2006:67-96.
76. Goldberg D, PA W. User Guide to the General Health Questionnaire. Windsor, UK: NFER-Nelson, 1988.
77. Stochl J, Böhnke JR, Pickett KE, et al. An evaluation of computerized adaptive testing for general psychological distress: combining GHQ-12 and Affectometer-2 in an item bank for public mental health research. *BMC Medical Research Methodology* 2016;16(1):58. doi: 10.1186/s12874-016-0158-7
78. ONS. Harmonised Concepts and Questions for Social Data Sources, Primary Principles: Long-lasting Health Conditions and Illnesses; Impairments and Disability [version 1.1]. London: Office for National Statistics (ONS), 2015.
79. Hall J, Mindell J. Respiratory symptoms and disease in adults In: Craig R, Mindell J, eds. Health Survey for England, 2010, Volume 1: Respiratory health: TSO 2011.

For peer review only - http://bmjopen.bmj.com/site/about/guidelines.xhtml

80. Parsons S, Ingram M, Clarke-Cornwell AM, et al. A Heavy Burden: The occurrence and impact of musculoskeletal conditions in the United Kingdom today. Manchester: Arthritis Research UK & University of Manchester, 2011.
81. OECD. Fit Mind, Fit Job: From evidence to practice in mental health and work. Paris: OECD, 2015.
82. Spiers N, Qassem T, Bebbington P, et al. Prevalence and treatment of common mental disorders in the English national population, 1993–2007. *The British Journal of Psychiatry* 2016;209(2):150-56. doi: 10.1192/bjp.bp.115.174979
83. Moncrieff J, Pomerleau J. Trends in sickness benefits in Great Britain and the contribution of mental disorders. *Journal of Public Health Medicine* 2000;22:59-67.
84. Stansfeld SA, Woodley-Jones D, Rasul F, et al. Work-related distress in the 1990s - a real increase in ill health? . *Journal of Public Mental Health* 2008;7(1):25-31.
85. McManus S, Bebbington P, Jenkins R, et al., editors. *Mental health and wellbeing in England: Adult Psychiatric Morbidity Survey 2014*. Leeds: NHS Digital, 2016.
86. Maheswaran H, Kupek E, Petrou S. Self-reported health and socio-economic inequalities in England, 1996–2009: Repeated national cross-sectional study. *Social Science & Medicine* 2015;136–137:135-46. doi: http://dx.doi.org/10.1016/j.socscimed.2015.05.026
87. Katikireddi SV, Niedzwiedz CL, Popham F. Trends in population mental health before and after the 2008 recession: a repeat cross-sectional analysis of the 1991–2010 Health Surveys of England. *BMJ Open* 2012;2(5) doi: 10.1136/bmjopen-2012-001790
88. Jones M, Wass V. Understanding changing disability-related employment gaps in Britain 1998–2011. *Work, Employment & Society* 2013;27(6):982-1003. doi: 10.1177/0950017013475372
89. Barr B, Kinderman P, Whitehead M. Trends in mental health inequalities in England during a period of recession, austerity and welfare reform 2004 to 2013. *Social Science & Medicine* 2015;147:324-31. doi: http://dx.doi.org/10.1016/j.socscimed.2015.11.009
90. Tull KI, Hirani V, Ali A, et al. Impact of different diagnostic thresholds and the anaemia–ferritin–transferrin receptor model on the prevalence of anaemia and impaired iron status in older people. *Age and Ageing* 2009;38(5):609-13. doi: 10.1093/ageing/afp102
91. Dhalwani NN, O'Donovan G, Zaccardi F, et al. Long terms trends of multimorbidity and association with physical activity in older English population. *International Journal of Behavioral Nutrition and Physical Activity* 2016;13(1):8. doi: 10.1186/s12966-016-0330-9
92. Koné Pefoyo AJ, Bronskill SE, Gruneir A, et al. The increasing burden and complexity of multimorbidity. *BMC Public Health* 2015;15(1):415. doi: 10.1186/s12889-015-1733-2
93. Tetzlaff J, Junius-Walker U, Muschik D, et al. Identifying time trends in multimorbidity—defining multimorbidity in times of changing diagnostic practices. *Journal of Public Health* 2016:1-8. doi: 10.1007/s10389-016-0771-2
94. Marfeo EE, Haley SM, Jette AM, et al. Conceptual Foundation for Measures of Physical Function and Behavioral Health Function for Social Security Work Disability Evaluation. *Archives of Physical Medicine and Rehabilitation* 2013;94(9):1645-52.e2. doi: 10.1016/j.apmr.2013.03.015
95. Murray CJL, Richards MA, Newton JN, et al. UK health performance: findings of the Global Burden of Disease Study 2010. *The Lancet* 2013;381(9871):997-1020. doi: https://doi.org/10.1016/S0140-6736(13)60355-4
96. WHO. Global Health Estimates 2015: Disease burden by Cause, Age, Sex, by Country and by Region, 2000-2015. Geneva: World Health Organization (WHO), 2016.
97. Salomon JA, Vos T, Hogan DR, et al. Common values in assessing health outcomes from disease and injury: disability weights measurement study for the Global Burden of Disease Study 2010. *The Lancet* 2012;380(9859):2129-43. doi: http://dx.doi.org/10.1016/S0140-6736(12)61680-8

For peer review only - http://bmjopen.bmj.com/site/about/guidelines.xhtml

98. WHO. World report on disability. Geneva: World Health Organization (WHO) 2011.
99. Soldo BJ, Mitchell OS, Tfaily R, et al. Cross-cohort differences in health on the verge of retirement. NBER Working Paper No 12762: National Bureau of Economic Research, 2006.
100. Stewart ST, Cutler DM, Rosen AB. Comparison of Trends in U.S. Health-Related Quality of Life over the 2000's Using the SF-6D, HALex, EQ-5D, and EQ-5D Visual Analog Scale versus a Broader Set of Symptoms and Impairments. *Medical care* 2014;52(12):1010-16. doi: 10.1097/MLR.0000000000000181
101. Diederichs CP, Wellmann J, Bartels DB, et al. How to weight chronic diseases in multimorbidity indices? Development of a new method on the basis of individual data from five population-based studies. *Journal of Clinical Epidemiology* 2012;65(6):679-85. doi: 10.1016/j.jclinepi.2011.11.006
102. NatCen Social Research, University College London Department of Epidemiology and Public Health. Health Survey for England, 2014. [computer file], 1st Edition. UK Data Archive, SN: 7919, 2016. http://dx.doi.org/10.5255/UKDA-SN-7919-1
103. NatCen Social Research, University College London Department of Epidemiology and Public Health. Health Survey for England, 2013. [computer file], 1st Edition. UK Data Archive, SN: 7649, 2015. http://dx.doi.org/10.5255/UKDA-SN-7649-1
104. NatCen Social Research, University College London Department of Epidemiology and Public Health. Health Survey for England, 2012. [computer file], 1st Edition. UK Data Archive, SN: 7480, 2014. http://dx.doi.org/10.5255/UKDA-SN-7480-1
105. NatCen Social Research, University College London Department of Epidemiology and Public Health. Health Survey for England, 2011. [computer file], 1st Edition. UK Data Archive, SN: 7260, 2013. http://dx.doi.org/10.5255/UKDA-SN-7260-1
106. NatCen Social Research, Royal Free and University College Medical School Department of Epidemiology and Public Health. Health Survey for England, 2010. [computer file], 2nd Edition. UK Data Archive, SN: 6986, 2012. http://dx.doi.org/10.5255/UKDA-SN-6986-2
107. National Centre for Social Research, University College London Department of Epidemiology and Public Health. Health Survey for England, 2009. [computer file], 2nd Edition. UK Data Archive, SN: 6732, 2011. http://dx.doi.org/10.5255/UKDA-SN-6732-1
108. National Centre for Social Research, University College London Department of Epidemiology and Public Health. Health Survey for England, 2008. [computer file], 3rd Edition. UK Data Archive, SN: 6397, 2011. http://dx.doi.org/10.5255/UKDA-SN-6397-1
109. National Centre for Social Research, University College London Department of Epidemiology and Public Health. Health Survey for England, 2007. [computer file], 2nd Edition. UK Data Archive, SN: 6112, 2010. http://dx.doi.org/10.5255/UKDA-SN-6112-1
110. National Centre for Social Research, University College London Department of Epidemiology and Public Health. Health Survey for England, 2006. [computer file], 2nd Edition. UK Data Archive, SN: 4150, 2008.
111. National Centre for Social Research, University College London Department of Epidemiology and Public Health. Health Survey for England, 2005. [computer file], 3rd Edition. UK Data Archive, SN: 5675, 2011. http://dx.doi.org/10.5255/UKDA-SN-5675-1
112. National Centre for Social Research, University College London Department of Epidemiology and Public Health. Health Survey for England, 2004. [computer file], 2nd Edition. UK Data Archive, SN: 5439, 2010. http://dx.doi.org/10.5255/UKDA-SN-5439-1
113. National Centre for Social Research, University College London Department of Epidemiology and Public Health. Health Survey for England, 2003. [computer file], 2nd Edition. UK Data Archive, SN: 5098, 2010. http://dx.doi.org/10.5255/UKDA-SN-5098-1

For peer review only - http://bmjopen.bmj.com/site/about/guidelines.xhtml

114. National Centre for Social Research, University College London Department of Epidemiology and Public Health. Health Survey for England, 2002. [computer file], 2nd Edition. UK Data Archive, SN: 4912, 2010. http://dx.doi.org/10.5255/UKDA-SN-4912-1
115. National Centre for Social Research, University College London Department of Epidemiology and Public Health. Health Survey for England, 2001. [computer file], 3rd Edition. UK Data Archive, SN: 4628, 2010. http://dx.doi.org/10.5255/UKDA-SN-4628-1
116. National Centre for Social Research, University College London Department of Epidemiology and Public Health. Health Survey for England, 2000. [computer file], 4th Edition. UK Data Archive, SN: 4487, 2011. http://dx.doi.org/10.5255/UKDA-SN-4487-1
117. National Centre for Social Research, University College London Department of Epidemiology and Public Health. Health Survey for England, 1999. [computer file], 3rd Edition. UK Data Archive, SN: 4365, 2002.
118. National Centre for Social Research, University College London Department of Epidemiology and Public Health. Health Survey for England, 1998. [computer file], 5th Edition. UK Data Archive, SN: 4150, 2010. http://dx.doi.org/10.5255/UKDA-SN-4150-1
119. Joint Health Surveys Unit of Social and Community Planning Research, University College London Department of Epidemiology and Public Health. Health Survey for England, 1997. [data collection], 4th Edition. UK Data Service, SN: 3979, 2017. http://doi.org/10.5255/UKDA-SN-3979-2
120. Joint Health Surveys Unit of Social and Community Planning Research, University College London Department of Epidemiology and Public Health. Health Survey for England, 1996. [data collection], 5th Edition. UK Data Service, SN: 3886, 2017. http://doi.org/10.5255/UKDA-SN-3886-2
121. Joint Health Surveys Unit of Social and Community Planning Research, University College London Department of Epidemiology and Public Health. Health Survey for England, 1995. [data collection], 5th Edition. UK Data Service, SN: 3796, 2017. http://doi.org/10.5255/UKDA-SN-3796-2
122. Joint Health Surveys Unit of Social and Community Planning Research, University College London Department of Epidemiology and Public Health. Health Survey for England, 1994. [data collection], 5th Edition. UK Data Service, SN: 3640, 2017. http://doi.org/10.5255/UKDA-SN-3640-2

For peer review only

For peer review only - http://bmjopen.bmj.com/site/about/guidelines.xhtml

WEB APPENDICES

***Table of contents** (pages refer to within web appendices)*

Appendix 1: Working-age mortality trends 2
Appendix 2: Overall missingness in health measures 3
Appendix 3: Changing non-response & weights 6
Appendix 4: General self-reported health/disability 10
Appendix 5: Health measures 12
Appendix 6: Measures not included in the main paper 24
Appendix 7: Year-by-year trends 29
Appendix 8: Others' analyses over change over time using HSE data 35
Appendix 9: Summarising multiple measures 37
Bibliography for Web Appendices 42

For peer review only

Appendix 1: Working-age mortality trends

Mortality in general

Given debates about whether historic improvements in life expectancy are being sustained, particularly in the US and UK, [1 2] it is important to note that in the period under study in this paper, working-age life expectancy was increasing. This can be seen in data from the Human Mortality Database (May 2016 update) 1993-2013, using one-year age and one-year period. This data shows that increases in mortality are not found for working-age people as a whole in any major country – for example, standardised working-age death rates have declined by 23% in the US and 35% in the UK over 1993-2013.

Cause-specific mortality for the 0-64 population

The main text refers to cause-specific morality in several places, referring to the death rate among 0-64 year olds from cardiovascular disease (CVD), respiratory conditions, diabetes, and liver cirrhosis. These death rates refer to UK deaths within relevant ICD-10 codes (I00-I99 for CVD, J00-J99 for respiratory conditions, E10-E14 for diabetes), standardised to the European standard population, and taken from the World Health Organization European Office's Health for All Database (May 2016 version), http://www.euro.who.int/en/data-and-evidence/databases/european-health-for-all-database-hfa-db.

For peer review only

For peer review only - http://bmjopen.bmj.com/site/about/guidelines.xhtml

Appendix 2: Overall missingness in health measures

This appendix refers to overall item-level missingness; changing item- and unit-level missingness is covered in Appendix 3.

Interview measures

For those who took part in the initial face-to-face interview, the level of item missingness is shown below (including only those years in which each question was asked). This shows the item-missingness is generally very low – only 1 of the 30 measures variables have item-missingness greater than 1%.

Table 1: Missingness at the initial face-to-face interview

	n non-missing	*n* missing	% missingness
BMI	124,682	15,415	11.0%
Any recent CVD	43,274	354	0.8%
Recent high blood pressure	43,366	262	0.6%
Breathlessness-Grade 2+	25,620	68	0.3%
Breathlessness-Grade 3	25,620	68	0.3%
Recent heart attack/stroke	43,519	109	0.3%
COPD symptoms	25,631	57	0.2%
Recent angina	43,551	77	0.2%
Heart attack symptoms	43,595	33	0.1%
Angina symptoms	43,592	36	0.1%
Recent diabetes	66,637	54	0.1%
Mini stroke (TIA) symptoms	23,487	16	0.1%
Diagnosed asthma	41,225	28	0.1%
Wheezing stopping sleep	41,224	29	0.1%
Recent wheezing/asthma	41,224	29	0.1%
Locomotor limitation	25,347	10	0.0%
Self-care limitation	25,347	10	0.0%
Limitations in past 2wks	140,041	56	0.0%
Longstanding illness (LSI)	124,906	43	0.0%
Limiting LSI (LLSI)	104,798	36	0.0%
Any CVD LSI	124,912	37	0.0%
IHD/stroke LSI	124,912	37	0.0%
Mental health LSI	124,912	37	0.0%
Arthritis LSI	124,912	37	0.0%
Asthma LSI	124,912	37	0.0%
Diabetes LSI	124,912	37	0.0%
High blood pressure LSI	124,912	37	0.0%
Other musculoskeletal LSI	124,912	37	0.0%
Good general health	140,048	49	0.0%
Bad general health	140,048	49	0.0%

The only variable with noticeable missingness is BMI, which is understandable as this involves the interviewer taking height and weight measurements rather than simply asking for a verbal response. There are various reasons why people do not have a BMI measurement:

For peer review only - http://bmjopen.bmj.com/site/about/guidelines.xhtml

- *High weight*: people with a very high weight are not weighed in HSE 'because the scales are inaccurate above this level', but the definition of this changed (from 130kg before 2011 to 200kg afterwards). This only applied to <0.1% of respondents 2012-14.
- *Difficult to take measurement*: other respondents (between 3.8% and 6.1% depending on the year) have no valid BMI measurement because height or weight measures were not attempted, attempted but not obtained or useable, because the respondent was pregnant, or the respondent was too sick or unsteady.
- *Refusal*: the most common reason for no BMI measurement is an outright refusal (including those refusing out of anxiety, though this tends to be a minor reason). Refusal rates are 8.3% in 2014.

Self-completion measures

For those who completed the self-completion booklet, the level of item missingness is shown in the table below.

Table 2: Missingness within the self-completion booklet

	n *non-missing*	*n* *missing*	% *missingness*
Psychological distress symptoms	108,324	2,462	2.2%
Problems washing/dressing today	62,703	1,310	2.1%
Anxiety/depression	62,725	1,288	2.0%
Problems w/activities	62,742	1,271	2.0%
Problems walking about today	62,772	1,241	1.9%
Pain	62,783	1,230	1.9%

Item missingness is relatively low compared to missingness from not completing the self-completion survey (51.5% of respondents in 2014).

Nurse visit measures

For those who took part in the nurse visit, the level of item missingness is shown in the table below.

Table 3: Missingness within the nurse visit

	n *non-missing*	*n* *missing*	% *missingness*
Biomarker high blood pressure	87,726	15,517	15.0%
High waist-hip ratio	78,637	2,664	3.3%

This shows that far more people have missing observations for measured high blood pressure than for their waist-hip ratio. This is despite the fact that we explicitly INCLUDE those who are on blood pressure-lowering drugs (about 5% of the sample at the start of the period and 10% at the end), on the grounds that their lowered blood pressure still conveys useful information about their health state. The main reason for the remaining high level of missingness is because people have recently exercised, smoked, drank or ate (12.2%).

Blood sample measures

For those from whom a blood sample was taken, the level of item missingness is shown in the table below.

For peer review only - http://bmjopen.bmj.com/site/about/guidelines.xhtml

Table 4: Missingness within the blood sample

	n *non-missing*	*n* *missing*	*%* *missingness*
Raised fibrinogen	16,166	3,341	17.1%
Raised C-reactive protein	17,814	1,693	8.7%
Glycated haemoglobin	28,810	1,436	4.8%
Anaemia	20,302	939	4.4%
Iron deficiency	20,375	866	4.1%
Low HDL cholesterol	36,076	1,406	3.8%
High total cholesterol	43,409	1,472	3.3%

All of these measures are affected by problems in transferring and storing the blood sample and with the measurement process, which results in problems with 3-10% of the blood samples depending on the measure and year. As for blood pressure, we explicitly INCLUDE those who are on lipid-lowering drugs (0.4% 1994 to 7.9% 2014), on the grounds that their changed cholesterol level still conveys useful information about their health state. Item missingness is highest for fibrinogen, which not only has high rates of such failures (7.0-9.5%), but also has ineligibility due to likely infection (from raised CRP, 3.6-5.6% of those with blood samples) and taking drugs that affect the reading (3.7% to 7.7% dependent on the year). Item missingness is also high for C-reactive protein (CRP), which also excludes those with likely infections.

Dealing with item-level missingness

Because of the high level of item non-response for certain measures (BMI, high blood pressure, fibrinogen, and CRP), and moderate level for others (other blood sample biomarkers and waist-hip ratio) – and because of evidence of changing non-response at various stages of the survey process – non-response weights were created to try to correct for any biases that these introduce. This is described in further detail in Appendix 3.

For peer review only - http://bmjopen.bmj.com/site/about/guidelines.xhtml

Appendix 3: Changing non-response & weights

This appendix focuses on *changes* in unit-level non-response at different stages of HSE.

Changing non-response

Sample frame coverage

As noted in the main paper, HSE is a household sample that excludes those in communal establishments. If we combine data from the 1991, 2001 and 2011 Censuses,[1] the communal population is as follows:

Table 5: Population in communal establishments over time (all working-age) and by age (in 2011)

		Education	Medical/ care	Defence	Prison	Other / not stated
**All working age**	*1991*	21,149	86,683	44,562	13,279	63,340
	2001	204,606	73,705	46,428	44,185	86,288
	2011	328,772	76,026	41,659	47,849	61,124
**16-24**	*2011*	305,154	9,346	22,677	12,607	25,673
**25-34**	*2011*	20,443	12,000	15,025	15,407	14,417
**35-49**	*2011*	2,663	26,796	3,725	14,725	14,708
**50-SPA[1] (est)**	*2011*	512	27,884	232	5,110	6,326

[1] SPA = State Pension Age, which is 60 for women and 65 for men. This is estimated because the Census totals are given for 50-64 year olds, so we have excluded 1/3 of women aged 50-64 from these totals.

This shows two things. Firstly, that there was a sharp rise in the working-age population in communal establishments 1991-2001 (from 230k to 560k), which was concentrated (>90% of the rise) among education-related communal establishments – although this is perhaps a slight overestimate given a definition change in the Census data.[2] Secondly, looking at education-related communal establishments in 2011, these are overwhelmingly (>90%) among 16-24 year olds. It therefore seems likely that the exclusion of communal establishments in HSE will lead to biases in young adults, and we therefore exclude 16-24 year olds from the trend analyses.

[1] Data are obtained from nomis on 6/8/2015, from Census tables DC1104EW and DC4210EWla (2011), S126 (2011) and L03/L04/L05 (2001).
[2] The guide to Census SARs notes, *"In the 1991 Census, students and schoolchildren were treated as usually resident at their 'home' or vacation address. In the 2001 census students and schoolchildren in full-time education studying away from the family home were enumerated as resident at their term-time address."* See https://census.ukdataservice.ac.uk/use-data/guides/microdata/comparability-91-01 [accessed 1/11/2016].

For peer review only - http://bmjopen.bmj.com/site/about/guidelines.xhtml

Changing unit non-response within the sample frame

As noted in the main paper, HSE supplies non-response weights from 2003, including adjustments for non-response to the nurse visit and blood sample using health and socioeconomic status from the initial interview. However, there had been a substantial decline in response rates prior to 2003, as shown in the table below:

Table 6: Response rates to HSE

	Household	Individual	Self-comp.	BMI	Nurse	Blood
1991	85.3%	81.1%				
1992	81.8%	77.4%				
1993	80.8%	75.7%				
1994	77.4%	71.6%	71.2%	67.1%	63.3%	53.3%
1995	78.3%	72.9%	72.0%	66.8%	63.7%	
1996	79.4%	74.7%	73.7%	69.6%	66.1%	
1997	76.0%	71.1%	69.8%	66.9%	64.0%	
1998	74.0%	68.9%	66.7%	63.3%	59.6%	49.0%
1999	76.2%	70.3%	68.5%	63.6%		
2000	75.5%	68.4%	65.8%	60.5%	58.2%	
2001	74.2%	67.1%	64.5%	60.1%	54.2%	
2002	74 %	67 %	64.4%	59.6%	54.3%	
2003	72.7%	66.4%	64.1%	59.7%	52.2%	39.9%
2004	72.4%	65.6%	62.4%	56.1%		
2005	71.4%	64.1%	60.6%	54.8%	46.7%	
2006	68.1%	60.5%	57.7%	52.8%	45.4%	34.7%
2007	65.7%	58.3%	56.1%	51.3%	42.6%	
2008	64.5%	57.9%	55.9%	50.0%	41.5%	30.4%
2009	67.6%	61.0%	58.7%	52.5%	43.1%	33.7%
2010	66.1%	58.7%	54.9%	49.3%	39.1%	29.9%
2011	65.7%	58.9%	54.3%	49.0%	39.4%	29.8%
2012	64.1%	56.3%	52.5%	47.4%	36.3%	27.9%
2013	63.8%	57.6%	54.2%	49.3%	40.1%	31.2%
2014	61.6%	55.5%	51.5%	48.4%	37.3%	28.7%

For peer review only

In general these trends are due to increases in refusal rates. However, the blood sample response rate is affected by two noticeable changes in eligibility over this period (people who are pregnant or who had blood/clotting disorders were ineligible throughout):

1. In 1998, people who had ever had an epileptic fit were excluded from the blood sample. This raised the ineligibility rate to 3.5% of the sample in 1998, from 0.6% in 1994.
2. In 2010, this was then relaxed so that those who had had an epileptic fit more than 5 years ago were again included in the blood sample. This lowered the ineligibility rate from 3.1% in 2009 to 2.4% in 2010.

Changing item non-response within responding people

There are also changes over time in item non-response (further detail on overall item non-response is given in Appendix 2). This includes:

- *BMI:* there has been little systematic trend in one reason for the absence of a BMI measure (difficulty in taking BMI measurements). However, there are trends in other reasons:

For peer review only - http://bmjopen.bmj.com/site/about/guidelines.xhtml

  - *High weight*: the definition of high weight changed from 130kg before 2011 to 200kg afterwards. 1.0% of respondents were not weighted for this reason in 2010, which fell to <0.1% 2012-14.
  - *Refusal*: in line with the general participation rates at each stage of the interview above, BMI refusal rates rose sharply from 1.9% in 1994 to a peak of 11.5% in 2011, and remain at 8.3% in the 2014 data.
- *Psychological distress:* similarly to wider participation rates at each stage of the survey, item missingness within the self-completion survey does increase over time (e.g. for psychological distress symptoms, from 1.8% 1994 to 5.9% 2014).
- *Measured high blood pressure:* there was a noticeable rise over time in exclusion of high blood pressure measures on the grounds that people recently exercised, smoked, drank or ate (from 6.1% to 13.6%).
- *Fibrinogen:* taking drugs that affect the fibrinogen reading rose from 3.7% 1994 to 7.7% 2009.

Creating non-response weights

To increase comparability over time, we create new weights 1994-2014 in several phases.

First-stage non-response weights

Firstly, we created a selection weight because some households were slightly more likely to be interviewed than others. (Until 2009, only three households at each address were interviewed. Those living at addresses with many households are therefore less likely to be interviewed). NatCen supplied selection weights for 2004-2013 to enable this (funded by this project), which are not available on the public HSE datasets.

Secondly, after adjusting for the selection weight, we created new individual-level (inverse probability) weights to match population age-sex-region totals in each year. Population data are annual mid-year population estimates from nomis. NatCen added the region variable for the 1994-1997 datasets to the public HSE datasets to enable this.

Second-stage non-response weights

After the first-stage adjustment for individual non-response, for the later stages of the interview (self-completion, BMI measurement, nurse visit, blood sample), we created a further weight that adjusts for non-response among those responding to the individual interview. This is based on a logit regression model to predict that stage of response based on:

- Age and gender (4 age group categories interacted with gender);
- Qualifications (degree or FT student / A-level or above / other qualifications / no qualifications);
- Household type (presence of other adults in the household);
- Employment status (yes/no);
- Smoking (never regular smoker / ex-regular smoker / current regular smoker); and
- Self-reported general health (bad or very bad health vs. other categories).

On the basis of these criteria, we create inverse probability weights – that is, we create a predicted probability of response for each respondent based on the logit regression model, and then create a

For peer review only - http://bmjopen.bmj.com/site/about/guidelines.xhtml

weight that is the inverse of this predicted probability. The revised weights are included in the Stata code to enable replication of the full paper.

Final sample size

The final sample size is as follows:

Table 7: HSE sample size in each year

	Interview	Self-completion	Nurse visit	Blood sample
1994	9,948	9,884	8,786	7,399
1995	10,167	10,049	8,881	
1996	10,401	10,269	9,206	
1997	5,563	5,458	5,005	
1998	10,177	9,843	8,805	7,236
1999	5,008	4,884		
2000	5,188	4,993	4,417	
2001	10,002	9,613	8,079	
2002	4,662	4,482	3,775	
2003	9,420	9,089	7,395	5,665
2004	4,165	3,961		
2005	4,810	4,548	3,505	
2006	8,825	8,420	6,622	5,064
2007	4,198	4,039	3,064	
2008	9,242	8,922	6,625	4,845
2009	2,795	2,689	1,973	1,542
2010	5,120	4,794	3,411	2,610
2011	5,258	4,853	3,518	2,667
2012	4,936	4,605	3,188	2,447
2013	5,303	4,992	3,691	2,875
2014	4,909	4,552	3,297	2,531
**Total**	**140,097**	**134,939**	**103,243**	**44,881**

For peer review only

For peer review only - http://bmjopen.bmj.com/site/about/guidelines.xhtml

Appendix 4: General self-reported health/disability

Trends in seven general health/disability measure are available in HSE:

Table 8: HSE general health measures

Measure	Operationalisation (years available)
Good general health	Health in general is 'good' or 'very good' (1994-2014)
Bad general health	Health in general is 'bad' or 'very bad' (1994-2014)
Longstanding illness (LSI)	Any long-standing illness, disability or infirmity (1994-2011)
Limiting LSI (LLSI)	LSI limits activities in any way (1996-2011)
Problems with activities-some	Some problems with performing usual activities (1996-2014)
Problems with activities-unable	Unable to perform usual activities (1996-2014)
Limitations in past 2wks	Cut down on activities in past 2wks due to LSI or other illness/injury (1994-2014)

See Web Appendix 5 for full details on all measures .

Trends for these measures are shown in Table 9 below. Looking first at good general health, the table shows the trend from 1994-6, when 80.9% reported good general health. By 2011-14, there had been a decline of 0.8 percentage points. When we adjust for the changing age and sex distribution of the working-age population (labelled 'Adj.' in Table 1), the decline is only 0.1%, with a wide confidence interval (-0.9 to +0.7%), and there is therefore little evidence for any systematic trend.

Table 9: Changes over time in general health

	Starting period		Change from start to end period			
	Period	Prevalence	End period	Raw change	Adj.[a] change	Adj. change 95% CI
Good general health	1994-96	80.9%	2011-14	-0.8%	-0.1%	[-0.9, 0.7%]
Bad general health	1994-96	4.4%	2011-14	1.3%	1.0%	[0.6, 1.5%]
Longstanding illness (LSI)	1994-96	36.2%	2011-14	-1.0%	-2.0%	[-3.7, -0.3%]
Limiting LSI (LLSI)	1994-96	21.4%	2011-14	-2.9%	-3.6%	[-5.2, -2.1%]
Problems w/activities-some	1994-96	14.8%	2011-14	-1.2%	-1.8%	[-2.8, -0.8%]
Problems w/activities-unable	1994-96	1.9%	2011-14	-0.6%	-0.8%	[-1.1, -0.4%]
Limitations in past 2wks	1994-96	14.7%	2011-14	-0.1%	-0.3%	[-1.0, 0.4%]

[a] 'Adj.' = adjusted for changing age and sex distribution of the working-age population.

For several of the general health measures, there is evidence of change over this period – but interpreting this is difficult, because the trends are in opposite directions. There is strong evidence for a *rise* in bad general health (a rise of 0.6-1.5% from a base of 4.4%), yet equally strong evidence for a *decline* in having problems with everyday activities (at both levels of severity), and being limited in activities by a longstanding illness. This shows the challenges in tracking population morbidity change through general, non-specific measures, which are likely to be as influenced by changes in reporting styles as much as changes in morbidity *per se*.

As an aside, UK Government publications have made claims based on healthy/disability-free life expectancy – sometimes using these to argue that morbidity has been improving [3], but more recently to argue that morbidity has been deteriorating.[4-6] However, these trends are potentially misleading: they include older people as well as the working-age population; they confuse a

For peer review only - http://bmjopen.bmj.com/site/about/guidelines.xhtml

combined mortality-morbidity measure with morbidity; and they are based on self-reports of global health that are unreliable, as we show here and discuss in the main text.

For peer review only

For peer review only - http://bmjopen.bmj.com/site/about/guidelines.xhtml

Appendix 5: Health measures

We systematically searched HSE questions, and have included every morbidity measure that is comparable over a significant duration. We have excluded questions only available for short time frames (ADLs 2012-14, EQ-5D visual analogue scale 2008-14, SF-12 1996-2000, eczema/hayfever 1995-2001, breathlessness 1991-98 and 1995-2001, lung function 1995-2001, bladder limitations 1995-2001, LDL cholesterol, triglycerides and glucose 1999-2003, IgE 1996-2002 and an alternate measure of high blood pressure 2009-14), with the exception of five key measures of activity limitations 1995-2001. We have also excluded questions that are not direct measures of health (medication or health service use, demispan, health risk factors such as fractures, accidents, alcohol/tobacco use (including biomarkers), physical activity, and wellbeing).

Short summaries of the resulting 39 measures are given in this paper, and full details are given in the table below. Measures are taken from the initial face-to-face survey unless otherwise specified. The Stata code to create these variables in consistent form from the publicly available HSE files are available from OSF[7] and www.benbgeiger.co.uk.

Measure	Details
Activity limitations and MSDs	
Problems walking today	In the self-completion survey in 1996, 2003-6, 2008, 2010-12 and 2014, respondents were asked *'Now we would like to know how your health is today. Please answer ALL the questions. By ticking one box for each question below, please indicate which statements best describe your own health state today'*:<br>- *"I have no problems in walking about"*<br>- *"I have some problems in walking about"*<br>- *"I am confined to bed"*<br>[This is part of the widely-used EQ-5D health status indicator [8]. However, for the purposes of this paper we have separated the individual measures that make up the EQ-5D in order to compare these to similar indicators of morbidity within each domain].<br>People are classified as having a problem with self-care today if they had some problems walking about or were confined to bed.
Locomotor limitation	This is based on the personal care disability scale used in the 2001 HSE report [9]. Respondents in 1995, 2000 and 2001 were asked if any of the following applied to them (interviewers were instructed to ignore temporary disabilities that are expected to last less than one year):<br>- *"Cannot walk 200 yards or more on own without stopping or discomfort"*. People who reported a limitation were asked if they used a walking aid, and if they did, were then asked if they could walk 200 yards without the walking aid.<br>- *"Cannot walk up and down a flight of 12 stairs without resting"*<br>- *"Cannot bend down and pick up a shoe from the floor when standing"*<br>People are classified as having a locomotor limitation if they reported ANY of these limitations.
Problems with washing/dressing today	In the self-completion survey in 1996, 2003-6, 2008, 2010-12 and 2014, respondents were asked *'Now we would like to know how your health is today. Please answer ALL the questions. By ticking one box for each question below, please indicate which statements best describe your own health state today'*:<br>- *"I have no problems with self-care"*<br>- *"I have some problems washing or dressing myself"*<br>- *"I am unable to wash or dress myself"*<br>[This is part of the widely-used EQ-5D health status indicator [8]. However, for the purposes of this paper we have separated the individual measures that make up the EQ-

For peer review only - http://bmjopen.bmj.com/site/about/guidelines.xhtml

	5D in order to compare these to similar indicators of morbidity within each domain]. People are classified as having a problem with self-care today if they had some problems washing/dressing or were unable to wash/dress themselves.
Self-care limitation	This is based on the personal care disability scale used in the 2001 HSE report [9]. Respondents in 1995, 2000 and 2001 were asked if any of the following applied to them (interviewers were instructed to ignore temporary disabilities that are expected to last less than one year):<br>- *"Cannot get in and out of bed on own without difficulty"*<br>- *"Cannot get in and out of a chair without difficulty"*<br>- *"Cannot dress and undress without difficulty"*<br>- *"Cannot wash hands and face without difficulty"*<br>- *"Cannot feed, including cutting up food without difficulty"*<br>- *"Cannot get to and use toilet on own without difficulty"*<br>People are classified as having a self-care limitation if they reported ANY of these limitations.
Pain *(any / extreme)*	In the self-completion survey in 1996, 2003-6, 2008, 2010-12 and 2014, respondents were asked *'Now we would like to know how your health is today. Please answer ALL the questions. By ticking one box for each question below, please indicate which statements best describe your own health state today'*:<br>- *"I have no pain or discomfort"*<br>- *"I have moderate pain or discomfort"*<br>- *"I have extreme pain or discomfort"*<br>[This is part of the widely-used EQ-5D health status indicator [8]. However, for the purposes of this paper we have separated the individual measures that make up the EQ-5D in order to compare these to similar indicators of morbidity within each domain].<br>Two outcome measures are based on this: whether people have any pain (the 2nd and 3rd categories combined), and whether they have extreme pain (3rd category only).
Arthritis LSI	Every year 1994-2011, people who report a longstanding illness (LSI) are then asked, *'what is the matter with you?'*; up to 6 responses are then coded by the interviewer into a consistent coding frame based on the International Classification of Diseases.<br>The arthritis LSI measure is based on the group labelled *'Arthritis/rheumatism/fibrositis'*, which as of 2011 includes: Arthritis as result of broken limb; Arthritis/rheumatism in any part of the body; Gout; Osteoarthritis, rheumatoid arthritis, polymyalgia rheumatic; Polyarteritis Nodosa; Psoriasis arthritis; Rheumatic symptoms; and Still's disease.<br>While the LSI coding frame generally stays consistent over this period, interpretation of 'LSI arthritis' is complicated by two changes: Gout and Polyarteritis Nodosa are moved into this code (the documentation is not clear on whether this occurred in 2000 or 2001).
Other musculoskeletal LSI	People who report a longstanding illness (LSI) are then asked, *'what is the matter with you?'*; up to 6 responses are then coded by the interviewer into a consistent coding frame based on the International Classification of Diseases.<br>The other musculoskeletal LSI measure is based on the groups labelled *'Back problems/slipped disc/spine/neck'* and *'Other problems of bones/joints/muscles'*, which as of 2011 includes: Brittle bones, osteoporosis; Bursitis, housemaid's knee, tennis elbow; Cartilage problems; Chondrodystrophia; Chondromalacia; Cramp in hand; Deformity of limbs eg. club foot, claw-hand, malformed jaw; Delayed healing of bones or badly set fractures; Deviated septum; Disc trouble; Dislocations eg. dislocation of hip, clicky hip, dislocated knee/finger; Disseminated lupus; Dupuytren's contraction; Fibromyalgia; Flat feet, bunions; Fracture, damage or injury to extremities, ribs, collarbone, pelvis, skull, eg. knee injury, broken leg, gun shot wounds in leg/shoulder, can't hold arm out flat - broke it as a child, broken nose; Frozen shoulder; Hip infection, TB hip; Hip replacement (nes); Legs won't go, difficulty in walking; Lumbago, inflammation of spinal joint; Marfan Syndrome; Osteomyelitis; Paget's disease; Perthe's disease; Physically handicapped (nes); Pierre Robin syndrome; Prolapsed invertebral discs; Schlatter's disease; Schuermann's

For peer review only - http://bmjopen.bmj.com/site/about/guidelines.xhtml

	disease; Sever's disease; Spondylitis, spondylosis; Stiff joints, joint pains, contraction of sinews, muscle wastage; Strained leg muscles, pain in thigh muscles; Systemic sclerosis, myotonia (nes); Tenosynovitis; Torn muscle in leg, torn ligaments, tendonitis; Walk with limp as a result of polio, polio (nes), after affects of polio (nes); Weak legs, leg trouble, pain in legs; and Worn discs in spine - affects legs. The code explicitly excludes: Damage/injury to spine results in paralysis; Sciatica or trapped nerve in spine; and Muscular dystrophy.
Circulatory	
High blood pressure LSI	Every year 1994-2011, people who report a longstanding illness (LSI) are then asked, *'what is the matter with you?'*; up to 6 responses are then coded by the interviewer into a consistent coding frame based on the International Classification of Diseases.<br>The high blood pressure LSI measure is based on the group labelled *'Hypertension/high blood pressure/blood pressure (nes)'*, which as of 2011 includes only the conditions listed in the group label.
Recent high blood pressure	Respondents in 1994, 1998, 2003, 2006 and 2009-2014 were asked a series of questions on whether they have high blood pressure:<br>- *"Do you now have, or have you ever had... high blood pressure (sometimes called hypertension)?"*<br>- Those responding 'yes' were then asked *"Were you told by a doctor or nurse that you had high blood pressure?"*<br>- Women responding 'yes' were then asked, *"Can I just check, were you pregnant when you were told that you had high blood pressure?"*, and those responding 'yes' were then asked *"Have you ever had high blood pressure apart from when you were pregnant?"*<br>- Finally, those with doctor-diagnosed high blood pressure (excluding only when pregnant were asked: *"Are you currently taking any medicines, tablets or pills for high blood pressure?*", and those saying 'no' (or not giving an answer) were then asked, *"Do you still have high blood pressure?"*<br>People were considered to have recent high blood pressure if they said they had ever been diagnosed as having high blood pressure by a doctor (excluding when pregnant), and that they still have high blood pressure or are currently taking medicines for it.<br>While the question wording has stayed consistent, a discontinuity seems to be introduced by a change in question context. In some years (1994, 1998, 2003, 2006 and 2011), this question was preceded by a question that asked, *"May I just check, have you ever had your blood pressure measured by a doctor or nurse?"* (and then for those saying yes, they were asked how recently this was, and whether they were told that it was *'normal (alright/fine), higher than normal, lower than normal, or were you not told anything?'*). However, in other years (2009-10, 2012-14), this question was not asked. Given the way in which context can affect question interpretation, we treat these as two separate measures of recent high blood pressure.
Biomarker high blood pressure	During the nurse visit (which took place for all consenting respondents in all years except 1999, 2002 and 2004, when the nurse visit focussed on particular subsamples), respondents' blood pressure was measured.<br>High blood pressure is defined as a systolic blood pressure >= 140mmHg and diastolic blood pressure >= 90mmHg following HSE established practice, in turn following [10].<br>The measurement of blood pressure changed in 2003, from a Dinamap monitor to an Omron monitor. A conversion is available between the two monitors based on a calibration study, and this has been regularly used by the HSE team to produce continuous trends in blood pressure – see www.hscic.gov.uk/catalogue/PUB00480. For adults, the conversion is as follows:<br>○ For systolic blood pressure: *Predicted Omron=8.90 (SE=2.94) + 0.91 (SE=0.02) * Dinamap.*<br>○ For diastolic blood pressure: *Predicted Omron=19.78 (SE=1.86) + 0.73 (SE=0.03) * Dinamap.*<br>There are several reasons why respondents who had a nurse visit do not have a valid

For peer review only - http://bmjopen.bmj.com/site/about/guidelines.xhtml

	blood pressure measurement – these are discussed in the Web Appendices 2 and 3.
High cholesterol	In the years 1994, 1998, 2006, and 2008-14, blood samples were obtained during the nurse visit, which were then analysed for total cholesterol. A high level of total cholesterol ('hypercholesterolaemia') is an established risk factor for CVD, and high cholesterol is defined following conventional practice at the NICE guidance 'audit level' of 5mmol/L or above [11 12].<br>The measurement of cholesterol changed slightly in 2010 when a new laboratory was used. This resulted in values that are an average of 0.1mmol/L higher, and later values are therefore adjusted by this amount to maintain comparability over time as in [11].
Low HDL cholesterol	In the years 1994, 1998, 2006, and 2008-14, blood samples were obtained during the nurse visit, which were then analysed for high density lipoprotein (HDL) cholesterol. HDL cholesterol *reduces* the risk of CVD (it carries cholesterol away from the arteries towards the liver), and it is therefore low HDL cholesterol that indicates poorer health; low HDL cholesterol is here defined as 1 mmol/L or less [11 12].<br>The measurement of HDL cholesterol changed slightly in 2010 when a new laboratory was used. This resulted in values that are an average of 0.1mmol/L lower, and later values are therefore adjusted by this amount to maintain comparability over time as in [11].
Recent heart attack/stroke	Respondents in 1994, 1998, 2003, 2006 and 2011 were asked a series of questions on whether they have had a heart attack (within a battery of questions about different types of heart disease):<br>- *"Have you ever had a heart attack (including myocardial infarction or coronary thrombosis)?"*<br>- Those responding 'yes' were then asked *"Were you told by a doctor that you had a Heart Attack (including myocardial infarction or coronary thrombosis)?"*<br>- Those with doctor-diagnosed angina were asked, *"Have you had a heart attack (including myocardial infarction and coronary thrombosis) during the past 12 months?"*<br>Respondents in these years were similarly asked about stroke:<br>- *"Have you ever had a stroke?"*<br>- Those responding 'yes' were then asked, *"Were you told by a doctor that you had a stroke?"*<br>- Those with doctor-diagnosed stroke were asked, *"Have you had a stroke during the past 12 months?"*<br>People were considered to have recent IHD or stroke if they said they had ever been diagnosed as having stroke or a heart attack by a doctor, and that they have had a heart attack or stroke during the past 12 months.
Recent angina	Respondents in 1994, 1998, 2003, 2006 and 2011 were asked a series of questions on whether they have angina (within a battery of questions about different types of heart disease):<br>- *"Have you ever had angina?"*<br>- Those responding 'yes' were then asked *"You said that you had Angina. Were you told by a doctor that you had Angina?"*<br>- Those with doctor-diagnosed angina were asked, *"Have you had angina during the past 12 months?"*<br>People were considered to have recent angina if they said they had ever been diagnosed as having angina by a doctor, and that they have had it during the past 12 months.
IHD LSI	Every year 1994-2011, people who report a longstanding illness (LSI) are then asked, *'what is the matter with you?'*; up to 6 responses are then coded by the interviewer into a consistent coding frame based on the International Classification of Diseases.<br>The IHD LSI measure is based on the groups labelled *'Stroke/cerebral haemorrhage/cerebral thrombosis'* and *'Heart attack/angina'*. As of 2011 this includes: Cerebro-vascular accident; Coronary thrombosis, myocardial infarction; Heart attack/angina; Hemiplegia, apoplexy, cerebral embolism; Stroke/cerebral haemorrhage/cerebral thrombosis; and Stroke victim - partially paralysed and speech difficulty.
Recent	Respondents in 1994, 1998, 2003, 2006 and 2011 were asked a series of questions on

For peer review only - http://bmjopen.bmj.com/site/about/guidelines.xhtml

cardiovascular disease (CVD)	different types of heart disease – including angina; heart attack (including myocardial infarction or coronary thrombosis); a heart murmur; abnormal heart rhythm; or other heart trouble. For EACH of these, they were asked:<br>- *“Have you ever had <type of heart disease>?”*<br>- Those responding ‘yes’ were then asked *“You said that you had <type of heart disease>. Were you told by a doctor that you had <type of heart disease>?”*<br>- For heart murmurs only, women saying they had doctor-diagnosed heart murmurs were asked if they were pregnant when told this, and if so, whether they were ever told they had a heart murmur when they were not pregnant.<br>- Those with doctor-diagnosed heart disease (excluding heart murmurs when pregnant) were asked, *“Have you had <type of heart disease> during the past 12 months?”*<br>People were considered to have recent CVD if they said they had a doctor-diagnosed heart condition and that they had had this during the past 12 months.
Cardiovascular (CVD) LSI	Every year 1994-2011, people who report a longstanding illness (LSI) are then asked, *‘what is the matter with you?’*; up to 6 responses are then coded by the interviewer into a consistent coding frame based on the International Classification of Diseases.<br>The CVD LSI measure is based on the groups labelled *‘Stroke/cerebral haemorrhage/cerebral thrombosis’, ‘Heart attack/angina’, Hypertension/high blood pressure/blood pressure (nes)’, ‘Other heart problems’, ‘Piles/haemorrhoids incl. Varicose Veins in anus’, ‘Varicose veins/phlebitis in lower extremities’, and ‘Other blood vessels/embolic’*. As of 2011 this includes: Aorta replacement; Aortic valve stenosis; Aortic/mitral valve regurgitation; Arterial thrombosis; Arteriosclerosis, hardening of arteries (nes); Artificial arteries (nes); Atrial Septal Defect (ASD); Blocked arteries in leg; Blood clots (nes); Cardiac asthma; Cardiac diffusion; Cardiac problems, heart trouble (nes); Cerebro-vascular accident; Coronary thrombosis, myocardial infarction; Dizziness, giddiness, balance problems (nes); Hand Arm Vibration Syndrome (White Finger); Hardening of arteries in heart; Heart attack/angina; Heart disease, heart complaint; Heart failure; Heart murmur, palpitations; Hemiplegia, apoplexy, cerebral embolism; Hole in the heart; Hypersensitive to the cold; Hypertension/high blood pressure/blood pressure (nes); Intermittent claudication; Ischaemic heart disease; Low blood pressure/hypertension; Mitral valve stenosis; Pacemaker; Pains in chest (nes); Pericarditis; Piles/haemorrhoids incl. Varicose Veins in anus; Poor circulation; Pulmonary embolism; Raynaud's disease; St Vitus dance; Stroke victim - partially paralysed and speech difficulty; Stroke/cerebral haemorrhage/cerebral thrombosis; Swollen legs and feet; Tachycardia, sick sinus syndrome; Telangiectasia (nes); Thrombosis (nes); Tired heart; Valvular heart disease; Valvular heart disease; Varicose veins in Oesophagus; Varicose veins/phlebitis in lower extremities; Various ulcers, varicose eczema; Weak heart because of rheumatic fever; Wolff - Parkinson - White syndrome; and Wright's syndrome. It explicitly <u>excludes</u> balance problems due to ear complaint & haemorrhage behind eye.<br>While the LSI coding frame generally stays consistent over this period, interpretation of ‘IHD LSI’ is complicated by two changes: ‘Too much cholesterol in blood’ is included in this category in 1994 only, and Polyarteritis Nodosa is later moved into this code (the documentation is not clear on whether this occurred in 2000 or 2001).
Angina symptoms	This is taken from the Rose Angina questionnaire [13,14]. Respondents in 1994, 1998, 2003, 2006 and 2011 were asked a series of questions about *symptoms* of heart trouble (rather than whether they had been diagnosed):<br>- *“I am now going to ask you some questions mainly about symptoms of the chest. Have you ever had any pain or discomfort in your chest?”*<br>- Those that said ‘yes’ were asked:<br>o *Do you get it when you walk uphill or hurry? Yes | No | Sometimes/ Occasionally | Never walks uphill or hurries | (Cannot walk)”*. If sometimes/occasionally, they were asked: *“Does this happen on most occasions?”*<br>o If not ‘no’ to having pain/discomfort in their chest, they were asked: *“Do you get it when you walk at an ordinary pace on the level? Yes | No |*

For peer review only - http://bmjopen.bmj.com/site/about/guidelines.xhtml

	Sometimes/Occasionally | Never walks at an ordinary pace on the level". If sometimes/occasionally, they were asked: *"Does this happen on most occasions?"*<br>- Those who every had pain/discomfort when walking uphill/hurrying or walking at ordinary pace on the level were asked:<br>○ *"What do you do if you get it while you are walking? Do you stop, slow down or carry on?"* (If respondents were unsure, they were asked, *"What do you do on most occasions?")*<br>○ Those who said they stop or slow down were asked, *"If you stand still does the pain go away or not?"* (If respondents were unsure, they were asked, *"What happens to the pain on most occasions?").* If the pain goes away, they were asked, *"How soon does the pain go away? Does it go in 10 minutes or less, or more than 10 minutes?"*<br>○ Those who said the pain goes away in 10 minutes or less were asked, *"Will you show me where you get this pain or discomfort? Where else"* The interviewer then coded the site as Sternum (upper or middle) | Sternum lower | Left anterior chest | Left arm | Right anterior chest | Right arm | (Somewhere else).<br>Following the HSE reports, possible angina is defined as chest pain or discomfort that (i) includes either the sternum or the left arm and left anterior chest; (ii) is prompted by hurrying or walking uphill (or by walking on the level, for those who never attempt more); (iii) makes the respondent either stop or slacken pace; and (iv) usually disappears in 10 minutes or less when they stand still.
Heart attack symptoms	This is taken from the Rose Angina questionnaire. Respondents in 1994, 1998, 2003, 2006 and 2011 were asked, *"Have you ever had a severe pain across the front of your chest lasting for half an hour or more?"* As in the 2006 HSE report, those responding 'yes' are treated as having a possible heart attack (myocardial infarction).
Mini stroke (TIA) symptoms	Respondents in 2003, 2006 and 2011 were asked:<br>○ *"In the last twelve months, have you had a sudden attack of weakness or numbness on one side of the body?"*<br>○ *"Have you had a sudden attack of slurred speech or difficulty in finding words in the last twelve months?"*<br>○ *"Have you had a sudden attack of vision loss or blurred vision in one or both eyes in the last twelve months?"*<br>People reporting ANY of these symptoms were considered as possibly having had a transient ischaemic attack (TIA), often called a 'mini stroke'.
Respiratory	
COPD symptoms	Respondents in 1995, 1996 and 2010 were asked:<br>○ *"Do you usually cough first thing in the morning in the winter?"* (In 2010 only, respondents had previously been asked *"Do you usually cough first thing in the morning?"* – but this is not used to filter people into the questions on coughing in winter).<br>○ *"Do you usually bring up any phlegm from your chest, first thing in the morning in the winter?"* (Again, this was asked to everyone in all years, but was preceded by an additional, non-winter-specific question in 2010).<br>○ Those saying 'yes' to each question were then asked, "*Do you [cough/bring up phlegm] like this on most days for as much as three months each year?"* In 2010 only, this was followed by the additional clarification '*That is, for three consecutive months*'.<br>People who reported three months/year of BOTH coughing first thing and of phlegm are considered to have possible symptoms of Chronic Obstructive Pulmonary Disease (COPD).
Diagnosed asthma	In 1995-7, 2001 and 2010, respondents were asked *"Did a doctor <1997 and 2010 only: or nurse> ever tell you that you had asthma?"* Whereas for other doctor-diagnosed conditions

For peer review only - http://bmjopen.bmj.com/site/about/guidelines.xhtml

	(heart problems/diabetes) we focus on those reporting problems in the past 12 months, it is not possible to construct a consistent measure of recent asthma, hence this variable refers to *lifetime* doctor-diagnosed asthma.
Asthma LSI	Every year 1994-2011, people who report a longstanding illness (LSI) are then asked, *'what is the matter with you?'*; up to 6 responses are then coded by the interviewer into a consistent coding frame based on the International Classification of Diseases.<br>The asthma LSI measure is based on the group labelled *'Asthma'*, which as of 2011 includes: Asthma; Bronchial asthma, allergic asthma; and Asthma - allergy to house dust/grass/cat fur. It explicitly <u>excludes</u> cardiac asthma.
Shortness of breath *(Grade 2+ / Grade 3)*	Respondents in 1995, 1996 and 2010 were asked the following questions about shortness of breath ('dyspnoea'):<br>○ *"Are you troubled by shortness of breath when hurrying on level ground or walking up a slight hill? Yes | No | Never walks up hill or hurries | Cannot walk"*<br>○ Those responding 'yes' or 'never walks up hill or hurries' are then asked, *"Do you get short of breath walking with other people of (your/his/her) own age on level ground? Yes | No | Never walks with people of own age on level ground"*.<br>○ Those responding 'yes' or 'never walks with people of own age' are then asked, *"Do you have to stop for breath after walking at (your/his/her) own pace on level ground?"*<br>This has been combined into the longstanding MRC dyspnoea scale [15] as follows:<br>- Grade 2 dyspnoea: people who report shortness of breath when hurrying on level ground or walking up a slight hill (or who report shortness of breath when walking on level ground, but who say they never walk up hill or hurry).<br>- Grade 3 dyspnoea: people who report shortness of breath when walking with people of own age on level ground, or who have to stop for breath when walking at own pace on level ground.<br>(The same questions also exist in 1994 and 1998, but (i) the wider bank of questions differs substantially in the two versions and question context effects are likely; and (ii) the filtering into the final question differs between versions. However, the 1991-98 trends are included below).
Recent wheezing/ asthma symptoms	Respondents in 1995-97, 2001 and 2010 were asked the following two questions as part of the battery of questions on breathing problems:<br>- *"I am now going to ask you some questions about your breathing... Have you ever had wheezing or whistling in the chest at any time, either now, or in the past?"*<br>- Those that said yes were then asked, *"Have you had wheezing or whistling in the chest in the last 12 months?"*<br>- (For those who said they had ever been told by a doctor they had asthma; see above), "*When was your most recent attack of asthma? PROMPT IF NECESSARY: Less than 4 weeks ago | More than 4 weeks but within the last 12 months | One to five years ago | More than 5 years ago"*<br>People who said they had EITHER wheezing/whistling in the past 12 months or an asthma attack in the past 12 months were counted as having recent wheezing/asthma symptoms.<br>[It should be noted that the filtering to the second question is very slightly different in 2010 compared to previous years (it was only asked to people who said they had not had wheezing/whistling in the chest in the past 12 months). However, given the way that the derived variable is calculated here, the change in filtering does not introduce any discontinuities over time].
Wheezing stopping sleep	Respondents in 1995-97, 2001 and 2010 were asked the following two questions as part of the battery of questions on breathing problems:<br>- *"I am now going to ask you some questions about your breathing... Have you ever had wheezing or whistling in the chest at any time, either now, or in the past?"*<br>- Those that said yes were then asked, *"Have you had wheezing or whistling in the chest in the last 12 months?"*<br>- Those that said yes were then asked, *"In the last 12 months, how often on average*

For peer review only - http://bmjopen.bmj.com/site/about/guidelines.xhtml

	has your sleep been disturbed due to wheezing or whistling in the chest?: Have you: Never woken with wheezing | Woken less than one night per week, or | Woken one or more nights per week?"<br>People were considered to have wheezing during sleep if they reported this at least once per week.
Anthropometric & diabetes	
BMI<br>*(Underweight / Obese)*	During the initial face-to-face interview in all years (except 2013), respondents were asked if they would consent to having their height and weight measured by the interviewer. The reasons for missingness (and their trends over time) are given in Web Appendices 2 & 3; note that there are three changes that give rise to small discontinuities in 2009 and 2011.<br>Obesity is a risk factor for diabetes (hence its inclusion in this section) but also heart disease and some cancers. Obesity is defined as a Body Mass Index (BMI) of >= 30kg/m^2 as per the World Health Organization's BMI classification [16]. Using the same definition, underweight is defined as <=18.5 kg/m^2.
High waist-hip ratio	During the nurse visit in most years (excluding 1995-96, 2002, 2004 and 2013), respondents had their waist and hip circumferences measured. While BMI is a standard measurement of obesity, some evidence suggests that fat around the waist – 'central adiposity' – is a greater risk to health than fat elsewhere [17]. We use NICE's suggested 2006 thresholds for a high waist-hip ratio of >1 for men and >0.85 for women [18], as used in Hotchkiss et al [19].
Recent diabetes	Respondents in 1994, 1998, 2003, 2006 and 2009-2014 were asked a series of questions on whether they have diabetes:<br>- *"Do you now have, or have you ever had diabetes?"*<br>- Those responding 'yes' were then asked *"Were you told by a doctor that you had diabetes?"*<br>- Women responding 'yes' were then asked, *"Can I just check, were you pregnant when you were told that you had diabetes?"*, and those responding 'yes' were then asked *"Have you ever had diabetes apart from when you were pregnant?"*<br>- Finally, those with doctor-diagnosed diabetes (excluding only when pregnant were asked: *"Do you currently inject insulin for diabetes?"* and *"Are you currently taking any medicines, tablets or pills (other than insulin injections) for diabetes?"*<br>People were considered to have recent diabetes if they said they had ever been diagnosed as having diabetes by a doctor (excluding when pregnant), and that they are injecting insulin or taking any other medicines for diabetes.
Diabetes LSI	Every year 1994-2011, people who report a longstanding illness (LSI) are then asked, *'what is the matter with you?'*; up to 6 responses are then coded by the interviewer into a consistent coding frame based on the International Classification of Diseases.<br>The diabetes LSI measure is based on the group labelled *'Diabetes'*, which as of 2011 includes Diabetes and Hyperglycaemia.
High glycated haemoglobin	In the years 2003, 2006, and 2008-14, blood samples were obtained during the nurse visit, which were then analysed for glycated haemoglobin (HbA_{1C}). HbA_{1C} is a measure of the share of haemoglobin (within red blood cells) that glucose is attached to, with higher levels indicated less well-controlled diabetes in the previous three months [20]. Following the recommendations of a 2009 expert committee, we mirror recent HSE reports in using a threshold of 48mmol/mol (i.e. 48 millimoles of glycated haemoglobin per mole of haemoglobin) as the threshold for raised HbA_{1C}, a different threshold to that used in earlier HSE reports.<br>While the measurement of HbA_{1C} has been consistent in HSE from 1994, the units reported have changed from the % of haemoglobin that is glycated to mmol/mol. Earlier measures have been transformed into mmol/mol through the formula, mmol/mol = (% - 2.15) x 10.929. HbA_{1C} was also measured in 1994 but using a different technique, which cannot be made comparable [21:67].
Other biomarkers	

For peer review only - http://bmjopen.bmj.com/site/about/guidelines.xhtml

Raised C-reactive protein	In the years 1998, 2003, 2006, and 2009, blood samples were obtained during the nurse visit, which were then analysed for C-reactive protein (CRP). CRP is an inflammatory marker, which can indicate heart-related inflammation (it is used to test for heart failure) but can also indicate other sorts of health damage including diabetes. However, there are still debates about exactly what CRP shows, both in terms of its causal role in heart disease, and whether it also indicates depression.[22]<br>Raised CRP is defined as >3mg/L, the standard cut-off for a clinically significant rise in CVD [23 24]. Participants with CRP >10mg/L are excluded, as this is taken to be evidence of current infection rather than inflammation from chronic disease.
Raised Fibrinogen	In the years 1998, 2003, 2006, and 2009, blood samples were obtained during the nurse visit, which were then analysed for fibrinogen. Like CRP, fibrinogen is an inflammatory marker, which is both commonly thought to be a causal risk factor for CVD (it is a component of coagulation), and which seems to be a risk factor for other diseases (including cancer and diabetes)[25].<br>While fibrinogen is often analysed as a continuous variable with no cutpoints [24], we here define raised fibrinogen as>4mg/L as in [12]. As for CRP, participants with CRP >10mg/L are excluded, as this is taken to be evidence of current infection rather than inflammation from chronic disease. A change of analysis method and laboratory between 1994 and 1998 means that the 1994 results are not comparable to the later results [26:8.10.4].
Anaemia	In the years 1994, 1998, 2006, and 2009, blood samples were obtained during the nurse visit, which were then analysed for haemoglobin. Haemoglobin dist ributes oxygen around the body, and low haemoglobin levels usually indicate anaemia. Various different thresholds for low haemoglobin have been used in the literature, particularly for older populations [27], but we here used the longstanding WHO definition of <13g/dL for men and <12g/dL for women [24].
Iron deficiency	In the years 1994, 1998, 2006, and 2009, blood samples were obtained during the nurse visit, which were then analysed for serum ferritin (which correlates directly with the amount of iron stored in the body). Iron deficiency is one of several possible causes of anaemia (alongside other nutritional deficiencies, genetic conditions such as sickle cell anaemia, infections, and blood loss). Iron deficiency is defined as a serum ferritin less than 45ng/ml [27].
Mental health	
Mental health LSI	Every year 1994-2011, people who report a longstanding illness (LSI) are then asked, *'what is the matter with you?'*; up to 6 responses are then coded by the interviewer into a consistent coding frame based on the International Classification of Diseases.<br>The mental health LSI measure is based on the group labelled *'Mental illness/anxiety/depression/nerves (nes)'*, which as of 2011 includes: Alcoholism, recovered not cured alcoholic; Angelman Syndrome; Anorexia nervosa; Anxiety, panic attacks; Asperger Syndrome; Autism/Autistic (BBG: changed from 'autistic child'); Bipolar Affective Disorder; Catalepsy; Concussion syndrome; Depression; Drug addict; Dyslexia; Hyperactive child.; Nerves (nes); Nervous breakdown, neurasthenia, nervous trouble; Phobias; Schizophrenia, manic depressive; Senile dementia, forgetfulness, gets confused; Speech impediment, stammer; and Stress. It explicitly excludes Alzheimer's disease, degenerative brain disease.<br>While the LSI coding frame generally stays consistent over this period, it is worth being aware of a minor wording change within 'mental health LSI': the condition labelled 'Autistic child' 1994-1997 was relabelled 'Autism/Autistic' in 1998.
Psychological distress (GHQ)	In the self-completion survey in most years (except 1996, 2007, 2011 and 2013), respondents were asked the following series of questions:<br>- *"Please read this carefully: We should like to know how your health has been in general over the past few weeks. Please answer ALL the questions by ticking the box below the answer which you think most applies to you. Have you recently...*<br>- *"…been able to concentrate on whatever you're doing?" RESPONSES: "Better than usual" | "Same as usual" | "Less than usual" | "Much less than usual"*<br>- *"…lost much sleep over worry?" RESPONSES: "Not at all" | "No more than usual" | "Rather more than usual" | "Much more than usual""*

For peer review only - http://bmjopen.bmj.com/site/about/guidelines.xhtml

	- *"…felt you were playing a useful part in things?"* RESPONSES: *"More so than usual" | "Same as usual" | "Less useful than usual" | "Much less useful""*<br>- *"…felt capable of making decisions about things?"* RESPONSES: *"More so than usual" | "Same as usual" | "Less so than usual" | "Much less capable""*<br>- *"…felt constantly under strain?* RESPONSES: *"Not at all" | "No more than usual" | "Rather more than usual" | "Much more than usual""*<br>- *"..felt you couldn't overcome your difficulties?"* RESPONSES: *"Not at all" | "No more than usual" | "Rather more than usual" | "Much more than usual""*<br>- *"…been able to enjoy your normal day-to-day activities?"* RESPONSES: *"More so than usual" | "Same as usual" | "Less so than usual" | "Much less than usual"*<br>- *"…been able to face up to your problems?"* RESPONSES: *"More so than usual" | "Same as usual" | "Less able than usual" | "Much less able"*<br>- *"…been feeling unhappy and depressed?* RESPONSES: *"Not at all" | "No more than usual" | "Rather more than usual" | "Much more than usual"*<br>- *"…been losing confidence in yourself?* RESPONSES: *"Not at all" | "No more than usual" | "Rather more than usual" | "Much more than usual"*<br>- *"…been thinking of yourself as a worthless person?"* RESPONSES: *"Not at all" | "No more than usual" | "Rather more than usual" | "Much more than usual""*<br>- *"…been feeling reasonably happy, all things considered?"* RESPONSES: *"More so than usual" | "Same as usual" | "Less so than usual" | "Much less happy"*<br>These make up the 12-item General Health Questionnaire GHQ-12; [28], a well-validated, widely-used measure of probable mental ill-health. This is often termed general non-psychotic psychiatric morbidity, but I here use the more easily understood term 'psychological distress' following Stochl et al 2016.[29]<br>A total score has been created by first ensuring that all questions were coded from 1 (positive symptom) to 4 (negative symptom), and then creating a sum score for all the number of questions in which people answered with categories 3 or 4 (indicating a negative symptom). A binary measure (often called GHQ caseness) was created for people who had negative symptoms for 4 or more of the 12 questions.
Anxiety/depression *(moderately / Extremely)*	In the self-completion survey in 1996, 2003-6, 2008, 2010-12 and 2014, respondents were asked *'Now we would like to know how your health is today. Please answer ALL the questions. By ticking one box for each question below, please indicate which statements best describe your own health state today'*:<br>- *"I am not anxious or depressed"*<br>- *"I am moderately anxious or depressed"*<br>- *"I am extremely anxious or depressed"*<br>[This is part of the widely-used EQ-5D health status indicator [8]. However, for the purposes of this paper we have separated the individual measures that make up the EQ-5D in order to compare these to similar indicators of morbidity within each domain].<br>Two outcome measures are based on this: whether people have any anxiety/depression (the 2nd and 3rd categories combined), and whether they have extreme anxiety/depression (3rd category only).
Communication	
Hearing, seeing & communication limitations	These measures were not included in the main paper due to the short time frame that we can examine trends over, but are included in the Web Appendix as they relate to important domains of morbidity.<br>They were included in the disability scale used in the 2001 HSE report [9]. Respondents in 1995, 2000 and 2001 were asked if of the following applied to them (interviewers were instructed to ignore temporary disabilities that are expected to last less than one year):<br>• *"Cannot follow a TV programme at a volume others find acceptable (with hearing aid if normally worn)"* ('hearing limitation')<br>• *"Cannot see well enough to recognise a friend across a road (four yards away) (with glasses or contact lenses if normally worn)"* ('seeing limitation')<br>• *"Have problem communicating with other people - that is, have problem*

For peer review only - http://bmjopen.bmj.com/site/about/guidelines.xhtml

	understanding them or being understood by them" ('communication limitation')
Eye/Ear LSI	Every year 1994-2011, people who report a longstanding illness (LSI) are then asked, *'what is the matter with you?'*; up to 6 responses are then coded by the interviewer into a consistent coding frame based on the International Classification of Diseases. The Eye/Ear LSI includes the following groups:<br>• *Poor hearing/deafness*, including Conductive/nerve/noise induced deafness, Deaf mute/deaf and dumb, Heard of hearing, slightly deaf, Otosclerosis, Poor hearing after mastoid operation.<br>• *Tinnitus/noises in the ear*, Incl. pulsing in the ear<br>• *Other ear complaints*, Incl. otitis media - glue ear, Disorders of Eustachian tube, Perforated ear drum (nes), Middle/inner ear problems, Mastoiditis, Ear trouble (nes),, Ear problem (wax), Ear aches and discharges, Ear infection<br>• *Cataract/poor eye sight/blindness*, Incl. operation for cataracts, now need glasses, Bad eyesight, restricted vision, partially sighted, Bad eyesight/nearly blind because of cataracts, Blind in one eye, loss of one eye, Blindness caused by diabetes, Blurred vision, Detached/scarred retina, Hardening of lens, Lens implants in both eyes, Short sighted, long sighted, myopia, Trouble with eyes (nes), eyes not good (nes), Tunnel vision<br>• *Other eye complaints*, including Astigmatism, Buphthalmos, Colour blind, Double vision, Dry eye syndrome, trouble with tear ducts, watery eyes, Eye infection, conjunctivitis, Eyes are light sensitive, Floater in eye, Glaucoma, Haemorrhage behind eye, Injury to eye, Iritis, Keratoconus, Night blindness, Retinitis pigmentosa, Scarred cornea, corneal ulcers, Squint, lazy eye, Sty on eye.

Changes over time in several other measures are only presented in Web Appendices 4 & 6, rather than the main paper. Details of these variables are included below:

**Measure**	**Details**
General health	
General health<br>*(bad / good)*	Every year, respondents were asked, *"How is your health in general? Would you say it was … very good, good, fair, bad, or very bad?"*<br>Two outcome measures are based on this, following standard practice in the HSE reports: bad general health (which includes 'bad' or 'very bad' health) and good general health (which includes 'good' or 'very good' health).
Longstanding illness (LSI)	Every year 1994-2011, respondents were asked *"Do you have any long-standing illness, disability or infirmity? By long-standing I mean anything that has troubled you over a period of time, or that is likely to affect you over a period of time?"* (The response options were 'Yes' and 'No').<br>In 2012 the question was changed to be consistent with the Government's new harmonised disability questions for use in social surveys [30], and is not comparable to the previous version.
Limiting LSI	Every year 1996-2011, respondents who said they had an LSI were than asked, *"Does this illness or disability (do any of these illnesses or disabilities) limit your activities in any way?"* (again allowing only Yes/No answers).<br>In 2012 the question was changed to be consistent with the Government's new harmonised disability questions for use in social surveys (see HSE 2012 report), and is not comparable to the previous version.
Problems with usual activities<br>*(some problems / unable)*	In the self-completion survey in 1996, 2003-6, 2008, 2010-12 and 2014, respondents were asked *'Now we would like to know how your health is today. Please answer ALL the questions. By ticking one box for each question below, please indicate which statements best describe your own health state today'*:<br>- *"I have no problems with performing my usual activities (e.g. work, study, housework,*

For peer review only - http://bmjopen.bmj.com/site/about/guidelines.xhtml

	family or leisure activities)"<br>- *"I have some problems with performing my usual activities"*<br>- *"I am unable to perform my usual activities"*<br>[This is part of the widely-used EQ-5D health status indicator [8]. However, for the purposes of this paper we have separated the individual measures that make up the EQ-5D in order to compare these to similar indicators of morbidity within each domain].<br>Two outcome measures are based on this: whether people have any problems (the 2nd and 3rd categories combined), and whether they are unable to perform their usual activities (3rd category only).
Limitations in past 2wks	Every year, respondents were asked, *"Now I'd like you to think about the two weeks ending yesterday. During those 2 weeks did you have to cut down on any of the things you usually do (about the house or at work or in your free time) because of your answer at <the LSI question> or some other illness or injury?"*<br>There have been two small changes to this question's wording in 1996. Firstly, 'work' was changed to 'work/school'. Secondly, *'your answer at <the LSI question>'* was changed to *'a condition you have just told me about'*. While it is impossible to be sure of the exact effect of these changes, neither seem likely to influence the results (at least for the 25+ age group where fewer individuals are in full-time education).

For peer review only

For peer review only - http://bmjopen.bmj.com/site/about/guidelines.xhtml

Appendix 6: Measures not included in the main paper

Trends in several measures are not included in the main paper, either

Table 10: Changes over time in measures not included in the main paper

	Starting period		Change from start to end period			
	Period	Prevalence	End period	Raw change	Adj.[a] change	Adj. change 95% CI
CVD						
Component measures nec[b]						
Recent heart murmur	1994-96	0.8%	2011-14	0.1%	0.0%	[-0.3, 0.4%]
Recent irregular heart rhythm	1994-96	1.6%	2011-14	0.4%	0.4%	[-0.1, 0.8%]
Recent other heart disease	1994-96	0.2%	2011-14	0.7%	0.7%	[0.4, 0.9%]
Ever had (not just recent)						
Ever had high BP	1994-96	19.0%	2011-14	4.5%	3.7%	[2.3, 5.1%]
DD high BP	1994-96	13.2%	2011-14	6.9%	6.0%	[4.7, 7.3%]
Ever IHD or stroke	1994-96	2.9%	2011-14	0.3%	-0.0%	[-0.6, 0.6%]
DD IHD or stroke	1994-96	2.5%	2011-14	0.5%	0.2%	[-0.3, 0.7%]
Ever had angina	1994-96	1.9%	2011-14	-0.2%	-0.4%	[-0.9, 0.0%]
Ever DD angina	1994-96	1.6%	2011-14	-0.1%	-0.3%	[-0.7, 0.1%]
Ever heart murmur	1994-96	3.2%	2011-14	-0.3%	-0.3%	[-0.9, 0.3%]
DD heart murmur	1994-96	2.6%	2011-14	-0.2%	-0.2%	[-0.7, 0.3%]
Ever irregular heart rhythm	1994-96	6.4%	2011-14	-0.7%	-0.9%	[-1.7, -0.1%]
DD irregular heart rhythm	1994-96	3.5%	2011-14	0.5%	0.3%	[-0.3, 1.0%]
Ever other heart disease	1994-96	0.9%	2011-14	1.1%	1.0%	[0.6, 1.5%]
DD other heart disease	1994-96	0.8%	2011-14	1.0%	1.0%	[0.6, 1.4%]
Respiratory						
**Alternate measures**						
Phlegm symptoms	1994-96	9.1%	2008-10	-1.3%	-1.4%	[-2.3, -0.5%]
LSI Respiratory All	1994-96	7.9%	2011-14	-0.7%	-0.7%	[-1.6, 0.1%]
**Ever had (not just recent)**						
Wheezing Ever	1994-96	32.3%	2008-10	0.0%	-0.1%	[-1.8, 1.5%]
Wheezing Past 12mths	1994-96	18.9%	2008-10	-1.0%	-1.1%	[-2.3, 0.2%]
Diabetes						
**Ever had (not just recent)**						
Ever diabetes	1994-96	2.0%	2011-14	2.9%	2.8%	[2.3, 3.2%]
DD diabetes	1994-96	1.7%	2011-14	2.5%	2.3%	[2.0, 2.7%]
Mental health						
**Alternate measures**						
High psychological distress	1994-96	3.2%	2011-14	1.0%	0.9%	[0.4, 1.4%]
Activity limitations & musculoskeletal						
**For comparison**						
Walking limitation	1994-96	4.6%	2001-03	1.4%	1.2%	[0.5, 1.9%]
Washing/dressing limitation	1994-96	1.9%	2001-03	0.5%	0.4%	[0.0, 0.8%]
Other LSIs						

For peer review only - http://bmjopen.bmj.com/site/about/guidelines.xhtml

	Starting period		Change from start to end period			
	Period	Prevalence	End period	Raw change	Adj.[a] change	Adj. change 95% CI
LSI Blood Disorders	1994-96	0.3%	2011-14	0.6%	0.5%	[0.3, 0.8%]
LSI Cancer	1994-96	1.0%	2011-14	0.3%	0.3%	[-0.1, 0.6%]
LSI D,GUM,E&M	1994-96	6.9%	2011-14	1.1%	0.8%	[0.0, 1.6%]
LSI Epilepsy	1994-96	0.7%	2011-14	0.1%	0.1%	[-0.2, 0.3%]
LSI Nervous System	1994-96	3.7%	2011-14	-0.2%	-0.3%	[-0.8, 0.3%]

[a] 'Adj.' = trend adjusted for changing age and sex distribution of the working-age population. [b] 'nec' = not elsewhere included.

The details of these measures are as follows:

Measure	Details
Circulatory	
Beyond 'recent': 'Ever had' and 'DD' CVD	In the main paper, we look at whether people report recent doctor-diagnosed CVD (looking separately at heart attack/stroke, angina, and any recent CVD). As shown above, this comes from three questions: whether people report ever having this condition; whether a doctor diagnosed this; and whether they have had an attack in the past 12 months / consider themselves to still have the condition.<br>Web Appendix 6 shows trends in the other versions of these measures, i.e. having ever had this type of CVD, and having ever doctor-diagnosed ('DD') CVD of this type.
Component measure: Heart murmur<br>Irregular heart rhythm<br>Other heart disease	In the main paper, we recent reports of doctor-diagnosed angina; heart attack (including myocardial infarction or coronary thrombosis); a heart murmur; abnormal heart rhythm; or other heart trouble (see above). Angina and heart attack are also analysed in the main paper in their own right; in Web Appendix 6, we further show trends separately in heart murmur, abnormal heart rhythm or other heart trouble.
Respiratory	
Component measure: 'phlegm'	In the main paper, we look at whether people report recent COPD (see above). This combines two measures: regular cough + phlegm. Web Appendix 6 shows the trend in the phlegm measure on its own, without being combined with a regular cough.
Alternative version: 'LSI respiratory'	In the main paper, we look at whether an asthma LSI (to examine alongside a direct question on diagnosed asthma); see above. Web Appendix 6 also shows people reporting a longstanding illness ('LSI') which is included within the broader category of respiratory conditions.<br>The respiratory LSI measure is based on the group labelled *'Asthma'*, *'Bronchitis'*, *'Hayfever'*, or *'Respiratory other'*, which as of 2011 includes:<br>Asthma: Asthma; Bronchial asthma, allergic asthma; and Asthma - allergy to house dust/grass/cat fur. It explicitly excludes cardiac asthma.<br>Hayfever: Hayfever, Allergic rhinitis<br>Bronchitis/emphysema: Bronchitis/emphysema, Bronchiectasis, Chronic bronchitis.<br>Other respiratory complaints: Other respiratory complaints, Abscess on larynx, Adenoid problems, nasal polyps, Allergy to dust/cat fur, Bad chest (nes), weak chest – wheezy, Breathlessness, Bronchial trouble, chest trouble (nes), Catarrh, Chest infections, get a lot of colds, Churg-Strauss syndrome, Chronic Obstructive Pulmonary Disease (COPD), Coughing fits, Croup, Damaged lung (nes), lost lower lobe of left lung, Fibrosis of lung, Furred up airways, collapsed lung, Lung complaint (nes), lung problems (nes), Lung damage by viral pneumonia, Paralysis of vocal cords, Pigeon fancier's lung, Pneumoconiosis, byssinosis, asbestosis and other industrial respiratory disease, Recurrent pleurisy, Rhinitis (nes), Sinus trouble, sinusitis, Sore throat, pharyngitis, Throat

For peer review only - http://bmjopen.bmj.com/site/about/guidelines.xhtml

Measure	Details
	infection, Throat trouble (nes), throat irritation, Tonsillitis, Ulcer on lung, fluid on lung. Note that:<br>• It explicitly excludes TB (pulmonary tuberculosis), Cystic fibrosis, Skin allergy, Food allergy, Allergy (nes), Pilonidal sinus, Sick sinus syndrome, Whooping cough.<br>• If complaint is breathlessness with the cause also stated, this is coded with the cause – hence it also excludes breathlessness as a result of anaemia, breathlessness due to hole in heart, and breathlessness due to angina.
Component measure: Wheezing	In the main paper, we look at whether people report recent wheezing/asthma. As shown above, this comes from three questions: whether people report ever having had wheezing or whistling in the chest; whether they have had this in the past 12 months; and whether they have had an asthma attack in the past 12 months.<br>Web Appendix 6 shows trends in the other versions of these measures, i.e. having ever had wheezing/whistling in the chest, and whether they have had this in the past 1 months.
Beyond 'recent': 'Ever had' and 'DD' diabetes	In the main paper, we look at whether people report recent doctor-diagnosed diabetes As shown above, this comes from three questions: whether people report ever having this condition; whether a doctor diagnosed this; and whether they currently inject insulin / take other medication for diabetes.<br>Web Appendix 6 shows trends in the other versions of these measures, i.e. having ever had diabetes, and having ever doctor-diagnosed ('DD') diabetes.
Activity limitations	
For comparison: Walking limitation	This is based on the personal care disability scale used in the 2001 HSE report [9]. Respondents in 1995, 2000 and 2001 were asked if of the following applied to them (interviewers were instructed to ignore temporary disabilities that are expected to last less than one year): *"Cannot walk 200 yards or more on own without stopping or discomfort"*. People who reported a limitation were asked if they used a walking aid, and if they did, were then asked if they could walk 200 yards without the walking aid.
For comparison: Washing & dressing limitation	This is based on the personal care disability scale used in the 2001 HSE report [9]. Respondents in 1995, 2000 and 2001 were asked if any of the following applied to them (interviewers were instructed to ignore temporary disabilities that are expected to last less than one year):<br>- *"Cannot dress and undress without difficulty"*<br>- *"Cannot wash hands and face without difficulty"*<br>For comparison to the 'problems with washing/dressing today' measure in the main paper (which covers a more extended period and is based on a different question; see above), a measure is derived if respondents say they report either of these problems.
Other LSIs	
Other LSIs	Every year 1994-2011, people who report a longstanding illness (LSI) are then asked, *'what is the matter with you?'*; up to 6 responses are then coded by the interviewer into a consistent coding frame based on the International Classification of Diseases. The various other LSIs are as follows:<br>• The Blood Disorders LSI measure is based on the group *'Disorders of blood and blood forming organs and immunity disorders'*, which as of 2011 includes: Anaemia, pernicious anaemia, Blood condition (nes), blood deficiency, Haemophilia, Idiopathic Thrombochopenic Purpura (ITP), Immunodeficiences, Polycthaemia (blood thickening), blood to thick, Purpura (nes), Removal of spleen, Sarcoidosis (previously code 37), Sickle cell anaemia/disease, Thalassaemia, Thrombocythenia. It explicitly excludes Leukaemia - code 01.<br>• The Cancer LSI measure is based on the group *'Cancer (neoplasm) including lumps, masses, tumours and growths and benign (non-malignant) lumps and cysts'*, which as of

For peer review only - http://bmjopen.bmj.com/site/about/guidelines.xhtml

Measure	Details
	2011 includes: Acoustic neuroma, After effect of cancer (nes), All tumours, growths, masses, lumps and cysts, whether malignant or benign eg. tumour on brain,, growth in bowel, growth on spinal cord, lump in, breast, Cancers sited in any part of the body or system eg., Lung, breast, stomach, Colostomy caused by cancer, Cyst on eye, cyst in kidney., General arthroma, Hereditary cancer, Hodgkin's disease, Hysterectomy for cancer of womb, Inch. leukaemia (cancer of the blood), Lymphoma, Mastectomy (nes), Neurofibromatosis, Part of intestines removed (cancer), Pituitary gland removed (cancer), Rodent ulcers, Sarcomas, carcinomas, Skin cancer, bone cancer, Wilms tumour<br>• The D,GUM,E&M (Digestive, Genitourinary Medicine, and Endocrine & Metabolic) LSI is based on the groups, *'Complaints of bowel/colon (large intestine,caecum, bowel, colon, rectum)'* (including Colitis, colon trouble, ulcerative colitis, Coleliac, Colostomy (nes), Crohn's disease, Diverticulitis, Enteritis, Faecal incontinence/encopresis., Frequent diarrhoea, constipation, Grumbling appendix, Hirschsprung's disease, Irritable bowel, inflammation of bowel, Polyp on bowel, Spastic colon, but explicitly excluding piles and Cancer of stomach/bowel), *Other digestive complaints (stomach, liver, pancreas, bile ducts, small intestine - duodenum, jejunum and ileum)* (including Cirrhosis of the liver, liver problems, Food allergies, Ileostomy, Indigestion, heart burn, dyspepsia, Inflamed duodenum, Liver disease, biliary artesia, Nervous stomach, acid stomach, Pancreas problems, Stomach trouble (nes), abdominal trouble (nes), Stone in gallbladder, gallbladder problems, Throat trouble - difficulty in swallowing, Weakness in intestines), *Stomach ulcer/ulcer (nes)/abdominal hernia/rupture* (including Double/inguinal/diaphragm/hiatus/umbilical hernia, Gastric/duodenal/peptic ulcer, Hernia (nes), rupture (nes), Ulcer (nes)), *Complaints of teeth/mouth/tongue* (including Cleft palate, hare lip, Impacted wisdom tooth, gingivitis, No sense of taste, Ulcers on tongue, mouth ulcers), *Other endocrine/metabolic* (including Addison's disease, Beckwith - Wiedemann syndrome, Coeliac disease, Cushing's syndrome, Cystic fibrosis, Gilbert's syndrome, Hormone deficiency, deficiency of growth hormone,, dwarfism, Hypercalcemia, Hypopotassaemia, lack of potassium, Malacia, Myxoedema (nes), Obesity/overweight, Phenylketonuria, Rickets, Too much cholesterol in blood, Underactive/overactive thyroid, goitre, Water/fluid retention, Wilson's disease, but explicitly excluding Thyroid trouble and tiredness and Overactive thyroid and swelling in neck, *Other bladder problems/incontinence* (including Bed wetting, enuresis, Bladder restriction, Water trouble (nes), Weak bladder, bladder complaint (nes), but explicitly excluding Prostate trouble), *Kidney complaints* (including Chronic renal failure, Horseshoe kidney, cystic kidney, Kidney trouble, tube damage, stone in the kidney, Nephritis, pyelonephritis, Nephrotic syndrome, Only one kidney, double kidney on right side, Renal TB, Uraemia), *Reproductive system disorders* (including Abscess on breast, mastitis, cracked nipple, Amenorrhea, Damaged testicles, Endometriosis, Gynaecological problems, Hysterectomy (nes), Impotence, infertility, Menopause, Pelvic inflammatory disease/PID (female), Period problems, flooding, pre-menstrual tension/syndrome, Prolapse (nes) if female, Prolapsed womb, Prostrate gland trouble, Turner's syndrome, Vaginitis, vulvitis, dysmenorrhoea) and *Urinary tract infection* (including Cystitis, urine infection).<br>• The Epilepsy LSI is based on the group, *'Epilepsy/fits/convulsion',* including Grand mal, Petit mal, Jacksonian fit, Lennox-Gastaut syndrome, blackouts, febrile convulsions, fit (nes)<br>• The Nervous System LSI is based on the groups:<br>○ *Migraine/headaches*<br>○ *Other problems of nervous system*, including Abscess on brain, Alzheimer's

For peer review only - http://bmjopen.bmj.com/site/about/guidelines.xhtml

Measure	Details
	disease, Bell's palsy, Brain damage resulting from infection (eg. meningitis,, encephalitis) or injury, Carpal tunnel syndrome, Cerebral palsy (spastic), Degenerative brain disease, Fibromyalgia, Friedreich's Ataxia, Guillain-Barre syndrome, Huntington's chorea, Hydrocephalus, microcephaly, fluid on brain, Injury to spine resulting in paralysis, Metachromatic leucodystrophy, Motor neurone disease, Multiple Sclerosis (MS), disseminated sclerosis, Muscular dystrophy, Myalgic encephalomyelitis (ME), Myasthenia gravis, Myotonic dystrophy, Neuralgia, neuritis, Numbness/loss of feeling in fingers, hand, leg etc, Paraplegia (paralysis of lower limbs), Parkinson's disease (paralysis agitans), Partially paralysed (nes), Physically handicapped - spasticity of all limbs, Pins and needles in arm, Post viral syndrome (ME), Removal of nerve in arm, Restless legs, Sciatica, Shingles, Spina bifida, Syringomyelia, Trapped nerve, Trigeminal neuralgia, Teraplegia"<br>○ *Meniere's disease/ear complaints causing balance problems* (including Labryrinthitis,, loss of balance - inner ear, Vertigo).

peer review only

For peer review only - http://bmjopen.bmj.com/site/about/guidelines.xhtml

Appendix 7: Year-by-year trends

This appendix presents the year-by-year trends for all of the variables included in the main paper. The table row labelled 'start v end sig' presents the p-value for testing the null hypothesis that there is no difference between the first and last years in the series (whichever these years are). Note that this will differ from the confidence intervals presented in the main paper as these are grouped into multi-year periods with larger sample sizes and therefore greater precision.

Table 11: Year-to-year trends in cardiovascular health

	High blood pressure LSI	Recent high blood pressure	*Biomarker high blood pressure*	Recent heart attack/stroke	IHD/stroke LSI	Heart attack symptoms	Mini stroke (TIA) symptoms	Recent angina	*Angina symptoms*
**1994**	2.2%	4.2%	8.4%	1.2%	1.4%	5.5%		1.1%	2.3%
**1995**	2.9%		8.3%		1.5%				
**1996**	3.0%		8.3%		1.5%				
**1997**	3.8%		7.7%		1.4%				
**1998**	3.1%	5.4%	7.0%	1.5%	1.3%	6.5%		1.4%	2.2%
**1999**	3.4%				1.4%				
**2000**	4.0%		6.5%		1.3%				
**2001**	4.5%		7.3%		1.7%				
**2002**	4.3%		6.1%		1.4%				
**2003**	4.5%	7.9%	4.9%	1.3%	1.3%	5.5%	8.1%	1.0%	1.8%
**2004**	4.0%				1.2%				
**2005**	5.0%		4.4%		1.3%				
**2006**	4.4%	8.7%	3.9%	1.1%	1.2%	6.2%	7.8%	0.9%	1.6%
**2007**	4.9%		4.5%		1.0%				
**2008**	5.1%		3.9%		1.1%				
**2009**	4.7%		3.2%		1.3%				
**2010**	4.6%		4.1%		1.1%				
**2011**	4.0%	9.5%	3.2%	1.0%	1.0%	5.2%	6.7%	0.7%	1.2%
**2012**			4.1%						
**2013**			3.7%						
**2014**			3.9%						
**Start v end sig.**	0.00	0.00	0.00	0.14	0.05	0.52	0.01	0.03	0.00
**N**	124,830	43,292	79,601	43,445	124,830	43,521	23,487	43,477	43,518

For peer review only - http://bmjopen.bmj.com/site/about/guidelines.xhtml

Table 12: Year-to-year trends in respiratory health

	COPD symptoms	Diagnosed asthma	Asthma LSI	Breathlessness-Grade 2+	Breathlessness-Grade 3	Recent wheezing/asthma	Wheezing stopping sleep
**1994**			4.7%				
**1995**	6.6%	10.8%	4.8%	19.1%	7.6%	19.8%	3.6%
**1996**	6.6%	11.5%	5.3%	20.3%	8.0%	19.3%	3.5%
**1997**		11.9%	6.0%			18.9%	3.7%
**1998**			5.3%				
**1999**			5.7%				
**2000**			5.5%				
**2001**		14.1%	5.9%			19.9%	3.4%
**2002**			6.0%				
**2003**			5.8%				
**2004**			6.3%				
**2005**			6.1%				
**2006**			5.8%				
**2007**			5.7%				
**2008**			6.2%				
**2009**			5.5%				
**2010**	5.1%	16.6%	6.0%	15.4%	6.4%	18.4%	3.2%
**2011**			5.6%				
**2012**							
**2013**							
**2014**							
**Start v end sig.**	0.00	0.00	0.02	0.00	0.01	0.05	0.18
**N**	25,631	41,219	124,830	25,620	25,620	41,218	41,218

For peer review only - http://bmjopen.bmj.com/site/about/guidelines.xhtml

Table 13: Year-to-year trends in activity limitations & musculoskeletal health

	Problems walking about today	Walking limitation	Any locomotor limitation	Problems washing/dressing today	Washing/dressing limitation	Any self-care limitation	Pain-any	Pain-extreme	Arthritis LSI	Other musculoskeletal LSI
**1994**									4.9%	8.9%
**1995**		4.6%	6.8%		1.9%	3.9%			5.4%	9.9%
**1996**	11.5%			3.4%			32.0%	3.0%	5.4%	10.3%
**1997**									6.0%	11.4%
**1998**									5.6%	11.7%
**1999**									5.5%	11.0%
**2000**		6.3%	8.2%		2.5%	5.2%			5.6%	10.7%
**2001**		5.9%	7.8%		2.4%	4.7%			6.1%	10.9%
**2002**									5.7%	12.3%
**2003**	11.8%			3.2%			27.1%	3.2%	6.2%	11.8%
**2004**	11.6%			3.6%			28.6%	3.5%	6.3%	11.6%
**2005**	12.3%			4.0%			27.8%	3.5%	6.0%	11.3%
**2006**	11.6%			3.6%			26.8%	3.1%	5.4%	10.1%
**2007**									5.4%	9.9%
**2008**	11.5%			3.6%			28.1%	3.1%	4.7%	9.5%
**2009**									5.2%	9.0%
**2010**	13.0%			4.1%			29.9%	3.2%	5.1%	10.3%
**2011**	13.6%			4.0%			34.0%	4.0%	4.9%	9.2%
**2012**	11.8%			3.8%			27.4%	3.1%		
**2013**										
**2014**	12.2%			4.2%			27.7%	3.0%		
**Start v end sig.**	0.30	0.00	0.01	0.05	0.04	0.01	0.00	0.89	0.97	0.57
**N**	62,680	25,341	25,341	62,612	25,341	25,341	62,692	62,692	124,830	124,830

w only

For peer review only - http://bmjopen.bmj.com/site/about/guidelines.xhtml

Table 14: Year-to-year trends in obesity & diabetes

	BMI-Underweight	BMI-Obese	High waist-hip ratio	Recent diabetes	Diabetes LSI	Glycated haemoglobin
**1994**	1.1%	15.7%	9.5%	1.2%	1.5%	
**1995**	1.1%	17.0%			1.6%	
**1996**	0.9%	17.9%			1.6%	
**1997**	0.9%	19.3%	12.1%		1.7%	
**1998**	1.0%	19.5%	11.3%	1.4%	1.5%	
**1999**	1.1%	20.1%	16.3%		1.9%	
**2000**	0.9%	21.5%			2.0%	
**2001**	0.9%	22.8%	15.8%		2.1%	
**2002**	1.0%	23.5%	16.5%		2.1%	
**2003**	0.9%	23.2%	18.7%	2.1%	2.4%	2.7%
**2004**	1.0%	24.3%			2.8%	
**2005**	0.8%	24.5%	21.6%		2.9%	
**2006**	0.8%	25.1%	20.7%	2.7%	2.9%	3.1%
**2007**	1.0%	25.3%	22.1%		3.4%	
**2008**	0.9%	25.3%	22.5%		2.9%	3.8%
**2009**	1.4%	24.3%	23.5%	3.4%	3.8%	4.3%
**2010**	1.1%	27.8%	24.3%	3.4%	3.5%	3.7%
**2011**	0.8%	25.4%	24.3%	3.6%	3.8%	5.5%
**2012**	1.1%	25.6%	24.0%	3.6%		4.9%
**2013**	1.0%	26.8%	24.2%	3.6%		4.8%
**2014**	0.8%	27.1%	24.7%	3.7%		4.4%
**Start v end sig.**	1.1%	15.7%	9.5%	1.2%	1.5%	
**N**	1.1%	17.0%			1.6%	

For peer review only - http://bmjopen.bmj.com/site/about/guidelines.xhtml

Table 15: Year-to-year trends in other biomarkers

	High total cholesterol	Low HDL cholesterol	Raised C-reactive protein	Raised fibrinogen	Anaemia	Iron deficiency	Cancer LSI
**1994**	75.7%				6.7%	39.9%	0.2%
**1995**							0.3%
**1996**							0.3%
**1997**							0.4%
**1998**	64.8%	11.8%	21.4%	2.3%	6.3%	38.2%	0.5%
**1999**							0.4%
**2000**							0.5%
**2001**							0.5%
**2002**							0.5%
**2003**	71.4%	4.0%	24.1%	5.7%			0.6%
**2004**							0.6%
**2005**							0.6%
**2006**	67.2%	5.1%	22.7%	5.7%	4.6%	29.3%	0.7%
**2007**							0.5%
**2008**	66.7%	4.3%					0.6%
**2009**	66.9%	4.5%	23.5%	3.8%	5.3%	27.0%	0.5%
**2010**	64.1%	4.6%					0.8%
**2011**	60.2%	4.5%					0.8%
**2012**	64.0%	4.4%					
**2013**	58.0%	3.4%					
**2014**	55.4%	2.9%					
**Start v end sig.**	0.00	0.00	0.11	0.01	0.04	0.00	0.00
**N**	41,224	33,937	17,749	16,105	20,228	20,304	124,830

For peer review only - http://bmjopen.bmj.com/site/about/guidelines.xhtml

Table 16: Year-to-year trends in mental health

	Mental health LSI	Psychological distress symptoms	Anxiety/depression -moderately	Anxiety/depression -extremely
**1994**	1.8%	16.1%		
**1995**	2.3%	18.0%		
**1996**	2.4%		21.9%	1.8%
**1997**	2.9%	16.5%		
**1998**	3.0%	15.6%		
**1999**	3.0%	17.7%		
**2000**	3.5%	14.4%		
**2001**	3.3%	13.7%		
**2002**	3.1%	16.6%		
**2003**	3.7%	13.5%	18.5%	1.9%
**2004**	3.6%	13.4%	18.8%	2.1%
**2005**	4.4%	14.0%	19.6%	2.1%
**2006**	4.1%	13.9%	18.8%	2.1%
**2007**	4.5%			
**2008**	4.2%	13.7%	18.5%	2.0%
**2009**	4.9%	17.1%		
**2010**	5.2%	16.1%	23.5%	2.7%
**2011**	4.6%		26.8%	3.0%
**2012**		16.0%	20.0%	2.7%
**2013**				
**2014**		15.6%	19.6%	2.5%
**Start v end sig.**	0.00	0.47	0.01	0.02
**N**	124,830	107,834	62,635	62,635

For peer review only - http://bmjopen.bmj.com/site/about/guidelines.xhtml

Appendix 8: Others' analyses over change over time using HSE data

Changes over time in some of these indicators have not previously been analysed (e.g. waist-hip ratio, fibrinogen). However, others have been studied but never integrated into a single picture of changing morbidity; we review these in this section. (For reasons of space these are included here rather than in the main text).

Cardiovascular morbidity

1998-2011 trends in the two biomarkers for total and HDL cholesterol using HSE data are shown in Oyebode,[11] who find similar results.

Respiratory morbidity

A subset of the HSE respiratory indicators (ever/past year wheezing, doctor-diagnosed asthma) were analysed by Hall and Mindell[31] looking at 2001-2010, and finding similar changes over time to our analysis. They found stability in some measures (ever wheezing) but improvements in others (past-year wheezing) – at the same time as the reported prevalence of doctor-diagnosed asthma increased.

Obesity & diabetes

While the English trends in waist-hip ratio have not previously been analysed, earlier Scottish trends are given in Hotchkiss et al 2012.[19] Trends in diabetes have been covered in several HSE reports, e.g. Moody 2012,[20] as has BMI (see particularly the paper by Sperrin et al 2014,[32] who also created a publicly-available time-series HSE dataset for this purpose).

Activity limitations, pain & musculoskeletal morbidity

While musculoskeletal LSIs have not previously been analysed in HSE, a decline can also be seen in the General Household Survey.[33]

Mental health

In the UK and most other high-income countries, benefit claims due to mental ill-health have been rising,[34] which has come alongside considerable increases in mental health diagnosis and treatment.[35] The extent to which this reflects rises in mental ill-health and genuinely declining work capacity, however, has long been the subject of debate.[36 37] Perhaps the most robust long-term general population data series in the UK is the Adult Psychiatric Morbidity Survey.[35 38]

While some studies have used HSE to show rises in mental ill-health, others have used the same data to come to the opposite conclusion.[39 40] These contrasting conclusions are explained by the tables in Web Appendix 7 which show year-by-year changes: moderate mental ill-health fell between the mid-1990s and the mid-2000s, before rising in 2009, and with a particularly high prevalence in 2011. The conclusions of studies will therefore depend on the years they use as their start and end periods for the trend analysis.[3] It is also worth noting that our results for considerable increases in mental health LSIs can also be seen in a similar measure in the Labour Force Survey.[41 42]

[3] The major explanation why 'moderate anxiety/depression today' does not show a decline 2011-14 compared to 1994-6 is because of a single very high reported prevalence in 2011, which had reduced by 2012 and 2014. The alternate measure ('psychological distress symptoms') was not asked in 2011.

For peer review only - http://bmjopen.bmj.com/site/about/guidelines.xhtml

Other morbidity measures

While CRP and fibrinogen are collected in HSE at considerable efforts, their trends have rarely been studied (e.g. they appear only in supplementary descriptive tables in Hughes et al [23]). A decline in anaemia using HSE data 1998-2005 has been observed by Tull et al 2009,[43] but this has not hitherto been updated to the 2008-10 period.

It has been suggested that multimorbidity has risen among older people in England [44] and for all age groups in Ontario,[45] although others have cautioned against using simple disease counts,[46] and the evidence cited in the introduction of the main paper suggests that rising chronic disease reporting may partly be a result of increasing awareness (rather than underlying prevalence) of disease.

For peer review only

For peer review only - http://bmjopen.bmj.com/site/about/guidelines.xhtml

Appendix 9: Summarising multiple measures

Having reviewed trends in 39 morbidity measures, we have seen that morbidity in the English working-age population has improved in some respects and deteriorated in others. For those who view work-related morbidity as intrinsically multidimensional,[47], this is the endpoint of our analysis. However, for those who conceive of morbidity as unidimensional – or those who are interested in morbidity as it relates to a unidimensional work capacity – this raises the question of how we weight different dimensions of morbidity to decide if the overall change in morbidity has been positive or negative.

Methods for creating unidimensional morbidity scales

Several methods have been proposed for creating unidimensional morbidity scales, but most of these are unavailable using the HSE data:

- Weights can be based on empirically-derived preferences for different health states, of which the most famous example is the WHO Global Burden of Disease (GBD) study [48]. Some GBD estimates for trends in disability in the UK do exist, and suggest that the prevalence of disability in the working-age population is unchanged 1990-2010, though these results are only presented in passing.[4] For our analyses, however, we have no preference-based weights for most of the HSE measures (excluding the subset of measures that make up the EQ-5D scale).
- Those reporting limitations beyond a certain severity in any domain can be categorised as 'disabled', as recommended by the Washington Group on Disability Statistics (see above). However, as previously discussed, we have few functional limitations measures available in HSE.
- Latent morbidity scales can be created based on the inter-correlations between different measures (using item response theory), as used in the World Disability Report [51] and by researchers associated with the US National Bureau of Economic Research e.g. [52]. However, it is unclear why we would wish to weight items in this way: a given morbidity indicator may be severe, yet if it is unrelated to other morbidity measures it will be given a low weight.
- Latent morbidity scales can also be created based on the independent correlation between each indicator and a general measure of morbidity, such as general self-reported health or [53] as in [54]. This maintains some of the advantages of single-item measures (in providing a basis for making morbidity unidimensional), while avoiding the potential threats to validity discussed above. However, the inconsistent inclusion of measures in each HSE wave prevents a unidimensional morbidity scale being constructed here.

---

[4] Trends in the UK GBD results are reported in Murray et al.[49] However, Murray et al do not focus on trends in years lived with disability (YLD), other than to note that *"YLDs per person by age and sex have not changed substantially in the UK, but age-specific mortality has been improving"* (p1005). The figure in the supplementary appendix shows that YLDs have barely changed for either men or women at any age. However, the confidence intervals for YLDs as a whole in the main paper (Table 1) suggest that the confidence intervals for these trends are very wide. The public GBD data [50] do provide cause-disaggregated YLDs for the UK (and all other countries) for a slightly different period (2000-2015), but are not age-standardised, are within broad age groups only (e.g. 15-29), and again lack estimates of uncertainty.

For peer review only - http://bmjopen.bmj.com/site/about/guidelines.xhtml

An alternative way of summarising heterogeneous trends

Nevertheless, we can examine if the areas in which morbidity has been improving or declining are those that are particularly important for general health.[53] (This uses the same intuition as the scales in Diederichs et al 2012).[54] To see how important measures are for general health, we regress 'bad' general health (see Appendix 5 for detail on the underlying question) on age, sex (and their interaction), educational level and each individual morbidity measure in turn, using all years for which that morbidity measure is available. That is, for each morbidity indicator morbidity_i^a we use the following model:

$$\text{badhealth}_i = \text{logit}\,[\,\beta_1 \text{morbidity}_i^a + \boldsymbol{\beta_2 \text{age}_i} + \beta_3 male_i + \boldsymbol{\beta_4}(\textbf{age}_\textbf{i} * \text{male}_\text{i}) + \ \boldsymbol{\beta_5 \text{education}_i}]$$

… where β_1 is our primary outcome coefficient showing the importance of that morbidity indicator for bad health, $\textbf{age}_\textbf{i}$ refers to a vector of age dummy variables, male_i refers to a binary gender dummy variable, $\textbf{education}_\textbf{i}$ refers to a vector of education dummy variables (with four levels: degree/full-time student, A-levels/NVQ3/higher education below degree, other qualifications, or no qualifications), and $\boldsymbol{\beta_2}$, β_3, $\boldsymbol{\beta_4}$, and $\boldsymbol{\beta_5}$ refer to the coefficients on age, gender, their interaction and education respectively.

We adjust for education as well as age & sex to enable us to examine the importance of the measure for bad health, after taking account of whether general health and the measure are both strongly related to social status. Note however that it is not possible to control for all morbidity measures simultaneously (as we discuss just above) – so this is a rough indicator of the importance of that morbidity measure for general health, rather than a reliable indicator of the causal impact net of co-morbidities.

The results of this analysis are shown overleaf, ordered by the effect on bad health. (We also repeat the trend in each measure for convenience; this is discussed following the table).

For peer review only

For peer review only - http://bmjopen.bmj.com/site/about/guidelines.xhtml

Measure	Type	Effect on bad health *(95% CI)*		Change over time in measure *(95% CI)*	
Pain-extreme	S	46.4%	*[44.0, 48.9%]*	0.2%	*[-0.3, 0.7%]*
Problems washing/dressing today	S	43.7%	*[41.4, 46.0%]*	0.3%	*[-0.2, 0.9%]*
Anxiety/depression-extremely	S	35.4%	*[32.8, 38.0%]*	0.9%	*[0.5, 1.3%]*
Any locomotor limitation	S	33.6%	*[31.2, 36.0%]*	0.9%	*[0.1, 1.7%]*
Any self-care limitation	S	32.6%	*[29.7, 35.5%]*	0.7%	*[0.1, 1.3%]*
Problems walking about today	S	26.3%	*[25.2, 27.4%]*	0.4%	*[-0.6, 1.3%]*
High psychological distress	S	26.4%	*[24.9, 27.9%]*	0.9%	*[0.4, 1.4%]*
Recent angina	L	23.8%	*[20.1, 27.5%]*	-0.5%	*[-0.8, -0.1%]*
Recent heart attack/stroke	L	23.2%	*[19.7, 26.7%]*	-0.4%	*[-0.7, 0.0%]*
Breathlessness-Grade 3	S	22.9%	*[20.9, 24.9%]*	-1.6%	*[-2.5, -0.8%]*
Mental health LSI	L	20.4%	*[19.1, 21.7%]*	2.4%	*[1.8, 3.0%]*
IHD/stroke LSI	L	19.7%	*[17.9, 21.5%]*	-0.6%	*[-0.9, -0.2%]*
Wheezing stopping sleep	S	19.1%	*[17.1, 21.1%]*	-0.5%	*[-1.0, 0.1%]*
Mini stroke (TIA) symptoms	S	16.8%	*[15.0, 18.6%]*	-1.4%	*[-2.4, -0.4%]*
Angina symptoms	S	16.6%	*[14.1, 19.1%]*	-1.2%	*[-1.6, -0.7%]*
Psychological distress symptoms	S	15.2%	*[14.6, 15.8%]*	-1.3%	*[-2.4, -0.3%]*
Arthritis LSI	L	15.2%	*[14.3, 16.1%]*	-0.7%	*[-1.4, 0.0%]*
Any recent CVD	L	14.4%	*[12.7, 16.1%]*	0.5%	*[-0.1, 1.2%]*
Heart attack symptoms	S	14.1%	*[12.6, 15.6%]*	-0.5%	*[-1.3, 0.3%]*
Anxiety/depression-moderately	S	13.6%	*[13.0, 14.2%]*	0.1%	*[-1.1, 1.3%]*
Pain-any	S	12.9%	*[12.4, 13.4%]*	-3.3%	*[-4.6, -2.0%]*
COPD symptoms	S	12.6%	*[11.0, 14.2%]*	-1.6%	*[-2.3, -0.8%]*
Diabetes LSI	L	12.4%	*[11.1, 13.7%]*	2.1%	*[1.5, 2.6%]*
Recent diabetes	L	11.8%	*[10.2, 13.4%]*	2.2%	*[1.9, 2.6%]*
Breathlessness-Grade 2+	S	11.5%	*[10.5, 12.5%]*	-4.8%	*[-6.1, -3.5%]*
Any CVD LSI	L	11.0%	*[10.3, 11.7%]*	0.6%	*[-0.1, 1.4%]*
Other musculoskeletal LSI	L	9.8%	*[9.2, 10.4%]*	-0.8%	*[-1.7, 0.1%]*
Glycated haemoglobin	B	9.9%	*[7.9, 11.9%]*	2.1%	*[1.4, 2.7%]*
Asthma LSI	L	8.6%	*[7.8, 9.4%]*	0.7%	*[0.0, 1.4%]*
Recent wheezing/asthma	S	8.4%	*[7.7, 9.1%]*	-1.2%	*[-2.5, 0.1%]*
Recent high blood pressure	L	6.8%	*[5.7, 7.9%]*	4.8%	*[3.9, 5.6%]*
BMI-Underweight	B	6.2%	*[4.3, 8.1%]*	-0.1%	*[-0.3, 0.1%]*
Diagnosed asthma	L	5.9%	*[5.1, 6.7%]*	5.7%	*[4.5, 6.8%]*
High waist-hip ratio	B	4.6%	*[4.1, 5.1%]*	14.1%	*[13.0, 15.2%]*
Raised fibrinogen	B	4.3%	*[1.9, 6.7%]*	1.5%	*[0.3, 2.6%]*
Low HDL cholesterol	B	4.3%	*[2.8, 5.8%]*	-8.0%	*[-9.0, -7.1%]*
Raised C-reactive protein	B	3.7%	*[2.7, 4.7%]*	1.9%	*[-0.7, 4.5%]*
BMI-Obese	B	2.8%	*[2.5, 3.1%]*	8.9%	*[8.0, 9.7%]*
Anaemia	B	2.4%	*[0.8, 4.0%]*	-1.4%	*[-2.7, -0.1%]*
Biomarker high blood pressure	B	0.4%	*[-0.3, 1.1%]*	-5.0%	*[-5.6, -4.5%]*
High total cholesterol	B	0.0%	*[-0.6, 0.6%]*	-17.6%	*[-19.1, -16.1%]*
Iron deficiency	B	-0.5%	*[-1.3, 0.3%]*	-12.5%	*[-14.8, -10.2%]*

For peer review only - http://bmjopen.bmj.com/site/about/guidelines.xhtml

Having estimated this, we can see if the areas in which morbidity has been improving or declining are those that are particularly important for general health. This is shown visually in Figure 1 below (the measures are not labelled to enable the overall pattern to be seen, but the top-to-bottom order of measures is the same in the figure as in the preceding table; i.e. the measure at the top of the figure is 'Pain-extreme').

Figure 1: Change over time in morbidity measures & their association with bad general health[a]

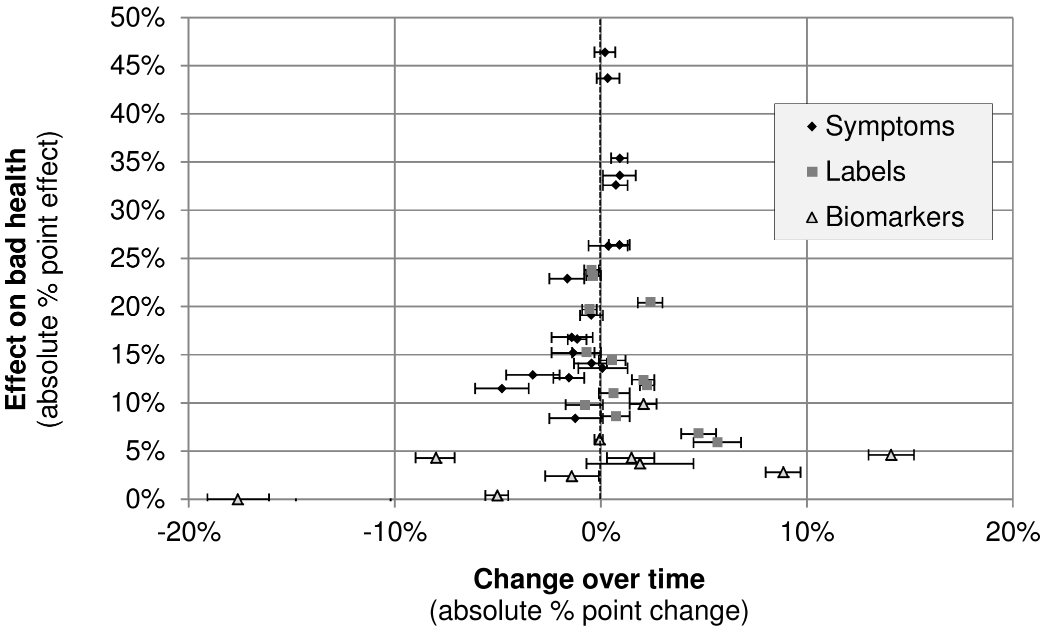

[a] 'Trend' is as reported above in the main paper. 'Effect on bad health' shows the effect of the morbidity measure on (very) bad health after controlling for age, sex (and their interaction) and educational level, using all years for which the individual morbidity measure is available. (This shows average marginal effects following a logistic regression; see text above).

It is easiest to interpret the figure by focussing on each group of measures in turn. Firstly, the biomarkers tend to have the weakest relationship with general health. Those with high levels of the diabetes biomarker (glycated haemoglobin) are 9.7% more likely to say they have bad health, and those who are underweight, with a high waist-hip ratio, raised fibrinogen, or low HDL cholesterol are 4-6% more likely to report bad health, but the other measures only had weaker relationships. Indeed, there was effectively no relationship between bad reported health and any of measured high blood pressure, high total cholesterol or iron deficiency.

Secondly, most of the measures based on medical labels have a moderately strong relationship with bad health (the weakest being lifetime asthma and recent high blood pressure, both of which can be asymptomatic), and these measures have mostly risen over time. There are however notable exceptions to this, including IHD/stroke LSI, recent angina and recent heart attack/stroke (the label-based measures with some of the strongest relationships with bad reported health), as well as arthritis and other musculoskeletal LSIs.

Finally, symptom-based measures unsurprisingly tend to have stronger relationships with bad reported health, although this ranges from the moderate (those reporting 'recent wheezing/asthma attack' were 8.5% more likely to report bad health) to the very strong (those reporting 'extreme

For peer review only - http://bmjopen.bmj.com/site/about/guidelines.xhtml

pain today' were 46.4% more likely to report bad health). In general, those symptoms-based measures with the strongest relationship with bad reported health were more likely to have increased over time ('extreme anxiety/depression today', 'locomotor limitations', and 'self-care limitations'). However, the size of the aforementioned declines in symptom-based measures of respiratory and cardiovascular morbidity was often greater.

For peer review only

For peer review only - http://bmjopen.bmj.com/site/about/guidelines.xhtml

Bibliography for Web Appendices

1. Case A, Deaton A. Rising morbidity and mortality in midlife among white non-Hispanic Americans in the 21st century. *Proceedings of the National Academy of Sciences* 2015;112(49):15078-83. doi: 10.1073/pnas.1518393112
2. Hiam L, Dorling D, Harrison D, et al. Why has mortality in England and Wales been increasing? An iterative demographic analysis. *Journal of the Royal Society of Medicine* 2017;110(4):153-62. doi: 10.1177/0141076817693599
3. Department of Health. Our Health and Wellbeing Today. London: HM Government, 2010.
4. Jagger C. Trends in life expectancy and healthy life expectancy. Future of an ageing population: evidence review. London: Foresight, Government Office for Science, 2015.
5. Office for Budget Responsibility. Welfare trends report: October 2016. Cm 9341. London: Her Majesty's Stationery Office, 2016.
6. Department for Work and Pensions, Department of Health. Improving Lives: The Work, Health and Disability Green Paper. Cm 9342. London: Her Majesty's Stationery Office, 2016.
7. Geiger BB. Morbidity in England 1994-2014 2019 [Available from:http://osf.io/dy6sv].
8. Szende A, Janssen B, Cabases J, editors. *Self-Reported Population Health: An International Perspective based on EQ-5D*. Netherlands: Springer, 2014.
9. Erens B, Primatesta P, Prior G. Health survey for England 1999: the health of minority ethnic groups. London: The Stationery Office, 2001.
10. National Heart Lung and Blood Institute. Sixth Report of the Joint National Committee on the Prevention, Detection, Evaluation and Treatment of High Blood Pressure. NIH Publication No 98-04080: National Institutes on Health, 1997.
11. Oyebode O. Cardiovascular disease. In: Craig R, Mindell J, eds. Health Survey for England 2011: Volume 1 - Health, social care and lifestyles. Leeds, UK: Health and Social Care Information Centre 2012.
12. Banks J, Marmot M, Oldfield Z, et al. The SES Health Gradient on Both Sides of the Atlantic. NBER Working Paper No 12674, 2006.
13. Lawlor DA, Adamson J, Ebrahim S. Performance of the WHO Rose angina questionnaire in post-menopausal women: Are all of the questions necessary? *Journal of Epidemiology and Community Health* 2003;57(7):538-41. doi: 10.1136/jech.57.7.538
14. Cook D, Shaper A, Macfarlane P. Using the WHO (Rose) Angina Questionnaire in Cardiovascular Epidemiology. *International Journal of Epidemiology* 1989;18(3):607-13. doi: 10.1093/ije/18.3.607
15. Fletcher C, Elmes P, Fairbairn M, et al. The significance of respiratory symptoms and the diagnosis of chronic bronchitis in a working population. *BMJ* 1959;2:257-66.
16. WHO. Physical status: the use and interpretation of anthropometry. Report of a WHO Expert Committee. WHO Technical Report Series 854. Geneva: World Health Organization (WHO), 1995.
17. National Obesity Observatory. Obesity and Overweight Surveillance in England: what is measured and where are the gaps?, 2009.
18. NICE. Obesity: The Prevention, Identification, Assessment and Management of Overweight and Obesity in Adults and Children NICE Clinical Guidelines, No 43. London: Centre for Public Health Excellence and National Collaborating Centre for Primary Care at the National Institute for Health and Clinical Excellence (NICE), 2006.
19. Hotchkiss JW, Davies CA, Gray L, et al. Trends in cardiovascular disease biomarkers and their socioeconomic patterning among adults in the Scottish population 1995 to 2009: cross-sectional surveys. *BMJ Open* 2012;2(3) doi: 10.1136/bmjopen-2011-000771
20. Moody A. Diabetes and hyperglycaemia. In: Craig R, Mindell J, eds. Health Survey for England 2011: Volume 1 - Health, social care and lifestyles. Leeds, UK: Health and Social Care Information Centre 2012.
21. Aresu M, Gordon-Dseagu V, Shelton N. Diabetes and glycaemia. In: Craig R, Hirani V, eds. Health Survey for England 2009, Volume 1: Health and lifestyles. Leeds, UK: The NHS Information Centre for health and social care 2010:59-74.

For peer review only - http://bmjopen.bmj.com/site/about/guidelines.xhtml

22. Steptoe A. Psychosocial biomarker research: integrating social, emotional and economic factors into population studies of aging and health. *Social Cognitive and Affective Neuroscience* 2011;6(2):226-33. doi: 10.1093/scan/nsq032
23. Hughes A, McMunn A, Bartley M, et al. Elevated inflammatory biomarkers during unemployment: modification by age and country in the UK. *Journal of Epidemiology and Community Health* 2015;69(7):673-79. doi: 10.1136/jech-2014-204404
24. Benzeval M, Davillas A, Kumari M, et al. Understanding Society: The UK Household Longitudinal Study Biomarker User Guide and Glossary (version 1). Colchester, Essex: Institute for Social and Economic Research, 2014.
25. Chaudhury M. Blood analytes. In: Sproston K, Primatesta P, eds. Health Survey for England, 2003, Vol 2: Risk Factors for Cardiovascular Disease: TSO 2004:241-88.
26. Erens B, Primatesta P. Health survey for England 1998: cardiovascular disease. *London: The Stationery Office* 1999
27. Chaudhury M, Tull K. Nutrition and haematological status. In: Craig R, Mindell J, eds. Health Survey for England, 2005: The health of older people, Vol 1: General health and function: TSO 2006:67-96.
28. Goldberg D, PA W. User Guide to the General Health Questionnaire. Windsor, UK: NFER-Nelson, 1988.
29. Stochl J, Böhnke JR, Pickett KE, et al. An evaluation of computerized adaptive testing for general psychological distress: combining GHQ-12 and Affectometer-2 in an item bank for public mental health research. *BMC Medical Research Methodology* 2016;16(1):58. doi: 10.1186/s12874-016-0158-7
30. ONS. Harmonised Concepts and Questions for Social Data Sources, Primary Principles: Long-lasting Health Conditions and Illnesses; Impairments and Disability [version 1.1]. London: Office for National Statistics (ONS), 2015.
31. Hall J, Mindell J. Respiratory symptoms and disease in adults In: Craig R, Mindell J, eds. Health Survey for England, 2010, Volume 1: Respiratory health: TSO 2011.
32. Sperrin M, Marshall A, Higgins V, et al. Slowing down of adult body mass index trend increases in England: a latent class analysis of cross-sectional surveys (1992-2010). *International Journal of Obesity* 2014;38(6)
33. Parsons S, Ingram M, Clarke-Cornwell AM, et al. A Heavy Burden: The occurrence and impact of musculoskeletal conditions in the United Kingdom today. Manchester: Arthritis Research UK & University of Manchester, 2011.
34. OECD. Fit Mind, Fit Job: From evidence to practice in mental health and work. Paris: OECD, 2015.
35. Spiers N, Qassem T, Bebbington P, et al. Prevalence and treatment of common mental disorders in the English national population, 1993–2007. *The British Journal of Psychiatry* 2016;209(2):150-56. doi: 10.1192/bjp.bp.115.174979
36. Moncrieff J, Pomerleau J. Trends in sickness benefits in Great Britain and the contribution of mental disorders. *Journal of Public Health Medicine* 2000;22:59-67.
37. Stansfeld SA, Woodley-Jones D, Rasul F, et al. Work-related distress in the 1990s - a real increase in ill health? . *Journal of Public Mental Health* 2008;7(1):25-31.
38. McManus S, Bebbington P, Jenkins R, et al., editors. *Mental health and wellbeing in England: Adult Psychiatric Morbidity Survey 2014*. Leeds: NHS Digital, 2016.
39. Maheswaran H, Kupek E, Petrou S. Self-reported health and socio-economic inequalities in England, 1996–2009: Repeated national cross-sectional study. *Social Science & Medicine* 2015;136–137:135-46. doi: http://dx.doi.org/10.1016/j.socscimed.2015.05.026
40. Katikireddi SV, Niedzwiedz CL, Popham F. Trends in population mental health before and after the 2008 recession: a repeat cross-sectional analysis of the 1991–2010 Health Surveys of England. *BMJ Open* 2012;2(5) doi: 10.1136/bmjopen-2012-001790
41. Jones M, Wass V. Understanding changing disability-related employment gaps in Britain 1998–2011. *Work, Employment & Society* 2013;27(6):982-1003. doi: 10.1177/0950017013475372

For peer review only - http://bmjopen.bmj.com/site/about/guidelines.xhtml

42. Barr B, Kinderman P, Whitehead M. Trends in mental health inequalities in England during a period of recession, austerity and welfare reform 2004 to 2013. *Social Science & Medicine* 2015;147:324-31. doi: http://dx.doi.org/10.1016/j.socscimed.2015.11.009
43. Tull KI, Hirani V, Ali A, et al. Impact of different diagnostic thresholds and the anaemia–ferritin–transferrin receptor model on the prevalence of anaemia and impaired iron status in older people. *Age and Ageing* 2009;38(5):609-13. doi: 10.1093/ageing/afp102
44. Dhalwani NN, O'Donovan G, Zaccardi F, et al. Long terms trends of multimorbidity and association with physical activity in older English population. *International Journal of Behavioral Nutrition and Physical Activity* 2016;13(1):8. doi: 10.1186/s12966-016-0330-9
45. Koné Pefoyo AJ, Bronskill SE, Gruneir A, et al. The increasing burden and complexity of multimorbidity. *BMC Public Health* 2015;15(1):415. doi: 10.1186/s12889-015-1733-2
46. Tetzlaff J, Junius-Walker U, Muschik D, et al. Identifying time trends in multimorbidity—defining multimorbidity in times of changing diagnostic practices. *Journal of Public Health* 2016:1-8. doi: 10.1007/s10389-016-0771-2
47. Marfeo EE, Haley SM, Jette AM, et al. Conceptual Foundation for Measures of Physical Function and Behavioral Health Function for Social Security Work Disability Evaluation. *Archives of Physical Medicine and Rehabilitation* 2013;94(9):1645-52.e2. doi: 10.1016/j.apmr.2013.03.015
48. Salomon JA, Vos T, Hogan DR, et al. Common values in assessing health outcomes from disease and injury: disability weights measurement study for the Global Burden of Disease Study 2010. *The Lancet* 2012;380(9859):2129-43. doi: http://dx.doi.org/10.1016/S0140-6736(12)61680-8
49. Murray CJL, Richards MA, Newton JN, et al. UK health performance: findings of the Global Burden of Disease Study 2010. *The Lancet* 2013;381(9871):997-1020. doi: https://doi.org/10.1016/S0140-6736(13)60355-4
50. WHO. Global Health Estimates 2015: Disease burden by Cause, Age, Sex, by Country and by Region, 2000-2015. Geneva: World Health Organization (WHO), 2016.
51. WHO. World report on disability. Geneva: World Health Organization (WHO) 2011.
52. Soldo BJ, Mitchell OS, Tfaily R, et al. Cross-cohort differences in health on the verge of retirement. NBER Working Paper No 12762: National Bureau of Economic Research, 2006.
53. Stewart ST, Cutler DM, Rosen AB. Comparison of Trends in U.S. Health-Related Quality of Life over the 2000's Using the SF-6D, HALex, EQ-5D, and EQ-5D Visual Analog Scale versus a Broader Set of Symptoms and Impairments. *Medical care* 2014;52(12):1010-16. doi: 10.1097/MLR.0000000000000181
54. Diederichs CP, Wellmann J, Bartels DB, et al. How to weight chronic diseases in multimorbidity indices? Development of a new method on the basis of individual data from five population-based studies. *Journal of Clinical Epidemiology* 2012;65(6):679-85. doi: 10.1016/j.jclinepi.2011.11.006

For peer review only

For peer review only - http://bmjopen.bmj.com/site/about/guidelines.xhtml

Reporting checklist for cross sectional study.

Based on the STROBE cross sectional guidelines.

Instructions to authors

Complete this checklist by entering the page numbers from your manuscript where readers will find each of the items listed below.

Your article may not currently address all the items on the checklist. Please modify your text to include the missing information. If you are certain that an item does not apply, please write "n/a" and provide a short explanation.

Upload your completed checklist as an extra file when you submit to a journal.

In your methods section, say that you used the STROBE cross sectionalreporting guidelines, and cite them as:

von Elm E, Altman DG, Egger M, Pocock SJ, Gotzsche PC, Vandenbroucke JP. The Strengthening the Reporting of Observational Studies in Epidemiology (STROBE) Statement: guidelines for reporting observational studies.

Author note: pages within the online Appendices are denoted by the prefix 'A'. Pages refer to submitted version with tracked changes visible.

		Reporting Item	Page Number
**Title and abstract**			
Title	#1a	Indicate the study's design with a commonly used term in the title or the abstract	1, 3
Abstract	#1b	Provide in the abstract an informative and balanced summary of what was done and what was found	3-4
**Introduction**			
Background / rationale	#2	Explain the scientific background and rationale for the investigation being reported	5-6
Objectives	#3	State specific objectives, including any prespecified hypotheses	5-10
**Methods**			
Study design	#4	Present key elements of study design early in the paper	6-14
Setting	#5	Describe the setting, locations, and relevant dates, including periods of recruitment, exposure, follow-up, and data collection	6-7, A6-9

For peer review only

Eligibility criteria	#6a	Give the eligibility criteria, and the sources and methods of selection of participants.	6-7, A6-9
	#7	Clearly define all outcomes, exposures, predictors, potential confounders, and effect modifiers. Give diagnostic criteria, if applicable	8-12, A12-23
Data sources / measurement	#8	For each variable of interest give sources of data and details of methods of assessment (measurement). Describe comparability of assessment methods if there is more than one group. Give information separately for for exposed and unexposed groups if applicable.	8-12, A12-23
Bias	#9	Describe any efforts to address potential sources of bias	6-14, 22, A6-9, A12-23
Study size	#10	Explain how the study size was arrived at	6, A9
Quantitative variables	#11	Explain how quantitative variables were handled in the analyses. If applicable, describe which groupings were chosen, and why	A12-23
Statistical methods	#12a	Describe all statistical methods, including those used to control for confounding	13-14
Statistical methods	#12b	Describe any methods used to examine subgroups and interactions	n/a
Statistical methods	#12c	Explain how missing data were addressed	A3-9
Statistical methods	#12d	If applicable, describe analytical methods taking account of sampling strategy	13-14
Statistical methods	#12e	Describe any sensitivity analyses	A10-11, A24-34
**Results**			
Participants	#13a	Report numbers of individuals at each stage of study—eg numbers potentially eligible, examined for eligibility, confirmed eligible, included in the study, completing follow-up, and analysed. Give information separately for for exposed and unexposed groups if applicable.	A3-A9
Participants	#13b	Give reasons for non-participation at each stage	A3-A9
Participants	#13c	Consider use of a flow diagram	n/a
Descriptive data	#14a	Give characteristics of study participants (eg demographic, clinical, social) and information on exposures and potential confounders. Give information separately for exposed and unexposed groups if applicable.	n/a [this is a descriptive study]

For peer review only

Descriptive data	#14b	Indicate number of participants with missing data for each variable of interest	A3-A9
Outcome data	#15	Report numbers of outcome events or summary measures. Give information separately for exposed and unexposed groups if applicable.	n/a [these form the main results]
Main results	#16a	Give unadjusted estimates and, if applicable, confounder-adjusted estimates and their precision (eg, 95% confidence interval). Make clear which confounders were adjusted for and why they were included	13-14, 15-21
Main results	#16b	Report category boundaries when continuous variables were categorized	A12-23
Main results	#16c	If relevant, consider translating estimates of relative risk into absolute risk for a meaningful time period	n/a [all estimates are given as absolute percentages]
Other analyses	#17	Report other analyses done—e.g., analyses of subgroups and interactions, and sensitivity analyses	A10-11, A24-34
**Discussion**			
Key results	#18	Summarise key results with reference to study objectives	21-23
Limitations	#19	Discuss limitations of the study, taking into account sources of potential bias or imprecision. Discuss both direction and magnitude of any potential bias.	21-22
Interpretation	#20	Give a cautious overall interpretation considering objectives, limitations, multiplicity of analyses, results from similar studies, and other relevant evidence.	21-23
Generalisability	#21	Discuss the generalisability (external validity) of the study results	21-23, A6-A9
**Other Information**			
Funding	#22	Give the source of funding and the role of the funders for the present study and, if applicable, for the original study on which the present article is based	1, A43

None The STROBE checklist is distributed under the terms of the Creative Commons Attribution License CC-BY. This checklist can be completed online using https://www.goodreports.org/, a tool made by the EQUATOR Network in collaboration with Penelope.ai

For peer review only

BMJ Open

Has working-age morbidity been declining? Changes over time in general health, chronic diseases, symptoms and biomarkers in England 1994-2014

Journal:	*BMJ Open*
Manuscript ID	bmjopen-2019-032378.R2
Article Type:	Original research
Date Submitted by the Author:	28-Nov-2019
Complete List of Authors:	Geiger, Ben; University of Kent, School of Social Policy, Sociology and Social Research (SSPSSR);
**Primary Subject Heading**:	Public health
Secondary Subject Heading:	Research methods
Keywords:	Morbidity, Population Health, Biomarkers, Cross-sectional studies, Trends, England

SCHOLARONE™
Manuscripts

I, the Submitting Author has the right to grant and does grant on behalf of all authors of the Work (as defined in the below author licence), an exclusive licence and/or a non-exclusive licence for contributions from authors who are: i) UK Crown employees; ii) where BMJ has agreed a CC-BY licence shall apply, and/or iii) in accordance with the terms applicable for US Federal Government officers or employees acting as part of their official duties; on a worldwide, perpetual, irrevocable, royalty-free basis to BMJ Publishing Group Ltd ("BMJ") its licensees and where the relevant Journal is co-owned by BMJ to the co-owners of the Journal, to publish the Work in this journal and any other BMJ products and to exploit all rights, as set out in our licence.

The Submitting Author accepts and understands that any supply made under these terms is made by BMJ to the Submitting Author unless you are acting as an employee on behalf of your employer or a postgraduate student of an affiliated institution which is paying any applicable article publishing charge ("APC") for Open Access articles. Where the Submitting Author wishes to make the Work available on an Open Access basis (and intends to pay the relevant APC), the terms of reuse of such Open Access shall be governed by a Creative Commons licence – details of these licences and which Creative Commons licence will apply to this Work are set out in our licence referred to above.

Other than as permitted in any relevant BMJ Author's Self Archiving Policies, I confirm this Work has not been accepted for publication elsewhere, is not being considered for publication elsewhere and does not duplicate material already published. I confirm all authors consent to publication of this Work and authorise the granting of this licence.

For peer review only

Has working-age morbidity been declining? Changes over time in general health, chronic diseases, symptoms and biomarkers in England 1994-2014

Authors:

Dr Ben B. Geiger, University of Kent, UK

Postal address: Ben B Geiger, School of Social Policy, Sociology and Social Research (SSPSSR), University of Kent, Canterbury, Kent, CT2 7NZ, UK

Email: b.b.geiger@kent.ac.uk

For peer review only

Word count *(exc. title page, abstract, tables, acknowledgements, contributions & references)*

3,950 words + 5 tables

MeSH Keywords

Morbidity; Population Health; Biomarkers; Cross-Sectional Studies; Trends; England

Acknowledgements

This work was supported by the Economic and Social Research Council (grant number ES/K009583/1).

Author statement

BBG was responsible for the design, data preparation, analysis and reporting of the study.

For peer review only - http://bmjopen.bmj.com/site/about/guidelines.xhtml

Acknowledgements

Many thanks to Clare Bambra for comments, and to Mariska van der Horst for research assistance; neither should be held responsible for the analysis or interpretation of the paper itself.

Funding

This work was supported by the Economic and Social Research Council (ESRC), grant number ES/K009583/1.

Competing interests

The author has worked on secondment at the UK Department for Work and Pensions (DWP) in 2015-16.

Data sharing

The Health Survey for England 1994-2014 are available for free to registered users at the UK Data Service - see https://beta.ukdataservice.ac.uk/datacatalogue/series/series?id=2000021#!/abstract. There are no conditions for re-use for non-commercial applications of the data.

The statistical code enabling replication using publicly available data is available from OSF (Morbidity in England 1994-2014 2019, available from: http://osf.io/dy6sv) and www.benbgeiger.co.uk.

For peer review only

Has working-age morbidity been declining? Changes over time in general health, chronic diseases, symptoms and biomarkers in England 1994-2014

Abstract:

Objectives: As life expectancy has increased in high-income countries, there has been a global debate about whether additional years of life are free from ill-health/disability. However, little attention has been given to changes over time in morbidity in the *working-age* population, particularly outside the US, despite its importance for health monitoring and social policy. This study therefore asks: what are the changes over time in working-age morbidity in England over two decades?

Design, setting and participants: We use a high-quality annual cross-sectional survey, the Health Survey for England ('HSE') 1994-2014. HSE uses a random sample of the English household population, with a combined sample size of over 140,000 people. We produce a newly-harmonised version of HSE that maximises comparability over time, including new non-response weights. While HSE is used for monitoring population health, it has hitherto not used for investigating morbidity as a whole.

Outcome measures: We analyse all 39 measures that are fully comparable over time – including chronic disease diagnoses, symptomatology and a number of biomarkers – adjusting for gender and age.

Results: We find a mixed picture: we see improving cardiovascular and respiratory health, but deteriorations in obesity, diabetes, some biomarkers, and feelings of extreme anxiety/depression, alongside stability in moderate mental ill-health and

For peer review only - http://bmjopen.bmj.com/site/about/guidelines.xhtml

musculoskeletal-related health. In several domains we also see stable or rising chronic disease *diagnoses* even where *symptomatology* has declined. While data limitations make it challenging to combine these measures into a single morbidity index, there is little systematic trend for declining morbidity to be seen in the measures that predict self-reported health most strongly.

Conclusions: Despite considerable falls in working-age mortality – and the assumptions of many policymakers that morbidity will follow mortality – there is no systematic improvement in overall working-age morbidity in England from 1994 to 2014.

Strengths and limitations of this study

- We provide a robust analysis of changes over time in morbidity in England for 39 measures across two decades using the Health Survey for England ('HSE').
- We include every morbidity measure for which consistent comparisons over time can be constructed in the HSE.
- We take care to maximise comparability over time, including constructing new non-response weights.
- However, response rates for each stage of the HSE have declined over time, and it is impossible to rule out changing non-response biases.
- There are also several dimensions of morbidity for which there is little trend data in HSE.

For peer review only - http://bmjopen.bmj.com/site/about/guidelines.xhtml

INTRODUCTION

As life expectancy has increased in high-income countries, there has been a global debate about whether additional years of life are free from ill-health/disability. It is now largely accepted that old-age disability has declined in the US (albeit varying by age/method),[1 2] although chronic illness increased,[3] and the picture beyond the US is more mixed.[4-6] Yet this research agenda has not been matched by similar attention to changes over time in morbidity in the *working-age* population. In the absence of direct evidence, policymakers have often made claims based on self-reports of general health,[6-8] which we know are unreliable.[9 10] The lack of evidence is even more problematic within social security, where many policymakers have assumed that working-age morbidity *must* have improved in recent decades given improvements in mortality (despite the potential for declining mortality to coexist with rising morbidity)[6] – and that therefore high/rising levels of claims are not 'genuine'.[11 12]

Almost the only direct evidence on changes over time in working-age morbidity in high-income countries comes from the US. Contrary to policymaker expectations, these studies have generally found *deteriorating* morbidity since the mid-1990s, particularly activities of daily living (ADLs) and physical functioning.[13-16] Other studies have focused on the older working-age population with similar results.[2 17] Again, not all measures show deteriorations, and not all studies come to identical conclusions,[18] but there is little sign of any improvement in morbidity among working-age Americans – despite a 23% fall in working-age mortality 1993-2013 (Web Appendix 1). Outside of the US, there is a paucity of evidence, but from the limited evidence that exists, there is again little sign of improving morbidity.[19-22]

For peer review only - http://bmjopen.bmj.com/site/about/guidelines.xhtml

This study therefore asks: is there empirical support for the hypothesis that working-age morbidity in England has declined? (H_1). Or does the evidence support alternative hypotheses of stable (H2) or even declining (H3) morbidity? We answer this using the Health Survey for England (HSE), a high quality Government survey with a combined sample of 140,000 individuals. We examine 39 specific aspects of morbidity rather than reducing morbidity to a single measure, partly because these produce more reliable trends, and partly to capture the multidimensional nature of morbidity.[23] However, we conclude by examining the broad picture of morbidity change, and how far this supports the competing hypotheses.

This analysis makes two contributions. Firstly, we provide one of the few systematic analyses of changes over time in working-age morbidity in any high-income country outside the US. Secondly, we supplement self-report measures with 10 'biomarkers', which are particularly valuable for showing genuine changes over time (rather than merely changes in how people describe their health), but which have rarely been examined alongside self-reported working-age morbidity trends (Martin et al. 2010[24] being an exception).

DATA AND METHODS

This section follows the STROBE cross sectional reporting guidelines.[25]

Data source

Robust evidence of change over time requires consistently-collected, high-quality data. We use the HSE, an annual government-sponsored cross-sectional survey of 3,000-11,000 adults with no proxy responses.[26-47] A particular advantage is that the

For peer review only - http://bmjopen.bmj.com/site/about/guidelines.xhtml

interview is followed by a nurse visit, which in selected years also includes a blood sample. Nevertheless, there are challenges in analysing change in HSE:

- Firstly, HSE was run by the Government Office of Population Censuses and Surveys in 1991-3, before changing to NatCen in 1994. We focus on 1994-2014 given evidence of a discontinuity at this point.
- Secondly, topic coverage of HSE varies year-to-year, accompanied by changes in question wording/filtering. Based on a systematic search of HSE questions, we have included every morbidity measure that is comparable over a significant duration. Even for measures that have been previously been analysed (e.g. BMI[48]), this new analysis uncovered further discontinuities (Web Appendices 2 & 3).
- Third, HSE excludes those in communal establishments. While a smaller problem for the working-age population than older ages,[2] we minimise the impact of rising university attendance by focussing on those aged 25+ (Web Appendix 3). The upper limit of the working-age population is set to 59 (women) and 64 (men) to match state pension ages at the start of the period.
- Fourth, HSE supplies non-response weights from 2003. However, there had been a substantial decline in response rates prior to the introduction of weights, particularly for blood samples (from 53.3% 1994 to 39.9% 2003; Web Appendix 3). We therefore reduce non-response biases by creating new non-response weights, described in Web Appendix 3.

The resulting sample sizes for the various stages of data collection are shown in Web Appendix 3. Our dataset substantially extends an existing HSE time-series

For peer review only

dataset (UK Data Archive SN7025); the code enabling other researchers to assemble this extended time-series dataset are freely available.[49]

Patient involvement

As this is a health monitoring study using secondary data, patients were not directly involved. However, from previous discussions we are aware that the study will be of interest to patient/disability advocacy groups, who will receive jargon-free summaries of the research.

Measures

We cannot interpret changes over time correctly without understanding different ways of operationalising 'morbidity'.[1] General health/disability measures – e.g. *"How is your health in general?"* – are a simple way of measuring morbidity unidimensionally, and clearly do capture something meaningful.[50] However, their generality means that despite consistent question wording, different people may interpret questions or response options differently (e.g. what 'good' health refers to).[51 p218-224] This can even occur *within* individuals, if they change their internal standards of measurement over time (contributing to 'response shift'[52]). Numerous causal factors contribute to variable comprehension/reporting, ranging from the experience of ill-health itself[52] to non-health factors such as social security incentives,[53] gendered- and age-related expectations, and medicalisation.[54]

These inconsistencies mean that general health/disability measures are inadequate for answering our question: trends in such measures can differ wildly between different surveys covering nominally the same concept and population, e.g. for disability in England[9] or self-rated health in the US.[10] Indeed, the HSE itself shows that England has experienced deteriorating 'bad general health' at the same time as

For peer review only - http://bmjopen.bmj.com/site/about/guidelines.xhtml

activity limitations have fallen (changes over time in seven general HSE health/disability measures are available in Web Appendix 4). Moreover, unidimensional measures are potentially misleading in that they gloss over the multidimensional nature of morbidity.[1]

To robustly answer our research question, we must instead focus on more *specific* morbidity measures that capture multiple aspects of morbidity. Our systematic search found 39 such measures that are comparable over time: these are summarised in Table 1, with further details in Web Appendix 5. (A further 29 measures are also included in Web Appendix 6; this includes 8 sub-components of measures in the main text, 16 reports of ever having a condition even if this not recent, and 5 other categories of LSI). These specific morbidity measures can be grouped into three types, which have different strengths and weaknesses with respect to our question:

1. *Medical labels:* some measures are based on medical labels, either diagnosed chronic diseases or self-reported types of longstanding illness. (Those reporting a longstanding illness were asked, *'what is the matter with you?'*; up to 6 responses were then coded by the interviewer based on ICD). These are imperfect measures of morbidity[55] as they partly reflect healthcare systems and medicalisation more broadly, both of which change over time. Nevertheless, they are an important element of morbidity as they have real consequences via increasing awareness/labelling of people's experiences.

2. *Symptom-based:* some measures are based on self-reports of ill-health symptoms or specific domains of activity limitations. These measures are

For peer review only - http://bmjopen.bmj.com/site/about/guidelines.xhtml

either single items (e.g. pain, anxiety/depression) or validated symptom scales (e.g. the Rose angina scale,[56 57] GHQ psychiatric distress[58]). The more specific and concrete nature of these measures *prima facie* makes more likely to be interpreted consistently over time than medical labels and general measures,. Others have reached a similar conclusion for comparisons across place,[55] particularly for disability measurement,[59 60] where the Washington Group on Disability Statistics – a UN agency founded in 2001 – have brokered a consensus that cross-country disability comparisons should be based on multiple measures of specific activity limitations.[61 62] We should nevertheless note that there is no guarantee that a given symptom/impairment-based question will be interpreted identically over time.[63 64]

3. *Biomarkers* – that is, objective measures of biological or physiological measures – have considerable strengths in analysing change, as they largely avoiding reporting biases that are likely to vary between socioeconomic groups and over time.[65] They do this at the price of an indirect and sometimes still-debated relationship to morbidity (see Web Appendix 5), and do not cover several important morbidity domains (e.g. we lack good biomarkers for mental distress, pain and fatigue).

These three types of measures are therefore complementary in understanding changing morbidity: biomarkers are least likely to be affected by changing respondent interpretations over time, but do not capture morbidity well; symptom-based measures capture morbidity well and are reasonably (if still imperfectly) reliable; and label-based measures are flawed in capturing symptoms/limitations but

For peer review only - http://bmjopen.bmj.com/site/about/guidelines.xhtml

do enable us to capture whether people consider themselves to have a medical condition.

For peer review only

For peer review only - http://bmjopen.bmj.com/site/about/guidelines.xhtml

Table 1: HSE morbidity measures

Category	Measure	Type[a]	Operationalisation (years available)
Cardio-vascular disease (CVD)	High blood pressure LSI[b]	L	Hypertension reported as longstanding illness (LSI) (1994-2011)
	Recent high blood pressure	L	Still has (or on medication for) doctor-diagnosed hypertension (1994-2013)
	Biomarker high blood pressure	B	Systolic BP >=140mmHg & diastolic BP >=90mmHg (1994-2013)
	High total cholesterol	B	Total cholesterol >= 5mmol/L (1994-2012)
	Low HDL cholesterol	B	High density lipoprotein (HDL) cholesterol <=1 mmol/L (1998-2013)
	Recent heart attack /stroke	L	Doctor-diagnosed heart attack or stroke in past 12mths (1994-2011)
	Recent angina	L	Doctor-diagnosed angina in past 12mths (1994-2011)
	Ischaemic heart/stroke LSI[b]	L	Stroke, heart attack or angina reported as longstanding illness (LSI) (1994-2011)
	Heart attack symptoms	S	Ever had severe pain across chest for ½hr (1994-2011)
	Mini stroke (TIA) symptoms	S	Attack of weakness/slurred speech/blurred vision in past 12mths (2003-11)
	Angina symptoms	S	Rose Angina scale definition of angina symptoms (1994-2011)
	Any recent CVD	L	Doctor-diagnosed heart condition (exc. hypertension) in past 12mths (1994-2011)
	Any CVD LSI	L	Any CVD reported as longstanding illness (LSI) (1994-2011)
Respiratory	COPD symptoms	S	Regular cough & phlegm for at least 3mths each year (1995-2010)
	Lifetime diagnosed asthma	L	Ever had doctor-diagnosed asthma (1995-2010)
	Asthma LSI[b]	L	Asthma reported as longstanding illness (LSI) (1994-2011)
	Breathlessness-grade 2	S	Short of breath when hurrying up walking uphill (1995-2010)
	Breathlessness-grade 3	S	Short of breath when walking on level ground (1995-2010)
	Recent wheezing/asthma	S	Wheezing, whistling in chest or asthma attack in past 12mths (1995-2010)
	Wheezing stopping sleep	S	Woken 1+ times/wk by wheezing/whistling in chest in last 12mths (1994-2010)
Obesity & diabetes	BMI-underweight	B	Body Mass Index (BMI) <=18.5kg/m^2 (1994-2013)
	BMI-obese	B	Body Mass Index (BMI) >= 30kg/m^2 (1994-2013)
	High waist-hip ratio	B	Waist-hip ratio of >1 for men and >0.85 for women (1994-2013)
	Recent diabetes	L	Currently taking medication for doctor-diagnosed diabetes (1994-2013)
	Diabetes LSI[b]	L	Diabetes reported as longstanding illness (LSI) (1994-2011)
	High glycated haemoglobin	B	HbA_{1C} >=48mmol/mol (2003-2013)
Mental Health	Mental health LSI[b]	L	Mental health reported as longstanding illness (LSI) (1994-2011)
	Psychiatric distress (GHQ)	S	4+ negative symptoms from 12-item General Health Questionnaire (1994-2014)
	Anxiety/depression-moderately	S	At least moderately anxious/depressed today (1996-2014)
	Anxiety/depression-extremely	S	Extremely anxious/depressed today (1996-2014)
Activity limitations	Problems walking today	S	Has at least some problems walking about today (1996-2014)
	Locomotor limitation	S	Can't walk far / bend down / go up or down stairs without resting (1996-2001)

For peer review only - http://bmjopen.bmj.com/site/about/guidelines.xhtml

Category	Measure	Type[a]	Operationalisation (years available)
& musculo-skeletal	Problems washing/dressing today	S	Has at least some problems washing/dressing today (1996-2014)
	Self-care limitation	S	Difficulty with one of six everyday activities (e.g. feeding, dressing) (1995-2001)
	Pain-any	S	Has at least some pain or discomfort today (1996-2014)
	Pain-extreme	S	Has extreme pain or discomfort today (1996-2014)
	Arthritis LSI[b]	L	Arthritis or rheumatism reported as longstanding illness (LSI) (1994-2011)
	Other musculoskeletal LSI[b]	L	Other musculoskeletal condition reported as longstanding illness (LSI) (1994-2011)
Sensory & Communication	LSI Eye or Ear	L	Eye or ear condition reported as longstanding illness (LSI) (1994-2011)
	Hearing limitation	S	Cannot follow TV programme at volume others find acceptable (1995-2001)
	Seeing limitation	S	Cannot see well enough to recognise friend across the road (1995-2001)
	Communicating limitation	S	Have problem communicating with other people (1995-2001)
Other Biomarkers	Raised C-reactive protein	B	CRP >3mg/L (1998-2009)
	Raised fibrinogen	B	Fibrinogen >4mg/L (1998-2009)
	Anaemia	B	Haemoglobin <13g/dL for men and <12g/dL for women (1994-2009)
	Iron deficiency	B	Serum ferritin < 45ng/ml (1994-2009)

See Web Appendix 5 for full details on all measures .[a] Measure type key: L=medical label; S=symptom-based; B=biomarker. [b] Particular causes of longstanding illness (LSI) come from the open question, 'what is the matter with you?' Up to 6 responses are then coded by the interviewer into a coding frame based on ICD.

For peer review only - http://bmjopen.bmj.com/site/about/guidelines.xhtml

ANALYSIS

In the first instance we look at unadjusted changes over time in each morbidity indicator, showing the actual levels of morbidity found in the population. However, we primarily focus on changes after adjustment for sex and age (following others[66 67]), akin to standardising for the age-sex composition of the population. Given that our aim is to *describe* changes rather than to explain them, we do not further adjust for potential causal influences on morbidity that are likely to vary over the period, such as employment over economic cycles. This is a task for future research, but we should note that such analysis is possible using our publicly-available time-series dataset that includes *inter alia* employment status, education and region.

We chose to examine discrete changes from the start to the end of available data for each measure, rather than using linear or non-linear trend terms. Given our aims of informing policy debates, this has three advantages: a discrete change is simple to interpret; it is compatible with the different start/end years available for different measures; and it does not require any assumptions about the functional form of trends (linear trends are particularly unlikely given the role of non-linear economic cycles). Individual survey years are grouped into 3-4 year periods to increase sample size and precision, but single-year prevalence is given in Web Appendix 7. Given our binary outcome measures, we use logistic regression models with the following form:

$$y_i = \text{logit}\,[\,\boldsymbol{\beta}_1\textbf{period}_\textbf{i} + \boldsymbol{\beta}_2\textbf{age}_\textbf{i} + \beta_3 male_i + \boldsymbol{\beta}_4(\textbf{age}_\textbf{i} * \text{male}_\text{i})\,]$$

For peer review only - http://bmjopen.bmj.com/site/about/guidelines.xhtml

…where $\mathbf{period_i}$ refers to a vector of period dummy variables (covering all periods in which there were any observations: 1994-96, 1997-2000, 2001-03, 2004-07, 2008-10 and 2011-14), $\boldsymbol{\beta_1}$ is a vector of our primary outcome coefficients showing change between each period and the earliest available period, $\mathbf{age_i}$ refers to a vector of age dummy variables, $\mathrm{male_i}$ refers to a binary gender dummy variable, and $\boldsymbol{\beta_2}$, β_3 and $\boldsymbol{\beta_4}$ refer to the coefficients on age, gender and their interaction respectively. We present average marginal effects rather than odds ratios, partly because these are simple to understand – odds ratios have no easy real-world interpretation for policymakers – but primarily because odds ratios are not fully comparable across different models, and cannot therefore underpin our comparison of changes over time between indicators.[68]

To avoid a binary cut-off of statistical significance,[69] 95% confidence intervals are used to convey precision. All analyses use weights, exclude boost samples that use different sampling methods, and adjust for the multistage clustered sample design and the stratification of the sample across survey years using the SVYSET command in Stata (although standard errors will be slightly underestimated as it is not possible to consistently adjust for sample stratification within years). For reasons of space, we are unable to discuss previous HSE studies of aspects of morbidity in the main text; these are instead described in Web Appendix 8.

For peer review only - http://bmjopen.bmj.com/site/about/guidelines.xhtml

RESULTS

Conditions with sharply declining mortality

We start by focussing on cardiovascular disease (CVD) and respiratory illness, which have both seen large falls in mortality (by >50% and >25% respectively among 0-64 year-olds 1994-2013; Web Appendix 1). Changes over time in *morbidity*, however, are shown in Table 2.

Table 2: Changes over time in cardiovascular and respiratory morbidity

	Starting period		Change from start to end period			
	Period	Prevalence	End period	Raw change	Adj.[a] change	Adj. change 95% CI
**Blood pressure/cholesterol**						
High blood pressure LSI[b]	1994-96	2.7%	2011-14	1.3%	1.0%	[0.4, 1.6%]
Recent high blood pressure	1994-96	4.2%	2011-14	5.2%	4.8%	[3.9, 5.6%]
Biomarker high BP	1994-96	8.4%	2011-14	-4.7%	-5.0%	[-5.6, -4.5%]
High total cholesterol	1994-96	75.7%	2011-14	-16.4%	-17.6%	[-19.1, -16.1%]
Low HDL cholesterol	1997-2000	11.8%	2011-14	-8.0%	-8.0%	[-9.0, -7.1%]
**Other CVD**						
Recent heart attack/stroke	1994-96	1.2%	2011-14	-0.3%	-0.4%	[-0.7, 0.0%]
Recent angina	1994-96	1.1%	2011-14	-0.4%	-0.5%	[-0.8, -0.1%]
IHD/stroke LSI[b]	1994-96	1.4%	2011-14	-0.4%	-0.6%	[-0.9, -0.2%]
Heart attack symptoms	1994-96	5.5%	2011-14	-0.3%	-0.5%	[-1.3, 0.3%]
Mini stroke (TIA) symptoms	2001-03	8.1%	2011-14	-1.4%	-1.4%	[-2.4, -0.4%]
Angina symptoms	1994-96	2.3%	2011-14	-1.1%	-1.2%	[-1.6, -0.7%]
Any CVD LSI[b]	1994-96	5.8%	2011-14	1.1%	0.6%	[-0.1, 1.4%]
Any recent CVD	1994-96	3.1%	2011-14	0.7%	0.5%	[-0.1, 1.2%]
**Respiratory**						
Lifetime diagnosed asthma	1994-96	11.2%	2008-10	5.5%	5.7%	[4.5, 6.8%]
Asthma LSI[b]	1994-96	5.0%	2011-14	0.7%	0.7%	[0.0, 1.4%]
Breathlessness-Grade 2+	1994-96	19.7%	2008-10	-4.4%	-4.8%	[-6.1, -3.5%]
Breathlessness-Grade 3	1994-96	7.8%	2008-10	-1.4%	-1.6%	[-2.5, -0.8%]
Recent wheezing/asthma	1994-96	19.5%	2008-10	-1.2%	-1.2%	[-2.5, 0.1%]
Wheezing stopping sleep	1994-96	3.6%	2008-10	-0.4%	-0.5%	[-1.0, 0.1%]
COPD symptoms	1994-96	6.6%	2008-10	-1.5%	-1.6%	[-2.3, -0.8%]

[a] 'Adj.' = adjusted for changing age and sex distribution of the working-age population. [b] LSI=longstanding illness; see Table 1.

Looking first at high blood pressure, biomarker-measured high blood pressure has halved over two decades (similar improvements are found for the biomarkers for total and HDL cholesterol). Yet when we look at self-reports (either people reporting this

as an LSI, or in response to a direct question about having recent diagnosed high blood pressure), we see large *rises* over time. There has been an increasing diagnosis of high blood pressure and increasing prescriptions of blood pressure-lowering drugs; these may have helped reduce the underlying incidence of high blood pressure while simultaneously raising people's awareness of morbidity.

Table 2 further shows declines in several key types of CVD (heart attack, mini-stroke, angina), whether measured through people's reports of the disease itself or their reports of its symptoms. Nevertheless, the morbidity declines (8-50%) are often not on the scale of the declines in mortality (>50%); this is likely to be because mortality declines are partly driven by improved treatment,[70] which means each incident CVD case is likely to last longer.[71 72] More surprisingly, the measures of 'any reported CVD' show no improvement (with some, uncertain signs of *rises*). Looking at its sub-components (Web Appendix 6), this seems to be due to possible increases in diagnosed irregular heart rhythm and other heart trouble.

Finally, Table 2 shows that symptoms-based measures of respiratory morbidity have improved, particularly COPD symptoms (regular cough & phlegm) and breathlessness (at both levels), and more uncertainly for recent wheezing/asthma and wheezing stopping sleep. Again, though, diagnosis-related measures of asthma – reported diagnoses, or self-reports of having asthma as a longstanding illness – have risen, even while underlying symptomatology is improving.

Overall, Table 2 illustrates how changes over time in morbidity do not necessarily follow changes in mortality. There are definite improvements in CVD risk factors and respiratory symptomatology on the scale of improvements in mortality. But the

For peer review only - http://bmjopen.bmj.com/site/about/guidelines.xhtml

For peer review only

prevalence of self-reported CVD conditions such as heart attacks have only declined by a smaller amount, and recent doctor-diagnosed hypertension, any CVD, and asthma diagnoses have either stayed stable or risen.

Conditions with claims of increasing prevalence

The previous section focussed on conditions where there may be an *a priori* expectation that morbidity has improved (given declining mortality); in this section, we focus on three areas where there have been widespread claims of increasing prevalence – obesity, diabetes, and mental health.

Looking at Table 3, we do indeed confirm a large rise in obesity in HSE (an 8.0-9.7% rise from an obesity prevalence of 16.9% in 1994-96). The rise in high waist-hip ratios – sometimes suggested to be a better measure of potential morbidity [73] – is even larger. This has come alongside little change in the prevalence of being *underweight* over this period.

Table 3: Changes over time in obesity, diabetes and mental health

	Starting period		Change from start to end period			
	Period	Prevalence	End period	Raw change	Adj.[a] change	Adj. change 95% CI
**Underweight/Obesity**						
BMI-Underweight	1994-96	1.0%	2011-14	-0.1%	-0.1%	[-0.3, 0.1%]
BMI-Obese	1994-96	16.9%	2011-14	9.3%	8.9%	[8.0, 9.7%]
High waist-hip ratio	1994-96	9.5%	2011-14	14.8%	14.1%	[13.0, 15.2%]
**Diabetes**						
Recent diabetes	1994-96	1.2%	2011-14	2.4%	2.2%	[1.9, 2.6%]
Diabetes LSI[b]	1994-96	1.5%	2011-14	2.3%	2.1%	[1.5, 2.6%]
Glycated haemoglobin	2001-03	2.7%	2011-14	2.1%	2.1%	[1.4, 2.7%]
**Mental health**						
Mental health LSI[b]	1994-96	2.1%	2011-14	2.5%	2.4%	[1.8, 3.0%]
Psychological distress[c]	1994-96	17.1%	2011-14	-1.3%	-1.3%	[-2.4, -0.3%]
Anx./depression-moderate[d]	1994-96	21.9%	2011-14	0.3%	0.1%	[-1.1, 1.3%]
Anx./depression-extremely[d]	1994-96	1.8%	2011-14	1.0%	0.9%	[0.5, 1.3%]

For peer review only - http://bmjopen.bmj.com/site/about/guidelines.xhtml

[a] 'Adj.' = adjusted for changing age and sex distribution of the working-age population. [b] LSI=longstanding illness; see Table 1. [c] GHQ; see Web Appendix 5. [d] 'Anx./depression'= Feeling of anxiety/depression today – see Table 1.

Table 3 also confirms a large rise in diabetes. This can be seen whether diabetes is measured through people reporting diabetes as an LSI, a specific question about people currently taking medication for diabetes, or via a diabetes biomarker (glycated haemoglobin). It is worth noting that this clear rise in diabetes has occurred despite a *decline* in the age 0-64 death rate from diabetes, by more than one-third 1994-2013 (Web Appendix 1) – indeed, rising prevalence is *because of* falling mortality [74] – again demonstrating the difference between changes in mortality and morbidity.

Trends in mental health are more contentious in the wider literature (see Web Appendix 8), and the measures in HSE are not as strong as the more occasional Adult Psychiatric Morbidity Surveys.[75] Nevertheless, HSE offers a unique annual perspective on self-reported mental health. As we might expect from increasing treatment/diagnosis, we see a doubling in people reporting a mental health LSI. However, the symptoms-based measures show a more mixed picture:

- Neither of the measures that capture more moderate mental ill-health show rising ill-health (these are psychological distress symptoms and people reporting a feeling of anxiety/depression today, both with a relatively common prevalence of 15-25%). If we break this down by year (see Web Appendix 7), we can see moderate mental ill-health symptoms fell between the mid-1990s and the mid-2000s, before rising in 2009.
- In contrast, the single measure capturing a feeling of extreme anxiety/depression today does show rising morbidity. To see if there were

similar signs of rising mental ill-health at extremes in our other measure (psychological distress), we looked at a much higher GHQ threshold of 10 negative responses out of 12 questions (compared to the conventional threshold of 4). Unlike the conventional GHQ measure, this also showed an increase over time (95% CI of a 0.4 to 1.4% rise; see Web Appendix 6). While the GHQ is not designed to capture *severe* psychological distress in this way, others have similarly looked at moderate and extreme psychological distress using GHQ – and indeed, have found that rises in distress over time 1991-2008 are concentrated in the more extreme measure.[76]

Overall, while labelling of mental health conditions has undoubtedly risen, trends in mental health symptoms vary across measures. If we interpret higher GHQ thresholds as indicating more serious psychological distress, then we can see a consistent picture: moderate mental ill-health symptoms fell from the mid-1990s to the mid-2000s before rising around the time of the 2008 economic crisis (as we would expect[77]), whereas more extreme mental ill-health has more consistently risen.

Activity limitations, musculoskeletal and pain

Pain/musculoskeletal conditions are a major component of working-age morbidity, yet very few previous studies show changes over time in symptomatology, and even those that exist[78] sometimes have debatable comparability.[79] Table 4 shows a fall in some – but not all – HSE measures focussed on pain and musculoskeletal morbidity. Arthritis as a longstanding illness (LSI) has declined (the precision of the estimates is greater when looking at 2008-10 rather than 2011-14, and shows a decline of 0.3-1.2%). There are some (similarly uncertain) signs that other musculoskeletal LSIs

For peer review only - http://bmjopen.bmj.com/site/about/guidelines.xhtml

have also fallen, and noticeably fewer people say that they have any pain/discomfort today, although there has been no change in people saying they have extreme pain/discomfort. The echoes a previous study that found different trends in low back pain of different levels of severity.[80]

Similarly, there has been a rise in all four activity limitations measures in HSE – although the increases are sometimes uncertain, and are smaller after adjusting for changes in age/sex structure. Moreover, the timing of the rises differ between the measures: the trend in limitations lasting at least a year shows a rise 1994-6 to 2001-3, but the two measures of 'limitations today' do not, instead showing a possible slight rise in the more recent period (see Web Appendix 7; this difference remains if we focus on the sub-components of year-long limitations that more closely match to the 'limitations today' questions, see Web Appendix 6). Still, the measures can collectively be seen as offering some, albeit relatively weak, evidence for an increase in activity limitations.

Table 4: Changes over time in activity limitations, pain & musculoskeletal morbidity

	**Starting period**		**Change from start to end period**			
	Period	Prevalence	End period	Raw change	Adj.[a] change	Adj. change 95% CI
**Activity limitations**						
Problems walking about	1994-96	11.5%	2011-14	1.0%	0.4%	[-0.6, 1.3%]
Any locomotor limitation	1994-96	6.8%	2001-03	1.1%	0.9%	[0.1, 1.7%]
Probs. washing/dressing	1994-96	3.4%	2011-14	0.6%	0.3%	[-0.2, 0.9%]
Any self-care limitation	1994-96	3.9%	2001-03	0.8%	0.7%	[0.1, 1.3%]
**Musculoskeletal/pain**						
Pain-any	1994-96	32.0%	2011-14	-2.2%	-3.3%	[-4.6, -2.0%]
Pain-extreme	1994-96	3.0%	2011-14	0.4%	0.2%	[-0.3, 0.7%]
Arthritis LSI[b]	1994-96	5.3%	2011-14	-0.3%	-0.7%	[-1.4, 0.0%]
Other musculoskeletal LSI[b]	1994-96	9.7%	2011-14	-0.5%	-0.8%	[-1.7, 0.1%]

[a] 'Adj.' = adjusted for changing age and sex distribution of the working-age population. [b] LSI=longstanding illness; see Table 1.

For peer review only - http://bmjopen.bmj.com/site/about/guidelines.xhtml

Other measures

Changes over time in other measures are shown in Table 5 below. This includes four biomarkers that are more difficult to compare directly to self-reports:

- Changes over time are available for two biomarkers of inflammation (C-reactive protein ('CRP') and fibrinogen). These are associated with a number of conditions including heart disease, diabetes, cancer [81] and – in the case of CRP – even depression.[82] Table 5 shows that both biomarkers have rising morbidity from 1997-2000 to 2008-10 (although for CRP, the confidence interval is wide and there is a non-negligible possibility that the change is negative).
- The two other biomarkers available in HSE are clearly focussed on anaemia and iron deficiency. Table 5 shows that both of these have declined, with particularly clear evidence for a decline in iron deficiency.

Table 5: Changes over time in other morbidity measures

	Starting period		Change from start to end period			
	Period	Prevalence	End period	Raw Change	Adj.[a] change	Adj. change 95% CI
**Other biomarkers**						
Raised C-reactive protein	1997-2000	21.4%	2008-10	2.1%	1.9%	[-0.7, 4.5%]
Raised fibrinogen	1997-2000	2.3%	2008-10	1.6%	1.5%	[0.3, 2.6%]
Anaemia	1994-96	6.7%	2008-10	-1.4%	-1.4%	[-2.7, -0.1%]
Iron deficiency	1994-96	39.9%	2008-10	-12.9%	-12.5%	[-14.8, -10.2%]
**Sensory & communication**						
LSI Eye or Ear[b]	1994-96	2.8%	2011-14	-0.9%	-1.0%	[-1.5, -0.6%]
Hearing limitation	1994-96	4.3%	2001-03	-1.5%	-1.6%	[-2.1, -1.0%]
Seeing limitation	1994-96	1.4%	2001-03	-0.2%	-0.2%	[-0.6, 0.1%]
Communicating limitation	1994-96	1.0%	2001-03	0.1%	0.1%	[-0.2, 0.4%]

[a] 'Adj.' = adjusted for changing age and sex distribution of the working-age population. [b] LSI=longstanding illness; see Table 1.

For peer review only - http://bmjopen.bmj.com/site/about/guidelines.xhtml

Table 5 also shows changes over time in sensory and communication-related morbidity. This shows a fall in eye/ear conditions (1994-6 to 2011-14) as well as hearing limitations in the earlier period (1994-6 to 2001-03), but no change in people having difficulty communicating with others.

DISCUSSION

Despite considerable evidence on morbidity trends among older people, there are few published studies on changes in morbidity among the working-age population, particularly outside the USA. In this paper, we have analysed changes over time in working-age morbidity in England 1994-2014 using a high-quality repeated cross-sectional study. We see improvements in cardiovascular morbidity, respiratory morbidity and anaemia, but deteriorating obesity, diabetes, some biomarkers (fibrinogen and possibly also CRP) and feelings of extreme anxiety/depression. We see little systematic change over time in more common mental ill-health or musculoskeletal conditions, pain/mobility, and self-care limitations. Symptomatology and chronic disease diagnoses also often go in different directions – chronic disease diagnoses have sometimes stayed stable or even risen at the same time that underlying symptomatology has declined (such as for mental health conditions, asthma, hypertension, and CVD as a whole), mirroring findings at older ages.[3]

Our analysis has several strengths. We include every morbidity measure for which consistent changes can be constructed, including chronic disease, functioning and symptomatology, and biomarkers. We use a single survey series collected by a single survey organisation; exclude under-25s for whom comparability of survey coverage is unlikely; and construct new non-response weights. Nevertheless, we must note three limitations. Firstly, response rates for each stage of the HSE have

For peer review only - http://bmjopen.bmj.com/site/about/guidelines.xhtml

declined over time (see Web Appendix 3), and while we create new non-response weights covering the entire period, it is still possible that socioeconomically disadvantaged people (within any age-sex-region group) have become less likely to respond – and as they tend to be in worse health, this could mask deteriorating morbidity. Secondly, even if non-response biases have not changed, it is possible that people respond differently over time even to identical questions. Third, there are several dimensions of morbidity for which there is little comparable data in HSE. This includes several areas in which morbidity among the working-age population seems to be rising, including *inter alia* cognitive complaints,[83] allergic disorders,[84] and liver cirrhosis (see Web Appendix 1), as well as some areas in which morbidity seems likely to have fallen, such as chronic kidney disease.[85]

It is clear that there are different trends in different dimensions of morbidity – but for policymakers, this leaves the question of whether working-age morbidity as a whole is unchanged (H2), getting better (H1) or getting worse (H3), to the extent that it makes sense to place health on a unidimensional scale. While we cannot create a single morbidity index here, Web Appendix 9 shows the association of each measure with bad general self-rated health (net of age, gender and education). This shows little systematic trend for falling morbidity to be seen in the measures that predict health the most (indeed, the evidence weakly points in the other direction, towards rising morbidity). This provides greater support for H2 than H1 or H3, mirroring evidence from the Global Burden of Disease study (see Web Appendix 9).

In conclusion, despite considerable falls in working-age mortality and gains in life expectancy – and the ensuing expectations of social security policymakers for improving morbidity – there is no evidence of systematic improvement in overall

For peer review only - http://bmjopen.bmj.com/site/about/guidelines.xhtml

working-age morbidity in England from 1994 to 2014. However, two pieces of further research could strengthen this evidence base. Firstly, the ideal measures for analysing changes in morbidity are functional limitations measures, which are included in the HSE from 1996. However, these were last asked to the working-age population in 2001, and it is a priority to repeat these measures in future years of HSE. Secondly, there is a surprising paucity of studies looking at the changing morbidity of the working-age population outside the US. Given their importance in public debate – particularly in discussions of retirement ages and disability benefits – we hope that other authors will repeat and extend our analyses here, including disaggregating these changes across different regions and sociodemographic groups.

For peer review only

For peer review only - http://bmjopen.bmj.com/site/about/guidelines.xhtml

REFERENCES

1. Freedman VA, Crimmins E, Schoeni RF, et al. Resolving inconsistencies in trends in old-age disability: report from a technical working group. *Demography* 2004;41(3):417-41.
2. Freedman VA, Spillman BC, Andreski PM, et al. Trends in Late-Life Activity Limitations in the United States: An Update From Five National Surveys. *Demography* 2013;50(2):661-71. doi: 10.1007/s13524-012-0167-z
3. Freedman VA, Schoeni RF, Martin LG, et al. Chronic Conditions and the Decline in Late-Life Disability. *Demography* 2007;44(3):459-77.
4. Lafortune G, Balestat G. Trends in severe disability among elderly people. Paris: OECD, 2007.
5. Bowling A. Commentary: Trends in activity limitation. *International Journal of Epidemiology* 2011;40(4):1068-70. doi: 10.1093/ije/dyr069
6. Jagger C. Trends in life expectancy and healthy life expectancy. Future of an ageing population: evidence review. London: Foresight, Government Office for Science, 2015.
7. Office for Budget Responsibility. Welfare trends report: October 2016. Cm 9341. London: Her Majesty's Stationery Office, 2016.
8. Department for Work and Pensions, Department of Health. Improving Lives: The Work, Health and Disability Green Paper. Cm 9342. London: Her Majesty's Stationery Office, 2016.
9. Baumberg B, Jones M, Wass V. Disability prevalence and disability-related employment gaps in the UK 1998–2012: Different trends in different surveys? *Social Science & Medicine* 2015;141:72-81. doi: http://dx.doi.org/10.1016/j.socscimed.2015.07.012
10. Salomon JA, Nordhagen S, Oza S, et al. Aer Americans feeling less healthy? The puzzle of trends in self-rated health. *American Journal of Epidemiology* 2009;170(3):343-51.
11. OECD. Sickness, Disability and Work: Breaking the Barriers, Vol 2: Australia, Luxembourg, Spain and the United Kingdom Paris: OECD, 2008.
12. Wise DA, editor. *Social Security Programs and Retirement Around the World: The Capacity to Work at Older Ages*. Chicago: University of Chicago Press for the National Bureau of Economic Research (NBER), 2017.
13. Martin LG, Freedman VA, Schoeni RF, et al. Trends In Disability And Related Chronic Conditions Among People Ages Fifty To Sixty-Four. *Health Affairs* 2010;29(4):725-31. doi: 10.1377/hlthaff.2008.0746
14. Bhattacharya J, Choudhry K, Lakdawalla D. Chronic Disease and Severe Disability among Working-Age Populations. *Medical Care* 2008;46(1):92-100.
15. Martin LG, Schoeni RF. Trends in disability and related chronic conditions among the forty-and-over population: 1997-2010. *Disability and Health Journal* 2014;7(1):S4-S14. doi: 10.1016/j.dhjo.2013.06.007
16. Lakdawalla DN, Bhattacharya J, Goldman DP. Are the young becoming more disabled? *Health affairs* 2004;23(1):168-76.
17. Seeman TE, Merkin SS, Crimmins EM, et al. Disability Trends Among Older Americans: National Health and Nutrition Examination Surveys, 1988–1994 and 1999–2004. *American Journal of Public Health* 2010;100(1):100-07. doi: 10.2105/AJPH.2008.157388
18. Weir D. Are baby boomers living well longer. In: Madrian B, Mitchell OS, Soldo BJ, eds. Redefining retirement: How will boomers fare. Oxford: Oxford UP 2007:95-111.
19. van Oostrom SH, Gijsen R, Stirbu I, et al. Time Trends in Prevalence of Chronic Diseases and Multimorbidity Not Only due to Aging: Data from General Practices and Health Surveys. *PLoS ONE* 2016;11(8):e0160264. doi: 10.1371/journal.pone.0160264

For peer review only - http://bmjopen.bmj.com/site/about/guidelines.xhtml

20. Solé-Auró A, Alcañiz M. Are we living longer but less healthy? Trends in mortality and morbidity in Catalonia (Spain), 1994–2011. *European Journal of Ageing* 2015;12(1):61-70. doi: 10.1007/s10433-014-0317-9
21. Audureau E, Rican S, Coste J. Worsening trends and increasing disparities in health-related quality of life: evidence from two French population-based cross-sectional surveys, 1995–2003. *Quality of Life Research* 2013;22(1):13-26. doi: 10.1007/s11136-012-0117-7
22. Clause-Verdreau A-C, Audureau É, Leplège A, et al. Contrasted trends in health-related quality of life across gender, age categories and work status in France, 1995–2016: repeated population-based cross-sectional surveys using the SF-36. *Journal of Epidemiology and Community Health* 2019;73(1):65-72. doi: 10.1136/jech-2018-210941
23. Marfeo EE, Haley SM, Jette AM, et al. Conceptual Foundation for Measures of Physical Function and Behavioral Health Function for Social Security Work Disability Evaluation. *Archives of Physical Medicine and Rehabilitation* 2013;94(9):1645-52.e2. doi: 10.1016/j.apmr.2013.03.015
24. Martin LG, Schoeni RF, Andreski PM. Trends in health of older adults in the United States: past, present, future. *Demography* 2010;47(Suppl):S17-S40.
25. von Elm E, Altman D, Egger M, et al. The Strengthening the Reporting of Observational Studies in Epidemiology (STROBE) Statement: guidelines for reporting observational studies. *Annals of Internal Medicine* 2007;147:573-77.
26. Mindell J, Biddulph JP, Hirani V, et al. Cohort Profile: The Health Survey for England. *International Journal of Epidemiology* 2012;41(6):1585-93. doi: 10.1093/ije/dyr199
27. NatCen Social Research, University College London Department of Epidemiology and Public Health. Health Survey for England, 2014. [computer file], 1st Edition. UK Data Archive, SN: 7919, 2016. http://dx.doi.org/10.5255/UKDA-SN-7919-1
28. NatCen Social Research, University College London Department of Epidemiology and Public Health. Health Survey for England, 2013. [computer file], 1st Edition. UK Data Archive, SN: 7649, 2015. http://dx.doi.org/10.5255/UKDA-SN-7649-1
29. NatCen Social Research, University College London Department of Epidemiology and Public Health. Health Survey for England, 2012. [computer file], 1st Edition. UK Data Archive, SN: 7480, 2014. http://dx.doi.org/10.5255/UKDA-SN-7480-1
30. NatCen Social Research, University College London Department of Epidemiology and Public Health. Health Survey for England, 2011. [computer file], 1st Edition. UK Data Archive, SN: 7260, 2013. http://dx.doi.org/10.5255/UKDA-SN-7260-1
31. NatCen Social Research, Royal Free and University College Medical School Department of Epidemiology and Public Health. Health Survey for England, 2010. [computer file], 2nd Edition. UK Data Archive, SN: 6986, 2012. http://dx.doi.org/10.5255/UKDA-SN-6986-2
32. National Centre for Social Research, University College London Department of Epidemiology and Public Health. Health Survey for England, 2009. [computer file], 2nd Edition. UK Data Archive, SN: 6732, 2011. http://dx.doi.org/10.5255/UKDA-SN-6732-1
33. National Centre for Social Research, University College London Department of Epidemiology and Public Health. Health Survey for England, 2008. [computer file], 3rd Edition. UK Data Archive, SN: 6397, 2011. http://dx.doi.org/10.5255/UKDA-SN-6397-1
34. National Centre for Social Research, University College London Department of Epidemiology and Public Health. Health Survey for England, 2007. [computer file], 2nd Edition. UK Data Archive, SN: 6112, 2010. http://dx.doi.org/10.5255/UKDA-SN-6112-1
35. National Centre for Social Research, University College London Department of Epidemiology and Public Health. Health Survey for England, 2006. [computer file], 2nd Edition. UK Data Archive, SN: 4150, 2008.

For peer review only - http://bmjopen.bmj.com/site/about/guidelines.xhtml

36. National Centre for Social Research, University College London Department of Epidemiology and Public Health. Health Survey for England, 2005. [computer file], 3rd Edition. UK Data Archive, SN: 5675, 2011. http://dx.doi.org/10.5255/UKDA-SN-5675-1
37. National Centre for Social Research, University College London Department of Epidemiology and Public Health. Health Survey for England, 2004. [computer file], 2nd Edition. UK Data Archive, SN: 5439, 2010. http://dx.doi.org/10.5255/UKDA-SN-5439-1
38. National Centre for Social Research, University College London Department of Epidemiology and Public Health. Health Survey for England, 2003. [computer file], 2nd Edition. UK Data Archive, SN: 5098, 2010. http://dx.doi.org/10.5255/UKDA-SN-5098-1
39. National Centre for Social Research, University College London Department of Epidemiology and Public Health. Health Survey for England, 2002. [computer file], 2nd Edition. UK Data Archive, SN: 4912, 2010. http://dx.doi.org/10.5255/UKDA-SN-4912-1
40. National Centre for Social Research, University College London Department of Epidemiology and Public Health. Health Survey for England, 2001. [computer file], 3rd Edition. UK Data Archive, SN: 4628, 2010. http://dx.doi.org/10.5255/UKDA-SN-4628-1
41. National Centre for Social Research, University College London Department of Epidemiology and Public Health. Health Survey for England, 2000. [computer file], 4th Edition. UK Data Archive, SN: 4487, 2011. http://dx.doi.org/10.5255/UKDA-SN-4487-1
42. National Centre for Social Research, University College London Department of Epidemiology and Public Health. Health Survey for England, 1999. [computer file], 3rd Edition. UK Data Archive, SN: 4365, 2002.
43. National Centre for Social Research, University College London Department of Epidemiology and Public Health. Health Survey for England, 1998. [computer file], 5th Edition. UK Data Archive, SN: 4150, 2010. http://dx.doi.org/10.5255/UKDA-SN-4150-1
44. Joint Health Surveys Unit of Social and Community Planning Research, University College London Department of Epidemiology and Public Health. Health Survey for England, 1997. [data collection], 4th Edition. UK Data Service, SN: 3979, 2017. http://doi.org/10.5255/UKDA-SN-3979-2
45. Joint Health Surveys Unit of Social and Community Planning Research, University College London Department of Epidemiology and Public Health. Health Survey for England, 1996. [data collection], 5th Edition. UK Data Service, SN: 3886, 2017. http://doi.org/10.5255/UKDA-SN-3886-2
46. Joint Health Surveys Unit of Social and Community Planning Research, University College London Department of Epidemiology and Public Health. Health Survey for England, 1995. [data collection], 5th Edition. UK Data Service, SN: 3796, 2017. http://doi.org/10.5255/UKDA-SN-3796-2
47. Joint Health Surveys Unit of Social and Community Planning Research, University College London Department of Epidemiology and Public Health. Health Survey for England, 1994. [data collection], 5th Edition. UK Data Service, SN: 3640, 2017. http://doi.org/10.5255/UKDA-SN-3640-2
48. Sperrin M, Marshall A, Higgins V, et al. Slowing down of adult body mass index trend increases in England: a latent class analysis of cross-sectional surveys (1992-2010). *International Journal of Obesity* 2014;38(6)
49. Geiger BB. Morbidity in England 1994-2014 2019 [Available from:http://osf.io/dy6sv].
50. Quesnel Vallee A. Self-rated health: caught in the crossfire of the quest for 'true' health? *International Journal of Epidemiology* 2007;36:1161-64.
51. Groves RM, Fowler FJ, Couper MP, et al. Survey Methodology [2e]. Hoboken, NJ: Wiley 2009.

For peer review only - http://bmjopen.bmj.com/site/about/guidelines.xhtml

52. Sprangers MA, Schwartz CE. Integrating response shift into health-related quality of life research: a theoretical model *Social Science & Medicine* 1999;48(11):1507-151.
53. O'Brien R. Disability and the Worlds of Welfare Capitalism. *Sociological Science* 2015;2(1):1-19. doi: 10.15195/v2.a1
54. OECD. Sick on the Job? Myths and Realities about Mental Health and Work. Paris: OECD, 2012.
55. Cieza A, Oberhauser C, Bickenbach J, et al. The English are healthier than the Americans: really? *International Journal of Epidemiology* 2015:229-38. doi: 10.1093/ije/dyu182
56. Lawlor DA, Adamson J, Ebrahim S. Performance of the WHO Rose angina questionnaire in post-menopausal women: Are all of the questions necessary? *Journal of Epidemiology and Community Health* 2003;57(7):538-41. doi: 10.1136/jech.57.7.538
57. Cook D, Shaper A, Macfarlane P. Using the WHO (Rose) Angina Questionnaire in Cardiovascular Epidemiology. *International Journal of Epidemiology* 1989;18(3):607-13. doi: 10.1093/ije/18.3.607
58. Goldberg D, PA W. User Guide to the General Health Questionnaire. Windsor, UK: NFER-Nelson, 1988.
59. Mont D. Measuring disability prevalence: World Bank 2007.
60. Burgard SA, Chen PV. Challenges of health measurement in studies of health disparities. *Social science & medicine (1982)* 2014;106:143-50. doi: 10.1016/j.socscimed.2014.01.045 [published Online First: 01/31]
61. Altman BM, editor. *International Measurement of Disability: Purpose, Method and Application*: Springer, 2016.
62. Groce NE, Mont D. Counting disability: emerging consensus on the Washington Group questionnaire. *The Lancet Global Health* 2017;5(7):e649-e50. doi: 10.1016/S2214-109X(17)30207-3
63. Chan KS, Kasper JD, Brandt J, et al. Measurement Equivalence in ADL and IADL Difficulty Across International Surveys of Aging: Findings From the HRS, SHARE, and ELSA. *The Journals of Gerontology Series B: Psychological Sciences and Social Sciences* 2012;67B(1):121-32. doi: 10.1093/geronb/gbr133
64. Bago d'Uva T, O'Donnell O, van Doorslaer E. Differential health reporting by education level and its impact on the measurement of health inequalities among older Europeans. *International Journal of Epidemiology* 2008;37:1375-83. doi: 10.1093/ije/dyn146
65. Benzeval M, Kumari M, Jones AM. How Do Biomarkers and Genetics Contribute to Understanding Society? *Health Economics* 2016;25(10):1219-22. doi: 10.1002/hec.3400
66. Martin LG, Freedman VA, Schoeni RF, et al. Health and Functioning Among Baby Boomers Approaching 60. *The Journals of Gerontology Series B: Psychological Sciences and Social Sciences* 2009;64B(3):369-77. doi: 10.1093/geronb/gbn040
67. Kaye HS. Disability Rates For Working-Age Adults And For The Elderly Have Stabilized, But Trends For Each Mean Different Results For Costs. *Health Affairs* 2013;32(1):127-34. doi: 10.1377/hlthaff.2012.0533
68. Mood C. Logistic Regression: Why We Cannot Do What We Think We Can Do, and What We Can Do About It. *European Sociological Review* 2010;26(1):67-82.
69. Wasserstein RL, Lazar NA. The ASA's Statement on p-Values: Context, Process, and Purpose. *The American Statistician* 2016;70(2):129-33. doi: 10.1080/00031305.2016.1154108
70. Unal B, Critchley JA, Capewell S. Explaining the Decline in Coronary Heart Disease Mortality in England and Wales Between 1981 and 2000. *Circulation* 2004;109(9):1101-07. doi: 10.1161/01.cir.0000118498.35499.b2
71. Davies AR, Smeeth L, Grundy E. Contribution of changes in incidence and mortality to trends in the prevalence of coronary heart disease in the UK: 1996–2005. *European Heart Journal* 2007;28(17):2142-47.

For peer review only - http://bmjopen.bmj.com/site/about/guidelines.xhtml

72. Oyebode O. Cardiovascular disease. In: Craig R, Mindell J, eds. Health Survey for England, 2011, Volume 1: Health, social care and lifestyles: TSO 2012:21-62.
73. Davillas A, Benzeval M. Alternative measures to BMI: Exploring income-related inequalities in adiposity in Great Britain. *Social Science & Medicine* 2016;166:223-32. doi: http://dx.doi.org/10.1016/j.socscimed.2016.08.032
74. Read SH, Kerssens JJ, McAllister DA, et al. Trends in type 2 diabetes incidence and mortality in Scotland between 2004 and 2013. *Diabetologia* 2016;59(10):2106-13. doi: 10.1007/s00125-016-4054-9
75. McManus S, Bebbington P, Jenkins R, et al., editors. *Mental health and wellbeing in England: Adult Psychiatric Morbidity Survey 2014*. Leeds: NHS Digital, 2016.
76. Ross A, Kelly Y, Sacker A. Time trends in mental well-being: the polarisation of young people's psychological distress. *Social Psychiatry and Psychiatric Epidemiology* 2017;52(9):1147-58. doi: 10.1007/s00127-017-1419-4
77. Gili M, Roca M, Basu S, et al. The mental health risks of economic crisis in Spain: evidence from primary care centres, 2006 and 2010. *European Journal of Public Health* 2012;23(1):103-08. doi: 10.1093/eurpub/cks035
78. McBeth J, Jones K. Epidemiology of chronic musculoskeletal pain. *Best Practice & Research Clinical Rheumatology* 2007;21(3):403-25. doi: 10.1016/j.berh.2007.03.003
79. Harkness EF, Macfarlane GJ, Silman AJ, et al. Is musculoskeletal pain more common now than 40 years ago?: two population-based cross-sectional studies. *Rheumatology* 2005;44(7):890-95. doi: 10.1093/rheumatology/keh599
80. Palmer KT, Walsh K, Bendall H, et al. Back pain in Britain: comparison of two prevalence surveys at an interval of 10 years. *BMJ* 2000;320:1577-8.
81. Chaudhury M. Blood analytes. In: Sproston K, Primatesta P, eds. Health Survey for England, 2003, Vol 2: Risk Factors for Cardiovascular Disease: TSO 2004:241-88.
82. Steptoe A. Psychosocial biomarker research: integrating social, emotional and economic factors into population studies of aging and health. *Social Cognitive and Affective Neuroscience* 2011;6(2):226-33. doi: 10.1093/scan/nsq032
83. Begum A, Dewey M, Hassiotis A, et al. Subjective cognitive complaints across the adult life span: a 14-year analysis of trends and associations using the 1993, 2000 and 2007 English Psychiatric Morbidity Surveys. *Psychological Medicine* 2014;44(9):1977-87. doi: 10.1017/S0033291713002444
84. Gupta R, Sheikh A, Strachan DP, et al. Time trends in allergic disorders in the UK. *Thorax* 2007;62(1):91-96. doi: 10.1136/thx.2004.038844
85. Aitken GR, Roderick PJ, Fraser S, et al. Change in prevalence of chronic kidney disease in England over time: comparison of nationally representative cross-sectional surveys from 2003 to 2010. *BMJ Open* 2014;4(9) doi: 10.1136/bmjopen-2014-005480

For peer review only

For peer review only - http://bmjopen.bmj.com/site/about/guidelines.xhtml

WEB APPENDICES

Table of contents *(pages refer to within web appendices)*

Appendix 1: Working-age mortality trends 2
Appendix 2: Overall missingness in health measures 3
Appendix 3: Changing non-response & weights 6
Appendix 4: General self-reported health/disability 10
Appendix 5: Health measures 12
Appendix 6: Measures not included in the main paper 24
Appendix 7: Year-by-year trends 29
Appendix 8: Others' analyses over change over time using HSE data 35
Appendix 9: Summarising multiple measures 37
Bibliography for Web Appendices 42

For peer review only

For peer review only - http://bmjopen.bmj.com/site/about/guidelines.xhtml

Appendix 1: Working-age mortality trends

Mortality in general

Given debates about whether historic improvements in life expectancy are being sustained, particularly in the US and UK, [1 2] it is important to note that in the period under study in this paper, working-age life expectancy was increasing. This can be seen in data from the Human Mortality Database (May 2016 update) 1993-2013, using one-year age and one-year period. This data shows that increases in mortality are not found for working-age people as a whole in any major country – for example, standardised working-age death rates have declined by 23% in the US and 35% in the UK over 1993-2013.

Cause-specific mortality for the 0-64 population

The main text refers to cause-specific morality in several places, referring to the death rate among 0-64 year olds from cardiovascular disease (CVD), respiratory conditions, diabetes, and liver cirrhosis. These death rates refer to UK deaths within relevant ICD-10 codes (I00-I99 for CVD, J00-J99 for respiratory conditions, E10-E14 for diabetes), standardised to the European standard population, and taken from the World Health Organization European Office's Health for All Database (May 2016 version), http://www.euro.who.int/en/data-and-evidence/databases/european-health-for-all-database-hfa-db.

For peer review only - http://bmjopen.bmj.com/site/about/guidelines.xhtml

Appendix 2: Overall missingness in health measures

This appendix refers to overall item-level missingness; changing item- and unit-level missingness is covered in Appendix 3.

Interview measures

For those who took part in the initial face-to-face interview, the level of item missingness is shown below (including only those years in which each question was asked). This shows the item-missingness is generally very low – only 1 of the 30 measures variables have item-missingness greater than 1%.

Table 1: Missingness at the initial face-to-face interview

	n non-missing	*n* missing	% missingness
BMI	124,682	15,415	11.0%
Any recent CVD	43,274	354	0.8%
Recent high blood pressure	43,366	262	0.6%
Breathlessness-Grade 2+	25,620	68	0.3%
Breathlessness-Grade 3	25,620	68	0.3%
Recent heart attack/stroke	43,519	109	0.3%
COPD symptoms	25,631	57	0.2%
Recent angina	43,551	77	0.2%
Heart attack symptoms	43,595	33	0.1%
Angina symptoms	43,592	36	0.1%
Recent diabetes	66,637	54	0.1%
Mini stroke (TIA) symptoms	23,487	16	0.1%
Diagnosed asthma	41,225	28	0.1%
Wheezing stopping sleep	41,224	29	0.1%
Recent wheezing/asthma	41,224	29	0.1%
Locomotor limitation	25,347	10	0.0%
Self-care limitation	25,347	10	0.0%
Limitations in past 2wks	140,041	56	0.0%
Longstanding illness (LSI)	124,906	43	0.0%
Limiting LSI (LLSI)	104,798	36	0.0%
Any CVD LSI	124,912	37	0.0%
IHD/stroke LSI	124,912	37	0.0%
Mental health LSI	124,912	37	0.0%
Arthritis LSI	124,912	37	0.0%
Asthma LSI	124,912	37	0.0%
Diabetes LSI	124,912	37	0.0%
High blood pressure LSI	124,912	37	0.0%
Other musculoskeletal LSI	124,912	37	0.0%
Good general health	140,048	49	0.0%
Bad general health	140,048	49	0.0%

The only variable with noticeable missingness is BMI, which is understandable as this involves the interviewer taking height and weight measurements rather than simply asking for a verbal response. There are various reasons why people do not have a BMI measurement:

For peer review only - http://bmjopen.bmj.com/site/about/guidelines.xhtml

- *High weight*: people with a very high weight are not weighed in HSE 'because the scales are inaccurate above this level', but the definition of this changed (from 130kg before 2011 to 200kg afterwards). This only applied to <0.1% of respondents 2012-14.
- *Difficult to take measurement*: other respondents (between 3.8% and 6.1% depending on the year) have no valid BMI measurement because height or weight measures were not attempted, attempted but not obtained or useable, because the respondent was pregnant, or the respondent was too sick or unsteady.
- *Refusal*: the most common reason for no BMI measurement is an outright refusal (including those refusing out of anxiety, though this tends to be a minor reason). Refusal rates are 8.3% in 2014.

Self-completion measures

For those who completed the self-completion booklet, the level of item missingness is shown in the table below.

Table 2: Missingness within the self-completion booklet

	n *non-missing*	*n* *missing*	% *missingness*
Psychological distress symptoms	108,324	2,462	2.2%
Problems washing/dressing today	62,703	1,310	2.1%
Anxiety/depression	62,725	1,288	2.0%
Problems w/activities	62,742	1,271	2.0%
Problems walking about today	62,772	1,241	1.9%
Pain	62,783	1,230	1.9%

Item missingness is relatively low compared to missingness from not completing the self-completion survey (51.5% of respondents in 2014).

Nurse visit measures

For those who took part in the nurse visit, the level of item missingness is shown in the table below.

Table 3: Missingness within the nurse visit

	n *non-missing*	*n* *missing*	% *missingness*
Biomarker high blood pressure	87,726	15,517	15.0%
High waist-hip ratio	78,637	2,664	3.3%

This shows that far more people have missing observations for measured high blood pressure than for their waist-hip ratio. This is despite the fact that we explicitly INCLUDE those who are on blood pressure-lowering drugs (about 5% of the sample at the start of the period and 10% at the end), on the grounds that their lowered blood pressure still conveys useful information about their health state. The main reason for the remaining high level of missingness is because people have recently exercised, smoked, drank or ate (12.2%).

Blood sample measures

For those from whom a blood sample was taken, the level of item missingness is shown in the table below.

For peer review only - http://bmjopen.bmj.com/site/about/guidelines.xhtml

Table 4: Missingness within the blood sample

	n *non-missing*	*n* *missing*	% *missingness*
Raised fibrinogen	16,166	3,341	17.1%
Raised C-reactive protein	17,814	1,693	8.7%
Glycated haemoglobin	28,810	1,436	4.8%
Anaemia	20,302	939	4.4%
Iron deficiency	20,375	866	4.1%
Low HDL cholesterol	36,076	1,406	3.8%
High total cholesterol	43,409	1,472	3.3%

All of these measures are affected by problems in transferring and storing the blood sample and with the measurement process, which results in problems with 3-10% of the blood samples depending on the measure and year. As for blood pressure, we explicitly INCLUDE those who are on lipid-lowering drugs (0.4% 1994 to 7.9% 2014), on the grounds that their changed cholesterol level still conveys useful information about their health state. Item missingness is highest for fibrinogen, which not only has high rates of such failures (7.0-9.5%), but also has ineligibility due to likely infection (from raised CRP, 3.6-5.6% of those with blood samples) and taking drugs that affect the reading (3.7% to 7.7% dependent on the year). Item missingness is also high for C-reactive protein (CRP), which also excludes those with likely infections.

Dealing with item-level missingness

Because of the high level of item non-response for certain measures (BMI, high blood pressure, fibrinogen, and CRP), and moderate level for others (other blood sample biomarkers and waist-hip ratio) – and because of evidence of changing non-response at various stages of the survey process – non-response weights were created to try to correct for any biases that these introduce. This is described in further detail in Appendix 3.

For peer review only

For peer review only - http://bmjopen.bmj.com/site/about/guidelines.xhtml

Appendix 3: Changing non-response & weights

This appendix focuses on *changes* in unit-level non-response at different stages of HSE.

Changing non-response

Sample frame coverage

As noted in the main paper, HSE is a household sample that excludes those in communal establishments. If we combine data from the 1991, 2001 and 2011 Censuses,[1] the communal population is as follows:

Table 5: Population in communal establishments over time (all working-age) and by age (in 2011)

		Education	Medical/ care	Defence	Prison	Other / not stated
**All working age**	*1991*	21,149	86,683	44,562	13,279	63,340
	2001	204,606	73,705	46,428	44,185	86,288
	2011	328,772	76,026	41,659	47,849	61,124
**16-24**	*2011*	305,154	9,346	22,677	12,607	25,673
**25-34**	*2011*	20,443	12,000	15,025	15,407	14,417
**35-49**	*2011*	2,663	26,796	3,725	14,725	14,708
**50-SPA[1] (est)**	*2011*	512	27,884	232	5,110	6,326

[1] SPA = State Pension Age, which is 60 for women and 65 for men. This is estimated because the Census totals are given for 50-64 year olds, so we have excluded 1/3 of women aged 50-64 from these totals.

This shows two things. Firstly, that there was a sharp rise in the working-age population in communal establishments 1991-2001 (from 230k to 560k), which was concentrated (>90% of the rise) among education-related communal establishments – although this is perhaps a slight overestimate given a definition change in the Census data.[2] Secondly, looking at education-related communal establishments in 2011, these are overwhelmingly (>90%) among 16-24 year olds. It therefore seems likely that the exclusion of communal establishments in HSE will lead to biases in young adults, and we therefore exclude 16-24 year olds from the trend analyses.

[1] Data are obtained from nomis on 6/8/2015, from Census tables DC1104EW and DC4210EWla (2011), S126 (2011) and L03/L04/L05 (2001).
[2] The guide to Census SARs notes, *"In the 1991 Census, students and schoolchildren were treated as usually resident at their 'home' or vacation address. In the 2001 census students and schoolchildren in full-time education studying away from the family home were enumerated as resident at their term-time address."* See https://census.ukdataservice.ac.uk/use-data/guides/microdata/comparability-91-01 [accessed 1/11/2016].

For peer review only - http://bmjopen.bmj.com/site/about/guidelines.xhtml

Changing unit non-response within the sample frame

As noted in the main paper, HSE supplies non-response weights from 2003, including adjustments for non-response to the nurse visit and blood sample using health and socioeconomic status from the initial interview. However, there had been a substantial decline in response rates prior to 2003, as shown in the table below:

Table 6: Response rates to HSE

	Household	Individual	Self-comp.	BMI	Nurse	Blood
1991	85.3%	81.1%				
1992	81.8%	77.4%				
1993	80.8%	75.7%				
1994	77.4%	71.6%	71.2%	67.1%	63.3%	53.3%
1995	78.3%	72.9%	72.0%	66.8%	63.7%	
1996	79.4%	74.7%	73.7%	69.6%	66.1%	
1997	76.0%	71.1%	69.8%	66.9%	64.0%	
1998	74.0%	68.9%	66.7%	63.3%	59.6%	49.0%
1999	76.2%	70.3%	68.5%	63.6%		
2000	75.5%	68.4%	65.8%	60.5%	58.2%	
2001	74.2%	67.1%	64.5%	60.1%	54.2%	
2002	74 %	67 %	64.4%	59.6%	54.3%	
2003	72.7%	66.4%	64.1%	59.7%	52.2%	39.9%
2004	72.4%	65.6%	62.4%	56.1%		
2005	71.4%	64.1%	60.6%	54.8%	46.7%	
2006	68.1%	60.5%	57.7%	52.8%	45.4%	34.7%
2007	65.7%	58.3%	56.1%	51.3%	42.6%	
2008	64.5%	57.9%	55.9%	50.0%	41.5%	30.4%
2009	67.6%	61.0%	58.7%	52.5%	43.1%	33.7%
2010	66.1%	58.7%	54.9%	49.3%	39.1%	29.9%
2011	65.7%	58.9%	54.3%	49.0%	39.4%	29.8%
2012	64.1%	56.3%	52.5%	47.4%	36.3%	27.9%
2013	63.8%	57.6%	54.2%	49.3%	40.1%	31.2%
2014	61.6%	55.5%	51.5%	48.4%	37.3%	28.7%

In general these trends are due to increases in refusal rates. However, the blood sample response rate is affected by two noticeable changes in eligibility over this period (people who are pregnant or who had blood/clotting disorders were ineligible throughout):

1. In 1998, people who had ever had an epileptic fit were excluded from the blood sample. This raised the ineligibility rate to 3.5% of the sample in 1998, from 0.6% in 1994.
2. In 2010, this was then relaxed so that those who had had an epileptic fit more than 5 years ago were again included in the blood sample. This lowered the ineligibility rate from 3.1% in 2009 to 2.4% in 2010.

Changing item non-response within responding people

There are also changes over time in item non-response (further detail on overall item non-response is given in Appendix 2). This includes:

- *BMI:* there has been little systematic trend in one reason for the absence of a BMI measure (difficulty in taking BMI measurements). However, there are trends in other reasons:

For peer review only - http://bmjopen.bmj.com/site/about/guidelines.xhtml

  - *High weight*: the definition of high weight changed from 130kg before 2011 to 200kg afterwards. 1.0% of respondents were not weighted for this reason in 2010, which fell to <0.1% 2012-14.
  - *Refusal*: in line with the general participation rates at each stage of the interview above, BMI refusal rates rose sharply from 1.9% in 1994 to a peak of 11.5% in 2011, and remain at 8.3% in the 2014 data.
- *Psychological distress:* similarly to wider participation rates at each stage of the survey, item missingness within the self-completion survey does increase over time (e.g. for psychological distress symptoms, from 1.8% 1994 to 5.9% 2014).
- *Measured high blood pressure:* there was a noticeable rise over time in exclusion of high blood pressure measures on the grounds that people recently exercised, smoked, drank or ate (from 6.1% to 13.6%).
- *Fibrinogen:* taking drugs that affect the fibrinogen reading rose from 3.7% 1994 to 7.7% 2009.

Creating non-response weights

To increase comparability over time, we create new weights 1994-2014 in several phases.

First-stage non-response weights

Firstly, we created a selection weight because some households were slightly more likely to be interviewed than others. (Until 2009, only three households at each address were interviewed. Those living at addresses with many households are therefore less likely to be interviewed). NatCen supplied selection weights for 2004-2013 to enable this (funded by this project), which are not available on the public HSE datasets.

Secondly, after adjusting for the selection weight, we created new individual-level (inverse probability) weights to match population age-sex-region totals in each year. Population data are annual mid-year population estimates from nomis. NatCen added the region variable for the 1994-1997 datasets to the public HSE datasets to enable this.

Second-stage non-response weights

After the first-stage adjustment for individual non-response, for the later stages of the interview (self-completion, BMI measurement, nurse visit, blood sample), we created a further weight that adjusts for non-response among those responding to the individual interview. This is based on a logit regression model to predict that stage of response based on:

- Age and gender (4 age group categories interacted with gender);
- Qualifications (degree or FT student / A-level or above / other qualifications / no qualifications);
- Household type (presence of other adults in the household);
- Employment status (yes/no);
- Smoking (never regular smoker / ex-regular smoker / current regular smoker); and
- Self-reported general health (bad or very bad health vs. other categories).

On the basis of these criteria, we create inverse probability weights – that is, we create a predicted probability of response for each respondent based on the logit regression model, and then create a

For peer review only - http://bmjopen.bmj.com/site/about/guidelines.xhtml

weight that is the inverse of this predicted probability. The revised weights are included in the Stata code to enable replication of the full paper.

Final sample size

The final sample size is as follows:

Table 7: HSE sample size in each year

	Interview	Self-completion	Nurse visit	Blood sample
1994	9,948	9,884	8,786	7,399
1995	10,167	10,049	8,881	
1996	10,401	10,269	9,206	
1997	5,563	5,458	5,005	
1998	10,177	9,843	8,805	7,236
1999	5,008	4,884		
2000	5,188	4,993	4,417	
2001	10,002	9,613	8,079	
2002	4,662	4,482	3,775	
2003	9,420	9,089	7,395	5,665
2004	4,165	3,961		
2005	4,810	4,548	3,505	
2006	8,825	8,420	6,622	5,064
2007	4,198	4,039	3,064	
2008	9,242	8,922	6,625	4,845
2009	2,795	2,689	1,973	1,542
2010	5,120	4,794	3,411	2,610
2011	5,258	4,853	3,518	2,667
2012	4,936	4,605	3,188	2,447
2013	5,303	4,992	3,691	2,875
2014	4,909	4,552	3,297	2,531
**Total**	**140,097**	**134,939**	**103,243**	**44,881**

For peer review only

For peer review only - http://bmjopen.bmj.com/site/about/guidelines.xhtml

Appendix 4: General self-reported health/disability

Trends in seven general health/disability measure are available in HSE:

Table 8: HSE general health measures

Measure	Operationalisation (years available)
Good general health	Health in general is 'good' or 'very good' (1994-2014)
Bad general health	Health in general is 'bad' or 'very bad' (1994-2014)
Longstanding illness (LSI)	Any long-standing illness, disability or infirmity (1994-2011)
Limiting LSI (LLSI)	LSI limits activities in any way (1996-2011)
Problems with activities-some	Some problems with performing usual activities (1996-2014)
Problems with activities-unable	Unable to perform usual activities (1996-2014)
Limitations in past 2wks	Cut down on activities in past 2wks due to LSI or other illness/injury (1994-2014)

See Web Appendix 5 for full details on all measures .

Trends for these measures are shown in Table 9 below. Looking first at good general health, the table shows the trend from 1994-6, when 80.9% reported good general health. By 2011-14, there had been a decline of 0.8 percentage points. When we adjust for the changing age and sex distribution of the working-age population (labelled 'Adj.' in Table 1), the decline is only 0.1%, with a wide confidence interval (-0.9 to +0.7%), and there is therefore little evidence for any systematic trend.

Table 9: Changes over time in general health

	Starting period		Change from start to end period			
	Period	Prevalence	End period	Raw change	Adj.[a] change	Adj. change 95% CI
Good general health	1994-96	80.9%	2011-14	-0.8%	-0.1%	[-0.9, 0.7%]
Bad general health	1994-96	4.4%	2011-14	1.3%	1.0%	[0.6, 1.5%]
Longstanding illness (LSI)	1994-96	36.2%	2011-14	-1.0%	-2.0%	[-3.7, -0.3%]
Limiting LSI (LLSI)	1994-96	21.4%	2011-14	-2.9%	-3.6%	[-5.2, -2.1%]
Problems w/activities-some	1994-96	14.8%	2011-14	-1.2%	-1.8%	[-2.8, -0.8%]
Problems w/activities-unable	1994-96	1.9%	2011-14	-0.6%	-0.8%	[-1.1, -0.4%]
Limitations in past 2wks	1994-96	14.7%	2011-14	-0.1%	-0.3%	[-1.0, 0.4%]

[a] 'Adj.' = adjusted for changing age and sex distribution of the working-age population.

For several of the general health measures, there is evidence of change over this period – but interpreting this is difficult, because the trends are in opposite directions. There is strong evidence for a *rise* in bad general health (a rise of 0.6-1.5% from a base of 4.4%), yet equally strong evidence for a *decline* in having problems with everyday activities (at both levels of severity), and being limited in activities by a longstanding illness. This shows the challenges in tracking population morbidity change through general, non-specific measures, which are likely to be as influenced by changes in reporting styles as much as changes in morbidity *per se*.

As an aside, UK Government publications have made claims based on healthy/disability-free life expectancy – sometimes using these to argue that morbidity has been improving [3], but more recently to argue that morbidity has been deteriorating.[4-6] However, these trends are potentially misleading: they include older people as well as the working-age population; they confuse a

For peer review only - http://bmjopen.bmj.com/site/about/guidelines.xhtml

combined mortality-morbidity measure with morbidity; and they are based on self-reports of global health that are unreliable, as we show here and discuss in the main text.

For peer review only

For peer review only - http://bmjopen.bmj.com/site/about/guidelines.xhtml

Appendix 5: Health measures

We systematically searched HSE questions, and have included every morbidity measure that is comparable over a significant duration. We have excluded questions only available for short time frames (ADLs 2012-14, EQ-5D visual analogue scale 2008-14, SF-12 1996-2000, eczema/hayfever 1995-2001, breathlessness 1991-98 and 1995-2001, lung function 1995-2001, bladder limitations 1995-2001, LDL cholesterol, triglycerides and glucose 1999-2003, IgE 1996-2002 and an alternate measure of high blood pressure 2009-14), with the exception of five key measures of activity limitations 1995-2001. We have also excluded questions that are not direct measures of health (medication or health service use, demispan, health risk factors such as fractures, accidents, alcohol/tobacco use (including biomarkers), physical activity, and wellbeing).

Short summaries of the resulting 39 measures are given in this paper, and full details are given in the table below. Measures are taken from the initial face-to-face survey unless otherwise specified. The Stata code to create these variables in consistent form from the publicly available HSE files are available from OSF[7] and www.benbgeiger.co.uk.

Measure	Details
Activity limitations and MSDs	
Problems walking today	In the self-completion survey in 1996, 2003-6, 2008, 2010-12 and 2014, respondents were asked *'Now we would like to know how your health is today. Please answer ALL the questions. By ticking one box for each question below, please indicate which statements best describe your own health state today':*<br>- *"I have no problems in walking about"*<br>- *"I have some problems in walking about"*<br>- *"I am confined to bed"*<br>[This is part of the widely-used EQ-5D health status indicator [8]. However, for the purposes of this paper we have separated the individual measures that make up the EQ-5D in order to compare these to similar indicators of morbidity within each domain].<br>People are classified as having a problem with self-care today if they had some problems walking about or were confined to bed.
Locomotor limitation	This is based on the personal care disability scale used in the 2001 HSE report [9]. Respondents in 1995, 2000 and 2001 were asked if any of the following applied to them (interviewers were instructed to ignore temporary disabilities that are expected to last less than one year):<br>- *"Cannot walk 200 yards or more on own without stopping or discomfort"*. People who reported a limitation were asked if they used a walking aid, and if they did, were then asked if they could walk 200 yards without the walking aid.<br>- *"Cannot walk up and down a flight of 12 stairs without resting"*<br>- *"Cannot bend down and pick up a shoe from the floor when standing"*<br>People are classified as having a locomotor limitation if they reported ANY of these limitations.
Problems with washing/dressing today	In the self-completion survey in 1996, 2003-6, 2008, 2010-12 and 2014, respondents were asked *'Now we would like to know how your health is today. Please answer ALL the questions. By ticking one box for each question below, please indicate which statements best describe your own health state today':*<br>- *"I have no problems with self-care"*<br>- *"I have some problems washing or dressing myself"*<br>- *"I am unable to wash or dress myself"*<br>[This is part of the widely-used EQ-5D health status indicator [8]. However, for the purposes of this paper we have separated the individual measures that make up the EQ-

For peer review only - http://bmjopen.bmj.com/site/about/guidelines.xhtml

	5D in order to compare these to similar indicators of morbidity within each domain].<br>People are classified as having a problem with self-care today if they had some problems washing/dressing or were unable to wash/dress themselves.
Self-care limitation	This is based on the personal care disability scale used in the 2001 HSE report [9]. Respondents in 1995, 2000 and 2001 were asked if any of the following applied to them (interviewers were instructed to ignore temporary disabilities that are expected to last less than one year):<br>- *"Cannot get in and out of bed on own without difficulty"*<br>- *"Cannot get in and out of a chair without difficulty"*<br>- *"Cannot dress and undress without difficulty"*<br>- *"Cannot wash hands and face without difficulty"*<br>- *"Cannot feed, including cutting up food without difficulty"*<br>- *"Cannot get to and use toilet on own without difficulty"*<br>People are classified as having a self-care limitation if they reported ANY of these limitations.
Pain<br>*(any / extreme)*	In the self-completion survey in 1996, 2003-6, 2008, 2010-12 and 2014, respondents were asked *'Now we would like to know how your health is today. Please answer ALL the questions. By ticking one box for each question below, please indicate which statements best describe your own health state today'*:<br>- *"I have no pain or discomfort"*<br>- *"I have moderate pain or discomfort"*<br>- *"I have extreme pain or discomfort"*<br>[This is part of the widely-used EQ-5D health status indicator [8]. However, for the purposes of this paper we have separated the individual measures that make up the EQ-5D in order to compare these to similar indicators of morbidity within each domain].<br>Two outcome measures are based on this: whether people have any pain (the 2nd and 3rd categories combined), and whether they have extreme pain (3rd category only).
Arthritis LSI	Every year 1994-2011, people who report a longstanding illness (LSI) are then asked, *'what is the matter with you?'*; up to 6 responses are then coded by the interviewer into a consistent coding frame based on the International Classification of Diseases.<br>The arthritis LSI measure is based on the group labelled *'Arthritis/rheumatism/fibrositis'*, which as of 2011 includes: Arthritis as result of broken limb; Arthritis/rheumatism in any part of the body; Gout; Osteoarthritis, rheumatoid arthritis, polymyalgia rheumatic; Polyarteritis Nodosa; Psoriasis arthritis; Rheumatic symptoms; and Still's disease.<br>While the LSI coding frame generally stays consistent over this period, interpretation of 'LSI arthritis' is complicated by two changes: Gout and Polyarteritis Nodosa are moved into this code (the documentation is not clear on whether this occurred in 2000 or 2001).
Other musculoskeletal LSI	People who report a longstanding illness (LSI) are then asked, *'what is the matter with you?'*; up to 6 responses are then coded by the interviewer into a consistent coding frame based on the International Classification of Diseases.<br>The other musculoskeletal LSI measure is based on the groups labelled *'Back problems/slipped disc/spine/neck'* and *'Other problems of bones/joints/muscles'*, which as of 2011 includes: Brittle bones, osteoporosis; Bursitis, housemaid's knee, tennis elbow; Cartilage problems; Chondrodystrophia; Chondromalacia; Cramp in hand; Deformity of limbs eg. club foot, claw-hand, malformed jaw; Delayed healing of bones or badly set fractures; Deviated septum; Disc trouble; Dislocations eg. dislocation of hip, clicky hip, dislocated knee/finger; Disseminated lupus; Dupuytren's contraction; Fibromyalgia; Flat feet, bunions; Fracture, damage or injury to extremities, ribs, collarbone, pelvis, skull, eg. knee injury, broken leg, gun shot wounds in leg/shoulder, can't hold arm out flat - broke it as a child, broken nose; Frozen shoulder; Hip infection, TB hip; Hip replacement (nes); Legs won't go, difficulty in walking; Lumbago, inflammation of spinal joint; Marfan Syndrome; Osteomyelitis; Paget's disease; Perthe's disease; Physically handicapped (nes); Pierre Robin syndrome; Prolapsed invertebral discs; Schlatter's disease; Schuermann's

For peer review only - http://bmjopen.bmj.com/site/about/guidelines.xhtml

	disease; Sever's disease; Spondylitis, spondylosis; Stiff joints, joint pains, contraction of sinews, muscle wastage; Strained leg muscles, pain in thigh muscles; Systemic sclerosis, myotonia (nes); Tenosynovitis; Torn muscle in leg, torn ligaments, tendonitis; Walk with limp as a result of polio, polio (nes), after affects of polio (nes); Weak legs, leg trouble, pain in legs; and Worn discs in spine - affects legs. The code explicitly excludes: Damage/injury to spine results in paralysis; Sciatica or trapped nerve in spine; and Muscular dystrophy.
Circulatory	
High blood pressure LSI	Every year 1994-2011, people who report a longstanding illness (LSI) are then asked, *'what is the matter with you?'*; up to 6 responses are then coded by the interviewer into a consistent coding frame based on the International Classification of Diseases.<br>The high blood pressure LSI measure is based on the group labelled *'Hypertension/high blood pressure/blood pressure (nes)'*, which as of 2011 includes only the conditions listed in the group label.
Recent high blood pressure	Respondents in 1994, 1998, 2003, 2006 and 2009-2014 were asked a series of questions on whether they have high blood pressure:<br>- *"Do you now have, or have you ever had… high blood pressure (sometimes called hypertension)?"*<br>- Those responding 'yes' were then asked *"Were you told by a doctor or nurse that you had high blood pressure?"*<br>- Women responding 'yes' were then asked, *"Can I just check, were you pregnant when you were told that you had high blood pressure?"*, and those responding 'yes' were then asked *"Have you ever had high blood pressure apart from when you were pregnant?"*<br>- Finally, those with doctor-diagnosed high blood pressure (excluding only when pregnant were asked: *"Are you currently taking any medicines, tablets or pills for high blood pressure?*", and those saying 'no' (or not giving an answer) were then asked, *"Do you still have high blood pressure?"*<br>People were considered to have recent high blood pressure if they said they had ever been diagnosed as having high blood pressure by a doctor (excluding when pregnant), and that they still have high blood pressure or are currently taking medicines for it.<br>While the question wording has stayed consistent, a discontinuity seems to be introduced by a change in question context. In some years (1994, 1998, 2003, 2006 and 2011), this question was preceded by a question that asked, *"May I just check, have you ever had your blood pressure measured by a doctor or nurse?"* (and then for those saying yes, they were asked how recently this was, and whether they were told that it was *'normal (alright/fine), higher than normal, lower than normal, or were you not told anything?'*). However, in other years (2009-10, 2012-14), this question was not asked. Given the way in which context can affect question interpretation, we treat these as two separate measures of recent high blood pressure.
Biomarker high blood pressure	During the nurse visit (which took place for all consenting respondents in all years except 1999, 2002 and 2004, when the nurse visit focussed on particular subsamples), respondents' blood pressure was measured.<br>High blood pressure is defined as a systolic blood pressure >= 140mmHg and diastolic blood pressure >= 90mmHg following HSE established practice, in turn following [10].<br>The measurement of blood pressure changed in 2003, from a Dinamap monitor to an Omron monitor. A conversion is available between the two monitors based on a calibration study, and this has been regularly used by the HSE team to produce continuous trends in blood pressure – see www.hscic.gov.uk/catalogue/PUB00480. For adults, the conversion is as follows:<br>○ For systolic blood pressure: *Predicted Omron=8.90 (SE=2.94) + 0.91 (SE=0.02) * Dinamap.*<br>○ For diastolic blood pressure: *Predicted Omron=19.78 (SE=1.86) + 0.73 (SE=0.03) * Dinamap.*<br>There are several reasons why respondents who had a nurse visit do not have a valid

For peer review only - http://bmjopen.bmj.com/site/about/guidelines.xhtml

	blood pressure measurement – these are discussed in the Web Appendices 2 and 3.
High cholesterol	In the years 1994, 1998, 2006, and 2008-14, blood samples were obtained during the nurse visit, which were then analysed for total cholesterol. A high level of total cholesterol ('hypercholesterolaemia') is an established risk factor for CVD, and high cholesterol is defined following conventional practice at the NICE guidance 'audit level' of 5mmol/L or above [11 12].<br>The measurement of cholesterol changed slightly in 2010 when a new laboratory was used. This resulted in values that are an average of 0.1mmol/L higher, and later values are therefore adjusted by this amount to maintain comparability over time as in [11].
Low HDL cholesterol	In the years 1994, 1998, 2006, and 2008-14, blood samples were obtained during the nurse visit, which were then analysed for high density lipoprotein (HDL) cholesterol. HDL cholesterol *reduces* the risk of CVD (it carries cholesterol away from the arteries towards the liver), and it is therefore low HDL cholesterol that indicates poorer health; low HDL cholesterol is here defined as 1 mmol/L or less [11 12].<br>The measurement of HDL cholesterol changed slightly in 2010 when a new laboratory was used. This resulted in values that are an average of 0.1mmol/L lower, and later values are therefore adjusted by this amount to maintain comparability over time as in [11].
Recent heart attack/stroke	Respondents in 1994, 1998, 2003, 2006 and 2011 were asked a series of questions on whether they have had a heart attack (within a battery of questions about different types of heart disease):<br>- *"Have you ever had a heart attack (including myocardial infarction or coronary thrombosis)?"*<br>- Those responding 'yes' were then asked *"Were you told by a doctor that you had a Heart Attack (including myocardial infarction or coronary thrombosis)?"*<br>- Those with doctor-diagnosed angina were asked, *"Have you had a heart attack (including myocardial infarction and coronary thrombosis) during the past 12 months?"*<br>Respondents in these years were similarly asked about stroke:<br>- *"Have you ever had a stroke?"*<br>- Those responding 'yes' were then asked, *"Were you told by a doctor that you had a stroke?"*<br>- Those with doctor-diagnosed stroke were asked, *"Have you had a stroke during the past 12 months?"*<br>People were considered to have recent IHD or stroke if they said they had ever been diagnosed as having stroke or a heart attack by a doctor, and that they have had a heart attack or stroke during the past 12 months.
Recent angina	Respondents in 1994, 1998, 2003, 2006 and 2011 were asked a series of questions on whether they have angina (within a battery of questions about different types of heart disease):<br>- *"Have you ever had angina?"*<br>- Those responding 'yes' were then asked *"You said that you had Angina. Were you told by a doctor that you had Angina?"*<br>- Those with doctor-diagnosed angina were asked, *"Have you had angina during the past 12 months?"*<br>People were considered to have recent angina if they said they had ever been diagnosed as having angina by a doctor, and that they have had it during the past 12 months.
IHD LSI	Every year 1994-2011, people who report a longstanding illness (LSI) are then asked, *'what is the matter with you?'*; up to 6 responses are then coded by the interviewer into a consistent coding frame based on the International Classification of Diseases.<br>The IHD LSI measure is based on the groups labelled *'Stroke/cerebral haemorrhage/cerebral thrombosis'* and *'Heart attack/angina'*. As of 2011 this includes: Cerebro-vascular accident; Coronary thrombosis, myocardial infarction; Heart attack/angina; Hemiplegia, apoplexy, cerebral embolism; Stroke/cerebral haemorrhage/cerebral thrombosis; and Stroke victim - partially paralysed and speech difficulty.
Recent	Respondents in 1994, 1998, 2003, 2006 and 2011 were asked a series of questions on

For peer review only - http://bmjopen.bmj.com/site/about/guidelines.xhtml

cardiovascular disease (CVD)	different types of heart disease – including angina; heart attack (including myocardial infarction or coronary thrombosis); a heart murmur; abnormal heart rhythm; or other heart trouble. For EACH of these, they were asked:<br>- *"Have you ever had <type of heart disease>?"*<br>- Those responding 'yes' were then asked *"You said that you had <type of heart disease>. Were you told by a doctor that you had <type of heart disease>?"*<br>- For heart murmurs only, women saying they had doctor-diagnosed heart murmurs were asked if they were pregnant when told this, and if so, whether they were ever told they had a heart murmur when they were not pregnant.<br>- Those with doctor-diagnosed heart disease (excluding heart murmurs when pregnant) were asked, *"Have you had <type of heart disease> during the past 12 months?"*<br>People were considered to have recent CVD if they said they had a doctor-diagnosed heart condition and that they had had this during the past 12 months.
Cardiovascular (CVD) LSI	Every year 1994-2011, people who report a longstanding illness (LSI) are then asked, *'what is the matter with you?'*; up to 6 responses are then coded by the interviewer into a consistent coding frame based on the International Classification of Diseases.<br>The CVD LSI measure is based on the groups labelled *'Stroke/cerebral haemorrhage/cerebral thrombosis', 'Heart attack/angina', Hypertension/high blood pressure/blood pressure (nes)', 'Other heart problems', 'Piles/haemorrhoids incl. Varicose Veins in anus', 'Varicose veins/phlebitis in lower extremities', and 'Other blood vessels/embolic'*. As of 2011 this includes: Aorta replacement; Aortic valve stenosis; Aortic/mitral valve regurgitation; Arterial thrombosis; Arteriosclerosis, hardening of arteries (nes); Artificial arteries (nes); Atrial Septal Defect (ASD); Blocked arteries in leg; Blood clots (nes); Cardiac asthma; Cardiac diffusion; Cardiac problems, heart trouble (nes); Cerebro-vascular accident; Coronary thrombosis, myocardial infarction; Dizziness, giddiness, balance problems (nes); Hand Arm Vibration Syndrome (White Finger); Hardening of arteries in heart; Heart attack/angina; Heart disease, heart complaint; Heart failure; Heart murmur, palpitations; Hemiplegia, apoplexy, cerebral embolism; Hole in the heart; Hypersensitive to the cold; Hypertension/high blood pressure/blood pressure (nes); Intermittent claudication; Ischaemic heart disease; Low blood pressure/hypertension; Mitral valve stenosis; Pacemaker; Pains in chest (nes); Pericarditis; Piles/haemorrhoids incl. Varicose Veins in anus; Poor circulation; Pulmonary embolism; Raynaud's disease; St Vitus dance; Stroke victim - partially paralysed and speech difficulty; Stroke/cerebral haemorrhage/cerebral thrombosis; Swollen legs and feet; Tachycardia, sick sinus syndrome; Telangiectasia (nes); Thrombosis (nes); Tired heart; Valvular heart disease; Valvular heart disease; Varicose veins in Oesophagus; Varicose veins/phlebitis in lower extremities; Various ulcers, varicose eczema; Weak heart because of rheumatic fever; Wolff - Parkinson - White syndrome; and Wright's syndrome. It explicitly excludes balance problems due to ear complaint & haemorrhage behind eye.<br>While the LSI coding frame generally stays consistent over this period, interpretation of 'IHD LSI' is complicated by two changes: 'Too much cholesterol in blood' is included in this category in 1994 only, and Polyarteritis Nodosa is later moved into this code (the documentation is not clear on whether this occurred in 2000 or 2001).
Angina symptoms	This is taken from the Rose Angina questionnaire [13 14]. Respondents in 1994, 1998, 2003, 2006 and 2011 were asked a series of questions about *symptoms* of heart trouble (rather than whether they had been diagnosed):<br>- *"I am now going to ask you some questions mainly about symptoms of the chest. Have you ever had any pain or discomfort in your chest?"*<br>- Those that said 'yes' were asked:<br>○ *Do you get it when you walk uphill or hurry? Yes | No | Sometimes/ Occasionally | Never walks uphill or hurries | (Cannot walk)"*. If sometimes/occasionally, they were asked: *"Does this happen on most occasions?"*<br>○ If not 'no' to having pain/discomfort in their chest, they were asked: *"Do you get it when you walk at an ordinary pace on the level? Yes | No |*

For peer review only - http://bmjopen.bmj.com/site/about/guidelines.xhtml

	Sometimes/Occasionally | Never walks at an ordinary pace on the level". If sometimes/occasionally, they were asked: *"Does this happen on most occasions?"*<br>- Those who every had pain/discomfort when walking uphill/hurrying or walking at ordinary pace on the level were asked:<br>○ *"What do you do if you get it while you are walking? Do you stop, slow down or carry on?"* (If respondents were unsure, they were asked, *"What do you do on most occasions?")*<br>○ Those who said they stop or slow down were asked, *"If you stand still does the pain go away or not?"* (If respondents were unsure, they were asked, *"What happens to the pain on most occasions?").* If the pain goes away, they were asked, *"How soon does the pain go away? Does it go in 10 minutes or less, or more than 10 minutes?"*<br>○ Those who said the pain goes away in 10 minutes or less were asked, *"Will you show me where you get this pain or discomfort? Where else"* The interviewer then coded the site as Sternum (upper or middle) | Sternum lower | Left anterior chest | Left arm | Right anterior chest | Right arm | (Somewhere else).<br>Following the HSE reports, possible angina is defined as chest pain or discomfort that (i) includes either the sternum or the left arm and left anterior chest; (ii) is prompted by hurrying or walking uphill (or by walking on the level, for those who never attempt more); (iii) makes the respondent either stop or slacken pace; and (iv) usually disappears in 10 minutes or less when they stand still.
Heart attack symptoms	This is taken from the Rose Angina questionnaire. Respondents in 1994, 1998, 2003, 2006 and 2011 were asked, *"Have you ever had a severe pain across the front of your chest lasting for half an hour or more?"* As in the 2006 HSE report, those responding 'yes' are treated as having a possible heart attack (myocardial infarction).
Mini stroke (TIA) symptoms	Respondents in 2003, 2006 and 2011 were asked:<br>○ *"In the last twelve months, have you had a sudden attack of weakness or numbness on one side of the body?"*<br>○ *"Have you had a sudden attack of slurred speech or difficulty in finding words in the last twelve months?"*<br>○ *"Have you had a sudden attack of vision loss or blurred vision in one or both eyes in the last twelve months?"*<br>People reporting ANY of these symptoms were considered as possibly having had a transient ischaemic attack (TIA), often called a 'mini stroke'.
Respiratory	
COPD symptoms	Respondents in 1995, 1996 and 2010 were asked:<br>○ *"Do you usually cough first thing in the morning in the winter?"* (In 2010 only, respondents had previously been asked *"Do you usually cough first thing in the morning?"* – but this is not used to filter people into the questions on coughing in winter).<br>○ *"Do you usually bring up any phlegm from your chest, first thing in the morning in the winter?"* (Again, this was asked to everyone in all years, but was preceded by an additional, non-winter-specific question in 2010).<br>○ Those saying 'yes' to each question were then asked, "*Do you [cough/bring up phlegm] like this on most days for as much as three months each year?"* In 2010 only, this was followed by the additional clarification '*That is, for three consecutive months'.*<br>People who reported three months/year of BOTH coughing first thing and of phlegm are considered to have possible symptoms of Chronic Obstructive Pulmonary Disease (COPD).
Diagnosed asthma	In 1995-7, 2001 and 2010, respondents were asked *"Did a doctor <1997 and 2010 only: or nurse> ever tell you that you had asthma?"* Whereas for other doctor-diagnosed conditions

For peer review only - http://bmjopen.bmj.com/site/about/guidelines.xhtml

	(heart problems/diabetes) we focus on those reporting problems in the past 12 months, it is not possible to construct a consistent measure of recent asthma, hence this variable refers to *lifetime* doctor-diagnosed asthma.
Asthma LSI	Every year 1994-2011, people who report a longstanding illness (LSI) are then asked, *'what is the matter with you?'*; up to 6 responses are then coded by the interviewer into a consistent coding frame based on the International Classification of Diseases.<br>The asthma LSI measure is based on the group labelled *'Asthma'*, which as of 2011 includes: Asthma; Bronchial asthma, allergic asthma; and Asthma - allergy to house dust/grass/cat fur. It explicitly <u>excludes</u> cardiac asthma.
Shortness of breath *(Grade 2+ / Grade 3)*	Respondents in 1995, 1996 and 2010 were asked the following questions about shortness of breath ('dyspnoea'):<br>○ *"Are you troubled by shortness of breath when hurrying on level ground or walking up a slight hill? Yes | No | Never walks up hill or hurries | Cannot walk"*<br>○ Those responding 'yes' or 'never walks up hill or hurries' are then asked, *"Do you get short of breath walking with other people of (your/his/her) own age on level ground? Yes | No | Never walks with people of own age on level ground"*.<br>○ Those responding 'yes' or 'never walks with people of own age' are then asked, *"Do you have to stop for breath after walking at (your/his/her) own pace on level ground?"*<br>This has been combined into the longstanding MRC dyspnoea scale [15] as follows:<br>- Grade 2 dyspnoea: people who report shortness of breath when hurrying on level ground or walking up a slight hill (or who report shortness of breath when walking on level ground, but who say they never walk up hill or hurry).<br>- Grade 3 dyspnoea: people who report shortness of breath when walking with people of own age on level ground, or who have to stop for breath when walking at own pace on level ground.<br>(The same questions also exist in 1994 and 1998, but (i) the wider bank of questions differs substantially in the two versions and question context effects are likely; and (ii) the filtering into the final question differs between versions. However, the 1991-98 trends are included below).
Recent wheezing/ asthma symptoms	Respondents in 1995-97, 2001 and 2010 were asked the following two questions as part of the battery of questions on breathing problems:<br>- *"I am now going to ask you some questions about your breathing... Have you ever had wheezing or whistling in the chest at any time, either now, or in the past?"*<br>- Those that said yes were then asked, *"Have you had wheezing or whistling in the chest in the last 12 months?"*<br>- (For those who said they had ever been told by a doctor they had asthma; see above), "*When was your most recent attack of asthma? PROMPT IF NECESSARY: Less than 4 weeks ago | More than 4 weeks but within the last 12 months | One to five years ago | More than 5 years ago"*<br>People who said they had EITHER wheezing/whistling in the past 12 months or an asthma attack in the past 12 months were counted as having recent wheezing/asthma symptoms.<br>[It should be noted that the filtering to the second question is very slightly different in 2010 compared to previous years (it was only asked to people who said they had not had wheezing/whistling in the chest in the past 12 months). However, given the way that the derived variable is calculated here, the change in filtering does not introduce any discontinuities over time].
Wheezing stopping sleep	Respondents in 1995-97, 2001 and 2010 were asked the following two questions as part of the battery of questions on breathing problems:<br>- *"I am now going to ask you some questions about your breathing... Have you ever had wheezing or whistling in the chest at any time, either now, or in the past?"*<br>- Those that said yes were then asked, *"Have you had wheezing or whistling in the chest in the last 12 months?"*<br>- Those that said yes were then asked, *"In the last 12 months, how often on average*

For peer review only - http://bmjopen.bmj.com/site/about/guidelines.xhtml

	has your sleep been disturbed due to wheezing or whistling in the chest?: Have you: Never woken with wheezing | Woken less than one night per week, or | Woken one or more nights per week?"<br>People were considered to have wheezing during sleep if they reported this at least once per week.
Anthropometric & diabetes	
BMI<br>*(Underweight / Obese)*	During the initial face-to-face interview in all years (except 2013), respondents were asked if they would consent to having their height and weight measured by the interviewer. The reasons for missingness (and their trends over time) are given in Web Appendices 2 & 3; note that there are three changes that give rise to small discontinuities in 2009 and 2011.<br>Obesity is a risk factor for diabetes (hence its inclusion in this section) but also heart disease and some cancers. Obesity is defined as a Body Mass Index (BMI) of >= $30kg/m^2$ as per the World Health Organization's BMI classification [16]. Using the same definition, underweight is defined as <=18.5 kg/m^2.
High waist-hip ratio	During the nurse visit in most years (excluding 1995-96, 2002, 2004 and 2013), respondents had their waist and hip circumferences measured. While BMI is a standard measurement of obesity, some evidence suggests that fat around the waist – 'central adiposity' – is a greater risk to health than fat elsewhere [17]. We use NICE's suggested 2006 thresholds for a high waist-hip ratio of >1 for men and >0.85 for women [18], as used in Hotchkiss et al [19].
Recent diabetes	Respondents in 1994, 1998, 2003, 2006 and 2009-2014 were asked a series of questions on whether they have diabetes:<br>- *"Do you now have, or have you ever had diabetes?"*<br>- Those responding 'yes' were then asked *"Were you told by a doctor that you had diabetes?"*<br>- Women responding 'yes' were then asked, *"Can I just check, were you pregnant when you were told that you had diabetes?"*, and those responding 'yes' were then asked *"Have you ever had diabetes apart from when you were pregnant?"*<br>- Finally, those with doctor-diagnosed diabetes (excluding only when pregnant were asked: *"Do you currently inject insulin for diabetes?"* and *"Are you currently taking any medicines, tablets or pills (other than insulin injections) for diabetes?"*<br>People were considered to have recent diabetes if they said they had ever been diagnosed as having diabetes by a doctor (excluding when pregnant), and that they are injecting insulin or taking any other medicines for diabetes.
Diabetes LSI	Every year 1994-2011, people who report a longstanding illness (LSI) are then asked, *'what is the matter with you?'*; up to 6 responses are then coded by the interviewer into a consistent coding frame based on the International Classification of Diseases.<br>The diabetes LSI measure is based on the group labelled *'Diabetes'*, which as of 2011 includes Diabetes and Hyperglycaemia.
High glycated haemoglobin	In the years 2003, 2006, and 2008-14, blood samples were obtained during the nurse visit, which were then analysed for glycated haemoglobin (HbA_{1C}). HbA_{1C} is a measure of the share of haemoglobin (within red blood cells) that glucose is attached to, with higher levels indicated less well-controlled diabetes in the previous three months [20]. Following the recommendations of a 2009 expert committee, we mirror recent HSE reports in using a threshold of 48mmol/mol (i.e. 48 millimoles of glycated haemoglobin per mole of haemoglobin) as the threshold for raised HbA_{1C}, a different threshold to that used in earlier HSE reports.<br>While the measurement of HbA_{1C} has been consistent in HSE from 1994, the units reported have changed from the % of haemoglobin that is glycated to mmol/mol. Earlier measures have been transformed into mmol/mol through the formula, mmol/mol = (% - 2.15) x 10.929. HbA_{1C} was also measured in 1994 but using a different technique, which cannot be made comparable [21:67].
Other biomarkers	

For peer review only - http://bmjopen.bmj.com/site/about/guidelines.xhtml

Raised C-reactive protein	In the years 1998, 2003, 2006, and 2009, blood samples were obtained during the nurse visit, which were then analysed for C-reactive protein (CRP). CRP is an inflammatory marker, which can indicate heart-related inflammation (it is used to test for heart failure) but can also indicate other sorts of health damage including diabetes. However, there are still debates about exactly what CRP shows, both in terms of its causal role in heart disease, and whether it also indicates depression.[22]<br>Raised CRP is defined as >3mg/L, the standard cut-off for a clinically significant rise in CVD [23 24]. Participants with CRP >10mg/L are excluded, as this is taken to be evidence of current infection rather than inflammation from chronic disease.
Raised Fibrinogen	In the years 1998, 2003, 2006, and 2009, blood samples were obtained during the nurse visit, which were then analysed for fibrinogen. Like CRP, fibrinogen is an inflammatory marker, which is both commonly thought to be a causal risk factor for CVD (it is a component of coagulation), and which seems to be a risk factor for other diseases (including cancer and diabetes)[25].<br>While fibrinogen is often analysed as a continuous variable with no cutpoints [24], we here define raised fibrinogen as>4mg/L as in [12]. As for CRP, participants with CRP >10mg/L are excluded, as this is taken to be evidence of current infection rather than inflammation from chronic disease. A change of analysis method and laboratory between 1994 and 1998 means that the 1994 results are not comparable to the later results [26:8.10.4].
Anaemia	In the years 1994, 1998, 2006, and 2009, blood samples were obtained during the nurse visit, which were then analysed for haemoglobin. Haemoglobin dist ributes oxygen around the body, and low haemoglobin levels usually indicate anaemia. Various different thresholds for low haemoglobin have been used in the literature, particularly for older populations [27], but we here used the longstanding WHO definition of <13g/dL for men and <12g/dL for women [24].
Iron deficiency	In the years 1994, 1998, 2006, and 2009, blood samples were obtained during the nurse visit, which were then analysed for serum ferritin (which correlates directly with the amount of iron stored in the body). Iron deficiency is one of several possible causes of anaemia (alongside other nutritional deficiencies, genetic conditions such as sickle cell anaemia, infections, and blood loss). Iron deficiency is defined as a serum ferritin less than 45ng/ml [27].
Mental health	
Mental health LSI	Every year 1994-2011, people who report a longstanding illness (LSI) are then asked, *'what is the matter with you?'*; up to 6 responses are then coded by the interviewer into a consistent coding frame based on the International Classification of Diseases.<br>The mental health LSI measure is based on the group labelled *'Mental illness/anxiety/depression/nerves (nes)'*, which as of 2011 includes: Alcoholism, recovered not cured alcoholic; Angelman Syndrome; Anorexia nervosa; Anxiety, panic attacks; Asperger Syndrome; Autism/Autistic (BBG: changed from 'autistic child'); Bipolar Affective Disorder; Catalepsy; Concussion syndrome; Depression; Drug addict; Dyslexia; Hyperactive child.; Nerves (nes); Nervous breakdown, neurasthenia, nervous trouble; Phobias; Schizophrenia, manic depressive; Senile dementia, forgetfulness, gets confused; Speech impediment, stammer; and Stress. It explicitly excludes Alzheimer's disease, degenerative brain disease.<br>While the LSI coding frame generally stays consistent over this period, it is worth being aware of a minor wording change within 'mental health LSI': the condition labelled 'Autistic child' 1994-1997 was relabelled 'Autism/Autistic' in 1998.
Psychological distress (GHQ)	In the self-completion survey in most years (except 1996, 2007, 2011 and 2013), respondents were asked the following series of questions:<br>- *"Please read this carefully: We should like to know how your health has been in general over the past few weeks. Please answer ALL the questions by ticking the box below the answer which you think most applies to you. Have you recently...*<br>- *"…been able to concentrate on whatever you're doing?" RESPONSES: "Better than usual" | "Same as usual" | "Less than usual" | "Much less than usual"*<br>- *"…lost much sleep over worry?" RESPONSES: "Not at all" | "No more than usual" | "Rather more than usual" | "Much more than usual""*

Web Appendix page 20

For peer review only - http://bmjopen.bmj.com/site/about/guidelines.xhtml

	- *"…felt you were playing a useful part in things?"* RESPONSES: *"More so than usual" | "Same as usual" | "Less useful than usual" | "Much less useful""*<br>- *"…felt capable of making decisions about things?"* RESPONSES: *"More so than usual" | "Same as usual" | "Less so than usual" | "Much less capable""*<br>- *"…felt constantly under strain?* RESPONSES: *"Not at all" | "No more than usual" | "Rather more than usual" | "Much more than usual""*<br>- *"..felt you couldn't overcome your difficulties?"* RESPONSES: *"Not at all" | "No more than usual" | "Rather more than usual" | "Much more than usual""*<br>- *"…been able to enjoy your normal day-to-day activities?"* RESPONSES: *"More so than usual" | "Same as usual" | "Less so than usual" | "Much less than usual"*<br>- *"…been able to face up to your problems?"* RESPONSES: *"More so than usual" | "Same as usual" | "Less able than usual" | "Much less able"*<br>- *"…been feeling unhappy and depressed?* RESPONSES: *"Not at all" | "No more than usual" | "Rather more than usual" | "Much more than usual"*<br>- *"…been losing confidence in yourself?* RESPONSES: *"Not at all" | "No more than usual" | "Rather more than usual" | "Much more than usual"*<br>- *"…been thinking of yourself as a worthless person?"* RESPONSES: *"Not at all" | "No more than usual" | "Rather more than usual" | "Much more than usual""*<br>- *"…been feeling reasonably happy, all things considered?"* RESPONSES: *"More so than usual" | "Same as usual" | "Less so than usual" | "Much less happy"*<br>These make up the 12-item General Health Questionnaire GHQ-12; [28], a well-validated, widely-used measure of probable mental ill-health. This is often termed general non-psychotic psychiatric morbidity, but I here use the more easily understood term 'psychological distress' following Stochl et al 2016.[29]<br>A total score has been created by first ensuring that all questions were coded from 1 (positive symptom) to 4 (negative symptom), and then creating a sum score for all the number of questions in which people answered with categories 3 or 4 (indicating a negative symptom). A binary measure (often called GHQ caseness) was created for people who had negative symptoms for 4 or more of the 12 questions.
Anxiety/depression *(moderately / Extremely)*	In the self-completion survey in 1996, 2003-6, 2008, 2010-12 and 2014, respondents were asked *'Now we would like to know how your health is today. Please answer ALL the questions. By ticking one box for each question below, please indicate which statements best describe your own health state today'*:<br>- *"I am not anxious or depressed"*<br>- *"I am moderately anxious or depressed"*<br>- *"I am extremely anxious or depressed"*<br>[This is part of the widely-used EQ-5D health status indicator [8]. However, for the purposes of this paper we have separated the individual measures that make up the EQ-5D in order to compare these to similar indicators of morbidity within each domain].<br>Two outcome measures are based on this: whether people have any anxiety/depression (the 2nd and 3rd categories combined), and whether they have extreme anxiety/depression (3rd category only).
Communication	
Hearing, seeing & communication limitations	These measures were not included in the main paper due to the short time frame that we can examine trends over, but are included in the Web Appendix as they relate to important domains of morbidity.<br>They were included in the disability scale used in the 2001 HSE report [9]. Respondents in 1995, 2000 and 2001 were asked if of the following applied to them (interviewers were instructed to ignore temporary disabilities that are expected to last less than one year):<br>• *"Cannot follow a TV programme at a volume others find acceptable (with hearing aid if normally worn)"* ('hearing limitation')<br>• *"Cannot see well enough to recognise a friend across a road (four yards away) (with glasses or contact lenses if normally worn)"* ('seeing limitation')<br>• *"Have problem communicating with other people - that is, have problem*

For peer review only - http://bmjopen.bmj.com/site/about/guidelines.xhtml

	understanding them or being understood by them" ('communication limitation')
Eye/Ear LSI	Every year 1994-2011, people who report a longstanding illness (LSI) are then asked, *'what is the matter with you?'*; up to 6 responses are then coded by the interviewer into a consistent coding frame based on the International Classification of Diseases. The Eye/Ear LSI includes the following groups:<br>• *Poor hearing/deafness*, including Conductive/nerve/noise induced deafness, Deaf mute/deaf and dumb, Heard of hearing, slightly deaf, Otosclerosis, Poor hearing after mastoid operation.<br>• *Tinnitus/noises in the ear*, Incl. pulsing in the ear<br>• *Other ear complaints*, Incl. otitis media - glue ear, Disorders of Eustachian tube, Perforated ear drum (nes), Middle/inner ear problems, Mastoiditis, Ear trouble (nes),, Ear problem (wax), Ear aches and discharges, Ear infection<br>• *Cataract/poor eye sight/blindness*, Incl. operation for cataracts, now need glasses, Bad eyesight, restricted vision, partially sighted, Bad eyesight/nearly blind because of cataracts, Blind in one eye, loss of one eye, Blindness caused by diabetes, Blurred vision, Detached/scarred retina, Hardening of lens, Lens implants in both eyes, Short sighted, long sighted, myopia, Trouble with eyes (nes), eyes not good (nes), Tunnel vision<br>• *Other eye complaints*, including Astigmatism, Buphthalmos, Colour blind, Double vision, Dry eye syndrome, trouble with tear ducts, watery eyes, Eye infection, conjunctivitis, Eyes are light sensitive, Floater in eye, Glaucoma, Haemorrhage behind eye, Injury to eye, Iritis, Keratoconus, Night blindness, Retinitis pigmentosa, Scarred cornea, corneal ulcers, Squint, lazy eye, Sty on eye.

Changes over time in several other measures are only presented in Web Appendices 4 & 6, rather than the main paper. Details of these variables are included below:

**Measure**	**Details**
General health	
General health<br>*(bad / good)*	Every year, respondents were asked, *"How is your health in general? Would you say it was ... very good, good, fair, bad, or very bad?"*<br>Two outcome measures are based on this, following standard practice in the HSE reports: bad general health (which includes 'bad' or 'very bad' health) and good general health (which includes 'good' or 'very good' health).
Longstanding illness (LSI)	Every year 1994-2011, respondents were asked *"Do you have any long-standing illness, disability or infirmity? By long-standing I mean anything that has troubled you over a period of time, or that is likely to affect you over a period of time?"* (The response options were 'Yes' and 'No').<br>In 2012 the question was changed to be consistent with the Government's new harmonised disability questions for use in social surveys [30], and is not comparable to the previous version.
Limiting LSI	Every year 1996-2011, respondents who said they had an LSI were than asked, *"Does this illness or disability (do any of these illnesses or disabilities) limit your activities in any way?"* (again allowing only Yes/No answers).<br>In 2012 the question was changed to be consistent with the Government's new harmonised disability questions for use in social surveys (see HSE 2012 report), and is not comparable to the previous version.
Problems with usual activities<br>*(some problems / unable)*	In the self-completion survey in 1996, 2003-6, 2008, 2010-12 and 2014, respondents were asked *'Now we would like to know how your health is today. Please answer ALL the questions. By ticking one box for each question below, please indicate which statements best describe your own health state today'*:<br>- *"I have no problems with performing my usual activities (e.g. work, study, housework,*

For peer review only - http://bmjopen.bmj.com/site/about/guidelines.xhtml

	family or leisure activities)"<br>- *"I have some problems with performing my usual activities"*<br>- *"I am unable to perform my usual activities"*<br>[This is part of the widely-used EQ-5D health status indicator [8]. However, for the purposes of this paper we have separated the individual measures that make up the EQ-5D in order to compare these to similar indicators of morbidity within each domain].<br>Two outcome measures are based on this: whether people have any problems (the 2nd and 3rd categories combined), and whether they are unable to perform their usual activities (3rd category only).
Limitations in past 2wks	Every year, respondents were asked, *"Now I'd like you to think about the two weeks ending yesterday. During those 2 weeks did you have to cut down on any of the things you usually do (about the house or at work or in your free time) because of your answer at <the LSI question> or some other illness or injury?"*<br>There have been two small changes to this question's wording in 1996. Firstly, 'work' was changed to 'work/school'. Secondly, *'your answer at <the LSI question>'* was changed to *'a condition you have just told me about'*. While it is impossible to be sure of the exact effect of these changes, neither seem likely to influence the results (at least for the 25+ age group where fewer individuals are in full-time education).

For peer review only

For peer review only - http://bmjopen.bmj.com/site/about/guidelines.xhtml

Appendix 6: Measures not included in the main paper

Trends in several measures are not included in the main paper, either

Table 10: Changes over time in measures not included in the main paper

	Starting period		Change from start to end period			
	Period	Prevalence	End period	Raw change	Adj.[a] change	Adj. change 95% CI
CVD						
Component measures nec[b]						
Recent heart murmur	1994-96	0.8%	2011-14	0.1%	0.0%	[-0.3, 0.4%]
Recent irregular heart rhythm	1994-96	1.6%	2011-14	0.4%	0.4%	[-0.1, 0.8%]
Recent other heart disease	1994-96	0.2%	2011-14	0.7%	0.7%	[0.4, 0.9%]
Ever had (not just recent)						
Ever had high BP	1994-96	19.0%	2011-14	4.5%	3.7%	[2.3, 5.1%]
DD high BP	1994-96	13.2%	2011-14	6.9%	6.0%	[4.7, 7.3%]
Ever IHD or stroke	1994-96	2.9%	2011-14	0.3%	-0.0%	[-0.6, 0.6%]
DD IHD or stroke	1994-96	2.5%	2011-14	0.5%	0.2%	[-0.3, 0.7%]
Ever had angina	1994-96	1.9%	2011-14	-0.2%	-0.4%	[-0.9, 0.0%]
Ever DD angina	1994-96	1.6%	2011-14	-0.1%	-0.3%	[-0.7, 0.1%]
Ever heart murmur	1994-96	3.2%	2011-14	-0.3%	-0.3%	[-0.9, 0.3%]
DD heart murmur	1994-96	2.6%	2011-14	-0.2%	-0.2%	[-0.7, 0.3%]
Ever irregular heart rhythm	1994-96	6.4%	2011-14	-0.7%	-0.9%	[-1.7, -0.1%]
DD irregular heart rhythm	1994-96	3.5%	2011-14	0.5%	0.3%	[-0.3, 1.0%]
Ever other heart disease	1994-96	0.9%	2011-14	1.1%	1.0%	[0.6, 1.5%]
DD other heart disease	1994-96	0.8%	2011-14	1.0%	1.0%	[0.6, 1.4%]
Respiratory						
**Alternate measures**						
Phlegm symptoms	1994-96	9.1%	2008-10	-1.3%	-1.4%	[-2.3, -0.5%]
LSI Respiratory All	1994-96	7.9%	2011-14	-0.7%	-0.7%	[-1.6, 0.1%]
**Ever had (not just recent)**						
Wheezing Ever	1994-96	32.3%	2008-10	0.0%	-0.1%	[-1.8, 1.5%]
Wheezing Past 12mths	1994-96	18.9%	2008-10	-1.0%	-1.1%	[-2.3, 0.2%]
Diabetes						
**Ever had (not just recent)**						
Ever diabetes	1994-96	2.0%	2011-14	2.9%	2.8%	[2.3, 3.2%]
DD diabetes	1994-96	1.7%	2011-14	2.5%	2.3%	[2.0, 2.7%]
Mental health						
**Alternate measures**						
High psychological distress	1994-96	3.2%	2011-14	1.0%	0.9%	[0.4, 1.4%]
Activity limitations & musculoskeletal						
**For comparison**						
Walking limitation	1994-96	4.6%	2001-03	1.4%	1.2%	[0.5, 1.9%]
Washing/dressing limitation	1994-96	1.9%	2001-03	0.5%	0.4%	[0.0, 0.8%]
Other LSIs						

For peer review only - http://bmjopen.bmj.com/site/about/guidelines.xhtml

	Starting period		Change from start to end period			
	Period	Prevalence	End period	Raw change	Adj.[a] change	Adj. change 95% CI
LSI Blood Disorders	1994-96	0.3%	2011-14	0.6%	0.5%	[0.3, 0.8%]
LSI Cancer	1994-96	1.0%	2011-14	0.3%	0.3%	[-0.1, 0.6%]
LSI D,GUM,E&M	1994-96	6.9%	2011-14	1.1%	0.8%	[0.0, 1.6%]
LSI Epilepsy	1994-96	0.7%	2011-14	0.1%	0.1%	[-0.2, 0.3%]
LSI Nervous System	1994-96	3.7%	2011-14	-0.2%	-0.3%	[-0.8, 0.3%]

[a] 'Adj.' = trend adjusted for changing age and sex distribution of the working-age population. [b] 'nec' = not elsewhere included.

The details of these measures are as follows:

Measure	Details
Circulatory	
Beyond 'recent': 'Ever had' and 'DD' CVD	In the main paper, we look at whether people report recent doctor-diagnosed CVD (looking separately at heart attack/stroke, angina, and any recent CVD). As shown above, this comes from three questions: whether people report ever having this condition; whether a doctor diagnosed this; and whether they have had an attack in the past 12 months / consider themselves to still have the condition.<br>Web Appendix 6 shows trends in the other versions of these measures, i.e. having ever had this type of CVD, and having ever doctor-diagnosed ('DD') CVD of this type.
Component measure: Heart murmur Irregular heart rhythm Other heart disease	In the main paper, we recent reports of doctor-diagnosed angina; heart attack (including myocardial infarction or coronary thrombosis); a heart murmur; abnormal heart rhythm; or other heart trouble (see above). Angina and heart attack are also analysed in the main paper in their own right; in Web Appendix 6, we further show trends separately in heart murmur, abnormal heart rhythm or other heart trouble.
Respiratory	
Component measure: 'phlegm'	In the main paper, we look at whether people report recent COPD (see above). This combines two measures: regular cough + phlegm. Web Appendix 6 shows the trend in the phlegm measure on its own, without being combined with a regular cough.
Alternative version: 'LSI respiratory'	In the main paper, we look at whether an asthma LSI (to examine alongside a direct question on diagnosed asthma); see above. Web Appendix 6 also shows people reporting a longstanding illness ('LSI') which is included within the broader category of respiratory conditions.<br>The respiratory LSI measure is based on the group labelled *'Asthma'*, *'Bronchitis'*, *'Hayfever'*, or *'Respiratory other'*, which as of 2011 includes:<br>Asthma: Asthma; Bronchial asthma, allergic asthma; and Asthma - allergy to house dust/grass/cat fur. It explicitly <u>excludes</u> cardiac asthma.<br>Hayfever: Hayfever, Allergic rhinitis<br>Bronchitis/emphysema: Bronchitis/emphysema, Bronchiectasis, Chronic bronchitis.<br>Other respiratory complaints: Other respiratory complaints, Abscess on larynx, Adenoid problems, nasal polyps, Allergy to dust/cat fur, Bad chest (nes), weak chest – wheezy, Breathlessness, Bronchial trouble, chest trouble (nes), Catarrh, Chest infections, get a lot of colds, Churg-Strauss syndrome, Chronic Obstructive Pulmonary Disease (COPD), Coughing fits, Croup, Damaged lung (nes), lost lower lobe of left lung, Fibrosis of lung, Furred up airways, collapsed lung, Lung complaint (nes), lung problems (nes), Lung damage by viral pneumonia, Paralysis of vocal cords, Pigeon fancier's lung, Pneumoconiosis, byssinosis, asbestosis and other industrial respiratory disease, Recurrent pleurisy, Rhinitis (nes), Sinus trouble, sinusitis, Sore throat, pharyngitis, Throat

For peer review only

For peer review only - http://bmjopen.bmj.com/site/about/guidelines.xhtml

Measure	Details
	infection, Throat trouble (nes), throat irritation, Tonsillitis, Ulcer on lung, fluid on lung. Note that:<br>• It explicitly excludes TB (pulmonary tuberculosis), Cystic fibrosis, Skin allergy, Food allergy, Allergy (nes), Pilonidal sinus, Sick sinus syndrome, Whooping cough.<br>• If complaint is breathlessness with the cause also stated, this is coded with the cause – hence it also excludes breathlessness as a result of anaemia, breathlessness due to hole in heart, and breathlessness due to angina.
Component measure: Wheezing	In the main paper, we look at whether people report recent wheezing/asthma. As shown above, this comes from three questions: whether people report ever having had wheezing or whistling in the chest; whether they have had this in the past 12 months; and whether they have had an asthma attack in the past 12 months.<br>Web Appendix 6 shows trends in the other versions of these measures, i.e. having ever had wheezing/whistling in the chest, and whether they have had this in the past 1 months.
Beyond 'recent': 'Ever had' and 'DD' diabetes	In the main paper, we look at whether people report recent doctor-diagnosed diabetes As shown above, this comes from three questions: whether people report ever having this condition; whether a doctor diagnosed this; and whether they currently inject insulin / take other medication for diabetes.<br>Web Appendix 6 shows trends in the other versions of these measures, i.e. having ever had diabetes, and having ever doctor-diagnosed ('DD') diabetes.
Activity limitations	
For comparison: Walking limitation	This is based on the personal care disability scale used in the 2001 HSE report [9]. Respondents in 1995, 2000 and 2001 were asked if of the following applied to them (interviewers were instructed to ignore temporary disabilities that are expected to last less than one year): *"Cannot walk 200 yards or more on own without stopping or discomfort"*. People who reported a limitation were asked if they used a walking aid, and if they did, were then asked if they could walk 200 yards without the walking aid.
For comparison: Washing & dressing limitation	This is based on the personal care disability scale used in the 2001 HSE report [9]. Respondents in 1995, 2000 and 2001 were asked if any of the following applied to them (interviewers were instructed to ignore temporary disabilities that are expected to last less than one year):<br>- *"Cannot dress and undress without difficulty"*<br>- *"Cannot wash hands and face without difficulty"*<br>For comparison to the 'problems with washing/dressing today' measure in the main paper (which covers a more extended period and is based on a different question; see above), a measure is derived if respondents say they report either of these problems.
Other LSIs	
Other LSIs	Every year 1994-2011, people who report a longstanding illness (LSI) are then asked, *'what is the matter with you?'*; up to 6 responses are then coded by the interviewer into a consistent coding frame based on the International Classification of Diseases. The various other LSIs are as follows:<br>• The Blood Disorders LSI measure is based on the group *'Disorders of blood and blood forming organs and immunity disorders'*, which as of 2011 includes: Anaemia, pernicious anaemia, Blood condition (nes), blood deficiency, Haemophilia, Idiopathic Thrombochopenic Purpura (ITP), Immunodeficiences, Polycthaemia (blood thickening), blood to thick, Purpura (nes), Removal of spleen, Sarcoidosis (previously code 37), Sickle cell anaemia/disease, Thalassaemia, Thrombocythenia. It explicitly excludes Leukaemia - code 01.<br>• The Cancer LSI measure is based on the group *'Cancer (neoplasm) including lumps, masses, tumours and growths and benign (non-malignant) lumps and cysts'*, which as of

For peer review only - http://bmjopen.bmj.com/site/about/guidelines.xhtml

Measure	Details
	2011 includes: Acoustic neuroma, After effect of cancer (nes), All tumours, growths, masses, lumps and cysts, whether malignant or benign eg. tumour on brain,, growth in bowel, growth on spinal cord, lump in, breast, Cancers sited in any part of the body or system eg., Lung, breast, stomach, Colostomy caused by cancer, Cyst on eye, cyst in kidney., General arthroma, Hereditary cancer, Hodgkin's disease, Hysterectomy for cancer of womb, Inch. leukaemia (cancer of the blood), Lymphoma, Mastectomy (nes), Neurofibromatosis, Part of intestines removed (cancer), Pituitary gland removed (cancer), Rodent ulcers, Sarcomas, carcinomas, Skin cancer, bone cancer, Wilms tumour<br>• The D,GUM,E&M (Digestive, Genitourinary Medicine, and Endocrine & Metabolic) LSI is based on the groups, *'Complaints of bowel/colon (large intestine,caecum, bowel, colon, rectum)'* (including Colitis, colon trouble, ulcerative colitis, Coleliac, Colostomy (nes), Crohn's disease, Diverticulitis, Enteritis, Faecal incontinence/encopresis., Frequent diarrhoea, constipation, Grumbling appendix, Hirschsprung's disease, Irritable bowel, inflammation of bowel, Polyp on bowel, Spastic colon, but explicitly excluding piles and Cancer of stomach/bowel), *Other digestive complaints (stomach, liver, pancreas, bile ducts, small intestine - duodenum, jejunum and ileum)* (including Cirrhosis of the liver, liver problems, Food allergies, Ileostomy, Indigestion, heart burn, dyspepsia, Inflamed duodenum, Liver disease, biliary artesia, Nervous stomach, acid stomach, Pancreas problems, Stomach trouble (nes), abdominal trouble (nes), Stone in gallbladder, gallbladder problems, Throat trouble - difficulty in swallowing, Weakness in intestines), *Stomach ulcer/ulcer (nes)/abdominal hernia/rupture* (including Double/inguinal/diaphragm/hiatus/umbilical hernia, Gastric/duodenal/peptic ulcer, Hernia (nes), rupture (nes), Ulcer (nes)), *Complaints of teeth/mouth/tongue* (including Cleft palate, hare lip, Impacted wisdom tooth, gingivitis, No sense of taste, Ulcers on tongue, mouth ulcers), *Other endocrine/metabolic* (including Addison's disease, Beckwith - Wiedemann syndrome, Coeliac disease, Cushing's syndrome, Cystic fibrosis, Gilbert's syndrome, Hormone deficiency, deficiency of growth hormone,, dwarfism, Hypercalcemia, Hypopotassaemia, lack of potassium, Malacia, Myxoedema (nes), Obesity/overweight, Phenylketonuria, Rickets, Too much cholesterol in blood, Underactive/overactive thyroid, goitre, Water/fluid retention, Wilson's disease, but explicitly excluding Thyroid trouble and tiredness and Overactive thyroid and swelling in neck, *Other bladder problems/incontinence* (including Bed wetting, enuresis, Bladder restriction, Water trouble (nes), Weak bladder, bladder complaint (nes), but explicitly excluding Prostate trouble), *Kidney complaints* (including Chronic renal failure, Horseshoe kidney, cystic kidney, Kidney trouble, tube damage, stone in the kidney, Nephritis, pyelonephritis, Nephrotic syndrome, Only one kidney, double kidney on right side, Renal TB, Uraemia), *Reproductive system disorders* (including Abscess on breast, mastitis, cracked nipple, Amenorrhea, Damaged testicles, Endometriosis, Gynaecological problems, Hysterectomy (nes), Impotence, infertility, Menopause, Pelvic inflammatory disease/PID (female), Period problems, flooding, pre-menstrual tension/syndrome, Prolapse (nes) if female, Prolapsed womb, Prostrate gland trouble, Turner's syndrome, Vaginitis, vulvitis, dysmenorrhoea) and *Urinary tract infection* (including Cystitis, urine infection).<br>• The Epilepsy LSI is based on the group, *'Epilepsy/fits/convulsion',* including Grand mal, Petit mal, Jacksonian fit, Lennox-Gastaut syndrome, blackouts, febrile convulsions, fit (nes)<br>• The Nervous System LSI is based on the groups:<br>o *Migraine/headaches*<br>o *Other problems of nervous system*, including Abscess on brain, Alzheimer's

For peer review only - http://bmjopen.bmj.com/site/about/guidelines.xhtml

Measure	Details	
		disease, Bell's palsy, Brain damage resulting from infection (eg. meningitis,, encephalitis) or injury, Carpal tunnel syndrome, Cerebral palsy (spastic), Degenerative brain disease, Fibromyalgia, Friedreich's Ataxia, Guillain-Barre syndrome, Huntington's chorea, Hydrocephalus, microcephaly, fluid on brain, Injury to spine resulting in paralysis, Metachromatic leucodystrophy, Motor neurone disease, Multiple Sclerosis (MS), disseminated sclerosis, Muscular dystrophy, Myalgic encephalomyelitis (ME), Myasthenia gravis, Myotonic dystrophy, Neuralgia, neuritis, Numbness/loss of feeling in fingers, hand, leg etc, Paraplegia (paralysis of lower limbs), Parkinson's disease (paralysis agitans), Partially paralysed (nes), Physically handicapped - spasticity of all limbs, Pins and needles in arm, Post viral syndrome (ME), Removal of nerve in arm, Restless legs, Sciatica, Shingles, Spina bifida, Syringomyelia, Trapped nerve, Trigeminal neuralgia, Teraplegia"
	○	*Meniere's disease/ear complaints causing balance problems* (including Labryrinthitis,, loss of balance - inner ear, Vertigo).

peer review only

For peer review only - http://bmjopen.bmj.com/site/about/guidelines.xhtml

Appendix 7: Year-by-year trends

This appendix presents the year-by-year trends for all of the variables included in the main paper. The table row labelled 'start v end sig' presents the p-value for testing the null hypothesis that there is no difference between the first and last years in the series (whichever these years are). Note that this will differ from the confidence intervals presented in the main paper as these are grouped into multi-year periods with larger sample sizes and therefore greater precision.

Table 11: Year-to-year trends in cardiovascular health

	High blood pressure LSI	Recent high blood pressure	*Biomarker high blood pressure*	Recent heart attack/stroke	IHD/stroke LSI	Heart attack symptoms	Mini stroke (TIA) symptoms	Recent angina	*Angina symptoms*
**1994**	2.2%	4.2%	8.4%	1.2%	1.4%	5.5%		1.1%	2.3%
**1995**	2.9%		8.3%		1.5%				
**1996**	3.0%		8.3%		1.5%				
**1997**	3.8%		7.7%		1.4%				
**1998**	3.1%	5.4%	7.0%	1.5%	1.3%	6.5%		1.4%	2.2%
**1999**	3.4%				1.4%				
**2000**	4.0%		6.5%		1.3%				
**2001**	4.5%		7.3%		1.7%				
**2002**	4.3%		6.1%		1.4%				
**2003**	4.5%	7.9%	4.9%	1.3%	1.3%	5.5%	8.1%	1.0%	1.8%
**2004**	4.0%				1.2%				
**2005**	5.0%		4.4%		1.3%				
**2006**	4.4%	8.7%	3.9%	1.1%	1.2%	6.2%	7.8%	0.9%	1.6%
**2007**	4.9%		4.5%		1.0%				
**2008**	5.1%		3.9%		1.1%				
**2009**	4.7%		3.2%		1.3%				
**2010**	4.6%		4.1%		1.1%				
**2011**	4.0%	9.5%	3.2%	1.0%	1.0%	5.2%	6.7%	0.7%	1.2%
**2012**			4.1%						
**2013**			3.7%						
**2014**			3.9%						
**Start v end sig.**	0.00	0.00	0.00	0.14	0.05	0.52	0.01	0.03	0.00
**N**	124,830	43,292	79,601	43,445	124,830	43,521	23,487	43,477	43,518

For peer review only - http://bmjopen.bmj.com/site/about/guidelines.xhtml

Table 12: Year-to-year trends in respiratory health

	COPD symptoms	Diagnosed asthma	Asthma LSI	Breathlessness-Grade 2+	Breathlessness-Grade 3	Recent wheezing/asthma	Wheezing stopping sleep
**1994**			4.7%				
**1995**	6.6%	10.8%	4.8%	19.1%	7.6%	19.8%	3.6%
**1996**	6.6%	11.5%	5.3%	20.3%	8.0%	19.3%	3.5%
**1997**		11.9%	6.0%			18.9%	3.7%
**1998**			5.3%				
**1999**			5.7%				
**2000**			5.5%				
**2001**		14.1%	5.9%			19.9%	3.4%
**2002**			6.0%				
**2003**			5.8%				
**2004**			6.3%				
**2005**			6.1%				
**2006**			5.8%				
**2007**			5.7%				
**2008**			6.2%				
**2009**			5.5%				
**2010**	5.1%	16.6%	6.0%	15.4%	6.4%	18.4%	3.2%
**2011**			5.6%				
**2012**							
**2013**							
**2014**							
**Start v end sig.**	0.00	0.00	0.02	0.00	0.01	0.05	0.18
**N**	25,631	41,219	124,830	25,620	25,620	41,218	41,218

For peer review only - http://bmjopen.bmj.com/site/about/guidelines.xhtml

Table 13: Year-to-year trends in activity limitations & musculoskeletal health

	Problems walking about today	Walking limitation	Any locomotor limitation	Problems washing/dressing today	Washing/dressing limitation	Any self-care limitation	Pain-any	Pain-extreme	Arthritis LSI	Other musculoskeletal LSI
**1994**									4.9%	8.9%
**1995**		4.6%	6.8%		1.9%	3.9%			5.4%	9.9%
**1996**	11.5%			3.4%			32.0%	3.0%	5.4%	10.3%
**1997**									6.0%	11.4%
**1998**									5.6%	11.7%
**1999**									5.5%	11.0%
**2000**		6.3%	8.2%		2.5%	5.2%			5.6%	10.7%
**2001**		5.9%	7.8%		2.4%	4.7%			6.1%	10.9%
**2002**									5.7%	12.3%
**2003**	11.8%			3.2%			27.1%	3.2%	6.2%	11.8%
**2004**	11.6%			3.6%			28.6%	3.5%	6.3%	11.6%
**2005**	12.3%			4.0%			27.8%	3.5%	6.0%	11.3%
**2006**	11.6%			3.6%			26.8%	3.1%	5.4%	10.1%
**2007**									5.4%	9.9%
**2008**	11.5%			3.6%			28.1%	3.1%	4.7%	9.5%
**2009**									5.2%	9.0%
**2010**	13.0%			4.1%			29.9%	3.2%	5.1%	10.3%
**2011**	13.6%			4.0%			34.0%	4.0%	4.9%	9.2%
**2012**	11.8%			3.8%			27.4%	3.1%		
**2013**										
**2014**	12.2%			4.2%			27.7%	3.0%		
**Start v end sig.**	0.30	0.00	0.01	0.05	0.04	0.01	0.00	0.89	0.97	0.57
**N**	62,680	25,341	25,341	62,612	25,341	25,341	62,692	62,692	124,830	124,830

For peer review only - http://bmjopen.bmj.com/site/about/guidelines.xhtml

Table 14: Year-to-year trends in obesity & diabetes

	BMI-Underweight	BMI-Obese	High waist-hip ratio	Recent diabetes	Diabetes LSI	Glycated haemoglobin
**1994**	1.1%	15.7%	9.5%	1.2%	1.5%	
**1995**	1.1%	17.0%			1.6%	
**1996**	0.9%	17.9%			1.6%	
**1997**	0.9%	19.3%	12.1%		1.7%	
**1998**	1.0%	19.5%	11.3%	1.4%	1.5%	
**1999**	1.1%	20.1%	16.3%		1.9%	
**2000**	0.9%	21.5%			2.0%	
**2001**	0.9%	22.8%	15.8%		2.1%	
**2002**	1.0%	23.5%	16.5%		2.1%	
**2003**	0.9%	23.2%	18.7%	2.1%	2.4%	2.7%
**2004**	1.0%	24.3%			2.8%	
**2005**	0.8%	24.5%	21.6%		2.9%	
**2006**	0.8%	25.1%	20.7%	2.7%	2.9%	3.1%
**2007**	1.0%	25.3%	22.1%		3.4%	
**2008**	0.9%	25.3%	22.5%		2.9%	3.8%
**2009**	1.4%	24.3%	23.5%	3.4%	3.8%	4.3%
**2010**	1.1%	27.8%	24.3%	3.4%	3.5%	3.7%
**2011**	0.8%	25.4%	24.3%	3.6%	3.8%	5.5%
**2012**	1.1%	25.6%	24.0%	3.6%		4.9%
**2013**	1.0%	26.8%	24.2%	3.6%		4.8%
**2014**	0.8%	27.1%	24.7%	3.7%		4.4%
**Start v end sig.**	1.1%	15.7%	9.5%	1.2%	1.5%	
**N**	1.1%	17.0%			1.6%	

Web Appendix page 32

Table 15: Year-to-year trends in other biomarkers

	High total cholesterol	Low HDL cholesterol	Raised C-reactive protein	Raised fibrinogen	Anaemia	Iron deficiency	Cancer LSI
**1994**	75.7%				6.7%	39.9%	0.2%
**1995**							0.3%
**1996**							0.3%
**1997**							0.4%
**1998**	64.8%	11.8%	21.4%	2.3%	6.3%	38.2%	0.5%
**1999**							0.4%
**2000**							0.5%
**2001**							0.5%
**2002**							0.5%
**2003**	71.4%	4.0%	24.1%	5.7%			0.6%
**2004**							0.6%
**2005**							0.6%
**2006**	67.2%	5.1%	22.7%	5.7%	4.6%	29.3%	0.7%
**2007**							0.5%
**2008**	66.7%	4.3%					0.6%
**2009**	66.9%	4.5%	23.5%	3.8%	5.3%	27.0%	0.5%
**2010**	64.1%	4.6%					0.8%
**2011**	60.2%	4.5%					0.8%
**2012**	64.0%	4.4%					
**2013**	58.0%	3.4%					
**2014**	55.4%	2.9%					
**Start v end sig.**	0.00	0.00	0.11	0.01	0.04	0.00	0.00
**N**	41,224	33,937	17,749	16,105	20,228	20,304	124,830

For peer review only - http://bmjopen.bmj.com/site/about/guidelines.xhtml

Table 16: Year-to-year trends in mental health

	Mental health LSI	Psychological distress symptoms	Anxiety/depression -moderately	Anxiety/depression -extremely
**1994**	1.8%	16.1%		
**1995**	2.3%	18.0%		
**1996**	2.4%		21.9%	1.8%
**1997**	2.9%	16.5%		
**1998**	3.0%	15.6%		
**1999**	3.0%	17.7%		
**2000**	3.5%	14.4%		
**2001**	3.3%	13.7%		
**2002**	3.1%	16.6%		
**2003**	3.7%	13.5%	18.5%	1.9%
**2004**	3.6%	13.4%	18.8%	2.1%
**2005**	4.4%	14.0%	19.6%	2.1%
**2006**	4.1%	13.9%	18.8%	2.1%
**2007**	4.5%			
**2008**	4.2%	13.7%	18.5%	2.0%
**2009**	4.9%	17.1%		
**2010**	5.2%	16.1%	23.5%	2.7%
**2011**	4.6%		26.8%	3.0%
**2012**		16.0%	20.0%	2.7%
**2013**				
**2014**		15.6%	19.6%	2.5%
**Start v end sig.**	0.00	0.47	0.01	0.02
**N**	124,830	107,834	62,635	62,635

For peer review only - http://bmjopen.bmj.com/site/about/guidelines.xhtml

Appendix 8: Others' analyses over change over time using HSE data

Changes over time in some of these indicators have not previously been analysed (e.g. waist-hip ratio, fibrinogen). However, others have been studied but never integrated into a single picture of changing morbidity; we review these in this section. (For reasons of space these are included here rather than in the main text).

Cardiovascular morbidity

1998-2011 trends in the two biomarkers for total and HDL cholesterol using HSE data are shown in Oyebode,[11] who find similar results.

Respiratory morbidity

A subset of the HSE respiratory indicators (ever/past year wheezing, doctor-diagnosed asthma) were analysed by Hall and Mindell[31] looking at 2001-2010, and finding similar changes over time to our analysis. They found stability in some measures (ever wheezing) but improvements in others (past-year wheezing) – at the same time as the reported prevalence of doctor-diagnosed asthma increased.

Obesity & diabetes

While the English trends in waist-hip ratio have not previously been analysed, earlier Scottish trends are given in Hotchkiss et al 2012.[19] Trends in diabetes have been covered in several HSE reports, e.g. Moody 2012,[20] as has BMI (see particularly the paper by Sperrin et al 2014,[32] who also created a publicly-available time-series HSE dataset for this purpose).

Activity limitations, pain & musculoskeletal morbidity

While musculoskeletal LSIs have not previously been analysed in HSE, a decline can also be seen in the General Household Survey.[33]

Mental health

In the UK and most other high-income countries, benefit claims due to mental ill-health have been rising,[34] which has come alongside considerable increases in mental health diagnosis and treatment.[35] The extent to which this reflects rises in mental ill-health and genuinely declining work capacity, however, has long been the subject of debate.[36 37] Perhaps the most robust long-term general population data series in the UK is the Adult Psychiatric Morbidity Survey.[35 38]

While some studies have used HSE to show rises in mental ill-health, others have used the same data to come to the opposite conclusion.[39 40] These contrasting conclusions are explained by the tables in Web Appendix 7 which show year-by-year changes: moderate mental ill-health fell between the mid-1990s and the mid-2000s, before rising in 2009, and with a particularly high prevalence in 2011. The conclusions of studies will therefore depend on the years they use as their start and end periods for the trend analysis.[3] It is also worth noting that our results for considerable increases in mental health LSIs can also be seen in a similar measure in the Labour Force Survey.[41 42]

[3] The major explanation why 'moderate anxiety/depression today' does not show a decline 2011-14 compared to 1994-6 is because of a single very high reported prevalence in 2011, which had reduced by 2012 and 2014. The alternate measure ('psychological distress symptoms') was not asked in 2011.

For peer review only - http://bmjopen.bmj.com/site/about/guidelines.xhtml

Other morbidity measures

While CRP and fibrinogen are collected in HSE at considerable efforts, their trends have rarely been studied (e.g. they appear only in supplementary descriptive tables in Hughes et al [23]). A decline in anaemia using HSE data 1998-2005 has been observed by Tull et al 2009,[43] but this has not hitherto been updated to the 2008-10 period.

It has been suggested that multimorbidity has risen among older people in England [44] and for all age groups in Ontario,[45] although others have cautioned against using simple disease counts,[46] and the evidence cited in the introduction of the main paper suggests that rising chronic disease reporting may partly be a result of increasing awareness (rather than underlying prevalence) of disease.

For peer review only

For peer review only - http://bmjopen.bmj.com/site/about/guidelines.xhtml

Appendix 9: Summarising multiple measures

Having reviewed trends in 39 morbidity measures, we have seen that morbidity in the English working-age population has improved in some respects and deteriorated in others. For those who view work-related morbidity as intrinsically multidimensional,[47], this is the endpoint of our analysis. However, for those who conceive of morbidity as unidimensional – or those who are interested in morbidity as it relates to a unidimensional work capacity – this raises the question of how we weight different dimensions of morbidity to decide if the overall change in morbidity has been positive or negative.

Methods for creating unidimensional morbidity scales

Several methods have been proposed for creating unidimensional morbidity scales, but most of these are unavailable using the HSE data:

- Weights can be based on empirically-derived preferences for different health states, of which the most famous example is the WHO Global Burden of Disease (GBD) study [48]. Some GBD estimates for trends in disability in the UK do exist, and suggest that the prevalence of disability in the working-age population is unchanged 1990-2010, though these results are only presented in passing.[4] For our analyses, however, we have no preference-based weights for most of the HSE measures (excluding the subset of measures that make up the EQ-5D scale).
- Those reporting limitations beyond a certain severity in any domain can be categorised as 'disabled', as recommended by the Washington Group on Disability Statistics (see above). However, as previously discussed, we have few functional limitations measures available in HSE.
- Latent morbidity scales can be created based on the inter-correlations between different measures (using item response theory), as used in the World Disability Report [51] and by researchers associated with the US National Bureau of Economic Research e.g. [52]. However, it is unclear why we would wish to weight items in this way: a given morbidity indicator may be severe, yet if it is unrelated to other morbidity measures it will be given a low weight.
- Latent morbidity scales can also be created based on the independent correlation between each indicator and a general measure of morbidity, such as general self-reported health or [53] as in [54]. This maintains some of the advantages of single-item measures (in providing a basis for making morbidity unidimensional), while avoiding the potential threats to validity discussed above. However, the inconsistent inclusion of measures in each HSE wave prevents a unidimensional morbidity scale being constructed here.

[4] Trends in the UK GBD results are reported in Murray et al.[49] However, Murray et al do not focus on trends in years lived with disability (YLD), other than to note that *"YLDs per person by age and sex have not changed substantially in the UK, but age-specific mortality has been improving"* (p1005). The figure in the supplementary appendix shows that YLDs have barely changed for either men or women at any age. However, the confidence intervals for YLDs as a whole in the main paper (Table 1) suggest that the confidence intervals for these trends are very wide. The public GBD data [50] do provide cause-disaggregated YLDs for the UK (and all other countries) for a slightly different period (2000-2015), but are not age-standardised, are within broad age groups only (e.g. 15-29), and again lack estimates of uncertainty.

For peer review only - http://bmjopen.bmj.com/site/about/guidelines.xhtml

An alternative way of summarising heterogeneous trends

Nevertheless, we can examine if the areas in which morbidity has been improving or declining are those that are particularly important for general health.[53] (This uses the same intuition as the scales in Diederichs et al 2012).[54] To see how important measures are for general health, we regress 'bad' general health (see Appendix 5 for detail on the underlying question) on age, sex (and their interaction), educational level and each individual morbidity measure in turn, using all years for which that morbidity measure is available. That is, for each morbidity indicator morbidity_i^a we use the following model:

$$\text{badhealth}_i = \text{logit}\,[\,\beta_1 \text{morbidity}_i^a + \boldsymbol{\beta_2 \text{age}_\text{i}} + \beta_3 male_i + \boldsymbol{\beta_4}(\mathbf{age_i} * \text{male}_\text{i}) + \ \boldsymbol{\beta_5 \text{education}_\text{i}}]$$

… where β_1 is our primary outcome coefficient showing the importance of that morbidity indicator for bad health, $\mathbf{age_i}$ refers to a vector of age dummy variables, male_i refers to a binary gender dummy variable, $\mathbf{education_i}$ refers to a vector of education dummy variables (with four levels: degree/full-time student, A-levels/NVQ3/higher education below degree, other qualifications, or no qualifications), and $\boldsymbol{\beta_2}$, β_3, $\boldsymbol{\beta_4}$, and $\boldsymbol{\beta_5}$ refer to the coefficients on age, gender, their interaction and education respectively.

We adjust for education as well as age & sex to enable us to examine the importance of the measure for bad health, after taking account of whether general health and the measure are both strongly related to social status. Note however that it is not possible to control for all morbidity measures simultaneously (as we discuss just above) – so this is a rough indicator of the importance of that morbidity measure for general health, rather than a reliable indicator of the causal impact net of co-morbidities.

The results of this analysis are shown overleaf, ordered by the effect on bad health. (We also repeat the trend in each measure for convenience; this is discussed following the table).

For peer review only

For peer review only - http://bmjopen.bmj.com/site/about/guidelines.xhtml

**Measure**	**Type**	**Effect on bad health** *(95% CI)*		**Change over time in measure** *(95% CI)*	
Pain-extreme	S	46.4%	*[44.0, 48.9%]*	0.2%	*[-0.3, 0.7%]*
Problems washing/dressing today	S	43.7%	*[41.4, 46.0%]*	0.3%	*[-0.2, 0.9%]*
Anxiety/depression-extremely	S	35.4%	*[32.8, 38.0%]*	0.9%	*[0.5, 1.3%]*
Any locomotor limitation	S	33.6%	*[31.2, 36.0%]*	0.9%	*[0.1, 1.7%]*
Any self-care limitation	S	32.6%	*[29.7, 35.5%]*	0.7%	*[0.1, 1.3%]*
Problems walking about today	S	26.3%	*[25.2, 27.4%]*	0.4%	*[-0.6, 1.3%]*
High psychological distress	S	26.4%	*[24.9, 27.9%]*	0.9%	*[0.4, 1.4%]*
Recent angina	L	23.8%	*[20.1, 27.5%]*	-0.5%	*[-0.8, -0.1%]*
Recent heart attack/stroke	L	23.2%	*[19.7, 26.7%]*	-0.4%	*[-0.7, 0.0%]*
Breathlessness-Grade 3	S	22.9%	*[20.9, 24.9%]*	-1.6%	*[-2.5, -0.8%]*
Mental health LSI	L	20.4%	*[19.1, 21.7%]*	2.4%	*[1.8, 3.0%]*
IHD/stroke LSI	L	19.7%	*[17.9, 21.5%]*	-0.6%	*[-0.9, -0.2%]*
Wheezing stopping sleep	S	19.1%	*[17.1, 21.1%]*	-0.5%	*[-1.0, 0.1%]*
Mini stroke (TIA) symptoms	S	16.8%	*[15.0, 18.6%]*	-1.4%	*[-2.4, -0.4%]*
Angina symptoms	S	16.6%	*[14.1, 19.1%]*	-1.2%	*[-1.6, -0.7%]*
Psychological distress symptoms	S	15.2%	*[14.6, 15.8%]*	-1.3%	*[-2.4, -0.3%]*
Arthritis LSI	L	15.2%	*[14.3, 16.1%]*	-0.7%	*[-1.4, 0.0%]*
Any recent CVD	L	14.4%	*[12.7, 16.1%]*	0.5%	*[-0.1, 1.2%]*
Heart attack symptoms	S	14.1%	*[12.6, 15.6%]*	-0.5%	*[-1.3, 0.3%]*
Anxiety/depression-moderately	S	13.6%	*[13.0, 14.2%]*	0.1%	*[-1.1, 1.3%]*
Pain-any	S	12.9%	*[12.4, 13.4%]*	-3.3%	*[-4.6, -2.0%]*
COPD symptoms	S	12.6%	*[11.0, 14.2%]*	-1.6%	*[-2.3, -0.8%]*
Diabetes LSI	L	12.4%	*[11.1, 13.7%]*	2.1%	*[1.5, 2.6%]*
Recent diabetes	L	11.8%	*[10.2, 13.4%]*	2.2%	*[1.9, 2.6%]*
Breathlessness-Grade 2+	S	11.5%	*[10.5, 12.5%]*	-4.8%	*[-6.1, -3.5%]*
Any CVD LSI	L	11.0%	*[10.3, 11.7%]*	0.6%	*[-0.1, 1.4%]*
Other musculoskeletal LSI	L	9.8%	*[9.2, 10.4%]*	-0.8%	*[-1.7, 0.1%]*
Glycated haemoglobin	B	9.9%	*[7.9, 11.9%]*	2.1%	*[1.4, 2.7%]*
Asthma LSI	L	8.6%	*[7.8, 9.4%]*	0.7%	*[0.0, 1.4%]*
Recent wheezing/asthma	S	8.4%	*[7.7, 9.1%]*	-1.2%	*[-2.5, 0.1%]*
Recent high blood pressure	L	6.8%	*[5.7, 7.9%]*	4.8%	*[3.9, 5.6%]*
BMI-Underweight	B	6.2%	*[4.3, 8.1%]*	-0.1%	*[-0.3, 0.1%]*
Diagnosed asthma	L	5.9%	*[5.1, 6.7%]*	5.7%	*[4.5, 6.8%]*
High waist-hip ratio	B	4.6%	*[4.1, 5.1%]*	14.1%	*[13.0, 15.2%]*
Raised fibrinogen	B	4.3%	*[1.9, 6.7%]*	1.5%	*[0.3, 2.6%]*
Low HDL cholesterol	B	4.3%	*[2.8, 5.8%]*	-8.0%	*[-9.0, -7.1%]*
Raised C-reactive protein	B	3.7%	*[2.7, 4.7%]*	1.9%	*[-0.7, 4.5%]*
BMI-Obese	B	2.8%	*[2.5, 3.1%]*	8.9%	*[8.0, 9.7%]*
Anaemia	B	2.4%	*[0.8, 4.0%]*	-1.4%	*[-2.7, -0.1%]*
Biomarker high blood pressure	B	0.4%	*[-0.3, 1.1%]*	-5.0%	*[-5.6, -4.5%]*
High total cholesterol	B	0.0%	*[-0.6, 0.6%]*	-17.6%	*[-19.1, -16.1%]*
Iron deficiency	B	-0.5%	*[-1.3, 0.3%]*	-12.5%	*[-14.8, -10.2%]*

For peer review only - http://bmjopen.bmj.com/site/about/guidelines.xhtml

Having estimated this, we can see if the areas in which morbidity has been improving or declining are those that are particularly important for general health. This is shown visually in Figure 1 below (the measures are not labelled to enable the overall pattern to be seen, but the top-to-bottom order of measures is the same in the figure as in the preceding table; i.e. the measure at the top of the figure is 'Pain-extreme').

Figure 1: Change over time in morbidity measures & their association with bad general health[a]

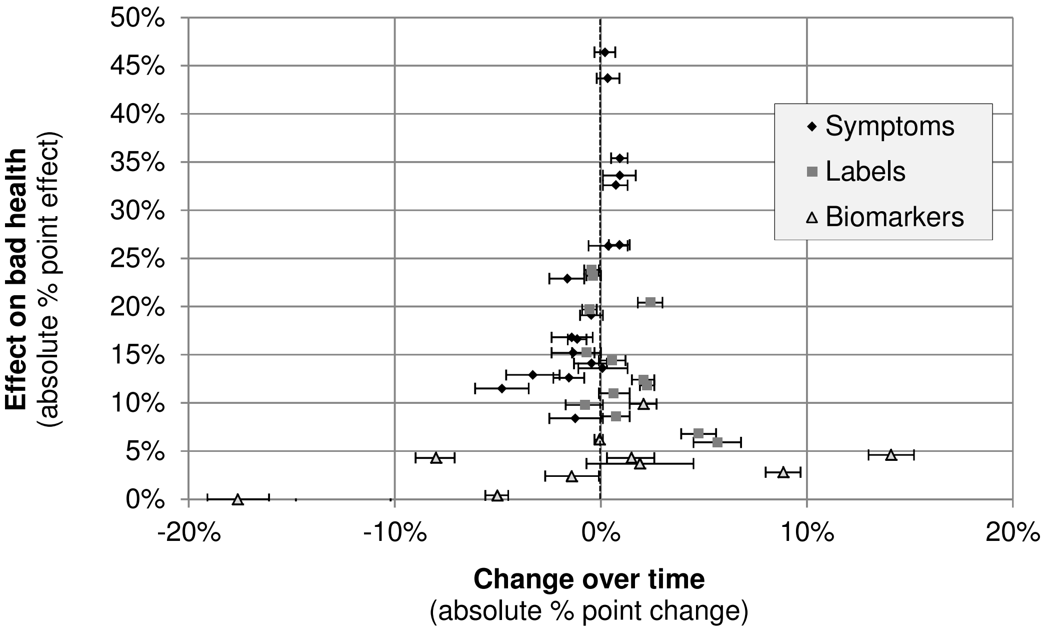

[a] 'Trend' is as reported above in the main paper. 'Effect on bad health' shows the effect of the morbidity measure on (very) bad health after controlling for age, sex (and their interaction) and educational level, using all years for which the individual morbidity measure is available. (This shows average marginal effects following a logistic regression; see text above).

It is easiest to interpret the figure by focussing on each group of measures in turn. Firstly, the biomarkers tend to have the weakest relationship with general health. Those with high levels of the diabetes biomarker (glycated haemoglobin) are 9.7% more likely to say they have bad health, and those who are underweight, with a high waist-hip ratio, raised fibrinogen, or low HDL cholesterol are 4-6% more likely to report bad health, but the other measures only had weaker relationships. Indeed, there was effectively no relationship between bad reported health and any of measured high blood pressure, high total cholesterol or iron deficiency.

Secondly, most of the measures based on medical labels have a moderately strong relationship with bad health (the weakest being lifetime asthma and recent high blood pressure, both of which can be asymptomatic), and these measures have mostly risen over time. There are however notable exceptions to this, including IHD/stroke LSI, recent angina and recent heart attack/stroke (the label-based measures with some of the strongest relationships with bad reported health), as well as arthritis and other musculoskeletal LSIs.

Finally, symptom-based measures unsurprisingly tend to have stronger relationships with bad reported health, although this ranges from the moderate (those reporting 'recent wheezing/asthma attack' were 8.5% more likely to report bad health) to the very strong (those reporting 'extreme

For peer review only - http://bmjopen.bmj.com/site/about/guidelines.xhtml

pain today' were 46.4% more likely to report bad health). In general, those symptoms-based measures with the strongest relationship with bad reported health were more likely to have increased over time ('extreme anxiety/depression today', 'locomotor limitations', and 'self-care limitations'). However, the size of the aforementioned declines in symptom-based measures of respiratory and cardiovascular morbidity was often greater.

For peer review only

For peer review only - http://bmjopen.bmj.com/site/about/guidelines.xhtml

Bibliography for Web Appendices

1. Case A, Deaton A. Rising morbidity and mortality in midlife among white non-Hispanic Americans in the 21st century. *Proceedings of the National Academy of Sciences* 2015;112(49):15078-83. doi: 10.1073/pnas.1518393112
2. Hiam L, Dorling D, Harrison D, et al. Why has mortality in England and Wales been increasing? An iterative demographic analysis. *Journal of the Royal Society of Medicine* 2017;110(4):153-62. doi: 10.1177/0141076817693599
3. Department of Health. Our Health and Wellbeing Today. London: HM Government, 2010.
4. Jagger C. Trends in life expectancy and healthy life expectancy. Future of an ageing population: evidence review. London: Foresight, Government Office for Science, 2015.
5. Office for Budget Responsibility. Welfare trends report: October 2016. Cm 9341. London: Her Majesty's Stationery Office, 2016.
6. Department for Work and Pensions, Department of Health. Improving Lives: The Work, Health and Disability Green Paper. Cm 9342. London: Her Majesty's Stationery Office, 2016.
7. Geiger BB. Morbidity in England 1994-2014 2019 [Available from:http://osf.io/dy6sv].
8. Szende A, Janssen B, Cabases J, editors. *Self-Reported Population Health: An International Perspective based on EQ-5D*. Netherlands: Springer, 2014.
9. Erens B, Primatesta P, Prior G. Health survey for England 1999: the health of minority ethnic groups. London: The Stationery Office, 2001.
10. National Heart Lung and Blood Institute. Sixth Report of the Joint National Committee on the Prevention, Detection, Evaluation and Treatment of High Blood Pressure. NIH Publication No 98-04080: National Institutes on Health, 1997.
11. Oyebode O. Cardiovascular disease. In: Craig R, Mindell J, eds. Health Survey for England 2011: Volume 1 - Health, social care and lifestyles. Leeds, UK: Health and Social Care Information Centre 2012.
12. Banks J, Marmot M, Oldfield Z, et al. The SES Health Gradient on Both Sides of the Atlantic. NBER Working Paper No 12674, 2006.
13. Lawlor DA, Adamson J, Ebrahim S. Performance of the WHO Rose angina questionnaire in post-menopausal women: Are all of the questions necessary? *Journal of Epidemiology and Community Health* 2003;57(7):538-41. doi: 10.1136/jech.57.7.538
14. Cook D, Shaper A, Macfarlane P. Using the WHO (Rose) Angina Questionnaire in Cardiovascular Epidemiology. *International Journal of Epidemiology* 1989;18(3):607-13. doi: 10.1093/ije/18.3.607
15. Fletcher C, Elmes P, Fairbairn M, et al. The significance of respiratory symptoms and the diagnosis of chronic bronchitis in a working population. *BMJ* 1959;2:257-66.
16. WHO. Physical status: the use and interpretation of anthropometry. Report of a WHO Expert Committee. WHO Technical Report Series 854. Geneva: World Health Organization (WHO), 1995.
17. National Obesity Observatory. Obesity and Overweight Surveillance in England: what is measured and where are the gaps?, 2009.
18. NICE. Obesity: The Prevention, Identification, Assessment and Management of Overweight and Obesity in Adults and Children NICE Clinical Guidelines, No 43. London: Centre for Public Health Excellence and National Collaborating Centre for Primary Care at the National Institute for Health and Clinical Excellence (NICE), 2006.
19. Hotchkiss JW, Davies CA, Gray L, et al. Trends in cardiovascular disease biomarkers and their socioeconomic patterning among adults in the Scottish population 1995 to 2009: cross-sectional surveys. *BMJ Open* 2012;2(3) doi: 10.1136/bmjopen-2011-000771
20. Moody A. Diabetes and hyperglycaemia. In: Craig R, Mindell J, eds. Health Survey for England 2011: Volume 1 - Health, social care and lifestyles. Leeds, UK: Health and Social Care Information Centre 2012.
21. Aresu M, Gordon-Dseagu V, Shelton N. Diabetes and glycaemia. In: Craig R, Hirani V, eds. Health Survey for England 2009, Volume 1: Health and lifestyles. Leeds, UK: The NHS Information Centre for health and social care 2010:59-74.

For peer review only - http://bmjopen.bmj.com/site/about/guidelines.xhtml

22. Steptoe A. Psychosocial biomarker research: integrating social, emotional and economic factors into population studies of aging and health. *Social Cognitive and Affective Neuroscience* 2011;6(2):226-33. doi: 10.1093/scan/nsq032
23. Hughes A, McMunn A, Bartley M, et al. Elevated inflammatory biomarkers during unemployment: modification by age and country in the UK. *Journal of Epidemiology and Community Health* 2015;69(7):673-79. doi: 10.1136/jech-2014-204404
24. Benzeval M, Davillas A, Kumari M, et al. Understanding Society: The UK Household Longitudinal Study Biomarker User Guide and Glossary (version 1). Colchester, Essex: Institute for Social and Economic Research, 2014.
25. Chaudhury M. Blood analytes. In: Sproston K, Primatesta P, eds. Health Survey for England, 2003, Vol 2: Risk Factors for Cardiovascular Disease: TSO 2004:241-88.
26. Erens B, Primatesta P. Health survey for England 1998: cardiovascular disease. *London: The Stationery Office* 1999
27. Chaudhury M, Tull K. Nutrition and haematological status. In: Craig R, Mindell J, eds. Health Survey for England, 2005: The health of older people, Vol 1: General health and function: TSO 2006:67-96.
28. Goldberg D, PA W. User Guide to the General Health Questionnaire. Windsor, UK: NFER-Nelson, 1988.
29. Stochl J, Böhnke JR, Pickett KE, et al. An evaluation of computerized adaptive testing for general psychological distress: combining GHQ-12 and Affectometer-2 in an item bank for public mental health research. *BMC Medical Research Methodology* 2016;16(1):58. doi: 10.1186/s12874-016-0158-7
30. ONS. Harmonised Concepts and Questions for Social Data Sources, Primary Principles: Long-lasting Health Conditions and Illnesses; Impairments and Disability [version 1.1]. London: Office for National Statistics (ONS), 2015.
31. Hall J, Mindell J. Respiratory symptoms and disease in adults In: Craig R, Mindell J, eds. Health Survey for England, 2010, Volume 1: Respiratory health: TSO 2011.
32. Sperrin M, Marshall A, Higgins V, et al. Slowing down of adult body mass index trend increases in England: a latent class analysis of cross-sectional surveys (1992-2010). *International Journal of Obesity* 2014;38(6)
33. Parsons S, Ingram M, Clarke-Cornwell AM, et al. A Heavy Burden: The occurrence and impact of musculoskeletal conditions in the United Kingdom today. Manchester: Arthritis Research UK & University of Manchester, 2011.
34. OECD. Fit Mind, Fit Job: From evidence to practice in mental health and work. Paris: OECD, 2015.
35. Spiers N, Qassem T, Bebbington P, et al. Prevalence and treatment of common mental disorders in the English national population, 1993–2007. *The British Journal of Psychiatry* 2016;209(2):150-56. doi: 10.1192/bjp.bp.115.174979
36. Moncrieff J, Pomerleau J. Trends in sickness benefits in Great Britain and the contribution of mental disorders. *Journal of Public Health Medicine* 2000;22:59-67.
37. Stansfeld SA, Woodley-Jones D, Rasul F, et al. Work-related distress in the 1990s - a real increase in ill health? . *Journal of Public Mental Health* 2008;7(1):25-31.
38. McManus S, Bebbington P, Jenkins R, et al., editors. *Mental health and wellbeing in England: Adult Psychiatric Morbidity Survey 2014*. Leeds: NHS Digital, 2016.
39. Maheswaran H, Kupek E, Petrou S. Self-reported health and socio-economic inequalities in England, 1996–2009: Repeated national cross-sectional study. *Social Science & Medicine* 2015;136–137:135-46. doi: http://dx.doi.org/10.1016/j.socscimed.2015.05.026
40. Katikireddi SV, Niedzwiedz CL, Popham F. Trends in population mental health before and after the 2008 recession: a repeat cross-sectional analysis of the 1991–2010 Health Surveys of England. *BMJ Open* 2012;2(5) doi: 10.1136/bmjopen-2012-001790
41. Jones M, Wass V. Understanding changing disability-related employment gaps in Britain 1998–2011. *Work, Employment & Society* 2013;27(6):982-1003. doi: 10.1177/0950017013475372

For peer review only - http://bmjopen.bmj.com/site/about/guidelines.xhtml

42. Barr B, Kinderman P, Whitehead M. Trends in mental health inequalities in England during a period of recession, austerity and welfare reform 2004 to 2013. *Social Science & Medicine* 2015;147:324-31. doi: http://dx.doi.org/10.1016/j.socscimed.2015.11.009
43. Tull KI, Hirani V, Ali A, et al. Impact of different diagnostic thresholds and the anaemia–ferritin–transferrin receptor model on the prevalence of anaemia and impaired iron status in older people. *Age and Ageing* 2009;38(5):609-13. doi: 10.1093/ageing/afp102
44. Dhalwani NN, O'Donovan G, Zaccardi F, et al. Long terms trends of multimorbidity and association with physical activity in older English population. *International Journal of Behavioral Nutrition and Physical Activity* 2016;13(1):8. doi: 10.1186/s12966-016-0330-9
45. Koné Pefoyo AJ, Bronskill SE, Gruneir A, et al. The increasing burden and complexity of multimorbidity. *BMC Public Health* 2015;15(1):415. doi: 10.1186/s12889-015-1733-2
46. Tetzlaff J, Junius-Walker U, Muschik D, et al. Identifying time trends in multimorbidity—defining multimorbidity in times of changing diagnostic practices. *Journal of Public Health* 2016:1-8. doi: 10.1007/s10389-016-0771-2
47. Marfeo EE, Haley SM, Jette AM, et al. Conceptual Foundation for Measures of Physical Function and Behavioral Health Function for Social Security Work Disability Evaluation. *Archives of Physical Medicine and Rehabilitation* 2013;94(9):1645-52.e2. doi: 10.1016/j.apmr.2013.03.015
48. Salomon JA, Vos T, Hogan DR, et al. Common values in assessing health outcomes from disease and injury: disability weights measurement study for the Global Burden of Disease Study 2010. *The Lancet* 2012;380(9859):2129-43. doi: http://dx.doi.org/10.1016/S0140-6736(12)61680-8
49. Murray CJL, Richards MA, Newton JN, et al. UK health performance: findings of the Global Burden of Disease Study 2010. *The Lancet* 2013;381(9871):997-1020. doi: https://doi.org/10.1016/S0140-6736(13)60355-4
50. WHO. Global Health Estimates 2015: Disease burden by Cause, Age, Sex, by Country and by Region, 2000-2015. Geneva: World Health Organization (WHO), 2016.
51. WHO. World report on disability. Geneva: World Health Organization (WHO) 2011.
52. Soldo BJ, Mitchell OS, Tfaily R, et al. Cross-cohort differences in health on the verge of retirement. NBER Working Paper No 12762: National Bureau of Economic Research, 2006.
53. Stewart ST, Cutler DM, Rosen AB. Comparison of Trends in U.S. Health-Related Quality of Life over the 2000's Using the SF-6D, HALex, EQ-5D, and EQ-5D Visual Analog Scale versus a Broader Set of Symptoms and Impairments. *Medical care* 2014;52(12):1010-16. doi: 10.1097/MLR.0000000000000181
54. Diederichs CP, Wellmann J, Bartels DB, et al. How to weight chronic diseases in multimorbidity indices? Development of a new method on the basis of individual data from five population-based studies. *Journal of Clinical Epidemiology* 2012;65(6):679-85. doi: 10.1016/j.jclinepi.2011.11.006

For peer review only

For peer review only - http://bmjopen.bmj.com/site/about/guidelines.xhtml

Reporting checklist for cross sectional study.

Based on the STROBE cross sectional guidelines.

Instructions to authors

Complete this checklist by entering the page numbers from your manuscript where readers will find each of the items listed below.

Your article may not currently address all the items on the checklist. Please modify your text to include the missing information. If you are certain that an item does not apply, please write "n/a" and provide a short explanation.

Upload your completed checklist as an extra file when you submit to a journal.

In your methods section, say that you used the STROBE cross sectionalreporting guidelines, and cite them as:

von Elm E, Altman DG, Egger M, Pocock SJ, Gotzsche PC, Vandenbroucke JP. The Strengthening the Reporting of Observational Studies in Epidemiology (STROBE) Statement: guidelines for reporting observational studies.

Author note: pages within the online Appendices are denoted by the prefix 'A'. Pages refer to submitted version with tracked changes visible.

		Reporting Item	Page Number
**Title and abstract**			
Title	#1a	Indicate the study's design with a commonly used term in the title or the abstract	1, 3
Abstract	#1b	Provide in the abstract an informative and balanced summary of what was done and what was found	3-4
**Introduction**			
Background / rationale	#2	Explain the scientific background and rationale for the investigation being reported	5-6
Objectives	#3	State specific objectives, including any prespecified hypotheses	5-10
**Methods**			
Study design	#4	Present key elements of study design early in the paper	6-14
Setting	#5	Describe the setting, locations, and relevant dates, including periods of recruitment, exposure, follow-up, and data collection	6-7, A6-9

For peer review only

Eligibility criteria	#6a	Give the eligibility criteria, and the sources and methods of selection of participants.	6-7, A6-9
	#7	Clearly define all outcomes, exposures, predictors, potential confounders, and effect modifiers. Give diagnostic criteria, if applicable	8-12, A12-23
Data sources / measurement	#8	For each variable of interest give sources of data and details of methods of assessment (measurement). Describe comparability of assessment methods if there is more than one group. Give information separately for for exposed and unexposed groups if applicable.	8-12, A12-23
Bias	#9	Describe any efforts to address potential sources of bias	6-14, 22, A6-9, A12-23
Study size	#10	Explain how the study size was arrived at	6, A9
Quantitative variables	#11	Explain how quantitative variables were handled in the analyses. If applicable, describe which groupings were chosen, and why	A12-23
Statistical methods	#12a	Describe all statistical methods, including those used to control for confounding	13-14
Statistical methods	#12b	Describe any methods used to examine subgroups and interactions	n/a
Statistical methods	#12c	Explain how missing data were addressed	A3-9
Statistical methods	#12d	If applicable, describe analytical methods taking account of sampling strategy	13-14
Statistical methods	#12e	Describe any sensitivity analyses	A10-11, A24-34
**Results**			
Participants	#13a	Report numbers of individuals at each stage of study—eg numbers potentially eligible, examined for eligibility, confirmed eligible, included in the study, completing follow-up, and analysed. Give information separately for for exposed and unexposed groups if applicable.	A3-A9
Participants	#13b	Give reasons for non-participation at each stage	A3-A9
Participants	#13c	Consider use of a flow diagram	n/a
Descriptive data	#14a	Give characteristics of study participants (eg demographic, clinical, social) and information on exposures and potential confounders. Give information separately for exposed and unexposed groups if applicable.	n/a [this is a descriptive study]

For peer review only

Descriptive data	#14b	Indicate number of participants with missing data for each variable of interest	A3-A9
Outcome data	#15	Report numbers of outcome events or summary measures. Give information separately for exposed and unexposed groups if applicable.	n/a [these form the main results]
Main results	#16a	Give unadjusted estimates and, if applicable, confounder-adjusted estimates and their precision (eg, 95% confidence interval). Make clear which confounders were adjusted for and why they were included	13-14, 15-21
Main results	#16b	Report category boundaries when continuous variables were categorized	A12-23
Main results	#16c	If relevant, consider translating estimates of relative risk into absolute risk for a meaningful time period	n/a [all estimates are given as absolute percentages]
Other analyses	#17	Report other analyses done—e.g., analyses of subgroups and interactions, and sensitivity analyses	A10-11, A24-34
**Discussion**			
Key results	#18	Summarise key results with reference to study objectives	21-23
Limitations	#19	Discuss limitations of the study, taking into account sources of potential bias or imprecision. Discuss both direction and magnitude of any potential bias.	21-22
Interpretation	#20	Give a cautious overall interpretation considering objectives, limitations, multiplicity of analyses, results from similar studies, and other relevant evidence.	21-23
Generalisability	#21	Discuss the generalisability (external validity) of the study results	21-23, A6-A9
**Other Information**			
Funding	#22	Give the source of funding and the role of the funders for the present study and, if applicable, for the original study on which the present article is based	1, A43

None The STROBE checklist is distributed under the terms of the Creative Commons Attribution License CC-BY. This checklist can be completed online using https://www.goodreports.org/, a tool made by the EQUATOR Network in collaboration with Penelope.ai

For peer review only

BMJ Open

Has working-age morbidity been declining? Changes over time in survey measures of general health, chronic diseases, symptoms and biomarkers in England 1994-2014

Journal:	*BMJ Open*
Manuscript ID	bmjopen-2019-032378.R3
Article Type:	Original research
Date Submitted by the Author:	22-Jan-2020
Complete List of Authors:	Geiger, Ben; University of Kent, School of Social Policy, Sociology and Social Research (SSPSSR);
**Primary Subject Heading**:	Public health
Secondary Subject Heading:	Research methods
Keywords:	Morbidity, Population Health, Biomarkers, Cross-sectional studies, Trends, England

SCHOLARONE™
Manuscripts

I, the Submitting Author has the right to grant and does grant on behalf of all authors of the Work (as defined in the below author licence), an exclusive licence and/or a non-exclusive licence for contributions from authors who are: i) UK Crown employees; ii) where BMJ has agreed a CC-BY licence shall apply, and/or iii) in accordance with the terms applicable for US Federal Government officers or employees acting as part of their official duties; on a worldwide, perpetual, irrevocable, royalty-free basis to BMJ Publishing Group Ltd ("BMJ") its licensees and where the relevant Journal is co-owned by BMJ to the co-owners of the Journal, to publish the Work in this journal and any other BMJ products and to exploit all rights, as set out in our licence.

The Submitting Author accepts and understands that any supply made under these terms is made by BMJ to the Submitting Author unless you are acting as an employee on behalf of your employer or a postgraduate student of an affiliated institution which is paying any applicable article publishing charge ("APC") for Open Access articles. Where the Submitting Author wishes to make the Work available on an Open Access basis (and intends to pay the relevant APC), the terms of reuse of such Open Access shall be governed by a Creative Commons licence – details of these licences and which Creative Commons licence will apply to this Work are set out in our licence referred to above.

Other than as permitted in any relevant BMJ Author's Self Archiving Policies, I confirm this Work has not been accepted for publication elsewhere, is not being considered for publication elsewhere and does not duplicate material already published. I confirm all authors consent to publication of this Work and authorise the granting of this licence.

For peer review only

Has working-age morbidity been declining? Changes over time in survey measures of general health, chronic diseases, symptoms and biomarkers in England 1994-2014

Authors:

Dr Ben B. Geiger, University of Kent, UK

Postal address: Ben B Geiger, School of Social Policy, Sociology and Social Research (SSPSSR), University of Kent, Canterbury, Kent, CT2 7NZ, UK

Email: b.b.geiger@kent.ac.uk

Word count *(exc. title page, abstract, tables, acknowledgements, contributions & references)*

3,950 words + 5 tables

MeSH Keywords

Morbidity; Population Health; Biomarkers; Cross-Sectional Studies; Trends; England

Acknowledgements

This work was supported by the Economic and Social Research Council (grant number ES/K009583/1).

For peer review only

Author statement

BBG was responsible for the design, data preparation, analysis and reporting of the study.

Acknowledgements

Many thanks to Clare Bambra for comments, and to Mariska van der Horst for research assistance; neither should be held responsible for the analysis or interpretation of the paper itself.

Funding

This work was supported by the Economic and Social Research Council (ESRC), grant number ES/K009583/1.

Competing interests

The author has worked on secondment at the UK Department for Work and Pensions (DWP) in 2015-16.

Data sharing

The Health Survey for England 1994-2014 are available for free to registered users at the UK Data Service - see https://beta.ukdataservice.ac.uk/datacatalogue/series/series?id=2000021#!/abstract. There are no conditions for re-use for non-commercial applications of the data.

For peer review only - http://bmjopen.bmj.com/site/about/guidelines.xhtml

The statistical code enabling replication using publicly available data is available from OSF (Morbidity in England 1994-2014 2019, available from: http://osf.io/dy6sv) and www.benbgeiger.co.uk.

For peer review only

For peer review only - http://bmjopen.bmj.com/site/about/guidelines.xhtml

Has working-age morbidity been declining? Changes over time in survey measures of general health, chronic diseases, symptoms and biomarkers in England 1994-2014

Abstract:

Objectives: As life expectancy has increased in high-income countries, there has been a global debate about whether additional years of life are free from ill-health/disability. However, little attention has been given to changes over time in morbidity in the *working-age* population, particularly outside the US, despite its importance for health monitoring and social policy. This study therefore asks: what are the changes over time in working-age morbidity in England over two decades?

Design, setting and participants: We use a high-quality annual cross-sectional survey, the Health Survey for England ('HSE') 1994-2014. HSE uses a random sample of the English household population, with a combined sample size of over 140,000 people. We produce a newly-harmonised version of HSE that maximises comparability over time, including new non-response weights. While HSE is used for monitoring population health, it has hitherto not used for investigating morbidity as a whole.

Outcome measures: We analyse all 39 measures that are fully comparable over time – including chronic disease diagnoses, symptomatology and a number of biomarkers – adjusting for gender and age.

For peer review only - http://bmjopen.bmj.com/site/about/guidelines.xhtml

Results: We find a mixed picture: we see improving cardiovascular and respiratory health, but deteriorations in obesity, diabetes, some biomarkers, and feelings of extreme anxiety/depression, alongside stability in moderate mental ill-health and musculoskeletal-related health. In several domains we also see stable or rising chronic disease *diagnoses* even where *symptomatology* has declined. While data limitations make it challenging to combine these measures into a single morbidity index, there is little systematic trend for declining morbidity to be seen in the measures that predict self-reported health most strongly.

Conclusions: Despite considerable falls in working-age mortality – and the assumptions of many policymakers that morbidity will follow mortality – there is no systematic improvement in overall working-age morbidity in England from 1994 to 2014.

Strengths and limitations of this study

- We provide a robust analysis of changes over time in morbidity in England for 39 measures across two decades using the Health Survey for England ('HSE').
- We include every morbidity measure for which consistent comparisons over time can be constructed in the HSE.
- We take care to maximise comparability over time, including constructing new non-response weights.

For peer review only - http://bmjopen.bmj.com/site/about/guidelines.xhtml

- However, response rates for each stage of the HSE have declined over time, and it is impossible to rule out changing non-response biases.
- There are also several dimensions of morbidity for which there is little trend data in HSE.

For peer review only

For peer review only - http://bmjopen.bmj.com/site/about/guidelines.xhtml

INTRODUCTION

As life expectancy has increased in high-income countries, there has been a global debate about whether additional years of life are free from ill-health/disability. It is now largely accepted that old-age disability has declined in the US (albeit varying by age/method),[1 2] although chronic illness increased,[3] and the picture beyond the US is more mixed.[4-6] Yet this research agenda has not been matched by similar attention to changes over time in morbidity in the *working-age* population. In the absence of direct evidence, policymakers have often made claims based on self-reports of general health,[6-8] which we know are unreliable.[9 10] The lack of evidence is even more problematic within social security, where many policymakers have assumed that working-age morbidity *must* have improved in recent decades given improvements in mortality (despite the potential for declining mortality to coexist with rising morbidity)[6] – and that therefore high/rising levels of claims are not 'genuine'.[11 12]

Almost the only direct evidence on changes over time in working-age morbidity in high-income countries comes from the US. Contrary to policymaker expectations, these studies have generally found *deteriorating* morbidity since the mid-1990s, particularly activities of daily living (ADLs) and physical functioning.[13-16] Other studies have focused on the older working-age population with similar results.[2 17] Again, not all measures show deteriorations, and not all studies come to identical conclusions,[18] but there is little sign of any improvement in morbidity among working-age Americans – despite a 23% fall in working-age mortality 1993-2013 (Web Appendix

For peer review only - http://bmjopen.bmj.com/site/about/guidelines.xhtml

1). Outside of the US, there is a paucity of evidence, but from the limited evidence that exists, there is again little sign of improving morbidity.[19-22]

This study therefore asks: is there empirical support for the hypothesis that working-age morbidity in England has declined? (H_1). Or does the evidence support alternative hypotheses of stable (H2) or even declining (H3) morbidity? We answer this using the Health Survey for England (HSE), a high quality Government survey with a combined sample of 140,000 individuals. We examine 39 specific aspects of morbidity rather than reducing morbidity to a single measure, partly because these produce more reliable trends, and partly to capture the multidimensional nature of morbidity.[23] However, we conclude by examining the broad picture of morbidity change, and how far this supports the competing hypotheses.

This analysis makes two contributions. Firstly, we provide one of the few systematic analyses of changes over time in working-age morbidity in any high-income country outside the US. Secondly, we supplement self-report measures with 10 'biomarkers', which are particularly valuable for showing genuine changes over time (rather than merely changes in how people describe their health), but which have rarely been examined alongside self-reported working-age morbidity trends (Martin et al. 2010[24] being an exception).

DATA AND METHODS

This section follows the STROBE cross sectional reporting guidelines.[25]

For peer review only - http://bmjopen.bmj.com/site/about/guidelines.xhtml

Data source

Robust evidence of change over time requires consistently-collected, high-quality data. We use the HSE, an annual government-sponsored cross-sectional survey of 3,000-11,000 adults with no proxy responses.[26-47] A particular advantage is that the interview is followed by a nurse visit, which in selected years also includes a blood sample. Nevertheless, there are challenges in analysing change in HSE:

- Firstly, HSE was run by the Government Office of Population Censuses and Surveys in 1991-3, before changing to NatCen in 1994. We focus on 1994-2014 given evidence of a discontinuity at this point.
- Secondly, topic coverage of HSE varies year-to-year, accompanied by changes in question wording/filtering. Based on a systematic search of HSE questions, we have included every morbidity measure that is comparable over a significant duration. Even for measures that have been previously been analysed (e.g. BMI[48]), this new analysis uncovered further discontinuities (Web Appendices 2 & 3).
- Third, HSE excludes those in communal establishments. While a smaller problem for the working-age population than older ages,[2] we minimise the impact of rising university attendance by focussing on those aged 25+ (Web Appendix 3). The upper limit of the working-age population is set to 59 (women) and 64 (men) to match state pension ages at the start of the period.

For peer review only

For peer review only - http://bmjopen.bmj.com/site/about/guidelines.xhtml

- Fourth, HSE supplies non-response weights from 2003. However, there had been a substantial decline in response rates prior to the introduction of weights, particularly for blood samples (from 53.3% 1994 to 39.9% 2003; Web Appendix 3). We therefore reduce non-response biases by creating new non-response weights, described in Web Appendix 3.

The resulting sample sizes for the various stages of data collection are shown in Web Appendix 3. Our dataset substantially extends an existing HSE time-series dataset (UK Data Archive SN7025); the code enabling other researchers to assemble this extended time-series dataset are freely available.[49]

Patient involvement

As this is a health monitoring study using secondary data, patients were not directly involved. However, from previous discussions we are aware that the study will be of interest to patient/disability advocacy groups, who will receive jargon-free summaries of the research.

Measures

We cannot interpret changes over time correctly without understanding different ways of operationalising 'morbidity'.[1] General health/disability measures – e.g. *"How is your health in general?"* – are a simple way of measuring morbidity with a single indicator, and clearly do capture something meaningful.[50] However, their generality means that despite consistent question wording, different people may interpret questions or response options differently (e.g. what 'good' health refers to).[51 p218-224]

For peer review only - http://bmjopen.bmj.com/site/about/guidelines.xhtml

This can even occur *within* individuals, if they change their internal standards of measurement over time (contributing to 'response shift'[52]). Numerous causal factors contribute to variable comprehension/reporting, ranging from the experience of ill-health itself[52] to non-health factors such as social security incentives,[53] gendered- and age-related expectations, and medicalisation.[54]

These inconsistencies mean that general health/disability measures are inadequate for answering our question: trends in such measures can differ wildly between different surveys covering nominally the same concept and population, e.g. for disability in England[9] or self-rated health in the US.[10] Indeed, the HSE itself shows that England has experienced deteriorating 'bad general health' at the same time as activity limitations have fallen (changes over time in seven general HSE health/disability measures are available in Web Appendix 4). Moreover, single indicator measures are potentially misleading in that they gloss over the multidimensional nature of morbidity.[1]

To robustly answer our research question, we must instead focus on more *specific* morbidity measures that capture multiple aspects of morbidity. Our systematic search found 39 such measures that are comparable over time: these are summarised in Table 1, with further details in Web Appendix 5. (A further 29 measures are also included in Web Appendix 6; this includes 8 sub-components of measures in the main text, 16 reports of ever having a condition even if this not recent, and 5 other

For peer review only - http://bmjopen.bmj.com/site/about/guidelines.xhtml

categories of LSI). These specific morbidity measures can be grouped into three types, which have different strengths and weaknesses with respect to our question:

1. *Medical labels:* some measures are based on medical labels, either diagnosed chronic diseases or self-reported types of longstanding illness. (Those reporting a longstanding illness were asked, *'what is the matter with you?'*; up to 6 responses were then coded by the interviewer based on ICD). These are imperfect measures of morbidity[55] as they partly reflect healthcare systems and medicalisation more broadly, both of which change over time. Nevertheless, they are an important element of morbidity as they have real consequences via increasing awareness/labelling of people's experiences.

2. *Symptom-based:* some measures are based on self-reports of ill-health symptoms or specific domains of activity limitations. These measures are either single items (e.g. pain, anxiety/depression) or validated symptom scales (e.g. the Rose angina scale,[56 57] GHQ psychiatric distress[58]). The more specific and concrete nature of these measures *prima facie* makes more likely to be interpreted consistently over time than medical labels and general measures,. Others have reached a similar conclusion for comparisons across place,[55] particularly for disability measurement,[59 60] where the Washington Group on Disability Statistics – a UN agency founded in 2001 – have brokered a consensus that cross-country disability comparisons should be based on multiple measures of specific activity limitations.[61 62] We should nevertheless

For peer review only - http://bmjopen.bmj.com/site/about/guidelines.xhtml

note that there is no guarantee that a given symptom/impairment-based question will be interpreted identically over time.[63 64]

3. *Biomarkers* – that is, objective measures of biological or physiological measures – have considerable strengths in analysing change, as they largely avoiding reporting biases that are likely to vary between socioeconomic groups and over time.[65] They do this at the price of an indirect and sometimes still-debated relationship to morbidity (see Web Appendix 5), and do not cover several important morbidity domains (e.g. we lack good biomarkers for mental distress, pain and fatigue).

These three types of measures are therefore complementary in understanding changing morbidity: biomarkers are least likely to be affected by changing respondent interpretations over time, but do not capture morbidity well; symptom-based measures capture morbidity well and are reasonably (if still imperfectly) reliable; and label-based measures are flawed in capturing symptoms/limitations but do enable us to capture whether people consider themselves to have a medical condition.

For peer review only - http://bmjopen.bmj.com/site/about/guidelines.xhtml

Table 1: HSE morbidity measures

Category	Measure	Type[a]	Operationalisation (years available)
Cardio-vascular disease (CVD)	High blood pressure LSI[b]	L	Hypertension reported as longstanding illness (LSI) (1994-2011)
	Recent high blood pressure	L	Still has (or on medication for) doctor-diagnosed hypertension (1994-2013)
	Biomarker high blood pressure	B	Systolic BP >=140mmHg & diastolic BP >=90mmHg (1994-2013)
	High total cholesterol	B	Total cholesterol >= 5mmol/L (1994-2012)
	Low HDL cholesterol	B	High density lipoprotein (HDL) cholesterol <=1 mmol/L (1998-2013)
	Recent heart attack /stroke	L	Doctor-diagnosed heart attack or stroke in past 12mths (1994-2011)
	Recent angina	L	Doctor-diagnosed angina in past 12mths (1994-2011)
	Ischaemic heart/stroke LSI[b]	L	Stroke, heart attack or angina reported as longstanding illness (LSI) (1994-2011)
	Heart attack symptoms	S	Ever had severe pain across chest for ½hr (1994-2011)
	Mini stroke (TIA) symptoms	S	Attack of weakness/slurred speech/blurred vision in past 12mths (2003-11)
	Angina symptoms	S	Rose Angina scale definition of angina symptoms (1994-2011)
	Any recent CVD	L	Doctor-diagnosed heart condition (exc. hypertension) in past 12mths (1994-2011)
	Any CVD LSI	L	Any CVD reported as longstanding illness (LSI) (1994-2011)
Respiratory	COPD symptoms	S	Regular cough & phlegm for at least 3mths each year (1995-2010)
	Lifetime diagnosed asthma	L	Ever had doctor-diagnosed asthma (1995-2010)
	Asthma LSI[b]	L	Asthma reported as longstanding illness (LSI) (1994-2011)
	Breathlessness-grade 2	S	Short of breath when hurrying up walking uphill (1995-2010)
	Breathlessness-grade 3	S	Short of breath when walking on level ground (1995-2010)
	Recent wheezing/asthma	S	Wheezing, whistling in chest or asthma attack in past 12mths (1995-2010)
	Wheezing stopping sleep	S	Woken 1+ times/wk by wheezing/whistling in chest in last 12mths (1994-2010)
Obesity & diabetes	BMI-underweight	B	Body Mass Index (BMI) <=18.5kg/m^2 (1994-2013)
	BMI-obese	B	Body Mass Index (BMI) >= 30kg/m^2 (1994-2013)
	High waist-hip ratio	B	Waist-hip ratio of >1 for men and >0.85 for women (1994-2013)
	Recent diabetes	L	Currently taking medication for doctor-diagnosed diabetes (1994-2013)
	Diabetes LSI[b]	L	Diabetes reported as longstanding illness (LSI) (1994-2011)
	High glycated haemoglobin	B	HbA_{1C} >=48mmol/mol (2003-2013)
Mental	Mental health LSI[b]	L	Mental health reported as longstanding illness (LSI) (1994-2011)

For peer review only - http://bmjopen.bmj.com/site/about/guidelines.xhtml

Category	Measure	Type[a]	Operationalisation (years available)
Health	Psychiatric distress (GHQ)	S	4+ negative symptoms from 12-item General Health Questionnaire (1994-2014)
	Anxiety/depression-moderately	S	At least moderately anxious/depressed today (1996-2014)
	Anxiety/depression-extremely	S	Extremely anxious/depressed today (1996-2014)
Activity limitations & musculo-skeletal	Problems walking today	S	Has at least some problems walking about today (1996-2014)
	Locomotor limitation	S	Can't walk far / bend down / go up or down stairs without resting (1996-2001)
	Problems washing/dressing today	S	Has at least some problems washing/dressing today (1996-2014)
	Self-care limitation	S	Difficulty with one of six everyday activities (e.g. feeding, dressing) (1995-2001)
	Pain-any	S	Has at least some pain or discomfort today (1996-2014)
	Pain-extreme	S	Has extreme pain or discomfort today (1996-2014)
	Arthritis LSI[b]	L	Arthritis or rheumatism reported as longstanding illness (LSI) (1994-2011)
	Other musculoskeletal LSI[b]	L	Other musculoskeletal condition reported as longstanding illness (LSI) (1994-2011)
Sensory & Communication	LSI Eye or Ear	L	Eye or ear condition reported as longstanding illness (LSI) (1994-2011)
	Hearing limitation	S	Cannot follow TV programme at volume others find acceptable (1995-2001)
	Seeing limitation	S	Cannot see well enough to recognise friend across the road (1995-2001)
	Communicating limitation	S	Have problem communicating with other people (1995-2001)
Other Biomarkers	Raised C-reactive protein	B	CRP >3mg/L (1998-2009)
	Raised fibrinogen	B	Fibrinogen >4mg/L (1998-2009)
	Anaemia	B	Haemoglobin <13g/dL for men and <12g/dL for women (1994-2009)
	Iron deficiency	B	Serum ferritin < 45ng/ml (1994-2009)

See Web Appendix 5 for full details on all measures .[a] Measure type key: L=medical label; S=symptom-based; B=biomarker. [b] Particular causes of longstanding illness (LSI) come from the open question, 'what is the matter with you?' Up to 6 responses are then coded by the interviewer into a coding frame based on ICD.

For peer review only

For peer review only - http://bmjopen.bmj.com/site/about/guidelines.xhtml

ANALYSIS

In the first instance we look at unadjusted changes over time in each morbidity indicator, showing the actual levels of morbidity found in the population. However, we primarily focus on changes after adjustment for sex and age (following others[66 67]), akin to standardising for the age-sex composition of the population. Given that our aim is to *describe* changes rather than to explain them, we do not further adjust for potential causal influences on morbidity that are likely to vary over the period, such as employment over economic cycles. This is a task for future research, but we should note that such analysis is possible using our publicly-available time-series dataset that includes *inter alia* employment status, education and region.

We chose to examine discrete changes from the start to the end of available data for each measure, rather than using linear or non-linear trend terms. Given our aims of informing policy debates, this has three advantages: a discrete change is simple to interpret; it is compatible with the different start/end years available for different measures; and it does not require any assumptions about the functional form of trends (linear trends are particularly unlikely given the role of non-linear economic cycles). Individual survey years are grouped into 3-4 year periods to increase sample size and precision, but single-year prevalence is given in Web Appendix 7. Given our binary outcome measures, we use logistic regression models with the following form:

$$y_i = logit\,[\,\beta_1 period_i + \beta_2 age_i + \beta_3 male_i + \beta_4 (age_i * male_i)\,]$$

For peer review only - http://bmjopen.bmj.com/site/about/guidelines.xhtml

…where $\underline{period_i}$ refers to a vector of period dummy variables (covering all periods in which there were any observations: 1994-96, 1997-2000, 2001-03, 2004-07, 2008-10 and 2011-14), $\underline{\beta_1}$ is a vector of our primary outcome coefficients showing change between each period and the earliest available period, $\underline{age_i}$ refers to a vector of age dummy variables, $\underline{male_i}$ refers to a binary gender dummy variable, and $\underline{\beta_2}$, $\underline{\beta_3}$ and $\underline{\beta_4}$ refer to the coefficients on age, gender and their interaction respectively. We present average marginal effects rather than odds ratios, partly because these are simple to understand – odds ratios have no easy real-world interpretation for policymakers – but primarily because odds ratios are not fully comparable across different models, and cannot therefore underpin our comparison of changes over time between indicators.[68]

To avoid a binary cut-off of statistical significance,[69] 95% confidence intervals are used to convey precision. All analyses use weights, exclude boost samples that use different sampling methods, and adjust for the multistage clustered sample design and the stratification of the sample across survey years using the SVYSET command in Stata (although standard errors will be slightly underestimated as it is not possible to consistently adjust for sample stratification within years). For reasons of space, we are unable to discuss previous HSE studies of aspects of morbidity in the main text; these are instead described in Web Appendix 8.

For peer review only - http://bmjopen.bmj.com/site/about/guidelines.xhtml

For peer review only

RESULTS

Conditions with sharply declining mortality

We start by focussing on cardiovascular disease (CVD) and respiratory illness, which have both seen large falls in mortality (by >50% and >25% respectively among 0-64 year-olds 1994-2013; Web Appendix 1). Changes over time in *morbidity*, however, are shown in Table 2.

Table 2: Changes over time in cardiovascular and respiratory morbidity

	Starting period		Change from start to end period			
	Period	Prevalence	End period	Raw change	Adj.[a] change	Adj. ch[illegible] 95%[illegible]
**Blood pressure/cholesterol**						
High blood pressure LSI[b]	1994-96	2.7%	2011-14	1.3%	1.0%	[0.4, 1[illegible]
Recent high blood pressure	1994-96	4.2%	2011-14	5.2%	4.8%	[3.9, 5[illegible]
Biomarker high BP	1994-96	8.4%	2011-14	-4.7%	-5.0%	[-5.6, -[illegible]
High total cholesterol	1994-96	75.7%	2011-14	-16.4%	-17.6%	[-19.1, -[illegible]
Low HDL cholesterol	1997-2000	11.8%	2011-14	-8.0%	-8.0%	[-9.0, -[illegible]
**Other CVD**						
Recent heart attack/stroke	1994-96	1.2%	2011-14	-0.3%	-0.4%	[-0.7, [illegible]
Recent angina	1994-96	1.1%	2011-14	-0.4%	-0.5%	[-0.8, -[illegible]
IHD/stroke LSI[b]	1994-96	1.4%	2011-14	-0.4%	-0.6%	[-0.9, -[illegible]
Heart attack symptoms	1994-96	5.5%	2011-14	-0.3%	-0.5%	[-1.3, [illegible]
Mini stroke (TIA) symptoms	2001-03	8.1%	2011-14	-1.4%	-1.4%	[-2.4, -[illegible]
Angina symptoms	1994-96	2.3%	2011-14	-1.1%	-1.2%	[-1.6, -[illegible]
Any CVD LSI[b]	1994-96	5.8%	2011-14	1.1%	0.6%	[-0.1, 1[illegible]
Any recent CVD	1994-96	3.1%	2011-14	0.7%	0.5%	[-0.1, 1[illegible]
**Respiratory**						
Lifetime diagnosed asthma	1994-96	11.2%	2008-10	5.5%	5.7%	[4.5, 6[illegible]
Asthma LSI[b]	1994-96	5.0%	2011-14	0.7%	0.7%	[0.0, 1[illegible]
Breathlessness-Grade 2+	1994-96	19.7%	2008-10	-4.4%	-4.8%	[-6.1, -[illegible]
Breathlessness-Grade 3	1994-96	7.8%	2008-10	-1.4%	-1.6%	[-2.5, -[illegible]
Recent wheezing/asthma	1994-96	19.5%	2008-10	-1.2%	-1.2%	[-2.5, [illegible]
Wheezing stopping sleep	1994-96	3.6%	2008-10	-0.4%	-0.5%	[-1.0, [illegible]
COPD symptoms	1994-96	6.6%	2008-10	-1.5%	-1.6%	[-2.3, -[illegible]

[a] 'Adj.' = adjusted for changing age and sex distribution of the working-age population. [b] LSI=longstanding illness; see Table 1.

For peer review only - http://bmjopen.bmj.com/site/about/guidelines.xhtml

Looking first at high blood pressure, biomarker-measured high blood pressure has halved over two decades (similar improvements are found for the biomarkers for total and HDL cholesterol). Yet when we look at self-reports (either people reporting this as an LSI, or in response to a direct question about having recent diagnosed high blood pressure), we see large *rises* over time. There has been an increasing diagnosis of high blood pressure and increasing prescriptions of blood pressure-lowering drugs; these may have helped reduce the underlying incidence of high blood pressure while simultaneously raising people's awareness of morbidity.

Table 2 further shows declines in several key types of CVD (heart attack, mini-stroke, angina), whether measured through people's reports of the disease itself or their reports of its symptoms. Nevertheless, the morbidity declines (8-50%) are often not on the scale of the declines in mortality (>50%); this is likely to be because mortality declines are partly driven by improved treatment,[70] which means each incident CVD case is likely to last longer.[71 72] More surprisingly, the measures of 'any reported CVD' show no improvement (with some, uncertain signs of *rises*). Looking at its sub-components (Web Appendix 6), this seems to be due to possible increases in diagnosed irregular heart rhythm and other heart trouble.

Finally, Table 2 shows that symptoms-based measures of respiratory morbidity have improved, particularly COPD symptoms (regular cough & phlegm) and breathlessness (at both levels), and more uncertainly for recent wheezing/asthma and wheezing stopping sleep. Again, though, diagnosis-related measures of asthma

For peer review only - http://bmjopen.bmj.com/site/about/guidelines.xhtml

– reported diagnoses, or self-reports of having asthma as a longstanding illness – have risen, even while underlying symptomatology is improving.

Overall, Table 2 illustrates how changes over time in morbidity do not necessarily follow changes in mortality. There are definite improvements in CVD risk factors and respiratory symptomatology on the scale of improvements in mortality. But the prevalence of self-reported CVD conditions such as heart attacks have only declined by a smaller amount, and recent doctor-diagnosed hypertension, any CVD, and asthma diagnoses have either stayed stable or risen.

For peer review only

Conditions with claims of increasing prevalence

The previous section focussed on conditions where there may be an *a priori* expectation that morbidity has improved (given declining mortality); in this section, we focus on three areas where there have been widespread claims of increasing prevalence – obesity, diabetes, and mental health.

Looking at Table 3, we do indeed confirm a large rise in obesity in HSE (an 8.0-9.7% rise from an obesity prevalence of 16.9% in 1994-96). The rise in high waist-hip ratios – sometimes suggested to be a better measure of potential morbidity [73] – is even larger. This has come alongside little change in the prevalence of being *underweight* over this period.

For peer review only - http://bmjopen.bmj.com/site/about/guidelines.xhtml

Table 3: Changes over time in obesity, diabetes and mental health

	Starting period		Change from start to end period			
	Period	Prevalence	End period	Raw change	Adj.[a] change	Adj. change 95% CI
**Underweight/Obesity**						
BMI-Underweight	1994-96	1.0%	2011-14	-0.1%	-0.1%	[-0.3, 0.1%]
BMI-Obese	1994-96	16.9%	2011-14	9.3%	8.9%	[8.0, 9.7%]
High waist-hip ratio	1994-96	9.5%	2011-14	14.8%	14.1%	[13.0, 15.2%]
**Diabetes**						
Recent diabetes	1994-96	1.2%	2011-14	2.4%	2.2%	[1.9, 2.6%]
Diabetes LSI[b]	1994-96	1.5%	2011-14	2.3%	2.1%	[1.5, 2.6%]
Glycated haemoglobin	2001-03	2.7%	2011-14	2.1%	2.1%	[1.4, 2.7%]
**Mental health**						
Mental health LSI[b]	1994-96	2.1%	2011-14	2.5%	2.4%	[1.8, 3.0%]
Psychological distress[c]	1994-96	17.1%	2011-14	-1.3%	-1.3%	[-2.4, -0.3%]
Anx./depression-moderate[d]	1994-96	21.9%	2011-14	0.3%	0.1%	[-1.1, 1.3%]
Anx./depression-extremely[d]	1994-96	1.8%	2011-14	1.0%	0.9%	[0.5, 1.3%]

[a] 'Adj.' = adjusted for changing age and sex distribution of the working-age population. [b] LSI=longstanding illness; see Table 1. [c] GHQ; see Web Appendix 5. [d] 'Anx./depression'= Feeling of anxiety/depression today – see Table 1.

Table 3 also confirms a large rise in diabetes. This can be seen whether diabetes is measured through people reporting diabetes as an LSI, a specific question about people currently taking medication for diabetes, or via a diabetes biomarker (glycated haemoglobin). It is worth noting that this clear rise in diabetes has occurred despite a *decline* in the age 0-64 death rate from diabetes, by more than one-third 1994-2013 (Web Appendix 1) – indeed, rising prevalence is *because of* falling mortality [74] – again demonstrating the difference between changes in mortality and morbidity.

Trends in mental health are more contentious in the wider literature (see Web Appendix 8), and the measures in HSE are not as strong as the more occasional Adult

For peer review only - http://bmjopen.bmj.com/site/about/guidelines.xhtml

Psychiatric Morbidity Surveys.[75] Nevertheless, HSE offers a unique annual perspective on self-reported mental health. As we might expect from increasing treatment/diagnosis, we see a doubling in people reporting a mental health LSI. However, the symptoms-based measures show a more mixed picture:

- Neither of the measures that capture more moderate mental ill-health show rising ill-health (these are psychological distress symptoms and people reporting a feeling of anxiety/depression today, both with a relatively common prevalence of 15-25%). If we break this down by year (see Web Appendix 7), we can see moderate mental ill-health symptoms fell between the mid-1990s and the mid-2000s, before rising in 2009.
- In contrast, the single measure capturing a feeling of extreme anxiety/depression today does show rising morbidity. To see if there were similar signs of rising mental ill-health at extremes in our other measure (psychological distress), we looked at a much higher GHQ threshold of 10 negative responses out of 12 questions (compared to the conventional threshold of 4). Unlike the conventional GHQ measure, this also showed an increase over time (95% CI of a 0.4 to 1.4% rise; see Web Appendix 6). While the GHQ is not designed to capture *severe* psychological distress in this way, others have similarly looked at moderate and extreme psychological distress using GHQ – and indeed, have found that rises in distress over time 1991-2008 are concentrated in the more extreme measure.[76]

For peer review only - http://bmjopen.bmj.com/site/about/guidelines.xhtml

Overall, while labelling of mental health conditions has undoubtedly risen, trends in mental health symptoms vary across measures. If we interpret higher GHQ thresholds as indicating more serious psychological distress, then we can see a consistent picture: moderate mental ill-health symptoms fell from the mid-1990s to the mid-2000s before rising around the time of the 2008 economic crisis (as we would expect[77]), whereas more extreme mental ill-health has more consistently risen.

Activity limitations, musculoskeletal and pain

Pain/musculoskeletal conditions are a major component of working-age morbidity, yet very few previous studies show changes over time in symptomatology, and even those that exist[78] sometimes have debatable comparability.[79] Table 4 shows a fall in some – but not all – HSE measures focussed on pain and musculoskeletal morbidity. Arthritis as a longstanding illness (LSI) has declined (the precision of the estimates is greater when looking at 2008-10 rather than 2011-14, and shows a decline of 0.3-1.2%). There are some (similarly uncertain) signs that other musculoskeletal LSIs have also fallen, and noticeably fewer people say that they have any pain/discomfort today, although there has been no change in people saying they have extreme pain/discomfort. The echoes a previous study that found different trends in low back pain of different levels of severity.[80]

Similarly, there has been a rise in all four activity limitations measures in HSE – although the increases are sometimes uncertain, and are smaller after adjusting for changes in age/sex structure. Moreover, the timing of the rises differ between the

For peer review only - http://bmjopen.bmj.com/site/about/guidelines.xhtml

measures: the trend in limitations lasting at least a year shows a rise 1994-6 to 2001-3, but the two measures of 'limitations today' do not, instead showing a possible slight rise in the more recent period (see Web Appendix 7; this difference remains if we focus on the sub-components of year-long limitations that more closely match to the 'limitations today' questions, see Web Appendix 6). Still, the measures can collectively be seen as offering some, albeit relatively weak, evidence for an increase in activity limitations.

Table 4: Changes over time in activity limitations, pain & musculoskeletal morbidity

	Starting period		Change from start to end period			
	Period	Prevalence	End period	Raw change	Adj.[a] change	Adj. change 95% CI
**Activity limitations**						
Problems walking about	1994-96	11.5%	2011-14	1.0%	0.4%	[-0.6, 1.3%]
Any locomotor limitation	1994-96	6.8%	2001-03	1.1%	0.9%	[0.1, 1.7%]
Probs. washing/dressing	1994-96	3.4%	2011-14	0.6%	0.3%	[-0.2, 0.9%]
Any self-care limitation	1994-96	3.9%	2001-03	0.8%	0.7%	[0.1, 1.3%]
**Musculoskeletal/pain**						
Pain-any	1994-96	32.0%	2011-14	-2.2%	-3.3%	[-4.6, -2.0%]
Pain-extreme	1994-96	3.0%	2011-14	0.4%	0.2%	[-0.3, 0.7%]
Arthritis LSI[b]	1994-96	5.3%	2011-14	-0.3%	-0.7%	[-1.4, 0.0%]
Other musculoskeletal LSI[b]	1994-96	9.7%	2011-14	-0.5%	-0.8%	[-1.7, 0.1%]

[a] 'Adj.' = adjusted for changing age and sex distribution of the working-age population. [b] LSI=longstanding illness; see Table 1.

Other measures

Changes over time in other measures are shown in Table 5 below. This includes four biomarkers that are more difficult to compare directly to self-reports:

For peer review only - http://bmjopen.bmj.com/site/about/guidelines.xhtml

- Changes over time are available for two biomarkers of inflammation (C-reactive protein ('CRP') and fibrinogen). These are associated with a number of conditions including heart disease, diabetes, cancer [81] and – in the case of CRP – even depression.[82] Table 5 shows that both biomarkers have rising morbidity from 1997-2000 to 2008-10 (although for CRP, the confidence interval is wide and there is a non-negligible possibility that the change is negative).
- The two other biomarkers available in HSE are clearly focussed on anaemia and iron deficiency. Table 5 shows that both of these have declined, with particularly clear evidence for a decline in iron deficiency.

Table 5: Changes over time in other morbidity measures

	Starting period		Change from start to end period			
	Period	Prevalence	End period	Raw Change	Adj.[a] change	Adj. change 95% CI
**Other biomarkers**						
Raised C-reactive protein	1997-2000	21.4%	2008-10	2.1%	1.9%	[-0.7, 4.5%]
Raised fibrinogen	1997-2000	2.3%	2008-10	1.6%	1.5%	[0.3, 2.6%]
Anaemia	1994-96	6.7%	2008-10	-1.4%	-1.4%	[-2.7, -0.1%]
Iron deficiency	1994-96	39.9%	2008-10	-12.9%	-12.5%	[-14.8, -10.2%]
**Sensory & communication**						
LSI Eye or Ear[b]	1994-96	2.8%	2011-14	-0.9%	-1.0%	[-1.5, -0.6%]
Hearing limitation	1994-96	4.3%	2001-03	-1.5%	-1.6%	[-2.1, -1.0%]
Seeing limitation	1994-96	1.4%	2001-03	-0.2%	-0.2%	[-0.6, 0.1%]
Communicating limitation	1994-96	1.0%	2001-03	0.1%	0.1%	[-0.2, 0.4%]

[a] 'Adj.' = adjusted for changing age and sex distribution of the working-age population. [b] LSI=longstanding illness; see Table 1.

Table 5 also shows changes over time in sensory and communication-related morbidity. This shows a fall in eye/ear conditions (1994-6 to 2011-14) as well as

For peer review only - http://bmjopen.bmj.com/site/about/guidelines.xhtml

hearing limitations in the earlier period (1994-6 to 2001-03), but no change in people having difficulty communicating with others.

DISCUSSION

Despite considerable evidence on morbidity trends among older people, there are few published studies on changes in morbidity among the working-age population, particularly outside the USA. In this paper, we have analysed changes over time in working-age morbidity in England 1994-2014 using a high-quality repeated cross-sectional study. We see improvements in cardiovascular morbidity, respiratory morbidity and anaemia, but deteriorating obesity, diabetes, some biomarkers (fibrinogen and possibly also CRP) and feelings of extreme anxiety/depression. We see little systematic change over time in more common mental ill-health or musculoskeletal conditions, pain/mobility, and self-care limitations. Symptomatology and chronic disease diagnoses also often go in different directions – chronic disease diagnoses have sometimes stayed stable or even risen at the same time that underlying symptomatology has declined (such as for mental health conditions, asthma, hypertension, and CVD as a whole), mirroring findings at older ages.[3]

Our analysis has several strengths. We include every morbidity measure for which consistent changes can be constructed, including chronic disease, functioning and symptomatology, and biomarkers. We use a single survey series collected by a single survey organisation; exclude under-25s for whom comparability of survey coverage is unlikely; and construct new non-response weights. Nevertheless, we must note three

For peer review only - http://bmjopen.bmj.com/site/about/guidelines.xhtml

For peer review only

limitations. Firstly, response rates for each stage of the HSE have declined over time (see Web Appendix 3), and while we create new non-response weights covering the entire period, it is still possible that socioeconomically disadvantaged people (within any age-sex-region group) have become less likely to respond – and as they tend to be in worse health, this could mask deteriorating morbidity. Secondly, even if non-response biases have not changed, it is possible that people respond differently over time even to identical questions. Third, there are several dimensions of morbidity for which there is little comparable data in HSE. This includes several areas in which morbidity among the working-age population seems to be rising, including *inter alia* cognitive complaints,[83] allergic disorders,[84] and liver cirrhosis (see Web Appendix 1), as well as some areas in which morbidity seems likely to have fallen, such as chronic kidney disease.[85]

It is clear that there are different trends in different dimensions of morbidity – but for policymakers, this leaves the question of whether working-age morbidity as a whole is unchanged (H2), getting better (H1) or getting worse (H3), to the extent that it makes sense to place health on a unidimensional scale. While we cannot create a single morbidity index here, Web Appendix 9 shows the association of each measure with bad general self-rated health (net of age, gender and education). This shows little systematic trend for falling morbidity to be seen in the measures that predict health the most (indeed, the evidence weakly points in the other direction, towards

For peer review only - http://bmjopen.bmj.com/site/about/guidelines.xhtml

For peer review only

rising morbidity). This provides greater support for H2 than H1 or H3, mirroring evidence from the Global Burden of Disease study (see Web Appendix 9).

In conclusion, despite considerable falls in working-age mortality and gains in life expectancy – and the ensuing expectations of social security policymakers for improving morbidity – there is no evidence of systematic improvement in overall working-age morbidity in England from 1994 to 2014. However, two pieces of further research could strengthen this evidence base. Firstly, the ideal measures for analysing changes in morbidity are functional limitations measures, which are included in the HSE from 1996. However, these were last asked to the working-age population in 2001, and it is a priority to repeat these measures in future years of HSE. Secondly, there is a surprising paucity of studies looking at the changing morbidity of the working-age population outside the US. Given their importance in public debate – particularly in discussions of retirement ages and disability benefits – we hope that other authors will repeat and extend our analyses here, including disaggregating these changes across different regions and sociodemographic groups.

For peer review only - http://bmjopen.bmj.com/site/about/guidelines.xhtml

REFERENCES

1. Freedman VA, Crimmins E, Schoeni RF, et al. Resolving inconsistencies in trends in old-age disability: report from a technical working group. *Demography* 2004;41(3):417-41.
2. Freedman VA, Spillman BC, Andreski PM, et al. Trends in Late-Life Activity Limitations in the United States: An Update From Five National Surveys. *Demography* 2013;50(2):661-71. doi: 10.1007/s13524-012-0167-z
3. Freedman VA, Schoeni RF, Martin LG, et al. Chronic Conditions and the Decline in Late-Life Disability. *Demography* 2007;44(3):459-77.
4. Lafortune G, Balestat G. Trends in severe disability among elderly people. Paris: OECD, 2007.
5. Bowling A. Commentary: Trends in activity limitation. *International Journal of Epidemiology* 2011;40(4):1068-70. doi: 10.1093/ije/dyr069
6. Jagger C. Trends in life expectancy and healthy life expectancy. Future of an ageing population: evidence review. London: Foresight, Government Office for Science, 2015.
7. Office for Budget Responsibility. Welfare trends report: October 2016. Cm 9341. London: Her Majesty's Stationery Office, 2016.
8. Department for Work and Pensions, Department of Health. Improving Lives: The Work, Health and Disability Green Paper. Cm 9342. London: Her Majesty's Stationery Office, 2016.
9. Baumberg B, Jones M, Wass V. Disability prevalence and disability-related employment gaps in the UK 1998–2012: Different trends in different surveys? *Social Science & Medicine* 2015;141:72-81. doi: http://dx.doi.org/10.1016/j.socscimed.2015.07.012
10. Salomon JA, Nordhagen S, Oza S, et al. Aer Americans feeling less healthy? The puzzle of trends in self-rated health. *American Journal of Epidemiology* 2009;170(3):343-51.
11. OECD. Sickness, Disability and Work: Breaking the Barriers, Vol 2: Australia, Luxembourg, Spain and the United Kingdom Paris: OECD, 2008.
12. Wise DA, editor. *Social Security Programs and Retirement Around the World: The Capacity to Work at Older Ages*. Chicago: University of Chicago Press for the National Bureau of Economic Research (NBER), 2017.
13. Martin LG, Freedman VA, Schoeni RF, et al. Trends In Disability And Related Chronic Conditions Among People Ages Fifty To Sixty-Four. *Health Affairs* 2010;29(4):725-31. doi: 10.1377/hlthaff.2008.0746
14. Bhattacharya J, Choudhry K, Lakdawalla D. Chronic Disease and Severe Disability among Working-Age Populations. *Medical Care* 2008;46(1):92-100.
15. Martin LG, Schoeni RF. Trends in disability and related chronic conditions among the forty-and-over population: 1997-2010. *Disability and Health Journal* 2014;7(1):S4-S14. doi: 10.1016/j.dhjo.2013.06.007
16. Lakdawalla DN, Bhattacharya J, Goldman DP. Are the young becoming more disabled? *Health affairs* 2004;23(1):168-76.
17. Seeman TE, Merkin SS, Crimmins EM, et al. Disability Trends Among Older Americans: National Health and Nutrition Examination Surveys, 1988–1994 and 1999–2004. *American Journal of Public Health* 2010;100(1):100-07. doi: 10.2105/AJPH.2008.157388
18. Weir D. Are baby boomers living well longer. In: Madrian B, Mitchell OS, Soldo BJ, eds. Redefining retirement: How will boomers fare. Oxford: Oxford UP 2007:95-111.

For peer review only

For peer review only - http://bmjopen.bmj.com/site/about/guidelines.xhtml

19. van Oostrom SH, Gijsen R, Stirbu I, et al. Time Trends in Prevalence of Chronic Diseases and Multimorbidity Not Only due to Aging: Data from General Practices and Health Surveys. *PLoS ONE* 2016;11(8):e0160264. doi: 10.1371/journal.pone.0160264
20. Solé-Auró A, Alcañiz M. Are we living longer but less healthy? Trends in mortality and morbidity in Catalonia (Spain), 1994–2011. *European Journal of Ageing* 2015;12(1):61-70. doi: 10.1007/s10433-014-0317-9
21. Audureau E, Rican S, Coste J. Worsening trends and increasing disparities in health-related quality of life: evidence from two French population-based cross-sectional surveys, 1995–2003. *Quality of Life Research* 2013;22(1):13-26. doi: 10.1007/s11136-012-0117-7
22. Clause-Verdreau A-C, Audureau É, Leplège A, et al. Contrasted trends in health-related quality of life across gender, age categories and work status in France, 1995–2016: repeated population-based cross-sectional surveys using the SF-36. *Journal of Epidemiology and Community Health* 2019;73(1):65-72. doi: 10.1136/jech-2018-210941
23. Marfeo EE, Haley SM, Jette AM, et al. Conceptual Foundation for Measures of Physical Function and Behavioral Health Function for Social Security Work Disability Evaluation. *Archives of Physical Medicine and Rehabilitation* 2013;94(9):1645-52.e2. doi: 10.1016/j.apmr.2013.03.015
24. Martin LG, Schoeni RF, Andreski PM. Trends in health of older adults in the United States: past, present, future. *Demography* 2010;47(Suppl):S17-S40.
25. von Elm E, Altman D, Egger M, et al. The Strengthening the Reporting of Observational Studies in Epidemiology (STROBE) Statement: guidelines for reporting observational studies. *Annals of Internal Medicine* 2007;147:573-77.
26. Mindell J, Biddulph JP, Hirani V, et al. Cohort Profile: The Health Survey for England. *International Journal of Epidemiology* 2012;41(6):1585-93. doi: 10.1093/ije/dyr199
27. NatCen Social Research, University College London Department of Epidemiology and Public Health. Health Survey for England, 2014. [computer file], 1st Edition. UK Data Archive, SN: 7919, 2016. http://dx.doi.org/10.5255/UKDA-SN-7919-1
28. NatCen Social Research, University College London Department of Epidemiology and Public Health. Health Survey for England, 2013. [computer file], 1st Edition. UK Data Archive, SN: 7649, 2015. http://dx.doi.org/10.5255/UKDA-SN-7649-1
29. NatCen Social Research, University College London Department of Epidemiology and Public Health. Health Survey for England, 2012. [computer file], 1st Edition. UK Data Archive, SN: 7480, 2014. http://dx.doi.org/10.5255/UKDA-SN-7480-1
30. NatCen Social Research, University College London Department of Epidemiology and Public Health. Health Survey for England, 2011. [computer file], 1st Edition. UK Data Archive, SN: 7260, 2013. http://dx.doi.org/10.5255/UKDA-SN-7260-1
31. NatCen Social Research, Royal Free and University College Medical School Department of Epidemiology and Public Health. Health Survey for England, 2010. [computer file], 2nd Edition. UK Data Archive, SN: 6986, 2012. http://dx.doi.org/10.5255/UKDA-SN-6986-2
32. National Centre for Social Research, University College London Department of Epidemiology and Public Health. Health Survey for England, 2009. [computer file], 2nd Edition. UK Data Archive, SN: 6732, 2011. http://dx.doi.org/10.5255/UKDA-SN-6732-1

For peer review only - http://bmjopen.bmj.com/site/about/guidelines.xhtml

33. National Centre for Social Research, University College London Department of Epidemiology and Public Health. Health Survey for England, 2008. [computer file], 3rd Edition. UK Data Archive, SN: 6397, 2011. http://dx.doi.org/10.5255/UKDA-SN-6397-1
34. National Centre for Social Research, University College London Department of Epidemiology and Public Health. Health Survey for England, 2007. [computer file], 2nd Edition. UK Data Archive, SN: 6112, 2010. http://dx.doi.org/10.5255/UKDA-SN-6112-1
35. National Centre for Social Research, University College London Department of Epidemiology and Public Health. Health Survey for England, 2006. [computer file], 2nd Edition. UK Data Archive, SN: 4150, 2008.
36. National Centre for Social Research, University College London Department of Epidemiology and Public Health. Health Survey for England, 2005. [computer file], 3rd Edition. UK Data Archive, SN: 5675, 2011. http://dx.doi.org/10.5255/UKDA-SN-5675-1
37. National Centre for Social Research, University College London Department of Epidemiology and Public Health. Health Survey for England, 2004. [computer file], 2nd Edition. UK Data Archive, SN: 5439, 2010. http://dx.doi.org/10.5255/UKDA-SN-5439-1
38. National Centre for Social Research, University College London Department of Epidemiology and Public Health. Health Survey for England, 2003. [computer file], 2nd Edition. UK Data Archive, SN: 5098, 2010. http://dx.doi.org/10.5255/UKDA-SN-5098-1
39. National Centre for Social Research, University College London Department of Epidemiology and Public Health. Health Survey for England, 2002. [computer file], 2nd Edition. UK Data Archive, SN: 4912, 2010. http://dx.doi.org/10.5255/UKDA-SN-4912-1
40. National Centre for Social Research, University College London Department of Epidemiology and Public Health. Health Survey for England, 2001. [computer file], 3rd Edition. UK Data Archive, SN: 4628, 2010. http://dx.doi.org/10.5255/UKDA-SN-4628-1
41. National Centre for Social Research, University College London Department of Epidemiology and Public Health. Health Survey for England, 2000. [computer file], 4th Edition. UK Data Archive, SN: 4487, 2011. http://dx.doi.org/10.5255/UKDA-SN-4487-1
42. National Centre for Social Research, University College London Department of Epidemiology and Public Health. Health Survey for England, 1999. [computer file], 3rd Edition. UK Data Archive, SN: 4365, 2002.
43. National Centre for Social Research, University College London Department of Epidemiology and Public Health. Health Survey for England, 1998. [computer file], 5th Edition. UK Data Archive, SN: 4150, 2010. http://dx.doi.org/10.5255/UKDA-SN-4150-1
44. Joint Health Surveys Unit of Social and Community Planning Research, University College London Department of Epidemiology and Public Health. Health Survey for England, 1997. [data collection], 4th Edition. UK Data Service, SN: 3979, 2017. http://doi.org/10.5255/UKDA-SN-3979-2
45. Joint Health Surveys Unit of Social and Community Planning Research, University College London Department of Epidemiology and Public Health. Health Survey for England, 1996. [data collection], 5th Edition. UK Data Service, SN: 3886, 2017. http://doi.org/10.5255/UKDA-SN-3886-2
46. Joint Health Surveys Unit of Social and Community Planning Research, University College London Department of Epidemiology and Public Health. Health Survey for England,

For peer review only - http://bmjopen.bmj.com/site/about/guidelines.xhtml

1995. [data collection], 5th Edition. UK Data Service, SN: 3796, 2017. http://doi.org/10.5255/UKDA-SN-3796-2
47. Joint Health Surveys Unit of Social and Community Planning Research, University College London Department of Epidemiology and Public Health. Health Survey for England, 1994. [data collection], 5th Edition. UK Data Service, SN: 3640, 2017. http://doi.org/10.5255/UKDA-SN-3640-2
48. Sperrin M, Marshall A, Higgins V, et al. Slowing down of adult body mass index trend increases in England: a latent class analysis of cross-sectional surveys (1992-2010). *International Journal of Obesity* 2014;38(6)
49. Geiger BB. Morbidity in England 1994-2014 2019 [Available from:http://osf.io/dy6sv].
50. Quesnel Vallee A. Self-rated health: caught in the crossfire of the quest for 'true' health? *International Journal of Epidemiology* 2007;36:1161-64.
51. Groves RM, Fowler FJ, Couper MP, et al. Survey Methodology [2e]. Hoboken, NJ: Wiley 2009.
52. Sprangers MA, Schwartz CE. Integrating response shift into health-related quality of life research: a theoretical model *Social Science & Medicine* 1999;48(11):1507-151.
53. O'Brien R. Disability and the Worlds of Welfare Capitalism. *Sociological Science* 2015;2(1):1-19. doi: 10.15195/v2.a1
54. OECD. Sick on the Job? Myths and Realities about Mental Health and Work. Paris: OECD, 2012.
55. Cieza A, Oberhauser C, Bickenbach J, et al. The English are healthier than the Americans: really? *International Journal of Epidemiology* 2015:229-38. doi: 10.1093/ije/dyu182
56. Lawlor DA, Adamson J, Ebrahim S. Performance of the WHO Rose angina questionnaire in post-menopausal women: Are all of the questions necessary? *Journal of Epidemiology and Community Health* 2003;57(7):538-41. doi: 10.1136/jech.57.7.538
57. Cook D, Shaper A, Macfarlane P. Using the WHO (Rose) Angina Questionnaire in Cardiovascular Epidemiology. *International Journal of Epidemiology* 1989;18(3):607-13. doi: 10.1093/ije/18.3.607
58. Goldberg D, PA W. User Guide to the General Health Questionnaire. Windsor, UK: NFER-Nelson, 1988.
59. Mont D. Measuring disability prevalence: World Bank 2007.
60. Burgard SA, Chen PV. Challenges of health measurement in studies of health disparities. *Social science & medicine (1982)* 2014;106:143-50. doi: 10.1016/j.socscimed.2014.01.045 [published Online First: 01/31]
61. Altman BM, editor. *International Measurement of Disability: Purpose, Method and Application*: Springer, 2016.
62. Groce NE, Mont D. Counting disability: emerging consensus on the Washington Group questionnaire. *The Lancet Global Health* 2017;5(7):e649-e50. doi: 10.1016/S2214-109X(17)30207-3
63. Chan KS, Kasper JD, Brandt J, et al. Measurement Equivalence in ADL and IADL Difficulty Across International Surveys of Aging: Findings From the HRS, SHARE, and ELSA. *The Journals of Gerontology Series B: Psychological Sciences and Social Sciences* 2012;67B(1):121-32. doi: 10.1093/geronb/gbr133
64. Bago d'Uva T, O'Donnell O, van Doorslaer E. Differential health reporting by education level and its impact on the measurement of health inequalities among older Europeans. *International Journal of Epidemiology* 2008;37:1375-83. doi: 10.1093/ije/dyn146

For peer review only - http://bmjopen.bmj.com/site/about/guidelines.xhtml

65. Benzeval M, Kumari M, Jones AM. How Do Biomarkers and Genetics Contribute to Understanding Society? *Health Economics* 2016;25(10):1219-22. doi: 10.1002/hec.3400
66. Martin LG, Freedman VA, Schoeni RF, et al. Health and Functioning Among Baby Boomers Approaching 60. *The Journals of Gerontology Series B: Psychological Sciences and Social Sciences* 2009;64B(3):369-77. doi: 10.1093/geronb/gbn040
67. Kaye HS. Disability Rates For Working-Age Adults And For The Elderly Have Stabilized, But Trends For Each Mean Different Results For Costs. *Health Affairs* 2013;32(1):127-34. doi: 10.1377/hlthaff.2012.0533
68. Mood C. Logistic Regression: Why We Cannot Do What We Think We Can Do, and What We Can Do About It. *European Sociological Review* 2010;26(1):67-82.
69. Wasserstein RL, Lazar NA. The ASA's Statement on p-Values: Context, Process, and Purpose. *The American Statistician* 2016;70(2):129-33. doi: 10.1080/00031305.2016.1154108
70. Unal B, Critchley JA, Capewell S. Explaining the Decline in Coronary Heart Disease Mortality in England and Wales Between 1981 and 2000. *Circulation* 2004;109(9):1101-07. doi: 10.1161/01.cir.0000118498.35499.b2
71. Davies AR, Smeeth L, Grundy E. Contribution of changes in incidence and mortality to trends in the prevalence of coronary heart disease in the UK: 1996–2005. *European Heart Journal* 2007;28(17):2142-47.
72. Oyebode O. Cardiovascular disease. In: Craig R, Mindell J, eds. Health Survey for England, 2011, Volume 1: Health, social care and lifestyles: TSO 2012:21-62.
73. Davillas A, Benzeval M. Alternative measures to BMI: Exploring income-related inequalities in adiposity in Great Britain. *Social Science & Medicine* 2016;166:223-32. doi: http://dx.doi.org/10.1016/j.socscimed.2016.08.032
74. Read SH, Kerssens JJ, McAllister DA, et al. Trends in type 2 diabetes incidence and mortality in Scotland between 2004 and 2013. *Diabetologia* 2016;59(10):2106-13. doi: 10.1007/s00125-016-4054-9
75. McManus S, Bebbington P, Jenkins R, et al., editors. *Mental health and wellbeing in England: Adult Psychiatric Morbidity Survey 2014*. Leeds: NHS Digital, 2016.
76. Ross A, Kelly Y, Sacker A. Time trends in mental well-being: the polarisation of young people's psychological distress. *Social Psychiatry and Psychiatric Epidemiology* 2017;52(9):1147-58. doi: 10.1007/s00127-017-1419-4
77. Gili M, Roca M, Basu S, et al. The mental health risks of economic crisis in Spain: evidence from primary care centres, 2006 and 2010. *European Journal of Public Health* 2012;23(1):103-08. doi: 10.1093/eurpub/cks035
78. McBeth J, Jones K. Epidemiology of chronic musculoskeletal pain. *Best Practice & Research Clinical Rheumatology* 2007;21(3):403-25. doi: 10.1016/j.berh.2007.03.003
79. Harkness EF, Macfarlane GJ, Silman AJ, et al. Is musculoskeletal pain more common now than 40 years ago?: two population-based cross-sectional studies. *Rheumatology* 2005;44(7):890-95. doi: 10.1093/rheumatology/keh599
80. Palmer KT, Walsh K, Bendall H, et al. Back pain in Britain: comparison of two prevalence surveys at an interval of 10 years. *BMJ* 2000;320:1577-8.
81. Chaudhury M. Blood analytes. In: Sproston K, Primatesta P, eds. Health Survey for England, 2003, Vol 2: Risk Factors for Cardiovascular Disease: TSO 2004:241-88.

For peer review only - http://bmjopen.bmj.com/site/about/guidelines.xhtml

82. Steptoe A. Psychosocial biomarker research: integrating social, emotional and economic factors into population studies of aging and health. *Social Cognitive and Affective Neuroscience* 2011;6(2):226-33. doi: 10.1093/scan/nsq032

83. Begum A, Dewey M, Hassiotis A, et al. Subjective cognitive complaints across the adult life span: a 14-year analysis of trends and associations using the 1993, 2000 and 2007 English Psychiatric Morbidity Surveys. *Psychological Medicine* 2014;44(9):1977-87. doi: 10.1017/S0033291713002444

84. Gupta R, Sheikh A, Strachan DP, et al. Time trends in allergic disorders in the UK. *Thorax* 2007;62(1):91-96. doi: 10.1136/thx.2004.038844

85. Aitken GR, Roderick PJ, Fraser S, et al. Change in prevalence of chronic kidney disease in England over time: comparison of nationally representative cross-sectional surveys from 2003 to 2010. *BMJ Open* 2014;4(9) doi: 10.1136/bmjopen-2014-005480

For peer review only

For peer review only - http://bmjopen.bmj.com/site/about/guidelines.xhtml

WEB APPENDICES

Table of contents (pages refer to within web appendices)

Appendix 1: Working-age mortality trends 1

Appendix 2: Overall missingness in health measures 2

Appendix 3: Changing non-response & weights 4

Appendix 4: General self-reported health/disability 8

Appendix 5: Health measures 10

Appendix 6: Measures not included in the main paper 23

Appendix 7: Year-by-year trends 30

Appendix 8: Others' analyses over change over time using HSE data 34

Appendix 9: Summarising multiple measures 36

Bibliography for Web Appendices 41

For peer review only

Appendix 1: Working-age mortality trends

Mortality in general

Given debates about whether historic improvements in life expectancy are being sustained, particularly in the US and UK, [1 2] it is important to note that in the period under study in this paper, working-age life expectancy was increasing. This can be seen in data from the Human Mortality Database (May 2016 update) 1993-2013, using one-year age and one-year period. This data shows that increases in mortality are not found for working-age people as a whole in any major country – for example, standardised working-age death rates have declined by 23% in the US and 35% in the UK over 1993-2013.

Cause-specific mortality for the 0-64 population

The main text refers to cause-specific morality in several places, referring to the death rate among 064 year olds from cardiovascular disease (CVD), respiratory conditions, diabetes, and liver cirrhosis. These death rates refer to UK deaths within relevant ICD-10 codes (I00-I99 for CVD, J00-J99 for respiratory conditions, E10-E14 for diabetes), standardised to the European standard population, and taken from the World Health Organization European Office's Health for All Database (May 2016 version), http://www.euro.who.int/en/data-and-evidence/databases/european-health-for-all-databasehfa-db.

For peer review only - http://bmjopen.bmj.com/site/about/guidelines.xhtml

Appendix 2: Overall missingness in health measures

This appendix refers to overall item-level missi

ngness; changing item- and unit-level missingness is covered in Appendix 3.

Interview measures

For those who took part in the initial face-to-face interview, the level of item missingness is shown below (including only those years in which each question was asked). This shows the itemmissingness is generally very low – only 1 of the 30 measures variables have item-missingness greater than 1%.

Table 1: Missingness at the initial face-to-face interview

	n non-missing	*n missing*	*% missingness*
BMI	124,682	15,415	11.0%
Any recent CVD	43,274	354	0.8%
Recent high blood pressure	43,366	262	0.6%
Breathlessness-Grade 2+	25,620	68	0.3%
Breathlessness-Grade 3	25,620	68	0.3%
Recent heart attack/stroke	43,519	109	0.3%
COPD symptoms	25,631	57	0.2%
Recent angina	43,551	77	0.2%
Heart attack symptoms	43,595	33	0.1%
Angina symptoms	43,592	36	0.1%
Recent diabetes	66,637	54	0.1%
Mini stroke (TIA) symptoms	23,487	16	0.1%
Diagnosed asthma	41,225	28	0.1%
Wheezing stopping sleep	41,224	29	0.1%
Recent wheezing/asthma	41,224	29	0.1%
Locomotor limitation	25,347	10	0.0%
Self-care limitation	25,347	10	0.0%
Limitations in past 2wks	140,041	56	0.0%
Longstanding illness (LSI)	124,906	43	0.0%
Limiting LSI (LLSI)	104,798	36	0.0%
Any CVD LSI	124,912	37	0.0%
IHD/stroke LSI	124,912	37	0.0%
Mental health LSI	124,912	37	0.0%
Arthritis LSI	124,912	37	0.0%
Asthma LSI	124,912	37	0.0%
Diabetes LSI	124,912	37	0.0%
High blood pressure LSI	124,912	37	0.0%
Other musculoskeletal LSI	124,912	37	0.0%
Good general health	140,048	49	0.0%
Bad general health	140,048	49	0.0%

The only variable with noticeable missingness is BMI, which is understandable as this involves the interviewer taking height and weight measurements rather than simply asking for a verbal response. There are various reasons why people do not have a BMI measurement:

For peer review only - http://bmjopen.bmj.com/site/about/guidelines.xhtml

- *High weight*: people with a very high weight are not weighed in HSE 'because the scales are inaccurate above this level', but the definition of this changed (from 130kg before 2011 to 200kg afterwards). This only applied to <0.1% of respondents 2012-14.
- *Difficult to take measurement*: other respondents (between 3.8% and 6.1% depending on the year) have no valid BMI measurement because height or weight measures were not attempted, attempted but not obtained or useable, because the respondent was pregnant, or the respondent was too sick or unsteady.
- *Refusal*: the most common reason for no BMI measurement is an outright refusal (including those refusing out of anxiety, though this tends to be a minor reason). Refusal rates are 8.3% in 2014.

Self-completion measures

For those who completed the self-completion booklet, the level of item missingness is shown in the table below.

Table 2: Missingness within the self-completion booklet

	n *non-missing*	*n* *missing*	% *missingness*
Psychological distress symptoms	108,324	2,462	2.2%
Problems washing/dressing today	62,703	1,310	2.1%
Anxiety/depression	62,725	1,288	2.0%
Problems w/activities	62,742	1,271	2.0%
Problems walking about today	62,772	1,241	1.9%
Pain	62,783	1,230	1.9%

Item missingness is relatively low compared to missingness from not completing the self-completion survey (51.5% of respondents in 2014).

Nurse visit measures

For those who took part in the nurse visit, the level of item missingness is shown in the table below.

Table 3: Missingness within the nurse visit

	n *non-missing*	*n* *missing*	% *missingness*
Biomarker high blood pressure	87,726	15,517	15.0%
High waist-hip ratio	78,637	2,664	3.3%

This shows that far more people have missing observations for measured high blood pressure than for their waist-hip ratio. This is despite the fact that we explicitly INCLUDE those who are on blood pressure-lowering drugs (about 5% of the sample at the start of the period and 10% at the end), on the grounds that their lowered blood pressure still conveys useful information about their health state. The main reason for the remaining high level of missingness is because people have recently exercised, smoked, drank or ate (12.2%).

Blood sample measures

For those from whom a blood sample was taken, the level of item missingness is shown in the table below.

For peer review only - http://bmjopen.bmj.com/site/about/guidelines.xhtml

Table 4: Missingness within the blood sample

	n *non-missing*	*n* *missing*	*%* *missingness*
Raised fibrinogen	16,166	3,341	17.1%
Raised C-reactive protein	17,814	1,693	8.7%
Glycated haemoglobin	28,810	1,436	4.8%
Anaemia	20,302	939	4.4%
Iron deficiency	20,375	866	4.1%
Low HDL cholesterol	36,076	1,406	3.8%
High total cholesterol	43,409	1,472	3.3%

All of these measures are affected by problems in transferring and storing the blood sample and with the measurement process, which results in problems with 3-10% of the blood samples depending on the measure and year. As for blood pressure, we explicitly INCLUDE those who are on lipidlowering drugs (0.4% 1994 to 7.9% 2014), on the grounds that their changed cholesterol level still conveys useful information about their health state. Item missingness is highest for fibrinogen, which not only has high rates of such failures (7.0-9.5%), but also has ineligibility due to likely infection (from raised CRP, 3.6-5.6% of those with blood samples) and taking drugs that affect the reading (3.7% to 7.7% dependent on the year). Item missingness is also high for C-reactive protein (CRP), which also excludes those with likely infections.

Dealing with item-level missingness

Because of the high level of item non-response for certain measures (BMI, high blood pressure, fibrinogen, and CRP), and moderate level for others (other blood sample biomarkers and waist-hip ratio) – and because of evidence of changing non-response at various stages of the survey process – non-response weights were created to try to correct for any biases that these introduce. This is described in further detail in Appendix 3.

Appendix 3: Changing non-response & weights

This appendix focuses on *changes* in unit-level non-response at different stages of HSE.

Changing non-response

Sample frame coverage

As noted in the main paper, HSE is a household sample that excludes those in communal establishments. If we combine data from the 1991, 2001 and 2011 Censuses,[1] the communal population is as follows:

Table 1: Population in communal establishments over time (all working-age) and by age (in 2011)

		Education	Medical/ care	Defence	Prison	Other / not stated
**All working age**	*1991*	21,149	86,683	44,562	13,279	63,340
	2001	204,606	73,705	46,428	44,185	86,288

For peer review only - http://bmjopen.bmj.com/site/about/guidelines.xhtml

	2011	328,772	76,026	41,659	47,849	61,124
**16-24**	*2011*	305,154	9,346	22,677	12,607	25,673
**25-34**	*2011*	20,443	12,000	15,025	15,407	14,417
**35-49**	*2011*	2,663	26,796	3,725	14,725	14,708
**50-SPA[1] (est)**	*2011*	512	27,884	232	5,110	6,326

1 SPA = State Pension Age, which is 60 for women and 65 for men. This is estimated because the Census totals are given for 50-64 year olds, so we have excluded 1/3 of women aged 50-64 from these totals.

This shows two things. Firstly, that there was a sharp rise in the working-age population in communal establishments 1991-2001 (from 230k to 560k), which was concentrated (>90% of the rise) among education-related communal establishments – although this is perhaps a slight overestimate given a definition change in the Census data.[2] Secondly, looking at education-related communal establishments in 2011, these are overwhelmingly (>90%) among 16-24 year olds. It therefore seems likely that the exclusion of communal establishments in HSE will lead to biases in young adults, and we therefore exclude 16-24 year olds from the trend analyses.

Changing unit non-response within the sample frame

As noted in the main paper, HSE supplies non-response weights from 2003, including adjustments for non-response to the nurse visit and blood sample using health and socioeconomic status from the initial interview. However, there had been a substantial decline in response rates prior to 2003, as shown in the table below:

Table 2: Response rates to HSE

	Household	Individual	Self-comp.	BMI	Nurse	Blood
1991	85.3%	81.1%				
1992	81.8%	77.4%				
1993	80.8%	75.7%				
1994	77.4%	71.6%	71.2%	67.1%	63.3%	53.3%
1995	78.3%	72.9%	72.0%	66.8%	63.7%	
1996	79.4%	74.7%	73.7%	69.6%	66.1%	
1997	76.0%	71.1%	69.8%	66.9%	64.0%	
1998	74.0%	68.9%	66.7%	63.3%	59.6%	49.0%
1999	76.2%	70.3%	68.5%	63.6%		
2000	75.5%	68.4%	65.8%	60.5%	58.2%	

1 Data are obtained from nomis on 6/8/2015, from Census tables DC1104EW and DC4210EWla (2011), S126 (2011) and L03/L04/L05 (2001).

2 The guide to Census SARs notes, *"In the 1991 Census, students and schoolchildren were treated as usually resident at their 'home' or vacation address. In the 2001 census students and schoolchildren in full-time education studying away from the family home were enumerated as resident at their term-time address."* See https://census.ukdataservice.ac.uk/use-data/guides/microdata/comparability-91-01 [accessed 1/11/2016].

For peer review only - http://bmjopen.bmj.com/site/about/guidelines.xhtml

2001	74.2%	67.1%	64.5%	60.1%	54.2%	
2002	74 %	67 %	64.4%	59.6%	54.3%	
2003	72.7%	66.4%	64.1%	59.7%	52.2%	39.9%
2004	72.4%	65.6%	62.4%	56.1%		
2005	71.4%	64.1%	60.6%	54.8%	46.7%	
2006	68.1%	60.5%	57.7%	52.8%	45.4%	34.7%
2007	65.7%	58.3%	56.1%	51.3%	42.6%	
2008	64.5%	57.9%	55.9%	50.0%	41.5%	30.4%
2009	67.6%	61.0%	58.7%	52.5%	43.1%	33.7%
2010	66.1%	58.7%	54.9%	49.3%	39.1%	29.9%
2011	65.7%	58.9%	54.3%	49.0%	39.4%	29.8%
2012	64.1%	56.3%	52.5%	47.4%	36.3%	27.9%
2013	63.8%	57.6%	54.2%	49.3%	40.1%	31.2%
2014	61.6%	55.5%	51.5%	48.4%	37.3%	28.7%

In general these trends are due to increases in refusal rates. However, the blood sample response rate is affected by two noticeable changes in eligibility over this period (people who are pregnant or who had blood/clotting disorders were ineligible throughout):

1. In 1998, people who had ever had an epileptic fit were excluded from the blood sample. This raised the ineligibility rate to 3.5% of the sample in 1998, from 0.6% in 1994.
2. In 2010, this was then relaxed so that those who had had an epileptic fit more than 5 years ago were again included in the blood sample. This lowered the ineligibility rate from 3.1% in 2009 to 2.4% in 2010.

Changing item non-response within responding people

There are also changes over time in item non-response (further detail on overall item non-response is given in Appendix 2). This includes:

- *BMI:* there has been little systematic trend in one reason for the absence of a BMI measure (difficulty in taking BMI measurements). However, there are trends in other reasons:
  - *High weight*: the definition of high weight changed from 130kg before 2011 to 200kg afterwards. 1.0% of respondents were not weighted for this reason in 2010, which fell to <0.1% 2012-14.
  - *Refusal*: in line with the general participation rates at each stage of the interview above, BMI refusal rates rose sharply from 1.9% in 1994 to a peak of 11.5% in 2011, and remain at 8.3% in the 2014 data.

For peer review only - http://bmjopen.bmj.com/site/about/guidelines.xhtml

- *Psychological distress:* similarly to wider participation rates at each stage of the survey, item missingness within the self-completion survey does increase over time (e.g. for psychological distress symptoms, from 1.8% 1994 to 5.9% 2014).
- *Measured high blood pressure:* there was a noticeable rise over time in exclusion of high blood pressure measures on the grounds that people recently exercised, smoked, drank or ate (from 6.1% to 13.6%).
- *Fibrinogen:* taking drugs that affect the fibrinogen reading rose from 3.7% 1994 to 7.7% 2009.

Creating non-response weights

To increase comparability over time, we create new weights 1994-2014 in several phases.

First-stage non-response weights

Firstly, we created a selection weight because some households were slightly more likely to be interviewed than others. (Until 2009, only three households at each address were interviewed. Those living at addresses with many households are therefore less likely to be interviewed). NatCen supplied selection weights for 2004-2013 to enable this (funded by this project), which are not available on the public HSE datasets.

Secondly, after adjusting for the selection weight, we created new individual-level (inverse probability) weights to match population age-sex-region totals in each year. Population data are annual mid-year population estimates from nomis. NatCen added the region variable for the 19941997 datasets to the public HSE datasets to enable this.

Second-stage non-response weights

After the first-stage adjustment for individual non-response, for the later stages of the interview (self-completion, BMI measurement, nurse visit, blood sample), we created a further weight that adjusts for non-response among those responding to the individual interview. This is based on a logit regression model to predict that stage of response based on:

- Age and gender (4 age group categories interacted with gender);
- Qualifications (degree or FT student / A-level or above / other qualifications / no qualifications);
- Household type (presence of other adults in the household);
- Employment status (yes/no);
- Smoking (never regular smoker / ex-regular smoker / current regular smoker); and
- Self-reported general health (bad or very bad health vs. other categories).

On the basis of these criteria, we create inverse probability weights – that is, we create a predicted probability of response for each respondent based on the logit regression model, and then create a weight that is the inverse of this predicted probability. The revised weights are included in the Stata code to enable replication of the full paper.

Final sample size

The final sample size is as follows:

For peer review only - http://bmjopen.bmj.com/site/about/guidelines.xhtml

Table 3: HSE sample size in each year

	Interview	Self-completion	Nurse visit	Blood sample
1994	9,948	9,884	8,786	7,399
1995	10,167	10,049	8,881	
1996	10,401	10,269	9,206	
1997	5,563	5,458	5,005	
1998	10,177	9,843	8,805	7,236
1999	5,008	4,884		
2000	5,188	4,993	4,417	
2001	10,002	9,613	8,079	
2002	4,662	4,482	3,775	
2003	9,420	9,089	7,395	5,665
2004	4,165	3,961		
2005	4,810	4,548	3,505	
2006	8,825	8,420	6,622	5,064
2007	4,198	4,039	3,064	
2008	9,242	8,922	6,625	4,845
2009	2,795	2,689	1,973	1,542
2010	5,120	4,794	3,411	2,610
2011	5,258	4,853	3,518	2,667
2012	4,936	4,605	3,188	2,447
2013	5,303	4,992	3,691	2,875
2014	4,909	4,552	[redacted]	2,531
**Total**	**140,097**	**134,939**	[redacted]	**44,881**

Appendix 4: General self-reported health/disability

Trends in seven general health/disability measure are available in HSE:

Table 1: HSE general health measures

Measure	Operationalisation (years available)
Good general health	Health in general is 'good' or 'very good' (1994-2014)
Bad general health	Health in general is 'bad' or 'very bad' (1994-2014)
Longstanding illness (LSI)	Any long-standing illness, disability or infirmity (1994-2011)
Limiting LSI (LLSI)	LSI limits activities in any way (1996-2011)
Problems with activities-some	Some problems with performing usual activities (1996-2014)
Problems with activities-unable	Unable to perform usual activities (1996-2014)
Limitations in past 2wks	Cut down on activities in past 2wks due to LSI or other illness/injury (1994-2014)

See Web Appendix 5 for full details on all measures .

For peer review only - http://bmjopen.bmj.com/site/about/guidelines.xhtml

Trends for these measures are shown in Table 9 below. Looking first at good general health, the table shows the trend from 1994-6, when 80.9% reported good general health. By 2011-14, there had been a decline of 0.8 percentage points. When we adjust for the changing age and sex distribution of the working-age population (labelled 'Adj.' in Table 1), the decline is only 0.1%, with a wide confidence interval (-0.9 to +0.7%), and there is therefore little evidence for any systematic trend.

Table 2: Changes over time in general health

	Starting period		Change from start to end period			
	Period	Prevalence	End period	Raw change	Adj.[a] change	Adj. change 95% CI
Good general health	1994-96	80.9%	2011-14	-0.8%	-0.1%	[-0.9, 0.7%]
Bad general health	1994-96	4.4%	2011-14	1.3%	1.0%	[0.6, 1.5%]
Longstanding illness (LSI)	1994-96	36.2%	2011-14	-1.0%	-2.0%	[-3.7, -0.3%]
Limiting LSI (LLSI)	1994-96	21.4%	2011-14	-2.9%	-3.6%	[-5.2, -2.1%]
Problems w/activities-some	1994-96	14.8%	2011-14	-1.2%	-1.8%	[-2.8, -0.8%]
Problems w/activities-unable	1994-96	1.9%	2011-14	-0.6%	-0.8%	[-1.1, -0.4%]
Limitations in past 2wks	1994-96	14.7%	2011-14	-0.1%	-0.3%	[-1.0, 0.4%]

[a] 'Adj.' = adjusted for changing age and sex distribution of the working-age population.

For several of the general health measures, there is evidence of change over this period – but interpreting this is difficult, because the trends are in opposite directions. There is strong evidence for a *rise* in bad general health (a rise of 0.6-1.5% from a base of 4.4%), yet equally strong evidence for a *decline* in having problems with everyday activities (at both levels of severity), and being limited in activities by a longstanding illness. This shows the challenges in tracking population morbidity change through general, non-specific measures, which are likely to be as influenced by changes in reporting styles as much as changes in morbidity *per se*.

As an aside, UK Government publications have made claims based on healthy/disability-free life expectancy – sometimes using these to argue that morbidity has been improving [3], but more recently to argue that morbidity has been deteriorating.[4-6] However, these trends are potentially misleading: they include older people as well as the working-age population; they confuse a combined mortality-morbidity measure with morbidity; and they are based on self-reports of global health that are unreliable, as we show here and discuss in the main text.

For peer review only

For peer review only - http://bmjopen.bmj.com/site/about/guidelines.xhtml

Appendix 5: Health measures

We systematically searched HSE questions, and have included every morbidity measure that is comparable over a significant duration. We have excluded questions only available for short time frames (ADLs 2012-14, EQ-5D visual analogue scale 2008-14, SF-12 1996-2000, eczema/hayfever 1995-2001, breathlessness 1991-98 and 1995-2001, lung function 1995-2001, bladder limitations 1995-2001, LDL cholesterol, triglycerides and glucose 1999-2003, IgE 1996-2002 and an alternate measure of high blood pressure 2009-14), with the exception of five key measures of activity limitations 1995-2001. We have also excluded questions that are not direct measures of health (medication or health service use, demispan, health risk factors such as fractures, accidents, alcohol/tobacco use (including biomarkers), physical activity, and wellbeing).

Short summaries of the resulting 39 measures are given in this paper, and full details are given in the table below. Measures are taken from the initial face-to-face survey unless otherwise specified. The Stata code to create these variables in consistent form from the publicly available HSE files are available from OSF[7] and www.benbgeiger.co.uk.

Measure	Details
Activity limitations and MSDs	
Problems walking today	In the self-completion survey in 1996, 2003-6, 2008, 2010-12 and 2014, respondents were asked *'Now we would like to know how your health is today. Please answer ALL the questions. By ticking one box for each question below, please indicate which statements best describe your own health state today'*:<br>- *"I have no problems in walking about"*<br>- *"I have some problems in walking about"* - *"I am confined to bed"*<br>[This is part of the widely-used EQ-5D health status indicator [8]. However, for the purposes of this paper we have separated the individual measures that make up the EQ5D in order to compare these to similar indicators of morbidity within each domain].<br>People are classified as having a problem with self-care today if they had some problems walking about or were confined to bed.
Locomotor limitation	This is based on the personal care disability scale used in the 2001 HSE report [9]. Respondents in 1995, 2000 and 2001 were asked if any of the following applied to them (interviewers were instructed to ignore temporary disabilities that are expected to last less than one year):<br>- *"Cannot walk 200 yards or more on own without stopping or discomfort"*. People who reported a limitation were asked if they used a walking aid, and if they did, were then asked if they could walk 200 yards without the walking aid.<br>- *"Cannot walk up and down a flight of 12 stairs without resting"*<br>- *"Cannot bend down and pick up a shoe from the floor when standing"*<br>People are classified as having a locomotor limitation if they reported ANY of these limitations.
Problems with washing/dressing today	In the self-completion survey in 1996, 2003-6, 2008, 2010-12 and 2014, respondents were asked *'Now we would like to know how your health is today. Please answer ALL the questions. By ticking one box for each question below, please indicate which statements best describe your own health state today'*:<br>- *"I have no problems with self-care"*<br>- *"I have some problems washing or dressing myself"*<br>- *"I am unable to wash or dress myself"*

For peer review only - http://bmjopen.bmj.com/site/about/guidelines.xhtml

	[This is part of the widely-used EQ-5D health status indicator [8]. However, for the purposes of this paper we have separated the individual measures that make up the EQ-5D in order to compare these to similar indicators of morbidity within each domain].<br>People are classified as having a problem with self-care today if they had some problems washing/dressing or were unable to wash/dress themselves.
Self-care limitation	This is based on the personal care disability scale used in the 2001 HSE report [9]. Respondents in 1995, 2000 and 2001 were asked if any of the following applied to them (interviewers were instructed to ignore temporary disabilities that are expected to last less than one year):<br>- *"Cannot get in and out of bed on own without difficulty"*<br>- *"Cannot get in and out of a chair without difficulty"*<br>- *"Cannot dress and undress without difficulty"*<br>- *"Cannot wash hands and face without difficulty"*<br>- *"Cannot feed, including cutting up food without difficulty"*<br>- *"Cannot get to and use toilet on own without difficulty"*<br>People are classified as having a self-care limitation if they reported ANY of these limitations.
Pain *(any / extreme)*	In the self-completion survey in 1996, 2003-6, 2008, 2010-12 and 2014, respondents were asked *'Now we would like to know how your health is today. Please answer ALL the questions. By ticking one box for each question below, please indicate which statements best describe your own health state today'*:<br>- *"I have no pain or discomfort"*<br>- *"I have moderate pain or discomfort"*<br>- *"I have extreme pain or discomfort"*<br>[This is part of the widely-used EQ-5D health status indicator [8]. However, for the purposes of this paper we have separated the individual measures that make up the EQ5D in order to compare these to similar indicators of morbidity within each domain]. Two outcome measures are based on this: whether people have any pain (the 2nd and 3rd categories combined), and whether they have extreme pain (3rd category only).
Arthritis LSI	Every year 1994-2011, people who report a longstanding illness (LSI) are then asked, *'what is the matter with you?'*; up to 6 responses are then coded by the interviewer into a consistent coding frame based on the International Classification of Diseases.<br>The arthritis LSI measure is based on the group labelled *'Arthritis/rheumatism/fibrositis'*, which as of 2011 includes: Arthritis as result of broken limb; Arthritis/rheumatism in any part of the body; Gout; Osteoarthritis, rheumatoid arthritis, polymyalgia rheumatic; Polyarteritis Nodosa; Psoriasis arthritis; Rheumatic symptoms; and Still's disease.<br>While the LSI coding frame generally stays consistent over this period, interpretation of 'LSI arthritis' is complicated by two changes: Gout and Polyarteritis Nodosa are moved into this code (the documentation is not clear on whether this occurred in 2000 or 2001).
Other musculoskeletal LSI	People who report a longstanding illness (LSI) are then asked, *'what is the matter with you?'*; up to 6 responses are then coded by the interviewer into a consistent coding frame based on the International Classification of Diseases.<br>The other musculoskeletal LSI measure is based on the groups labelled *'Back problems/slipped disc/spine/neck'* and *'Other problems of bones/joints/muscles'*, which as of 2011 includes: Brittle bones, osteoporosis; Bursitis, housemaid's knee, tennis elbow; Cartilage problems; Chondrodystrophia; Chondromalacia; Cramp in hand; Deformity of limbs eg. club foot, claw-hand, malformed jaw; Delayed healing of bones or badly set fractures; Deviated septum; Disc trouble; Dislocations eg. dislocation of hip, clicky hip, dislocated knee/finger; Disseminated lupus; Dupuytren's contraction; Fibromyalgia; Flat feet, bunions; Fracture, damage or injury to extremities, ribs, collarbone, pelvis, skull, eg. knee injury, broken leg, gun shot wounds in leg/shoulder, can't hold arm out flat - broke

For peer review only - http://bmjopen.bmj.com/site/about/guidelines.xhtml

	it as a child, broken nose; Frozen shoulder; Hip infection, TB hip; Hip replacement (nes); Legs won't go, difficulty in walking; Lumbago, inflammation of spinal joint; Marfan Syndrome; Osteomyelitis; Paget's disease; Perthe's disease; Physically handicapped (nes); Pierre Robin syndrome; Prolapsed invertebral discs; Schlatter's disease; Schuermann's disease; Sever's disease; Spondylitis, spondylosis; Stiff joints, joint pains, contraction of sinews, muscle wastage; Strained leg muscles, pain in thigh muscles; Systemic sclerosis, myotonia (nes); Tenosynovitis; Torn muscle in leg, torn ligaments, tendonitis; Walk with limp as a result of polio, polio (nes), after affects of polio (nes); Weak legs, leg trouble, pain in legs; and Worn discs in spine - affects legs. The code explicitly excludes: Damage/injury to spine results in paralysis; Sciatica or trapped nerve in spine; and Muscular dystrophy.
Circulatory	
High blood pressure LSI	Every year 1994-2011, people who report a longstanding illness (LSI) are then asked, '*what is the matter with you?*'; up to 6 responses are then coded by the interviewer into a consistent coding frame based on the International Classification of Diseases.<br>The high blood pressure LSI measure is based on the group labelled '*Hypertension/high blood pressure/blood pressure (nes)*', which as of 2011 includes only the conditions listed in the group label.
Recent high blood pressure	Respondents in 1994, 1998, 2003, 2006 and 2009-2014 were asked a series of questions on whether they have high blood pressure:<br>- *"Do you now have, or have you ever had... high blood pressure (sometimes called hypertension)?"*<br>- Those responding 'yes' were then asked *"Were you told by a doctor or nurse that you had high blood pressure?"*<br>- Women responding 'yes' were then asked, *"Can I just check, were you pregnant when you were told that you had high blood pressure?"*, and those responding 'yes' were then asked *"Have you ever had high blood pressure apart from when you were pregnant?"*<br>- Finally, those with doctor-diagnosed high blood pressure (excluding only when pregnant were asked: *"Are you currently taking any medicines, tablets or pills for high blood pressure?"*, and those saying 'no' (or not giving an answer) were then asked, *"Do you still have high blood pressure?"*<br>People were considered to have recent high blood pressure if they said they had ever been diagnosed as having high blood pressure by a doctor (excluding when pregnant), and that they still have high blood pressure or are currently taking medicines for it.<br>While the question wording has stayed consistent, a discontinuity seems to be introduced by a change in question context. In some years (1994, 1998, 2003, 2006 and 2011), this question was preceded by a question that asked, *"May I just check, have you ever had your blood pressure measured by a doctor or nurse?"* (and then for those saying yes, they were asked how recently this was, and whether they were told that it was '*normal (alright/fine), higher than normal, lower than normal, or were you not told anything?*'). However, in other years (2009-10, 2012-14), this question was not asked. Given the way in which context can affect question interpretation, we treat these as two separate measures of recent high blood pressure.
Biomarker high blood pressure	During the nurse visit (which took place for all consenting respondents in all years except 1999, 2002 and 2004, when the nurse visit focussed on particular subsamples), respondents' blood pressure was measured.<br>High blood pressure is defined as a systolic blood pressure >= 140mmHg and diastolic blood pressure >= 90mmHg following HSE established practice, in turn following [10].<br>The measurement of blood pressure changed in 2003, from a Dinamap monitor to an Omron monitor. A conversion is available between the two monitors based on a calibration study, and this has been regularly used by the HSE team to produce

For peer review only - http://bmjopen.bmj.com/site/about/guidelines.xhtml

	continuous trends in blood pressure – see www.hscic.gov.uk/catalogue/PUB00480. For adults, the conversion is as follows:<br>○ For systolic blood pressure: *Predicted Omron=8.90 (SE=2.94) + 0.91 (SE=0.02) * Dinamap*. ○ For diastolic blood pressure: *Predicted Omron=19.78 (SE=1.86) + 0.73 (SE=0.03) * Dinamap*.<br>There are several reasons why respondents who had a nurse visit do not have a valid blood pressure measurement – these are discussed in the Web Appendices 2 and 3.
High cholesterol	In the years 1994, 1998, 2006, and 2008-14, blood samples were obtained during the nurse visit, which were then analysed for total cholesterol. A high level of total cholesterol ('hypercholesterolaemia') is an established risk factor for CVD, and high cholesterol is defined following conventional practice at the NICE guidance 'audit level' of 5mmol/L or above [11 12].<br><br>The measurement of cholesterol changed slightly in 2010 when a new laboratory was used. This resulted in values that are an average of 0.1mmol/L higher, and later values are therefore adjusted by this amount to maintain comparability over time as in [11].
Low HDL cholesterol	In the years 1994, 1998, 2006, and 2008-14, blood samples were obtained during the nurse visit, which were then analysed for high density lipoprotein (HDL) cholesterol. HDL cholesterol *reduces* the risk of CVD (it carries cholesterol away from the arteries towards the liver), and it is therefore low HDL cholesterol that indicates poorer health; low HDL cholesterol is here defined as 1 mmol/L or less [11 12].<br><br>The measurement of HDL cholesterol changed slightly in 2010 when a new laboratory was used. This resulted in values that are an average of 0.1mmol/L lower, and later values are therefore adjusted by this amount to maintain comparability over time as in [11].
Recent heart attack/stroke	Respondents in 1994, 1998, 2003, 2006 and 2011 were asked a series of questions on whether they have had a heart attack (within a battery of questions about different types of heart disease):<br>- *"Have you ever had a heart attack (including myocardial infarction or coronary thrombosis)?"*<br>- Those responding 'yes' were then asked *"Were you told by a doctor that you had a Heart Attack (including myocardial infarction or coronary thrombosis)?"*<br>- Those with doctor-diagnosed angina were asked, *"Have you had a heart attack (including myocardial infarction and coronary thrombosis) during the past 12 months?"*<br>Respondents in these years were similarly asked about stroke:<br>- *"Have you ever had a stroke?"*<br>- Those responding 'yes' were then asked, *"Were you told by a doctor that you had a stroke?"*<br>- Those with doctor-diagnosed stroke were asked, *"Have you had a stroke during the past 12 months?"*<br>People were considered to have recent IHD or stroke if they said they had ever been diagnosed as having stroke or a heart attack by a doctor, and that they have had a heart attack or stroke during the past 12 months.
Recent angina	Respondents in 1994, 1998, 2003, 2006 and 2011 were asked a series of questions on whether they have angina (within a battery of questions about different types of heart disease):<br>- *"Have you ever had angina?"*<br>- Those responding 'yes' were then asked *"You said that you had Angina. Were you told by a doctor that you had Angina?"*<br>- Those with doctor-diagnosed angina were asked, *"Have you had angina during the past 12 months?"*<br>People were considered to have recent angina if they said they had ever been diagnosed as having angina by a doctor, and that they have had it during the past 12 months.

For peer review only - http://bmjopen.bmj.com/site/about/guidelines.xhtml

IHD LSI	Every year 1994-2011, people who report a longstanding illness (LSI) are then asked, *'what is the matter with you?'*; up to 6 responses are then coded by the interviewer into a consistent coding frame based on the International Classification of Diseases.<br>The IHD LSI measure is based on the groups labelled *'Stroke/cerebral haemorrhage/cerebral thrombosis'* and *'Heart attack/angina'*. As of 2011 this includes: Cerebro-vascular accident; Coronary thrombosis, myocardial infarction; Heart attack/angina; Hemiplegia, apoplexy, cerebral embolism; Stroke/cerebral haemorrhage/cerebral thrombosis; and Stroke victim - partially paralysed and speech difficulty.
Recent	Respondents in 1994, 1998, 2003, 2006 and 2011 were asked a series of questions on

For peer review only

For peer review only - http://bmjopen.bmj.com/site/about/guidelines.xhtml

cardiovascular disease (CVD)	different types of heart disease – including angina; heart attack (including myocardial infarction or coronary thrombosis); a heart murmur; abnormal heart rhythm; or other heart trouble. For EACH of these, they were asked:<br>- *“Have you ever had <type of heart disease>?”*<br>- Those responding ‘yes’ were then asked *“You said that you had <type of heart disease>. Were you told by a doctor that you had <type of heart disease>?”*<br>- For heart murmurs only, women saying they had doctor-diagnosed heart murmurs were asked if they were pregnant when told this, and if so, whether they were ever told they had a heart murmur when they were not pregnant. - Those with doctor-diagnosed heart disease (excluding heart murmurs when pregnant) were asked, *“Have you had <type of heart disease> during the past 12 months?”*<br><br>People were considered to have recent CVD if they said they had a doctor-diagnosed heart condition and that they had had this during the past 12 months.
Cardiovascular (CVD) LSI	Every year 1994-2011, people who report a longstanding illness (LSI) are then asked, *‘what is the matter with you?’*; up to 6 responses are then coded by the interviewer into a consistent coding frame based on the International Classification of Diseases. The CVD LSI measure is based on the groups labelled *‘Stroke/cerebral haemorrhage/cerebral thrombosis’, ‘Heart attack/angina’, Hypertension/high blood pressure/blood pressure (nes)’, ‘Other heart problems’, ‘Piles/haemorrhoids incl. Varicose Veins in anus’, ‘Varicose veins/phlebitis in lower extremities’, and ‘Other blood vessels/embolic’*. As of 2011 this includes: Aorta replacement; Aortic valve stenosis; Aortic/mitral valve regurgitation; Arterial thrombosis; Arteriosclerosis, hardening of arteries (nes); Artificial arteries (nes); Atrial Septal Defect (ASD); Blocked arteries in leg; Blood clots (nes); Cardiac asthma; Cardiac diffusion; Cardiac problems, heart trouble (nes); Cerebrovascular accident; Coronary thrombosis, myocardial infarction; Dizziness, giddiness, balance problems (nes); Hand Arm Vibration Syndrome (White Finger); Hardening of arteries in heart; Heart attack/angina; Heart disease, heart complaint; Heart failure; Heart murmur, palpitations; Hemiplegia, apoplexy, cerebral embolism; Hole in the heart; Hypersensitive to the cold; Hypertension/high blood pressure/blood pressure (nes);<br>Intermittent claudication; Ischaemic heart disease; Low blood pressure/hypertension; Mitral valve stenosis; Pacemaker; Pains in chest (nes); Pericarditis; Piles/haemorrhoids incl. Varicose Veins in anus; Poor circulation; Pulmonary embolism; Raynaud's disease; St Vitus dance; Stroke victim - partially paralysed and speech difficulty; Stroke/cerebral haemorrhage/cerebral thrombosis; Swollen legs and feet; Tachycardia, sick sinus syndrome; Telangiectasia (nes); Thrombosis (nes); Tired heart; Valvular heart disease; Valvular heart disease; Varicose veins in Oesophagus; Varicose veins/phlebitis in lower extremities; Various ulcers, varicose eczema; Weak heart because of rheumatic fever; Wolff - Parkinson - White syndrome; and Wright's syndrome. It explicitly excludes balance problems due to ear complaint & haemorrhage behind eye.<br><br>While the LSI coding frame generally stays consistent over this period, interpretation of ‘IHD LSI’ is complicated by two changes: ‘Too much cholesterol in blood’ is included in this category in 1994 only, and Polyarteritis Nodosa is later moved into this code (the documentation is not clear on whether this occurred in 2000 or 2001).
Angina symptoms	This is taken from the Rose Angina questionnaire [13,14]. Respondents in 1994, 1998, 2003, 2006 and 2011 were asked a series of questions about *symptoms* of heart trouble (rather than whether they had been diagnosed):<br>- *“I am now going to ask you some questions mainly about symptoms of the chest. Have you ever had any pain or discomfort in your chest?”* - Those that said ‘yes’ were asked:<br>o *Do you get it when you walk uphill or hurry? Yes | No | Sometimes/ Occasionally | Never walks uphill or hurries | (Cannot walk)”.* If sometimes/occasionally, they were asked: *“Does this happen on most occasions?”*<br>o If not ‘no’ to having pain/discomfort in their chest, they were asked: *“Do you get it when you walk at an ordinary pace on the level? Yes | No |*

For peer review only - http://bmjopen.bmj.com/site/about/guidelines.xhtml

	Sometimes/Occasionally | Never walks at an ordinary pace on the level". If sometimes/occasionally, they were asked: *"Does this happen on most occasions?"*<br>- Those who every had pain/discomfort when walking uphill/hurrying or walking at ordinary pace on the level were asked:<br>o *"What do you do if you get it while you are walking? Do you stop, slow down or carry on?"* (If respondents were unsure, they were asked, *"What do you do on most occasions?")*<br>o Those who said they stop or slow down were asked, *"If you stand still does the pain go away or not?"* (If respondents were unsure, they were asked, *"What happens to the pain on most occasions?")*. If the pain goes away, they were asked, *"How soon does the pain go away? Does it go in 10 minutes or less, or more than 10 minutes?"*<br>o Those who said the pain goes away in 10 minutes or less were asked, *"Will you show me where you get this pain or discomfort? Where else"* The interviewer then coded the site as Sternum (upper or middle) | Sternum lower | Left anterior chest | Left arm | Right anterior chest | Right arm | (Somewhere else).<br>Following the HSE reports, possible angina is defined as chest pain or discomfort that (i) includes either the sternum or the left arm and left anterior chest; (ii) is prompted by hurrying or walking uphill (or by walking on the level, for those who never attempt more); (iii) makes the respondent either stop or slacken pace; and (iv) usually disappears in 10 minutes or less when they stand still.
Heart attack symptoms	This is taken from the Rose Angina questionnaire. Respondents in 1994, 1998, 2003, 2006 and 2011 were asked, *"Have you ever had a severe pain across the front of your chest lasting for half an hour or more?"* As in the 2006 HSE report, those responding 'yes' are treated as having a possible heart attack (myocardial infarction).
Mini stroke (TIA) symptoms	Respondents in 2003, 2006 and 2011 were asked:<br>o *"In the last twelve months, have you had a sudden attack of weakness or numbness on one side of the body?"*<br>o *"Have you had a sudden attack of slurred speech or difficulty in finding words in the last twelve months?"*<br>o *"Have you had a sudden attack of vision loss or blurred vision in one or both eyes in the last twelve months?"*<br>People reporting ANY of these symptoms were considered as possibly having had a transient ischaemic attack (TIA), often called a 'mini stroke'.
Respiratory	
COPD symptoms	Respondents in 1995, 1996 and 2010 were asked:<br>o *"Do you usually cough first thing in the morning in the winter?"* (In 2010 only, respondents had previously been asked *"Do you usually cough first thing in the morning?"* – but this is not used to filter people into the questions on coughing in winter).<br>o *"Do you usually bring up any phlegm from your chest, first thing in the morning in the winter?"* (Again, this was asked to everyone in all years, but was preceded by an additional, non-winter-specific question in 2010).<br>o Those saying 'yes' to each question were then asked, "*Do you [cough/bring up phlegm] like this on most days for as much as three months each year?"* In 2010 only, this was followed by the additional clarification '*That is, for three consecutive months'*.<br>People who reported three months/year of BOTH coughing first thing and of phlegm are considered to have possible symptoms of Chronic Obstructive Pulmonary Disease (COPD).

For peer review only - http://bmjopen.bmj.com/site/about/guidelines.xhtml

Diagnosed asthma

In 1995-7, 2001 and 2010, respondents were asked *"Did a doctor <1997 and 2010 only: or nurse> ever tell you that you had asthma?"* Whereas for other doctor-diagnosed conditions (heart problems/diabetes) we focus on those reporting problems in the past 12 months, it is not possible to construct a consistent measure of recent asthma, hence this variable refers to *lifetime* doctor-diagnosed asthma.

Asthma LSI

Every year 1994-2011, people who report a longstanding illness (LSI) are then asked, *'what is the matter with you?'*; up to 6 responses are then coded by the interviewer into a consistent coding frame based on the International Classification of Diseases.

The asthma LSI measure is based on the group labelled *'Asthma'*, which as of 2011 includes: Asthma; Bronchial asthma, allergic asthma; and Asthma - allergy to house dust/grass/cat fur. It explicitly <u>excludes</u> cardiac asthma.

Shortness of breath *(Grade 2+ / Grade 3)*

Respondents in 1995, 1996 and 2010 were asked the following questions about shortness of breath ('dyspnoea'):

- *"Are you troubled by shortness of breath when hurrying on level ground or walking up a slight hill? Yes | No | Never walks up hill or hurries | Cannot walk"*
- Those responding 'yes' or 'never walks up hill or hurries' are then asked, *"Do you get short of breath walking with other people of (your/his/her) own age on level ground? Yes | No | Never walks with people of own age on level ground"*.
- Those responding 'yes' or 'never walks with people of own age' are then asked, *"Do you have to stop for breath after walking at (your/his/her) own pace on level ground?"*

This has been combined into the longstanding MRC dyspnoea scale [15] as follows:

- Grade 2 dyspnoea: people who report shortness of breath when hurrying on level ground or walking up a slight hill (or who report shortness of breath when walking on level ground, but who say they never walk up hill or hurry).
- Grade 3 dyspnoea: people who report shortness of breath when walking with people of own age on level ground, or who have to stop for breath when walking at own pace on level ground.

(The same questions also exist in 1994 and 1998, but (i) the wider bank of questions differs substantially in the two versions and question context effects are likely; and (ii) the filtering into the final question differs between versions. However, the 1991-98 trends are included below).

Recent wheezing/ asthma symptoms

Respondents in 1995-97, 2001 and 2010 were asked the following two questions as part of the battery of questions on breathing problems:

- *"I am now going to ask you some questions about your breathing... Have you ever had wheezing or whistling in the chest at any time, either now, or in the past?"*
- Those that said yes were then asked, *"Have you had wheezing or whistling in the chest in the last 12 months?"*
- (For those who said they had ever been told by a doctor they had asthma; see above), "*When was your most recent attack of asthma? PROMPT IF NECESSARY: Less than 4 weeks ago | More than 4 weeks but within the last 12 months | One to five years ago | More than 5 years ago"*

People who said they had EITHER wheezing/whistling in the past 12 months or an asthma attack in the past 12 months were counted as having recent wheezing/asthma symptoms.

[It should be noted that the filtering to the second question is very slightly different in 2010 compared to previous years (it was only asked to people who said they had not had wheezing/whistling in the chest in the past 12 months). However, given the way that the derived variable is calculated here, the change in filtering does not introduce any discontinuities over time].

For peer review only - http://bmjopen.bmj.com/site/about/guidelines.xhtml

Wheezing stopping sleep	Respondents in 1995-97, 2001 and 2010 were asked the following two questions as part of the battery of questions on breathing problems:<br>- *"I am now going to ask you some questions about your breathing... Have you ever had wheezing or whistling in the chest at any time, either now, or in the past?"*<br>- Those that said yes were then asked, *"Have you had wheezing or whistling in the chest in the last 12 months?"*<br>- Those that said yes were then asked, *"In the last 12 months, how often on average has your sleep been disturbed due to wheezing or whistling in the chest?: Have you: Never woken with wheezing | Woken less than one night per week, or | Woken one or more nights per week?"*<br>People were considered to have wheezing during sleep if they reported this at least once per week.
Anthropometric & diabetes	
BMI *(Underweight / Obese)*	During the initial face-to-face interview in all years (except 2013), respondents were asked if they would consent to having their height and weight measured by the interviewer. The reasons for missingness (and their trends over time) are given in Web Appendices 2 & 3; note that there are three changes that give rise to small discontinuities in 2009 and 2011.<br>Obesity is a risk factor for diabetes (hence its inclusion in this section) but also heart disease and some cancers. Obesity is defined as a Body Mass Index (BMI) of >= 30kg/m^2 as per the World Health Organization's BMI classification [16]. Using the same definition, underweight is defined as <=18.5 kg/m^2.
High waist-hip ratio	During the nurse visit in most years (excluding 1995-96, 2002, 2004 and 2013), respondents had their waist and hip circumferences measured. While BMI is a standard measurement of obesity, some evidence suggests that fat around the waist – 'central adiposity' – is a greater risk to health than fat elsewhere [17]. We use NICE's suggested 2006 thresholds for a high waist-hip ratio of >1 for men and >0.85 for women [18], as used in Hotchkiss et al [19].
Recent diabetes	Respondents in 1994, 1998, 2003, 2006 and 2009-2014 were asked a series of questions on whether they have diabetes:<br>- *"Do you now have, or have you ever had diabetes?"*<br>- Those responding 'yes' were then asked *"Were you told by a doctor that you had diabetes?"*<br>- Women responding 'yes' were then asked, *"Can I just check, were you pregnant when you were told that you had diabetes?"*, and those responding 'yes' were then asked *"Have you ever had diabetes apart from when you were pregnant?"*<br>- Finally, those with doctor-diagnosed diabetes (excluding only when pregnant were asked: *"Do you currently inject insulin for diabetes?"* and *"Are you currently taking any medicines, tablets or pills (other than insulin injections) for diabetes?"*<br>People were considered to have recent diabetes if they said they had ever been diagnosed as having diabetes by a doctor (excluding when pregnant), and that they are injecting insulin or taking any other medicines for diabetes.
Diabetes LSI	Every year 1994-2011, people who report a longstanding illness (LSI) are then asked, *'what is the matter with you?'*; up to 6 responses are then coded by the interviewer into a consistent coding frame based on the International Classification of Diseases.<br>The diabetes LSI measure is based on the group labelled *'Diabetes'*, which as of 2011 includes Diabetes and Hyperglycaemia.

For peer review only - http://bmjopen.bmj.com/site/about/guidelines.xhtml

High glycated haemoglobin	In the years 2003, 2006, and 2008-14, blood samples were obtained during the nurse visit, which were then analysed for glycated haemoglobin (HbA_{1C}). HbA_{1C} is a measure of the share of haemoglobin (within red blood cells) that glucose is attached to, with higher levels indicated less well-controlled diabetes in the previous three months [20]. Following the recommendations of a 2009 expert committee, we mirror recent HSE reports in using a threshold of 48mmol/mol (i.e. 48 millimoles of glycated haemoglobin per mole of haemoglobin) as the threshold for raised HbA_{1C}, a different threshold to that used in earlier HSE reports.<br><br>While the measurement of HbA_{1C} has been consistent in HSE from 1994, the units reported have changed from the % of haemoglobin that is glycated to mmol/mol. Earlier measures have been transformed into mmol/mol through the formula, mmol/mol = (% - 2.15) x 10.929. HbA_{1C} was also measured in 1994 but using a different technique, which cannot be made comparable [21:67].

Other biomarkers

Raised C-reactive protein	In the years 1998, 2003, 2006, and 2009, blood samples were obtained during the nurse visit, which were then analysed for C-reactive protein (CRP). CRP is an inflammatory marker, which can indicate heart-related inflammation (it is used to test for heart failure) but can also indicate other sorts of health damage including diabetes. However, there are still debates about exactly what CRP shows, both in terms of its causal role in heart disease, and whether it also indicates depression.[22]<br><br>Raised CRP is defined as >3mg/L, the standard cut-off for a clinically significant rise in CVD [23 24]. Participants with CRP >10mg/L are excluded, as this is taken to be evidence of current infection rather than inflammation from chronic disease.
Raised Fibrinogen	In the years 1998, 2003, 2006, and 2009, blood samples were obtained during the nurse visit, which were then analysed for fibrinogen. Like CRP, fibrinogen is an inflammatory marker, which is both commonly thought to be a causal risk factor for CVD (it is a component of coagulation), and which seems to be a risk factor for other diseases (including cancer and diabetes)[25].<br><br>While fibrinogen is often analysed as a continuous variable with no cutpoints [24], we here define raised fibrinogen as>4mg/L as in [12]. As for CRP, participants with CRP >10mg/L are excluded, as this is taken to be evidence of current infection rather than inflammation from chronic disease. A change of analysis method and laboratory between 1994 and 1998 means that the 1994 results are not comparable to the later results [26:8.10.4].
Anaemia	In the years 1994, 1998, 2006, and 2009, blood samples were obtained during the nurse visit, which were then analysed for haemoglobin. Haemoglobin dist ributes oxygen around the body, and low haemoglobin levels usually indicate anaemia. Various different thresholds for low haemoglobin have been used in the literature, particularly for older populations [27], but we here used the longstanding WHO definition of <13g/dL for men and <12g/dL for women [24].
Iron deficiency	In the years 1994, 1998, 2006, and 2009, blood samples were obtained during the nurse visit, which were then analysed for serum ferritin (which correlates directly with the amount of iron stored in the body). Iron deficiency is one of several possible causes of anaemia (alongside other nutritional deficiencies, genetic conditions such as sickle cell anaemia, infections, and blood loss). Iron deficiency is defined as a serum ferritin less than 45ng/ml [27].

Mental health

Mental health LSI	Every year 1994-2011, people who report a longstanding illness (LSI) are then asked, *'what is the matter with you?'*; up to 6 responses are then coded by the interviewer into a consistent coding frame based on the International Classification of Diseases. The mental health LSI measure is based on the group labelled *'Mental illness/anxiety/depression/nerves (nes)'*, which as of 2011 includes: Alcoholism, recovered not cured alcoholic; Angelman Syndrome; Anorexia nervosa; Anxiety, panic attacks; Asperger

For peer review only - http://bmjopen.bmj.com/site/about/guidelines.xhtml

Syndrome; Autism/Autistic (BBG: changed from 'autistic child'); Bipolar Affective Disorder; Catalepsy; Concussion syndrome; Depression; Drug addict; Dyslexia; Hyperactive child.; Nerves (nes); Nervous breakdown, neurasthenia, nervous trouble; Phobias; Schizophrenia, manic depressive; Senile dementia, forgetfulness, gets confused; Speech impediment, stammer; and Stress. It explicitly excludes Alzheimer's disease, degenerative brain disease.

While the LSI coding frame generally stays consistent over this period, it is worth being aware of a minor wording change within 'mental health LSI': the condition labelled 'Autistic child' 1994-1997 was relabelled 'Autism/Autistic' in 1998.

Psychological distress (GHQ)

In the self-completion survey in most years (except 1996, 2007, 2011 and 2013), respondents were asked the following series of questions:

- *"Please read this carefully: We should like to know how your health has been in general over the past few weeks. Please answer ALL the questions by ticking the box below the answer which you think most applies to you. Have you recently...*
- *"…been able to concentrate on whatever you're doing?" RESPONSES: "Better than usual" | "Same as usual" | "Less than usual" | "Much less than usual"*
- *"…lost much sleep over worry?" RESPONSES: "Not at all" | "No more than usual" | "Rather more than usual" | "Much more than usual""*
- *"…felt you were playing a useful part in things?" RESPONSES: "More so than usual" | "Same as usual" | "Less useful than usual" | "Much less useful""*
- *"…felt capable of making decisions about things?" RESPONSES: "More so than usual" | "Same as usual" | "Less so than usual" | "Much less capable""*
- *"…felt constantly under strain? RESPONSES: "Not at all" | "No more than usual" | "Rather more than usual" | "Much more than usual""*
- *"..felt you couldn't overcome your difficulties?" RESPONSES: "Not at all" | "No more than usual" | "Rather more than usual" | "Much more than usual""*
- *"…been able to enjoy your normal day-to-day activities?" RESPONSES: "More so than usual" | "Same as usual" | "Less so than usual" | "Much less than usual"*
- *"…been able to face up to your problems?" RESPONSES: "More so than usual" | "Same as usual" | "Less able than usual" | "Much less able"*
- *"…been feeling unhappy and depressed? RESPONSES: "Not at all" | "No more than usual" | "Rather more than usual" | "Much more than usual"*
- *"…been losing confidence in yourself? RESPONSES: "Not at all" | "No more than usual" | "Rather more than usual" | "Much more than usual"*
- *"…been thinking of yourself as a worthless person?" RESPONSES: "Not at all" | "No more than usual" | "Rather more than usual" | "Much more than usual""*
- *"…been feeling reasonably happy, all things considered?" RESPONSES: "More so than usual" | "Same as usual" | "Less so than usual" | "Much less happy"*

These make up the 12-item General Health Questionnaire GHQ-12; [28], a well-validated, widely-used measure of probable mental ill-health. This is often termed general nonpsychotic psychiatric morbidity, but I here use the more easily understood term 'psychological distress' following Stochl et al 2016.[29]

A total score has been created by first ensuring that all questions were coded from 1 (positive symptom) to 4 (negative symptom), and then creating a sum score for all the number of questions in which people answered with categories 3 or 4 (indicating a negative symptom). A binary measure (often called GHQ caseness) was created for people who had negative symptoms for 4 or more of the 12 questions.

Anxiety/depression (moderately / Extremely)

In the self-completion survey in 1996, 2003-6, 2008, 2010-12 and 2014, respondents were asked *'Now we would like to know how your health is today. Please answer ALL the questions. By ticking one box for each question below, please indicate which statements best describe your own health state today'*:

- *"I am not anxious or depressed"*
- *"I am moderately anxious or depressed"*
- *"I am extremely anxious or depressed"*

For peer review only - http://bmjopen.bmj.com/site/about/guidelines.xhtml

	[This is part of the widely-used EQ-5D health status indicator [8]. However, for the purposes of this paper we have separated the individual measures that make up the EQ5D in order to compare these to similar indicators of morbidity within each domain]. Two outcome measures are based on this: whether people have any anxiety/depression (the 2nd and 3rd categories combined), and whether they have extreme anxiety/depression (3rd category only).
Communication	
Hearing, seeing & communication limitations	These measures were not included in the main paper due to the short time frame that we can examine trends over, but are included in the Web Appendix as they relate to important domains of morbidity.<br>They were included in the disability scale used in the 2001 HSE report [9]. Respondents in 1995, 2000 and 2001 were asked if of the following applied to them (interviewers were instructed to ignore temporary disabilities that are expected to last less than one year):<br>• *"Cannot follow a TV programme at a volume others find acceptable (with hearing aid if normally worn)"* ('hearing limitation')<br>• *"Cannot see well enough to recognise a friend across a road (four yards away) (with glasses or contact lenses if normally worn)"* ('seeing limitation')<br>• *"Have problem communicating with other people - that is, have problem understanding them or being understood by them"* ('communication limitation')
Eye/Ear LSI	Every year 1994-2011, people who report a longstanding illness (LSI) are then asked, *'what is the matter with you?'*; up to 6 responses are then coded by the interviewer into a consistent coding frame based on the International Classification of Diseases. The Eye/Ear LSI includes the following groups:<br>• *Poor hearing/deafness*, including Conductive/nerve/noise induced deafness, Deaf mute/deaf and dumb, Heard of hearing, slightly deaf, Otosclerosis, Poor hearing after mastoid operation.<br>• *Tinnitus/noises in the ear*, Incl. pulsing in the ear<br>• *Other ear complaints*, Incl. otitis media - glue ear, Disorders of Eustachian tube, Perforated ear drum (nes), Middle/inner ear problems, Mastoiditis, Ear trouble (nes),, Ear problem (wax), Ear aches and discharges, Ear infection<br>• *Cataract/poor eye sight/blindness*, Incl. operation for cataracts, now need glasses, Bad eyesight, restricted vision, partially sighted, Bad eyesight/nearly blind because of cataracts, Blind in one eye, loss of one eye, Blindness caused by diabetes, Blurred vision, Detached/scarred retina, Hardening of lens, Lens implants in both eyes, Short sighted, long sighted, myopia, Trouble with eyes (nes), eyes not good (nes), Tunnel vision<br>• *Other eye complaints*, including Astigmatism, Buphthalmos, Colour blind, Double vision, Dry eye syndrome, trouble with tear ducts, watery eyes, Eye infection, conjunctivitis, Eyes are light sensitive, Floater in eye, Glaucoma, Haemorrhage behind eye, Injury to eye, Iritis, Keratoconus, Night blindness, Retinitis pigmentosa, Scarred cornea, corneal ulcers, Squint, lazy eye, Sty on eye.

Changes over time in several other measures are only presented in Web Appendices 4 & 6, rather than the main paper. Details of these variables are included below:

**Measure**	**Details**
General health	
General health *(bad / good)*	Every year, respondents were asked, *"How is your health in general? Would you say it was … very good, good, fair, bad, or very bad?"*

For peer review only - http://bmjopen.bmj.com/site/about/guidelines.xhtml

	Two outcome measures are based on this, following standard practice in the HSE reports: bad general health (which includes 'bad' or 'very bad' health) and good general health (which includes 'good' or 'very good' health).
Longstanding illness (LSI)	Every year 1994-2011, respondents were asked *"Do you have any long-standing illness, disability or infirmity? By long-standing I mean anything that has troubled you over a period of time, or that is likely to affect you over a period of time?"* (The response options were 'Yes' and 'No').<br>In 2012 the question was changed to be consistent with the Government's new harmonised disability questions for use in social surveys [30], and is not comparable to the previous version.
Limiting LSI	Every year 1996-2011, respondents who said they had an LSI were than asked, *"Does this illness or disability (do any of these illnesses or disabilities) limit your activities in any way?"* (again allowing only Yes/No answers).<br>In 2012 the question was changed to be consistent with the Government's new harmonised disability questions for use in social surveys (see HSE 2012 report), and is not comparable to the previous version.
Problems with usual activities *(some problems / unable)*	In the self-completion survey in 1996, 2003-6, 2008, 2010-12 and 2014, respondents were asked *'Now we would like to know how your health is today. Please answer ALL the questions. By ticking one box for each question below, please indicate which statements best describe your own health state today'*:<br>- *"I have no problems with performing my usual activities (e.g. work, study, housework, family or leisure activities)"*<br>- *"I have some problems with performing my usual activities"*<br>- *"I am unable to perform my usual activities"*<br>[This is part of the widely-used EQ-5D health status indicator [8]. However, for the purposes of this paper we have separated the individual measures that make up the EQ5D in order to compare these to similar indicators of morbidity within each domain].<br>Two outcome measures are based on this: whether people have any problems (the 2nd and 3rd categories combined), and whether they are unable to perform their usual activities (3rd category only).
Limitations in past 2wks	Every year, respondents were asked, *"Now I'd like you to think about the two weeks ending yesterday. During those 2 weeks did you have to cut down on any of the things you usually do (about the house or at work or in your free time) because of your answer at <the LSI question> or some other illness or injury?"*<br>There have been two small changes to this question's wording in 1996. Firstly, 'work' was changed to 'work/school'. Secondly, *'your answer at <the LSI question>'* was changed to *'a condition you have just told me about'*. While it is impossible to be sure of the exact effect of these changes, neither seem likely to influence the results (at least for the 25+ age group where fewer individuals are in full-time education).

For peer review only - http://bmjopen.bmj.com/site/about/guidelines.xhtml

Appendix 6: Measures not included in the main paper

Trends in several measures are not included in the main paper, either

Table 1: Changes over time in measures not included in the main paper

	Starting period		Change from start to end period			
	Period	Prevalence	End period	Raw change	Adj.[a] change	Adj. change 95% CI
CVD						
Component measures nec[b]						
Recent heart murmur	1994-96	0.8%	2011-14	0.1%	0.0%	[-0.3, 0.4%]
Recent irregular heart rhythm	1994-96	1.6%	2011-14	0.4%	0.4%	[-0.1, 0.8%]
Recent other heart disease	1994-96	0.2%	2011-14	0.7%	0.7%	[0.4, 0.9%]
Ever had (not just recent)						
Ever had high BP DD	1994-96	19.0%	2011-14	4.5%	3.7%	[2.3, 5.1%]
high BP	1994-96	13.2%	2011-14	6.9%	6.0%	[4.7, 7.3%]
Ever IHD or stroke	1994-96	2.9%	2011-14	0.3%	-0.0%	[-0.6, 0.6%]
DD IHD or stroke	1994-96	2.5%	2011-14	0.5%	0.2%	[-0.3, 0.7%]
Ever had angina	1994-96	1.9%	2011-14	-0.2%	-0.4%	[-0.9, 0.0%]
Ever DD angina	1994-96	1.6%	2011-14	-0.1%	-0.3%	[-0.7, 0.1%]
Ever heart murmur	1994-96	3.2%	2011-14	-0.3%	-0.3%	[-0.9, 0.3%]
DD heart murmur	1994-96	2.6%	2011-14	-0.2%	-0.2%	[-0.7, 0.3%]
Ever irregular heart rhythm	1994-96	6.4%	2011-14	-0.7%	-0.9%	[-1.7, -0.1%]
DD irregular heart rhythm	1994-96	3.5%	2011-14	0.5%	0.3%	[-0.3, 1.0%]
Ever other heart disease	1994-96	0.9%	2011-14	1.1%	1.0%	[0.6, 1.5%]
DD other heart disease	1994-96	0.8%	2011-14	1.0%	1.0%	[0.6, 1.4%]
Respiratory						
**Alternate measures**						
Phlegm symptoms	1994-96	9.1%	2008-10	-1.3%	-1.4%	[-2.3, -0.5%]
LSI Respiratory All	1994-96	7.9%	2011-14	-0.7%	-0.7%	[-1.6, 0.1%]
**Ever had (not just recent)**						
Wheezing Ever	1994-96	32.3%	2008-10	0.0%	-0.1%	[-1.8, 1.5%]
Wheezing Past 12mths	1994-96	18.9%	2008-10	-1.0%	-1.1%	[-2.3, 0.2%]
Diabetes						
**Ever had (not just recent)**						
Ever diabetes	1994-96	2.0%	2011-14	2.9%	2.8%	[2.3, 3.2%]
DD diabetes	1994-96	1.7%	2011-14	2.5%	2.3%	[2.0, 2.7%]
Mental health						
**Alternate measures**						
High psychological distress	1994-96	3.2%	2011-14	1.0%	0.9%	[0.4, 1.4%]
Activity limitations & musculoskeletal						
**For comparison**						
Walking limitation	1994-96	4.6%	2001-03	1.4%	1.2%	[0.5, 1.9%]
Washing/dressing limitation	1994-96	1.9%	2001-03	0.5%	0.4%	[0.0, 0.8%]

For peer review only - http://bmjopen.bmj.com/site/about/guidelines.xhtml

Other LSIs	Starting period		Change from start to end period			
	Period	Prevalence	End period	Raw change	Adj.a change	Adj. change 95% CI
LSI Blood Disorders	1994-96	0.3%	2011-14	0.6%	0.5%	[0.3, 0.8%]
LSI Cancer	1994-96	1.0%	2011-14	0.3%	0.3%	[-0.1, 0.6%]
LSI D,GUM,E&M	1994-96	6.9%	2011-14	1.1%	0.8%	[0.0, 1.6%]
LSI Epilepsy	1994-96	0.7%	2011-14	0.1%	0.1%	[-0.2, 0.3%]
LSI Nervous System	1994-96	3.7%	2011-14	-0.2%	-0.3%	[-0.8, 0.3%]

a 'Adj.' = trend adjusted for changing age and sex distribution of the working-age population. b 'nec' = not elsewhere included.

The details of these measures are as follows:

Measure	Details
**Circulatory**	
Beyond 'recent': 'Ever had' and 'DD' CVD	In the main paper, we look at whether people report recent doctor-diagnosed CVD (looking separately at heart attack/stroke, angina, and any recent CVD). As shown above, this comes from three questions: whether people report ever having this condition; whether a doctor diagnosed this; and whether they have had an attack in the past 12 months / consider themselves to still have the condition.<br>Web Appendix 6 shows trends in the other versions of these measures, i.e. having ever had this type of CVD, and having ever doctor-diagnosed ('DD') CVD of this type.
Component measure: Heart murmur Irregular heart rhythm Other heart disease	In the main paper, we recent reports of doctor-diagnosed angina; heart attack (including myocardial infarction or coronary thrombosis); a heart murmur; abnormal heart rhythm; or other heart trouble (see above). Angina and heart attack are also analysed in the main paper in their own right; in Web Appendix 6, we further show trends separately in heart murmur, abnormal heart rhythm or other heart trouble.
**Respiratory**	
Component measure: 'phlegm'	In the main paper, we look at whether people report recent COPD (see above). This combines two measures: regular cough + phlegm. Web Appendix 6 shows the trend in the phlegm measure on its own, without being combined with a regular cough.
Alternative version: respiratory' longstanding illness ('LSI')	In the main paper, we look at whether an asthma LSI (to examine alongside a direct 'LSI question on diagnosed asthma); see above. Web Appendix 6 also shows people reporting a which is included within the broader category of respiratory conditions.<br>The respiratory LSI measure is based on the group labelled *'Asthma'*, *'Bronchitis'*, *'Hayfever'*, or *'Respiratory other'*, which as of 2011 includes:<br>Asthma: Asthma; Bronchial asthma, allergic asthma; and Asthma - allergy to house dust/grass/cat fur. It explicitly <u>excludes</u> cardiac asthma.<br>Hayfever: Hayfever, Allergic rhinitis<br>Bronchitis/emphysema: Bronchitis/emphysema, Bronchiectasis, Chronic bronchitis.<br>Other respiratory complaints: Other respiratory complaints, Abscess on larynx, Adenoid problems, nasal polyps, Allergy to dust/cat fur, Bad chest (nes), weak chest – wheezy, Breathlessness, Bronchial trouble, chest trouble (nes), Catarrh, Chest infections, get a lot of colds, Churg-Strauss syndrome, Chronic<br>Obstructive Pulmonary Disease (COPD), Coughing fits, Croup, Damaged lung (nes), lost lower lobe of left lung, Fibrosis of lung, Furred up airways,

For peer review only - http://bmjopen.bmj.com/site/about/guidelines.xhtml

collapsed lung, Lung complaint (nes), lung problems (nes), Lung damage by viral pneumonia, Paralysis of vocal cords, Pigeon fancier's lung, Pneumoconiosis, byssinosis, asbestosis and other industrial respiratory disease, Recurrent pleurisy, Rhinitis (nes), Sinus trouble, sinusitis, Sore throat, pharyngitis, Throat

For peer review only

For peer review only - http://bmjopen.bmj.com/site/about/guidelines.xhtml

Measure	Details
	infection, Throat trouble (nes), throat irritation, Tonsillitis, Ulcer on lung, fluid on lung. Note that:<br>• It explicitly excludes TB (pulmonary tuberculosis), Cystic fibrosis, Skin allergy, Food allergy, Allergy (nes), Pilonidal sinus, Sick sinus syndrome, Whooping cough.
For comparison: Washing & dressing limitation	This is based on the personal care disability scale used in the 2001 HSE report [9]. Respondents in 1995, 2000 and 2001 were asked if any of the following applied to them (interviewers were instructed to ignore temporary disabilities that are expected to last less than one year):<br>- *"Cannot dress and undress without difficulty"*<br>- *"Cannot wash hands and face without difficulty"*<br>For comparison to the 'problems with washing/dressing today' measure in the main paper (which covers a more extended period and is based on a different question; see above), a measure is derived if respondents say they report either of these problems.
Other LSIs	

For peer review only - http://bmjopen.bmj.com/site/about/guidelines.xhtml

Measure	Details
Other LSIs	Every year 1994-2011, people who report a longstanding illness (LSI) are then asked, *'what is the matter with you?'*; up to 6 responses are then coded by the interviewer into a consistent coding frame based on the International Classification of Diseases. The various other LSIs are as follows:<br>• The Blood Disorders LSI measure is based on the group *'Disorders of blood and blood forming organs and immunity disorders'*, which as of 2011 includes: Anaemia, pernicious anaemia, Blood condition (nes), blood deficiency, Haemophilia, Idiopathic Thrombochopenic Purpura (ITP), Immunodeficiences, Polycthaemia (blood thickening), blood to thick, Purpura (nes), Removal of spleen, Sarcoidosis (previously code 37), Sickle cell anaemia/disease, Thalassaemia, Thrombocythenia. It explicitly excludes Leukaemia - code 01.<br>• The Cancer LSI measure is based on the group *'Cancer (neoplasm) including lumps, masses, tumours and growths and benign (non-malignant) lumps and cysts'*, which as of
	• If complaint is breathlessness with the cause also stated, this is coded with the cause – hence it also excludes breathlessness as a result of anaemia, breathlessness due to hole in heart, and breathlessness due to angina.
Component measure: Wheezing	In the main paper, we look at whether people report recent wheezing/asthma. As shown above, this comes from three questions: whether people report ever having had wheezing or whistling in the chest; whether they have had this in the past 12 months; and whether they have had an asthma attack in the past 12 months.<br>Web Appendix 6 shows trends in the other versions of these measures, i.e. having ever had wheezing/whistling in the chest, and whether they have had this in the past 1 months.
Beyond 'recent': 'Ever had' and 'DD' having diabetes	In the main paper, we look at whether people report recent doctor-diagnosed diabetes As shown above, this comes from three questions: whether people report ever this condition; whether a doctor diagnosed this; and whether they currently inject insulin / take other medication for diabetes.<br>Web Appendix 6 shows trends in the other versions of these measures, i.e. having ever had diabetes, and having ever doctor-diagnosed ('DD') diabetes.
Activity limitations	
For comparison: Walking limitation	This is based on the personal care disability scale used in the 2001 HSE report [9]. Respondents in 1995, 2000 and 2001 were asked if of the following applied to them (interviewers were instructed to ignore temporary disabilities that are expected to last less than one year): *"Cannot walk 200 yards or more on own without stopping or discomfort"*. People who reported a limitation were asked if they used a walking aid, and if they did, were then asked if they could walk 200 yards without the walking aid.
	2011 includes: Acoustic neuroma, After effect of cancer (nes), All tumours, growths, masses, lumps and cysts, whether malignant or benign eg. tumour on brain,, growth in bowel, growth on spinal cord, lump in, breast, Cancers sited in any part of the body or system eg., Lung, breast, stomach, Colostomy caused by cancer, Cyst on eye, cyst in kidney., General arthroma, Hereditary cancer, Hodgkin's disease, Hysterectomy for cancer of womb, Inch. leukaemia (cancer of the blood), Lymphoma, Mastectomy (nes), Neurofibromatosis, Part of intestines removed (cancer), Pituitary gland removed (cancer), Rodent ulcers, Sarcomas, carcinomas, Skin cancer, bone cancer, Wilms tumour

For peer review only - http://bmjopen.bmj.com/site/about/guidelines.xhtml

Measure	Details
	• The D,GUM,E&M (Digestive, Genitourinary Medicine, and Endocrine & Metabolic) LSI is based on the groups, *'Complaints of bowel/colon (large intestine,caecum, bowel, colon, rectum)'* (including Colitis, colon trouble, ulcerative colitis, Coleliac, Colostomy (nes), Crohn's disease, Diverticulitis, Enteritis, Faecal incontinence/encopresis., Frequent diarrhoea, constipation, Grumbling appendix, Hirschsprung's disease, Irritable bowel, inflammation of bowel, Polyp on bowel, Spastic colon, but explicitly excluding piles and Cancer of stomach/bowel), *Other digestive complaints (stomach, liver, pancreas, bile ducts, small intestine - duodenum, jejunum and ileum)* (including Cirrhosis of the liver, liver problems, Food allergies, Ileostomy, Indigestion, heart burn, dyspepsia, Inflamed duodenum, Liver disease, biliary artesia, Nervous stomach, acid stomach, Pancreas problems, Stomach trouble (nes), abdominal trouble (nes), Stone in gallbladder, gallbladder problems, Throat trouble - difficulty in swallowing, Weakness in intestines), *Stomach ulcer/ulcer (nes)/abdominal hernia/rupture* (including Double/inguinal/diaphragm/hiatus/umbilical hernia, Gastric/duodenal/peptic ulcer, Hernia (nes), rupture (nes), Ulcer (nes)), *Complaints of teeth/mouth/tongue* (including Cleft palate, hare lip, Impacted wisdom tooth, gingivitis, No sense of taste, Ulcers on tongue, mouth ulcers), *Other endocrine/metabolic* (including Addison's disease, Beckwith - Wiedemann syndrome, Coeliac disease, Cushing's syndrome, Cystic fibrosis, Gilbert's syndrome, Hormone deficiency, deficiency of growth hormone,, dwarfism, Hypercalcemia, Hypopotassaemia, lack of potassium, Malacia, Myxoedema (nes), Obesity/overweight, Phenylketonuria, Rickets, Too much cholesterol in blood, Underactive/overactive thyroid, goitre, Water/fluid retention, Wilson's disease, but explicitly excluding Thyroid trouble and tiredness and Overactive thyroid and swelling in neck, *Other bladder problems/incontinence* (including Bed wetting, enuresis, Bladder restriction, Water trouble (nes), Weak bladder, bladder complaint (nes), but explicitly excluding Prostate trouble), *Kidney complaints* (including Chronic renal failure, Horseshoe kidney, cystic kidney, Kidney trouble, tube damage, stone in the kidney, Nephritis, pyelonephritis, Nephrotic syndrome, Only one kidney, double kidney on right side, Renal TB, Uraemia), *Reproductive system disorders* (including Abscess on breast, mastitis, cracked nipple, Amenorrhea, Damaged testicles, Endometriosis, Gynaecological problems, Hysterectomy (nes), Impotence, infertility, Menopause, Pelvic inflammatory disease/PID (female), Period problems, flooding, pre-menstrual tension/syndrome, Prolapse (nes) if female, Prolapsed womb, Prostrate gland trouble, Turner's syndrome, Vaginitis, vulvitis, dysmenorrhoea) and *Urinary tract infection* (including Cystitis, urine infection).<br>• The Epilepsy LSI is based on the group, *'Epilepsy/fits/convulsion',* including Grand mal, Petit mal, Jacksonian fit, Lennox-Gastaut syndrome, blackouts, febrile convulsions, fit (nes)<br>• The Nervous System LSI is based on the groups:<br>○ *Migraine/headaches* ○ *Other problems of nervous system*, including Abscess on brain, Alzheimer's

For peer review only - http://bmjopen.bmj.com/site/about/guidelines.xhtml

Measure	Details
	disease, Bell's palsy, Brain damage resulting from infection (eg. meningitis,, encephalitis) or injury, Carpal tunnel syndrome, Cerebral palsy (spastic), Degenerative brain disease, Fibromyalgia, Friedreich's Ataxia, GuillainBarre syndrome, Huntington's chorea, Hydrocephalus, microcephaly, fluid on brain, Injury to spine resulting in paralysis, Metachromatic leucodystrophy, Motor neurone disease, Multiple Sclerosis (MS), disseminated sclerosis, Muscular dystrophy, Myalgic encephalomyelitis (ME), Myasthenia gravis, Myotonic dystrophy, Neuralgia, neuritis, Numbness/loss of feeling in fingers, hand, leg etc, Paraplegia (paralysis of lower limbs), Parkinson's disease (paralysis agitans), Partially paralysed (nes), Physically handicapped - spasticity of all limbs, Pins and needles in arm, Post viral syndrome (ME), Removal of nerve in arm, Restless legs, Sciatica, Shingles, Spina bifida, Syringomyelia, Trapped nerve, Trigeminal neuralgia, Teraplegia"<br>○ *Meniere's disease/ear complaints causing balance problems* (including Labryrinthitis,, loss of balance - inner ear, Vertigo).

peer review only

For peer review only - http://bmjopen.bmj.com/site/about/guidelines.xhtml

Appendix 7: Year-by-year trends

This appendix presents the year-by-year trends for all of the variables included in the main paper. The table row labelled 'start v end sig' presents the p-value for testing the null hypothesis that there is no difference between the first and last years in the series (whichever these years are). Note that this will differ from the confidence intervals presented in the main paper as these are grouped into multi-year periods with larger sample sizes and therefore greater precision.

Table 1: Year-to-year trends in cardiovascular health

	High blood pressure LSI	Recent high blood pressure	*Biomarker high blood pressure*	Recent heart attack	IHD stroke LSI	Heart attack symptoms	Mini stroke (TIA) symptoms	Recent angina	*Angina symptoms*
**1994**	2.2%	4.2%	8.4%	1.2%	1.4%	5.5%		1.1%	2.3%
**1995**	2.9%		8.3%		1.5%				
**1996**	3.0%		8.3%		1.5%				
**1997**	3.8%		7.7%		1.4%				
**1998**	3.1%	5.4%	7.0%	1.5%	1.3%	6.5%		1.4%	2.2%
**1999**	3.4%				1.4%				
**2000**	4.0%		6.5%		1.3%				
**2001**	4.5%		7.3%		1.7%				
**2002**	4.3%		6.1%		1.4%				
**2003**	4.5%	7.9%	4.9%	1.3%	1.3%	5.5%	8.1%	1.0%	1.8%
**2004**	4.0%				1.2%				
**2005**	5.0%		4.4%		1.3%				
**2006**	4.4%	8.7%	3.9%	1.1%	1.2%	6.2%	7.8%	0.9%	1.6%
**2007**	4.9%		4.5%		1.0%				
**2008**	5.1%		3.9%		1.1%				
**2009**	4.7%		3.2%		1.3%				
**2010**	4.6%		4.1%		1.1%				
**2011**	4.0%	9.5%	3.2%	1.0%	1.0%	5.2%	6.7%	0.7%	1.2%
**2012**			4.1%						
**2013**			3.7%						
**2014**			3.9%						
**Start v end sig.**	0.00	0.00	0.00	0.14	0.05	0.52	0.01	0.03	0.00
**N**	124,830	43,292	79,601	43,445	124,830	43,521	23,487	43,477	43,518

For peer review only - http://bmjopen.bmj.com/site/about/guidelines.xhtml

Table 12: Year-to-year trends in respiratory health

Year	COPD symptoms	Diagnosed asthma	Asthma LSI	Breathlessness Grade 2+	Breathlessness Grade 3	Recent wheezing/asthma	Wheezing stopping slee
**1994**		10.8%	4.7%				3.6%
**1995**	6.6%		4.8%	19.1%	7.6%	19.8%	
**1996**	6.6%	11.5%	5.3%	20.3%	8.0%	19.3%	3.5%
**1997**		11.9%	6.0%			18.9%	3.7%
**1998**			5.3%				
**1999**			5.7%				
**2000**			5.5%				
**2001**		14.1%	5.9%			19.9%	3.4%
**2002**			6.0%				
**2003**			5.8%				
**2004**			6.3%				
**2005**			6.1%				
**2006**			5.8%				
**2007**			5.7%				
**2008**			6.2%				
**2009**			5.5%				
**2010**	5.1%	16.6%	6.0%	15.4%	6.4%	18.4%	3.2%
**2011**			5.6%				
**2012**							
**2013**							
**2014**							
**Start v end sig.**	0.00	0.00	0.02	0.00	0.01	0.05	0.18
**N**	25,631	41,219	124,830	25,620	25,620	41,218	41,218

For peer review only - http://bmjopen.bmj.com/site/about/guidelines.xhtml

Table 2: Year-to-year trends in activity limitations & musculoskeletal health

Problems walking about today	Walking limitation	Underweight - BMI / Any locomotor limitation	Obese - BMI / washing/dressing Problems today	hip - High waist ratio / Washing/dressing limitation	Recent diabetes - / Any self care limitation	Diabetes any - Pai	extreme LSI - Pai	Glycated haemoglobin / Arthritis LSI	musculoskeletal	Other	LSI
1994	1994	1.1% 6.8%	15.7%	1.9% 9.5%	3.9% 1.2%		1.5%		4.9	%	8.9%
1995	1995	1.1%	17.0%				1.6%			%	9.9%
1996	1996	0.9%	17.3% 9.4%			[illegible]2.0%	1.6% 3.			%	10.3%
1997	1997	0.9%	19.3%	12.1%			1.7%		6.0	%	11.4%
1998	1998	1.0%	19.5%	11.3%	1.4%		1.5%			%	11.7%
1999	1999	1.1%	20.1%	16.3%			1.9%			%	11.0%
2000	2000	0.9% [illegible]%	21.5%				2.0%			%	10.7%
2001	2001	5.9% 0.9% 7.8%	22.8%	15.8% 2.4%	4.7%		2.1%		6.1	%	10.9%
2002	2002	1.0%	23.5%	16.5%			2.1%			%	12.3%
2003	2003	0.9%	23.2% 3.2%	18.7%	2.1%	[illegible]7.1%	2.4% 3.	2.7%		%	11.8%
2004	11.6% 2004	1.0%	24.3% 3.6%			28.6%	2.8% 3.5%		6.3	%	11.6%
2005	2005	0.8%	24.5% 4.0%	21.6%		[illegible]7.8%	2.9% 3.			%	11.3%
2006	2006	0.8%	25.1% 3.6%	20.7%	2.7%	[illegible]6.8%	2.9% 3.	3.1%		%	10.1%
2007	2007	1.0%	25.3%	22.1%			3.4%			%	9.9%
2008	11.5% 2008	0.9%	25.3% 3.6%	22.5%		28.1%	2.9% 3.1%	3.8%	4.7	%	9.5%
2009	2009	1.4%	24.3%	23.5%	3.4%		3.8%	4.3%		%	9.0%
2010	2010	1.1%	27.8% 4.1%	24.3%	3.4%	[illegible]9.9%	3.5% 3.	3.7%		%	10.3%
2011	13.6% 2011	0.8%	25.4% 4.0%	24.3%	3.6%	34.0%	3.8% 4.0%	5.5%	4.9	%	9.2%
2012	2012	1.1%	25.6% 3.8%	24.0%	3.6%	[illegible]7.4%	3.	4.9%			
2013	2013	1.0%	26.8%	24.2%	3.6%			4.8%			
2014	2014	0.8%	27.1% 4.2%	24.7%	3.7%	[illegible]7.7%	3.[illegible]	4.4%			
Start v end sig.	Start v end sig.	0.00 0.01 1.1%	0.05 15.7%	0.04 9.5%	0.01 1.2%	0.00	0.89 1.5%	0.97			0.57
N	N	[illegible]41 1.1%	62,612 17.0%	[illegible]	[illegible]	[illegible]2,692	62,6[illegible] 1.6%	[illegible]30			124,830

Table 14: Year-to-year trends in obesity & diabetes

Web Appendix page 32

Table 3: Year-to-year trends in other biomarkers

	High total cholesterol	Low HDL cholesterol	Raised C-reactive protein	Raised fibrinogen	Anaemia	Iron deficiency	Cancer LSI
**1994**	75.7%				6.7%	39.9%	0.2%
**1995**							0.3%
**1996**							0.3%
**1997**							0.4%
**1998**	64.8%	11.8%	21.4%	2.3%	6.3%	38.2%	0.5%
**1999**							0.4%
**2000**							0.5%
**2001**							0.5%
**2002**							0.5%
**2003**	71.4%	4.0%	24.1%	5.7%			0.6%
**2004**							0.6%
**2005**							0.6%
**2006**	67.2%	5.1%	22.7%	5.7%	4.6%	29.3%	0.7%
**2007**							0.5%
**2008**	66.7%	4.3%					0.6%
**2009**	66.9%	4.5%	23.5%	3.8%	5.3%	27.0%	0.5%
**2010**	64.1%	4.6%					0.8%
**2011**	60.2%	4.5%					0.8%
**2012**	64.0%	4.4%					
**2013**	58.0%	3.4%					
**2014**	55.4%	2.9%					
**Start v end sig.**	0.00	0.00	0.11	0.01	0.04	0.00	0.00
**N**	41,224	33,937	17,749	16,105	20,228	20,304	124,830

	Mental health LSI	Psychological distress symptoms	Anxiety/depression - moderately	Anxiety/depression - extremely
**1994**	1.8%	16.1%		
**1995**	2.3%	18.0%		
**1996**	2.4%		21.9%	1.8%
**1997**	2.9%	16.5%		
**1998**	3.0%	15.6%		
**1999**	3.0%	17.7%		

For peer review only

For peer review only - http://bmjopen.bmj.com/site/about/guidelines.xhtml

Table 16: Year-to-year trends in mental health

**2000**	3.5%	14.4%		
**2001**	3.3%	13.7%		
**2002**	3.1%	16.6%		
**2003**	3.7%	13.5%	18.5%	1.9%
**2004**	3.6%	13.4%	18.8%	2.1%
**2005**	4.4%	14.0%	19.6%	2.1%
**2006**	4.1%	13.9%	18.8%	2.1%
**2007**	4.5%			
**2008**	4.2%	13.7%	18.5%	2.0%
**2009**	4.9%	17.1%		
**2010**	5.2%	16.1%	23.5%	2.7%
**2011**	4.6%		26.8%	3.0%
**2012**		16.0%	20.0%	2.7%
**2013**				
**2014**		15.6%	19.6%	2.5%
**Start v end sig.**	0.00	0.47	0.01	0.02
**N**	124,830	107,834	62,635	62,635

Appendix 8: Others' analyses over change over time using HSE data

Changes over time in some of these indicators have not previously been analysed (e.g. waist-hip ratio, fibrinogen). However, others have been studied but never integrated into a single picture of changing morbidity; we review these in this section. (For reasons of space these are included here rather than in the main text).

Cardiovascular morbidity

1998-2011 trends in the two biomarkers for total and HDL cholesterol using HSE data are shown in Oyebode,[11] who find similar results.

Respiratory morbidity

A subset of the HSE respiratory indicators (ever/past year wheezing, doctor-diagnosed asthma) were analysed by Hall and Mindell[31] looking at 2001-2010, and finding similar changes over time to our analysis. They found stability in some measures (ever wheezing) but improvements in others

For peer review only - http://bmjopen.bmj.com/site/about/guidelines.xhtml

(pastyear wheezing) – at the same time as the reported prevalence of doctor-diagnosed asthma increased.

Obesity & diabetes

While the English trends in waist-hip ratio have not previously been analysed, earlier Scottish trends are given in Hotchkiss et al 2012.[19] Trends in diabetes have been covered in several HSE reports, e.g. Moody 2012,[20] as has BMI (see particularly the paper by Sperrin et al 2014,[32] who also created a publicly-available time-series HSE dataset for this purpose).

Activity limitations, pain & musculoskeletal morbidity

While musculoskeletal LSIs have not previously been analysed in HSE, a decline can also be seen in the General Household Survey.[33]

Mental health

In the UK and most other high-income countries, benefit claims due to mental ill-health have been rising,[34] which has come alongside considerable increases in mental health diagnosis and treatment.[35] The extent to which this reflects rises in mental ill-health and genuinely declining work capacity, however, has long been the subject of debate.[36 37] Perhaps the most robust long-term general population data series in the UK is the Adult Psychiatric Morbidity Survey.[35 38]

While some studies have used HSE to show rises in mental ill-health, others have used the same data to come to the opposite conclusion.[39 40] These contrasting conclusions are explained by the tables in Web Appendix 7 which show year-by-year changes: moderate mental ill-health fell between the mid1990s and the mid-2000s, before rising in 2009, and with a particularly high prevalence in 2011. The conclusions of studies will therefore depend on the years they use as their start and end periods for the trend analysis.[3] It is also worth noting that our results for considerable increases in mental health LSIs can also be seen in a similar measure in the Labour Force Survey.[41 42]

Other morbidity measures

While CRP and fibrinogen are collected in HSE at considerable efforts, their trends have rarely been studied (e.g. they appear only in supplementary descriptive tables in Hughes et al [23]). A decline in anaemia using HSE data 1998-2005 has been observed by Tull et al 2009,[43] but this has not hitherto been updated to the 2008-10 period.

It has been suggested that multimorbidity has risen among older people in England [44] and for all age groups in Ontario,[45] although others have cautioned against using simple disease counts,[46] and the evidence cited in the introduction of the main paper suggests that rising chronic disease reporting may partly be a result of increasing awareness (rather than underlying prevalence) of disease.

---

[3] The major explanation why 'moderate anxiety/depression today' does not show a decline 2011-14 compared to 1994-6 is because of a single very high reported prevalence in 2011, which had reduced by 2012 and 2014.
The alternate measure ('psychological distress symptoms') was not asked in 2011.

For peer review only - http://bmjopen.bmj.com/site/about/guidelines.xhtml

Appendix 9: Summarising multiple measures

Having reviewed trends in 39 morbidity measures, we have seen that morbidity in the English working-age population has improved in some respects and deteriorated in others. For those who view work-related morbidity as intrinsically multidimensional,47, this is the endpoint of our analysis. However, for those who conceive of morbidity as unidimensional – or those who are interested in morbidity as it relates to a unidimensional work capacity – this raises the question of how we weight different dimensions of morbidity to decide if the overall change in morbidity has been positive or negative.

Methods for creating unidimensional morbidity scales

Several methods have been proposed for creating unidimensional morbidity scales, but most of these are unavailable using the HSE data:

- Weights can be based on empirically-derived preferences for different health states, of which the most famous example is the WHO Global Burden of Disease (GBD) study 48. Some GBD estimates for trends in disability in the UK do exist, and suggest that the prevalence of disability in the working-age population is unchanged 1990-2010, though these results are only presented in passing.[4] For our analyses, however, we have no preference-based weights for most of the HSE measures (excluding the subset of measures that make up the EQ-5D scale).
- Those reporting limitations beyond a certain severity in any domain can be categorised as 'disabled', as recommended by the Washington Group on Disability Statistics (see above). However, as previously discussed, we have few functional limitations measures available in HSE.
- Latent morbidity scales can be created based on the inter-correlations between different measures (using item response theory), as used in the World Disability Report 51 and by researchers associated with the US National Bureau of Economic Research e.g. 52. However, it is unclear why we would wish to weight items in this way: a given morbidity indicator may be severe, yet if it is unrelated to other morbidity measures it will be given a low weight.
- Latent morbidity scales can also be created based on the independent correlation between each indicator and a general measure of morbidity, such as general self-reported health or 53 as in 54. This maintains some of the advantages of single-item measures (in providing a basis for making morbidity unidimensional), while avoiding the potential threats to validity discussed above. However, the inconsistent inclusion of measures in each HSE wave prevents a unidimensional morbidity scale being constructed here.

---

[4] Trends in the UK GBD results are reported in Murray et al.[49] However, Murray et al do not focus on trends in years lived with disability (YLD), other than to note that *"YLDs per person by age and sex have not changed substantially in the UK, but age-specific mortality has been improving"* (p1005). The figure in the supplementary appendix shows that YLDs have barely changed for either men or women at any age. However, the confidence intervals for YLDs as a whole in the main paper (Table 1) suggest that the confidence intervals for these trends are very wide. The public GBD data [50] do provide cause-disaggregated YLDs for the UK (and all other countries) for a slightly different period (2000-2015), but are not age-standardised, are within broad age groups only (e.g. 15-29), and again lack estimates of uncertainty.

For peer review only - http://bmjopen.bmj.com/site/about/guidelines.xhtml

An alternative way of summarising heterogeneous trends

Nevertheless, we can examine if the areas in which morbidity has been improving or declining are those that are particularly important for general health.[53] (This uses the same intuition as the scales in Diederichs et al 2012).[54] To see how important measures are for general health, we regress 'bad' general health (see Appendix 5 for detail on the underlying question) on age, sex (and their interaction), educational level and each individual morbidity measure in turn, using all years for which that morbidity measure is available. That is, for each morbidity indicator morbidity we use the following model:

badhealth =logit β morbidity + +β +
!" *male$%+ &'() *+,-

… where β is our primary outcome coefficient showing the importance of that morbidity indicator for bad health, refers to a vector of age dummy variables, male$ refers to a binary gender dummy variable, '() *+, refers to a vector of education dummy variables (with four levels: degree/full-time student, A-levels/NVQ3/higher education below degree, other qualifications, or no qualifications), and , . , !, and & refer to the coefficients on age, gender, their interaction and education respectively.

We adjust for education as well as age & sex to enable us to examine the importance of the measure for bad health, after taking account of whether general health and the measure are both strongly related to social status. Note however that it is not possible to control for all morbidity measures simultaneously (as we discuss just above) – so this is a rough indicator of the importance of that morbidity measure for general health, rather than a reliable indicator of the causal impact net of comorbidities.

The results of this analysis are shown overleaf, ordered by the effect on bad health. (We also repeat the trend in each measure for convenience; this is discussed following the table).

**Measure**	**Type**	**Effect on bad health** *(95% CI)*		**Change over time in measure** *(95% CI)*	
Pain-extreme	S	46.4%	*[44.0, 48.9%]*	0.2%	*[-0.3, 0.7%]*
Problems washing/dressing today	S	43.7%	*[41.4, 46.0%]*	0.3%	*[-0.2, 0.9%]*
Anxiety/depression-extremely	S	35.4%	*[32.8, 38.0%]*	0.9%	*[0.5, 1.3%]*
Any locomotor limitation	S	33.6%	*[31.2, 36.0%]*	0.9%	*[0.1, 1.7%]*
Any self-care limitation	S	32.6%	*[29.7, 35.5%]*	0.7%	*[0.1, 1.3%]*
Problems walking about today	S	26.3%	*[25.2, 27.4%]*	0.4%	*[-0.6, 1.3%]*
High psychological distress	S	26.4%	*[24.9, 27.9%]*	0.9%	*[0.4, 1.4%]*
Recent angina	L	23.8%	*[20.1, 27.5%]*	-0.5%	*[-0.8, -0.1%]*
Recent heart attack/stroke	L	23.2%	*[19.7, 26.7%]*	-0.4%	*[-0.7, 0.0%]*
Breathlessness-Grade 3	S	22.9%	*[20.9, 24.9%]*	-1.6%	*[-2.5, -0.8%]*
Mental health LSI	L	20.4%	*[19.1, 21.7%]*	2.4%	*[1.8, 3.0%]*
IHD/stroke LSI	L	19.7%	*[17.9, 21.5%]*	-0.6%	*[-0.9, -0.2%]*
Wheezing stopping sleep	S	19.1%	*[17.1, 21.1%]*	-0.5%	*[-1.0, 0.1%]*
Mini stroke (TIA) symptoms	S	16.8%	*[15.0, 18.6%]*	-1.4%	*[-2.4, -0.4%]*
Angina symptoms	S	16.6%	*[14.1, 19.1%]*	-1.2%	*[-1.6, -0.7%]*
Psychological distress symptoms	S	15.2%	*[14.6, 15.8%]*	-1.3%	*[-2.4, -0.3%]*
Arthritis LSI	L	15.2%	*[14.3, 16.1%]*	-0.7%	*[-1.4, 0.0%]*
Any recent CVD	L	14.4%	*[12.7, 16.1%]*	0.5%	*[-0.1, 1.2%]*
Heart attack symptoms	S	14.1%	*[12.6, 15.6%]*	-0.5%	*[-1.3, 0.3%]*

For peer review only - http://bmjopen.bmj.com/site/about/guidelines.xhtml

Anxiety/depression-moderately	S	13.6%	*[13.0, 14.2%]*	0.1%	*[-1.1, 1.3%]*
Pain-any	S	12.9%	*[12.4, 13.4%]*	-3.3%	*[-4.6, -2.0%]*
COPD symptoms	S	12.6%	*[11.0, 14.2%]*	-1.6%	*[-2.3, -0.8%]*
Diabetes LSI	L	12.4%	*[11.1, 13.7%]*	2.1%	*[1.5, 2.6%]*
Recent diabetes	L	11.8%	*[10.2, 13.4%]*	2.2%	*[1.9, 2.6%]*
Breathlessness-Grade 2+	S	11.5%	*[10.5, 12.5%]*	-4.8%	*[-6.1, -3.5%]*
Any CVD LSI	L	11.0%	*[10.3, 11.7%]*	0.6%	*[-0.1, 1.4%]*
Other musculoskeletal LSI	L	9.8%	*[9.2, 10.4%]*	-0.8%	*[-1.7, 0.1%]*
Glycated haemoglobin	B	9.9%	*[7.9, 11.9%]*	2.1%	*[1.4, 2.7%]*
Asthma LSI	L	8.6%	*[7.8, 9.4%]*	0.7%	*[0.0, 1.4%]*
Recent wheezing/asthma	S	8.4%	*[7.7, 9.1%]*	-1.2%	*[-2.5, 0.1%]*
Recent high blood pressure	L	6.8%	*[5.7, 7.9%]*	4.8%	*[3.9, 5.6%]*
BMI-Underweight	B	6.2%	*[4.3, 8.1%]*	-0.1%	*[-0.3, 0.1%]*
Diagnosed asthma	L	5.9%	*[5.1, 6.7%]*	5.7%	*[4.5, 6.8%]*
High waist-hip ratio	B	4.6%	*[4.1, 5.1%]*	14.1%	*[13.0, 15.2%]*
Raised fibrinogen	B	4.3%	*[1.9, 6.7%]*	1.5%	*[0.3, 2.6%]*
Low HDL cholesterol	B	4.3%	*[2.8, 5.8%]*	-8.0%	*[-9.0, -7.1%]*
Raised C-reactive protein	B	3.7%	*[2.7, 4.7%]*	1.9%	*[-0.7, 4.5%]*
BMI-Obese	B	2.8%	*[2.5, 3.1%]*	8.9%	*[8.0, 9.7%]*
Anaemia	B	2.4%	*[0.8, 4.0%]*	-1.4%	*[-2.7, -0.1%]*
Biomarker high blood pressure	B	0.4%	*[-0.3, 1.1%]*	-5.0%	*[-5.6, -4.5%]*
High total cholesterol	B	0.0%	*[-0.6, 0.6%]*	-17.6%	*[-19.1, -16.1%]*
Iron deficiency	B	-0.5%	*[-1.3, 0.3%]*	-12.5%	*[-14.8, -10.2%]*

Having estimated this, we can see if the areas in which morbidity has been improving or declining are those that are particularly important for general health. This is shown visually in Figure 1 below (the measures are not labelled to enable the overall pattern to be seen, but the top-to-bottom order of measures is the same in the figure as in the preceding table; i.e. the measure at the top of the figure is 'Pain-extreme').

For peer review only - http://bmjopen.bmj.com/site/about/guidelines.xhtml

Figure 1: Change over time in morbidity measures & their association with bad general health[a]

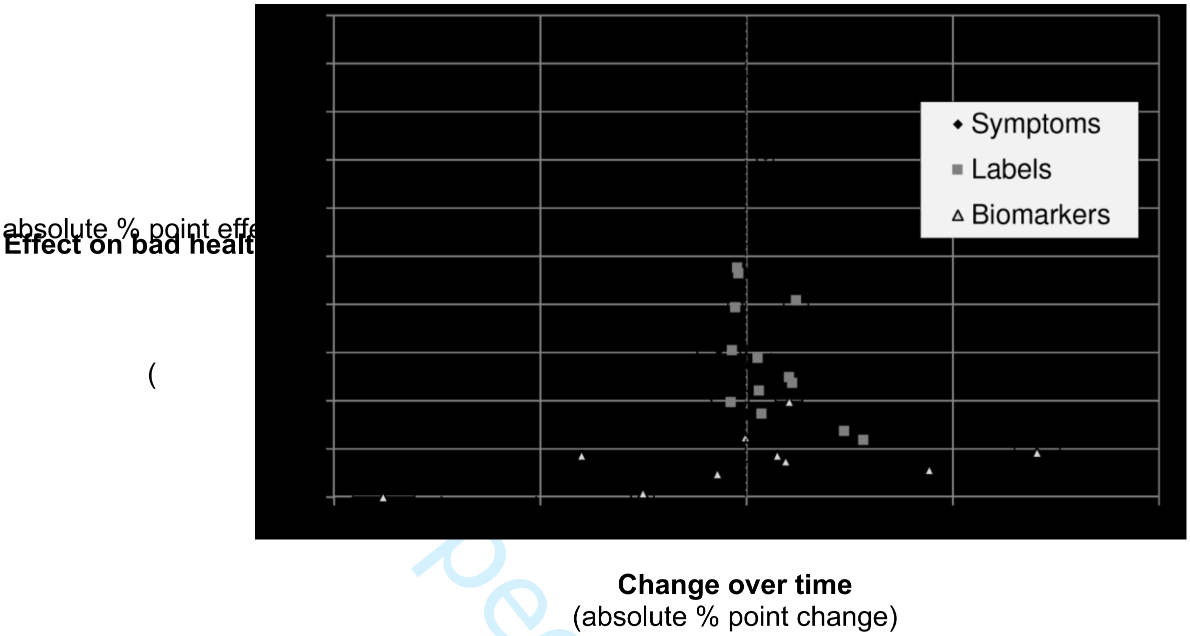

[a] 'Trend' is as reported above in the main paper. 'Effect on bad health' shows the effect of the morbidity measure on (very) bad health after controlling for age, sex (and their interaction) and educational level, using all years for which the individual morbidity measure is available. (This shows average marginal effects following a logistic regression; see text above).

It is easiest to interpret the figure by focussing on each group of measures in turn. Firstly, the biomarkers tend to have the weakest relationship with general health. Those with high levels of the diabetes biomarker (glycated haemoglobin) are 9.7% more likely to say they have bad health, and those who are underweight, with a high waist-hip ratio, raised fibrinogen, or low HDL cholesterol are 4-6% more likely to report bad health, but the other measures only had weaker relationships. Indeed, there was effectively no relationship between bad reported health and any of measured high blood pressure, high total cholesterol or iron deficiency.

Secondly, most of the measures based on medical labels have a moderately strong relationship with bad health (the weakest being lifetime asthma and recent high blood pressure, both of which can be asymptomatic), and these measures have mostly risen over time. There are however notable exceptions to this, including IHD/stroke LSI, recent angina and recent heart attack/stroke (the labelbased measures with some of the strongest relationships with bad reported health), as well as arthritis and other musculoskeletal LSIs.

Finally, symptom-based measures unsurprisingly tend to have stronger relationships with bad reported health, although this ranges from the moderate (those reporting 'recent wheezing/asthma attack' were 8.5% more likely to report bad health) to the very strong (those reporting 'extreme pain today' were 46.4% more likely to report bad health). In general, those symptoms-based measures with the strongest relationship with bad reported health were more likely to have increased over time ('extreme anxiety/depression today', 'locomotor limitations', and 'self-care limitations'). However, the size of the aforementioned declines in symptom-based measures of respiratory and cardiovascular morbidity was often greater.

For peer review only - http://bmjopen.bmj.com/site/about/guidelines.xhtml

For peer review only

For peer review only - http://bmjopen.bmj.com/site/about/guidelines.xhtml

Bibliography for Web Appendices

1. Case A, Deaton A. Rising morbidity and mortality in midlife among white non-Hispanic Americans in the 21st century. *Proceedings of the National Academy of Sciences* 2015;112(49):15078-83. doi: 10.1073/pnas.1518393112
2. Hiam L, Dorling D, Harrison D, et al. Why has mortality in England and Wales been increasing? An iterative demographic analysis. *Journal of the Royal Society of Medicine* 2017;110(4):153-62. doi: 10.1177/0141076817693599
3. Department of Health. Our Health and Wellbeing Today. London: HM Government, 2010.
4. Jagger C. Trends in life expectancy and healthy life expectancy. Future of an ageing population: evidence review. London: Foresight, Government Office for Science, 2015.
5. Office for Budget Responsibility. Welfare trends report: October 2016. Cm 9341. London: Her Majesty's Stationery Office, 2016.
6. Department for Work and Pensions, Department of Health. Improving Lives: The Work, Health and Disability Green Paper. Cm 9342. London: Her Majesty's Stationery Office, 2016.
7. Geiger BB. Morbidity in England 1994-2014 2019 [Available from:http://osf.io/dy6sv].
8. Szende A, Janssen B, Cabases J, editors. *Self-Reported Population Health: An International Perspective based on EQ-5D*. Netherlands: Springer, 2014.
9. Erens B, Primatesta P, Prior G. Health survey for England 1999: the health of minority ethnic groups. London: The Stationery Office, 2001.
10. National Heart Lung and Blood Institute. Sixth Report of the Joint National Committee on the Prevention, Detection, Evaluation and Treatment of High Blood Pressure. NIH Publication No 98-04080: National Institutes on Health, 1997.
11. Oyebode O. Cardiovascular disease. In: Craig R, Mindell J, eds. Health Survey for England 2011: Volume 1 - Health, social care and lifestyles. Leeds, UK: Health and Social Care Information Centre 2012.
12. Banks J, Marmot M, Oldfield Z, et al. The SES Health Gradient on Both Sides of the Atlantic. NBER Working Paper No 12674, 2006.
13. Lawlor DA, Adamson J, Ebrahim S. Performance of the WHO Rose angina questionnaire in postmenopausal women: Are all of the questions necessary? *Journal of Epidemiology and Community Health* 2003;57(7):538-41. doi: 10.1136/jech.57.7.538
14. Cook D, Shaper A, Macfarlane P. Using the WHO (Rose) Angina Questionnaire in Cardiovascular Epidemiology. *International Journal of Epidemiology* 1989;18(3):607-13. doi: 10.1093/ije/18.3.607
15. Fletcher C, Elmes P, Fairbairn M, et al. The significance of respiratory symptoms and the diagnosis of chronic bronchitis in a working population. *BMJ* 1959;2:257-66.
16. WHO. Physical status: the use and interpretation of anthropometry. Report of a WHO Expert Committee. WHO Technical Report Series 854. Geneva: World Health Organization (WHO), 1995.
17. National Obesity Observatory. Obesity and Overweight Surveillance in England: what is measured and where are the gaps?, 2009.
18. NICE. Obesity: The Prevention, Identification, Assessment and Management of Overweight and Obesity in Adults and Children NICE Clinical Guidelines, No 43. London: Centre for Public Health Excellence and National Collaborating Centre for Primary Care at the National Institute for Health and Clinical Excellence (NICE), 2006.

For peer review only

For peer review only - http://bmjopen.bmj.com/site/about/guidelines.xhtml

19. Hotchkiss JW, Davies CA, Gray L, et al. Trends in cardiovascular disease biomarkers and their socioeconomic patterning among adults in the Scottish population 1995 to 2009: crosssectional surveys. *BMJ Open* 2012;2(3) doi: 10.1136/bmjopen-2011-000771
20. Moody A. Diabetes and hyperglycaemia. In: Craig R, Mindell J, eds. Health Survey for England 2011: Volume 1 - Health, social care and lifestyles. Leeds, UK: Health and Social Care Information Centre 2012.
21. Aresu M, Gordon-Dseagu V, Shelton N. Diabetes and glycaemia. In: Craig R, Hirani V, eds. Health Survey for England 2009, Volume 1: Health and lifestyles. Leeds, UK: The NHS Information Centre for health and social care 2010:59-74.
22. Steptoe A. Psychosocial biomarker research: integrating social, emotional and economic factors into population studies of aging and health. *Social Cognitive and Affective Neuroscience* 2011;6(2):226-33. doi: 10.1093/scan/nsq032
23. Hughes A, McMunn A, Bartley M, et al. Elevated inflammatory biomarkers during unemployment: modification by age and country in the UK. *Journal of Epidemiology and Community Health* 2015;69(7):673-79. doi: 10.1136/jech-2014-204404
24. Benzeval M, Davillas A, Kumari M, et al. Understanding Society: The UK Household Longitudinal Study Biomarker User Guide and Glossary (version 1). Colchester, Essex: Institute for Social and Economic Research, 2014.
25. Chaudhury M. Blood analytes. In: Sproston K, Primatesta P, eds. Health Survey for England, 2003, Vol 2: Risk Factors for Cardiovascular Disease: TSO 2004:241-88.
26. Erens B, Primatesta P. Health survey for England 1998: cardiovascular disease. *London: The Stationery Office* 1999
27. Chaudhury M, Tull K. Nutrition and haematological status. In: Craig R, Mindell J, eds. Health Survey for England, 2005: The health of older people, Vol 1: General health and function: TSO 2006:67-96.
28. Goldberg D, PA W. User Guide to the General Health Questionnaire. Windsor, UK: NFERNelson, 1988.
29. Stochl J, Böhnke JR, Pickett KE, et al. An evaluation of computerized adaptive testing for general psychological distress: combining GHQ-12 and Affectometer-2 in an item bank for public mental health research. *BMC Medical Research Methodology* 2016;16(1):58. doi: 10.1186/s12874-016-0158-7
30. ONS. Harmonised Concepts and Questions for Social Data Sources, Primary Principles: Longlasting Health Conditions and Illnesses; Impairments and Disability [version 1.1]. London: Office for National Statistics (ONS), 2015.
31. Hall J, Mindell J. Respiratory symptoms and disease in adults In: Craig R, Mindell J, eds. Health Survey for England, 2010, Volume 1: Respiratory health: TSO 2011.
32. Sperrin M, Marshall A, Higgins V, et al. Slowing down of adult body mass index trend increases in England: a latent class analysis of cross-sectional surveys (1992-2010). *International Journal of Obesity* 2014;38(6)
33. Parsons S, Ingram M, Clarke-Cornwell AM, et al. A Heavy Burden: The occurrence and impact of musculoskeletal conditions in the United Kingdom today. Manchester: Arthritis Research UK & University of Manchester, 2011.
34. OECD. Fit Mind, Fit Job: From evidence to practice in mental health and work. Paris: OECD, 2015.
35. Spiers N, Qassem T, Bebbington P, et al. Prevalence and treatment of common mental disorders in the English national population, 1993–2007. *The British Journal of Psychiatry* 2016;209(2):150-56. doi: 10.1192/bjp.bp.115.174979

For peer review only - http://bmjopen.bmj.com/site/about/guidelines.xhtml

36. Moncrieff J, Pomerleau J. Trends in sickness benefits in Great Britain and the contribution of mental disorders. *Journal of Public Health Medicine* 2000;22:59-67.
37. Stansfeld SA, Woodley-Jones D, Rasul F, et al. Work-related distress in the 1990s - a real increase in ill health? . *Journal of Public Mental Health* 2008;7(1):25-31.
38. McManus S, Bebbington P, Jenkins R, et al., editors. *Mental health and wellbeing in England: Adult Psychiatric Morbidity Survey 2014*. Leeds: NHS Digital, 2016.
39. Maheswaran H, Kupek E, Petrou S. Self-reported health and socio-economic inequalities in England, 1996–2009: Repeated national cross-sectional study. *Social Science & Medicine* 2015;136–137:135-46. doi: http://dx.doi.org/10.1016/j.socscimed.2015.05.026
40. Katikireddi SV, Niedzwiedz CL, Popham F. Trends in population mental health before and after the 2008 recession: a repeat cross-sectional analysis of the 1991–2010 Health Surveys of England. *BMJ Open* 2012;2(5) doi: 10.1136/bmjopen-2012-001790
41. Jones M, Wass V. Understanding changing disability-related employment gaps in Britain 1998–2011. *Work, Employment & Society* 2013;27(6):982-1003. doi: 10.1177/0950017013475372
42. Barr B, Kinderman P, Whitehead M. Trends in mental health inequalities in England during a period of recession, austerity and welfare reform 2004 to 2013. *Social Science & Medicine* 2015;147:324-31. doi: http://dx.doi.org/10.1016/j.socscimed.2015.11.009
43. Tull KI, Hirani V, Ali A, et al. Impact of different diagnostic thresholds and the anaemia–ferritin–transferrin receptor model on the prevalence of anaemia and impaired iron status in older people. *Age and Ageing* 2009;38(5):609-13. doi: 10.1093/ageing/afp102
44. Dhalwani NN, O'Donovan G, Zaccardi F, et al. Long terms trends of multimorbidity and association with physical activity in older English population. *International Journal of Behavioral Nutrition and Physical Activity* 2016;13(1):8. doi: 10.1186/s12966-016-0330-9
45. Koné Pefoyo AJ, Bronskill SE, Gruneir A, et al. The increasing burden and complexity of multimorbidity. *BMC Public Health* 2015;15(1):415. doi: 10.1186/s12889-015-1733-2
46. Tetzlaff J, Junius-Walker U, Muschik D, et al. Identifying time trends in multimorbidity—defining multimorbidity in times of changing diagnostic practices. *Journal of Public Health* 2016:1-8. doi: 10.1007/s10389-016-0771-2
47. Marfeo EE, Haley SM, Jette AM, et al. Conceptual Foundation for Measures of Physical Function and Behavioral Health Function for Social Security Work Disability Evaluation. *Archives of Physical Medicine and Rehabilitation* 2013;94(9):1645-52.e2. doi: 10.1016/j.apmr.2013.03.015
48. Salomon JA, Vos T, Hogan DR, et al. Common values in assessing health outcomes from disease and injury: disability weights measurement study for the Global Burden of Disease Study 2010. *The Lancet* 2012;380(9859):2129-43. doi: http://dx.doi.org/10.1016/S01406736(12)61680-8
49. Murray CJL, Richards MA, Newton JN, et al. UK health performance: findings of the Global Burden of Disease Study 2010. *The Lancet* 2013;381(9871):997-1020. doi: https://doi.org/10.1016/S0140-6736(13)60355-4
50. WHO. Global Health Estimates 2015: Disease burden by Cause, Age, Sex, by Country and by Region, 2000-2015. Geneva: World Health Organization (WHO), 2016.
51. WHO. World report on disability. Geneva: World Health Organization (WHO) 2011.
52. Soldo BJ, Mitchell OS, Tfaily R, et al. Cross-cohort differences in health on the verge of retirement. NBER Working Paper No 12762: National Bureau of Economic Research, 2006.
53. Stewart ST, Cutler DM, Rosen AB. Comparison of Trends in U.S. Health-Related Quality of Life over the 2000's Using the SF-6D, HALex, EQ-5D, and EQ-5D Visual Analog Scale versus a Broader Set of Symptoms and Impairments. *Medical care* 2014;52(12):1010-16. doi: 10.1097/MLR.0000000000000181

For peer review only - http://bmjopen.bmj.com/site/about/guidelines.xhtml

54. Diederichs CP, Wellmann J, Bartels DB, et al. How to weight chronic diseases in multimorbidity indices? Development of a new method on the basis of individual data from five populationbased studies. *Journal of Clinical Epidemiology* 2012;65(6):679-85. doi: 10.1016/j.jclinepi.2011.11.006

For peer review only

For peer review only - http://bmjopen.bmj.com/site/about/guidelines.xhtml

Reporting checklist for cross sectional study.

Based on the STROBE cross sectional guidelines.

Instructions to authors

Complete this checklist by entering the page numbers from your manuscript where readers will find each of the items listed below.

Your article may not currently address all the items on the checklist. Please modify your text to include the missing information. If you are certain that an item does not apply, please write "n/a" and provide a short explanation.

Upload your completed checklist as an extra file when you submit to a journal.

In your methods section, say that you used the STROBE cross sectionalreporting guidelines, and cite them as:

von Elm E, Altman DG, Egger M, Pocock SJ, Gotzsche PC, Vandenbroucke JP. The Strengthening the Reporting of Observational Studies in Epidemiology (STROBE) Statement: guidelines for reporting observational studies.

Author note: pages within the online Appendices are denoted by the prefix 'A'. Pages refer to submitted version with tracked changes visible.

For peer review only

		Reporting Item	Page Number
**Title and abstract**			
Title	#1a	Indicate the study's design with a commonly used term in the title or the abstract	1, 3
Abstract	#1b	Provide in the abstract an informative and balanced summary of what was done and what was found	3-4
**Introduction**			
Background / rationale	#2	Explain the scientific background and rationale for the investigation being reported	5-6
Objectives	#3	State specific objectives, including any prespecified hypotheses	5-10
**Methods**			
Study design	#4	Present key elements of study design early in the paper	6-14
Setting	#5	Describe the setting, locations, and relevant dates, including periods of recruitment, exposure, follow-up, and data collection	6-7, A6-9

Eligibility criteria	#6a	Give the eligibility criteria, and the sources and methods of selection of participants.	6-7, A6-9
	#7	Clearly define all outcomes, exposures, predictors, potential confounders, and effect modifiers. Give diagnostic criteria, if applicable	8-12, A12-23
Data sources / measurement	#8	For each variable of interest give sources of data and details of methods of assessment (measurement). Describe comparability of assessment methods if there is more than one group. Give information separately for for exposed and unexposed groups if applicable.	8-12, A12-23
Bias	#9	Describe any efforts to address potential sources of bias	6-14, 22, A6-9, A12-23
Study size	#10	Explain how the study size was arrived at	6, A9
Quantitative variables	#11	Explain how quantitative variables were handled in the analyses. If applicable, describe which groupings were chosen, and why	A12-23
Statistical methods	#12a	Describe all statistical methods, including those used to control for confounding	13-14
Statistical methods	#12b	Describe any methods used to examine subgroups and interactions	n/a
Statistical methods	#12c	Explain how missing data were addressed	A3-9
Statistical methods	#12d	If applicable, describe analytical methods taking account of sampling strategy	13-14
Statistical methods	#12e	Describe any sensitivity analyses	A10-11, A24-34
**Results**			
Participants	#13a	Report numbers of individuals at each stage of study—eg numbers potentially eligible, examined for eligibility, confirmed eligible, included in the study, completing follow-up, and analysed. Give information separately for for exposed and unexposed groups if applicable.	A3-A9
Participants	#13b	Give reasons for non-participation at each stage	A3-A9
Participants	#13c	Consider use of a flow diagram	n/a
Descriptive data	#14a	Give characteristics of study participants (eg demographic, clinical, social) and information on exposures and potential confounders. Give information separately for exposed and unexposed groups if applicable.	n/a [this is a descriptive study]

For peer review only

Descriptive data	#14b	Indicate number of participants with missing data for each variable of interest	A3-A9
Outcome data	#15	Report numbers of outcome events or summary measures. Give information separately for exposed and unexposed groups if applicable.	n/a [these form the main results]
Main results	#16a	Give unadjusted estimates and, if applicable, confounder-adjusted estimates and their precision (eg, 95% confidence interval). Make clear which confounders were adjusted for and why they were included	13-14, 15-21
Main results	#16b	Report category boundaries when continuous variables were categorized	A12-23
Main results	#16c	If relevant, consider translating estimates of relative risk into absolute risk for a meaningful time period	n/a [all estimates are given as absolute percentages]
Other analyses	#17	Report other analyses done—e.g., analyses of subgroups and interactions, and sensitivity analyses	A10-11, A24-34
**Discussion**			
Key results	#18	Summarise key results with reference to study objectives	21-23
Limitations	#19	Discuss limitations of the study, taking into account sources of potential bias or imprecision. Discuss both direction and magnitude of any potential bias.	21-22
Interpretation	#20	Give a cautious overall interpretation considering objectives, limitations, multiplicity of analyses, results from similar studies, and other relevant evidence.	21-23
Generalisability	#21	Discuss the generalisability (external validity) of the study results	21-23, A6-A9
**Other Information**			
Funding	#22	Give the source of funding and the role of the funders for the present study and, if applicable, for the original study on which the present article is based	1, A43

None The STROBE checklist is distributed under the terms of the Creative Commons Attribution License CC-BY. This checklist can be completed online using https://www.goodreports.org/, a tool made by the EQUATOR Network in collaboration with Penelope.ai

For peer review only
